# Large-Scale Investigation of Human TF-miRNA Relations Based on Coexpression Profiles

**DOI:** 10.1155/2014/623078

**Published:** 2014-06-09

**Authors:** Chia-Hung Chien, Yi-Fan Chiang-Hsieh, Ann-Ping Tsou, Shun-Long Weng, Wen-Chi Chang, Hsien-Da Huang

**Affiliations:** ^1^Institute of Tropical Plant Sciences, National Cheng Kung University, Tainan 701, Taiwan; ^2^Department of Biotechnology and Laboratory Science in Medicine, National Yang-Ming University, Taipei 112, Taiwan; ^3^Mackay Medicine, Nursing and Management College, Taipei 112, Taiwan; ^4^Department of Medicine, Mackay Medical College, New Taipei City 252, Taiwan; ^5^Department of Obstetrics and Gynecology, Hsinchu Mackay Memorial Hospital, Hsinchu 300, Taiwan; ^6^Institute of Bioinformatics and Systems Biology, National Chiao Tung University, Hsinchu 300, Taiwan; ^7^Department of Biological Science and Technology, National Chiao Tung University, Hsinchu 300, Taiwan; ^8^Department of Biomedical Science and Environmental Biology, Kaohsiung Medical University, Kaohsiung 807, Taiwan; ^9^Institute of Biomedical Engineering, National Chiao Tung University, Hsinchu 300, Taiwan

## Abstract

Noncoding, endogenous microRNAs (miRNAs) are fairly well known for regulating gene expression rather than protein coding. Dysregulation of miRNA gene, either upregulated or downregulated, may lead to severe diseases or oncogenesis, especially when the miRNA disorder involves significant bioreactions or pathways. Thus, how miRNA genes are transcriptionally regulated has been highlighted as well as target recognition in recent years. In this study, a large-scale investigation of novel *cis*- and *trans*-elements was undertaken to further determine TF-miRNA regulatory relations, which are necessary to unravel the transcriptional regulation of miRNA genes. Based on miRNA and annotated gene expression profiles, the term “coTFBS” was introduced to detect common transcription factors and the corresponding binding sites within the promoter regions of each miRNA and its coexpressed annotated genes. The computational pipeline was successfully established to filter redundancy due to short sequence motifs for TFBS pattern search. Eventually, we identified more convinced TF-miRNA regulatory relations for 225 human miRNAs. This valuable information is helpful in understanding miRNA functions and provides knowledge to evaluate the therapeutic potential in clinical research. Once most expression profiles of miRNAs in the latest database are completed, TF candidates of more miRNAs can be explored by this filtering approach in the future.

## 1. Introduction

Among functional noncoding RNAs (ncRNAs), microRNAs (miRNAs) are tiny molecules (~21–23 nt) with giant roles. miRNAs participate in gene regulation by targeting messenger RNAs (mRNAs) and influencing their stability and the initiation of translation. It is implied that if the expression of miRNA is aberrant, miRNA-mediated gene circuitries will be disordered, resulting in homeostatic imbalance, pathogenesis, and oncogenesis [1, 2]. In recent years, elucidating transcriptional regulatory mechanisms of miRNA genes has been highlighted when studying miRNA function. Core promoters of miRNA genes were promptly identified for depicting full-length primary transcripts [3–5]. High-throughput sequencing datasets derived from epigenetic signature and TSS-relevant experiments unfold transcriptional start sites (TSSs) of miRNAs and offer a practical strategy to determine miRNA promoters [6–9].

To further understand upstream regulatory elements controlling miRNA expression, transcription factors (TFs) and their binding sites of miRNA promoters were deciphered by either literature survey or computational prediction [10–13]. By integrating the information of TF-miRNA regulatory relations and miRNA target interactions, regulatory networks that revolved around miRNAs provide a biological insight into how miRNAs dominate functional processes in biochemical reactions or metabolic pathways [14–17]. However, most of the foregoing studies regarded the upstream regions of pre-miRNAs as promoters (e.g., 10 kb upstream or −900~ +100 of the 5′-start of the pre-miRNA). More convinced miRNA promoters should be used for searching putative TF-binding sites. In addition, the experimentally verified TF-miRNA relations are insufficient for current miRNAs. According to the statistics in the latest version 1.2 of TransmiR, only 735 entries, which include ~201 transcriptional factors, ~209 miRNAs, and 16 organisms from 268 publications, were curated. A large-scale investigation of novel* cis*- and* trans*-elements is necessary to fulfill the unmet need for locating transcription factor binding sites (TFBSs) within miRNA promoter regions.

Since sequence-specific TFs possess DNA-binding domains (DBDs) to recognize specific motifs in miRNA promoter sequences, potential binding sites can be detected by sequence-based computational approaches, for example, position weight matrix (PWM). A position weight matrix (also called position specific scoring matrix, PSSM) infers a pattern of DNA segment and is widely applied in searching TFBSs [18–21]. Two well-known databases collecting matrix information are TRANSFAC [22] and JASPAR [23]. To avoid excess false positives due to short sequence motifs used for TFBS pattern search,* cis*-regulatory analysis of coregulated gene sets was executed to determine overrepresented TFBSs [24, 25]. Although previously published web server Pscan [26] and oPOSSUM [27] can search transcription factor binding sites in coexpressed gene promoters to remit the impact of false positive issue, users have to define their coexpressed gene groups with valid gene IDs. In addition, miRNA promoter sequences are not included in these two web servers.

Therefore, the purpose of this study is to create a computational pipeline to undertake large-scale investigation of novel* cis*- and* trans*-elements for human miRNA genes based on coexpression strategy. First, we constructed 255 coexpressed gene groups of human miRNAs. Moreover, instead of grapping the upstream regions of pre-miRNAs as promoter sequences, we exploited more concrete human miRNA promoters by following the processes as previously described [7]. Through the detection of transcription factor binding sites within promoter regions of human miRNAs and their coexpressed genes by using matrix information from TRANSFAC, the common TFBSs were identified. We then filtered the redundancy by not only the occurrence of common TFBSs but also the expression correlation between TF-encoded genes and corresponding human miRNA gene groups. Finally, more reliable TF-miRNA regulatory relations of 225 human miRNAs were provided. Furthermore, the liver-specific hsa-miR-122 was also selected as the case study to demonstrate the usage of this filtering approach and its practicability.

## 2. Materials and Methods

### 2.1. Human miRNA Promoter Sequences

The latest genomic coordinates of human miRNAs were retrieved from miRBase release 19 [28]. Human miRNA TSSs were identified by reproducing the computational procedures described in miRStart resource [7]. The formula which determines tag-enriched loci was modified by weighted tag density (tag density multiply by tag number) in the SVM step to precisely reveal the effect of tag intensity. Then, 1 kb upstream sequences of human miRNA TSSs were acquired from UCSC Genome Browser [29] (hg19/GRCh37 assembly) in FASTA format. The updated intergenic miRNA TSSs in human genome are listed in supplementary Table S1 (available online at http://dx.doi.org/10.1155/2014/623078) in additional file for reference.

### 2.2. Expression Profiles of Human miRNAs and Annotated Genes

In order to determine human genes that are coexpressed with specific miRNA genes, GDS596 record, the Affymetrix gene expression profiles from 79 physiologically human normal tissues, was downloaded from Gene Expression Omnibus (GEO) [30, 31]. Among annotated human genes in GDS596, genes were filtered out if their HGNC symbols are invalid or have been withdrawn. On the other hand, the expression data of 345 miRNAs in 40 normal human tissues generated by a new type of real time reverse transcription- (RT-) PCR-based miRNA assays were also collected [32]. Mature miRNAs with eliminated miRBase IDs (release 19) were discarded. In both expression datasets, only 17 tissues (adrenal, brain, heart, kidney, liver, lung, lymph node, ovary, pancreas, placenta, prostate, skeletal muscle, testicle, trachea, thymus, thyroid, and uterus) are in common and were considered to be integrated expression profiles of annotated genes and miRNA genes with 17 conditions. To standardize expression levels across extensive range of both datasets, the raw intensity was *Z*-score transformed [33].

### 2.3. Coexpressed Gene Groups of Human miRNAs

After the transformation process between two expression datasets, Pearson's correlation coefficient (PCC) was calculated to estimate which annotated genes are coexpressed with specific miRNAs. The formula of Pearson's correlation coefficient (usually using the letter *r*) is as follows:

(1) $$\begin{array}{c} r_{xy}=\frac{\sum_{i=1}^{n}\left(X_{i}-\overset{-}{X}\right)\left(Y_{i}-\overset{-}{Y}\right)}{\sqrt{\sum_{i=1}^{n}{\left(X_{i}-\overset{-}{X}\right)}^{2}}\sqrt{\sum_{i=1}^{n}{\left(Y_{i}-\overset{-}{Y}\right)}^{2}}}, \end{array}$$

where *X*_*i*_ and *Y*_*i*_ denote the expression level of *i* condition (tissue) of two genes *x* and *y* that are calculated for  *r*, whereas $\overset{-}{X}$ and $\overset{-}{Y}$ represent the average of corresponding expression levels in total *n* conditions. Since the value of Pearson's correlation coefficient ranges from +1 (positively correlated) to −1 (negatively correlated), we defined that two genes of interest are coexpressed if their Pearson's correlation coefficient is more than 0.8. Subsequently, coexpressed genes of human miRNAs were determined, and their promoter sequences (1 kb upstream from TSS) were obtained from BioMart of Ensembl release 69 [34].

### 2.4. CoTFBSs in Human miRNA Promoters

In this work, we defined* coTFBS* as a common TFBS located in promoter regions of coexpressed genes. The TRANSFAC database [22], comprising data on transcription factors, their target genes, and regulatory binding sites, has been widely used when studying eukaryotic transcriptional regulation. After acquiring 1 kb promoter sequences of miRNA genes and their coexpressed genes in FASTA format, the Match program [35] was executed for sequence motif search of transcription factor binding sites according to the matrix information provided by TRANSFAC (version 2011.4). A customized profile which specified human matrices in TRANSFAC library was used for Match to minimize both false positive and false negative rates with core similarity and matrix similarity cut-off values for each matrix. As defined in the publication of Match, the core of each matrix refers to the first five most conserved consecutive positions of a matrix. A putative TFBS with core similarity less than 1 was filtered out. The occurrence of each coTFBS and its expression correlation with miRNA in coexpressed gene groups was calculated finally. Only those whose encoded genes are coexpressed with corresponding miRNA were considered.

## 3. Results

### 3.1. Human miRNAs with Coexpressed Gene Group for Detecting TF-miRNA Relations

**Figure 1:**
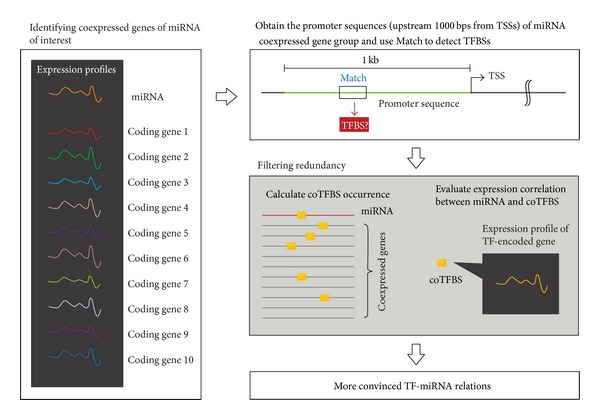
The summary of detecting TF-miR regulatory relations in human genome.

Figure 1 summarizes the workflow to investigate human TF-miRNA regulatory relations based on expression profiles of miRNA and annotated genes. Pearson's correlation coefficient (PCC) was applied to measure the similarity of expression patterns across 17 human normal tissues, which represents the coexpressed level between a specific miRNA gene and an annotated gene of interest. Due to the reason that Pearson's *r* cannot be calculated if the expression levels in all 17 conditions are identical, 29 mature miRNAs with such expression profiles were excluded. After discarding the mature miRNAs whose miRBase IDs are eliminated, 289 human miRNAs were selected to estimate PCC values with annotated human genes in GDS596 record.

Among them, however, only 255 human miRNAs have more than one coexpressed genes (PCC > 0.8). Moreover, 25 out of 255 human miRNAs have no identified TSSs by following the previous strategy [7] and have to be filtered. In total, 230 human miRNAs were qualified to discover putative* cis*- and* trans*-elements in their promoter regions. The number of members of each miRNA coexpressed gene group ranges from several to thousand (see Table S2 in additional file for details).

### 3.2. Putative TF-miRNA Relations Were Explored according to the Occurrence of CoTFBS

**Figure 2:**
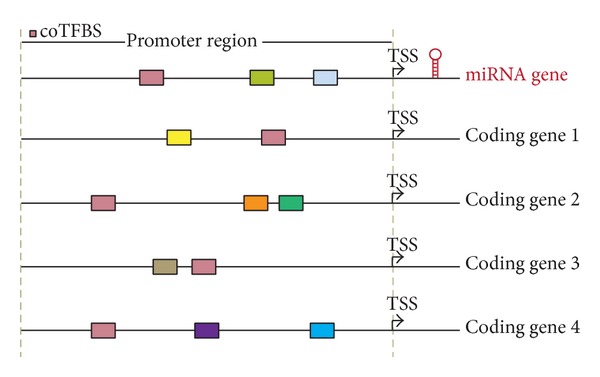
The concept of coTFBS. Protein-coding genes 1, 2, 3, and 4 are coexpressed with the miRNA gene. Colored rectangles represent transcription factor binding motifs in each promoter region. The common TFBS (pink) located within the promoters of miRNA gene and other four genes represents the “coTFBS” of this gene group.

Theoretically, a group of genes that are coexpressed may be regulated by common transcription factors. Based on this concept, a specific transcription factor binding site located in the promoter regions of most genes in each coexpressed group implies that its corresponding TF is the most possible one controlling the expression of these genes. Here, we introduce the term “coTFBS” which represents the common TFBS of a coexpressed gene group to filter redundant TF candidate of miRNA genes. For example, the “pink rectangle” binding motifs were detected in the proposed miRNA and other four gene promoters in Figure 2. The occurrence of this coTFBS is five. By scanning TFBSs in promoter regions of each miRNA gene and its coexpressed gene group using Match based on TRANSFAC library (version 2011.4) and following the filtering process, the putative TFs that regulate a specific miRNA were determined. In this work, we successfully identified putative TF-miRNA relations of 225 human miRNAs. The full list of putative TFs for each human miRNA promoter can be accessed in Table S3.

### 3.3. Case Study: hsa-miR-122

**Figure 3:**
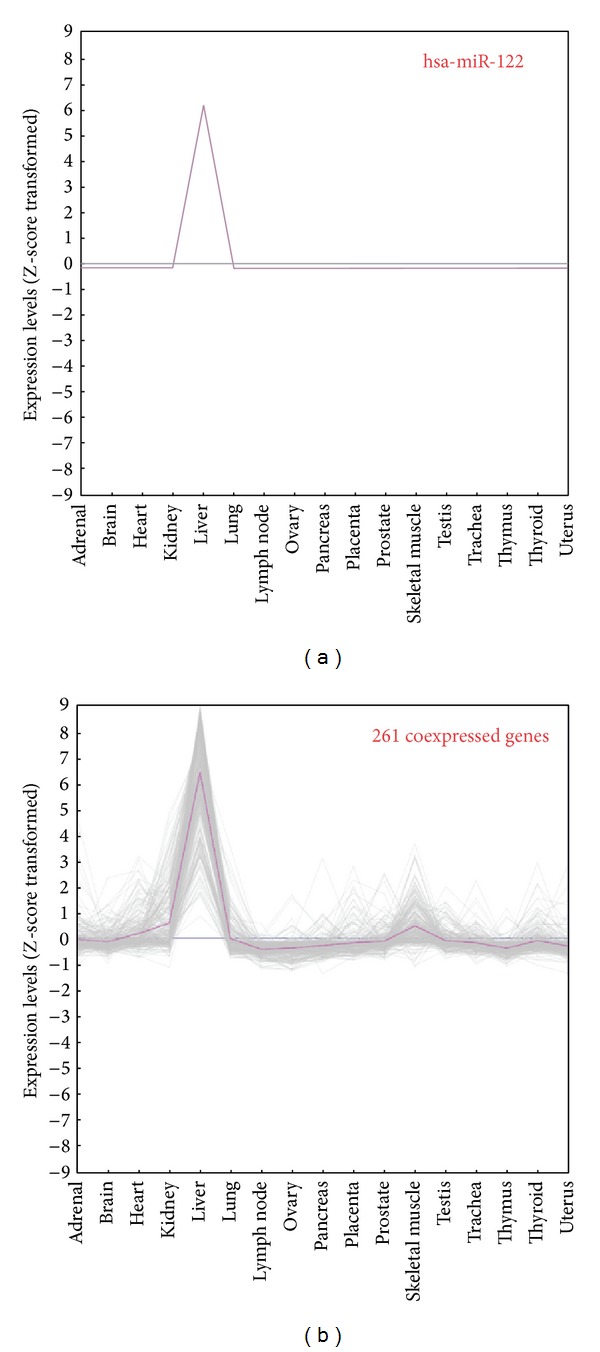
261 human protein-coding genes are coexpressed with hsa-miR-122. The pink curve in 3(b) represents the average value of miR-122 coexpressed genes among 17 human normal tissues. The remarkable peaks appear in liver.

hsa-miR-122 is one of the intergenic miRNAs whose TSS and promoter have been experimentally characterized [7]. Previous studies reported that this liver-specific miRNA is significantly downregulated in hepatocellular carcinoma and profoundly affects carcinogenesis [36]. Because of the explicit promoter and biological importance, hsa-miR-122 was selected as the case study to investigate which TFs may regulate its gene expression. 261 annotated genes coexpressed with miR-122 were identified according to their expression levels with Pearson's correlation coefficient (PCC) more than 0.8.  Figure 3 compares the expression patterns of 261 coexpressed genes (the pink line represents the average value of their expression) with hsa-miR-122 among 17 human normal tissues, indicating that remarkable peaks appear in liver for all the coexpressed genes. The expression image (see Figure S1 in additional file) also reveals the similar trends between hsa-miR-122 and its coexpressed genes.

Then, the promoter sequences (1 kb upstream from TSS) of hsa-miR-122 gene and 261 coexpressed genes were collected to identify coTFBSs using Match. The occurrence of putative transcription factors of miR-122 is listed in Table 1. Among TF candidates regulating hsa-miR-122, the TF binding motif of HNF-4alpha can be found in 191 coexpressed gene promoters. In 2011, Li et al. reported that HNF-4alpha is a key regulator positively controlling the expression of miR-122 in liver [37], proving our computational finding. They performed not only the luciferase reporter gene assay to detect the* trans*-activation effect of HNF-4alpha in miR-122 promoter but also the ChIP and EMSA assays to determine HNF-4alpha binding of miR-122 promoter in vitro and in vivo. Moreover, other liver-enriched transcription factors including HNF-1alpha, HNF-3alpha, HNF-3beta, and HNF-6 showed a strong positive correlation with miR-122. The knockout of HNF1A, FOXA1, and FOXA2 by RNAi assay reduces the expression of miR-122, suggesting that these transcription factors may bind to miR-122 promoter and transcriptionally regulate miR-122 [38]. Importantly, HNF-1alpha, HNF-3alpha, and HNF-3beta were identified in our list of TF candidates, and a HNF-6 binding site was determined (−2720 from miR-122 TSS) if the 3 kb promoter sequence of miR-122 was used.

In addition, the TF binding site of NR1B2 can be found in 226 coexpressed gene promoters. A previous research article published in 1987 indicated that the inappropriate expression of HAP gene (the official HGNC symbol is RARB) may relate to the hepatocellular carcinogenesis, and hap protein may directly participate in the hepatocellular transformation [39]. It is implied that transcription factor NR1B2 may bind to miR-122 promoter and regulate its expression.

## 4. Discussion

For large-scale investigation of human TF-miRNA relations in this study, the expression profiles of human miRNAs and over ten thousands genes from normal human tissues facilitate the acquirement of miRNA coexpressed gene groups. However, the latest usable RT-qPCR miRNA expression data for human normal tissues were published in 2007 [32]. The currently available data are almost derived from cancer cell lines or tumor tissues. Totally 230 coexpressed gene groups limit the coTFBS analysis of most miRNAs in existence. Besides, based on the cut-off 0.8 PCC value applied to define coexpression between a miRNA and an annotated gene, 34 miRNAs have no coexpressed genes and 50 miRNAs have less than ten coexpressed genes. Although each miRNA has sufficient coexpressed genes to analyze its putative* cis*- and* trans*-elements if the lower PCC cut-off was used, it may cause the trade-off of specificity when determining miRNA coexpressed genes. For example, hsa-let-7a-1 has no coexpressed gene when using 0.8 PCC value but has 29 coexpressed genes if 0.6 PCC value was applied.

It is noteworthy that the normalization process between two raw expression datasets was only *Z*-score transformed, without using log_10_ transformation before *Z*-score standardization. According to the definition of formula, Pearson's correlation coefficient calculates linear correlation between two variables and has an invariant property in statistics. In fact, the log_10_ transformation tends to alter the original expression patterns and sharply reduces the scale of raw intensity, resulting in unexpected affection of PCC values. Figure S2 illustrates an example of hsa-miR-122 expression patterns with and without log_10_ transformation. In addition to the high expression level in liver, two obvious peaks of brain and thymus appear in the log_10_ transformed expression profile of hsa-miR-122. The members of hsa-miR-122 coexpression group also reflect the variation. Unlike more than two hundred coexpression genes by using *Z*-score transformed expression data, merely five coexpressed genes left based on the cut-off 0.8 PCC value by *Z*-score and log_10_ transformed data.

Another limitation of TFBS/TF detection depends on present transcription factor binding matrices collected in TRANSFAC. As is well known, sequence motif search of transcription factor binding sites is executed by the Match program according to the matrix information in TRANSFAC library. A specific TFBS in miRNA promoter will not be detected if the corresponding matrix has not been obtained by in vitro selection studies. Here is an example. Unlike hsa-miR-122, miR-224 is upregulated in HCC through epigenetic mechanisms and controls several crucial cellular processes [40]. Wang et al. indicated that miR-224 expression is reciprocally regulated by HDAC1, HDAC3, and EP300. Our result shows that P300 (encoded by EP300) is the TF candidate of miR-224, and its TFBS can be found in 227 coexpressed gene promoters. Because matrices of histone deacetylases 1 and 3 are not available in TRANSFAC, no such transcription factors were predicted.

In conclusion, rather than traditional PWM search characterizing putative* cis*-elements in promoter regions, the coTFBS strategy was developed to determine more confident TFBSs for human miRNAs. The investigation was restricted by the incomplete expression profiles of present human miRNAs. Once most expression profiles of miRNAs in the latest database are available, TF candidates of more miRNAs can be explored in the future. Furthermore, although more and more ChIP-seq data were generated and were useful to identify transcription factor binding sites [41–43], the corresponding ChIP-seq data of specific TFs are still the minority. It is expected that large-scale ChIP analysis of general TFs contributes more confident TFBSs by observing the aggregated peaks within promoter regions of human miRNAs.

## 5. Conclusions

In organisms, not all of ribonucleic acids (RNAs) are translated to proteins. miRNAs are such noncoding RNAs which play critical roles in gene regulation, even if it is generally believed that proteins convey vital information from genes and execute biological functions to maintain life processes. Although target prediction has been the mainstream when studying miRNA functions for a while, researchers start to explore TF-miRNA interactions and study the transcriptional regulation of miRNAs, which are necessary to depict how miRNAs participate in diverse biological processes. To determine putative TFs and TFBSs located in human miRNA promoters, we created a computational pipeline which not only allows large-scale investigation as long as the expression profiles of miRNAs are available, but also filters the redundancy when searching short sequence. This valuable information is helpful in understanding miRNA functions and provides knowledge to evaluate the therapeutic potential in clinical research.

## Supplementary Material

The additional file provides the expression profile of hsa-miR-122 and its 261 co-expressed genes among 17 human normal tissues (Figure S1 and S2), and full list of TSSs, the number of co-expressed gene groups, and putative TFs of human miRNAs (Table S1-S3).

## Figures and Tables

**Table 1:** Putative transcription factors regulating miR-122 gene. The bold items indicate the experimental-supported TFs. PCC represents the Pearson's correlation coefficient between each TF-encoded gene and miR-122.

Matrix_ID	Transcription factor	Gene	PCC	Occurrence
V$ELF1_Q6	Elf-1	ELF1	0.192827	242
V$MAFB_01	MAFB	MAFB	0.383476	239
V$TBX5_02	TBX5	TBX5	0.132419	227
**V$NR1B2_Q6**	**NR1B2**	**RARB**	**0.117271**	**226**
V$CDX2_Q5_02	CDX-2	CDX2	0.105817	215
V$GATA1_01	GATA-1	GATA1	0.0128229	215
V$SMAD3_Q6_01	Smad3	SMAD3	0.0915205	203
**V$HNF4A_Q6_01**	**HNF-4alpha**	**HNF4A**	**0.316168**	**191**
V$SOX5_01	SOX5	SOX5	0.149287	185
V$SPI1_03	SPI1	SPI1	0.268782	178
V$ERBETA_Q5	ER-beta	ESR2	0.303769	176
V$GATA1_02	GATA-1	GATA1	0.0128229	173
V$GATA1_05	GATA-1	GATA1	0.0128229	168
V$GATA1_06	GATA-1	GATA1	0.0128229	168
V$CDX2_Q5_01	Cdx-2	CDX2	0.105817	167
V$MAZ_Q6	MAZ	MAZ	0.414832	166
V$MYOD_Q6_01	MyoD	MYOD1	0.306424	164
V$PITX3_Q2	PITX3	PITX3	0.127413	160
V$ING4_01	ING4	ING4	0.234372	158
**V$HNF3B_Q6**	**HNF-3beta**	**FOXA2**	**0.44036**	**154**
V$CDX2_01	Cdx-2	CDX2	0.105817	152
V$CRX_Q4	Crx	CRX	0.243011	149
**V$HNF3A_01**	**HNF3A**	**FOXA1**	**0.140429**	**148**
V$GATA1_04	GATA-1	GATA1	0.0128229	139
V$ERR1_Q3	ERR1	ESRRA	0.18197	135
V$CEBPE_Q6	CEBPE	CEBPE	0.0949125	133
**V$HNF1_02**	**HNF-1alpha**	**HNF1A**	**0.363153**	**122**
V$CRX_02	Crx	CRX	0.243011	119
V$NEUROD_02	NeuroD	NEUROD1	0.0402891	114
V$MAZ_Q6_01	MAZ	MAZ	0.414832	110
V$OC2_Q3	OC-2	ONECUT2	0.124899	102
V$IRF7_Q3	IRF-7	IRF7	0.13082	100
V$PIT1_Q6	Pit-1	POU1F1	0.319886	99
V$DBP_Q6_01	DBP	DBP	0.043001	76
V$CEBPG_Q6_01	C/EBPgamma	CEBPG	0.027117	73
V$MYOD_01	MyoD	MYOD1	0.306424	72
V$CREL_01	c-Rel	REL	0.29444	44
V$CEBPG_Q6	C/EBPgamma	CEBPG	0.027117	43
V$ATF4_Q6	ATF-4	ATF4	0.0251237	40
V$HOXA7_01	HOXA7	HOXA7	0.397453	31
V$E2F1_Q4	E2F-1	E2F1	0.110614	29
V$ATF5_01	ATF5	ATF5	0.912782	26
