# Supplementary material for: Large-Scale Investigation of Human TF-miRNA Relations Based on Coexpression Profiles
Source: Biomed Res Int. 2014 Jun 9;2014:623078. doi: 10.1155/2014/623078 (PMC4068100; doi:10.1155/2014/623078)
Supplement: Supplementary file 1 — The additional file provides the expression profile of hsa-miR-122 and its 261 co-expressed genes among 17 human normal tissues (Figure S1 and S2), and full list of TSSs, the number of co-expressed gene groups, and putative TFs of human miRNAs (Table S1-S3). [file 623078.f1.pdf]

Figure S1. The image of expression profile for hsa-miR-122 and its 261 co-expressed genes among 17 human normal tissues.

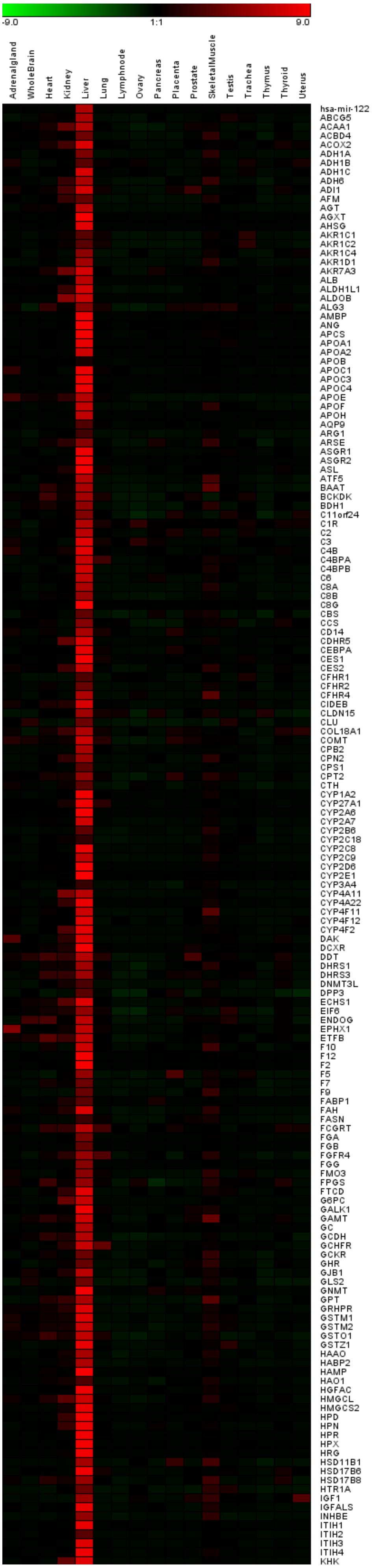

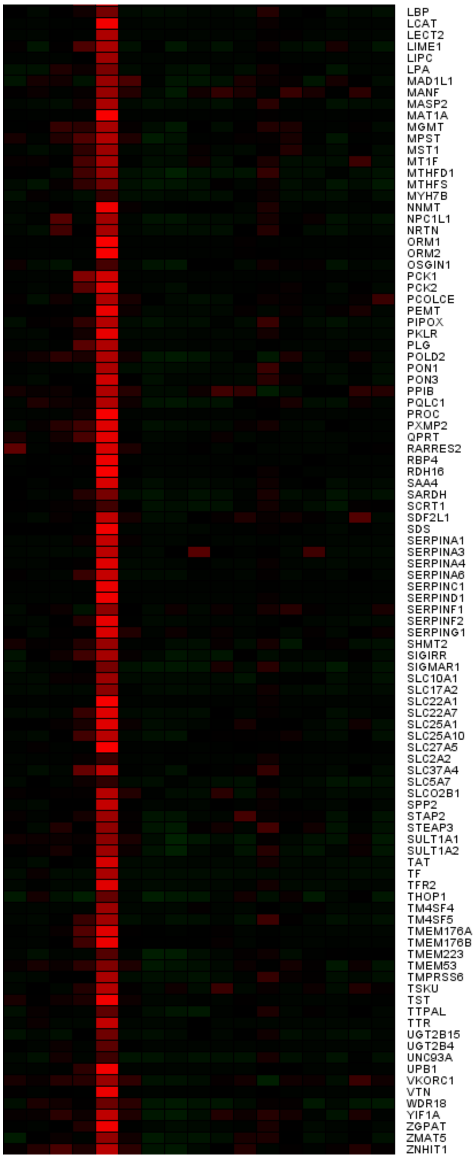
LBP
LCAT
LECT2
LIME1
LIPC
LPA
MAD1L1
MANF
MASP2
MAT1A
MGMT
MPST
MST1
MT1F
MTHFD1
MTHFS
MYH7B
NNMT
NPC1L1
NRTN
ORM1
ORM2
OSGIN1
PCK1
PCK2
PCOLCE
PEMT
PIPOX
PKLR
PLG
POLD2
PON1
PON3
PPIB
PQLC1
PROC
PXMP2
QPRT
RARRES2
RBP4
RDH16
SAA4
SARDH
SCRT1
SDF2L1
SDS
SERPINA1
SERPINA3
SERPINA4
SERPINA6
SERPINC1
SERPIND1
SERPINF1
SERPINF2
SERPING1
SHMT2
SIGIRR
SIGMAR1
SLC10A1
SLC17A2
SLC22A1
SLC22A7
SLC25A1
SLC25A10
SLC27A5
SLC2A2
SLC37A4
SLC5A7
SLCO2B1
SPP2
STAP2
STEAP3
SULT1A1
SULT1A2
TAT
TF
TFR2
THOP1
TM4SF4
TM4SF5
TMEM176A
TMEM176B
TMEM223
TMEM53
TMPRSS6
TSKU
TST
TTPAL
TTR
UGT2B15
UGT2B4
UNC93A
UPB1
VKORC1
VTN
WDR18
YIF1A
ZGPAT
ZMAT5
ZNHIT1

Figure S2. The comparison of hsa-miR-122 expression patterns with and without log_{10} transformation.

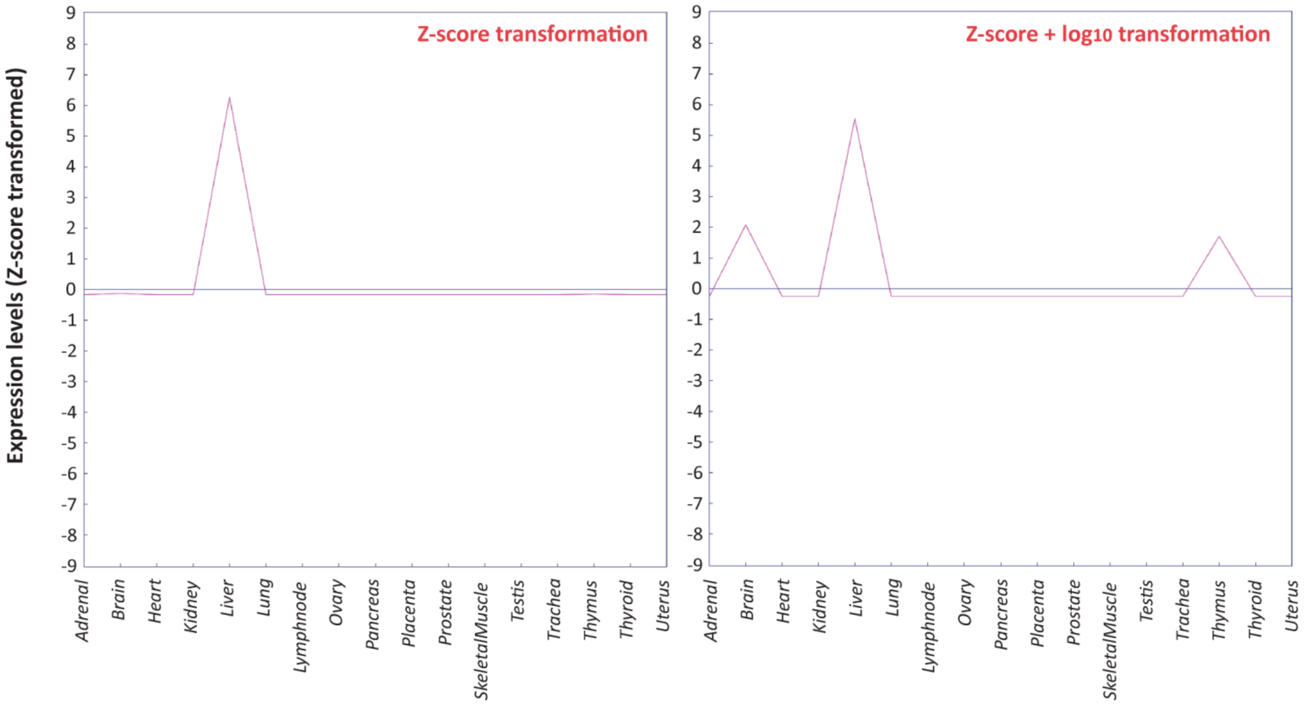

Table S1. Putative TSSs of intergenic miRNAs in human genome. (miRBase release 19)

miRNA	Genomic coordinates	Putative TSS	Distance from precursor	# of CAGE tags	# of TSS tags	H3K4me3	# of ESTs	Conservation score
hsa-let-7a-1	chr9: 96938239 [+]	96928529	9710	124	0	16	1	0.36996
hsa-let-7d	chr9: 96941116 [+]	96929155	11961	0	13	30	0	0.64614
hsa-let-7e	chr19: 52196039 [+]	52192635	3404	0	1884	9	0	0.87933
hsa-let-7f-1	chr9: 96938629 [+]	96928529	10100	124	0	16	1	0.41264
hsa-mir-19b-2	chrX: 133303796 [-]	133307920	4124	0	12	24	0	0.41535
hsa-mir-21	chr17: 57918627 [+]	57915292	3335	33	135761	8	182	0.40689
hsa-mir-30a	chr6: 72113324 [-]	72129855	16531	21	0	7	9	0.35098
hsa-mir-96	chr7: 129414609 [-]	129420061	5452	27	0	17	1	0.57303
hsa-mir-101-1	chr1: 65524191 [-]	65533415	9224	0	4659	45	5	0.93795
hsa-mir-29b-2	chr1: 207975868 [-]	207997257	21389	0	105	39	0	0.97563
hsa-mir-106a	chrX: 133304308 [-]	133307920	3612	0	12	24	0	0.39961
hsa-mir-196a-1	chr17: 46709921 [-]	46711028	1107	13	0	9	4	0.64567
hsa-mir-197	chr1: 110141515 [+]	110135089	6426	0	185	6	24	0.80787
hsa-mir-199a-1	chr19: 10928172 [-]	10946886	18714	0	2909	34	83	0.57614
hsa-mir-129-1	chr7: 127847925 [+]	127807707	40218	7	0	22	0	0.7763
hsa-mir-148a	chr7: 25989606 [-]	25990794	1188	0	99	28	0	0.92795
hsa-mir-30c-2	chr6: 72086734 [-]	72129855	43121	21	0	7	9	0.39378
hsa-mir-30d	chr8: 135817188 [-]	135844520	27332	0	193	35	0	0.75496
hsa-mir-147a	chr9: 123007328 [-]	123019092	11764	20	0	1	0	0.00986
hsa-mir-10b	chr2: 177015031 [+]	176987472	27559	0	124	33	11	0.99705
hsa-mir-34a	chr1: 9211836 [-]	9242248	30412	8	0	17	2	0.03933
hsa-mir-181c	chr19: 13985513 [+]	13976314	9199	90	0	12	1	0.74532
hsa-mir-182	chr7: 129410332 [-]	129420061	9729	27	0	17	1	0.64409
hsa-mir-183	chr7: 129414854 [-]	129420061	5207	27	0	17	1	0.65996
hsa-mir-196a-2	chr12: 54385522 [+]	54379040	6482	0	90	9	29	0.99277
hsa-mir-199b	chr9: 131007109 [-]	131038220	31111	0	2422	34	45	0.3135
hsa-mir-203a	chr14: 104583742 [+]	104583548	194	43	0	7	0	0.06831
hsa-mir-212	chr17: 1953674 [-]	1960152	6478	73	0	40	4	0.95646
hsa-mir-215	chr1: 220291304 [-]	220317326	26022	21	0	0	22	0.94614

hsa-mir-219-1	chr6: 33175612 [+]	33168676	6936	4	7928	19	323	0.59681
hsa-mir-221	chrX: 45605694 [-]	45629251	23557	15	0	25	0	0.94453
hsa-mir-222	chrX: 45606530 [-]	45629750	23220	2	0	26	0	0.91579
hsa-mir-223	chrX: 65238712 [+]	65235303	3409	11	121	12	4	0.51894
hsa-mir-200b	chr1: 1102484 [+]	1098321	4163	15	0	2	1	0.3148
hsa-let-7i	chr12: 62997466 [+]	62986639	10827	0	292	5	36	0.71429
hsa-mir-1-2	chr18: 19409049 [-]	19411367	2318	0	1207	4	9	0.97823
hsa-mir-30b	chr8: 135812850 [-]	135844582	31732	0	80	36	0	0.77768
hsa-mir-122	chr18: 56118306 [+]	56113495	4811	0	21	3	2	0.39445
hsa-mir-124-1	chr8: 9760982 [-]	9776694	15712	0	38	2	7	0.91244
hsa-mir-124-3	chr20: 61809852 [+]	61808949	903	7	0	17	0	0.96398
hsa-mir-132	chr17: 1953302 [-]	1960152	6850	73	0	40	4	0.94496
hsa-mir-133a-1	chr18: 19405746 [-]	19411367	5621	0	1207	4	9	0.97823
hsa-mir-135a-1	chr3: 52328324 [-]	52341559	13235	38	0	4	0	0.6972
hsa-mir-138-2	chr16: 56892430 [+]	56882708	9722	120	0	4	2	0.02641
hsa-mir-141	chr12: 7073260 [+]	7023747	49513	0	887	35	871	0.52831
hsa-mir-142	chr17: 56408679 [-]	56429562	20883	1	53849	32	269	0.44976
hsa-mir-144	chr17: 27188636 [-]	27224681	36045	2061	0	32	136	0.66807
hsa-mir-9-3	chr15: 89911248 [+]	89905739	5509	56	0	13	5	0.37512
hsa-mir-125a	chr19: 52196507 [+]	52192545	3962	4	0	9	0	0.94409
hsa-mir-127	chr14: 101349316 [+]	101315647	33669	1	0	3	11	0.13544
hsa-mir-129-2	chr11: 43602944 [+]	43600962	1982	15	0	28	0	0.87346
hsa-mir-134	chr14: 101521024 [+]	101491439	29585	66	0	3	0	0.58449
hsa-mir-136	chr14: 101351039 [+]	101315812	35227	1	0	20	9	0.3389
hsa-mir-146a	chr5: 159912359 [+]	159895244	17115	0	686	52	1	0.74933
hsa-mir-150	chr19: 50004125 [-]	50004150	25	221	0	76	0	0.39949
hsa-mir-154	chr14: 101526092 [+]	101491439	34653	66	0	3	0	0.56465
hsa-mir-184	chr15: 79502130 [+]	79463212	38918	69	0	0	0	8.70E-04
hsa-mir-193a	chr17: 29887015 [+]	29886135	880	240	0	12	0	0.60551
hsa-mir-194-1	chr1: 220291583 [-]	220317326	25743	21	0	0	22	0.85985
hsa-mir-206	chr6: 52009147 [+]	51993643	15504	121	0	2	0	0.00955

hsa-mir-320a	chr8: 22102556 [-]	22103064	508	5	0	41	0	0.71724
hsa-mir-200c	chr12: 7072862 [+]	7023747	49115	0	887	35	875	0.53713
hsa-mir-29c	chr1: 207975284 [-]	207997257	21973	0	105	39	0	0.97469
hsa-mir-200a	chr1: 1103243 [+]	1098321	4922	15	0	2	1	0.45598
hsa-mir-219-2	chr9: 131154993 [-]	131155780	787	11	60	14	0	0.99984
hsa-mir-299	chr14: 101490131 [+]	101463609	26522	0	69	0	0	0.00199
hsa-mir-99b	chr19: 52195865 [+]	52192545	3320	4	0	9	0	0.86591
hsa-mir-296	chr20: 57392749 [-]	57425957	33208	7	155	38	2	0.68457
hsa-mir-130b	chr22: 22007593 [+]	21996608	10985	819	6	32	105	0.48484
hsa-mir-363	chrX: 133303482 [-]	133307920	4438	0	12	24	0	0.42413
hsa-mir-365a	chr16: 14403142 [+]	14396158	6984	0	95	7	4	0.34417
hsa-mir-365b	chr17: 29902430 [+]	29860524	41906	0	208	9	4	0.02929
hsa-mir-376c	chr14: 101506027 [+]	101491439	14588	66	0	3	0	0.58445
hsa-mir-369	chr14: 101531935 [+]	101491439	40496	66	0	3	0	0.58071
hsa-mir-370	chr14: 101377476 [+]	101328211	49265	46	0	2	1	0.00407
hsa-mir-371a	chr19: 54290929 [+]	54278723	12206	0	123	1	2	0.20502
hsa-mir-372	chr19: 54291144 [+]	54278723	12421	0	123	1	4	0.20498
hsa-mir-373	chr19: 54291959 [+]	54278723	13236	0	123	1	4	0.20615
hsa-mir-375	chr2: 219866430 [-]	219906238	39808	0	75	26	15	0.46949
hsa-mir-376a-1	chr14: 101507119 [+]	101491439	15680	66	0	3	0	0.57929
hsa-mir-377	chr14: 101528387 [+]	101491439	36948	66	0	3	0	0.57276
hsa-mir-379	chr14: 101488403 [+]	101439124	49279	0	14	0	0	0.00598
hsa-mir-380	chr14: 101491354 [+]	101463609	27745	0	69	0	0	0.002
hsa-mir-381	chr14: 101512257 [+]	101491439	20818	66	0	3	0	0.5826
hsa-mir-382	chr14: 101520643 [+]	101491439	29204	66	0	3	0	0.57146
hsa-mir-328	chr16: 67236298 [-]	67281385	45087	1052	0	57	46	0.39516
hsa-mir-337	chr14: 101340830 [+]	101292454	48376	2083	0	6	358	0.87394
hsa-mir-323a	chr14: 101492069 [+]	101491439	630	66	0	3	0	0.59402
hsa-mir-135b	chr1: 205417526 [-]	205425195	7669	0	991	8	2	0.92118
hsa-mir-331	chr12: 95702196 [+]	95693246	8950	1	0	3	5	0.76736
hsa-mir-324	chr17: 7126698 [-]	7145679	18981	3	36026	32	467	0.88535

hsa-mir-133b	chr6: 52013721 [+]	51993643	20078	121	0	2	0	0.01512
hsa-mir-345	chr14: 100774196 [+]	100744115	30081	0	94	2	37	0.95843
hsa-mir-422a	chr15: 64163218 [-]	64188708	25490	8	0	2	0	0.00791
hsa-mir-424	chrX: 133680741 [-]	133680810	69	0	688	3	0	0.09323
hsa-mir-18b	chrX: 133304141 [-]	133307920	3779	0	12	24	0	0.42984
hsa-mir-20b	chrX: 133303907 [-]	133307920	4013	0	12	24	0	0.39945
hsa-mir-429	chr1: 1104385 [+]	1098321	6064	15	0	2	1	0.31024
hsa-mir-450a-1	chrX: 133674461 [-]	133680578	6117	4	0	4	12	0.47634
hsa-mir-431	chr14: 101347344 [+]	101316423	30921	1	0	1	10	0.00728
hsa-mir-433	chr14: 101348223 [+]	101315647	32576	1	0	3	11	0.17032
hsa-mir-329-1	chr14: 101493122 [+]	101491439	1683	66	0	3	0	0.58378
hsa-mir-329-2	chr14: 101493437 [+]	101491439	1998	66	0	3	0	0.57709
hsa-mir-451a	chr17: 27188458 [-]	27224681	36223	2061	0	32	143	0.68012
hsa-mir-409	chr14: 101531637 [+]	101491439	40198	66	0	3	0	0.57709
hsa-mir-412	chr14: 101531784 [+]	101491439	40345	66	0	3	0	0.58315
hsa-mir-410	chr14: 101532249 [+]	101491439	40810	66	0	3	0	0.57142
hsa-mir-376b	chr14: 101506773 [+]	101491439	15334	66	0	3	0	0.60449
hsa-mir-485	chr14: 101521756 [+]	101491439	30317	66	0	3	0	0.57886
hsa-mir-487a	chr14: 101518783 [+]	101491439	27344	66	0	3	0	0.58807
hsa-mir-146b	chr10: 104196269 [+]	104155462	40807	2	180	10	55	0.89929
hsa-mir-202	chr10: 135061124 [-]	135090371	29247	8	5	40	80	0.04334
hsa-mir-493	chr14: 101335397 [+]	101292454	42943	2083	0	6	357	0.94638
hsa-mir-432	chr14: 101350820 [+]	101315647	35173	1	0	3	11	0.15536
hsa-mir-494	chr14: 101495971 [+]	101491439	4532	66	0	3	0	0.59909
hsa-mir-495	chr14: 101500092 [+]	101491439	8653	66	0	3	0	0.56465
hsa-mir-496	chr14: 101526910 [+]	101491439	35471	66	0	3	0	0.56465
hsa-mir-193b	chr16: 14397824 [+]	14396158	1666	0	95	7	4	0.32866
hsa-mir-181d	chr19: 13985689 [+]	13976314	9375	90	0	12	1	0.74661
hsa-mir-512-1	chr19: 54169933 [+]	54120041	49892	79	0	0	0	0.18172
hsa-mir-512-2	chr19: 54172411 [+]	54172186	225	24	0	3	0	0.00807
hsa-mir-498	chr19: 54177451 [+]	54172186	5265	24	0	3	0	0.01278

hsa-mir-520e	chr19: 54178965 [+]	54172186	6779	24	0	3	0	0.01316
hsa-mir-515-1	chr19: 54182257 [+]	54172186	10071	24	0	3	0	0.01294
hsa-mir-519e	chr19: 54183194 [+]	54172186	11008	24	0	3	0	0.00885
hsa-mir-520f	chr19: 54185413 [+]	54172186	13227	24	0	3	0	0.00779
hsa-mir-515-2	chr19: 54188263 [+]	54172186	16077	24	0	3	0	0.01319
hsa-mir-519c	chr19: 54189723 [+]	54172186	17537	24	0	3	0	0.01812
hsa-mir-520a	chr19: 54194135 [+]	54172186	21949	24	0	3	0	0.01452
hsa-mir-526b	chr19: 54197647 [+]	54172186	25461	24	0	3	0	0.01285
hsa-mir-519b	chr19: 54198467 [+]	54172186	26281	24	0	3	0	0.01303
hsa-mir-525	chr19: 54200787 [+]	54172186	28601	24	0	3	0	0.01012
hsa-mir-523	chr19: 54201639 [+]	54172186	29453	24	0	3	0	0.0137
hsa-mir-518f	chr19: 54203269 [+]	54172186	31083	24	0	3	0	0.01287
hsa-mir-520b	chr19: 54204481 [+]	54172186	32295	24	0	3	0	0.01148
hsa-mir-518b	chr19: 54205991 [+]	54172186	33805	24	0	3	0	0.00929
hsa-mir-526a-1	chr19: 54209506 [+]	54172186	37320	24	0	3	0	0.00843
hsa-mir-520c	chr19: 54210707 [+]	54172186	38521	24	0	3	0	0.00838
hsa-mir-518c	chr19: 54211989 [+]	54172186	39803	24	0	3	0	0.00968
hsa-mir-524	chr19: 54214256 [+]	54172186	42070	24	0	3	0	0.0129
hsa-mir-517a	chr19: 54215522 [+]	54172186	43336	24	0	3	0	0.01854
hsa-mir-519d	chr19: 54216601 [+]	54172186	44415	24	0	3	0	0.00855
hsa-mir-521-2	chr19: 54219848 [+]	54172186	47662	24	0	3	0	0.01282
hsa-mir-450a-2	chrX: 133674637 [-]	133680578	5941	4	0	4	12	0.37752
hsa-mir-505	chrX: 139006390 [-]	139015194	8804	0	14656	40	7	0.43185
hsa-mir-513a-1	chrX: 146295109 [-]	146314685	19576	89	0	1	0	0.05121
hsa-mir-513a-2	chrX: 146307470 [-]	146314685	7215	89	0	1	0	0.05791
hsa-mir-506	chrX: 146312361 [-]	146314685	2324	89	0	1	0	0.0572
hsa-mir-507	chrX: 146312595 [-]	146314685	2090	89	0	1	0	0.04473
hsa-mir-508	chrX: 146318545 [-]	146351035	32490	20	0	2	0	0.2214
hsa-mir-509-1	chrX: 146342143 [-]	146390339	48196	100	0	2	0	0.20831
hsa-mir-510	chrX: 146353926 [-]	146390339	36413	100	0	2	0	0.20376
hsa-mir-514a-1	chrX: 146360862 [-]	146390339	29477	100	0	2	0	0.19487

hsa-mir-514a-2	chrX: 146363548 [-]	146390339	26791	100	0	2	0	0.20551
hsa-mir-514a-3	chrX: 146366246 [-]	146390339	24093	100	0	2	0	0.20657
hsa-mir-539	chr14: 101513658 [+]	101491439	22219	66	0	3	0	0.58299
hsa-mir-544a	chr14: 101514995 [+]	101491439	23556	66	0	3	0	0.56465
hsa-mir-376a-2	chr14: 101506406 [+]	101491439	14967	66	0	3	0	0.56465
hsa-mir-487b	chr14: 101512792 [+]	101491439	21353	66	0	3	0	0.56465
hsa-mir-552	chr1: 35135295 [-]	35150930	15635	8	0	4	0	0.00126
hsa-mir-92b	chr1: 155164968 [+]	155146376	18592	0	493	21	151	0.90268
hsa-mir-557	chr1: 168344762 [+]	168338249	6513	32	0	2	0	0.18015
hsa-mir-563	chr3: 15915278 [+]	15901669	13609	0	211	33	6	0.1111
hsa-mir-564	chr3: 44903380 [+]	44901601	1779	246	0	4	0	0.10757
hsa-mir-568	chr3: 114035416 [-]	114052542	17126	0	96	5	8	0.72563
hsa-mir-570	chr3: 195426272 [+]	195384935	41337	4400	0	4	86	0.06903
hsa-mir-572	chr4: 11370451 [+]	11370266	185	3	0	1	0	0.26876
hsa-mir-573	chr4: 24521913 [-]	24531367	9454	0	26	15	36	0.90283
hsa-mir-583	chr5: 95414842 [+]	95374210	40632	319	0	0	0	0.25126
hsa-mir-587	chr6: 107232000 [+]	107194425	37575	0	320	3	0	0.46213
hsa-mir-548a-2	chr6: 135560298 [+]	135516916	43382	27	0	2	3	0.94161
hsa-mir-596	chr8: 1765397 [+]	1745447	19950	2	0	3	0	0.15124
hsa-mir-548a-3	chr8: 105496693 [-]	105501878	5185	0	50	0	8	0.85945
hsa-mir-602	chr9: 140732871 [+]	140730243	2628	0	82	4	6	0.7598
hsa-mir-606	chr10: 77312216 [+]	77307735	4481	13	0	3	0	0.93811
hsa-mir-607	chr10: 98588521 [-]	98592185	3664	246	0	23	2	0.58161
hsa-mir-610	chr11: 28078362 [+]	28041088	37274	0	187	2	0	0.06189
hsa-mir-614	chr12: 13068763 [+]	13044499	24264	0	6977	38	256	0.29492
hsa-mir-622	chr13: 90883436 [+]	90874748	8688	35	0	1	0	0.05756
hsa-mir-630	chr15: 72879558 [+]	72873125	6433	0	25	18	16	0.98602
hsa-mir-631	chr15: 75646026 [-]	75660946	14920	17	1230	41	285	0.53657
hsa-mir-632	chr17: 30677128 [+]	30676882	246	1	0	42	4	0.0019
hsa-mir-633	chr17: 61021576 [+]	60998963	22613	31	0	2	0	0.24551
hsa-mir-639	chr19: 14640355 [+]	14627409	12946	0	5	40	17	0.93898

hsa-mir-645	chr20: 49202323 [+]	49201170	1153	0	191	6	4	0.5737
hsa-mir-649	chr22: 21388561 [-]	21398551	9990	0	411	4	1	0.56878
hsa-mir-662	chr16: 820183 [+]	771146	49037	0	124	27	33	0.07854
hsa-mir-548d-2	chr17: 65467701 [-]	65474627	6926	0	39	14	0	0.15827
hsa-mir-411	chr14: 101489662 [+]	101457982	31680	0	31	0	0	1.20E-04
hsa-mir-654	chr14: 101506556 [+]	101491439	15117	66	0	3	0	0.57886
hsa-mir-655	chr14: 101515887 [+]	101491439	24448	66	0	3	0	0.57276
hsa-mir-656	chr14: 101533061 [+]	101491439	41622	66	0	3	0	0.58508
hsa-mir-549a	chr15: 81134414 [-]	81160813	26399	23	0	3	0	0.00141
hsa-mir-658	chr22: 38240378 [-]	38273766	33388	0	225	6	80	0.9585
hsa-mir-659	chr22: 38243781 [-]	38273766	29985	0	225	6	80	0.95866
hsa-mir-542	chrX: 133675467 [-]	133680578	5111	4	0	4	12	0.47634
hsa-mir-758	chr14: 101492357 [+]	101491439	918	66	0	3	0	0.5826
hsa-mir-668	chr14: 101521595 [+]	101491439	30156	66	0	3	0	0.56465
hsa-mir-320b-1	chr1: 117214371 [+]	117208978	5393	38	0	0	0	0.28244
hsa-mir-320c-1	chr18: 19263471 [+]	19250302	13169	0	76	2	1	0.0685
hsa-mir-1323	chr19: 54175222 [+]	54172186	3036	24	0	3	0	0.01854
hsa-mir-1185-2	chr14: 101510535 [+]	101491439	19096	66	0	3	0	0.58071
hsa-mir-1283-1	chr19: 54191735 [+]	54172186	19549	24	0	3	0	0.01452
hsa-mir-769	chr19: 46522190 [+]	46498725	23465	13	6028	30	17	0.44715
hsa-mir-1185-1	chr14: 101509314 [+]	101491439	17875	66	0	3	0	0.56457
hsa-mir-802	chr21: 37093013 [+]	37077286	15727	27	0	2	0	0.08574
hsa-mir-670	chr11: 43581206 [+]	43569577	11629	14	0	3	0	0.40016
hsa-mir-759	chr13: 53384185 [+]	53365045	19140	59	0	2	0	0.02012
hsa-mir-298	chr20: 57393368 [-]	57425957	32589	7	155	38	2	0.70996
hsa-mir-300	chr14: 101507700 [+]	101491439	16261	66	0	3	0	0.56465
hsa-mir-892a	chrX: 145078261 [-]	145078831	570	37	0	2	0	4.10E-04
hsa-mir-509-2	chrX: 146340368 [-]	146390339	49971	100	0	2	0	0.18851
hsa-mir-450b	chrX: 133674292 [-]	133680673	6381	3	0	2	12	0.45
hsa-mir-890	chrX: 145075869 [-]	145078831	2962	37	0	2	0	4.10E-04
hsa-mir-888	chrX: 145076378 [-]	145078831	2453	37	0	2	0	4.20E-04

hsa-mir-892b	chrX: 145078792 [-]	145078831	39	37	0	2	0	3.30E-04
hsa-mir-541	chr14: 101530832 [+]	101491439	39393	66	0	3	0	0.58417
hsa-mir-889	chr14: 101514238 [+]	101491439	22799	66	0	3	0	0.57551
hsa-mir-190b	chr1: 154166219 [-]	154193113	26894	0	32881	46	486	0.25752
hsa-mir-665	chr14: 101341370 [+]	101292454	48916	2083	0	6	358	0.80091
hsa-mir-543	chr14: 101498324 [+]	101491439	6885	66	0	3	0	0.58449
hsa-mir-760	chr1: 94312388 [+]	94292348	20040	52	0	3	0	0.04175
hsa-mir-301b	chr22: 22007270 [+]	21996608	10662	819	6	32	108	0.41417
hsa-mir-920	chr12: 24365355 [+]	24338975	26380	50	0	1	0	0.00268
hsa-mir-509-3	chrX: 146341244 [-]	146390339	49095	100	0	2	0	0.20772
hsa-mir-940	chr16: 2321748 [+]	2273659	48089	1	150	39	70	0.94559
hsa-mir-297	chr4: 111781803 [-]	111797978	16175	139	0	2	0	0.15996
hsa-mir-1208	chr8: 129162362 [+]	129121485	40877	0	5	2	0	0.30114
hsa-mir-1285-2	chr2: 70480137 [-]	70520812	40675	0	49980	54	322	0.32563
hsa-mir-1289-1	chr20: 34041919 [-]	34090627	48708	0	338	13	2	0.86965
hsa-mir-1299	chr9: 69002321 [-]	69032078	29757	5	0	1	0	0.00701
hsa-mir-1302-1	chr12: 113132981 [-]	113136628	3647	41	0	3	0	0.03973
hsa-mir-1302-2	chr1: 30366 [+]	10842	19524	13	0	0	0	0.03614
hsa-mir-1302-3	chr2: 114340673 [-]	114384658	43985	0	2117	4	44	0.10555
hsa-mir-1302-6	chr7: 18166932 [-]	18173133	6201	41	0	0	0	0.68894
hsa-mir-1302-8	chr9: 100125963 [-]	100174452	48489	31	0	29	3	0.62063
hsa-mir-1303	chr5: 154065336 [+]	154062551	2785	0	241	19	5	0.2113
hsa-mir-1244-1	chr12: 9392147 [-]	9392585	438	0	25	7	7	0.83721
hsa-mir-1246	chr2: 177465780 [-]	177490993	25213	96	0	1	0	0.09412
hsa-mir-1247	chr14: 102026759 [-]	102074469	47710	1056	0	2	0	0.05578
hsa-mir-1253	chr17: 2651476 [-]	2699517	48041	0	77	73	0	0.62787
hsa-mir-1257	chr20: 60528718 [-]	60550456	21738	0	16	9	8	0.71598
hsa-mir-548g	chr4: 148265869 [-]	148280562	14693	69	0	0	0	0.02206
hsa-mir-1263	chr3: 163889344 [-]	163938392	49048	24	0	1	0	0.00234
hsa-mir-548m	chrX: 94318225 [-]	94342154	23929	47	0	0	0	0.36418
hsa-mir-1265	chr10: 14478575 [+]	14447281	31294	0	56	4	0	0.00259

hsa-mir-548o	chr7: 102046302 [-]	102073988	27686	11	0	9	1	0.54177
hsa-mir-548h-1	chr14: 64561843 [-]	64586290	24447	0	207	5	0	0.83575
hsa-mir-548h-2	chr16: 11400384 [-]	11439139	38755	0	125	25	2	0.06724
hsa-mir-1275	chr6: 33967828 [-]	34017440	49612	28	0	3	0	0.01024
hsa-mir-548i-2	chr4: 9557937 [-]	9606531	48594	0	31	30	0	0.07432
hsa-mir-548i-4	chrX: 83480836 [-]	83517181	36345	23	0	1	0	0.04104
hsa-mir-1279	chr12: 69666998 [-]	69678016	11018	21	0	15	0	0.07324
hsa-mir-1281	chr22: 41488517 [+]	41460694	27823	0	15	0	23	0.23319
hsa-mir-513b	chrX: 146280645 [-]	146314685	34040	89	0	1	0	0.05078
hsa-mir-513c	chrX: 146271305 [-]	146314685	43380	89	0	1	0	0.04919
hsa-mir-1197	chr14: 101491901 [+]	101491439	462	66	0	3	0	0.56465
hsa-mir-1324	chr3: 75679914 [+]	75639730	40184	198	0	1	0	0.04246
hsa-mir-1470	chr19: 15560359 [+]	15535724	24635	0	172	11	40	0.90571
hsa-mir-1471	chr2: 232757008 [-]	232791107	34099	20	0	29	2	0.62032
hsa-mir-1538	chr16: 69599771 [-]	69600164	393	1935	0	61	0	0.99685
hsa-mir-1539	chr18: 47013743 [+]	47013372	371	12	0	85	1	4.00E-05
hsa-mir-103b-1	chr5: 167987909 [+]	167957147	30762	0	16	4	3	0.90697
hsa-mir-103b-2	chr20: 3898210 [-]	3942186	43976	2	0	2	0	0.2296
hsa-mir-320d-1	chr13: 41302011 [-]	41345305	43294	4	1938	53	134	0.02999
hsa-mir-320c-2	chr18: 21901650 [+]	21851937	49713	23	0	7	0	0.18589
hsa-mir-320d-2	chrX: 140008384 [-]	140050697	42313	23	0	0	0	0.11747
hsa-mir-1827	chr12: 100583662 [+]	100535657	48005	0	79	25	0	0.53705
hsa-mir-1972-1	chr16: 15104254 [-]	15149887	45633	6	24	13	56	0.26949
hsa-mir-1973	chr4: 117220881 [+]	117219397	1484	1293	0	0	7	0.37661
hsa-mir-2114	chrX: 149396239 [+]	149390740	5499	53	0	0	0	0.0029
hsa-mir-2117	chr17: 41522174 [+]	41476361	45813	13	309	48	133	0.14669
hsa-mir-548q	chr10: 12767352 [-]	12811010	43658	40	0	7	0	3.10E-04
hsa-mir-718	chrX: 153285440 [-]	153296454	11014	127	0	5	7	0.79807
hsa-mir-2861	chr9: 130548197 [+]	130538868	9329	0	400	54	7	0.23807
hsa-mir-3116-2	chr1: 62544528 [-]	62594508	49980	0	186	6	0	0.48291
hsa-mir-3118-3	chr1: 143424215 [-]	143467631	43416	77	0	13	6	0.00589

hsa-mir-3119-1	chr1: 170120603 [-]	170133088	12485	49	0	4	0	0.02441
hsa-mir-3123	chr1: 241295572 [+]	241277618	17954	62	0	0	0	0.00134
hsa-mir-3124	chr1: 249120576 [+]	249107201	13375	0	6824	6	2	0.8998
hsa-mir-3132	chr2: 220413869 [-]	220436176	22307	668	0	8	23	0.69161
hsa-mir-378b	chr3: 10371913 [+]	10342978	28935	0	212	13	5	0.50906
hsa-mir-3141	chr5: 153975632 [-]	153990220	14588	495	0	11	0	0.10177
hsa-mir-3142	chr5: 159901409 [+]	159895244	6165	0	686	52	2	0.61665
hsa-mir-3143	chr6: 27115405 [+]	27106111	9294	0	3	2	45	0.00197
hsa-mir-3144	chr6: 120336325 [+]	120326870	9455	0	69	1	1	0.0732
hsa-mir-3147	chr7: 57472731 [+]	57436499	36232	102	0	1	0	0.30364
hsa-mir-3149	chr8: 77879086 [-]	77912462	33376	7	0	84	32	0.20508
hsa-mir-3074	chr9: 97848376 [-]	97851479	3103	0	81	3	0	5.60E-04
hsa-mir-3154	chr9: 131007309 [-]	131038220	30911	0	2422	34	45	0.3135
hsa-mir-3158-2	chr10: 103361254 [-]	103368681	7427	0	98	13	0	0.59882
hsa-mir-3159	chr11: 18409334 [+]	18387720	21614	0	36	5	7	0.78657
hsa-mir-3166	chr11: 87909670 [+]	87908653	1017	8	0	17	0	0.48618
hsa-mir-3168	chr13: 41675236 [-]	41706935	31699	4	3953	63	167	0.11354
hsa-mir-3169	chr13: 61774014 [-]	61781193	7179	37	0	0	0	0
hsa-mir-1193	chr14: 101496389 [+]	101491439	4950	66	0	3	0	0.56953
hsa-mir-323b	chr14: 101522556 [+]	101491439	31117	66	0	3	0	0.57886
hsa-mir-3118-4	chr15: 21038124 [+]	21016116	22008	88	0	1	0	0.17932
hsa-mir-3178	chr16: 2582006 [-]	2587967	5961	15	0	16	0	0.55815
hsa-mir-3179-1	chr16: 14995365 [+]	14946913	48452	19	0	0	1	0.0161
hsa-mir-3180-1	chr16: 15005077 [+]	14989917	15160	0	14	3	11	0.16272
hsa-mir-3180-2	chr16: 16403736 [+]	16403136	600	1	0	0	0	0.09771
hsa-mir-3179-2	chr16: 16394016 [+]	16345569	48447	22	0	1	1	0.0013
hsa-mir-3180-3	chr16: 18496128 [-]	18496279	151	36	0	0	0	0
hsa-mir-3179-3	chr16: 18505834 [-]	18554304	48470	22	0	0	1	0.00528
hsa-mir-3184	chr17: 28444178 [-]	28448153	3975	0	176	4	0	0.08126
hsa-mir-3185	chr17: 46801837 [-]	46806074	4237	2	0	13	50	0.88248
hsa-mir-3065	chr17: 79099677 [+]	79071417	28260	91	0	6	18	0.14236

hsa-mir-3186	chr17: 79418214 [-]	79431623	13409	0	340	0	1	0.22028
hsa-mir-3187	chr19: 813584 [+]	797452	16132	162	140960	12	403	0.64992
hsa-mir-3188	chr19: 18392887 [+]	18390574	2313	0	918	29	222	0.43512
hsa-mir-3190	chr19: 47730199 [+]	47724204	5995	80	0	10	11	0.95717
hsa-mir-3193	chr20: 30194989 [+]	30193092	1897	20	20284	53	487	0.48032
hsa-mir-3195	chr20: 60639858 [+]	60639501	357	0	20	31	0	0.78555
hsa-mir-3118-5	chr21: 15017171 [-]	15037875	20704	88	0	0	0	0.03695
hsa-mir-3197	chr21: 42539484 [+]	42536347	3137	61	0	4	0	0.0291
hsa-mir-3199-1	chr22: 28316600 [-]	28329277	12677	0	90	1	0	0.08204
hsa-mir-3201	chr22: 48670176 [+]	48649582	20594	31	0	0	0	0.04813
hsa-mir-514b	chrX: 146331748 [-]	146351035	19287	20	0	2	0	0.22206
hsa-mir-3202-2	chrX: 153246627 [-]	153285349	38722	6554	0	8	48	0.64559
hsa-mir-4294	chr10: 50193632 [-]	50194526	894	35	0	2	0	0.0021
hsa-mir-4298	chr11: 1880766 [-]	1913366	32600	61	0	27	17	8.40E-04
hsa-mir-4305	chr13: 40238272 [-]	40244256	5984	88	0	2	0	0.02626
hsa-mir-4309	chr14: 103005981 [+]	102976060	29921	1226	0	7	0	0.81894
hsa-mir-4307	chr14: 27377848 [+]	27336297	41551	33	0	0	0	0.17681
hsa-mir-4311	chr15: 66332571 [+]	66317283	15288	30	0	1	0	0.53685
hsa-mir-4316	chr17: 75393136 [-]	75398252	5116	0	95	6	0	0.86398
hsa-mir-4314	chr17: 7991374 [+]	7982776	8598	18	6	36	6	0.64441
hsa-mir-4319	chr18: 42550131 [-]	42558168	8037	0	62	3	0	0.38783
hsa-mir-4322	chr19: 10341089 [+]	10317122	23967	41	0	11	0	0.06231
hsa-mir-4323	chr19: 42637665 [-]	42681426	43761	10	8	25	0	0.99988
hsa-mir-4256	chr1: 113004455 [-]	113021936	17481	49	0	0	0	0.06831
hsa-mir-4253	chr1: 23189719 [-]	23233240	43521	0	190	5	2	0.95614
hsa-mir-4252	chr1: 6489956 [-]	6520505	30549	0	12	14	24	0.31953
hsa-mir-4325	chr20: 55896647 [-]	55934250	37603	51	0	4	5	0.08746
hsa-mir-4261	chr2: 10332797 [-]	10379625	46828	66	0	2	0	0.04506
hsa-mir-4266	chr2: 109930081 [-]	109935653	5572	0	347	4	0	0.0013
hsa-mir-4267	chr2: 110827619 [-]	110873396	45777	23	542	6	84	0.38185
hsa-mir-4262	chr2: 11977112 [-]	12015280	38168	3	0	4	0	0.00385

hsa-mir-4269	chr2: 240227157 [+]	240182677	44480	38	0	30	0	6.70E-04
hsa-mir-4275	chr4: 28821204 [+]	28801527	19677	51	0	0	0	0.05636
hsa-mir-4277	chr5: 1708983 [-]	1733643	24660	72	0	3	0	0.00288
hsa-mir-4279	chr5: 31936265 [-]	31962581	26316	0	48	1	0	0.02484
hsa-mir-4278	chr5: 6828034 [-]	6848575	20541	512	0	4	0	0.05283
hsa-mir-4280	chr5: 86410771 [-]	86418012	7241	0	444	0	0	0.00459
hsa-mir-4282	chr6: 73677476 [-]	73704410	26934	55	0	1	0	0.00245
hsa-mir-4283-1	chr7: 57023571 [-]	57071886	48315	11	0	0	0	0.65606
hsa-mir-4284	chr7: 73125647 [+]	73097927	27720	4	27000	54	320	0.14453
hsa-mir-4291	chr9: 96581639 [+]	96536745	44894	53	0	1	0	0.23038
hsa-mir-1184-2	chrX: 154612847 [-]	154613085	238	5	0	0	3	8.70E-04
hsa-mir-1184-3	chrX: 154687178 [+]	154686940	238	5	0	0	4	0.00169
hsa-mir-1302-9	chr15: 102500799 [-]	102520430	19631	13	0	1	0	0.14644
hsa-mir-1302-11	chr9: 30144 [+]	11165	18979	0	23	4	2	0.07767
hsa-mir-3118-6	chr15: 22049274 [+]	22027266	22008	88	0	1	0	0.08627
hsa-mir-4283-2	chr7: 63081468 [+]	63033294	48174	67	0	0	0	0.7511
hsa-mir-3615	chr17: 72744752 [+]	72733394	11358	31	0	35	42	0
hsa-mir-3617	chr20: 44333819 [-]	44374632	40813	16	0	0	0	0
hsa-mir-3622a	chr8: 27559194 [+]	27543344	15850	0	179	1	0	0
hsa-mir-3622b	chr8: 27559284 [-]	27593114	33830	0	7	10	19	0
hsa-mir-3649	chr12: 1769546 [-]	1800041	30495	0	17	34	0	0
hsa-mir-3659	chr1: 38554903 [+]	38512306	42597	21	0	12	0	0
hsa-mir-3660	chr5: 89312537 [-]	89317982	5445	0	4	1	1	0
hsa-mir-3661	chr5: 133561448 [+]	133541775	19673	0	162	11	2	0
hsa-mir-3663	chr10: 118927285 [-]	118934696	7411	5	0	59	0	0
hsa-mir-3665	chr13: 78272251 [-]	78272264	13	33	0	27	0	0
hsa-mir-3670-1	chr16: 15001574 [+]	14989917	11657	0	14	3	11	0.17665
hsa-mir-3671	chr1: 65523525 [-]	65533415	9890	0	4659	45	5	0.9389
hsa-mir-3672	chrX: 120504826 [+]	120481451	23375	10	0	1	0	0
hsa-mir-3674	chr8: 1749291 [+]	1711955	37336	11	1434	35	77	0
hsa-mir-3675	chr1: 17185516 [-]	17216129	30613	0	1001	52	11	0

hsa-mir-3676	chr17: 8090493 [+]	8063611	26882	0	114	9	93	0
hsa-mir-3677	chr16: 2320714 [+]	2273659	47055	1	150	39	61	0.80823
hsa-mir-3678	chr17: 73402150 [+]	73390227	11923	0	137	56	1	0
hsa-mir-3679	chr2: 134884696 [+]	134877520	7176	8	535	49	5	0
hsa-mir-3683	chr7: 7106676 [-]	7144112	37436	0	63	1	0	0
hsa-mir-3685	chr12: 95703699 [+]	95693246	10453	1	0	3	5	0.75303
hsa-mir-3687	chr21: 9826203 [+]	9826225	22	0	13	0	0	0
hsa-mir-3688-1	chr4: 160050046 [-]	160053052	3006	0	122	1	1	0
hsa-mir-3180-4	chr16: 15248859 [-]	15249481	622	1	0	0	0	0
hsa-mir-3180-5	chr16: 2186130 [-]	2205359	19229	0	7860	47	32	0
hsa-mir-3908	chr12: 124020956 [+]	124018175	2781	84	0	15	0	0
hsa-mir-3910-1	chr9: 94398533 [+]	94390223	8310	90	0	0	0	0
hsa-mir-3911	chr9: 130453074 [-]	130497605	44531	644	0	26	86	0
hsa-mir-3912	chr5: 170813764 [-]	170832347	18583	0	160	7	10	0
hsa-mir-3913-1	chr12: 69978603 [-]	70004684	26081	1	300	3	1	0
hsa-mir-3913-2	chr12: 69978503 [+]	69973312	5191	0	29	2	3	0
hsa-mir-3916	chr1: 247365362 [-]	247374150	8788	10	417	54	6	0
hsa-mir-3918	chr6: 159185785 [-]	159187608	1823	0	383	11	68	0
hsa-mir-3150b	chr8: 96085224 [-]	96099988	14764	91	0	3	0	0
hsa-mir-3925	chr6: 36590289 [-]	36607449	17160	806	0	2	0	0
hsa-mir-3927	chr9: 112273825 [-]	112296752	22927	655562	0	0	15	0
hsa-mir-3928	chr22: 31556105 [-]	31599689	43584	55	0	4	0	0
hsa-mir-3929	chr18: 33514105 [-]	33552426	38321	0	2	38	3	0
hsa-mir-3934	chr6: 33665905 [+]	33655444	10461	0	3	10	0	0
hsa-mir-3937	chrX: 39520470 [+]	39497866	22604	48	0	1	0	0
hsa-mir-548y	chr14: 48230307 [-]	48264208	33901	43	0	1	0	0
hsa-mir-3939	chr6: 167411400 [-]	167412570	1170	0	270	44	5	0
hsa-mir-374c	chrX: 73438384 [+]	73393889	44495	0	537	2	1	0
hsa-mir-642b	chr19: 46178266 [-]	46195192	16926	1	721074	65	380	0
hsa-mir-550b-1	chr7: 30329506 [-]	30378058	48552	25	0	0	0	0
hsa-mir-550b-2	chr7: 32772689 [-]	32801498	28809	17	0	3	1	0

hsa-mir-548z	chr12: 65016385 [-]	65058436	42051	34	0	3	0	0
hsa-mir-151b	chr14: 100575851 [-]	100594946	19095	17	0	31	3	0
hsa-mir-378d-2	chr8: 94928347 [-]	94969755	41408	97	0	0	5	0
hsa-mir-1254-2	chr10: 23682334 [+]	23632980	49354	0	8	7	4	0
hsa-mir-548h-5	chr6: 132113312 [+]	132067158	46154	13	0	1	0	0
hsa-mir-4417	chr1: 5624131 [+]	5623101	1030	27	0	4	0	0
hsa-mir-4418	chr1: 22592732 [+]	22565992	26740	39	0	3	0	0
hsa-mir-4419a	chr1: 23384427 [-]	23413775	29348	0	9	5	13	0
hsa-mir-378f	chr1: 24255560 [+]	24229281	26279	51	0	1	2	0
hsa-mir-4421	chr1: 51525509 [+]	51519207	6302	81	0	2	0	0
hsa-mir-4422	chr1: 55691314 [+]	55681096	10218	0	7	46	1	0
hsa-mir-4423	chr1: 85599477 [+]	85556134	43343	71	0	1	0	0
hsa-mir-4424	chr1: 178646884 [+]	178613674	33210	0	60	1	0	0
hsa-mir-4426	chr1: 192685458 [+]	192685274	184	1638	8	1	4	0
hsa-mir-4429	chr2: 11680803 [-]	11699965	19162	28	0	4	0	0
hsa-mir-4430	chr2: 33643583 [+]	33623847	19736	0	453	3	10	0
hsa-mir-4433	chr2: 64567893 [+]	64532278	35615	25	0	0	0	0
hsa-mir-4436a	chr2: 89111884 [+]	89065390	46494	0	1134	22	21	0
hsa-mir-548ae-2	chr5: 57825936 [-]	57861021	35085	0	55	5	0	0
hsa-mir-4443	chr3: 48238054 [+]	48222991	15063	102	18	0	2	0
hsa-mir-4445	chr3: 109321675 [+]	109281078	40597	48	0	0	0	0
hsa-mir-4448	chr3: 183604588 [+]	183602519	2069	7	0	97	0	0
hsa-mir-4456	chr5: 535997 [-]	537831	1834	49	0	4	0	0
hsa-mir-4457	chr5: 1309492 [-]	1344980	35488	17	0	32	49	0
hsa-mir-4460	chr5: 128732840 [-]	128775816	42976	1	0	3	0	0
hsa-mir-378h	chr5: 154209018 [+]	154194477	14541	0	415	7	46	0
hsa-mir-4462	chr6: 37523198 [-]	37545439	22241	0	1438	0	0	0
hsa-mir-4463	chr6: 76138123 [+]	76106034	32089	0	575	4	0	0
hsa-mir-4464	chr6: 91022461 [+]	91006524	15937	17	0	31	0	0
hsa-mir-548ai	chr6: 99572485 [+]	99569510	2975	38	0	1	0	0
hsa-mir-4466	chr6: 157100865 [-]	157101589	724	0	351	28	1	0

hsa-mir-4468	chr7: 137808504 [+]	137805833	2671	7	60	3	0	0
hsa-mir-4470	chr8: 62627347 [+]	62621485	5862	0	179	1	0	0
hsa-mir-4471	chr8: 101394991 [+]	101349856	45135	0	153	2	1	0
hsa-mir-4472-1	chr8: 143257700 [+]	143209035	48665	36	0	2	0	0
hsa-mir-4472-2	chr12: 116866123 [-]	116892824	26701	0	81	1	0	0
hsa-mir-4475	chr9: 36823596 [-]	36859598	36002	28	0	0	0	0
hsa-mir-4477a	chr9: 68415388 [-]	68455349	39961	129	0	9	7	0
hsa-mir-4477b	chr9: 68415308 [+]	68413228	2080	11	0	62	27	0
hsa-mir-4478	chr9: 124882446 [-]	124922058	39612	1	259696	50	186	0
hsa-mir-3155b	chr10: 6194225 [-]	6206657	12432	0	232	3	0	0
hsa-mir-548ak	chr10: 12172815 [-]	12221128	48313	0	81	6	17	0
hsa-mir-4482	chr10: 106028163 [-]	106072210	44047	0	172	20	11	0
hsa-mir-4483	chr10: 115537813 [-]	115546735	8922	18	0	1	0	0
hsa-mir-4484	chr10: 127508309 [+]	127461578	46731	85	0	3	0	0
hsa-mir-4488	chr11: 61276068 [+]	61248591	27477	24	0	4	67	0
hsa-mir-548al	chr11: 74110282 [+]	74108651	1631	0	43	19	1	0
hsa-mir-4491	chr11: 111218482 [+]	111170033	48449	14	0	17	4	0
hsa-mir-4492	chr11: 118781417 [+]	118754603	26814	0	19	13	61	0
hsa-mir-4493	chr11: 123252220 [-]	123301784	49564	17	0	56	1	0
hsa-mir-4495	chr12: 98332899 [-]	98348741	15842	80	0	3	0	0
hsa-mir-4496	chr12: 109029586 [+]	109022443	7143	6	0	55	1	0
hsa-mir-4502	chr13: 115039303 [+]	115000422	38881	1	375	21	69	0
hsa-mir-4505	chr14: 74225450 [+]	74186050	39400	0	456	11	11	0
hsa-mir-2392	chr14: 101280828 [+]	101255501	25327	29	0	2	0	0
hsa-mir-4508	chr15: 23807278 [-]	23810584	3306	0	151	1	0	0
hsa-mir-4509-2	chr15: 28671637 [+]	28664048	7589	25	0	0	0	0
hsa-mir-4509-3	chr15: 28735991 [-]	28737891	1900	22	0	0	0	0
hsa-mir-4510	chr15: 36219057 [+]	36177883	41174	20	0	3	0	0
hsa-mir-4513	chr15: 75081098 [-]	75128649	47551	0	58	4	8	0
hsa-mir-4514	chr15: 81289814 [-]	81293857	4043	7	0	53	11	0
hsa-mir-4515	chr15: 83736087 [+]	83735824	263	546	0	76	0	0

hsa-mir-4516	chr16: 2183120 [+]	2141925	41195	36	0	19	1	0.18055
hsa-mir-4520a	chr17: 6558828 [-]	6562992	4164	123	0	2	0	0
hsa-mir-4521	chr17: 8090263 [+]	8063611	26652	0	114	9	91	0
hsa-mir-1269b	chr17: 12820659 [-]	12868660	48001	33	0	3	0	0
hsa-mir-4522	chr17: 25621022 [-]	25630540	9518	0	105	7	10	0
hsa-mir-4523	chr17: 27717680 [+]	27717359	321	21	389	54	4	0
hsa-mir-4529	chr18: 53146452 [+]	53114480	31972	0	177	1	0	0
hsa-mir-4530	chr19: 39900318 [-]	39926588	26270	54	71332	80	2208	0
hsa-mir-4531	chr19: 45157002 [-]	45165729	8727	0	375	4	0	0
hsa-mir-4532	chr20: 56470450 [+]	56425864	44586	219	0	1	0	0
hsa-mir-4534	chr22: 38384801 [+]	38349706	35095	7	3214464	48	213	0
hsa-mir-378i	chr22: 42319301 [-]	42343128	23827	13	991	70	93	0
hsa-mir-4535	chr22: 49176107 [+]	49165515	10592	281	0	3	1	0
hsa-mir-1587	chrX: 39696815 [+]	39680696	16119	31	0	11	0	0
hsa-mir-4536-1	chrX: 55478015 [-]	55515262	37247	8	0	55	1	0
hsa-mir-3976	chr18: 5840694 [+]	5802416	38278	64	0	0	0	0
hsa-mir-4634	chr5: 174178737 [+]	174151591	27146	6	100	18	29	0
hsa-mir-4638	chr5: 180649633 [-]	180670906	21273	48	45455	73	6173	0
hsa-mir-4645	chr6: 2854341 [-]	2903544	49203	1592	0	70	61	0
hsa-mir-4656	chr7: 4828270 [-]	4847260	18990	33	0	2	0	0
hsa-mir-4660	chr8: 8905955 [+]	8860439	45516	20	5104	46	58	0
hsa-mir-4659b	chr8: 6602761 [-]	6612636	9875	0	5869	3	1	0
hsa-mir-4663	chr8: 124228103 [-]	124253617	25514	1	890	20	47	0
hsa-mir-4662b	chr8: 125834300 [-]	125852767	18467	69	0	12	0	0
hsa-mir-4665	chr9: 6007826 [+]	6007156	670	0	6	26	0	0
hsa-mir-4666a	chr1: 228649775 [+]	228645804	3971	1	2098	30	10	0
hsa-mir-2964a	chr9: 131154900 [+]	131133651	21249	9	1406	51	155	0
hsa-mir-4674	chr9: 139440711 [-]	139461472	20761	73	0	3	0	0
hsa-mir-4675	chr10: 20840899 [+]	20794634	46265	204	0	1	0	0
hsa-mir-4678	chr10: 89263638 [+]	89253140	10498	19	0	3	0	0
hsa-mir-4682	chr10: 121718025 [+]	121700321	17704	0	151	6	101	0

hsa-mir-4686	chr11: 2194293 [+]	2153879	40414	10	0	0	156	0
hsa-mir-4689	chr1: 5922801 [-]	5956874	34073	0	45	4	9	0
hsa-mir-4692	chr11: 72494575 [+]	72492896	1679	8	0	7	3	0
hsa-mir-4694	chr11: 19781629 [-]	19798661	17032	73	0	3	0	0
hsa-mir-4703	chr13: 52126725 [+]	52077672	49053	0	37	3	0	0
hsa-mir-4708	chr14: 65801901 [-]	65807982	6081	90	0	0	0	0
hsa-mir-203b	chr14: 104583840 [-]	104604175	20335	119	0	16	1	0
hsa-mir-4710	chr14: 105144086 [-]	105173863	29777	18	0	10	4	0
hsa-mir-4712	chr15: 50652526 [+]	50647186	5340	0	65	57	18	0
hsa-mir-4713	chr15: 51534387 [+]	51492977	41410	26	0	0	0	0
hsa-mir-4716	chr15: 49461350 [-]	49470980	9630	105	0	7	0	0
hsa-mir-3529	chr15: 89155155 [-]	89195596	40441	7	0	30	1	0
hsa-mir-4717	chr16: 2324621 [+]	2303924	20697	0	97	3	31	0.2926
hsa-mir-4718	chr16: 12814178 [+]	12798424	15754	0	62	13	2	0
hsa-mir-4720	chr16: 81418623 [+]	81414373	4250	8	0	4	0	0
hsa-mir-4520b	chr17: 6558768 [+]	6544380	14388	0	16491	84	151	0
hsa-mir-451b	chr17: 27188389 [+]	27182033	6356	10163	0	63	429	0
hsa-mir-4725	chr17: 29902288 [+]	29860524	41764	0	208	9	3	0.02938
hsa-mir-4727	chr17: 36982091 [+]	36977199	4892	0	5539	7	4	0
hsa-mir-4732	chr17: 27188748 [-]	27224681	35933	2061	0	32	143	0.68008
hsa-mir-4733	chr17: 29421443 [-]	29423139	1696	0	219	17	1	0
hsa-mir-4734	chr17: 36858584 [-]	36890783	32199	1	0	8	34	0
hsa-mir-4736	chr17: 56413383 [-]	56429562	16179	1	53849	32	273	0.46595
hsa-mir-4737	chr17: 58120466 [-]	58165676	45210	0	2052	2	115	0
hsa-mir-4738	chr17: 73780688 [-]	73821301	40613	39	0	5	0	0
hsa-mir-4739	chr17: 77681058 [-]	77711077	30019	103	0	3	0	0
hsa-mir-4740	chr17: 79374578 [-]	79387018	12440	62	0	8	0	0
hsa-mir-3591	chr18: 56118384 [-]	56133681	15297	39	0	6	0	0
hsa-mir-4746	chr19: 4445975 [+]	4402638	43337	3	22805	15	33	0
hsa-mir-4752	chr19: 54785964 [+]	54780709	5255	57	0	1	0	0
hsa-mir-371b	chr19: 54290996 [-]	54330189	39193	25	0	2	0	0

hsa-mir-4754	chr19: 58898225 [-]	58919821	21596	0	99	89	20	0
hsa-mir-499b	chr20: 33578275 [-]	33589421	11146	23	0	7	1	0
hsa-mir-4757	chr2: 19548190 [+]	19547863	327	0	18	54	0	0
hsa-mir-4759	chr21: 28326280 [+]	28307013	19267	34	0	4	0	0
hsa-mir-4769	chrX: 47446828 [+]	47420605	26223	573	245	27	348	0
hsa-mir-4770	chrX: 6302004 [-]	6340651	38647	0	40	0	1	0
hsa-mir-4778	chr2: 66585460 [-]	66618646	33186	0	76	3	2	0
hsa-mir-4780	chr2: 88382118 [-]	88427578	45460	0	18818	0	91	0
hsa-mir-4783	chr2: 128181194 [-]	128181785	591	0	16	2	0	0
hsa-mir-1245b	chr2: 189842887 [-]	189860876	17989	161	0	1	1	0
hsa-mir-4786	chr2: 240882511 [-]	240900303	17792	0	15	5	9	0
hsa-mir-4787	chr3: 50712511 [+]	50692819	19692	128	0	6	0	0
hsa-mir-4788	chr3: 134156669 [+]	134151287	5382	0	187	2	1	0
hsa-mir-4792	chr3: 24562926 [-]	24563333	407	10	0	5	0	0
hsa-mir-4795	chr3: 87275427 [-]	87276353	926	0	27	90	2	0
hsa-mir-3688-2	chr4: 160049957 [+]	160025306	24651	0	236	31	1	0
hsa-mir-4801	chr4: 37243613 [-]	37246180	2567	76	0	3	0	0
hsa-mir-5001	chr2: 233415283 [-]	233415301	18	237	0	70	16	0
hsa-mir-548ap	chr15: 86368866 [+]	86337798	31068	19	0	28	0	0.35661
hsa-mir-5091	chr4: 13629489 [+]	13629476	13	6	0	78	6	0
hsa-mir-5186	chr3: 151283783 [-]	151289858	6075	35	0	0	0	0
hsa-mir-5188	chr12: 125400093 [+]	125397751	2342	0	81	8	32	0
hsa-mir-4436b-2	chr2: 111042430 [+]	111071972	29542	8	0	7	2	0
hsa-mir-4444-2	chr3: 75263627 [+]	75263621	6	14	0	0	1	0
hsa-mir-3670-2	chr16: 16400227 [+]	16398348	1879	44	0	0	0	0.00373
hsa-mir-4524b	chr17: 67095683 [+]	67091860	3823	23	0	0	0	0
hsa-mir-5100	chr10: 43493011 [+]	43471255	21756	77	0	4	0	0
hsa-mir-5580	chr14: 54415202 [-]	54420113	4911	1	2901	10	41	0
hsa-mir-5589	chr19: 10149030 [+]	10107402	41628	147	0	1	0	0
hsa-mir-4536-2	chrX: 55477928 [+]	55477725	203	1	0	1	0	0
hsa-mir-5680	chr8: 103137660 [+]	103136502	1158	38	0	19	0	0

hsa-mir-5681a	chr8: 75460778 [+]	75434575	26203	27	0	0	0	0
hsa-mir-548aw	chr9: 135821094 [+]	135785966	35128	0	827	9	3	0
hsa-mir-5684	chr19: 12897942 [+]	12849375	48567	0	6	11	25	0
hsa-mir-5685	chr6: 53141791 [+]	53139974	1817	0	174	19	1	0
hsa-mir-5692c-1	chr5: 135138764 [-]	135160134	21370	47	0	1	0	0
hsa-mir-5686	chr10: 98901440 [+]	98875963	25477	29	43	2	0	0
hsa-mir-5689	chr6: 10439950 [+]	10398233	41717	0	2	0	31	0
hsa-mir-5693	chr13: 51922775 [-]	51968347	45572	0	32	5	4	0
hsa-mir-5694	chr14: 67908647 [-]	67955439	46792	56	0	62	10	0
hsa-mir-5695	chr19: 13031134 [+]	13001973	29161	608	0	46	203	0
hsa-mir-5696	chr2: 101925912 [+]	101878935	46977	0	399	16	18	0
hsa-mir-5700	chr12: 94955565 [+]	94952878	2687	83	0	4	0	0
hsa-mir-5702	chr2: 227523509 [-]	227526624	3115	0	44	2	0	0
hsa-mir-5703	chr2: 228336848 [+]	228335845	1003	1	0	6	0	0
hsa-mir-5692b	chr21: 44371124 [-]	44394143	23019	1	0	36	1	0
hsa-mir-5705	chr4: 88221735 [-]	88244004	22269	0	80	4	13	0
hsa-mir-5707	chr7: 158384308 [+]	158380401	3907	37	0	14	0	0
hsa-mir-5739	chr22: 28855857 [+]	28838831	17026	1	0	42	1	0
hsa-mir-6068	chr1: 63792655 [-]	63803504	10849	109	0	0	0	0
hsa-mir-6069	chr22: 35732792 [-]	35781303	48511	150	0	0	0	0
hsa-mir-6072	chr10: 2118283 [-]	2128781	10498	59	0	1	0	0
hsa-mir-6076	chr14: 50433117 [+]	50433105	12	0	59	4	0	0
hsa-mir-6078	chr10: 4033352 [+]	3985545	47807	2	0	9	0	0
hsa-mir-6080	chr17: 62776877 [+]	62776597	280	0	36	25	0	0
hsa-mir-6082	chr4: 172107335 [+]	172104244	3091	14	0	0	0	0
hsa-mir-6085	chr15: 62635228 [+]	62624089	11139	43	0	0	0	0
hsa-mir-6087	chrX: 108297772 [+]	108285189	12583	353	0	0	0	0
hsa-mir-6088	chr19: 45939912 [+]	45909902	30010	1	669	38	31	0
hsa-mir-6089-1	chrX: 2527232 [+]	2502378	24854	22	0	0	0	0
hsa-mir-6090	chr11: 128392285 [+]	128350576	41709	0	2346	22	1	0
hsa-mir-6126	chr16: 3535469 [-]	3559585	24116	51	0	8	0	0

hsa-mir-6127	chr1: 22959859 [-]	22987861	28002	0	430	4	123	0
hsa-mir-6129	chr17: 47365816 [-]	47372897	7081	1	0	2	6	0
hsa-mir-6131	chr5: 10478149 [+]	10442004	36145	0	30	70	64	0
hsa-mir-6134	chrX: 28513780 [-]	28544415	30635	216	0	0	0	0
hsa-mir-548ay	chr3: 32547881 [-]	32576093	28212	0	26	5	13	0
hsa-mir-6500	chr1: 51525690 [+]	51519207	6483	81	0	2	0	0
hsa-mir-548az	chr8: 120337411 [+]	120304274	33137	36	0	1	0	0
hsa-mir-6503	chr11: 59976629 [-]	60010561	33932	1	12	0	1	0
hsa-mir-6511a-1	chr16: 15019794 [+]	14989917	29877	0	14	3	11	0.12409
hsa-mir-6715b	chr10: 114059446 [-]	114102545	43099	40	0	0	0	0
hsa-mir-6720	chr6: 1390646 [-]	1390760	114	1	0	18	0	0
hsa-mir-6723	chr1: 567793 [-]	569481	1688	13	0	0	268	0
hsa-mir-892c	chrX: 145074344 [-]	145078831	4487	37	0	2	0	3.90E-04
hsa-mir-6089-2	chrY: 2477232 [+]	2452378	24854	22	0	0	0	0
hsa-mir-6511a-3	chr16: 16462733 [+]	16434198	28535	19	0	0	20	0

Table S2. The number of members of 230 miRNA co-expressed gene groups.

miRNA	Co-expressed gene #
hsa-let-7d	2
hsa-let-7f-1	4
hsa-let-7f-2	4
hsa-let-7i	47
hsa-mir-101-1	8
hsa-mir-101-2	8
hsa-mir-103a-1	2
hsa-mir-103a-2	2
hsa-mir-106a	18
hsa-mir-107	36
hsa-mir-10a	6
hsa-mir-10b	1
hsa-mir-1-1	884
hsa-mir-1-2	884
hsa-mir-122	261
hsa-mir-124-1	493
hsa-mir-124-2	493
hsa-mir-124-3	493
hsa-mir-125a	7
hsa-mir-126	12
hsa-mir-127	43
hsa-mir-128-1	443
hsa-mir-128-2	443
hsa-mir-129-1	502
hsa-mir-129-2	502
hsa-mir-130a	206
hsa-mir-130b	2
hsa-mir-132	424
hsa-mir-133a-1	2597
hsa-mir-133a-2	2597
hsa-mir-133b	2735
hsa-mir-134	42
hsa-mir-135a-1	56
hsa-mir-135a-2	56
hsa-mir-135b	215
hsa-mir-137	440
hsa-mir-138-2	52
hsa-mir-139	51
hsa-mir-141	35
hsa-mir-142	23
hsa-mir-143	26
hsa-mir-145	11
hsa-mir-146a	25
hsa-mir-146b	12

hsa-mir-148a	40
hsa-mir-148b	38
hsa-mir-149	51
hsa-mir-150	20
hsa-mir-151a	1
hsa-mir-152	2
hsa-mir-153-1	397
hsa-mir-153-2	397
hsa-mir-154	25
hsa-mir-155	23
hsa-mir-15a	1
hsa-mir-15b	6
hsa-mir-16-1	1
hsa-mir-16-2	1
hsa-mir-17	35
hsa-mir-181a-1	9
hsa-mir-181a-2	9
hsa-mir-181b-1	223
hsa-mir-181b-2	223
hsa-mir-181c	2
hsa-mir-181d	61
hsa-mir-183	19
hsa-mir-184	176
hsa-mir-188	14
hsa-mir-18a	1
hsa-mir-190a	107
hsa-mir-191	132
hsa-mir-192	97
hsa-mir-193b	215
hsa-mir-194-1	119
hsa-mir-194-2	119
hsa-mir-196a-1	17
hsa-mir-196a-2	17
hsa-mir-196b	5
hsa-mir-197	78
hsa-mir-198	238
hsa-mir-199a-1	1
hsa-mir-199a-2	1
hsa-mir-199b	18
hsa-mir-200a	2
hsa-mir-200b	6
hsa-mir-200c	31
hsa-mir-202	312
hsa-mir-203a	16
hsa-mir-204	83
hsa-mir-205	4

hsa-mir-206	2373
hsa-mir-20b	2
hsa-mir-210	30
hsa-mir-211	122
hsa-mir-212	3
hsa-mir-215	36
hsa-mir-218-1	16
hsa-mir-218-2	16
hsa-mir-219-1	497
hsa-mir-219-2	497
hsa-mir-22	103
hsa-mir-221	3
hsa-mir-222	6
hsa-mir-223	40
hsa-mir-224	254
hsa-mir-23a	222
hsa-mir-24-1	239
hsa-mir-24-2	239
hsa-mir-25	3
hsa-mir-26a-1	15
hsa-mir-26a-2	15
hsa-mir-26b	113
hsa-mir-27a	287
hsa-mir-27b	1
hsa-mir-28	31
hsa-mir-299	11
hsa-mir-29a	3
hsa-mir-29b-1	175
hsa-mir-29b-2	175
hsa-mir-29c	4
hsa-mir-301a	186
hsa-mir-30a	59
hsa-mir-30b	146
hsa-mir-30c-1	10
hsa-mir-30c-2	10
hsa-mir-30d	269
hsa-mir-30e	94
hsa-mir-31	3
hsa-mir-320a	206
hsa-mir-323a	29
hsa-mir-324	110
hsa-mir-328	289
hsa-mir-330	497
hsa-mir-331	19
hsa-mir-335	244
hsa-mir-338	427

hsa-mir-340	550
hsa-mir-342	4
hsa-mir-345	4
hsa-mir-346	473
hsa-mir-34c	254
hsa-mir-361	2
hsa-mir-362	14
hsa-mir-365a	3
hsa-mir-369	20
hsa-mir-370	33
hsa-mir-371a	263
hsa-mir-372	265
hsa-mir-373	266
hsa-mir-374a	2
hsa-mir-375	40
hsa-mir-376a-1	193
hsa-mir-376a-2	193
hsa-mir-377	263
hsa-mir-378a	1956
hsa-mir-379	14
hsa-mir-381	37
hsa-mir-382	43
hsa-mir-383	273
hsa-mir-409	51
hsa-mir-422a	729
hsa-mir-423	23
hsa-mir-424	254
hsa-mir-425	6
hsa-mir-429	1
hsa-mir-432	25
hsa-mir-433	63
hsa-mir-449a	336
hsa-mir-450a-1	526
hsa-mir-451a	123
hsa-mir-452	263
hsa-mir-485	20
hsa-mir-489	186
hsa-mir-490	107
hsa-mir-491	74
hsa-mir-493	180
hsa-mir-494	32
hsa-mir-495	113
hsa-mir-500a	31
hsa-mir-501	3
hsa-mir-502	5
hsa-mir-503	263

hsa-mir-505	1
hsa-mir-506	356
hsa-mir-507	358
hsa-mir-508	345
hsa-mir-509-1	315
hsa-mir-509-2	315
hsa-mir-509-3	315
hsa-mir-510	350
hsa-mir-511-1	3
hsa-mir-511-2	3
hsa-mir-512-1	261
hsa-mir-512-2	261
hsa-mir-513a-1	358
hsa-mir-513a-2	358
hsa-mir-514a-1	357
hsa-mir-514a-2	357
hsa-mir-514a-3	357
hsa-mir-515-1	261
hsa-mir-515-2	261
hsa-mir-517a	783
hsa-mir-518b	261
hsa-mir-518c	260
hsa-mir-518f	261
hsa-mir-519b	263
hsa-mir-519c	261
hsa-mir-519d	261
hsa-mir-519e	263
hsa-mir-520a	263
hsa-mir-520b	256
hsa-mir-520c	255
hsa-mir-520e	262
hsa-mir-520f	249
hsa-mir-521-2	263
hsa-mir-523	263
hsa-mir-524	263
hsa-mir-525	263
hsa-mir-526a-1	263
hsa-mir-526b	263
hsa-mir-7-1	44
hsa-mir-7-3	44
hsa-mir-9-1	499
hsa-mir-9-2	499
hsa-mir-93	5
hsa-mir-9-3	499
hsa-mir-95	2091
hsa-mir-96	18

hsa-mir-98	150
hsa-mir-99b	174

Table S3. The full list of putative TFs of 225 human miRNAs.

hsa-let-7d

Matrix_id	transcription factor	Gene	PCC	Occurrence
V$NR1B2_Q6	NR1B2	RARB	0.217843	3
V$E2F1_Q3	E2F-1	E2F1	0.212664	3
V$TBX5_02	TBX5	TBX5	0.13954	3
V$E2F1_Q6_01	E2F-1	E2F1	0.212664	3
V$AP2REP_01	AP-2rep	KLF12	0.201937	3
V$RNF96_01	RNF96	TRIM28	0.0106128	3
V$TTF1_Q5	TTF-1	NKX2-1	0.56838	3
V$HNF4A_Q6_01	HNF-4alpha	HNF4A	0.0239993	3
V$HIF1A_Q6	HIF-1alpha	HIF1A	0.346466	3
V$PUR1_Q4	PUR1	PURA	0.370554	3
V$PAX8_01	Pax-8	PAX8	0.664907	3
V$SOX9_Q4	SOX9	SOX9	0.157758	3
V$MAFB_01	MAFB	MAFB	0.0648627	3
V$CDX2_Q5_02	CDX-2	CDX2	0.132877	3
V$LRF_Q2	LRF	ZBTB7A	0.208006	3
V$NKX32_01	Nkx3-2	NKX3-2	0.150967	3
V$IK_Q5	Ikaros	IKZF1	0.162701	3
V$SP1_01	Sp1	SP1	0.16392	3
V$ARNT_01	Arnt	ARNT	0.115811	3
V$YY1_01	YY1	YY1	0.40292	3
V$CDX2_01	Cdx-2	CDX2	0.132877	2
V$TCF4_01	TCF-4	TCF7L2	0.181813	2
V$ZBP89_Q4	ZBP89	ZNF148	0.49716	2
V$GABPA_Q4	GABP-alpha	GABPA	0.0665612	2
V$ZFX_01	Zfx	ZFX	0.266894	2
V$PDEF_02	PDEF	SPDEF	0.182224	2
V$NKX22_02	NKX2B	NKX2-2	0.142135	2
V$SP1_02	SP1	SP1	0.16392	2
V$BEN_01	BEN	GTF2IRD1	0.324806	2
V$ERG_01	ERG	ERG	0.0322723	2
V$AP2BETA_Q3	AP-2beta	TFAP2B	0.151932	2
V$AP4_Q6_02	AP-4	TFAP4	0.520819	2
V$CETS1_Q6	C-ets-1	ETS1	0.108727	2
V$SPI1_03	SPI1	SPI1	0.0691841	2
V$PET1_02	Pet-1	FEV	0.263222	2
V$ETV7_01	ETV7	ETV7	0.17368	2
V$TEL1_02	TEL1	ETV6	0.220726	2
V$ETV3_02	ETV3	ETV3	0.0164795	2
V$ER71_02	ER71	ETV2	0.136421	2
V$ERG_03	ERG	ERG	0.0322723	2
V$ERF_02	ERF	ERF	0.19843	2

V$ELK1_06	ELK-1	ELK1	0.17819	2
V$Elf5_03	ELF5	ELF5	0.197539	2
V$EHF_03	EHF	EHF	0.181625	2
V$WT1_Q6_01	WT1	WT1	0.0142041	2
V$PBX1_Q3	Pbx1	PBX1	0.460525	2
V$EGR1_02	EGR-1	EGR1	0.335899	2
V$YY1_Q6_03	YY1	YY1	0.40292	2
V$SMAD4_Q6_01	Smad4	SMAD4	0.308817	2
V$GABPBETA_Q3	GABP-beta	GABPB1	0.376782	2
V$NKX2B_Q3	NKX2B	NKX2-2	0.142135	2
V$ETS2_Q6	c-Ets-2	ETS2	0.204252	2
V$TFIII_Q6	TFII-I	GTF2I	0.387066	2
V$SP1_Q6_01	Sp1	SP1	0.16392	2
V$SP1_Q6	Sp1	SP1	0.16392	2
V$YY1_Q6	YY1	YY1	0.40292	2
V$PAX3_B	Pax-3	PAX3	0.443837	2
V$GATA1_01	GATA-1	GATA1	0.353108	2
V$ZIC3_01	Zic3	ZIC3	0.265518	2
V$ELF1_Q6	Elf-1	ELF1	0.148091	2
V$MAZ_Q6	MAZ	MAZ	0.12945	2
V$PEA3_Q6	PEA3	ETV4	0.0404392	2
V$SMAD3_Q6	SMAD3	SMAD3	0.284121	2
V$SP1_Q4_01	Sp1	SP1	0.16392	2
V$SP1_Q2_01	Sp1	SP1	0.16392	2
V$CMYB_01	c-Myb	MYB	0.274576	2
V$SAP1A_01	SAP-1a	ELK4	0.158937	2
V$ELK1_01	Elk-1	ELK1	0.17819	2
V$ELK1_02	Elk-1	ELK1	0.17819	2
V$E2F1_Q3_01	E2F-1	E2F1	0.212664	2
V$STAF_02	Staf	ZNF143	0.263938	1
V$E12_Q6	E12	TCF3	0.153493	1
V$MAZ_Q6_01	MAZ	MAZ	0.12945	1
V$SP3_Q3	Sp3	SP3	0.237637	1
V$MAX_01	Max	MAX	0.321685	1
V$MYOD_Q6	MyoD	MYOD1	0.0331499	1
V$YY1_03	YY1	YY1	0.40292	1
V$E4F1_Q6_01	E4F1	E4F1	0.504233	1
V$ARNT_02	Arnt	ARNT	0.115811	1
V$ZID_01	ZID	ZBTB6	0.0471627	1
V$ZIC1_01	Zic1	ZIC1	0.256711	1
V$E47_02	E47	TCF3	0.153493	1
V$SPIB_03	Spi-B	SPIB	0.0665149	1
V$E2F1_Q6	E2F-1	E2F1	0.212664	1
V$OC2_Q3	OC-2	ONECUT2	0.320577	1
V$TEF1_Q6	TEF-1	TEAD1	0.205679	1
V$MYOGENIN_Q6	myogenin	MYOG	0.122675	1

V$E2A_Q2	E2A	TCF3	0.153493	1
V$MYOD_Q6_01	MyoD	MYOD1	0.0331499	1
V$PBX1_04	Pbx1	PBX1	0.460525	1
V$E2A_Q6	E2A	TCF3	0.153493	1
V$YY1_Q6_02	YY1	YY1	0.40292	1
V$IRF3_Q3	IRF-3	IRF3	0.340956	1
V$SP4_Q5	SP4	SP4	0.27079	1
V$CNOT3_01	CNOT3	CNOT3	0.10391	1
V$WT1_Q6	WT1	WT1	0.0142041	1
V$ELF5_01	ELF5	ELF5	0.197539	1
V$BEN_02	BEN	GTF2IRD1	0.324806	1
V$SP2_01	SP2	SP2	0.390237	1
V$TEF1_Q6_03	TEF-1	TEAD1	0.205679	1
V$SMAD3_Q6_01	Smad3	SMAD3	0.284121	1
V$USF2_Q6	USF2	USF2	0.481291	1
V$SMAD4_Q6	SMAD4	SMAD4	0.308817	1
V$ATF4_Q6	ATF-4	ATF4	0.141526	1
V$ATF2_Q5	ATF-2	ATF2	0.381982	1
V$ATF1_Q6_01	ATF-1	ATF1	0.646826	1
V$AML1_Q6	AML1	RUNX1	0.235418	1
V$DEC2_Q2	2-Dec	BHLHE41	0.563678	1
V$MAX_Q6	MAX	MAX	0.321685	1
V$CREM_Q6	CREM	CREM	0.122916	1
V$PARP_Q3	PARP	PARP1	0.104683	1

----

hsa-let-7f-1

Matrix_id	transcription factor	Gene	PCC	Occurrence
V$TCF4_Q5	TCF-4	TCF7L2	0.167176	5
V$SOX9_B1	SOX9	SOX9	0.166783	5
V$MAFB_01	MAFB	MAFB	0.125766	5
V$ZIC3_01	Zic3	ZIC3	0.476662	5
V$NKX32_01	Nkx3-2	NKX3-2	0.21476	5
V$IK_Q5	Ikaros	IKZF1	0.120097	5
V$HNF4A_Q6_01	HNF-4alpha	HNF4A	0.275799	5
V$PARP_Q4	PARP	PARP1	0.12812	5
V$SRY_02	SRY	SRY	0.179	5
V$SOX9_Q4	SOX9	SOX9	0.166783	5
V$P300_01	p300	EP300	0.262498	5
V$SOX5_01	SOX5	SOX5	0.216083	5
V$YY1_01	YY1	YY1	0.396304	5
V$GATA1_01	GATA-1	GATA1	0.475532	5
V$NR1B2_Q6	NR1B2	RARB	0.359067	5

V$PUR1_Q4	PUR1	PURA	0.346986	5
V$TBP_Q6	TBP	TBP	0.0323614	4
V$ELF1_Q6	Elf-1	ELF1	0.0247277	4
V$HIF1A_Q6	HIF-1alpha	HIF1A	0.334539	4
V$BEN_01	BEN	GTF2IRD1	0.33885	4
V$IPF1_01	IPF1	PDX1	0.350797	4
V$ETS2_Q6	c-Ets-2	ETS2	0.157383	4
V$TBX5_02	TBX5	TBX5	0.225643	4
V$PARP_Q3	PARP	PARP1	0.12812	4
V$CMYB_Q5	c-Myb	MYB	0.064287	4
V$SP2_01	SP2	SP2	0.505953	4
V$FKLF_Q5	FKLF	KLF11	0.141546	4
V$NFAT2_Q5	NF-AT2	NFATC1	0.297007	4
V$TCF4_01	TCF-4	TCF7L2	0.167176	4
V$CETS1_Q6	C-ets-1	ETS1	0.333032	4
V$TCF3_01	TCF-3	TCF7L1	0.409469	4
V$ERBETA_Q5	ER-beta	ESR2	0.0658926	4
V$IRF4_Q6	IRF-4	IRF4	0.343543	4
V$NFAT4_Q3	NF-AT4	NFATC3	0.0345672	4
V$SMAD3_Q6_01	Smad3	SMAD3	0.261458	4
V$SMAD4_Q6_01	Smad4	SMAD4	0.326552	4
V$HNF1_02	HNF-1alpha	HNF1A	0.236484	4
V$YY1_Q6_03	YY1	YY1	0.396304	4
V$Elf5_03	ELF5	ELF5	0.206314	4
V$ER71_02	ER71	ETV2	0.400414	4
V$GATA2_02	GATA-2	GATA2	0.0627379	4
V$ARNT_01	Arnt	ARNT	0.193224	4
V$GATA6_01	GATA-6	GATA6	0.179109	4
V$GATA1_04	GATA-1	GATA1	0.475532	4
V$SP1_Q6	Sp1	SP1	0.222982	4
V$TEL1_02	TEL1	ETV6	0.202309	4
V$GATA1_06	GATA-1	GATA1	0.475532	4
V$PEA3_Q6	PEA3	ETV4	0.0580077	4
V$SP1_01	Sp1	SP1	0.222982	4
V$GR_Q6	GR	NR3C1	0.38144	4
V$CDX2_Q5_02	CDX-2	CDX2	0.187121	4
V$MYB_Q6	c-Myb	MYB	0.064287	4
V$GATA1_02	GATA-1	GATA1	0.475532	4
V$OC2_Q3	OC-2	ONECUT2	0.441936	4
V$ETV3_02	ETV3	ETV3	0.0724637	4
V$TFIII_Q6	TFII-I	GTF2I	0.522362	4
V$ELK1_02	Elk-1	ELK1	0.331426	4
V$GATA1_05	GATA-1	GATA1	0.475532	4
V$AP2REP_01	AP-2rep	KLF12	0.250664	4
V$GATA2_03	GATA-2	GATA2	0.0627379	3
V$LHX3b_01	LHX3b	LHX3	0.353531	3

V$SP1_02	SP1	SP1	0.222982	3
V$MEIS1_01	MEIS1	MEIS1	0.0341909	3
V$NKX22_02	NKX2B	NKX2-2	0.0441284	3
V$NCX_02	Ncx	TLX2	0.179059	3
V$ETS1_B	c-Ets-1	ETS1	0.333032	3
V$MITF_Q6	MITF	MITF	0.371673	3
V$GABPBETA_Q3	GABP-beta	GABPB1	0.459125	3
V$NKX2B_Q3	NKX2B	NKX2-2	0.0441284	3
V$FOXP3_01	FOXP3	FOXP3	0.226657	3
V$EHF_03	EHF	EHF	0.0714727	3
V$ZBP89_Q4	ZBP89	ZNF148	0.362883	3
V$EGR1_02	EGR-1	EGR1	0.274623	3
V$IPF1_Q6	IPF1	PDX1	0.350797	3
V$ETV7_01	ETV7	ETV7	0.240579	3
V$GABPA_Q4	GABP-alpha	GABPA	0.0261077	3
V$TBX5_01	TBX5	TBX5	0.225643	3
V$TTF1_Q5	TTF-1	NKX2-1	0.549287	3
V$ESE1_02	ESE-1	ELF3	0.0302076	3
V$ZFX_01	Zfx	ZFX	0.225822	3
V$SP1_Q4_01	Sp1	SP1	0.222982	3
V$SP1_Q6_01	Sp1	SP1	0.222982	3
V$ELK1_06	ELK-1	ELK1	0.331426	3
V$ERF_02	ERF	ERF	0.199647	3
V$LRF_Q2	LRF	ZBTB7A	0.366758	3
V$FOXO3A_Q1	FOXO3A	FOXO3	0.153395	3
V$ESE1_Q3	ESE-1	ELF3	0.0302076	3
V$DAX1_01	Dax1	NR0B1	0.184728	3
V$HNF3B_Q6	HNF-3beta	FOXA2	0.0973956	3
V$SP1_Q2_01	Sp1	SP1	0.222982	3
V$PET1_02	Pet-1	FEV	0.466246	3
V$IPF1_Q4	IPF1	PDX1	0.350797	3
V$FAC1_01	FAC1	BPTF	0.118777	3
V$IPF1_Q4_01	IPF1	PDX1	0.350797	3
V$ZIC1_01	Zic1	ZIC1	0.144175	3
V$MAX_Q6	MAX	MAX	0.444138	2
V$HOXD9_Q2	Hoxd9	HOXD9	0.426109	2
V$PBX1_Q3	Pbx1	PBX1	0.387493	2
V$PDEF_02	PDEF	SPDEF	0.383401	2
V$ERG_03	ERG	ERG	0.153297	2
V$GFI1_Q6_01	Gfi1	GFI1	0.130711	2
V$ATF1_Q6_01	ATF-1	ATF1	0.489274	2
V$MAX_01	Max	MAX	0.444138	2
V$SP4_Q5	SP4	SP4	0.240922	2
V$CNOT3_01	CNOT3	CNOT3	0.25576	2
V$NANOG_02	Nanog	NANOG	0.281388	2
V$SOX10_Q6	SOX10	SOX10	0.16417	2

V$GFI1_Q6	Gfi1	GFI1	0.130711	2
V$YY1_Q6_02	YY1	YY1	0.396304	2
V$CIZ_01	CIZ	ZNF384	0.260205	2
V$SMAD4_Q6	SMAD4	SMAD4	0.326552	2
V$USF2_Q6	USF2	USF2	0.486612	2
V$SMAD3_Q6	SMAD3	SMAD3	0.261458	2
V$ARNT_02	Arnt	ARNT	0.193224	2
V$FOXJ2_01	FOXJ2	FOXJ2	0.295646	2
V$PAX3_B	Pax-3	PAX3	0.534025	2
V$STAT4_Q5	STAT4	STAT4	0.197478	2
V$CDX2_01	Cdx-2	CDX2	0.187121	2
V$RORBETA_Q2	RORBETA	RORB	0.159092	2
V$PITX3_Q2	PITX3	PITX3	0.211989	2
V$CDX2_Q5_01	Cdx-2	CDX2	0.187121	2
V$CREM_Q6	CREM	CREM	0.215295	2
V$HNF1B_01	HNF-1beta	HNF1B	0.307825	2
V$MEIS1_02	Meis1	MEIS1	0.0341909	2
V$SREBP1_01	SREBP-1	SREBF1	0.130259	1
V$DEC2_Q2	2-Dec	BHLHE41	0.403748	1
V$EVI1_02	Evi-1	MECOM	0.285835	1
V$PITX2_Q2	Pitx2	PITX2	0.0241907	1
V$E2F1_Q6	E2F-1	E2F1	0.38014	1
V$E2F1_Q3	E2F-1	E2F1	0.38014	1
V$E2F4_Q6	E2F-4	E2F4	0.0808061	1
V$ERG_01	ERG	ERG	0.153297	1
V$CDX1_01	Cdx-1	CDX1	0.0207746	1
V$PAX8_01	Pax-8	PAX8	0.714106	1
V$CDX2_Q5	Cdx-2	CDX2	0.187121	1
V$HOXA9_01	hoxa9	HOXA9	0.354511	1
V$CRX_02	Crx	CRX	0.220501	1
V$PITX2_01	PITX2	PITX2	0.0241907	1
V$CRX_Q4_01	CRX	CRX	0.220501	1
V$PITX1_01	Pitx1	PITX1	0.231602	1
V$E2F1_Q6_01	E2F-1	E2F1	0.38014	1
V$IRF3_Q3	IRF-3	IRF3	0.462128	1
V$E2F1_Q3_01	E2F-1	E2F1	0.38014	1
V$GFI1B_01	Gfi1b	GFI1B	0.273257	1
V$ATF2_Q5	ATF-2	ATF2	0.386045	1
V$ATF4_Q6	ATF-4	ATF4	0.0610864	1

----

hsa-let-7f-2

Matrix_id	transcription factor	Gene	PCC	Occurrence

V$TCF4_Q5	TCF-4	TCF7L2	0.167176	5
V$PUR1_Q4	PUR1	PURA	0.346986	5
V$ZIC3_01	Zic3	ZIC3	0.476662	5
V$HNF4A_Q6_01	HNF-4alpha	HNF4A	0.275799	5
V$PARP_Q4	PARP	PARP1	0.12812	5
V$SOX9_Q4	SOX9	SOX9	0.166783	5
V$SRY_02	SRY	SRY	0.179	5
V$NR1B2_Q6	NR1B2	RARB	0.359067	5
V$NKX32_01	Nkx3-2	NKX3-2	0.21476	5
V$AML1_Q6	AML1	RUNX1	0.278907	5
V$P300_01	p300	EP300	0.262498	5
V$SOX5_01	SOX5	SOX5	0.216083	5
V$YY1_01	YY1	YY1	0.396304	5
V$MAFB_01	MAFB	MAFB	0.125766	5
V$SOX9_B1	SOX9	SOX9	0.166783	5
V$HNF1_02	HNF-1alpha	HNF1A	0.236484	4
V$ELF1_Q6	Elf-1	ELF1	0.0247277	4
V$HIF1A_Q6	HIF-1alpha	HIF1A	0.334539	4
V$TFIII_Q6	TFII-I	GTF2I	0.522362	4
V$TBP_Q6	TBP	TBP	0.0323614	4
V$YY1_Q6_03	YY1	YY1	0.396304	4
V$TCF4_01	TCF-4	TCF7L2	0.167176	4
V$IPF1_01	IPF1	PDX1	0.350797	4
V$PARP_Q3	PARP	PARP1	0.12812	4
V$ERBETA_Q5	ER-beta	ESR2	0.0658926	4
V$PBX1_04	Pbx1	PBX1	0.387493	4
V$MEF2C_Q4	MEF-2C	MEF2C	0.115352	4
V$FKLF_Q5	FKLF	KLF11	0.141546	4
V$NFAT4_Q3	NF-AT4	NFATC3	0.0345672	4
V$PEA3_Q6	PEA3	ETV4	0.0580077	4
V$YY1_Q6	YY1	YY1	0.396304	4
V$ARNT_01	Arnt	ARNT	0.193224	4
V$NFAT2_Q5	NF-AT2	NFATC1	0.297007	4
V$GR_Q6	GR	NR3C1	0.38144	4
V$GATA1_04	GATA-1	GATA1	0.475532	4
V$GATA1_02	GATA-1	GATA1	0.475532	4
V$OC2_Q3	OC-2	ONECUT2	0.441936	4
V$TCF3_01	TCF-3	TCF7L1	0.409469	4
V$ELK1_02	Elk-1	ELK1	0.331426	4
V$AP2REP_01	AP-2rep	KLF12	0.250664	4
V$AML1_01	AML1a	RUNX1	0.278907	4
V$GATA1_05	GATA-1	GATA1	0.475532	4
V$GATA1_06	GATA-1	GATA1	0.475532	4
V$Elf5_03	ELF5	ELF5	0.206314	4
V$GATA2_02	GATA-2	GATA2	0.0627379	4
V$CDX2_Q5_02	CDX-2	CDX2	0.187121	4

V$GATA6_01	GATA-6	GATA6	0.179109	4
V$HOXB8_01	HOXB8	HOXB8	0.487216	4
V$TEL1_02	TEL1	ETV6	0.202309	4
V$IPF1_Q6	IPF1	PDX1	0.350797	3
V$AML2_01	AML2	RUNX3	0.0752658	3
V$NCX_02	Ncx	TLX2	0.179059	3
V$HOX13_02	HOXA5	HOXA5	0.212458	3
V$NKX22_02	NKX2B	NKX2-2	0.0441284	3
V$IRF7_Q3	IRF-7	IRF7	0.0552326	3
V$NURR1_Q3	NURR1	NR4A2	0.352283	3
V$GATA2_01	GATA-2	GATA2	0.0627379	3
V$DAX1_01	Dax1	NR0B1	0.184728	3
V$NKX2B_Q3	NKX2B	NKX2-2	0.0441284	3
V$FAC1_01	FAC1	BPTF	0.118777	3
V$BCL6_Q3_01	Bcl-6	BCL6	0.196284	3
V$SREBP1_Q6	SREBP-1	SREBF1	0.130259	3
V$HNF6_Q6	HNF6	ONECUT1	0.222769	3
V$IPF1_Q4_01	IPF1	PDX1	0.350797	3
V$CMAF_01	c-Maf	MAF	0.44562	3
V$LHX3b_01	LHX3b	LHX3	0.353531	3
V$FOXO3A_Q1	FOXO3A	FOXO3	0.153395	3
V$GFI1_Q6_01	Gfi1	GFI1	0.130711	2
V$ATF1_Q6_01	ATF-1	ATF1	0.489274	2
V$HOXD9_Q2	Hoxd9	HOXD9	0.426109	2
V$TCF4_Q5_01	TCF-4	TCF7L2	0.167176	2
V$CREM_Q6	CREM	CREM	0.215295	2
V$PBX1_Q3	Pbx1	PBX1	0.387493	2
V$AP4_Q6_02	AP-4	TFAP4	0.479309	2
V$STAT4_Q5	STAT4	STAT4	0.197478	2
V$CDX2_Q5_01	Cdx-2	CDX2	0.187121	2
V$NANOG_02	Nanog	NANOG	0.281388	2
V$IPF1_03	IPF1	PDX1	0.350797	2
V$SOX10_Q6	SOX10	SOX10	0.16417	2
V$GFI1_Q6	Gfi1	GFI1	0.130711	2
V$YY1_Q6_02	YY1	YY1	0.396304	2
V$GR_01	GR	NR3C1	0.38144	2
V$USF2_Q6	USF2	USF2	0.486612	2
V$ARNT_02	Arnt	ARNT	0.193224	2
V$FOXJ2_01	FOXJ2	FOXJ2	0.295646	2
V$MAX_01	Max	MAX	0.444138	2
V$CMYB_01	c-Myb	MYB	0.064287	2
V$IPF1_06	ipf1	PDX1	0.350797	2
V$HNF1B_01	HNF-1beta	HNF1B	0.307825	2
V$CDX2_01	Cdx-2	CDX2	0.187121	2
V$CART1_02	CART1	ALX1	0.300124	2
V$NKX32_02	Nkx3-2	NKX3-2	0.21476	2

V$IRF7_01	IRF-7	IRF7	0.0552326	1
V$LHX3_01	Lhx3	LHX3	0.353531	1
V$POU6F1_01	POU6F1	POU6F1	0.407496	1
V$SREBP1_01	SREBP-1	SREBF1	0.130259	1
V$HSF1_01	HSF1	HSF1	0.431774	1
V$PAX8_01	Pax-8	PAX8	0.714106	1
V$CDP_04	CDP	CUX1	0.328374	1
V$CDX1_01	Cdx-1	CDX1	0.0207746	1
V$GFI1B_01	Gfi1b	GFI1B	0.273257	1
V$DEC2_Q2	2-Dec	BHLHE41	0.403748	1
V$HOXA9_01	hoxa9	HOXA9	0.354511	1
V$ATF2_Q5	ATF-2	ATF2	0.386045	1
V$ATF4_Q6	ATF-4	ATF4	0.0610864	1
V$SOX2_Q6	SOX2	SOX2	0.179148	1
V$EVI1_06	Evi-1	MECOM	0.285835	1

----

hsa-let-7i

Matrix_id	transcription factor	Gene	PCC	Occurrence
V$PUR1_Q4	PUR1	PURA	0.0633072	45
V$SMAD4_Q6_01	Smad4	SMAD4	0.0879548	44
V$ELF1_Q6	Elf-1	ELF1	0.247528	43
V$ZIC3_01	Zic3	ZIC3	0.0529918	42
V$GKLF_Q4	GKLF	KLF4	0.112727	42
V$GATA1_01	GATA-1	GATA1	0.0640399	42
V$SMAD3_Q6_01	Smad3	SMAD3	0.0226512	41
V$SOX9_Q4	SOX9	SOX9	0.0735218	41
V$GR_Q6	GR	NR3C1	0.12375	38
V$GATA1_02	GATA-1	GATA1	0.0640399	35
V$TTF1_Q5	TTF-1	NKX2-1	0.716708	35
V$SOX9_B1	SOX9	SOX9	0.0735218	33
V$Elf5_03	ELF5	ELF5	0.061747	33
V$AP4_Q6_02	AP-4	TFAP4	0.19314	33
V$YY1_01	YY1	YY1	0.376342	33
V$GATA1_06	GATA-1	GATA1	0.0640399	32
V$GATA1_05	GATA-1	GATA1	0.0640399	32
V$GABPA_Q4	GABP-alpha	GABPA	0.0737584	31
V$SPI1_Q5	SPI1	SPI1	0.0276628	30
V$GATA1_04	GATA-1	GATA1	0.0640399	29
V$PBX1_04	Pbx1	PBX1	0.385119	29
V$YY1_Q6_03	YY1	YY1	0.376342	26
V$AP4_Q5	AP-4	TFAP4	0.19314	24
V$ZBP89_Q4	ZBP89	ZNF148	0.147627	24

V$TCF3_01	TCF-3	TCF7L1	0.0502226	24
V$TCF4_Q5	TCF-4	TCF7L2	0.349534	23
V$HBP1_Q2	hbp1	HBP1	0.0161133	22
V$TCF4_01	TCF-4	TCF7L2	0.349534	21
V$SREBP1_Q6	SREBP-1	SREBF1	0.0459608	20
V$MSX1_01	Msx-1	MSX1	0.0771198	20
V$AP4_Q6	AP-4	TFAP4	0.19314	20
V$SMAD3_Q6	SMAD3	SMAD3	0.0226512	19
V$GR_01	GR	NR3C1	0.12375	19
V$MEF2A_Q6	mef2A	MEF2A	0.243245	17
V$AP4_Q6_01	AP-4	TFAP4	0.19314	15
V$DBP_Q6_01	DBP	DBP	0.257961	13
V$STAT1_05	STAT1	STAT1	0.272389	11
V$HOXB8_01	HOXB8	HOXB8	0.0457218	11
V$HOX13_02	HOXA5	HOXA5	0.0135114	11
V$HSF1_01	HSF1	HSF1	0.0269921	10
V$AMEF2_Q6	aMEF-2	MEF2A	0.243245	7
V$GRE_C	GR	NR3C1	0.12375	4
V$MEF2A_05	MEF2A	MEF2A	0.243245	1

hsa-mir-101-1

Matrix_id	transcription factor	Gene	PCC	Occurrence
V$PEA3_Q6	PEA3	ETV4	0.682685	9
V$PARP_Q4	PARP	PARP1	0.127428	9
V$P300_01	p300	EP300	0.50372	9
V$PUR1_Q4	PUR1	PURA	0.393818	9
V$SMAD3_Q6_01	Smad3	SMAD3	0.603115	9
V$SMAD4_Q6_01	Smad4	SMAD4	0.54774	9
V$IK_Q5	Ikaros	IKZF1	0.413263	8
V$CDX2_Q5_01	Cdx-2	CDX2	0.574546	8
V$CDX2_Q5_02	CDX-2	CDX2	0.574546	8
V$ETS2_B	c-Ets-2	ETS2	0.608624	8
V$CDX2_01	Cdx-2	CDX2	0.574546	8
V$DLX5_01	dlx5	DLX5	0.152369	8
V$GKLF_Q4	GKLF	KLF4	0.163624	8
V$AP4_Q6_02	AP-4	TFAP4	0.452837	8
V$IPF1_Q4_01	IPF1	PDX1	0.561481	8
V$SP1_01	Sp1	SP1	0.476443	8
V$MEF2C_Q4	MEF-2C	MEF2C	0.0200641	8
V$HNF4A_Q6_01	HNF-4alpha	HNF4A	0.0355781	8
V$GATA1_01	GATA-1	GATA1	0.129136	8
V$NKX32_01	Nkx3-2	NKX3-2	0.474029	8

V$IPF1_Q6	IPF1	PDX1	0.561481	8
V$MYOD_Q6_01	MyoD	MYOD1	0.495647	7
V$E2A_Q6	E2A	TCF3	0.400686	7
V$TBX5_02	TBX5	TBX5	0.408121	7
V$SOX9_Q4	SOX9	SOX9	0.0399767	7
V$PBX1_04	Pbx1	PBX1	0.13064	7
V$AML2_01	AML2	RUNX3	0.234858	7
V$CREM_Q6	CREM	CREM	0.0940004	7
V$CMYB_Q5	c-Myb	MYB	0.0250382	7
V$ATF1_Q6_01	ATF-1	ATF1	0.418742	7
V$IRF4_Q6	IRF-4	IRF4	0.586713	7
V$NFAT4_Q3	NF-AT4	NFATC3	0.257489	7
V$HNF3B_Q6	HNF-3beta	FOXA2	0.248369	7
V$AHR_Q5	AhR	AHR	0.0480466	7
V$E12_Q6	E12	TCF3	0.400686	7
V$GR_Q6	GR	NR3C1	0.564865	7
V$MYB_Q6	c-Myb	MYB	0.0250382	7
V$NR1B2_Q6	NR1B2	RARB	0.476286	7
V$YY1_01	YY1	YY1	0.172724	7
V$GATA1_04	GATA-1	GATA1	0.129136	7
V$GATA1_02	GATA-1	GATA1	0.129136	7
V$AP2REP_01	AP-2rep	KLF12	0.450204	7
V$E47_02	E47	TCF3	0.400686	7
V$SOX9_B1	SOX9	SOX9	0.0399767	7
V$GATA2_02	GATA-2	GATA2	0.431424	7
V$GATA1_06	GATA-1	GATA1	0.129136	7
V$SOX5_01	SOX5	SOX5	0.147394	7
V$GATA1_05	GATA-1	GATA1	0.129136	7
V$ARNT_01	Arnt	ARNT	0.548269	6
V$FOXP3_01	FOXP3	FOXP3	0.535235	6
V$PARP_Q3	PARP	PARP1	0.127428	6
V$HBP1_Q2	hbp1	HBP1	0.523956	6
V$IPF1_01	IPF1	PDX1	0.561481	6
V$NFAT2_Q5	NF-AT2	NFATC1	0.56407	6
V$NANOG_02	Nanog	NANOG	0.695304	6
V$HIF1A_Q6	HIF-1alpha	HIF1A	0.132218	6
V$PBX1_Q3	Pbx1	PBX1	0.13064	6
V$ERBETA_Q5	ER-beta	ESR2	0.381705	6
V$TBX5_01	TBX5	TBX5	0.408121	6
V$YY1_Q6_03	YY1	YY1	0.172724	6
V$GFI1B_01	Gfi1b	GFI1B	0.409807	6
V$SRY_02	SRY	SRY	0.546578	6
V$FOXO3A_Q1	FOXO3A	FOXO3	0.633824	6
V$MYOD_01	MyoD	MYOD1	0.495647	5
V$AML1_Q4	AML1	RUNX1	0.469438	5
V$TEF1_Q6_03	TEF-1	TEAD1	0.616313	5

V$GFI1_Q6_01	Gfi1	GFI1	0.090633	5
V$TCF4_01	TCF-4	TCF7L2	0.189508	5
V$CRX_Q4_01	CRX	CRX	0.441681	5
V$AP4_Q5	AP-4	TFAP4	0.452837	5
V$PITX3_Q2	PITX3	PITX3	0.403807	5
V$AML1_01	AML1a	RUNX1	0.469438	5
V$KAISO_01	KAISO	ZBTB33	0.639948	5
V$GR_01	GR	NR3C1	0.564865	5
V$HNF3A_01	HNF3A	FOXA1	0.528814	5
V$GFI1_Q6	Gfi1	GFI1	0.090633	5
V$MSX1_01	Msx-1	MSX1	0.465542	5
V$HOXA9_01	hoxa9	HOXA9	0.616064	5
V$TCF4_Q5	TCF-4	TCF7L2	0.189508	5
V$AML1_Q6	AML1	RUNX1	0.469438	5
V$CMAF_01	c-Maf	MAF	0.258982	5
V$PAX8_01	Pax-8	PAX8	0.0585053	5
V$ELF5_01	ELF5	ELF5	0.370192	5
V$LRF_Q2	LRF	ZBTB7A	0.454155	4
V$FOXO4_02	FOXO4	FOXO4	0.0949138	4
V$PITX2_Q2	Pitx2	PITX2	0.661672	4
V$TTF1_Q5	TTF-1	NKX2-1	0.108288	4
V$MYOGENIN_Q6_01	myogenin	MYOG	0.544144	4
V$ZFX_01	Zfx	ZFX	0.709511	4
V$TEF1_Q6	TEF-1	TEAD1	0.616313	4
V$AP4_Q6	AP-4	TFAP4	0.452837	4
V$SMAD3_Q6	SMAD3	SMAD3	0.603115	4
V$FOXJ2_01	FOXJ2	FOXJ2	0.516915	4
V$TCF3_01	TCF-3	TCF7L1	0.403167	4
V$MATH1_Q2	MATH1	ATOH1	0.384249	3
V$PITX2_01	PITX2	PITX2	0.661672	3
V$HNF6_Q6	HNF6	ONECUT1	0.183798	3
V$CRX_Q4	Crx	CRX	0.441681	3
V$CRX_02	Crx	CRX	0.441681	3
V$NKX32_02	Nkx3-2	NKX3-2	0.474029	3
V$IPF1_02	IPF1	PDX1	0.561481	3
V$PITX1_01	Pitx1	PITX1	0.662468	3
V$RFX1_02	RFX1	RFX1	0.611434	2
V$BCL6_Q3_01	Bcl-6	BCL6	0.639601	2
V$AP4_01	AP-4	TFAP4	0.452837	2
V$MEF2A_Q6	mef2A	MEF2A	0.0245837	2
V$ERR1_Q3	ERR1	ESRRA	0.393265	2
V$IRF2_01	IRF-2	IRF2	0.0779576	2
V$AIRE_01	AIRE	AIRE	0.122894	2
V$SOX10_Q6	SOX10	SOX10	0.0945659	2
V$IRF1_01	IRF-1	IRF1	0.483882	1

----

hsa-mir-101-2

Matrix_id	transcription factor	Gene	PCC	Occurrence
V$Elf5_03	ELF5	ELF5	0.370192	9
V$BEN_01	BEN	GTF2IRD1	0.622735	9
V$PARP_Q4	PARP	PARP1	0.127428	9
V$SMAD4_Q6_01	Smad4	SMAD4	0.54774	9
V$PEA3_Q6	PEA3	ETV4	0.682685	9
V$PUR1_Q4	PUR1	PURA	0.393818	9
V$P300_01	p300	EP300	0.50372	9
V$SMAD3_Q6_01	Smad3	SMAD3	0.603115	9
V$IPF1_Q4	IPF1	PDX1	0.561481	8
V$MEF2C_Q4	MEF-2C	MEF2C	0.0200641	8
V$HNF4A_Q6_01	HNF-4alpha	HNF4A	0.0355781	8
V$YY1_Q6	YY1	YY1	0.172724	8
V$AP4_Q6_02	AP-4	TFAP4	0.452837	8
V$IPF1_Q4_01	IPF1	PDX1	0.561481	8
V$GABPA_Q4	GABP-alpha	GABPA	0.0553642	8
V$YY1_Q6_02	YY1	YY1	0.172724	8
V$GKLF_Q4	GKLF	KLF4	0.163624	8
V$IK_Q5	Ikaros	IKZF1	0.413263	8
V$NKX32_01	Nkx3-2	NKX3-2	0.474029	8
V$CDX2_Q5_01	Cdx-2	CDX2	0.574546	8
V$CDX2_01	Cdx-2	CDX2	0.574546	8
V$IPF1_Q6	IPF1	PDX1	0.561481	8
V$GATA1_01	GATA-1	GATA1	0.129136	8
V$CDX2_Q5_02	CDX-2	CDX2	0.574546	8
V$SP1_Q6	Sp1	SP1	0.476443	8
V$ELK1_02	Elk-1	ELK1	0.325443	8
V$SP1_01	Sp1	SP1	0.476443	8
V$TEL1_02	TEL1	ETV6	0.595503	8
V$TBX5_02	TBX5	TBX5	0.408121	7
V$GATA1_04	GATA-1	GATA1	0.129136	7
V$GATA1_02	GATA-1	GATA1	0.129136	7
V$ETV7_01	ETV7	ETV7	0.581358	7
V$CMYB_Q5	c-Myb	MYB	0.0250382	7
V$ELK1_06	ELK-1	ELK1	0.325443	7
V$CREM_Q6	CREM	CREM	0.0940004	7
V$ESE1_Q3	ESE-1	ELF3	0.539295	7
V$SPI1_03	SPI1	SPI1	0.065947	7
V$YY1_01	YY1	YY1	0.172724	7
V$SOX9_Q4	SOX9	SOX9	0.0399767	7
V$MYB_Q6	c-Myb	MYB	0.0250382	7

V$ATF1_Q6_01	ATF-1	ATF1	0.418742	7
V$IRF4_Q6	IRF-4	IRF4	0.586713	7
V$GATA2_02	GATA-2	GATA2	0.431424	7
V$GATA1_06	GATA-1	GATA1	0.129136	7
V$EHF_03	EHF	EHF	0.529766	7
V$AP2REP_01	AP-2rep	KLF12	0.450204	7
V$GATA1_05	GATA-1	GATA1	0.129136	7
V$ER71_02	ER71	ETV2	0.267502	7
V$ETV3_02	ETV3	ETV3	0.163675	7
V$NFAT4_Q3	NF-AT4	NFATC3	0.257489	7
V$OC2_Q3	OC-2	ONECUT2	0.336029	7
V$AHR_Q5	AhR	AHR	0.0480466	7
V$PBX1_04	Pbx1	PBX1	0.13064	7
V$WT1_Q6_01	WT1	WT1	0.177647	6
V$ARNT_01	Arnt	ARNT	0.548269	6
V$PBX1_Q3	Pbx1	PBX1	0.13064	6
V$HIF1A_Q6	HIF-1alpha	HIF1A	0.132218	6
V$ESE1_02	ESE-1	ELF3	0.539295	6
V$ERG_03	ERG	ERG	0.550102	6
V$HBP1_Q2	hbp1	HBP1	0.523956	6
V$TBX5_01	TBX5	TBX5	0.408121	6
V$PARP_Q3	PARP	PARP1	0.127428	6
V$SP1_Q6_01	Sp1	SP1	0.476443	6
V$SP1_Q2_01	Sp1	SP1	0.476443	6
V$SP1_Q4_01	Sp1	SP1	0.476443	6
V$GATA2_01	GATA-2	GATA2	0.431424	6
V$NANOG_02	Nanog	NANOG	0.695304	6
V$YY1_02	YY1	YY1	0.172724	5
V$AML1_Q6	AML1	RUNX1	0.469438	5
V$RFX1_01	RFX1	RFX1	0.611434	5
V$PET1_02	Pet-1	FEV	0.507372	5
V$KAISO_01	KAISO	ZBTB33	0.639948	5
V$CP2_01	CP2	TFCP2	0.142877	5
V$TEF1_Q6_03	TEF-1	TEAD1	0.616313	5
V$PITX3_Q2	PITX3	PITX3	0.403807	5
V$SP1_02	SP1	SP1	0.476443	5
V$CRX_Q4_01	CRX	CRX	0.441681	5
V$HOXA9_01	hoxa9	HOXA9	0.616064	5
V$TCF3_01	TCF-3	TCF7L1	0.403167	4
V$PDEF_02	PDEF	SPDEF	0.290548	4
V$HNF1_02	HNF-1alpha	HNF1A	0.397431	4
V$TEF1_Q6	TEF-1	TEAD1	0.616313	4
V$ERG_01	ERG	ERG	0.550102	4
V$PITX2_Q2	Pitx2	PITX2	0.661672	4
V$E2F1_Q3	E2F-1	E2F1	0.148693	4
V$GATA2_03	GATA-2	GATA2	0.431424	4

V$STAF_02	Staf	ZNF143	0.607874	4
V$E2F1_Q3_01	E2F-1	E2F1	0.148693	4
V$SP4_Q5	SP4	SP4	0.628221	4
V$CNOT3_01	CNOT3	CNOT3	0.383166	4
V$AP2BETA_Q3	AP-2beta	TFAP2B	0.591809	4
V$LRF_Q2	LRF	ZBTB7A	0.454155	4
V$NURR1_Q3	NURR1	NR4A2	0.37923	4
V$GABPBETA_Q3	GABP-beta	GABPB1	0.436995	4
V$E2F1_Q6	E2F-1	E2F1	0.148693	3
V$CEBPB_02	C/EBPbeta	CEBPB	0.182821	3
V$AML2_Q3	AML2	RUNX3	0.234858	3
V$CRX_02	Crx	CRX	0.441681	3
V$ZBP89_Q4	ZBP89	ZNF148	0.61102	3
V$PITX2_01	PITX2	PITX2	0.661672	3
V$FAC1_01	FAC1	BPTF	0.275689	3
V$DBP_Q6_01	DBP	DBP	0.381441	3
V$CRX_Q4	Crx	CRX	0.441681	3
V$AML2_Q3_01	AML2	RUNX3	0.234858	3
V$CIZ_01	CIZ	ZNF384	0.23869	3
V$E2F1_Q6_01	E2F-1	E2F1	0.148693	3
V$PITX1_01	Pitx1	PITX1	0.662468	3
V$RFX1_02	RFX1	RFX1	0.611434	2
V$IRF1_Q6_01	IRF-1	IRF1	0.483882	2
V$HNF1B_01	HNF-1beta	HNF1B	0.3637	1
V$IRF1_Q6	IRF-1	IRF1	0.483882	1
V$WT1_Q6	WT1	WT1	0.177647	1
V$AHR_01	AhR	AHR	0.0480466	1

----

hsa-mir-103a-1

Matrix_id	transcription factor	Gene	PCC	Occurrence
V$MAFB_01	MAFB	MAFB	0.339311	3
V$ELF1_Q6	Elf-1	ELF1	0.0079201	3
V$AHR_Q5	AhR	AHR	0.352312	3
V$GATA6_01	GATA-6	GATA6	0.251442	3
V$SOX9_B1	SOX9	SOX9	0.0280758	3
V$GATA3_02	GATA-3	GATA3	0.416383	3
V$GATA2_02	GATA-2	GATA2	0.146062	3
V$DLX5_01	dlx5	DLX5	0.209698	3
V$TBP_Q6	TBP	TBP	0.107054	3
V$PARP_Q4	PARP	PARP1	0.208507	3
V$SPI1_03	SPI1	SPI1	0.0141189	3
V$HOX13_02	HOXA5	HOXA5	0.262718	2

V$POU6F1_03	POU6F1	POU6F1	0.102675	2
V$CEBPE_Q6	CEBPE	CEBPE	0.0731097	2
V$STAT5A_Q6	STAT5A	STAT5A	0.192008	2
V$CEBPB_Q6	C/EBPbeta	CEBPB	0.257561	2
V$MEF2C_Q4	MEF-2C	MEF2C	0.25504	2
V$NKX2B_Q3	NKX2B	NKX2-2	0.352958	2
V$PARP_Q3	PARP	PARP1	0.208507	2
V$GATA2_01	GATA-2	GATA2	0.146062	2
V$GATA3_01	GATA-3	GATA3	0.416383	2
V$CEBPB_02	C/EBPbeta	CEBPB	0.257561	2
V$CEBPB_01	C/EBPbeta	CEBPB	0.257561	1
V$MEF2A_Q6	mef2A	MEF2A	0.119907	1
V$POU6F1_01	POU6F1	POU6F1	0.102675	1
V$CEBPG_Q6_01	C/EBPgamma	CEBPG	0.298742	1
V$FOXO4_01	FOXO4	FOXO4	0.414082	1
V$SATB1_Q3	SATB1	SATB1	0.0758978	1
V$HMGIY_01	HMGIY	HMGA1	0.102285	1
V$FOXO4_02	FOXO4	FOXO4	0.414082	1
V$POU6F1_02	POU6F1	POU6F1	0.102675	1
V$CEBPD_Q6	C/EBPdelta	CEBPD	0.158856	1
V$PIT1_Q6	Pit-1	POU1F1	0.06035	1
V$NKX22_02	NKX2B	NKX2-2	0.352958	1
V$CDP_04	CDP	CUX1	0.177681	1
V$YY1_01	YY1	YY1	0.162605	1

----

hsa-mir-103a-2

Matrix_id	transcription factor	Gene	PCC	Occurrence
V$ELK1_02	Elk-1	ELK1	0.0703832	3
V$SOX9_Q4	SOX9	SOX9	0.0280758	3
V$SPI1_03	SPI1	SPI1	0.0141189	3
V$DLX5_01	dlx5	DLX5	0.209698	3
V$GABPA_Q4	GABP-alpha	GABPA	0.00877552	3
V$IRF8_Q6	IRF-8	IRF8	0.25653	3
V$PUR1_Q4	PUR1	PURA	0.144841	3
V$GKLF_Q4	GKLF	KLF4	0.189779	3
V$IRF7_Q3	IRF-7	IRF7	0.0451349	3
V$PARP_Q4	PARP	PARP1	0.208507	3
V$ELK1_06	ELK-1	ELK1	0.0703832	3
V$MAFB_01	MAFB	MAFB	0.339311	3
V$ELF1_Q6	Elf-1	ELF1	0.0079201	3
V$TBP_Q6	TBP	TBP	0.107054	3
V$SOX9_B1	SOX9	SOX9	0.0280758	3

V$TFIII_Q6	TFII-I	GTF2I	0.085451	3
V$AHR_Q5	AhR	AHR	0.352312	3
V$CEBPB_02	C/EBPbeta	CEBPB	0.257561	2
V$CEBPB_Q6	C/EBPbeta	CEBPB	0.257561	2
V$PARP_Q3	PARP	PARP1	0.208507	2
V$HIF1A_Q6	HIF-1alpha	HIF1A	0.0294576	2
V$MEF2C_Q4	MEF-2C	MEF2C	0.25504	2
V$SPI1_Q5	SPI1	SPI1	0.0141189	2
V$AP2ALPHA_Q6	AP-2alpha	TFAP2A	0.3199	2
V$YY1_Q6	YY1	YY1	0.162605	2
V$ING4_01	ING4	ING4	0.558481	2
V$HOX13_02	HOXA5	HOXA5	0.262718	2
V$ZABC1_01	ZABC1	ZNF217	0.176485	2
V$NURR1_Q3	NURR1	NR4A2	0.0300761	2
V$ERM_02	Erm	ETV5	0.51048	1
V$YY1_01	YY1	YY1	0.162605	1
V$CP2_01	CP2	TFCP2	0.414045	1
V$EGR2_01	Egr-2	EGR2	0.0647265	1
V$CEBPD_Q6	C/EBPdelta	CEBPD	0.158856	1
V$CDP_04	CDP	CUX1	0.177681	1

hsa-mir-106a

Matrix_id	transcription factor	Gene	PCC	Occurrence
V$ETS1_B	c-Ets-1	ETS1	0.155974	19
V$ELF1_Q6	Elf-1	ELF1	0.558313	19
V$PARP_Q4	PARP	PARP1	0.0141356	19
V$NFAT4_Q3	NF-AT4	NFATC3	0.595036	19
V$IK_Q5	Ikaros	IKZF1	0.207224	18
V$TBP_Q6	TBP	TBP	0.0966152	17
V$CETS1_Q6	C-ets-1	ETS1	0.155974	17
V$GABPA_Q4	GABP-alpha	GABPA	0.00840813	16
V$DLX5_01	dlx5	DLX5	0.163376	15
V$YY1_01	YY1	YY1	0.164824	15
V$IRF8_Q6	IRF-8	IRF8	0.165231	14
V$AHR_Q5	AhR	AHR	0.207425	14
V$E2A_Q6	E2A	TCF3	0.074395	13
V$HMGIY_01	HMGIY	HMGA1	0.708875	13
V$E12_Q6	E12	TCF3	0.074395	13
V$GATA3_01	GATA-3	GATA3	0.496512	13
V$E47_02	E47	TCF3	0.074395	13
V$YY1_Q6_02	YY1	YY1	0.164824	12
V$GFI1_Q6	Gfi1	GFI1	0.58291	12

V$YY1_Q6_03	YY1	YY1	0.164824	11
V$CMYB_01	c-Myb	MYB	0.651373	11
V$E2A_Q2	E2A	TCF3	0.074395	11
V$YY1_Q6	YY1	YY1	0.164824	11
V$ING4_01	ING4	ING4	0.237336	10
V$FOXO4_02	FOXO4	FOXO4	0.169378	10
V$GFI1_Q6_01	Gfi1	GFI1	0.58291	9
V$AML2_Q3	AML2	RUNX3	0.0768146	9
V$CEBPA_01	C/EBPalpha	CEBPA	0.135586	9
V$GATA3_03	GATA-3	GATA3	0.496512	7
V$IRF7_Q3	IRF-7	IRF7	0.234445	7
V$STAT1_05	STAT1	STAT1	0.147706	7
V$ZABC1_01	ZABC1	ZNF217	0.444808	5
V$YY1_03	YY1	YY1	0.164824	4
V$CP2_01	CP2	TFCP2	0.11434	3
V$AHR_01	AhR	AHR	0.207425	2
V$STAT1_Q6	STAT1	STAT1	0.147706	2

hsa-mir-107

Matrix_id	transcription factor	Gene	PCC	Occurrence
V$PUR1_Q4	PUR1	PURA	0.114982	37
V$MAFB_01	MAFB	MAFB	0.321674	35
V$P300_01	p300	EP300	0.0443999	34
V$PARP_Q4	PARP	PARP1	0.179995	34
V$ELF1_Q6	Elf-1	ELF1	0.0532784	33
V$AP2REP_01	AP-2rep	KLF12	0.0668963	32
V$DLX5_01	dlx5	DLX5	0.0791999	31
V$SMAD3_Q6_01	Smad3	SMAD3	0.058733	31
V$HNF4A_Q6_01	HNF-4alpha	HNF4A	0.755797	30
V$Elf5_03	ELF5	ELF5	0.127355	30
V$ETS1_B	c-Ets-1	ETS1	0.50589	28
V$CDX2_Q5_02	CDX-2	CDX2	0.121061	28
V$TBP_Q6	TBP	TBP	0.085369	28
V$GABPA_Q4	GABP-alpha	GABPA	0.419672	27
V$SOX5_01	SOX5	SOX5	0.252095	27
V$CETS1_Q6	C-ets-1	ETS1	0.50589	27
V$MEF2C_Q4	MEF-2C	MEF2C	0.154708	26
V$AP4_Q6_02	AP-4	TFAP4	0.192697	26
V$TBX5_02	TBX5	TBX5	0.221053	25
V$CDX2_01	Cdx-2	CDX2	0.121061	24
V$PARP_Q3	PARP	PARP1	0.179995	24
V$SPI1_Q5	SPI1	SPI1	0.116644	24

V$IPF1_Q4_01	IPF1	PDX1	0.110608	23
V$CDX2_Q5_01	Cdx-2	CDX2	0.121061	23
V$IPF1_Q6	IPF1	PDX1	0.110608	23
V$SPI1_03	SPI1	SPI1	0.116644	23
V$OC2_Q3	OC-2	ONECUT2	0.393874	22
V$CEBPE_Q6	CEBPE	CEBPE	0.519654	22
V$TCF3_01	TCF-3	TCF7L1	0.377132	22
V$AML1_Q6	AML1	RUNX1	0.0555668	22
V$GATA1_06	GATA-1	GATA1	0.215598	21
V$GATA6_01	GATA-6	GATA6	0.113392	21
V$GATA1_05	GATA-1	GATA1	0.215598	21
V$HNF3B_Q6	HNF-3beta	FOXA2	0.184636	20
V$LRF_Q2	LRF	ZBTB7A	0.315163	20
V$TBX5_01	TBX5	TBX5	0.221053	19
V$GATA3_02	GATA-3	GATA3	0.0871137	19
V$IRF4_Q6	IRF-4	IRF4	0.136451	19
V$GATA1_02	GATA-1	GATA1	0.215598	19
V$IPF1_Q4	IPF1	PDX1	0.110608	18
V$GATA1_04	GATA-1	GATA1	0.215598	17
V$TEF1_Q6	TEF-1	TEAD1	0.0676334	17
V$GFI1_Q6_01	Gfi1	GFI1	0.117638	17
V$FOXJ2_01	FOXJ2	FOXJ2	0.0151085	16
V$IRF7_Q3	IRF-7	IRF7	0.0225962	16
V$SOX10_Q6	SOX10	SOX10	0.230931	16
V$TEF1_Q6_03	TEF-1	TEAD1	0.0676334	16
V$GFI1_Q6	Gfi1	GFI1	0.117638	16
V$CMAF_01	c-Maf	MAF	0.419311	16
V$HNF6_Q6	HNF6	ONECUT1	0.340872	15
V$CEBPB_Q6	C/EBPbeta	CEBPB	0.045712	15
V$CREM_Q6	CREM	CREM	0.0494795	14
V$CEBPA_01	C/EBPalpha	CEBPA	0.450553	14
V$CEBPA_Q6	C/EBPalpha	CEBPA	0.450553	14
V$HOXA9_01	hoxa9	HOXA9	0.162973	14
V$FOXP3_01	FOXP3	FOXP3	0.132803	14
V$AML1_01	AML1a	RUNX1	0.0555668	13
V$GFI1B_01	Gfi1b	GFI1B	0.114454	13
V$CDX2_Q5	Cdx-2	CDX2	0.121061	13
V$CDX1_01	Cdx-1	CDX1	0.325199	13
V$FOXO4_02	FOXO4	FOXO4	0.0990171	13
V$AML1_Q4	AML1	RUNX1	0.0555668	12
V$GATA3_03	GATA-3	GATA3	0.0871137	12
V$CEBPB_01	C/EBPbeta	CEBPB	0.045712	10
V$ELF5_01	ELF5	ELF5	0.127355	10
V$EAR2_Q2	EAR2	NR2F6	0.466664	8
V$SOX2_Q6	SOX2	SOX2	0.502482	7
V$ELK1_01	Elk-1	ELK1	0.325609	6

V$FOXJ2_02	FOXJ2	FOXJ2	0.0151085	6
V$SPIB_03	Spi-B	SPIB	0.145739	6
V$POU6F1_03	POU6F1	POU6F1	0.543765	5
V$SPIB_01	SPI-B	SPIB	0.145739	5
V$PIT1_Q6	Pit-1	POU1F1	0.680037	5
V$FOXO4_01	FOXO4	FOXO4	0.0990171	4
V$OCT2_01	2-Oct	POU2F2	0.402634	3
V$STAT5A_Q6	STAT5A	STAT5A	0.302946	2

---

hsa-mir-10a

Matrix_id	transcription factor	Gene	PCC	Occurrence
V$IRF4_Q6	IRF-4	IRF4	0.0354482	7
V$YY1_Q6	YY1	YY1	0.100086	7
V$GR_01	GR	NR3C1	0.0910587	7
V$GR_Q6	GR	NR3C1	0.0910587	7
V$YY1_Q6_02	YY1	YY1	0.100086	7
V$YY1_Q6_03	YY1	YY1	0.100086	6
V$HNF3A_01	HNF3A	FOXA1	0.0426083	6
V$HNF1_02	HNF-1alpha	HNF1A	0.0038385	6
V$DLX5_01	dlx5	DLX5	0.0150466	6
V$SMAD4_Q6_01	Smad4	SMAD4	0.00922339	6
V$AP4_Q6_02	AP-4	TFAP4	0.106593	6
V$ELF1_Q6	Elf-1	ELF1	0.293935	6
V$SOX5_01	SOX5	SOX5	0.0477576	6
V$YY1_01	YY1	YY1	0.100086	6
V$SOX9_B1	SOX9	SOX9	0.137998	6
V$SOX9_Q4	SOX9	SOX9	0.137998	5
V$HIF1A_Q6	HIF-1alpha	HIF1A	0.436819	5
V$YY1_02	YY1	YY1	0.100086	5
V$GATA6_01	GATA-6	GATA6	0.0875485	5
V$HOXD9_Q2	Hoxd9	HOXD9	0.171452	5
V$HNF4A_Q6_01	HNF-4alpha	HNF4A	0.465915	5
V$TCF4_Q5	TCF-4	TCF7L2	0.104259	5
V$HOXA9_01	hoxa9	HOXA9	0.447263	5
V$HOX13_02	HOXA5	HOXA5	0.373906	4
V$FOXP3_01	FOXP3	FOXP3	0.0230651	4
V$AR_03	AR	AR	0.00418553	4
V$PITX3_Q2	PITX3	PITX3	0.139699	4
V$YY1_03	YY1	YY1	0.100086	4
V$CMAF_01	c-Maf	MAF	0.571389	4
V$AR_02	AR	AR	0.00418553	4
V$PIT1_Q6	Pit-1	POU1F1	0.237864	3

V$TCF3_01	TCF-3	TCF7L1	0.167114	3
V$CRX_Q4_01	CRX	CRX	0.126537	3
V$AR_Q2	AR	AR	0.00418553	3
V$CART1_02	CART1	ALX1	0.0977328	3
V$FOXJ2_01	FOXJ2	FOXJ2	0.0460081	3
V$E2F1_Q4	E2F-1	E2F1	0.174805	2
V$STAT3_03	STAT3	STAT3	0.342888	2
V$MITF_Q6	MITF	MITF	0.262291	2
V$POU6F1_01	POU6F1	POU6F1	0.0820239	2
V$AR_01	AR	AR	0.00418553	1
V$AR_04	AR	AR	0.00418553	1
V$CDP_01	CDP	CUX1	0.110613	1
V$CDP_04	CDP	CUX1	0.110613	1
V$OCT2_01	2-Oct	POU2F2	0.208946	1
V$HNF6_Q6	HNF6	ONECUT1	0.296772	1

----

hsa-mir-10b

Matrix_id	transcription factor	Gene	PCC	Occurrence
V$GR_Q6	GR	NR3C1	0.105942	2
V$TCF4_01	TCF-4	TCF7L2	0.232012	2
V$PUR1_Q4	PUR1	PURA	0.0460455	2
V$ZBP89_Q4	ZBP89	ZNF148	0.0751403	2
V$WT1_Q6	WT1	WT1	0.801625	2
V$FKLF_Q5	FKLF	KLF11	0.091198	2
V$SMAD4_Q6_01	Smad4	SMAD4	0.121975	2
V$E2F1_Q3_01	E2F-1	E2F1	0.0573181	2
V$SP3_Q3	Sp3	SP3	0.157676	2
V$EGR1_02	EGR-1	EGR1	0.177964	2
V$ER71_02	ER71	ETV2	0.0463571	2
V$E2F1_Q3	E2F-1	E2F1	0.0573181	2
V$WT1_Q6_01	WT1	WT1	0.801625	2
V$E2F1_Q6	E2F-1	E2F1	0.0573181	2
V$ZIC3_01	Zic3	ZIC3	0.243079	2
V$DLX5_01	dlx5	DLX5	0.0422554	2
V$HOXD9_Q2	Hoxd9	HOXD9	0.292917	1
V$HIF1A_Q6	HIF-1alpha	HIF1A	0.370632	1
V$HBP1_Q2	hbp1	HBP1	0.0562159	1
V$PBX1_04	Pbx1	PBX1	0.663488	1
V$MSX1_01	Msx-1	MSX1	0.396623	1
V$E2F1_Q4	E2F-1	E2F1	0.0573181	1
V$FAC1_01	FAC1	BPTF	0.298454	1
V$AR_01	AR	AR	0.0287441	1

V$E2F1_Q6_01	E2F-1	E2F1	0.0573181	1
V$GR_01	GR	NR3C1	0.105942	1
V$TBP_Q6	TBP	TBP	0.117195	1
V$AR_04	AR	AR	0.0287441	1
V$CDP_04	CDP	CUX1	0.207064	1
V$HOXA9_01	hoxa9	HOXA9	0.364197	1
V$MITF_Q6	MITF	MITF	0.580163	1

----

hsa-mir-1-1

Matrix_id	transcription factor	Gene	PCC	Occurrence
V$PUR1_Q4	PUR1	PURA	0.284904	871
V$IK_Q5	Ikaros	IKZF1	0.636806	859
V$PEA3_Q6	PEA3	ETV4	0.645544	827
V$AP2REP_01	AP-2rep	KLF12	0.820084	822
V$P300_01	p300	EP300	0.685502	806
V$SMAD4_Q6_01	Smad4	SMAD4	0.660973	757
V$ZIC3_01	Zic3	ZIC3	0.442801	755
V$CMYB_Q5	c-Myb	MYB	0.227328	733
V$MYB_Q6	c-Myb	MYB	0.227328	733
V$NR1B2_Q6	NR1B2	RARB	0.724407	705
V$NFAT4_Q3	NF-AT4	NFATC3	0.429876	699
V$TBX5_02	TBX5	TBX5	0.865621	696
V$CETS1_Q6	C-ets-1	ETS1	0.626654	694
V$GATA1_01	GATA-1	GATA1	0.733512	688
V$TTF1_Q5	TTF-1	NKX2-1	0.388528	685
V$ETS2_Q6	c-Ets-2	ETS2	0.53543	675
V$SMAD3_Q6_01	Smad3	SMAD3	0.69902	665
V$YY1_01	YY1	YY1	0.289722	659
V$GABPA_Q4	GABP-alpha	GABPA	0.369465	658
V$Elf5_03	ELF5	ELF5	0.130899	653
V$MEF2C_Q4	MEF-2C	MEF2C	0.344095	653
V$DLX5_01	dlx5	DLX5	0.00406789	640
V$GR_Q6	GR	NR3C1	0.556708	639
V$IPF1_01	IPF1	PDX1	0.86259	628
V$AML1_Q6	AML1	RUNX1	0.735806	626
V$NANOG_02	Nanog	NANOG	0.787963	625
V$AP4_Q6_02	AP-4	TFAP4	0.56931	604
V$HNF4A_Q6_01	HNF-4alpha	HNF4A	0.272079	599
V$TBX5_01	TBX5	TBX5	0.865621	575
V$MAZ_Q6	MAZ	MAZ	0.328774	551
V$LRF_Q2	LRF	ZBTB7A	0.824246	541
V$BEN_01	BEN	GTF2IRD1	0.823154	541

V$ERBETA_Q5	ER-beta	ESR2	0.601198	537
V$SP1_Q6	Sp1	SP1	0.837829	509
V$E2A_Q6	E2A	TCF3	0.704103	505
V$E12_Q6	E12	TCF3	0.704103	505
V$TEL1_02	TEL1	ETV6	0.804904	504
V$ELK1_02	Elk-1	ELK1	0.645852	503
V$E47_02	E47	TCF3	0.704103	502
V$MYOD_Q6_01	MyoD	MYOD1	0.693623	501
V$ETV3_02	ETV3	ETV3	0.584393	489
V$ING4_01	ING4	ING4	0.0867976	487
V$ZBP89_Q4	ZBP89	ZNF148	0.669612	485
V$MYOGENIN_Q6	myogenin	MYOG	0.666344	481
V$ER71_02	ER71	ETV2	0.601489	480
V$AP2ALPHA_Q6	AP-2alpha	TFAP2A	0.0823676	459
V$ELK1_06	ELK-1	ELK1	0.645852	457
V$SP1_Q6_01	Sp1	SP1	0.837829	452
V$TCF3_01	TCF-3	TCF7L1	0.65737	448
V$SP1_01	Sp1	SP1	0.837829	446
V$SP1_Q4_01	Sp1	SP1	0.837829	437
V$TEF1_Q6_03	TEF-1	TEAD1	0.805734	433
V$AP4_Q5	AP-4	TFAP4	0.56931	430
V$ETV7_01	ETV7	ETV7	0.790083	429
V$AP2ALPHA_01	AP-2alpha	TFAP2A	0.0823676	429
V$FKLF_Q5	FKLF	KLF11	0.17278	427
V$SP1_Q2_01	Sp1	SP1	0.837829	426
V$WT1_Q6_01	WT1	WT1	0.0446018	422
V$TFIII_Q6	TFII-I	GTF2I	0.667357	406
V$EHF_03	EHF	EHF	0.139131	404
V$ERF_02	ERF	ERF	0.549768	403
V$E2A_Q2	E2A	TCF3	0.704103	402
V$CMYB_01	c-Myb	MYB	0.227328	401
V$SP1_02	SP1	SP1	0.837829	396
V$AP4_Q6	AP-4	TFAP4	0.56931	385
V$TEF1_Q6	TEF-1	TEAD1	0.805734	379
V$AML1_01	AML1a	RUNX1	0.735806	371
V$ZFX_01	Zfx	ZFX	0.745319	370
V$ERR1_Q3	ERR1	ESRRA	0.811703	358
V$MYOGENIN_Q6_01	myogenin	MYOG	0.666344	355
V$AML1_Q4	AML1	RUNX1	0.735806	338
V$CREM_Q6	CREM	CREM	0.284686	333
V$ZIC1_01	Zic1	ZIC1	0.154819	326
V$NEUROD_02	NeuroD	NEUROD1	0.722997	317
V$SMAD3_Q6	SMAD3	SMAD3	0.69902	314
V$MYOD_Q6	MyoD	MYOD1	0.693623	309
V$AP2GAMMA_01	AP-2gamma	TFAP2C	0.191264	305
V$AP2BETA_Q3	AP-2beta	TFAP2B	0.732459	302

V$DAX1_01	Dax1	NR0B1	0.549336	302
V$PAX8_01	Pax-8	PAX8	0.460136	294
V$MEF2A_Q6	mef2A	MEF2A	0.0937993	292
V$GABPBETA_Q3	GABP-beta	GABPB1	0.593327	292
V$CNOT3_01	CNOT3	CNOT3	0.721805	281
V$MYOD_Q6_02	MyoD	MYOD1	0.693623	274
V$VDR_Q3	VDR	VDR	0.747682	271
V$ERG_03	ERG	ERG	0.689524	271
V$PET1_02	Pet-1	FEV	0.509968	270
V$DBP_Q6_01	DBP	DBP	0.797486	269
V$MATH1_Q2	MATH1	ATOH1	0.593323	256
V$KAISO_01	KAISO	ZBTB33	0.862789	252
V$ATF1_Q6_01	ATF-1	ATF1	0.527509	249
V$MYOD_01	MyoD	MYOD1	0.693623	243
V$ATF3_Q6_01	ATF-3	ATF3	0.157726	219
V$ERG_01	ERG	ERG	0.689524	205
V$CIZ_01	CIZ	ZNF384	0.466011	204
V$P53_02	p53	TP53	0.313002	186
V$BEN_02	BEN	GTF2IRD1	0.823154	149
V$STAF_02	Staf	ZNF143	0.731546	140
V$SMAD4_Q6	SMAD4	SMAD4	0.660973	135
V$EGR2_01	Egr-2	EGR2	0.230855	129
V$GLI_Q2	GLI	GLI1	0.68613	117
V$NUR77_Q5	NUR77	NR4A1	0.347567	116
V$ERR3_Q2	ERR3	ESRRG	0.585696	108
V$GLI3_Q5_01	GLI3	GLI3	0.634096	94
V$ATF6_01	ATF6	ATF6	0.294647	91
V$AMEF2_Q6	aMEF-2	MEF2A	0.0937993	91
V$GLI2_01	GLI2	GLI2	0.768536	76
V$RSRFC4_Q2	RSRFC4	MEF2A	0.0937993	76
V$ERR3_Q2_01	ERR3	ESRRG	0.585696	72
V$GLI3_02	GLI3	GLI3	0.634096	70
V$GCNF_Q3	GCNF	NR6A1	0.446207	69
V$RSRFC4_01	RSRFC4	MEF2A	0.0937993	61
V$GLI3_01	GLI3	GLI3	0.634096	59
V$HLF_01	HLF	HLF	0.0846727	42
V$E4BP4_01	E4BP4	NFIL3	0.572627	37
V$MEF2A_05	MEF2A	MEF2A	0.0937993	17

----

hsa-mir-1-2

Matrix_id	transcription factor	Gene	PCC	Occurrence
V$IK_Q5	Ikaros	IKZF1	0.636806	859

V$PARP_Q4	PARP	PARP1	0.509037	828
V$PEA3_Q6	PEA3	ETV4	0.645544	827
V$AP2REP_01	AP-2rep	KLF12	0.820084	822
V$P300_01	p300	EP300	0.685502	806
V$SMAD4_Q6_01	Smad4	SMAD4	0.660973	757
V$ZIC3_01	Zic3	ZIC3	0.442801	755
V$CMYB_Q5	c-Myb	MYB	0.227328	733
V$MYB_Q6	c-Myb	MYB	0.227328	733
V$CDX2_Q5_02	CDX-2	CDX2	0.741281	718
V$TBP_Q6	TBP	TBP	0.258549	718
V$NR1B2_Q6	NR1B2	RARB	0.724407	705
V$NFAT4_Q3	NF-AT4	NFATC3	0.429876	699
V$TBX5_02	TBX5	TBX5	0.865621	696
V$CETS1_Q6	C-ets-1	ETS1	0.626654	694
V$GATA1_01	GATA-1	GATA1	0.733512	688
V$ETS1_B	c-Ets-1	ETS1	0.626654	687
V$TTF1_Q5	TTF-1	NKX2-1	0.388528	685
V$ETS2_Q6	c-Ets-2	ETS2	0.53543	675
V$SMAD3_Q6_01	Smad3	SMAD3	0.69902	665
V$YY1_01	YY1	YY1	0.289722	659
V$GABPA_Q4	GABP-alpha	GABPA	0.369465	658
V$Elf5_03	ELF5	ELF5	0.130899	653
V$MEF2C_Q4	MEF-2C	MEF2C	0.344095	653
V$DLX5_01	dlx5	DLX5	0.00406789	640
V$GR_Q6	GR	NR3C1	0.556708	639
V$IPF1_01	IPF1	PDX1	0.86259	628
V$AML1_Q6	AML1	RUNX1	0.735806	626
V$NANOG_02	Nanog	NANOG	0.787963	625
V$YY1_Q6	YY1	YY1	0.289722	623
V$SOX5_01	SOX5	SOX5	0.483194	618
V$AP4_Q6_02	AP-4	TFAP4	0.56931	604
V$HNF4A_Q6_01	HNF-4alpha	HNF4A	0.272079	599
V$GATA1_02	GATA-1	GATA1	0.733512	599
V$ETS2_B	c-Ets-2	ETS2	0.53543	587
V$GATA2_02	GATA-2	GATA2	0.298638	580
V$GATA6_01	GATA-6	GATA6	0.0850993	580
V$GATA1_06	GATA-1	GATA1	0.733512	580
V$GATA1_05	GATA-1	GATA1	0.733512	580
V$TBX5_01	TBX5	TBX5	0.865621	575
V$SPI1_Q5	SPI1	SPI1	0.261517	571
V$IPF1_Q6	IPF1	PDX1	0.86259	571
V$CDX2_Q5_01	Cdx-2	CDX2	0.741281	558
V$SRY_02	SRY	SRY	0.541791	551
V$MAZ_Q6	MAZ	MAZ	0.328774	551
V$IPF1_Q4_01	IPF1	PDX1	0.86259	547
V$LRF_Q2	LRF	ZBTB7A	0.824246	541

V$CDX2_01	Cdx-2	CDX2	0.741281	539
V$FOXO3A_Q1	FOXO3A	FOXO3	0.423087	528
V$PARP_Q3	PARP	PARP1	0.509037	521
V$NFAT2_Q5	NF-AT2	NFATC1	0.846281	519
V$HNF3B_Q6	HNF-3beta	FOXA2	0.431652	514
V$IRF4_Q6	IRF-4	IRF4	0.804591	511
V$E2A_Q6	E2A	TCF3	0.704103	505
V$E12_Q6	E12	TCF3	0.704103	505
V$TEL1_02	TEL1	ETV6	0.804904	504
V$ELK1_02	Elk-1	ELK1	0.645852	503
V$E47_02	E47	TCF3	0.704103	502
V$MYOD_Q6_01	MyoD	MYOD1	0.693623	501
V$GATA1_04	GATA-1	GATA1	0.733512	500
V$ETV3_02	ETV3	ETV3	0.584393	489
V$ING4_01	ING4	ING4	0.0867976	487
V$ZBP89_Q4	ZBP89	ZNF148	0.669612	485
V$ER71_02	ER71	ETV2	0.601489	480
V$HNF3A_01	HNF3A	FOXA1	0.373011	478
V$ELK1_06	ELK-1	ELK1	0.645852	457
V$IPF1_Q4	IPF1	PDX1	0.86259	456
V$TCF3_01	TCF-3	TCF7L1	0.65737	448
V$ARNT_01	Arnt	ARNT	0.759719	438
V$HNF1_02	HNF-1alpha	HNF1A	0.738035	433
V$AP4_Q5	AP-4	TFAP4	0.56931	430
V$GFI1_Q6	Gfi1	GFI1	0.341059	429
V$RFX1_02	RFX1	RFX1	0.807434	419
V$FOXJ2_01	FOXJ2	FOXJ2	0.643938	419
V$GFI1_Q6_01	Gfi1	GFI1	0.341059	415
V$FOXP3_01	FOXP3	FOXP3	0.736199	415
V$CEBPE_Q6	CEBPE	CEBPE	0.560226	412
V$GATA2_01	GATA-2	GATA2	0.298638	409
V$SOX10_Q6	SOX10	SOX10	0.341071	408
V$FOXM1_01	FOXM1	FOXM1	0.157659	408
V$ERF_02	ERF	ERF	0.549768	403
V$CMYB_01	c-Myb	MYB	0.227328	401
V$OC2_Q3	OC-2	ONECUT2	0.805198	382
V$AML1_01	AML1a	RUNX1	0.735806	371
V$RFX1_01	RFX1	RFX1	0.807434	369
V$GFI1B_01	Gfi1b	GFI1B	0.886126	346
V$AML1_Q4	AML1	RUNX1	0.735806	338
V$CEBPB_02	C/EBPbeta	CEBPB	0.177725	325
V$HOXA9_01	hoxa9	HOXA9	0.426344	315
V$NKX2B_Q3	NKX2B	NKX2-2	0.186511	314
V$CEBPD_Q6	C/EBPdelta	CEBPD	0.16415	312
V$BCL6_Q3_01	Bcl-6	BCL6	0.769567	308
V$E47_01	E47	TCF3	0.704103	307

V$FAC1_01	FAC1	BPTF	0.200283	307
V$CDX1_01	Cdx-1	CDX1	0.507112	301
V$NKX22_02	NKX2B	NKX2-2	0.186511	300
V$PAX8_01	Pax-8	PAX8	0.460136	294
V$LHX3b_01	LHX3b	LHX3	0.809368	289
V$MSX1_01	Msx-1	MSX1	0.478472	280
V$CEBPB_Q6	C/EBPbeta	CEBPB	0.177725	267
V$AML2_01	AML2	RUNX3	0.498381	258
V$NCX_02	Ncx	TLX2	0.815303	247
V$CART1_02	CART1	ALX1	0.728961	223
V$CDX2_Q5	Cdx-2	CDX2	0.741281	222
V$IPF1_06	ipf1	PDX1	0.86259	204
V$HOXB8_01	HOXB8	HOXB8	0.447908	203
V$IPF1_03	IPF1	PDX1	0.86259	192
V$LHX3_01	Lhx3	LHX3	0.809368	147
V$PAX3_B	Pax-3	PAX3	0.753377	140
V$CEBPB_01	C/EBPbeta	CEBPB	0.177725	139
V$HNF1B_01	HNF-1beta	HNF1B	0.345026	122
V$RSRFC4_Q2	RSRFC4	MEF2A	0.0937993	76
V$HOXA7_01	HOXA7	HOXA7	0.257072	70
V$RSRFC4_01	RSRFC4	MEF2A	0.0937993	61
V$LHX3A_01	Lhx3a	LHX3	0.809368	29
V$AFP1_Q6	AFP1	ZFHX3	0.326333	24

hsa-mir-122

Matrix_id	transcription factor	Gene	PCC	Occurrence
V$ELF1_Q6	Elf-1	ELF1	0.192827	242
V$MAFB_01	MAFB	MAFB	0.383476	239
V$TBX5_02	TBX5	TBX5	0.132419	227
V$NR1B2_Q6	NR1B2	RARB	0.117271	226
V$CDX2_Q5_02	CDX-2	CDX2	0.105817	215
V$GATA1_01	GATA-1	GATA1	0.0128229	215
V$SMAD3_Q6_01	Smad3	SMAD3	0.0915205	203
V$HNF4A_Q6_01	HNF-4alpha	HNF4A	0.316168	191
V$SOX5_01	SOX5	SOX5	0.149287	185
V$SPI1_03	SPI1	SPI1	0.268782	178
V$ERBETA_Q5	ER-beta	ESR2	0.303769	176
V$GATA1_02	GATA-1	GATA1	0.0128229	173
V$GATA1_05	GATA-1	GATA1	0.0128229	168
V$GATA1_06	GATA-1	GATA1	0.0128229	168
V$CDX2_Q5_01	Cdx-2	CDX2	0.105817	167
V$MAZ_Q6	MAZ	MAZ	0.414832	166

V$MYOD_Q6_01	MyoD	MYOD1	0.306424	164
V$PITX3_Q2	PITX3	PITX3	0.127413	160
V$ING4_01	ING4	ING4	0.234372	158
V$HNF3B_Q6	HNF-3beta	FOXA2	0.44036	154
V$CDX2_01	Cdx-2	CDX2	0.105817	152
V$CRX_Q4	Crx	CRX	0.243011	149
V$HNF3A_01	HNF3A	FOXA1	0.140429	148
V$GATA1_04	GATA-1	GATA1	0.0128229	139
V$ERR1_Q3	ERR1	ESRRA	0.18197	135
V$CEBPE_Q6	CEBPE	CEBPE	0.0949125	133
V$HNF1_02	HNF-1alpha	HNF1A	0.363153	122
V$CRX_02	Crx	CRX	0.243011	119
V$NEUROD_02	NeuroD	NEUROD1	0.0402891	114
V$MAZ_Q6_01	MAZ	MAZ	0.414832	110
V$OC2_Q3	OC-2	ONECUT2	0.124899	102
V$IRF7_Q3	IRF-7	IRF7	0.13082	100
V$PIT1_Q6	Pit-1	POU1F1	0.319886	99
V$DBP_Q6_01	DBP	DBP	0.043001	76
V$CEBPG_Q6_01	C/EBPgamma	CEBPG	0.027117	73
V$MYOD_01	MyoD	MYOD1	0.306424	72
V$CREL_01	c-Rel	REL	0.29444	44
V$CEBPG_Q6	C/EBPgamma	CEBPG	0.027117	43
V$ATF4_Q6	ATF-4	ATF4	0.0251237	40
V$HOXA7_01	HOXA7	HOXA7	0.397453	31
V$E2F1_Q4	E2F-1	E2F1	0.110614	29
V$ATF5_01	ATF5	ATF5	0.912782	26

hsa-mir-124-1

Matrix_id	transcription factor	Gene	PCC	Occurrence
V$PUR1_Q4	PUR1	PURA	0.415305	488
V$MAFB_01	MAFB	MAFB	0.168403	452
V$PARP_Q4	PARP	PARP1	0.42105	444
V$ZIC3_01	Zic3	ZIC3	0.193283	441
V$ETS2_Q6	c-Ets-2	ETS2	0.191264	405
V$GABPA_Q4	GABP-alpha	GABPA	0.137733	392
V$TBP_Q6	TBP	TBP	0.0201133	383
V$AP4_Q6_02	AP-4	TFAP4	0.410215	365
V$YY1_01	YY1	YY1	0.0996927	358
V$GR_Q6	GR	NR3C1	0.0589335	352
V$MEF2C_Q4	MEF-2C	MEF2C	0.810817	348
V$YY1_Q6	YY1	YY1	0.0996927	330
V$YY1_Q6_02	YY1	YY1	0.0996927	330

V$ETS2_B	c-Ets-2	ETS2	0.191264	328
V$DLX5_01	dlx5	DLX5	0.00562739	325
V$SOX9_B1	SOX9	SOX9	0.237282	320
V$SRY_02	SRY	SRY	0.175179	291
V$PARP_Q3	PARP	PARP1	0.42105	286
V$ING4_01	ING4	ING4	0.696964	281
V$HNF3B_Q6	HNF-3beta	FOXA2	0.0600266	272
V$FOXO3A_Q1	FOXO3A	FOXO3	0.0455022	262
V$PBX1_04	Pbx1	PBX1	0.226624	257
V$YY1_Q6_03	YY1	YY1	0.0996927	248
V$IRF8_Q6	IRF-8	IRF8	0.0939808	214
V$CREM_Q6	CREM	CREM	0.0171356	213
V$GR_01	GR	NR3C1	0.0589335	205
V$CP2_01	CP2	TFCP2	0.248145	174
V$NEUROD_02	NeuroD	NEUROD1	0.0986588	172
V$ATF1_Q6_01	ATF-1	ATF1	0.00388072	169
V$NKX2B_Q3	NKX2B	NKX2-2	0.879144	155
V$FAC1_01	FAC1	BPTF	0.141342	151
V$NURR1_Q3	NURR1	NR4A2	0.125676	149
V$NKX22_02	NKX2B	NKX2-2	0.879144	149
V$LHX3b_01	LHX3b	LHX3	0.014696	148
V$DBP_Q6_01	DBP	DBP	0.268051	146
V$HOXB8_01	HOXB8	HOXB8	0.317144	106
V$DEC2_Q2	2-Dec	BHLHE41	0.670424	95
V$E2F1_Q4	E2F-1	E2F1	0.133834	79
V$STAT4_Q5	STAT4	STAT4	0.0379536	77
V$LHX3_01	Lhx3	LHX3	0.014696	74
V$CDP_04	CDP	CUX1	0.0599151	68
V$AMEF2_Q6	aMEF-2	MEF2A	0.671304	54
V$IRF2_01	IRF-2	IRF2	0.00731017	44
V$RSRFC4_Q2	RSRFC4	MEF2A	0.671304	41
V$RSRFC4_01	RSRFC4	MEF2A	0.671304	38

----

hsa-mir-124-2

Matrix_id	transcription factor	Gene	PCC	Occurrence
V$PUR1_Q4	PUR1	PURA	0.415305	488
V$MAFB_01	MAFB	MAFB	0.168403	452
V$PARP_Q4	PARP	PARP1	0.42105	444
V$ZIC3_01	Zic3	ZIC3	0.193283	441
V$ETS2_Q6	c-Ets-2	ETS2	0.191264	405
V$GABPA_Q4	GABP-alpha	GABPA	0.137733	392
V$SOX9_Q4	SOX9	SOX9	0.237282	392

V$TBP_Q6	TBP	TBP	0.0201133	383
V$AP4_Q6_02	AP-4	TFAP4	0.410215	365
V$MAZ_Q6	MAZ	MAZ	0.22392	358
V$YY1_01	YY1	YY1	0.0996927	358
V$GR_Q6	GR	NR3C1	0.0589335	352
V$MEF2C_Q4	MEF-2C	MEF2C	0.810817	348
V$YY1_Q6_02	YY1	YY1	0.0996927	330
V$YY1_Q6	YY1	YY1	0.0996927	330
V$ETS2_B	c-Ets-2	ETS2	0.191264	328
V$DLX5_01	dlx5	DLX5	0.00562739	325
V$SOX9_B1	SOX9	SOX9	0.237282	320
V$ZBP89_Q4	ZBP89	ZNF148	0.114715	291
V$SRY_02	SRY	SRY	0.175179	291
V$PARP_Q3	PARP	PARP1	0.42105	286
V$TFIII_Q6	TFII-I	GTF2I	0.265351	286
V$ING4_01	ING4	ING4	0.696964	281
V$FOXO3A_Q1	FOXO3A	FOXO3	0.0455022	262
V$PITX3_Q2	PITX3	PITX3	0.0373143	257
V$PBX1_04	Pbx1	PBX1	0.226624	257
V$MAZ_Q6_01	MAZ	MAZ	0.22392	253
V$YY1_Q6_03	YY1	YY1	0.0996927	248
V$KLF15_Q2	KLF15	KLF15	0.0367954	220
V$IRF8_Q6	IRF-8	IRF8	0.0939808	214
V$SREBP1_Q6	SREBP-1	SREBF1	0.015246	214
V$CREM_Q6	CREM	CREM	0.0171356	213
V$SOX10_Q6	SOX10	SOX10	0.725179	207
V$GR_01	GR	NR3C1	0.0589335	205
V$CP2_01	CP2	TFCP2	0.248145	174
V$ATF1_Q6_01	ATF-1	ATF1	0.00388072	169
V$MEF2A_Q6	mef2A	MEF2A	0.671304	149
V$LHX3b_01	LHX3b	LHX3	0.014696	148
V$ATF4_Q6	ATF-4	ATF4	0.0796929	115
V$HOXB8_01	HOXB8	HOXB8	0.317144	106
V$EAR2_Q2	EAR2	NR2F6	0.00028374	102
V$DEC2_Q2	2-Dec	BHLHE41	0.670424	95
V$ATF2_Q5	ATF-2	ATF2	0.0154679	81
V$EGR2_01	Egr-2	EGR2	0.220865	81
V$ERR3_Q2	ERR3	ESRRG	0.0992981	76
V$LHX3_01	Lhx3	LHX3	0.014696	74
V$CEBPG_Q6	C/EBPgamma	CEBPG	0.128006	66
V$ERR3_Q2_01	ERR3	ESRRG	0.0992981	60
V$AMEF2_Q6	aMEF-2	MEF2A	0.671304	54
V$LHX3A_01	Lhx3a	LHX3	0.014696	22
V$MEF2A_05	MEF2A	MEF2A	0.671304	20

----

hsa-mir-124-3

Matrix_id	transcription factor	Gene	PCC	Occurrence
V$PUR1_Q4	PUR1	PURA	0.415305	488
V$MAFB_01	MAFB	MAFB	0.168403	452
V$PARP_Q4	PARP	PARP1	0.42105	444
V$ZIC3_01	Zic3	ZIC3	0.193283	441
V$ETS2_Q6	c-Ets-2	ETS2	0.191264	405
V$SOX9_Q4	SOX9	SOX9	0.237282	392
V$GABPA_Q4	GABP-alpha	GABPA	0.137733	392
V$AP4_Q6_02	AP-4	TFAP4	0.410215	365
V$YY1_Q6_02	YY1	YY1	0.0996927	330
V$YY1_Q6	YY1	YY1	0.0996927	330
V$ETS2_B	c-Ets-2	ETS2	0.191264	328
V$DLX5_01	dlx5	DLX5	0.00562739	325
V$ZBP89_Q4	ZBP89	ZNF148	0.114715	291
V$TFIII_Q6	TFII-I	GTF2I	0.265351	286
V$ELK1_02	Elk-1	ELK1	0.10691	271
V$FOXO3A_Q1	FOXO3A	FOXO3	0.0455022	262
V$AP4_Q5	AP-4	TFAP4	0.410215	254
V$AP4_Q6	AP-4	TFAP4	0.410215	232
V$IRF8_Q6	IRF-8	IRF8	0.0939808	214
V$SREBP1_Q6	SREBP-1	SREBF1	0.015246	214
V$CREM_Q6	CREM	CREM	0.0171356	213
V$SOX10_Q6	SOX10	SOX10	0.725179	207
V$SP2_01	SP2	SP2	0.0894308	196
V$E2F1_Q3_01	E2F-1	E2F1	0.133834	181
V$E2F1_Q3	E2F-1	E2F1	0.133834	181
V$CP2_01	CP2	TFCP2	0.248145	174
V$NEUROD_02	NeuroD	NEUROD1	0.0986588	172
V$ATF1_Q6_01	ATF-1	ATF1	0.00388072	169
V$SP4_Q5	SP4	SP4	0.038657	162
V$AP4_Q6_01	AP-4	TFAP4	0.410215	162
V$NURR1_Q3	NURR1	NR4A2	0.125676	149
V$E2F1_Q6	E2F-1	E2F1	0.133834	145
V$YY1_02	YY1	YY1	0.0996927	141
V$ATF4_Q6	ATF-4	ATF4	0.0796929	115
V$HOXB8_01	HOXB8	HOXB8	0.317144	106
V$ATF2_Q5	ATF-2	ATF2	0.0154679	81
V$EGR2_01	Egr-2	EGR2	0.220865	81

hsa-mir-125a

Matrix_id	transcription factor	Gene	PCC	Occurrence
V$PUR1_Q4	PUR1	PURA	0.183695	8
V$GKLF_Q4	GKLF	KLF4	0.42926	8
V$GABPA_Q4	GABP-alpha	GABPA	0.0633009	7
V$ING4_01	ING4	ING4	0.0913762	6
V$SP1_Q4_01	Sp1	SP1	0.105232	6
V$SP1_Q6_01	Sp1	SP1	0.105232	6
V$FKLF_Q5	FKLF	KLF11	0.225845	6
V$AP2ALPHA_01	AP-2alpha	TFAP2A	0.643495	6
V$GATA6_01	GATA-6	GATA6	0.366278	6
V$GATA2_02	GATA-2	GATA2	0.437837	6
V$GATA3_01	GATA-3	GATA3	0.604553	6
V$YY1_01	YY1	YY1	0.241894	6
V$SP1_Q6	Sp1	SP1	0.105232	6
V$ELK1_06	ELK-1	ELK1	0.0671742	5
V$TBP_Q6	TBP	TBP	0.158908	5
V$WT1_Q6	WT1	WT1	0.168605	5
V$ELK1_02	Elk-1	ELK1	0.0671742	5
V$SP1_01	Sp1	SP1	0.105232	5
V$WT1_Q6_01	WT1	WT1	0.168605	5
V$SP1_Q2_01	Sp1	SP1	0.105232	5
V$AHR_Q5	AhR	AHR	0.683838	5
V$SP1_02	SP1	SP1	0.105232	5
V$AP2ALPHA_Q6	AP-2alpha	TFAP2A	0.643495	5
V$GR_Q6	GR	NR3C1	0.0138162	5
V$GATA3_02	GATA-3	GATA3	0.604553	5
V$SMAD4_Q6_01	Smad4	SMAD4	0.120369	5
V$YY1_Q6_03	YY1	YY1	0.241894	5
V$SP2_01	SP2	SP2	0.0487716	4
V$CIZ_01	CIZ	ZNF384	0.0497608	4
V$RNF96_01	RNF96	TRIM28	0.222863	4
V$GR_01	GR	NR3C1	0.0138162	4
V$CP2_01	CP2	TFCP2	0.444727	4
V$GATA2_01	GATA-2	GATA2	0.437837	4
V$P53_02	p53	TP53	0.220725	4
V$ZABC1_01	ZABC1	ZNF217	0.500447	3
V$SMAD4_Q6	SMAD4	SMAD4	0.120369	3
V$GABPBETA_Q3	GABP-beta	GABPB1	0.202144	3
V$TCF4_Q5	TCF-4	TCF7L2	0.406284	2
V$GLI3_Q5_01	GLI3	GLI3	0.0344213	2
V$CREM_Q6	CREM	CREM	0.320594	2
V$MEIS1_01	MEIS1	MEIS1	0.295294	1
V$ELK1_01	Elk-1	ELK1	0.0671742	1
V$ATF3_Q6_01	ATF-3	ATF3	0.240844	1

----

hsa-mir-126

Matrix_id	transcription factor	Gene	PCC	Occurrence
V$MAFB_01	MAFB	MAFB	0.56019	13
V$SMAD4_Q6_01	Smad4	SMAD4	0.0696685	12
V$ELF1_Q6	Elf-1	ELF1	0.0377749	12
V$GKLF_Q4	GKLF	KLF4	0.665474	12
V$P300_01	p300	EP300	0.107642	11
V$GATA2_02	GATA-2	GATA2	0.41508	11
V$GATA3_02	GATA-3	GATA3	0.493125	11
V$GATA6_01	GATA-6	GATA6	0.33894	11
V$TBX5_02	TBX5	TBX5	0.0657622	11
V$GATA1_05	GATA-1	GATA1	0.284604	11
V$AML1_Q6	AML1	RUNX1	0.127943	11
V$GATA1_06	GATA-1	GATA1	0.284604	11
V$GATA1_02	GATA-1	GATA1	0.284604	11
V$GATA1_01	GATA-1	GATA1	0.284604	10
V$TBX5_01	TBX5	TBX5	0.0657622	10
V$CETS1_Q6	C-ets-1	ETS1	0.10267	10
V$ETS2_Q6	c-Ets-2	ETS2	0.102418	10
V$ETS1_B	c-Ets-1	ETS1	0.10267	10
V$ETS2_B	c-Ets-2	ETS2	0.102418	10
V$YY1_Q6	YY1	YY1	0.163781	9
V$YY1_Q6_02	YY1	YY1	0.163781	8
V$FKLF_Q5	FKLF	KLF11	0.37236	8
V$SPI1_Q5	SPI1	SPI1	0.484517	8
V$TCF4_Q5	TCF-4	TCF7L2	0.444716	8
V$SREBP1_Q6	SREBP-1	SREBF1	0.10043	8
V$CP2_01	CP2	TFCP2	0.149265	8
V$AP2ALPHA_Q6	AP-2alpha	TFAP2A	0.537871	7
V$AHR_Q5	AhR	AHR	0.66393	7
V$AML2_Q3	AML2	RUNX3	0.251293	7
V$LRF_Q2	LRF	ZBTB7A	0.00379928	7
V$GATA1_04	GATA-1	GATA1	0.284604	7
V$IRF8_Q6	IRF-8	IRF8	0.124863	6
V$AML2_Q3_01	AML2	RUNX3	0.251293	6
V$P53_02	p53	TP53	0.222258	6
V$HMGIY_01	HMGIY	HMGA1	0.0370167	6
V$ARNT_01	Arnt	ARNT	0.190972	6
V$GATA2_03	GATA-2	GATA2	0.41508	5
V$CEBPB_Q6	C/EBPbeta	CEBPB	0.665841	5
V$TCF4_01	TCF-4	TCF7L2	0.444716	5

V$CEBPD_Q6	C/EBPdelta	CEBPD	0.665534	5
V$AML1_01	AML1a	RUNX1	0.127943	5
V$SP1_02	SP1	SP1	0.244451	4
V$AP2ALPHA_01	AP-2alpha	TFAP2A	0.537871	4
V$SP1_Q6_01	Sp1	SP1	0.244451	4
V$DEC2_Q2	2-Dec	BHLHE41	0.0638497	3
V$EAR2_Q2	EAR2	NR2F6	0.188304	3
V$AML1_Q4	AML1	RUNX1	0.127943	3
V$AP2ALPHA_02	AP-2alphaA	TFAP2A	0.537871	3
V$IRF7_01	IRF-7	IRF7	0.639271	3
V$SREBP1_02	SREBP-1	SREBF1	0.10043	2
V$IRF1_Q6_01	IRF-1	IRF1	0.296333	2
V$AP2GAMMA_01	AP-2gamma	TFAP2C	0.478627	2
V$IRF7_Q3	IRF-7	IRF7	0.639271	1
V$IRF1_Q6	IRF-1	IRF1	0.296333	1

---

hsa-mir-127

Matrix_id	transcription factor	Gene	PCC	Occurrence
V$PUR1_Q4	PUR1	PURA	0.0763966	44
V$MAFB_01	MAFB	MAFB	0.0165872	42
V$GKLF_Q4	GKLF	KLF4	0.0778385	42
V$PARP_Q4	PARP	PARP1	0.159384	40
V$P300_01	p300	EP300	0.0265468	39
V$PEA3_Q6	PEA3	ETV4	0.0205414	38
V$TBP_Q6	TBP	TBP	0.0671996	37
V$GATA6_01	GATA-6	GATA6	0.557475	35
V$GATA2_02	GATA-2	GATA2	0.0663594	35
V$GABPA_Q4	GABP-alpha	GABPA	0.153042	33
V$MEF2C_Q4	MEF-2C	MEF2C	0.150333	32
V$YY1_01	YY1	YY1	0.205443	32
V$MAZ_Q6	MAZ	MAZ	0.0153349	31
V$GATA3_02	GATA-3	GATA3	0.214396	31
V$GATA3_01	GATA-3	GATA3	0.214396	30
V$DLX5_01	dlx5	DLX5	0.389742	27
V$GATA2_01	GATA-2	GATA2	0.0663594	27
V$PARP_Q3	PARP	PARP1	0.159384	26
V$PBX1_Q3	Pbx1	PBX1	0.263068	25
V$PBX1_04	Pbx1	PBX1	0.263068	25
V$ING4_01	ING4	ING4	0.0684935	23
V$GATA3_03	GATA-3	GATA3	0.214396	23
V$AHR_Q5	AhR	AHR	0.207381	21
V$MAZ_Q6_01	MAZ	MAZ	0.0153349	20

V$CDX1_01	Cdx-1	CDX1	0.0279134	18
V$TCF4_01	TCF-4	TCF7L2	0.0190837	18
V$FOXJ2_01	FOXJ2	FOXJ2	0.187553	17
V$PIT1_Q6	Pit-1	POU1F1	0.246501	17
V$GATA2_03	GATA-2	GATA2	0.0663594	15
V$GABPBETA_Q3	GABP-beta	GABPB1	0.0872082	15
V$HNF6_Q6	HNF6	ONECUT1	0.325378	14
V$HBP1_Q2	hbp1	HBP1	0.234734	12
V$YY1_02	YY1	YY1	0.205443	12
V$HOX13_02	HOXA5	HOXA5	0.741616	11
V$MEIS1_01	MEIS1	MEIS1	0.244201	10
V$CIZ_01	CIZ	ZNF384	0.303459	10
V$ELK1_01	Elk-1	ELK1	0.26451	9
V$CART1_02	CART1	ALX1	0.0545584	9
V$OC2_Q3	OC-2	ONECUT2	0.0832852	9
V$RORA1_01	RORalpha1	RORA	0.45594	7
V$IRF2_01	IRF-2	IRF2	0.520216	7
V$OCT2_01	2-Oct	POU2F2	0.0239429	5

----

hsa-mir-128-1

Matrix_id	transcription factor	Gene	PCC	Occurrence
V$PUR1_Q4	PUR1	PURA	0.440977	368
V$IK_Q5	Ikaros	IKZF1	0.337253	364
V$PEA3_Q6	PEA3	ETV4	0.0554766	352
V$PARP_Q4	PARP	PARP1	0.619831	337
V$ZIC3_01	Zic3	ZIC3	0.183524	335
V$MAFB_01	MAFB	MAFB	0.0172242	334
V$AP2REP_01	AP-2rep	KLF12	0.161844	330
V$P300_01	p300	EP300	0.170577	327
V$TBX5_02	TBX5	TBX5	0.111103	301
V$CETS1_Q6	C-ets-1	ETS1	0.0953673	301
V$ETS2_Q6	c-Ets-2	ETS2	0.360013	300
V$MYB_Q6	c-Myb	MYB	0.378662	298
V$CMYB_Q5	c-Myb	MYB	0.378662	298
V$ETS1_B	c-Ets-1	ETS1	0.0953673	296
V$SOX9_Q4	SOX9	SOX9	0.111326	295
V$NFAT4_Q3	NF-AT4	NFATC3	0.273329	291
V$TBP_Q6	TBP	TBP	0.0438854	287
V$CDX2_Q5_02	CDX-2	CDX2	0.174017	287
V$GABPA_Q4	GABP-alpha	GABPA	0.19654	286
V$YY1_01	YY1	YY1	0.224304	271
V$AP4_Q6_02	AP-4	TFAP4	0.458864	270

V$GR_Q6	GR	NR3C1	0.298956	268
V$BEN_01	BEN	GTF2IRD1	0.247748	261
V$AML1_Q6	AML1	RUNX1	0.112435	257
V$YY1_Q6	YY1	YY1	0.224304	250
V$MEF2C_Q4	MEF-2C	MEF2C	0.86629	250
V$YY1_Q6_02	YY1	YY1	0.224304	246
V$ETS2_B	c-Ets-2	ETS2	0.360013	246
V$SP1_Q6	Sp1	SP1	0.106283	242
V$SOX9_B1	SOX9	SOX9	0.111326	241
V$ZBP89_Q4	ZBP89	ZNF148	0.338395	239
V$DLX5_01	dlx5	DLX5	0.060424	235
V$TBX5_01	TBX5	TBX5	0.111103	233
V$CDX2_Q5_01	Cdx-2	CDX2	0.174017	231
V$IPF1_01	IPF1	PDX1	0.211926	228
V$SP1_Q6_01	Sp1	SP1	0.106283	227
V$SP1_01	Sp1	SP1	0.106283	223
V$NKX32_01	Nkx3-2	NKX3-2	0.178923	222
V$PARP_Q3	PARP	PARP1	0.619831	219
V$SP1_Q4_01	Sp1	SP1	0.106283	219
V$GATA1_05	GATA-1	GATA1	0.0203872	219
V$GATA1_06	GATA-1	GATA1	0.0203872	219
V$SP1_Q2_01	Sp1	SP1	0.106283	218
V$E47_02	E47	TCF3	0.161979	217
V$E12_Q6	E12	TCF3	0.161979	217
V$MYOD_Q6_01	MyoD	MYOD1	0.163917	217
V$E2A_Q6	E2A	TCF3	0.161979	217
V$ING4_01	ING4	ING4	0.709806	216
V$SP1_02	SP1	SP1	0.106283	212
V$IRF4_Q6	IRF-4	IRF4	0.169721	211
V$MYOGENIN_Q6	myogenin	MYOG	0.160997	211
V$HMGIY_01	HMGIY	HMGA1	0.116398	201
V$TEL1_02	TEL1	ETV6	0.264841	200
V$ELK1_02	Elk-1	ELK1	0.260213	200
V$MAZ_Q6_01	MAZ	MAZ	0.236493	199
V$ARNT_01	Arnt	ARNT	0.100035	198
V$ETV3_02	ETV3	ETV3	0.020757	192
V$PBX1_Q3	Pbx1	PBX1	0.0630592	191
V$YY1_Q6_03	YY1	YY1	0.224304	185
V$CNOT3_01	CNOT3	CNOT3	0.115739	176
V$ELK1_06	ELK-1	ELK1	0.260213	174
V$CMYB_01	c-Myb	MYB	0.378662	174
V$ZFX_01	Zfx	ZFX	0.185787	173
V$E2A_Q2	E2A	TCF3	0.161979	173
V$LEF1_Q5	LEF-1	LEF1	0.341588	170
V$ETV7_01	ETV7	ETV7	0.209903	168
V$ERF_02	ERF	ERF	0.315025	160

V$CREM_Q6	CREM	CREM	0.0642486	157
V$IRF8_Q6	IRF-8	IRF8	0.136582	156
V$SP2_01	SP2	SP2	0.253567	141
V$MYOD_Q6	MyoD	MYOD1	0.163917	136
V$E2F1_Q3	E2F-1	E2F1	0.230356	129
V$E47_01	E47	TCF3	0.161979	126
V$ATF1_Q6_01	ATF-1	ATF1	0.13103	124
V$GABPBETA_Q3	GABP-beta	GABPB1	0.120451	122
V$NKX22_02	NKX2B	NKX2-2	0.852477	110
V$YY1_02	YY1	YY1	0.224304	104
V$E2F1_Q6	E2F-1	E2F1	0.230356	103
V$PET1_02	Pet-1	FEV	0.0264327	100
V$ERG_03	ERG	ERG	0.0659125	97
V$ELK1_01	Elk-1	ELK1	0.260213	92
V$ATF4_Q6	ATF-4	ATF4	0.361983	92
V$MYOD_01	MyoD	MYOD1	0.163917	91
V$CIZ_01	CIZ	ZNF384	0.0872695	89
V$ERM_02	Erm	ETV5	0.125972	89
V$BEN_02	BEN	GTF2IRD1	0.247748	82
V$P53_02	p53	TP53	0.0353976	80
V$ERG_01	ERG	ERG	0.0659125	80
V$SPIB_03	Spi-B	SPIB	0.0486756	77
V$DEC2_Q2	2-Dec	BHLHE41	0.510249	71
V$YY1_03	YY1	YY1	0.224304	67
V$ATF2_Q5	ATF-2	ATF2	0.271434	66
V$SMAD4_Q6	SMAD4	SMAD4	0.101403	59
V$SAP1A_01	SAP-1a	ELK4	0.178852	56
V$STAT4_Q5	STAT4	STAT4	0.254948	55
V$SPIB_01	SPI-B	SPIB	0.0486756	54
V$FLI1_02	Fli-1	FLI1	0.15984	54
V$STAF_02	Staf	ZNF143	0.256526	50
V$RSRFC4_Q2	RSRFC4	MEF2A	0.591716	35
V$RSRFC4_01	RSRFC4	MEF2A	0.591716	34

---

hsa-mir-128-2

Matrix_id	transcription factor	Gene	PCC	Occurrence
V$PUR1_Q4	PUR1	PURA	0.440977	368
V$IK_Q5	Ikaros	IKZF1	0.337253	364
V$PEA3_Q6	PEA3	ETV4	0.0554766	352
V$PARP_Q4	PARP	PARP1	0.619831	337
V$MAFB_01	MAFB	MAFB	0.0172242	334
V$AP2REP_01	AP-2rep	KLF12	0.161844	330

V$SMAD4_Q6_01	Smad4	SMAD4	0.101403	312
V$CETS1_Q6	C-ets-1	ETS1	0.0953673	301
V$TBX5_02	TBX5	TBX5	0.111103	301
V$ETS2_Q6	c-Ets-2	ETS2	0.360013	300
V$MYB_Q6	c-Myb	MYB	0.378662	298
V$CMYB_Q5	c-Myb	MYB	0.378662	298
V$ETS1_B	c-Ets-1	ETS1	0.0953673	296
V$SOX9_Q4	SOX9	SOX9	0.111326	295
V$CDX2_Q5_02	CDX-2	CDX2	0.174017	287
V$TBP_Q6	TBP	TBP	0.0438854	287
V$GABPA_Q4	GABP-alpha	GABPA	0.19654	286
V$SMAD3_Q6_01	Smad3	SMAD3	0.241707	286
V$YY1_01	YY1	YY1	0.224304	271
V$AP4_Q6_02	AP-4	TFAP4	0.458864	270
V$GR_Q6	GR	NR3C1	0.298956	268
V$BEN_01	BEN	GTF2IRD1	0.247748	261
V$AML1_Q6	AML1	RUNX1	0.112435	257
V$MEF2C_Q4	MEF-2C	MEF2C	0.86629	250
V$YY1_Q6	YY1	YY1	0.224304	250
V$YY1_Q6_02	YY1	YY1	0.224304	246
V$ETS2_B	c-Ets-2	ETS2	0.360013	246
V$NANOG_02	Nanog	NANOG	0.173492	245
V$SOX9_B1	SOX9	SOX9	0.111326	241
V$GATA1_02	GATA-1	GATA1	0.0203872	240
V$DLX5_01	dlx5	DLX5	0.060424	235
V$TBX5_01	TBX5	TBX5	0.111103	233
V$CDX2_Q5_01	Cdx-2	CDX2	0.174017	231
V$IPF1_01	IPF1	PDX1	0.211926	228
V$SRY_02	SRY	SRY	0.305661	221
V$PARP_Q3	PARP	PARP1	0.619831	219
V$E12_Q6	E12	TCF3	0.161979	217
V$E47_02	E47	TCF3	0.161979	217
V$MYOD_Q6_01	MyoD	MYOD1	0.163917	217
V$E2A_Q6	E2A	TCF3	0.161979	217
V$ING4_01	ING4	ING4	0.709806	216
V$NFAT2_Q5	NF-AT2	NFATC1	0.143227	216
V$CDX2_01	Cdx-2	CDX2	0.174017	212
V$MYOGENIN_Q6	myogenin	MYOG	0.160997	211
V$IRF4_Q6	IRF-4	IRF4	0.169721	211
V$HNF3B_Q6	HNF-3beta	FOXA2	0.166871	205
V$GATA1_04	GATA-1	GATA1	0.0203872	204
V$FOXO3A_Q1	FOXO3A	FOXO3	0.259516	204
V$ELK1_02	Elk-1	ELK1	0.260213	200
V$TEL1_02	TEL1	ETV6	0.264841	200
V$MAZ_Q6_01	MAZ	MAZ	0.236493	199
V$ARNT_01	Arnt	ARNT	0.100035	198

V$PBX1_04	Pbx1	PBX1	0.0630592	193
V$ETV3_02	ETV3	ETV3	0.020757	192
V$PBX1_Q3	Pbx1	PBX1	0.0630592	191
V$AP4_Q5	AP-4	TFAP4	0.458864	185
V$YY1_Q6_03	YY1	YY1	0.224304	185
V$PITX3_Q2	PITX3	PITX3	0.192608	183
V$GFI1_Q6	Gfi1	GFI1	0.358669	177
V$AP4_Q6	AP-4	TFAP4	0.458864	175
V$CMYB_01	c-Myb	MYB	0.378662	174
V$ELK1_06	ELK-1	ELK1	0.260213	174
V$E2A_Q2	E2A	TCF3	0.161979	173
V$GFI1_Q6_01	Gfi1	GFI1	0.358669	168
V$HNF1_02	HNF-1alpha	HNF1A	0.0554183	164
V$ERF_02	ERF	ERF	0.315025	160
V$MYOGENIN_Q6_01	myogenin	MYOG	0.160997	160
V$IRF8_Q6	IRF-8	IRF8	0.136582	156
V$FOXJ2_01	FOXJ2	FOXJ2	0.109748	154
V$FOXP3_01	FOXP3	FOXP3	0.184892	152
V$GFI1B_01	Gfi1b	GFI1B	0.173764	143
V$MYOD_Q6	MyoD	MYOD1	0.163917	136
V$NEUROD_02	NeuroD	NEUROD1	0.379829	136
V$BCL6_Q3_01	Bcl-6	BCL6	0.290989	129
V$E2F1_Q3	E2F-1	E2F1	0.230356	129
V$E47_01	E47	TCF3	0.161979	126
V$AML1_01	AML1a	RUNX1	0.112435	125
V$OC2_Q3	OC-2	ONECUT2	0.135491	125
V$DAX1_01	Dax1	NR0B1	0.0438224	125
V$ATF1_Q6_01	ATF-1	ATF1	0.13103	124
V$FAC1_01	FAC1	BPTF	0.217786	112
V$AML1_Q4	AML1	RUNX1	0.112435	111
V$MEF2A_Q6	mef2A	MEF2A	0.591716	110
V$DBP_Q6_01	DBP	DBP	0.47676	108
V$LHX3b_01	LHX3b	LHX3	0.22777	108
V$HOXD9_Q2	Hoxd9	HOXD9	0.0117175	107
V$MYOD_01	MyoD	MYOD1	0.163917	91
V$CIZ_01	CIZ	ZNF384	0.0872695	89
V$HTF4_Q2	HTF4	TCF12	0.26246	85
V$HOXB8_01	HOXB8	HOXB8	0.420728	79
V$NCX_02	Ncx	TLX2	0.219675	79
V$DEC2_Q2	2-Dec	BHLHE41	0.510249	71
V$E2F1_Q4	E2F-1	E2F1	0.230356	63
V$FOXJ2_02	FOXJ2	FOXJ2	0.109748	62
V$SPIB_01	SPI-B	SPIB	0.0486756	54

----

hsa-mir-129-1

Matrix_id	transcription factor	Gene	PCC	Occurrence
V$PUR1_Q4	PUR1	PURA	0.389458	495
V$MAFB_01	MAFB	MAFB	0.133513	457
V$PARP_Q4	PARP	PARP1	0.448735	453
V$ZIC3_01	Zic3	ZIC3	0.248691	450
V$SOX9_Q4	SOX9	SOX9	0.331727	395
V$AP4_Q6_02	AP-4	TFAP4	0.414392	368
V$MAZ_Q6	MAZ	MAZ	0.277827	366
V$GR_Q6	GR	NR3C1	0.0290927	354
V$MEF2C_Q4	MEF-2C	MEF2C	0.801687	351
V$YY1_Q6	YY1	YY1	0.120808	337
V$YY1_Q6_02	YY1	YY1	0.120808	335
V$SOX9_B1	SOX9	SOX9	0.331727	329
V$DLX5_01	dlx5	DLX5	0.0483772	329
V$ZBP89_Q4	ZBP89	ZNF148	0.102069	295
V$TFIII_Q6	TFII-I	GTF2I	0.234131	290
V$HNF3B_Q6	HNF-3beta	FOXA2	0.0752312	278
V$ELK1_02	Elk-1	ELK1	0.146971	275
V$MAZ_Q6_01	MAZ	MAZ	0.277827	259
V$YY1_Q6_03	YY1	YY1	0.120808	255
V$AP4_Q5	AP-4	TFAP4	0.414392	255
V$ELK1_06	ELK-1	ELK1	0.146971	239
V$AP4_Q6	AP-4	TFAP4	0.414392	231
V$KLF15_Q2	KLF15	KLF15	0.0194347	221
V$SREBP1_Q6	SREBP-1	SREBF1	0.0369535	218
V$CREM_Q6	CREM	CREM	0.0948141	215
V$SOX10_Q6	SOX10	SOX10	0.722931	209
V$GR_01	GR	NR3C1	0.0290927	205
V$E2F1_Q3_01	E2F-1	E2F1	0.197641	184
V$E2F1_Q3	E2F-1	E2F1	0.197641	184
V$CP2_01	CP2	TFCP2	0.233849	181
V$NEUROD_02	NeuroD	NEUROD1	0.0885898	177
V$ZIC1_01	Zic1	ZIC1	0.945802	175
V$LHX3b_01	LHX3b	LHX3	0.0408343	149
V$MEF2A_Q6	mef2A	MEF2A	0.658035	148
V$E2F1_Q6	E2F-1	E2F1	0.197641	145
V$CACCCBINDINGFACT	CACCC-binding factor	ZNF148	0.102069	120
V$ATF4_Q6	ATF-4	ATF4	0.0734864	117
V$ATF2_Q5	ATF-2	ATF2	0.0329958	83
V$E2F1_Q4	E2F-1	E2F1	0.197641	83
V$RORBETA_Q2	RORBETA	RORB	0.323883	79
V$ZID_01	ZID	ZBTB6	0.0774791	45

hsa-mir-129-2

Matrix_id	transcription factor	Gene	PCC	Occurrence
V$PUR1_Q4	PUR1	PURA	0.389458	495
V$MAFB_01	MAFB	MAFB	0.133513	457
V$ZIC3_01	Zic3	ZIC3	0.248691	450
V$ETS2_Q6	c-Ets-2	ETS2	0.163768	405
V$GABPA_Q4	GABP-alpha	GABPA	0.144381	396
V$SOX9_Q4	SOX9	SOX9	0.331727	395
V$TBP_Q6	TBP	TBP	0.137566	393
V$MAZ_Q6	MAZ	MAZ	0.277827	366
V$YY1_01	YY1	YY1	0.120808	364
V$MEF2C_Q4	MEF-2C	MEF2C	0.801687	351
V$YY1_Q6	YY1	YY1	0.120808	337
V$DLX5_01	dlx5	DLX5	0.0483772	329
V$SOX9_B1	SOX9	SOX9	0.331727	329
V$SRY_02	SRY	SRY	0.197346	299
V$ZBP89_Q4	ZBP89	ZNF148	0.102069	295
V$ING4_01	ING4	ING4	0.706475	294
V$TFIII_Q6	TFII-I	GTF2I	0.234131	290
V$PARP_Q3	PARP	PARP1	0.448735	289
V$HNF3B_Q6	HNF-3beta	FOXA2	0.0752312	278
V$ELK1_02	Elk-1	ELK1	0.146971	275
V$FOXO3A_Q1	FOXO3A	FOXO3	0.0434161	269
V$HMGIY_01	HMGIY	HMGA1	0.00635564	267
V$HIF1A_Q6	HIF-1alpha	HIF1A	0.00104194	255
V$ELK1_06	ELK-1	ELK1	0.146971	239
V$SREBP1_Q6	SREBP-1	SREBF1	0.0369535	218
V$CREM_Q6	CREM	CREM	0.0948141	215
V$ERF_02	ERF	ERF	0.242571	214
V$SOX10_Q6	SOX10	SOX10	0.722931	209
V$E2F1_Q3_01	E2F-1	E2F1	0.197641	184
V$ZIC1_01	Zic1	ZIC1	0.945802	175
V$SP4_Q5	SP4	SP4	0.0514432	165
V$MSX1_01	Msx-1	MSX1	0.1602	120
V$ATF4_Q6	ATF-4	ATF4	0.0734864	117
V$PAX3_B	Pax-3	PAX3	0.0532104	103
V$USF2_Q6	USF2	USF2	0.320827	85
V$E2F1_Q4	E2F-1	E2F1	0.197641	83
V$MAX_01	Max	MAX	0.541682	82
V$EGR2_01	Egr-2	EGR2	0.260654	80
V$SOX2_Q6	SOX2	SOX2	0.535576	79
V$CTCF_02	CTCF	CTCF	0.415948	55

V$CTCF_01	CTCF	CTCF	0.415948	54

----

hsa-mir-130a

Matrix_id	transcription factor	Gene	PCC	Occurrence
V$PUR1_Q4	PUR1	PURA	0.116355	206
V$GKLF_Q4	GKLF	KLF4	0.560999	197
V$ELF1_Q6	Elf-1	ELF1	0.164572	196
V$MAFB_01	MAFB	MAFB	0.269534	188
V$P300_01	p300	EP300	0.246139	184
V$SMAD4_Q6_01	Smad4	SMAD4	0.223118	172
V$GABPA_Q4	GABP-alpha	GABPA	0.279315	169
V$YY1_01	YY1	YY1	0.29121	153
V$TBP_Q6	TBP	TBP	0.0611908	151
V$YY1_Q6_02	YY1	YY1	0.29121	142
V$YY1_Q6	YY1	YY1	0.29121	141
V$DLX5_01	dlx5	DLX5	0.620126	136
V$AHR_Q5	AhR	AHR	0.910479	129
V$AP2ALPHA_Q6	AP-2alpha	TFAP2A	0.887202	121
V$WT1_Q6	WT1	WT1	0.0167677	117
V$TFIII_Q6	TFII-I	GTF2I	0.0195905	116
V$AP2ALPHA_01	AP-2alpha	TFAP2A	0.887202	112
V$GATA3_01	GATA-3	GATA3	0.868185	111
V$SREBP1_Q6	SREBP-1	SREBF1	0.139465	105
V$ARNT_01	Arnt	ARNT	0.26003	101
V$GATA2_01	GATA-2	GATA2	0.623351	100
V$SP1_01	Sp1	SP1	0.239241	100
V$WT1_Q6_01	WT1	WT1	0.0167677	95
V$TCF4_Q5	TCF-4	TCF7L2	0.496404	87
V$PITX2_Q2	Pitx2	PITX2	0.32663	83
V$CREM_Q6	CREM	CREM	0.0977584	78
V$PITX2_01	PITX2	PITX2	0.32663	77
V$GABPBETA_Q3	GABP-beta	GABPB1	0.153194	77
V$ESE1_Q3	ESE-1	ELF3	0.195519	74
V$GATA3_03	GATA-3	GATA3	0.868185	72
V$SP2_01	SP2	SP2	0.0862784	71
V$CEBPD_Q6	C/EBPdelta	CEBPD	0.417084	69
V$CEBPB_02	C/EBPbeta	CEBPB	0.526739	66
V$STAT3_03	STAT3	STAT3	0.176632	62
V$CEBPB_Q6	C/EBPbeta	CEBPB	0.526739	54
V$ATF1_Q6_01	ATF-1	ATF1	0.00417839	53
V$SP3_Q3	Sp3	SP3	0.380046	29
V$AP2ALPHA_02	AP-2alphaA	TFAP2A	0.887202	28

V$GLI3_02	GLI3	GLI3	0.23356	16
V$GLI3_01	GLI3	GLI3	0.23356	14

hsa-mir-130b

Matrix_id	transcription factor	Gene	PCC	Occurrence
V$PITX2_01	PITX2	PITX2	0.230026	3
V$MAFB_01	MAFB	MAFB	0.0274179	3
V$YY1_Q6_02	YY1	YY1	0.0372295	3
V$PUR1_Q4	PUR1	PURA	0.0158336	3
V$YY1_Q6	YY1	YY1	0.0372295	3
V$ELF1_Q6	Elf-1	ELF1	0.156631	3
V$GKLF_Q4	GKLF	KLF4	0.190808	3
V$PITX2_Q2	Pitx2	PITX2	0.230026	3
V$AP2ALPHA_Q6	AP-2alpha	TFAP2A	0.609786	3
V$CEBPB_02	C/EBPbeta	CEBPB	0.273995	3
V$AP2GAMMA_01	AP-2gamma	TFAP2C	0.490835	2
V$P300_01	p300	EP300	0.0373138	2
V$ESE1_Q3	ESE-1	ELF3	0.191992	2
V$HMGIY_01	HMGIY	HMGA1	0.22093	2
V$YY1_Q6_03	YY1	YY1	0.0372295	2
V$GABPA_Q4	GABP-alpha	GABPA	0.238255	2
V$TCF4_01	TCF-4	TCF7L2	0.10522	2
V$AHR_Q5	AhR	AHR	0.638405	2
V$ARNT_01	Arnt	ARNT	0.0193809	2
V$TCF4_Q5	TCF-4	TCF7L2	0.10522	2
V$AP2ALPHA_01	AP-2alpha	TFAP2A	0.609786	2
V$SP1_01	Sp1	SP1	0.0798378	1
V$SP1_02	SP1	SP1	0.0798378	1
V$RNF96_01	RNF96	TRIM28	0.139409	1
V$SP1_Q2_01	Sp1	SP1	0.0798378	1
V$SP1_Q4_01	Sp1	SP1	0.0798378	1
V$SP1_Q6_01	Sp1	SP1	0.0798378	1
V$SP3_Q3	Sp3	SP3	0.229309	1
V$SP1_Q6	Sp1	SP1	0.0798378	1
V$ZID_01	ZID	ZBTB6	0.0229121	1
V$ESE1_02	ESE-1	ELF3	0.191992	1

hsa-mir-132

Matrix_id	transcription factor	Gene	PCC	Occurrence

V$PUR1_Q4	PUR1	PURA	0.384592	420
V$MAFB_01	MAFB	MAFB	0.0436043	387
V$PARP_Q4	PARP	PARP1	0.389398	387
V$ZIC3_01	Zic3	ZIC3	0.219049	382
V$ETS2_Q6	c-Ets-2	ETS2	0.0817771	343
V$GABPA_Q4	GABP-alpha	GABPA	0.0552729	337
V$TBP_Q6	TBP	TBP	0.0880895	336
V$SOX9_Q4	SOX9	SOX9	0.284964	329
V$MAZ_Q6	MAZ	MAZ	0.246073	308
V$AP4_Q6_02	AP-4	TFAP4	0.357839	308
V$GR_Q6	GR	NR3C1	0.0149042	307
V$YY1_01	YY1	YY1	0.1231	306
V$HNF4A_Q6_01	HNF-4alpha	HNF4A	0.0513668	292
V$DLX5_01	dlx5	DLX5	0.041707	287
V$YY1_Q6	YY1	YY1	0.1231	283
V$ETS2_B	c-Ets-2	ETS2	0.0817771	282
V$YY1_Q6_02	YY1	YY1	0.1231	282
V$SOX9_B1	SOX9	SOX9	0.284964	281
V$GATA6_01	GATA-6	GATA6	0.0744763	261
V$SRY_02	SRY	SRY	0.107058	257
V$ZBP89_Q4	ZBP89	ZNF148	0.0759542	249
V$ING4_01	ING4	ING4	0.606537	248
V$ELK1_02	Elk-1	ELK1	0.20784	231
V$MAZ_Q6_01	MAZ	MAZ	0.246073	215
V$AP4_Q5	AP-4	TFAP4	0.357839	215
V$YY1_Q6_03	YY1	YY1	0.1231	212
V$ELK1_06	ELK-1	ELK1	0.20784	204
V$AP4_Q6	AP-4	TFAP4	0.357839	197
V$ERF_02	ERF	ERF	0.147416	185
V$GR_01	GR	NR3C1	0.0149042	184
V$CREM_Q6	CREM	CREM	0.199121	178
V$SP2_01	SP2	SP2	0.0968112	165
V$CP2_01	CP2	TFCP2	0.263042	157
V$NEUROD_02	NeuroD	NEUROD1	0.01718	150
V$ZIC1_01	Zic1	ZIC1	0.878687	144
V$GABPBETA_Q3	GABP-beta	GABPB1	0.00593285	142
V$AP4_Q6_01	AP-4	TFAP4	0.357839	139
V$YY1_02	YY1	YY1	0.1231	122
V$CACCCBINDINGFACT	CACCC-binding factor	ZNF148	0.0759542	108
V$ERM_02	Erm	ETV5	0.276381	102
V$MSX1_01	Msx-1	MSX1	0.170961	102
V$ELK1_04	Elk-1	ELK1	0.20784	16
V$ELK1_03	Elk-1	ELK1	0.20784	9

----

hsa-mir-133a-1

Matrix_id	transcription factor	Gene	PCC	Occurrence
V$IK_Q5	Ikaros	IKZF1	0.781805	2498
V$PARP_Q4	PARP	PARP1	0.634708	2446
V$PEA3_Q6	PEA3	ETV4	0.794138	2424
V$AP2REP_01	AP-2rep	KLF12	0.913247	2397
V$P300_01	p300	EP300	0.768497	2358
V$ZIC3_01	Zic3	ZIC3	0.413491	2195
V$SMAD4_Q6_01	Smad4	SMAD4	0.719688	2177
V$CMYB_Q5	c-Myb	MYB	0.278121	2152
V$MYB_Q6	c-Myb	MYB	0.278121	2152
V$CDX2_Q5_02	CDX-2	CDX2	0.884664	2108
V$TBP_Q6	TBP	TBP	0.310926	2108
V$TBX5_02	TBX5	TBX5	0.916782	2092
V$CETS1_Q6	C-ets-1	ETS1	0.576544	2086
V$NFAT4_Q3	NF-AT4	NFATC3	0.540865	2084
V$NR1B2_Q6	NR1B2	RARB	0.80578	2057
V$GATA1_01	GATA-1	GATA1	0.637845	2053
V$TTF1_Q5	TTF-1	NKX2-1	0.232646	2046
V$ETS1_B	c-Ets-1	ETS1	0.576544	2045
V$ETS2_Q6	c-Ets-2	ETS2	0.672813	2009
V$GABPA_Q4	GABP-alpha	GABPA	0.382255	1986
V$YY1_01	YY1	YY1	0.288409	1969
V$SMAD3_Q6_01	Smad3	SMAD3	0.819455	1957
V$MEF2C_Q4	MEF-2C	MEF2C	0.43874	1940
V$Elf5_03	ELF5	ELF5	0.13696	1919
V$GR_Q6	GR	NR3C1	0.650298	1909
V$DLX5_01	dlx5	DLX5	0.132943	1886
V$NANOG_02	Nanog	NANOG	0.917603	1871
V$AML1_Q6	AML1	RUNX1	0.786352	1821
V$IPF1_01	IPF1	PDX1	0.95749	1817
V$SOX5_01	SOX5	SOX5	0.547572	1812
V$HNF4A_Q6_01	HNF-4alpha	HNF4A	0.309515	1809
V$YY1_Q6	YY1	YY1	0.288409	1796
V$GATA1_02	GATA-1	GATA1	0.637845	1774
V$ETS2_B	c-Ets-2	ETS2	0.672813	1770
V$AP4_Q6_02	AP-4	TFAP4	0.612706	1741
V$SPI1_Q5	SPI1	SPI1	0.221832	1733
V$GATA6_01	GATA-6	GATA6	0.0143678	1727
V$GATA2_02	GATA-2	GATA2	0.373527	1727
V$GATA1_06	GATA-1	GATA1	0.637845	1727
V$GATA1_05	GATA-1	GATA1	0.637845	1727
V$IPF1_Q6	IPF1	PDX1	0.95749	1717

V$TBX5_01	TBX5	TBX5	0.916782	1705
V$IPF1_Q4_01	IPF1	PDX1	0.95749	1654
V$CDX2_Q5_01	Cdx-2	CDX2	0.884664	1653
V$SRY_02	SRY	SRY	0.671164	1628
V$CDX2_01	Cdx-2	CDX2	0.884664	1574
V$MAZ_Q6	MAZ	MAZ	0.288488	1564
V$PARP_Q3	PARP	PARP1	0.634708	1550
V$FOXO3A_Q1	FOXO3A	FOXO3	0.599381	1539
V$LRF_Q2	LRF	ZBTB7A	0.881985	1539
V$NFAT2_Q5	NF-AT2	NFATC1	0.938323	1536
V$IRF4_Q6	IRF-4	IRF4	0.914376	1507
V$E2A_Q6	E2A	TCF3	0.793331	1504
V$E12_Q6	E12	TCF3	0.793331	1503
V$HNF3B_Q6	HNF-3beta	FOXA2	0.51566	1501
V$E47_02	E47	TCF3	0.793331	1496
V$MYOD_Q6_01	MyoD	MYOD1	0.83508	1493
V$ING4_01	ING4	ING4	0.0842627	1485
V$GATA1_04	GATA-1	GATA1	0.637845	1482
V$TEL1_02	TEL1	ETV6	0.929123	1449
V$ELK1_02	Elk-1	ELK1	0.727822	1444
V$HNF3A_01	HNF3A	FOXA1	0.455722	1427
V$ETV3_02	ETV3	ETV3	0.616466	1403
V$IPF1_Q4	IPF1	PDX1	0.95749	1387
V$ER71_02	ER71	ETV2	0.597756	1380
V$ZBP89_Q4	ZBP89	ZNF148	0.768483	1311
V$ELK1_06	ELK-1	ELK1	0.727822	1308
V$GFI1_Q6	Gfi1	GFI1	0.399122	1296
V$TCF3_01	TCF-3	TCF7L1	0.706001	1290
V$ARNT_01	Arnt	ARNT	0.840299	1287
V$HNF1_02	HNF-1alpha	HNF1A	0.797891	1271
V$GFI1_Q6_01	Gfi1	GFI1	0.399122	1270
V$FOXJ2_01	FOXJ2	FOXJ2	0.743163	1262
V$GATA2_01	GATA-2	GATA2	0.373527	1250
V$CEBPE_Q6	CEBPE	CEBPE	0.452175	1233
V$SOX10_Q6	SOX10	SOX10	0.421981	1222
V$AP4_Q5	AP-4	TFAP4	0.612706	1216
V$FOXM1_01	FOXM1	FOXM1	0.209204	1208
V$FOXP3_01	FOXP3	FOXP3	0.873354	1198
V$CMYB_01	c-Myb	MYB	0.278121	1183
V$ERF_02	ERF	ERF	0.539968	1166
V$RFX1_02	RFX1	RFX1	0.929992	1152
V$OC2_Q3	OC-2	ONECUT2	0.832897	1125
V$RFX1_01	RFX1	RFX1	0.929992	1084
V$GFI1B_01	Gfi1b	GFI1B	0.932811	1083
V$AML1_01	AML1a	RUNX1	0.786352	1075
V$AML1_Q4	AML1	RUNX1	0.786352	975

V$BCL6_Q3_01	Bcl-6	BCL6	0.914351	962
V$FAC1_01	FAC1	BPTF	0.23267	940
V$E47_01	E47	TCF3	0.793331	931
V$HOXA9_01	hoxa9	HOXA9	0.567369	929
V$CEBPB_02	C/EBPbeta	CEBPB	0.224992	925
V$NKX2B_Q3	NKX2B	NKX2-2	0.268141	922
V$CEBPD_Q6	C/EBPdelta	CEBPD	0.188505	919
V$NKX22_02	NKX2B	NKX2-2	0.268141	906
V$CDX1_01	Cdx-1	CDX1	0.623226	877
V$LHX3b_01	LHX3b	LHX3	0.889692	839
V$PAX8_01	Pax-8	PAX8	0.276466	832
V$CEBPB_Q6	C/EBPbeta	CEBPB	0.224992	792
V$AML2_01	AML2	RUNX3	0.523476	752
V$MSX1_01	Msx-1	MSX1	0.557727	749
V$NCX_02	Ncx	TLX2	0.898704	714
V$CART1_02	CART1	ALX1	0.856029	671
V$CDX2_Q5	Cdx-2	CDX2	0.884664	649
V$HOX13_02	HOXA5	HOXA5	0.027899	630
V$IPF1_06	ipf1	PDX1	0.95749	609
V$HOXB8_01	HOXB8	HOXB8	0.504901	600
V$IPF1_03	IPF1	PDX1	0.95749	574
V$LHX3_01	Lhx3	LHX3	0.889692	449
V$PAX3_B	Pax-3	PAX3	0.670896	403
V$CEBPB_01	C/EBPbeta	CEBPB	0.224992	391
V$HNF1B_01	HNF-1beta	HNF1B	0.445795	377
V$HOXA7_01	HOXA7	HOXA7	0.282853	240
V$RSRFC4_Q2	RSRFC4	MEF2A	0.10651	228
V$RSRFC4_01	RSRFC4	MEF2A	0.10651	197
V$LHX3A_01	Lhx3a	LHX3	0.889692	98
V$AFP1_Q6	AFP1	ZFHX3	0.328241	79

----

hsa-mir-133a-2

Matrix_id	transcription factor	Gene	PCC	Occurrence
V$PUR1_Q4	PUR1	PURA	0.356652	2546
V$IK_Q5	Ikaros	IKZF1	0.781805	2498
V$PEA3_Q6	PEA3	ETV4	0.794138	2424
V$AP2REP_01	AP-2rep	KLF12	0.913247	2397
V$P300_01	p300	EP300	0.768497	2358
V$ZIC3_01	Zic3	ZIC3	0.413491	2195
V$SMAD4_Q6_01	Smad4	SMAD4	0.719688	2177
V$CMYB_Q5	c-Myb	MYB	0.278121	2152
V$MYB_Q6	c-Myb	MYB	0.278121	2152

V$TBX5_02	TBX5	TBX5	0.916782	2092
V$CETS1_Q6	C-ets-1	ETS1	0.576544	2086
V$NFAT4_Q3	NF-AT4	NFATC3	0.540865	2084
V$NR1B2_Q6	NR1B2	RARB	0.80578	2057
V$GATA1_01	GATA-1	GATA1	0.637845	2053
V$TTF1_Q5	TTF-1	NKX2-1	0.232646	2046
V$ETS2_Q6	c-Ets-2	ETS2	0.672813	2009
V$GABPA_Q4	GABP-alpha	GABPA	0.382255	1986
V$YY1_01	YY1	YY1	0.288409	1969
V$SMAD3_Q6_01	Smad3	SMAD3	0.819455	1957
V$MEF2C_Q4	MEF-2C	MEF2C	0.43874	1940
V$Elf5_03	ELF5	ELF5	0.13696	1919
V$GR_Q6	GR	NR3C1	0.650298	1909
V$DLX5_01	dlx5	DLX5	0.132943	1886
V$NANOG_02	Nanog	NANOG	0.917603	1871
V$AML1_Q6	AML1	RUNX1	0.786352	1821
V$IPF1_01	IPF1	PDX1	0.95749	1817
V$HNF4A_Q6_01	HNF-4alpha	HNF4A	0.309515	1809
V$AP4_Q6_02	AP-4	TFAP4	0.612706	1741
V$TBX5_01	TBX5	TBX5	0.916782	1705
V$BEN_01	BEN	GTF2IRD1	0.930847	1694
V$MAZ_Q6	MAZ	MAZ	0.288488	1564
V$ERBETA_Q5	ER-beta	ESR2	0.730676	1552
V$LRF_Q2	LRF	ZBTB7A	0.881985	1539
V$E2A_Q6	E2A	TCF3	0.793331	1504
V$E12_Q6	E12	TCF3	0.793331	1503
V$E47_02	E47	TCF3	0.793331	1496
V$MYOD_Q6_01	MyoD	MYOD1	0.83508	1493
V$SP1_Q6	Sp1	SP1	0.881089	1491
V$ING4_01	ING4	ING4	0.0842627	1485
V$TEL1_02	TEL1	ETV6	0.929123	1449
V$ELK1_02	Elk-1	ELK1	0.727822	1444
V$ETV3_02	ETV3	ETV3	0.616466	1403
V$ER71_02	ER71	ETV2	0.597756	1380
V$MYOGENIN_Q6	myogenin	MYOG	0.797785	1345
V$SP1_01	Sp1	SP1	0.881089	1343
V$SP1_Q6_01	Sp1	SP1	0.881089	1332
V$AP2ALPHA_Q6	AP-2alpha	TFAP2A	0.122318	1317
V$ZBP89_Q4	ZBP89	ZNF148	0.768483	1311
V$ELK1_06	ELK-1	ELK1	0.727822	1308
V$SP1_Q4_01	Sp1	SP1	0.881089	1297
V$TCF3_01	TCF-3	TCF7L1	0.706001	1290
V$SP1_Q2_01	Sp1	SP1	0.881089	1263
V$AP2ALPHA_01	AP-2alpha	TFAP2A	0.122318	1256
V$FKLF_Q5	FKLF	KLF11	0.108569	1237
V$ETV7_01	ETV7	ETV7	0.915056	1224

V$WT1_Q6_01	WT1	WT1	0.114369	1223
V$AP4_Q5	AP-4	TFAP4	0.612706	1216
V$TEF1_Q6_03	TEF-1	TEAD1	0.926341	1193
V$CMYB_01	c-Myb	MYB	0.278121	1183
V$SP1_02	SP1	SP1	0.881089	1180
V$E2A_Q2	E2A	TCF3	0.793331	1178
V$ERF_02	ERF	ERF	0.539968	1166
V$EHF_03	EHF	EHF	0.205449	1138
V$TFIII_Q6	TFII-I	GTF2I	0.685134	1128
V$ZFX_01	Zfx	ZFX	0.884315	1117
V$AML1_01	AML1a	RUNX1	0.786352	1075
V$AP4_Q6	AP-4	TFAP4	0.612706	1063
V$TEF1_Q6	TEF-1	TEAD1	0.926341	1055
V$CREM_Q6	CREM	CREM	0.332275	1054
V$ERR1_Q3	ERR1	ESRRA	0.854699	1042
V$AML1_Q4	AML1	RUNX1	0.786352	975
V$MYOGENIN_Q6_01	myogenin	MYOG	0.797785	971
V$SMAD3_Q6	SMAD3	SMAD3	0.819455	957
V$NEUROD_02	NeuroD	NEUROD1	0.871442	944
V$EGR1_02	EGR-1	EGR1	0.0258495	931
V$GABPBETA_Q3	GABP-beta	GABPB1	0.629243	914
V$AP2GAMMA_01	AP-2gamma	TFAP2C	0.267061	904
V$ZIC1_01	Zic1	ZIC1	0.1822	898
V$MYOD_Q6	MyoD	MYOD1	0.83508	879
V$AP2BETA_Q3	AP-2beta	TFAP2B	0.880094	871
V$CNOT3_01	CNOT3	CNOT3	0.799569	863
V$PAX8_01	Pax-8	PAX8	0.276466	832
V$DBP_Q6_01	DBP	DBP	0.830254	821
V$MEF2A_Q6	mef2A	MEF2A	0.10651	814
V$VDR_Q3	VDR	VDR	0.83353	813
V$DAX1_01	Dax1	NR0B1	0.628736	811
V$ATF1_Q6_01	ATF-1	ATF1	0.484191	786
V$PET1_02	Pet-1	FEV	0.57498	780
V$ERG_03	ERG	ERG	0.790627	773
V$MYOD_Q6_02	MyoD	MYOD1	0.83508	710
V$MATH1_Q2	MATH1	ATOH1	0.691884	710
V$KAISO_01	KAISO	ZBTB33	0.961287	694
V$ATF3_Q6_01	ATF-3	ATF3	0.213496	683
V$MYOD_01	MyoD	MYOD1	0.83508	640
V$CIZ_01	CIZ	ZNF384	0.560262	619
V$MEIS1_01	MEIS1	MEIS1	0.0377805	617
V$ERM_02	Erm	ETV5	0.0796504	613
V$ERG_01	ERG	ERG	0.790627	580
V$P53_02	p53	TP53	0.406827	526
V$BEN_02	BEN	GTF2IRD1	0.930847	463
V$SMAD4_Q6	SMAD4	SMAD4	0.719688	407

V$STAF_02	Staf	ZNF143	0.872827	404
V$EGR2_01	Egr-2	EGR2	0.0895177	365
V$NUR77_Q5	NUR77	NR4A1	0.44718	325
V$ERR3_Q2	ERR3	ESRRG	0.641345	319
V$AMEF2_Q6	aMEF-2	MEF2A	0.10651	301
V$GLI_Q2	GLI	GLI1	0.5996	298
V$ATF6_01	ATF6	ATF6	0.456557	261
V$GLI3_Q5_01	GLI3	GLI3	0.78778	251
V$RSRFC4_Q2	RSRFC4	MEF2A	0.10651	228
V$ERR3_Q2_01	ERR3	ESRRG	0.641345	210
V$GCNF_Q3	GCNF	NR6A1	0.566637	205
V$GLI2_01	GLI2	GLI2	0.906412	198
V$RSRFC4_01	RSRFC4	MEF2A	0.10651	197
V$GLI3_02	GLI3	GLI3	0.78778	189
V$GLI3_01	GLI3	GLI3	0.78778	161
V$EGR1_01	Egr-1	EGR1	0.0258495	149
V$HLF_01	HLF	HLF	0.075662	124
V$E4BP4_01	E4BP4	NFIL3	0.728643	102
V$MEF2A_05	MEF2A	MEF2A	0.10651	65

----

hsa-mir-133b

Matrix_id	transcription factor	Gene	PCC	Occurrence
V$PUR1_Q4	PUR1	PURA	0.367101	2684
V$IK_Q5	Ikaros	IKZF1	0.800265	2632
V$PARP_Q4	PARP	PARP1	0.650879	2574
V$PEA3_Q6	PEA3	ETV4	0.813679	2553
V$AP2REP_01	AP-2rep	KLF12	0.921335	2515
V$P300_01	p300	EP300	0.774408	2484
V$ZIC3_01	Zic3	ZIC3	0.403813	2314
V$SMAD4_Q6_01	Smad4	SMAD4	0.72307	2297
V$CMYB_Q5	c-Myb	MYB	0.284568	2269
V$MYB_Q6	c-Myb	MYB	0.284568	2269
V$TBP_Q6	TBP	TBP	0.318628	2216
V$CDX2_Q5_02	CDX-2	CDX2	0.902202	2216
V$CETS1_Q6	C-ets-1	ETS1	0.564842	2196
V$NFAT4_Q3	NF-AT4	NFATC3	0.555554	2194
V$NR1B2_Q6	NR1B2	RARB	0.814755	2163
V$ETS1_B	c-Ets-1	ETS1	0.564842	2160
V$TTF1_Q5	TTF-1	NKX2-1	0.196866	2160
V$GATA1_01	GATA-1	GATA1	0.617388	2155
V$ETS2_Q6	c-Ets-2	ETS2	0.691759	2117
V$GABPA_Q4	GABP-alpha	GABPA	0.377479	2088

V$YY1_01	YY1	YY1	0.282576	2079
V$SMAD3_Q6_01	Smad3	SMAD3	0.831938	2066
V$MEF2C_Q4	MEF-2C	MEF2C	0.450612	2043
V$GR_Q6	GR	NR3C1	0.660689	2019
V$Elf5_03	ELF5	ELF5	0.138493	2012
V$DLX5_01	dlx5	DLX5	0.154941	1985
V$NANOG_02	Nanog	NANOG	0.931967	1969
V$SPI1_03	SPI1	SPI1	0.213047	1937
V$AML1_Q6	AML1	RUNX1	0.790303	1915
V$IPF1_01	IPF1	PDX1	0.965777	1911
V$HNF4A_Q6_01	HNF-4alpha	HNF4A	0.315531	1909
V$SOX5_01	SOX5	SOX5	0.554934	1902
V$YY1_Q6	YY1	YY1	0.282576	1884
V$ETS2_B	c-Ets-2	ETS2	0.691759	1864
V$AP4_Q6_02	AP-4	TFAP4	0.613354	1832
V$SPI1_Q5	SPI1	SPI1	0.213047	1829
V$GATA6_01	GATA-6	GATA6	0.00447234	1822
V$GATA2_02	GATA-2	GATA2	0.38488	1822
V$GATA1_06	GATA-1	GATA1	0.617388	1822
V$GATA1_05	GATA-1	GATA1	0.617388	1822
V$IPF1_Q6	IPF1	PDX1	0.965777	1806
V$YY1_Q6_02	YY1	YY1	0.282576	1795
V$IPF1_Q4_01	IPF1	PDX1	0.965777	1739
V$SRY_02	SRY	SRY	0.688077	1716
V$NKX32_01	Nkx3-2	NKX3-2	0.876274	1698
V$CDX2_01	Cdx-2	CDX2	0.902202	1654
V$MAZ_Q6	MAZ	MAZ	0.279355	1654
V$ERBETA_Q5	ER-beta	ESR2	0.747344	1640
V$PARP_Q3	PARP	PARP1	0.650879	1629
V$PITX3_Q2	PITX3	PITX3	0.668702	1628
V$LRF_Q2	LRF	ZBTB7A	0.8858	1623
V$NFAT2_Q5	NF-AT2	NFATC1	0.946838	1616
V$IRF4_Q6	IRF-4	IRF4	0.926229	1587
V$HMGIY_01	HMGIY	HMGA1	0.0218946	1557
V$HNF3A_01	HNF3A	FOXA1	0.469678	1492
V$CRX_Q4	Crx	CRX	0.780119	1491
V$IPF1_Q4	IPF1	PDX1	0.965777	1459
V$MYOGENIN_Q6	myogenin	MYOG	0.814275	1414
V$LEF1_Q5	LEF-1	LEF1	0.00741054	1370
V$TCF3_01	TCF-3	TCF7L1	0.707523	1366
V$GFI1_Q6	Gfi1	GFI1	0.4075	1365
V$PITX2_Q2	Pitx2	PITX2	0.794081	1342
V$GFI1_Q6_01	Gfi1	GFI1	0.4075	1341
V$FKLF_Q5	FKLF	KLF11	0.0949936	1309
V$SOX10_Q6	SOX10	SOX10	0.432243	1301
V$CEBPE_Q6	CEBPE	CEBPE	0.432728	1298

V$AP4_Q5	AP-4	TFAP4	0.613354	1274
V$FOXM1_01	FOXM1	FOXM1	0.216338	1264
V$TEF1_Q6_03	TEF-1	TEAD1	0.939755	1253
V$RFX1_02	RFX1	RFX1	0.94301	1208
V$RFX1_01	RFX1	RFX1	0.94301	1146
V$CRX_Q4_01	CRX	CRX	0.780119	1122
V$AP4_Q6	AP-4	TFAP4	0.613354	1114
V$TEF1_Q6	TEF-1	TEAD1	0.939755	1112
V$ELF5_01	ELF5	ELF5	0.138493	1073
V$IRF7_Q3	IRF-7	IRF7	0.14757	1050
V$MYOGENIN_Q6_01	myogenin	MYOG	0.814275	1017
V$FAC1_01	FAC1	BPTF	0.232955	989
V$CEBPB_02	C/EBPbeta	CEBPB	0.231195	969
V$CEBPD_Q6	C/EBPdelta	CEBPD	0.190911	968
V$DBP_Q6_01	DBP	DBP	0.826902	869
V$HOXD9_Q2	Hoxd9	HOXD9	0.757739	866
V$CEBPB_Q6	C/EBPbeta	CEBPB	0.231195	826
V$AP4_Q6_01	AP-4	TFAP4	0.613354	816
V$YY1_02	YY1	YY1	0.282576	759
V$IPF1_02	IPF1	PDX1	0.965777	748
V$HNF6_Q6	HNF6	ONECUT1	0.279227	729
V$HOXB8_01	HOXB8	HOXB8	0.511831	635
V$P53_02	p53	TP53	0.420412	549
V$STAT4_Q5	STAT4	STAT4	0.861611	542
V$EAR2_Q2	EAR2	NR2F6	0.250559	537
V$SOX2_Q6	SOX2	SOX2	0.178983	497
V$RORBETA_Q2	RORBETA	RORB	0.854319	485
V$CREL_01	c-Rel	REL	0.751442	482
V$POU6F1_03	POU6F1	POU6F1	0.641259	458
V$CEBPB_01	C/EBPbeta	CEBPB	0.231195	410
V$POU6F1_02	POU6F1	POU6F1	0.641259	377
V$SPIB_01	SPI-B	SPIB	0.535111	363
V$AMEF2_Q6	aMEF-2	MEF2A	0.106519	316

---

hsa-mir-134

Matrix_id	transcription factor	Gene	PCC	Occurrence
V$PARP_Q4	PARP	PARP1	0.105838	41
V$PEA3_Q6	PEA3	ETV4	0.0375251	38
V$YY1_01	YY1	YY1	0.126463	34
V$GABPA_Q4	GABP-alpha	GABPA	0.072031	32
V$SOX9_Q4	SOX9	SOX9	0.0411387	31
V$YY1_Q6	YY1	YY1	0.126463	30

V$HNF4A_Q6_01	HNF-4alpha	HNF4A	0.114714	30
V$YY1_Q6_02	YY1	YY1	0.126463	30
V$GATA3_01	GATA-3	GATA3	0.0589134	27
V$AP2ALPHA_Q6	AP-2alpha	TFAP2A	0.0802117	24
V$AHR_Q5	AhR	AHR	0.0322404	23
V$PBX1_04	Pbx1	PBX1	0.231858	23
V$CREM_Q6	CREM	CREM	0.603441	20
V$SOX10_Q6	SOX10	SOX10	0.0277026	18
V$PIT1_Q6	Pit-1	POU1F1	0.280054	14
V$CEBPB_02	C/EBPbeta	CEBPB	0.0744071	14
V$GABPBETA_Q3	GABP-beta	GABPB1	0.0540247	13
V$ATF4_Q6	ATF-4	ATF4	0.0153788	12
V$FOXP3_01	FOXP3	FOXP3	0.00295086	11
V$YY1_02	YY1	YY1	0.126463	10
V$MAFK_Q3	MafK	MAFK	0.19942	10
V$OC2_Q3	OC-2	ONECUT2	0.0934791	8
V$ELK1_01	Elk-1	ELK1	0.277918	6

----

hsa-mir-135a-1

Matrix_id	transcription factor	Gene	PCC	Occurrence
V$PUR1_Q4	PUR1	PURA	0.121079	55
V$ELF1_Q6	Elf-1	ELF1	0.214849	53
V$P300_01	p300	EP300	0.153531	52
V$GKLF_Q4	GKLF	KLF4	0.0770016	51
V$SOX9_Q4	SOX9	SOX9	0.171507	50
V$SMAD4_Q6_01	Smad4	SMAD4	0.108653	50
V$ZIC3_01	Zic3	ZIC3	0.308834	49
V$SMAD3_Q6_01	Smad3	SMAD3	0.0169106	46
V$TTF1_Q5	TTF-1	NKX2-1	0.722604	46
V$GR_Q6	GR	NR3C1	0.0954178	45
V$YY1_01	YY1	YY1	0.460208	43
V$SOX9_B1	SOX9	SOX9	0.171507	41
V$PBX1_04	Pbx1	PBX1	0.260413	38
V$TCF3_01	TCF-3	TCF7L1	0.0626344	32
V$ZABC1_01	ZABC1	ZNF217	0.0431223	32
V$YY1_Q6_03	YY1	YY1	0.460208	31
V$HMGIY_01	HMGIY	HMGA1	0.120464	29
V$GR_01	GR	NR3C1	0.0954178	25
V$ZBP89_Q4	ZBP89	ZNF148	0.0857699	25
V$GFI1B_01	Gfi1b	GFI1B	0.0220511	24
V$DBP_Q6_01	DBP	DBP	0.234292	21
V$MSX1_01	Msx-1	MSX1	0.0684242	19

V$OC2_Q3	OC-2	ONECUT2	0.0315688	18
V$SOX2_Q6	SOX2	SOX2	0.0967753	14
V$HOXB8_01	HOXB8	HOXB8	0.166763	12
V$IRF2_01	IRF-2	IRF2	0.0232381	7
V$ZID_01	ZID	ZBTB6	0.0969331	5

----

hsa-mir-135a-2

Matrix_id	transcription factor	Gene	PCC	Occurrence
V$ELF1_Q6	Elf-1	ELF1	0.214849	53
V$P300_01	p300	EP300	0.153531	52
V$SMAD4_Q6_01	Smad4	SMAD4	0.108653	50
V$SOX9_Q4	SOX9	SOX9	0.171507	50
V$ETS1_B	c-Ets-1	ETS1	0.07448	48
V$GATA1_01	GATA-1	GATA1	0.163084	47
V$CETS1_Q6	C-ets-1	ETS1	0.07448	47
V$SMAD3_Q6_01	Smad3	SMAD3	0.0169106	46
V$TTF1_Q5	TTF-1	NKX2-1	0.722604	46
V$GR_Q6	GR	NR3C1	0.0954178	45
V$GABPA_Q4	GABP-alpha	GABPA	0.292074	44
V$SPI1_Q5	SPI1	SPI1	0.0301576	43
V$YY1_01	YY1	YY1	0.460208	43
V$GATA1_02	GATA-1	GATA1	0.163084	42
V$SOX9_B1	SOX9	SOX9	0.171507	41
V$YY1_Q6	YY1	YY1	0.460208	39
V$PBX1_04	Pbx1	PBX1	0.260413	38
V$GATA1_05	GATA-1	GATA1	0.163084	38
V$GATA1_06	GATA-1	GATA1	0.163084	38
V$GATA1_04	GATA-1	GATA1	0.163084	32
V$ZABC1_01	ZABC1	ZNF217	0.0431223	32
V$PBX1_Q3	Pbx1	PBX1	0.260413	31
V$YY1_Q6_03	YY1	YY1	0.460208	31
V$ING4_01	ING4	ING4	0.0312823	30
V$HMGIY_01	HMGIY	HMGA1	0.120464	29
V$HIF1A_Q6	HIF-1alpha	HIF1A	0.26739	27
V$GFI1B_01	Gfi1b	GFI1B	0.0220511	24
V$CREM_Q6	CREM	CREM	0.142725	23
V$DBP_Q6_01	DBP	DBP	0.234292	21
V$FOXO4_02	FOXO4	FOXO4	0.00715137	19
V$MSX1_01	Msx-1	MSX1	0.0684242	19
V$MEF2A_Q6	mef2A	MEF2A	0.150717	19
V$OC2_Q3	OC-2	ONECUT2	0.0315688	18
V$CEBPG_Q6_01	C/EBPgamma	CEBPG	0.0859869	18

V$FOXO4_01	FOXO4	FOXO4	0.00715137	13
V$HOXB8_01	HOXB8	HOXB8	0.166763	12
V$RSRFC4_Q2	RSRFC4	MEF2A	0.150717	7
V$RSRFC4_01	RSRFC4	MEF2A	0.150717	7
V$USF2_Q6	USF2	USF2	0.106333	6
V$MAX_01	Max	MAX	0.221346	6
V$CDP_01	CDP	CUX1	0.13476	1

----

hsa-mir-135b

Matrix_id	transcription factor	Gene	PCC	Occurrence
V$PUR1_Q4	PUR1	PURA	0.114205	215
V$PEA3_Q6	PEA3	ETV4	0.0677754	210
V$PARP_Q4	PARP	PARP1	0.113208	209
V$ELF1_Q6	Elf-1	ELF1	0.18553	206
V$GKLF_Q4	GKLF	KLF4	0.641596	202
V$MAFB_01	MAFB	MAFB	0.313849	198
V$P300_01	p300	EP300	0.371673	192
V$ETS1_B	c-Ets-1	ETS1	0.0765311	191
V$ZIC3_01	Zic3	ZIC3	0.0733606	190
V$CETS1_Q6	C-ets-1	ETS1	0.0765311	189
V$SMAD4_Q6_01	Smad4	SMAD4	0.29145	183
V$SOX9_Q4	SOX9	SOX9	0.0601283	181
V$GABPA_Q4	GABP-alpha	GABPA	0.401935	179
V$TBX5_02	TBX5	TBX5	0.0306843	169
V$GR_Q6	GR	NR3C1	0.0925263	165
V$TTF1_Q5	TTF-1	NKX2-1	0.0420681	164
V$YY1_01	YY1	YY1	0.485084	163
V$AML1_Q6	AML1	RUNX1	0.0109222	151
V$MAZ_Q6	MAZ	MAZ	0.0437113	149
V$SOX5_01	SOX5	SOX5	0.0599985	145
V$SOX9_B1	SOX9	SOX9	0.0601283	145
V$GATA1_02	GATA-1	GATA1	0.0636809	142
V$TBX5_01	TBX5	TBX5	0.0306843	140
V$GATA2_02	GATA-2	GATA2	0.626252	133
V$GATA1_06	GATA-1	GATA1	0.0636809	133
V$GATA1_05	GATA-1	GATA1	0.0636809	133
V$E2A_Q6	E2A	TCF3	0.0465449	128
V$E12_Q6	E12	TCF3	0.0465449	128
V$E47_02	E47	TCF3	0.0465449	126
V$FKLF_Q5	FKLF	KLF11	0.365427	122
V$GATA1_04	GATA-1	GATA1	0.0636809	119
V$GATA3_02	GATA-3	GATA3	0.830837	114

V$YY1_Q6_03	YY1	YY1	0.485084	111
V$E2A_Q2	E2A	TCF3	0.0465449	111
V$GATA3_01	GATA-3	GATA3	0.830837	110
V$MAZ_Q6_01	MAZ	MAZ	0.0437113	110
V$ARNT_01	Arnt	ARNT	0.234802	109
V$ELK1_02	Elk-1	ELK1	0.0417119	105
V$CMAF_01	c-Maf	MAF	0.0660337	105
V$TCF4_01	TCF-4	TCF7L2	0.569759	103
V$GR_01	GR	NR3C1	0.0925263	100
V$TCF4_Q5	TCF-4	TCF7L2	0.569759	96
V$ELK1_06	ELK-1	ELK1	0.0417119	95
V$CEBPA_Q6	C/EBPalpha	CEBPA	0.00256014	92
V$FOXJ2_01	FOXJ2	FOXJ2	0.101755	90
V$AML1_01	AML1a	RUNX1	0.0109222	90
V$GABPBETA_Q3	GABP-beta	GABPB1	0.282764	86
V$ESE1_Q3	ESE-1	ELF3	0.100243	82
V$AML1_Q4	AML1	RUNX1	0.0109222	80
V$ZABC1_01	ZABC1	ZNF217	0.874513	78
V$E47_01	E47	TCF3	0.0465449	71
V$CEBPB_02	C/EBPbeta	CEBPB	0.494546	70
V$ESE1_02	ESE-1	ELF3	0.100243	66
V$STAT3_03	STAT3	STAT3	0.0831841	62
V$ERG_03	ERG	ERG	0.234707	49
V$DEC2_Q2	2-Dec	BHLHE41	0.0610578	45
V$ERM_02	Erm	ETV5	0.59021	35
V$ERG_01	ERG	ERG	0.234707	34
V$SP3_Q3	Sp3	SP3	0.460519	25
V$FLI1_02	Fli-1	FLI1	0.191373	20
V$TCF4_Q5_01	TCF-4	TCF7L2	0.569759	16
V$GRE_C	GR	NR3C1	0.0925263	13

----

hsa-mir-137

Matrix_id	transcription factor	Gene	PCC	Occurrence
V$PUR1_Q4	PUR1	PURA	0.413083	436
V$PARP_Q4	PARP	PARP1	0.430662	401
V$MAFB_01	MAFB	MAFB	0.187755	398
V$ZIC3_01	Zic3	ZIC3	0.140937	397
V$ETS2_Q6	c-Ets-2	ETS2	0.146818	358
V$GABPA_Q4	GABP-alpha	GABPA	0.188799	349
V$TBP_Q6	TBP	TBP	0.0297352	348
V$SOX9_Q4	SOX9	SOX9	0.180876	345
V$GR_Q6	GR	NR3C1	0.0548101	317

V$YY1_01	YY1	YY1	0.172617	315
V$MEF2C_Q4	MEF-2C	MEF2C	0.766878	312
V$HNF4A_Q6_01	HNF-4alpha	HNF4A	0.00545371	302
V$DLX5_01	dlx5	DLX5	0.141943	298
V$SOX9_B1	SOX9	SOX9	0.180876	294
V$YY1_Q6_02	YY1	YY1	0.172617	291
V$YY1_Q6	YY1	YY1	0.172617	289
V$SRY_02	SRY	SRY	0.112293	268
V$ING4_01	ING4	ING4	0.65092	258
V$ELK1_02	Elk-1	ELK1	0.169323	243
V$FOXO3A_Q1	FOXO3A	FOXO3	0.0149623	241
V$PITX3_Q2	PITX3	PITX3	0.143531	237
V$PBX1_04	Pbx1	PBX1	0.2775	237
V$PBX1_Q3	Pbx1	PBX1	0.2775	225
V$YY1_Q6_03	YY1	YY1	0.172617	223
V$GR_01	GR	NR3C1	0.0548101	190
V$IRF8_Q6	IRF-8	IRF8	0.115117	186
V$LHX3b_01	LHX3b	LHX3	0.00877322	147
V$MEF2A_Q6	mef2A	MEF2A	0.598674	140
V$FAC1_01	FAC1	BPTF	0.122007	139
V$PIT1_Q6	Pit-1	POU1F1	0.0295181	133
V$NKX22_02	NKX2B	NKX2-2	0.824319	133
V$DBP_Q6_01	DBP	DBP	0.22362	130
V$NURR1_Q3	NURR1	NR4A2	0.244727	127
V$YY1_02	YY1	YY1	0.172617	124
V$MSX1_01	Msx-1	MSX1	0.17531	113
V$HOX13_02	HOXA5	HOXA5	0.0855414	99
V$LHX3_01	Lhx3	LHX3	0.00877322	74
V$RORBETA_Q2	RORBETA	RORB	0.291374	67
V$CEBPG_Q6	C/EBPgamma	CEBPG	0.140723	62
V$AMEF2_Q6	aMEF-2	MEF2A	0.598674	53
V$RSRFC4_Q2	RSRFC4	MEF2A	0.598674	39
V$POU3F2_02	POU3F2	POU3F2	0.0595025	37
V$RSRFC4_01	RSRFC4	MEF2A	0.598674	36
V$LHX3A_01	Lhx3a	LHX3	0.00877322	21

----

hsa-mir-138-2

Matrix_id	transcription factor	Gene	PCC	Occurrence
V$PUR1_Q4	PUR1	PURA	0.116302	51
V$SMAD4_Q6_01	Smad4	SMAD4	0.078533	50
V$ELF1_Q6	Elf-1	ELF1	0.138143	49
V$MAFB_01	MAFB	MAFB	0.462494	48

V$ZIC3_01	Zic3	ZIC3	0.207155	48
V$P300_01	p300	EP300	0.134196	47
V$GATA1_01	GATA-1	GATA1	0.144224	47
V$ETS1_B	c-Ets-1	ETS1	0.0214665	46
V$SOX9_Q4	SOX9	SOX9	0.0661001	45
V$CETS1_Q6	C-ets-1	ETS1	0.0214665	43
V$TTF1_Q5	TTF-1	NKX2-1	0.758126	43
V$AP4_Q6_02	AP-4	TFAP4	0.274173	41
V$MEF2C_Q4	MEF-2C	MEF2C	0.165845	41
V$GABPA_Q4	GABP-alpha	GABPA	0.325585	41
V$GATA1_02	GATA-1	GATA1	0.144224	40
V$GR_Q6	GR	NR3C1	0.0798799	39
V$SOX9_B1	SOX9	SOX9	0.0661001	38
V$SPI1_Q5	SPI1	SPI1	0.0754872	38
V$PBX1_04	Pbx1	PBX1	0.262137	38
V$YY1_01	YY1	YY1	0.406494	36
V$BEN_01	BEN	GTF2IRD1	0.00396902	35
V$SPI1_03	SPI1	SPI1	0.0754872	34
V$GATA1_05	GATA-1	GATA1	0.144224	33
V$GATA1_06	GATA-1	GATA1	0.144224	33
V$MAZ_Q6	MAZ	MAZ	0.104485	32
V$SOX10_Q6	SOX10	SOX10	0.102967	31
V$TCF4_Q5	TCF-4	TCF7L2	0.27886	30
V$GATA1_04	GATA-1	GATA1	0.144224	30
V$ING4_01	ING4	ING4	0.147685	29
V$AP4_Q5	AP-4	TFAP4	0.274173	29
V$SP1_01	Sp1	SP1	0.0183846	28
V$TCF3_01	TCF-3	TCF7L1	0.0573506	28
V$HMGIY_01	HMGIY	HMGA1	0.0461375	28
V$CMAF_01	c-Maf	MAF	0.04921	27
V$AP4_Q6	AP-4	TFAP4	0.274173	27
V$HIF1A_Q6	HIF-1alpha	HIF1A	0.259716	26
V$TCF4_01	TCF-4	TCF7L2	0.27886	25
V$GFI1B_01	Gfi1b	GFI1B	0.0829057	22
V$DBP_Q6_01	DBP	DBP	0.327356	21
V$GABPBETA_Q3	GABP-beta	GABPB1	0.195189	19
V$GR_01	GR	NR3C1	0.0798799	19
V$NKX2B_Q3	NKX2B	NKX2-2	0.0862461	19
V$NKX22_02	NKX2B	NKX2-2	0.0862461	19
V$POU6F1_03	POU6F1	POU6F1	0.00421631	18
V$CEBPG_Q6_01	C/EBPgamma	CEBPG	0.0492425	16
V$POU6F1_02	POU6F1	POU6F1	0.00421631	15
V$AP4_01	AP-4	TFAP4	0.274173	15
V$CEBPG_Q6	C/EBPgamma	CEBPG	0.0492425	12
V$AMEF2_Q6	aMEF-2	MEF2A	0.293808	9
V$MAX_01	Max	MAX	0.252062	6

V$USF2_Q6	USF2	USF2	0.2393	6
V$MAX_Q6	MAX	MAX	0.252062	3

----

hsa-mir-139

Matrix_id	transcription factor	Gene	PCC	Occurrence
V$PUR1_Q4	PUR1	PURA	0.241085	52
V$PARP_Q4	PARP	PARP1	0.330438	49
V$ZIC3_01	Zic3	ZIC3	0.141595	47
V$GABPA_Q4	GABP-alpha	GABPA	0.129231	47
V$MAFB_01	MAFB	MAFB	0.0721167	45
V$NR1B2_Q6	NR1B2	RARB	0.0797253	44
V$ETS2_Q6	c-Ets-2	ETS2	0.0193522	44
V$SOX9_Q4	SOX9	SOX9	0.116808	42
V$MEF2C_Q4	MEF-2C	MEF2C	0.589447	41
V$SOX9_B1	SOX9	SOX9	0.116808	40
V$ETS2_B	c-Ets-2	ETS2	0.0193522	40
V$AP4_Q6_02	AP-4	TFAP4	0.27257	40
V$YY1_01	YY1	YY1	0.0507061	39
V$YY1_Q6	YY1	YY1	0.0507061	38
V$GATA6_01	GATA-6	GATA6	0.363316	38
V$YY1_Q6_02	YY1	YY1	0.0507061	38
V$HNF4A_Q6_01	HNF-4alpha	HNF4A	0.225703	36
V$PARP_Q3	PARP	PARP1	0.330438	32
V$CRX_Q4	Crx	CRX	0.0240351	29
V$PITX3_Q2	PITX3	PITX3	0.380527	28
V$ING4_01	ING4	ING4	0.464415	28
V$FOXJ2_01	FOXJ2	FOXJ2	0.0545314	28
V$PBX1_Q3	Pbx1	PBX1	0.301013	27
V$HIF1A_Q6	HIF-1alpha	HIF1A	0.383404	26
V$SOX10_Q6	SOX10	SOX10	0.545042	25
V$PIT1_Q6	Pit-1	POU1F1	0.349317	24
V$PBX1_04	Pbx1	PBX1	0.301013	23
V$AP4_Q5	AP-4	TFAP4	0.27257	23
V$SREBP1_Q6	SREBP-1	SREBF1	0.509506	22
V$CP2_01	CP2	TFCP2	0.265714	21
V$CRX_02	Crx	CRX	0.0240351	21
V$AP4_Q6	AP-4	TFAP4	0.27257	20
V$MEF2A_Q6	mef2A	MEF2A	0.395519	17
V$MEIS1_01	MEIS1	MEIS1	0.0576939	17
V$ZIC1_01	Zic1	ZIC1	0.721344	14
V$AP4_Q6_01	AP-4	TFAP4	0.27257	12
V$DEC2_Q2	2-Dec	BHLHE41	0.468235	10

V$SREBP1_02	SREBP-1	SREBF1	0.509506	8
V$HOXA7_01	HOXA7	HOXA7	0.174426	6
V$POU3F2_01	POU3F2	POU3F2	0.0844723	6

hsa-mir-141

Matrix_id	transcription factor	Gene	PCC	Occurrence
V$DLX5_01	dlx5	DLX5	0.235932	31
V$GKLF_Q4	GKLF	KLF4	0.125629	29
V$PITX2_Q2	Pitx2	PITX2	0.163871	22
V$TCF4_01	TCF-4	TCF7L2	0.0204144	21
V$PITX2_01	PITX2	PITX2	0.163871	21
V$AP2ALPHA_Q6	AP-2alpha	TFAP2A	0.376872	17
V$CEBPB_Q6	C/EBPbeta	CEBPB	0.198804	16
V$TCF4_Q5	TCF-4	TCF7L2	0.0204144	16
V$CEBPD_Q6	C/EBPdelta	CEBPD	0.0752722	16
V$AP2ALPHA_01	AP-2alpha	TFAP2A	0.376872	15
V$AHR_Q5	AhR	AHR	0.40502	14
V$AP2GAMMA_01	AP-2gamma	TFAP2C	0.296112	6
V$RNF96_01	RNF96	TRIM28	0.00059289	3
V$XBP1_01	XBP-1	XBP1	0.133744	2

hsa-mir-143

Matrix_id	transcription factor	Gene	PCC	Occurrence
V$PUR1_Q4	PUR1	PURA	0.436133	27
V$GKLF_Q4	GKLF	KLF4	0.237178	25
V$SMAD4_Q6_01	Smad4	SMAD4	0.185539	24
V$ELF1_Q6	Elf-1	ELF1	0.308795	23
V$GATA1_01	GATA-1	GATA1	0.145382	22
V$SOX9_B1	SOX9	SOX9	0.217618	22
V$GR_Q6	GR	NR3C1	0.301896	21
V$ZBP89_Q4	ZBP89	ZNF148	0.0292075	20
V$SRY_02	SRY	SRY	0.061738	18
V$CMAF_01	c-Maf	MAF	0.0934531	16
V$AP2ALPHA_Q6	AP-2alpha	TFAP2A	0.100039	15
V$HIF1A_Q6	HIF-1alpha	HIF1A	0.0628462	11
V$SPIB_03	Spi-B	SPIB	0.0543614	11
V$GR_01	GR	NR3C1	0.301896	11
V$CACCCBINDINGFACT	CACCC-binding factor	ZNF148	0.0292075	11

V$SMAD4_Q6	SMAD4	SMAD4	0.185539	9
V$MAX_01	Max	MAX	0.189647	5
V$CTCF_02	CTCF	CTCF	0.235929	4
V$SREBP1_01	SREBP-1	SREBF1	0.0879663	4
V$CTCF_01	CTCF	CTCF	0.235929	3
V$CMYC_02	c-Myc	MYC	0.105553	3
V$MAX_Q6	MAX	MAX	0.189647	2

hsa-mir-145

Matrix_id	transcription factor	Gene	PCC	Occurrence
V$PUR1_Q4	PUR1	PURA	0.312986	12
V$ELF1_Q6	Elf-1	ELF1	0.186542	12
V$SOX9_B1	SOX9	SOX9	0.23151	11
V$AML1_Q6	AML1	RUNX1	0.0166488	11
V$SMAD4_Q6_01	Smad4	SMAD4	0.158445	11
V$ETS1_B	c-Ets-1	ETS1	0.125711	11
V$GKLF_Q4	GKLF	KLF4	0.323143	10
V$CETS1_Q6	C-ets-1	ETS1	0.125711	10
V$GATA1_01	GATA-1	GATA1	0.302754	10
V$Elf5_03	ELF5	ELF5	0.132948	10
V$GR_Q6	GR	NR3C1	0.17774	9
V$SRY_02	SRY	SRY	0.014206	9
V$AML1_01	AML1a	RUNX1	0.0166488	8
V$AML1_Q4	AML1	RUNX1	0.0166488	7
V$ELF5_01	ELF5	ELF5	0.132948	6
V$SPIB_03	Spi-B	SPIB	0.22183	6
V$GR_01	GR	NR3C1	0.17774	6
V$AP2ALPHA_Q6	AP-2alpha	TFAP2A	0.16682	5
V$TFIII_Q6	TFII-I	GTF2I	0.0220585	4
V$SMAD4_Q6	SMAD4	SMAD4	0.158445	3
V$MAX_01	Max	MAX	0.194661	3
V$SREBP1_01	SREBP-1	SREBF1	0.0726815	2
V$CMYC_02	c-Myc	MYC	0.122316	2
V$MAX_Q6	MAX	MAX	0.194661	1
V$CTCF_02	CTCF	CTCF	0.158113	1
V$CTCF_01	CTCF	CTCF	0.158113	1

hsa-mir-146a

Matrix_id	transcription factor	Gene	PCC	Occurrence

V$IK_Q5	Ikaros	IKZF1	0.0517865	25
V$ELF1_Q6	Elf-1	ELF1	0.102942	23
V$HMGIY_01	HMGIY	HMGA1	0.0255429	23
V$Elf5_03	ELF5	ELF5	0.00601771	15
V$IRF7_Q3	IRF-7	IRF7	0.256817	13
V$IRF8_Q6	IRF-8	IRF8	0.795124	13
V$ELF5_01	ELF5	ELF5	0.00601771	12
V$ZABC1_01	ZABC1	ZNF217	0.135545	11
V$LEF1_Q5	LEF-1	LEF1	0.186332	11
V$HIF1A_Q6	HIF-1alpha	HIF1A	0.0379595	9
V$AML2_Q3	AML2	RUNX3	0.358001	8
V$IRF7_01	IRF-7	IRF7	0.256817	7
V$SPIB_03	Spi-B	SPIB	0.328494	5
V$AML2_Q3_01	AML2	RUNX3	0.358001	4
V$OCAB_Q6	OCA-B	POU2AF1	0.944722	4
V$DEC2_Q2	2-Dec	BHLHE41	0.0138589	3
V$CMYC_02	c-Myc	MYC	0.125937	2
V$CMYC_01	c-Myc	MYC	0.125937	2

hsa-mir-146b

Matrix_id	transcription factor	Gene	PCC	Occurrence
V$MAFB_01	MAFB	MAFB	0.0188025	12
V$AML2_Q3	AML2	RUNX3	0.253896	5
V$EGR1_02	EGR-1	EGR1	0.0164927	5
V$AML2_Q3_01	AML2	RUNX3	0.253896	2
V$SP3_Q3	Sp3	SP3	0.0352425	1

hsa-mir-148a

Matrix_id	transcription factor	Gene	PCC	Occurrence
V$SOX9_Q4	SOX9	SOX9	0.0664914	39
V$ELF1_Q6	Elf-1	ELF1	0.0244017	38
V$MYOD_Q6_01	MyoD	MYOD1	0.0444632	32
V$HNF3B_Q6	HNF-3beta	FOXA2	0.108646	32
V$GABPA_Q4	GABP-alpha	GABPA	0.077227	31
V$SOX9_B1	SOX9	SOX9	0.0664914	30
V$MYOD_Q6	MyoD	MYOD1	0.0444632	24
V$MYOD_01	MyoD	MYOD1	0.0444632	18

---

hsa-mir-148b

Matrix_id	transcription factor	Gene	PCC	Occurrence
V$SOX9_Q4	SOX9	SOX9	0.0243595	38
V$ELF1_Q6	Elf-1	ELF1	0.0744663	36
V$GATA3_02	GATA-3	GATA3	0.178762	33
V$GATA6_01	GATA-6	GATA6	0.00165737	33
V$DLX5_01	dlx5	DLX5	0.15231	32
V$GABPA_Q4	GABP-alpha	GABPA	0.175519	30
V$SOX9_B1	SOX9	SOX9	0.0243595	29
V$PITX3_Q2	PITX3	PITX3	0.103397	29
V$PITX2_Q2	Pitx2	PITX2	0.0415412	22
V$PITX2_01	PITX2	PITX2	0.0415412	21
V$HMGIY_01	HMGIY	HMGA1	0.0210428	21
V$GATA3_01	GATA-3	GATA3	0.178762	19
V$HBP1_Q2	hbp1	HBP1	0.0530861	18
V$FOXO4_02	FOXO4	FOXO4	0.383679	16
V$AHR_Q5	AhR	AHR	0.24791	15
V$HOX13_02	HOXA5	HOXA5	0.0211161	9
V$ESE1_Q3	ESE-1	ELF3	0.251442	8
V$CMYC_02	c-Myc	MYC	0.404479	5
V$CMYC_01	c-Myc	MYC	0.404479	5
V$ESE1_02	ESE-1	ELF3	0.251442	4
V$ERM_02	Erm	ETV5	0.159448	4
V$SREBP1_01	SREBP-1	SREBF1	0.0629834	3

---

hsa-mir-149

Matrix_id	transcription factor	Gene	PCC	Occurrence
V$GKLF_Q4	GKLF	KLF4	0.187456	51
V$PUR1_Q4	PUR1	PURA	0.428863	50
V$PARP_Q4	PARP	PARP1	0.403254	48
V$P300_01	p300	EP300	0.134896	47
V$ELF1_Q6	Elf-1	ELF1	0.0681071	45
V$AHR_Q5	AhR	AHR	0.498189	43
V$AP4_Q6_02	AP-4	TFAP4	0.205621	42
V$SOX9_Q4	SOX9	SOX9	0.145477	41
V$ZIC3_01	Zic3	ZIC3	0.0722261	41
V$DLX5_01	dlx5	DLX5	0.452318	40

V$GABPA_Q4	GABP-alpha	GABPA	0.273087	39
V$ETS2_Q6	c-Ets-2	ETS2	0.0741151	38
V$SOX9_B1	SOX9	SOX9	0.145477	37
V$SP1_Q6	Sp1	SP1	0.0696733	36
V$YY1_Q6	YY1	YY1	0.3566	34
V$SP1_Q6_01	Sp1	SP1	0.0696733	34
V$SP1_01	Sp1	SP1	0.0696733	34
V$ETS2_B	c-Ets-2	ETS2	0.0741151	34
V$YY1_Q6_02	YY1	YY1	0.3566	33
V$SP1_Q4_01	Sp1	SP1	0.0696733	33
V$SP1_Q2_01	Sp1	SP1	0.0696733	33
V$SRY_02	SRY	SRY	0.0778808	32
V$MAZ_Q6	MAZ	MAZ	0.161334	32
V$SP1_02	SP1	SP1	0.0696733	30
V$AP4_Q5	AP-4	TFAP4	0.205621	28
V$PBX1_Q3	Pbx1	PBX1	0.140239	28
V$ARNT_01	Arnt	ARNT	0.0331279	27
V$TFIII_Q6	TFII-I	GTF2I	0.191775	26
V$GATA3_01	GATA-3	GATA3	0.49408	26
V$LEF1_Q5	LEF-1	LEF1	0.0350501	25
V$AP2ALPHA_Q6	AP-2alpha	TFAP2A	0.471457	25
V$MAZ_Q6_01	MAZ	MAZ	0.161334	24
V$AP4_Q6	AP-4	TFAP4	0.205621	24
V$GFI1_Q6_01	Gfi1	GFI1	0.00013302	24
V$SP2_01	SP2	SP2	0.15121	24
V$IRF8_Q6	IRF-8	IRF8	0.0220163	23
V$GFI1_Q6	Gfi1	GFI1	0.00013302	23
V$AP2ALPHA_01	AP-2alpha	TFAP2A	0.471457	22
V$AP4_Q6_01	AP-4	TFAP4	0.205621	21
V$AP2GAMMA_01	AP-2gamma	TFAP2C	0.3899	20
V$CP2_01	CP2	TFCP2	0.570772	20
V$GATA2_01	GATA-2	GATA2	0.27693	20
V$TCF4_Q5	TCF-4	TCF7L2	0.158702	19
V$MSX1_01	Msx-1	MSX1	0.10026	17
V$NEUROD_02	NeuroD	NEUROD1	0.00970685	16
V$HOXB8_01	HOXB8	HOXB8	0.197998	15
V$RNF96_01	RNF96	TRIM28	0.225263	15
V$TCF4_01	TCF-4	TCF7L2	0.158702	15
V$ERM_02	Erm	ETV5	0.49386	14
V$DEC2_Q2	2-Dec	BHLHE41	0.488862	13
V$AP2ALPHA_02	AP-2alphaA	TFAP2A	0.471457	10
V$SREBP1_02	SREBP-1	SREBF1	0.185648	9
V$SP3_Q3	Sp3	SP3	0.133184	8
V$EGR2_01	Egr-2	EGR2	0.159986	8
V$SRF_Q6	SRF	SRF	0.180292	4
V$SRF_C	SRF	SRF	0.180292	4

V$TGIF_01	TGIF	TGIF1	0.238161	4
V$SRF_Q4	SRF	SRF	0.180292	3
V$SRF_Q5_02	SRF	SRF	0.180292	3
V$BRF1_01	BRF-1	BRF1	0.139457	2
V$CDP_02	CDP	CUX1	0.288346	2

hsa-mir-150

Matrix_id	transcription factor	Gene	PCC	Occurrence
V$IK_Q5	Ikaros	IKZF1	0.057942	20
V$LEF1_Q5	LEF-1	LEF1	0.122083	13
V$IRF7_Q3	IRF-7	IRF7	0.243398	12
V$ING4_01	ING4	ING4	0.0303228	10
V$AML2_Q3	AML2	RUNX3	0.292784	7
V$STAT1_05	STAT1	STAT1	0.640702	6
V$AML2_Q3_01	AML2	RUNX3	0.292784	3

hsa-mir-151a

Matrix_id	transcription factor	Gene	PCC	Occurrence
V$P300_01	p300	EP300	0.0303532	2
V$PARP_Q4	PARP	PARP1	0.219812	2
V$AHR_Q5	AhR	AHR	0.30269	2
V$YY1_Q6	YY1	YY1	0.25437	2
V$GR_01	GR	NR3C1	0.00377341	2
V$TBP_Q6	TBP	TBP	0.088165	2
V$YY1_Q6_02	YY1	YY1	0.25437	2
V$MAFB_01	MAFB	MAFB	0.121702	2
V$DLX5_01	dlx5	DLX5	0.249212	2
V$HMGIY_01	HMGIY	HMGA1	0.00700842	2
V$PUR1_Q4	PUR1	PURA	0.42702	2
V$GKLF_Q4	GKLF	KLF4	0.0749585	2
V$AP4_Q6_02	AP-4	TFAP4	0.282611	2
V$CEBPB_Q6	C/EBPbeta	CEBPB	0.00767225	2
V$CEBPG_Q6	C/EBPgamma	CEBPG	0.359308	2
V$GR_Q6	GR	NR3C1	0.00377341	2
V$GATA3_02	GATA-3	GATA3	0.165287	2
V$MSX1_01	Msx-1	MSX1	0.116278	2
V$SOX9_B1	SOX9	SOX9	0.330123	2
V$ZIC1_01	Zic1	ZIC1	0.654562	2

V$CP2_01	CP2	TFCP2	0.323954	2
V$ZIC3_01	Zic3	ZIC3	0.191712	2
V$GATA6_01	GATA-6	GATA6	0.0167044	2
V$GATA2_02	GATA-2	GATA2	0.139018	2
V$CDP_02	CDP	CUX1	0.183439	1
V$PITX3_Q2	PITX3	PITX3	0.090475	1
V$AP2ALPHA_Q6	AP-2alpha	TFAP2A	0.252345	1
V$YY1_01	YY1	YY1	0.25437	1
V$CEBPB_02	C/EBPbeta	CEBPB	0.00767225	1
V$AP4_Q5	AP-4	TFAP4	0.282611	1
V$AP4_Q6	AP-4	TFAP4	0.282611	1
V$SOX9_Q4	SOX9	SOX9	0.330123	1
V$AP2ALPHA_01	AP-2alpha	TFAP2A	0.252345	1
V$AP4_Q6_01	AP-4	TFAP4	0.282611	1
V$SREBP1_Q6	SREBP-1	SREBF1	0.277848	1
V$AP2GAMMA_01	AP-2gamma	TFAP2C	0.179622	1
V$TFIII_Q6	TFII-I	GTF2I	0.085692	1

----

hsa-mir-152

Matrix_id	transcription factor	Gene	PCC	Occurrence
V$CETS1_Q6	C-ets-1	ETS1	0.320488	3
V$GFI1_Q6_01	Gfi1	GFI1	0.19076	3
V$PEA3_Q6	PEA3	ETV4	0.434564	3
V$HNF1_02	HNF-1alpha	HNF1A	0.185865	3
V$FOXM1_01	FOXM1	FOXM1	0.0731977	3
V$SOX9_Q4	SOX9	SOX9	0.266525	3
V$GATA6_01	GATA-6	GATA6	0.357952	3
V$ZIC3_01	Zic3	ZIC3	0.181275	3
V$YY1_Q6_02	YY1	YY1	0.551921	3
V$EGR1_02	EGR-1	EGR1	0.260808	3
V$ETS2_Q6	c-Ets-2	ETS2	0.0998072	3
V$GKLF_Q4	GKLF	KLF4	0.324423	3
V$TBP_Q6	TBP	TBP	0.25147	3
V$GFI1_Q6	Gfi1	GFI1	0.19076	3
V$NFAT4_Q3	NF-AT4	NFATC3	0.188429	3
V$ERBETA_Q5	ER-beta	ESR2	0.255675	3
V$AP4_Q6_02	AP-4	TFAP4	0.336967	3
V$AML1_Q6	AML1	RUNX1	0.485305	3
V$IK_Q5	Ikaros	IKZF1	0.201536	3
V$ELF1_Q6	Elf-1	ELF1	0.105662	3
V$GATA3_02	GATA-3	GATA3	0.178223	3
V$GATA2_02	GATA-2	GATA2	0.441808	3

V$PUR1_Q4	PUR1	PURA	0.359639	3
V$Elf5_03	ELF5	ELF5	0.334434	3
V$MYOGENIN_Q6_01	myogenin	MYOG	0.317573	3
V$NR1B2_Q6	NR1B2	RARB	0.436334	3
V$CDX2_Q5_02	CDX-2	CDX2	0.282579	3
V$P300_01	p300	EP300	0.469496	3
V$PBX1_Q3	Pbx1	PBX1	0.254089	3
V$ETS1_B	c-Ets-1	ETS1	0.320488	3
V$ETS2_B	c-Ets-2	ETS2	0.0998072	3
V$GATA1_05	GATA-1	GATA1	0.475638	3
V$GABPA_Q4	GABP-alpha	GABPA	0.155834	3
V$AP2REP_01	AP-2rep	KLF12	0.398929	3
V$PARP_Q4	PARP	PARP1	0.218495	3
V$GATA1_06	GATA-1	GATA1	0.475638	3
V$PITX1_01	Pitx1	PITX1	0.362019	2
V$PITX3_Q2	PITX3	PITX3	0.354173	2
V$SOX10_Q6	SOX10	SOX10	0.0826294	2
V$CDX2_Q5_01	Cdx-2	CDX2	0.282579	2
V$CRX_Q4	Crx	CRX	0.29134	2
V$CDX2_01	Cdx-2	CDX2	0.282579	2
V$PITX2_01	PITX2	PITX2	0.407729	2
V$PBX1_04	Pbx1	PBX1	0.254089	2
V$CMYB_Q5	c-Myb	MYB	0.0226967	2
V$ZABC1_01	ZABC1	ZNF217	0.248275	2
V$IPF1_Q6	IPF1	PDX1	0.36999	2
V$HNF3A_01	HNF3A	FOXA1	0.441391	2
V$NEUROD_02	NeuroD	NEUROD1	0.289105	2
V$HOXD9_Q2	Hoxd9	HOXD9	0.387976	2
V$DLX5_01	dlx5	DLX5	0.242626	2
V$IPF1_01	IPF1	PDX1	0.36999	2
V$CRX_02	Crx	CRX	0.29134	2
V$PARP_Q3	PARP	PARP1	0.218495	2
V$CMAF_01	c-Maf	MAF	0.242116	2
V$GABPBETA_Q3	GABP-beta	GABPB1	0.664999	2
V$SOX9_B1	SOX9	SOX9	0.266525	2
V$MSX1_01	Msx-1	MSX1	0.188119	2
V$GATA3_03	GATA-3	GATA3	0.178223	2
V$SRY_02	SRY	SRY	0.301153	2
V$SMAD3_Q6_01	Smad3	SMAD3	0.304218	2
V$GR_Q6	GR	NR3C1	0.480127	2
V$MYB_Q6	c-Myb	MYB	0.0226967	2
V$GATA1_04	GATA-1	GATA1	0.475638	2
V$GATA1_02	GATA-1	GATA1	0.475638	2
V$YY1_01	YY1	YY1	0.551921	2
V$CREL_01	c-Rel	REL	0.185866	2
V$SOX5_01	SOX5	SOX5	0.289029	2

V$MAFK_Q3	MafK	MAFK	0.411822	2
V$PITX2_Q2	Pitx2	PITX2	0.407729	2
V$SREBP1_Q6	SREBP-1	SREBF1	0.389292	2
V$PIT1_Q6	Pit-1	POU1F1	0.264037	2
V$SMAD4_Q6	SMAD4	SMAD4	0.59101	2
V$YY1_Q6_03	YY1	YY1	0.551921	2
V$CACCCBINDINGFACT	CACCC-binding factor	ZNF148	0.388971	2
V$IRF4_Q6	IRF-4	IRF4	0.411362	2
V$MAZ_Q6	MAZ	MAZ	0.205731	2
V$IPF1_Q4_01	IPF1	PDX1	0.36999	2
V$SMAD4_Q6_01	Smad4	SMAD4	0.59101	2
V$ARNT_01	Arnt	ARNT	0.414906	1
V$EAR2_Q2	EAR2	NR2F6	0.134678	1
V$NFAT2_Q5	NF-AT2	NFATC1	0.450634	1
V$MITF_Q6	MITF	MITF	0.293741	1
V$HTF4_Q2	HTF4	TCF12	0.387049	1
V$AML2_01	AML2	RUNX3	0.150722	1
V$HIF1A_Q6	HIF-1alpha	HIF1A	0.509226	1
V$MAX_Q6	MAX	MAX	0.189309	1
V$HNF4A_Q6_01	HNF-4alpha	HNF4A	0.146194	1
V$CRX_Q4_01	CRX	CRX	0.29134	1
V$HBP1_Q2	hbp1	HBP1	0.384163	1
V$E2A_Q2	E2A	TCF3	0.369155	1
V$CDX2_Q5	Cdx-2	CDX2	0.282579	1
V$USF2_Q6	USF2	USF2	0.380066	1
V$MYOGENIN_Q6	myogenin	MYOG	0.317573	1
V$E12_Q6	E12	TCF3	0.369155	1
V$ARNT_02	Arnt	ARNT	0.414906	1
V$MYOD_01	MyoD	MYOD1	0.142649	1
V$FOXJ2_01	FOXJ2	FOXJ2	0.590845	1
V$GATA2_03	GATA-2	GATA2	0.441808	1
V$AML1_01	AML1a	RUNX1	0.485305	1
V$MYOD_Q6	MyoD	MYOD1	0.142649	1
V$MAX_01	Max	MAX	0.189309	1
V$E47_02	E47	TCF3	0.369155	1
V$NMYC_01	N-Myc	MYCN	0.292955	1
V$MYOD_Q6_01	MyoD	MYOD1	0.142649	1
V$GR_01	GR	NR3C1	0.480127	1
V$AML1_Q4	AML1	RUNX1	0.485305	1
V$HMGIY_01	HMGIY	HMGA1	0.12409	1
V$TCF3_01	TCF-3	TCF7L1	0.321965	1
V$CDX1_01	Cdx-1	CDX1	0.258243	1
V$OCT2_01	2-Oct	POU2F2	0.393043	1
V$HOXA9_01	hoxa9	HOXA9	0.324687	1
V$NANOG_02	Nanog	NANOG	0.344827	1
V$IPF1_02	IPF1	PDX1	0.36999	1

V$ELF5_01	ELF5	ELF5	0.334434	1
V$CMYC_02	c-Myc	MYC	0.103898	1
V$GFI1B_01	Gfi1b	GFI1B	0.310299	1
V$TBX5_02	TBX5	TBX5	0.369802	1
V$TBX5_01	TBX5	TBX5	0.369802	1
V$E2A_Q6	E2A	TCF3	0.369155	1
V$E47_01	E47	TCF3	0.369155	1

----

hsa-mir-153-1

Matrix_id	transcription factor	Gene	PCC	Occurrence
V$PUR1_Q4	PUR1	PURA	0.340461	393
V$MAFB_01	MAFB	MAFB	0.0504199	365
V$PARP_Q4	PARP	PARP1	0.369141	363
V$SOX9_Q4	SOX9	SOX9	0.351227	313
V$TBP_Q6	TBP	TBP	0.0125622	313
V$YY1_01	YY1	YY1	0.0131308	287
V$MEF2C_Q4	MEF-2C	MEF2C	0.761938	278
V$DLX5_01	dlx5	DLX5	0.0303345	268
V$YY1_Q6	YY1	YY1	0.0131308	264
V$ETS2_B	c-Ets-2	ETS2	0.145157	264
V$SOX9_B1	SOX9	SOX9	0.351227	261
V$SRY_02	SRY	SRY	0.148964	240
V$PARP_Q3	PARP	PARP1	0.369141	240
V$TFIII_Q6	TFII-I	GTF2I	0.140021	232
V$HNF3B_Q6	HNF-3beta	FOXA2	0.024515	227
V$FOXO3A_Q1	FOXO3A	FOXO3	0.00467824	217
V$PBX1_04	Pbx1	PBX1	0.189042	215
V$PITX3_Q2	PITX3	PITX3	0.0598077	213
V$HIF1A_Q6	HIF-1alpha	HIF1A	0.0258937	203
V$YY1_Q6_03	YY1	YY1	0.0131308	201
V$IRF8_Q6	IRF-8	IRF8	0.0641219	178
V$SOX10_Q6	SOX10	SOX10	0.713636	166
V$NEUROD_02	NeuroD	NEUROD1	0.0689893	142
V$E2F1_Q3	E2F-1	E2F1	0.0999082	140
V$CEBPG_Q6_01	C/EBPgamma	CEBPG	0.140519	129
V$NKX2B_Q3	NKX2B	NKX2-2	0.837286	126
V$NKX22_02	NKX2B	NKX2-2	0.837286	123
V$MEF2A_Q6	mef2A	MEF2A	0.629138	122
V$E2F1_Q6	E2F-1	E2F1	0.0999082	110
V$MSX1_01	Msx-1	MSX1	0.121858	102
V$SOX2_Q6	SOX2	SOX2	0.491028	66
V$CEBPG_Q6	C/EBPgamma	CEBPG	0.140519	56

----

hsa-mir-153-2

Matrix_id	transcription factor	Gene	PCC	Occurrence
V$PUR1_Q4	PUR1	PURA	0.340461	393
V$MAFB_01	MAFB	MAFB	0.0504199	365
V$PARP_Q4	PARP	PARP1	0.369141	363
V$ZIC3_01	Zic3	ZIC3	0.184233	357
V$ETS2_Q6	c-Ets-2	ETS2	0.145157	324
V$GABPA_Q4	GABP-alpha	GABPA	0.106234	315
V$SOX9_Q4	SOX9	SOX9	0.351227	313
V$TBP_Q6	TBP	TBP	0.0125622	313
V$MAZ_Q6	MAZ	MAZ	0.171243	296
V$YY1_01	YY1	YY1	0.0131308	287
V$AP4_Q6_02	AP-4	TFAP4	0.397559	286
V$MEF2C_Q4	MEF-2C	MEF2C	0.761938	278
V$DLX5_01	dlx5	DLX5	0.0303345	268
V$ETS2_B	c-Ets-2	ETS2	0.145157	264
V$SOX9_B1	SOX9	SOX9	0.351227	261
V$SRY_02	SRY	SRY	0.148964	240
V$PARP_Q3	PARP	PARP1	0.369141	240
V$ZBP89_Q4	ZBP89	ZNF148	0.074935	237
V$TFIII_Q6	TFII-I	GTF2I	0.140021	232
V$ING4_01	ING4	ING4	0.555058	229
V$HNF3B_Q6	HNF-3beta	FOXA2	0.024515	227
V$FOXO3A_Q1	FOXO3A	FOXO3	0.00467824	217
V$PITX3_Q2	PITX3	PITX3	0.0598077	213
V$MAZ_Q6_01	MAZ	MAZ	0.171243	207
V$HIF1A_Q6	HIF-1alpha	HIF1A	0.0258937	203
V$AP4_Q5	AP-4	TFAP4	0.397559	200
V$CREM_Q6	CREM	CREM	0.0211417	173
V$EGR1_02	EGR-1	EGR1	0.0343084	172
V$SOX10_Q6	SOX10	SOX10	0.713636	166
V$ZIC1_01	Zic1	ZIC1	0.912365	139
V$NKX2B_Q3	NKX2B	NKX2-2	0.837286	126
V$NKX22_02	NKX2B	NKX2-2	0.837286	123
V$MEF2A_Q6	mef2A	MEF2A	0.629138	122
V$ELK1_01	Elk-1	ELK1	0.0758934	102
V$ATF4_Q6	ATF-4	ATF4	0.0646993	101
V$USF2_Q6	USF2	USF2	0.244189	76
V$MAX_01	Max	MAX	0.424959	74
V$SOX2_Q6	SOX2	SOX2	0.491028	66
V$CEBPG_Q6	C/EBPgamma	CEBPG	0.140519	56

----

hsa-mir-154

Matrix_id	transcription factor	Gene	PCC	Occurrence
V$MAFB_01	MAFB	MAFB	0.171488	26
V$GKLF_Q4	GKLF	KLF4	0.382525	26
V$PUR1_Q4	PUR1	PURA	0.0511859	26
V$ELF1_Q6	Elf-1	ELF1	0.0648347	25
V$PARP_Q4	PARP	PARP1	0.0927483	25
V$PEA3_Q6	PEA3	ETV4	0.109329	24
V$P300_01	p300	EP300	0.221685	23
V$SMAD4_Q6_01	Smad4	SMAD4	0.151727	22
V$GABPA_Q4	GABP-alpha	GABPA	0.274004	20
V$GR_Q6	GR	NR3C1	0.0439713	19
V$GATA3_01	GATA-3	GATA3	0.648057	18
V$YY1_01	YY1	YY1	0.322823	18
V$ERBETA_Q5	ER-beta	ESR2	0.0020106	17
V$GATA2_01	GATA-2	GATA2	0.438894	17
V$AP2ALPHA_Q6	AP-2alpha	TFAP2A	0.685194	16
V$YY1_Q6	YY1	YY1	0.322823	16
V$YY1_Q6_02	YY1	YY1	0.322823	15
V$AHR_Q5	AhR	AHR	0.65753	15
V$FOXP3_01	FOXP3	FOXP3	0.0177845	14
V$PBX1_04	Pbx1	PBX1	0.106296	14
V$ATF3_Q6_01	ATF-3	ATF3	0.352309	12
V$CREM_Q6	CREM	CREM	0.476714	12
V$SMAD4_Q6	SMAD4	SMAD4	0.151727	11
V$PIT1_Q6	Pit-1	POU1F1	0.123327	10
V$GABPBETA_Q3	GABP-beta	GABPB1	0.184879	10
V$GR_01	GR	NR3C1	0.0439713	10
V$YY1_02	YY1	YY1	0.322823	9
V$ATF4_Q6	ATF-4	ATF4	0.0622201	9
V$OC2_Q3	OC-2	ONECUT2	0.0558151	8
V$TCF4_01	TCF-4	TCF7L2	0.279164	7
V$CEBPB_02	C/EBPbeta	CEBPB	0.497467	7
V$MAFK_Q3	MafK	MAFK	0.358422	6
V$ELK1_01	Elk-1	ELK1	0.178428	5

----

hsa-mir-155

Matrix_id	transcription factor	Gene	PCC	Occurrence

V$IK_Q5	Ikaros	IKZF1	0.0558765	23
V$GFI1_Q6_01	Gfi1	GFI1	0.0131647	17
V$SPI1_03	SPI1	SPI1	0.0276305	16
V$GFI1_Q6	Gfi1	GFI1	0.0131647	16
V$SPI1_Q5	SPI1	SPI1	0.0276305	15
V$IRF7_Q3	IRF-7	IRF7	0.305614	13
V$LEF1_Q5	LEF-1	LEF1	0.197306	13
V$STAT1_05	STAT1	STAT1	0.679087	8
V$HIF1A_Q6	HIF-1alpha	HIF1A	0.0201737	8
V$SPIB_03	Spi-B	SPIB	0.32805	5
V$CTCF_02	CTCF	CTCF	0.00122013	2
V$SP3_Q3	Sp3	SP3	0.136373	2

hsa-mir-15a

Matrix_id	transcription factor	Gene	PCC	Occurrence
V$CDX2_Q5_02	CDX-2	CDX2	0.290434	2
V$HNF1B_01	HNF-1beta	HNF1B	0.0320753	2
V$PARP_Q3	PARP	PARP1	0.447241	2
V$ETS2_Q6	c-Ets-2	ETS2	0.382401	2
V$NKX32_01	Nkx3-2	NKX3-2	0.276649	2
V$IK_Q5	Ikaros	IKZF1	0.59559	2
V$IPF1_Q4_01	IPF1	PDX1	0.266586	2
V$TBP_Q6	TBP	TBP	0.0817948	2
V$GR_01	GR	NR3C1	0.340357	2
V$AHR_Q5	AhR	AHR	0.0432753	2
V$ELF1_Q6	Elf-1	ELF1	0.238781	2
V$IPF1_Q6	IPF1	PDX1	0.266586	2
V$HNF1_02	HNF-1alpha	HNF1A	0.0424573	2
V$SPI1_Q5	SPI1	SPI1	0.180726	2
V$PARP_Q4	PARP	PARP1	0.447241	2
V$LEF1_Q5	LEF-1	LEF1	0.561067	2
V$NFAT4_Q3	NF-AT4	NFATC3	0.65541	2
V$CETS1_Q6	C-ets-1	ETS1	0.131715	2
V$CMYB_Q5	c-Myb	MYB	0.54124	2
V$PUR1_Q4	PUR1	PURA	0.155779	2
V$HBP1_Q2	hbp1	HBP1	0.239023	2
V$OC2_Q3	OC-2	ONECUT2	0.193749	2
V$NKX32_02	Nkx3-2	NKX3-2	0.276649	2
V$PEA3_Q6	PEA3	ETV4	0.267836	2
V$AP2REP_01	AP-2rep	KLF12	0.335061	2
V$GATA1_01	GATA-1	GATA1	0.0703413	2

V$GATA1_05	GATA-1	GATA1	0.0703413	2
V$GATA1_06	GATA-1	GATA1	0.0703413	2
V$GATA2_02	GATA-2	GATA2	0.256038	2
V$GATA3_02	GATA-3	GATA3	0.277486	2
V$ETS1_B	c-Ets-1	ETS1	0.131715	2
V$IPF1_Q4	IPF1	PDX1	0.266586	2
V$ETS2_B	c-Ets-2	ETS2	0.382401	2
V$FAC1_01	FAC1	BPTF	0.00474533	2
V$MYB_Q6	c-Myb	MYB	0.54124	2
V$GR_Q6	GR	NR3C1	0.340357	2
V$CMYB_01	c-Myb	MYB	0.54124	2
V$YY1_01	YY1	YY1	0.308855	2
V$P300_01	p300	EP300	0.252043	2
V$SRY_02	SRY	SRY	0.0738644	1
V$PITX1_01	Pitx1	PITX1	0.353794	1
V$PITX3_Q2	PITX3	PITX3	0.292916	1
V$EAR2_Q2	EAR2	NR2F6	0.120794	1
V$GATA1_04	GATA-1	GATA1	0.0703413	1
V$CRX_Q4_01	CRX	CRX	0.143287	1
V$FOXP3_01	FOXP3	FOXP3	0.330726	1
V$HMGIY_01	HMGIY	HMGA1	0.249707	1
V$AML1_Q4	AML1	RUNX1	0.307339	1
V$IRF8_Q6	IRF-8	IRF8	0.535904	1
V$AML2_01	AML2	RUNX3	0.455899	1
V$CDX2_Q5_01	Cdx-2	CDX2	0.290434	1
V$NKX2B_Q3	NKX2B	NKX2-2	0.0533756	1
V$SPI1_03	SPI1	SPI1	0.180726	1
V$SPIB_03	Spi-B	SPIB	0.320968	1
V$MEF2C_Q4	MEF-2C	MEF2C	0.150013	1
V$GATA1_02	GATA-1	GATA1	0.0703413	1
V$HNF3B_Q6	HNF-3beta	FOXA2	0.208908	1
V$GFI1_Q6_01	Gfi1	GFI1	0.621768	1
V$LHX3b_01	LHX3b	LHX3	0.266134	1
V$GATA2_01	GATA-2	GATA2	0.256038	1
V$IRF4_Q6	IRF-4	IRF4	0.310614	1
V$GABPBETA_Q3	GABP-beta	GABPB1	0.156605	1
V$GATA3_01	GATA-3	GATA3	0.277486	1
V$HOX13_02	HOXA5	HOXA5	0.281354	1
V$CDX2_01	Cdx-2	CDX2	0.290434	1
V$PITX2_01	PITX2	PITX2	0.31012	1
V$KAISO_01	KAISO	ZBTB33	0.347212	1
V$FOXJ2_01	FOXJ2	FOXJ2	0.26646	1
V$PIT1_Q6	Pit-1	POU1F1	0.00787537	1
V$AML1_Q6	AML1	RUNX1	0.307339	1
V$POU3F2_02	POU3F2	POU3F2	0.342984	1
V$POU6F1_01	POU6F1	POU6F1	0.109321	1

V$HNF6_Q6	HNF6	ONECUT1	0.0116368	1
V$FOXM1_01	FOXM1	FOXM1	0.261817	1
V$CRX_Q4	Crx	CRX	0.143287	1
V$CEBPD_Q6	C/EBPdelta	CEBPD	0.201812	1
V$AFP1_Q6	AFP1	ZFHX3	0.393896	1
V$PITX2_Q2	Pitx2	PITX2	0.31012	1
V$FOXO4_02	FOXO4	FOXO4	0.0554914	1
V$SOX10_Q6	SOX10	SOX10	0.070614	1
V$FOXO3A_Q1	FOXO3A	FOXO3	0.44777	1
V$GATA2_03	GATA-2	GATA2	0.256038	1
V$IPF1_06	ipf1	PDX1	0.266586	1
V$CRX_02	Crx	CRX	0.143287	1
V$IPF1_01	IPF1	PDX1	0.266586	1
V$DLX5_01	dlx5	DLX5	0.0411586	1
V$AML1_01	AML1a	RUNX1	0.307339	1
V$NKX22_02	NKX2B	NKX2-2	0.0533756	1
V$CART1_02	CART1	ALX1	0.267248	1
V$CDP_04	CDP	CUX1	0.125381	1
V$NURR1_Q3	NURR1	NR4A2	0.0492048	1
V$HNF3A_01	HNF3A	FOXA1	0.358573	1
V$STAT1_05	STAT1	STAT1	0.462315	1
V$NANOG_02	Nanog	NANOG	0.291514	1
V$IPF1_03	IPF1	PDX1	0.266586	1
V$FOXO4_01	FOXO4	FOXO4	0.0554914	1

---

hsa-mir-15b

Matrix_id	transcription factor	Gene	PCC	Occurrence
V$ELF1_Q6	Elf-1	ELF1	0.329838	7
V$IK_Q5	Ikaros	IKZF1	0.141912	7
V$GKLF_Q4	GKLF	KLF4	0.155463	7
V$NFAT4_Q3	NF-AT4	NFATC3	0.499334	7
V$PARP_Q4	PARP	PARP1	0.0279517	7
V$YY1_01	YY1	YY1	0.151964	6
V$CMYB_Q5	c-Myb	MYB	0.567284	6
V$DLX5_01	dlx5	DLX5	0.0725963	6
V$MYB_Q6	c-Myb	MYB	0.567284	6
V$IRF8_Q6	IRF-8	IRF8	0.31015	6
V$TBP_Q6	TBP	TBP	0.235425	6
V$CREM_Q6	CREM	CREM	0.0819696	5
V$HMGIY_01	HMGIY	HMGA1	0.700652	5
V$YY1_Q6_02	YY1	YY1	0.151964	5
V$YY1_Q6	YY1	YY1	0.151964	5

V$AML1_Q6	AML1	RUNX1	0.0470122	5
V$YY1_02	YY1	YY1	0.151964	5
V$SP2_01	SP2	SP2	0.0324013	5
V$CEBPD_Q6	C/EBPdelta	CEBPD	0.0421394	4
V$AML1_Q4	AML1	RUNX1	0.0470122	4
V$YY1_03	YY1	YY1	0.151964	4
V$GATA3_01	GATA-3	GATA3	0.300141	4
V$AML1_01	AML1a	RUNX1	0.0470122	4
V$GFI1_Q6	Gfi1	GFI1	0.483537	4
V$PARP_Q3	PARP	PARP1	0.0279517	4
V$IRF7_Q3	IRF-7	IRF7	0.177883	3
V$CEBPG_Q6_01	C/EBPgamma	CEBPG	0.210753	3
V$GFI1_Q6_01	Gfi1	GFI1	0.483537	3
V$SOX9_B1	SOX9	SOX9	0.0811999	3
V$STAT1_05	STAT1	STAT1	0.126554	3
V$RNF96_01	RNF96	TRIM28	0.531227	3
V$FOXM1_01	FOXM1	FOXM1	0.653598	3
V$CP2_01	CP2	TFCP2	0.0255312	2
V$GATA3_02	GATA-3	GATA3	0.300141	2
V$HOX13_02	HOXA5	HOXA5	0.0701904	2
V$GATA6_01	GATA-6	GATA6	0.035831	2
V$STAT3_01	STAT3	STAT3	0.0769405	1
V$SPIB_03	Spi-B	SPIB	0.0496612	1

----

hsa-mir-16-1

Matrix_id	transcription factor	Gene	PCC	Occurrence
V$PITX2_01	PITX2	PITX2	0.0568135	2
V$PITX2_Q2	Pitx2	PITX2	0.0568135	2
V$SPIB_03	Spi-B	SPIB	0.157515	2
V$IK_Q5	Ikaros	IKZF1	0.208065	2
V$PUR1_Q4	PUR1	PURA	0.0801906	2
V$ELF1_Q6	Elf-1	ELF1	0.451085	2
V$AHR_Q5	AhR	AHR	0.379238	2
V$IRF8_Q6	IRF-8	IRF8	0.45578	2
V$ETS2_Q6	c-Ets-2	ETS2	0.0119174	2
V$PARP_Q3	PARP	PARP1	0.222478	2
V$MAFB_01	MAFB	MAFB	0.110017	2
V$HBP1_Q2	hbp1	HBP1	0.367931	2
V$DLX5_01	dlx5	DLX5	0.0221504	2
V$FOXO4_02	FOXO4	FOXO4	0.25615	2
V$GKLF_Q4	GKLF	KLF4	0.176069	2
V$PARP_Q4	PARP	PARP1	0.222478	2

V$GR_Q6	GR	NR3C1	0.243596	2
V$ETS2_B	c-Ets-2	ETS2	0.0119174	2
V$MEF2C_Q4	MEF-2C	MEF2C	0.072969	2
V$YY1_01	YY1	YY1	0.471961	2
V$NFAT4_Q3	NF-AT4	NFATC3	0.393422	2
V$P300_01	p300	EP300	0.136604	2
V$GABPBETA_Q3	GABP-beta	GABPB1	0.173044	2
V$GATA3_01	GATA-3	GATA3	0.402271	2
V$HMGIY_01	HMGIY	HMGA1	0.43742	1
V$TTF1_Q5	TTF-1	NKX2-1	0.139561	1
V$TCF4_01	TCF-4	TCF7L2	0.107433	1
V$LEF1_Q5	LEF-1	LEF1	0.52525	1
V$GFI1_Q6_01	Gfi1	GFI1	0.369276	1
V$CMYB_Q5	c-Myb	MYB	0.360717	1
V$AML2_01	AML2	RUNX3	0.189055	1
V$CDP_04	CDP	CUX1	0.161203	1
V$GATA2_01	GATA-2	GATA2	0.134851	1
V$MYB_Q6	c-Myb	MYB	0.360717	1
V$GATA2_02	GATA-2	GATA2	0.134851	1
V$GATA2_03	GATA-2	GATA2	0.134851	1
V$GATA3_02	GATA-3	GATA3	0.402271	1
V$AMEF2_Q6	aMEF-2	MEF2A	0.204991	1
V$FAC1_01	FAC1	BPTF	0.374946	1
V$FOXO4_01	FOXO4	FOXO4	0.25615	1
V$AFP1_Q6	AFP1	ZFHX3	0.266602	1
V$CEBPD_Q6	C/EBPdelta	CEBPD	0.0585045	1
V$TCF4_Q5	TCF-4	TCF7L2	0.107433	1
V$GR_01	GR	NR3C1	0.243596	1
V$FOXM1_01	FOXM1	FOXM1	0.112792	1
V$STAT1_05	STAT1	STAT1	0.68435	1
V$CMYB_01	c-Myb	MYB	0.360717	1

----

hsa-mir-16-2

Matrix_id	transcription factor	Gene	PCC	Occurrence
V$SPIB_03	Spi-B	SPIB	0.157515	2
V$DLX5_01	dlx5	DLX5	0.0221504	2
V$MAFB_01	MAFB	MAFB	0.110017	2
V$PARP_Q3	PARP	PARP1	0.222478	2
V$ETS2_Q6	c-Ets-2	ETS2	0.0119174	2
V$IK_Q5	Ikaros	IKZF1	0.208065	2
V$YY1_Q6_02	YY1	YY1	0.471961	2
V$PUR1_Q4	PUR1	PURA	0.0801906	2

V$YY1_Q6	YY1	YY1	0.471961	2
V$IRF8_Q6	IRF-8	IRF8	0.45578	2
V$GKLF_Q4	GKLF	KLF4	0.176069	2
V$ATF1_Q6_01	ATF-1	ATF1	0.424276	2
V$TEL1_02	TEL1	ETV6	0.0501334	2
V$PARP_Q4	PARP	PARP1	0.222478	2
V$MEF2C_Q4	MEF-2C	MEF2C	0.072969	2
V$MEF2A_Q6	mef2A	MEF2A	0.204991	2
V$NFAT4_Q3	NF-AT4	NFATC3	0.393422	2
V$GABPBETA_Q3	GABP-beta	GABPB1	0.173044	2
V$BCL6_Q3_01	Bcl-6	BCL6	0.0301348	2
V$ATF3_Q6_01	ATF-3	ATF3	0.136991	2
V$ELF1_Q6	Elf-1	ELF1	0.451085	2
V$GR_Q6	GR	NR3C1	0.243596	2
V$RSRFC4_Q2	RSRFC4	MEF2A	0.204991	2
V$P300_01	p300	EP300	0.136604	2
V$ETS2_B	c-Ets-2	ETS2	0.0119174	2
V$YY1_01	YY1	YY1	0.471961	2
V$HIF1A_Q6	HIF-1alpha	HIF1A	0.189382	2
V$PITX2_Q2	Pitx2	PITX2	0.0568135	2
V$GATA3_01	GATA-3	GATA3	0.402271	2
V$SP1_Q6	Sp1	SP1	0.117796	2
V$CEBPG_Q6_01	C/EBPgamma	CEBPG	0.121066	1
V$CDP_02	CDP	CUX1	0.161203	1
V$ATF2_Q5	ATF-2	ATF2	0.0724699	1
V$MYB_Q6	c-Myb	MYB	0.360717	1
V$IRF1_Q6_01	IRF-1	IRF1	0.0789493	1
V$IRF7_Q3	IRF-7	IRF7	0.219251	1
V$YY1_03	YY1	YY1	0.471961	1
V$TTF1_Q5	TTF-1	NKX2-1	0.139561	1
V$CEBPB_Q6	C/EBPbeta	CEBPB	0.0382952	1
V$YY1_02	YY1	YY1	0.471961	1
V$CP2_01	CP2	TFCP2	0.254471	1
V$CEBPB_02	C/EBPbeta	CEBPB	0.0382952	1
V$GFI1_Q6_01	Gfi1	GFI1	0.369276	1
V$GATA2_01	GATA-2	GATA2	0.134851	1
V$ATF4_Q6	ATF-4	ATF4	0.0713477	1
V$CMYB_Q5	c-Myb	MYB	0.360717	1
V$RNF96_01	RNF96	TRIM28	0.222316	1
V$SP1_01	Sp1	SP1	0.117796	1
V$GFI1_Q6	Gfi1	GFI1	0.369276	1
V$SP1_Q2_01	Sp1	SP1	0.117796	1
V$SP1_Q4_01	Sp1	SP1	0.117796	1
V$ATF6_01	ATF6	ATF6	0.0236223	1
V$CEBPD_Q6	C/EBPdelta	CEBPD	0.0585045	1
V$IRF1_Q6	IRF-1	IRF1	0.0789493	1

V$AMEF2_Q6	aMEF-2	MEF2A	0.204991	1
V$GATA3_02	GATA-3	GATA3	0.402271	1
V$ZBP89_Q4	ZBP89	ZNF148	0.0282761	1
V$SP2_01	SP2	SP2	0.171699	1
V$SP1_Q6_01	Sp1	SP1	0.117796	1
V$NFAT2_Q5	NF-AT2	NFATC1	0.00800998	1
V$HMGIY_01	HMGIY	HMGA1	0.43742	1
V$MEF2A_05	MEF2A	MEF2A	0.204991	1
V$STAT1_05	STAT1	STAT1	0.68435	1
V$GATA2_02	GATA-2	GATA2	0.134851	1
V$FOXM1_01	FOXM1	FOXM1	0.112792	1

hsa-mir-17

Matrix_id	transcription factor	Gene	PCC	Occurrence
V$IK_Q5	Ikaros	IKZF1	0.211411	35
V$ELF1_Q6	Elf-1	ELF1	0.588036	34
V$ETS1_B	c-Ets-1	ETS1	0.065034	33
V$CETS1_Q6	C-ets-1	ETS1	0.065034	32
V$CMYB_Q5	c-Myb	MYB	0.724512	32
V$MYB_Q6	c-Myb	MYB	0.724512	32
V$MAFB_01	MAFB	MAFB	0.00836639	31
V$DLX5_01	dlx5	DLX5	0.0927624	29
V$NFAT4_Q3	NF-AT4	NFATC3	0.603291	29
V$YY1_01	YY1	YY1	0.138297	29
V$HMGIY_01	HMGIY	HMGA1	0.644162	24
V$AHR_Q5	AhR	AHR	0.192492	22
V$GFI1_Q6_01	Gfi1	GFI1	0.640864	21
V$ING4_01	ING4	ING4	0.222285	19
V$SREBP1_Q6	SREBP-1	SREBF1	0.0125372	17
V$FOXO4_02	FOXO4	FOXO4	0.117141	17
V$CMYB_01	c-Myb	MYB	0.724512	16
V$AP2ALPHA_01	AP-2alpha	TFAP2A	0.129008	14
V$AP2ALPHA_Q6	AP-2alpha	TFAP2A	0.129008	12
V$AP2GAMMA_01	AP-2gamma	TFAP2C	0.0514223	11
V$AML2_01	AML2	RUNX3	0.070263	11
V$FOXO4_01	FOXO4	FOXO4	0.117141	10
V$CP2_01	CP2	TFCP2	0.119473	9
V$FLI1_02	Fli-1	FLI1	0.00038874	5
V$AP2ALPHA_02	AP-2alphaA	TFAP2A	0.129008	2
V$AP2ALPHA_03	AP-2alphaA	TFAP2A	0.129008	1

hsa-mir-181a-1

Matrix_id	transcription factor	Gene	PCC	Occurrence
V$P300_01	p300	EP300	0.0327329	7
V$IK_Q5	Ikaros	IKZF1	0.0146265	7
V$MAFB_01	MAFB	MAFB	0.314605	7
V$PUR1_Q4	PUR1	PURA	0.21412	7
V$ELF1_Q6	Elf-1	ELF1	0.245454	7
V$YY1_Q6_03	YY1	YY1	0.28848	7
V$PARP_Q4	PARP	PARP1	0.332844	7
V$PBX1_Q3	Pbx1	PBX1	0.0135267	6
V$ING4_01	ING4	ING4	0.640921	6
V$NFAT4_Q3	NF-AT4	NFATC3	0.156185	6
V$AP4_Q6_02	AP-4	TFAP4	0.0278496	6
V$YY1_Q6	YY1	YY1	0.28848	5
V$MYB_Q6	c-Myb	MYB	0.265248	5
V$ETS2_Q6	c-Ets-2	ETS2	0.059237	5
V$MEF2C_Q4	MEF-2C	MEF2C	0.437175	5
V$GABPA_Q4	GABP-alpha	GABPA	0.151764	5
V$CMYB_Q5	c-Myb	MYB	0.265248	5
V$AP4_Q5	AP-4	TFAP4	0.0278496	5
V$GATA2_02	GATA-2	GATA2	0.119769	5
V$GATA3_02	GATA-3	GATA3	0.538261	5
V$YY1_01	YY1	YY1	0.28848	5
V$DLX5_01	dlx5	DLX5	0.315641	4
V$SOX10_Q6	SOX10	SOX10	0.306898	4
V$YY1_Q6_02	YY1	YY1	0.28848	4
V$GATA3_03	GATA-3	GATA3	0.538261	4
V$IRF8_Q6	IRF-8	IRF8	0.155707	4
V$TFIII_Q6	TFII-I	GTF2I	0.157267	4
V$GATA3_01	GATA-3	GATA3	0.538261	4
V$FOXM1_01	FOXM1	FOXM1	0.0347618	4
V$GR_Q6	GR	NR3C1	0.0644551	4
V$ETS2_B	c-Ets-2	ETS2	0.059237	4
V$GATA2_01	GATA-2	GATA2	0.119769	3
V$CEBPG_Q6_01	C/EBPgamma	CEBPG	0.263329	3
V$LEF1_Q5	LEF-1	LEF1	0.396508	3
V$GATA2_03	GATA-2	GATA2	0.119769	3
V$CEBPD_Q6	C/EBPdelta	CEBPD	0.197921	3
V$TCF4_Q5	TCF-4	TCF7L2	0.0382588	3
V$MEF2A_Q6	mef2A	MEF2A	0.254403	3
V$SREBP1_Q6	SREBP-1	SREBF1	0.209193	3
V$GR_01	GR	NR3C1	0.0644551	3
V$FOXO3A_Q1	FOXO3A	FOXO3	0.00116256	3

V$DEC2_Q2	2-Dec	BHLHE41	0.346768	2
V$CP2_01	CP2	TFCP2	0.544619	2
V$FOXO4_02	FOXO4	FOXO4	0.386297	2
V$GFI1_Q6	Gfi1	GFI1	0.302095	2
V$TCF4_01	TCF-4	TCF7L2	0.0382588	2
V$POU6F1_03	POU6F1	POU6F1	0.103271	2
V$MSX1_01	Msx-1	MSX1	0.0250862	2
V$NMYC_01	N-Myc	MYCN	0.175607	1
V$USF2_Q6	USF2	USF2	0.0398815	1
V$PBX1_04	Pbx1	PBX1	0.0135267	1
V$GFI1_Q6_01	Gfi1	GFI1	0.302095	1
V$SATB1_Q3	SATB1	SATB1	0.520394	1
V$POU6F1_02	POU6F1	POU6F1	0.103271	1
V$EGR2_01	Egr-2	EGR2	0.20325	1
V$SREBP1_01	SREBP-1	SREBF1	0.209193	1
V$RORBETA_Q2	RORBETA	RORB	0.0456342	1

----

hsa-mir-181a-2

Matrix_id	transcription factor	Gene	PCC	Occurrence
V$P300_01	p300	EP300	0.0327329	7
V$ELF1_Q6	Elf-1	ELF1	0.245454	7
V$PARP_Q4	PARP	PARP1	0.332844	7
V$PUR1_Q4	PUR1	PURA	0.21412	7
V$MAFB_01	MAFB	MAFB	0.314605	7
V$ING4_01	ING4	ING4	0.640921	6
V$NFAT4_Q3	NF-AT4	NFATC3	0.156185	6
V$PBX1_Q3	Pbx1	PBX1	0.0135267	6
V$GABPA_Q4	GABP-alpha	GABPA	0.151764	5
V$ETS2_Q6	c-Ets-2	ETS2	0.059237	5
V$GATA3_02	GATA-3	GATA3	0.538261	5
V$AP4_Q5	AP-4	TFAP4	0.0278496	5
V$YY1_01	YY1	YY1	0.28848	5
V$MEF2C_Q4	MEF-2C	MEF2C	0.437175	5
V$MYB_Q6	c-Myb	MYB	0.265248	5
V$CMYB_Q5	c-Myb	MYB	0.265248	5
V$GATA2_02	GATA-2	GATA2	0.119769	5
V$YY1_Q6	YY1	YY1	0.28848	5
V$DLX5_01	dlx5	DLX5	0.315641	4
V$IRF8_Q6	IRF-8	IRF8	0.155707	4
V$SOX10_Q6	SOX10	SOX10	0.306898	4
V$YY1_Q6_02	YY1	YY1	0.28848	4
V$ETS2_B	c-Ets-2	ETS2	0.059237	4

V$CEBPB_02	C/EBPbeta	CEBPB	0.218778	4
V$CEBPG_Q6	C/EBPgamma	CEBPG	0.263329	4
V$GATA3_01	GATA-3	GATA3	0.538261	4
V$FOXM1_01	FOXM1	FOXM1	0.0347618	4
V$PARP_Q3	PARP	PARP1	0.332844	3
V$TCF4_Q5	TCF-4	TCF7L2	0.0382588	3
V$FOXO3A_Q1	FOXO3A	FOXO3	0.00116256	3
V$SREBP1_Q6	SREBP-1	SREBF1	0.209193	3
V$MEF2A_Q6	mef2A	MEF2A	0.254403	3
V$LEF1_Q5	LEF-1	LEF1	0.396508	3
V$GATA2_03	GATA-2	GATA2	0.119769	3
V$AP4_Q6	AP-4	TFAP4	0.0278496	3
V$GFI1_Q6	Gfi1	GFI1	0.302095	2
V$MSX1_01	Msx-1	MSX1	0.0250862	2
V$HMGIY_01	HMGIY	HMGA1	0.314198	2
V$TCF4_01	TCF-4	TCF7L2	0.0382588	2
V$FOXO4_01	FOXO4	FOXO4	0.386297	2
V$FOXO4_02	FOXO4	FOXO4	0.386297	2
V$POU6F1_03	POU6F1	POU6F1	0.103271	2
V$PBX1_04	Pbx1	PBX1	0.0135267	1
V$POU6F1_02	POU6F1	POU6F1	0.103271	1
V$RORBETA_Q2	RORBETA	RORB	0.0456342	1
V$RSRFC4_01	RSRFC4	MEF2A	0.254403	1
V$GFI1_Q6_01	Gfi1	GFI1	0.302095	1

----

hsa-mir-181b-1

Matrix_id	transcription factor	Gene	PCC	Occurrence
V$PUR1_Q4	PUR1	PURA	0.233432	221
V$IK_Q5	Ikaros	IKZF1	0.00236632	219
V$ELF1_Q6	Elf-1	ELF1	0.0455922	207
V$MAFB_01	MAFB	MAFB	0.154448	206
V$PARP_Q4	PARP	PARP1	0.36657	206
V$ZIC3_01	Zic3	ZIC3	0.0355729	201
V$NFAT4_Q3	NF-AT4	NFATC3	0.0322154	183
V$MYB_Q6	c-Myb	MYB	0.246159	180
V$ETS2_Q6	c-Ets-2	ETS2	0.0731369	180
V$CMYB_Q5	c-Myb	MYB	0.246159	180
V$SOX9_Q4	SOX9	SOX9	0.0664039	179
V$GABPA_Q4	GABP-alpha	GABPA	0.00638435	175
V$YY1_01	YY1	YY1	0.0981945	164
V$AP4_Q6_02	AP-4	TFAP4	0.169186	164
V$MEF2C_Q4	MEF-2C	MEF2C	0.670208	157

V$SOX9_B1	SOX9	SOX9	0.0664039	150
V$YY1_Q6_02	YY1	YY1	0.0981945	146
V$YY1_Q6	YY1	YY1	0.0981945	146
V$ETS2_B	c-Ets-2	ETS2	0.0731369	145
V$TFIII_Q6	TFII-I	GTF2I	0.151038	137
V$ING4_01	ING4	ING4	0.734366	130
V$GATA3_01	GATA-3	GATA3	0.0413532	124
V$PBX1_04	Pbx1	PBX1	0.084533	124
V$GATA3_02	GATA-3	GATA3	0.0413532	122
V$AP4_Q5	AP-4	TFAP4	0.169186	121
V$LEF1_Q5	LEF-1	LEF1	0.337477	114
V$PBX1_Q3	Pbx1	PBX1	0.084533	111
V$GFI1_Q6	Gfi1	GFI1	0.210698	109
V$YY1_Q6_03	YY1	YY1	0.0981945	107
V$GFI1_Q6_01	Gfi1	GFI1	0.210698	105
V$SREBP1_Q6	SREBP-1	SREBF1	0.0332049	104
V$FOXM1_01	FOXM1	FOXM1	0.0409194	96
V$IRF8_Q6	IRF-8	IRF8	0.210383	95
V$SOX10_Q6	SOX10	SOX10	0.55624	92
V$GATA3_03	GATA-3	GATA3	0.0413532	85
V$CP2_01	CP2	TFCP2	0.25997	84
V$CEBPG_Q6_01	C/EBPgamma	CEBPG	0.062904	78
V$MEF2A_Q6	mef2A	MEF2A	0.516423	71
V$DBP_Q6_01	DBP	DBP	0.125443	64
V$NURR1_Q3	NURR1	NR4A2	0.00263606	62
V$MSX1_01	Msx-1	MSX1	0.0559476	51
V$HOXB8_01	HOXB8	HOXB8	0.164495	47
V$DEC2_Q2	2-Dec	BHLHE41	0.555073	41
V$EGR2_01	Egr-2	EGR2	0.210019	38
V$USF2_Q6	USF2	USF2	0.202392	32
V$RORBETA_Q2	RORBETA	RORB	0.161762	30
V$POU6F1_03	POU6F1	POU6F1	0.253449	28
V$SATB1_Q3	SATB1	SATB1	0.56059	23
V$POU6F1_02	POU6F1	POU6F1	0.253449	21
V$SREBP1_01	SREBP-1	SREBF1	0.0332049	12

---

hsa-mir-181b-2

Matrix_id	transcription factor	Gene	PCC	Occurrence
V$PUR1_Q4	PUR1	PURA	0.233432	221
V$ELF1_Q6	Elf-1	ELF1	0.0455922	207
V$MAFB_01	MAFB	MAFB	0.154448	206
V$PARP_Q4	PARP	PARP1	0.36657	206

V$ZIC3_01	Zic3	ZIC3	0.0355729	201
V$NFAT4_Q3	NF-AT4	NFATC3	0.0322154	183
V$MYB_Q6	c-Myb	MYB	0.246159	180
V$CMYB_Q5	c-Myb	MYB	0.246159	180
V$ETS2_Q6	c-Ets-2	ETS2	0.0731369	180
V$SOX9_Q4	SOX9	SOX9	0.0664039	179
V$GABPA_Q4	GABP-alpha	GABPA	0.00638435	175
V$YY1_01	YY1	YY1	0.0981945	164
V$MEF2C_Q4	MEF-2C	MEF2C	0.670208	157
V$SOX9_B1	SOX9	SOX9	0.0664039	150
V$YY1_Q6	YY1	YY1	0.0981945	146
V$YY1_Q6_02	YY1	YY1	0.0981945	146
V$ETS2_B	c-Ets-2	ETS2	0.0731369	145
V$PARP_Q3	PARP	PARP1	0.36657	137
V$ING4_01	ING4	ING4	0.734366	130
V$GATA3_01	GATA-3	GATA3	0.0413532	124
V$PBX1_04	Pbx1	PBX1	0.084533	124
V$GATA3_02	GATA-3	GATA3	0.0413532	122
V$HMGIY_01	HMGIY	HMGA1	0.127908	121
V$AP4_Q5	AP-4	TFAP4	0.169186	121
V$LEF1_Q5	LEF-1	LEF1	0.337477	114
V$AP4_Q6	AP-4	TFAP4	0.169186	113
V$PBX1_Q3	Pbx1	PBX1	0.084533	111
V$GFI1_Q6	Gfi1	GFI1	0.210698	109
V$GFI1_Q6_01	Gfi1	GFI1	0.210698	105
V$SREBP1_Q6	SREBP-1	SREBF1	0.0332049	104
V$FOXM1_01	FOXM1	FOXM1	0.0409194	96
V$IRF8_Q6	IRF-8	IRF8	0.210383	95
V$SOX10_Q6	SOX10	SOX10	0.55624	92
V$FAC1_01	FAC1	BPTF	0.0545814	79
V$MEF2A_Q6	mef2A	MEF2A	0.516423	71
V$NURR1_Q3	NURR1	NR4A2	0.00263606	62
V$MSX1_01	Msx-1	MSX1	0.0559476	51
V$CEBPG_Q6	C/EBPgamma	CEBPG	0.062904	34
V$RORBETA_Q2	RORBETA	RORB	0.161762	30
V$POU6F1_03	POU6F1	POU6F1	0.253449	28
V$RSRFC4_01	RSRFC4	MEF2A	0.516423	22
V$POU6F1_02	POU6F1	POU6F1	0.253449	21

----

hsa-mir-181c

Matrix_id	transcription factor	Gene	PCC	Occurrence
V$CMYB_01	c-Myb	MYB	0.351094	3

V$PARP_Q4	PARP	PARP1	0.222841	3
V$MEF2C_Q4	MEF-2C	MEF2C	0.415377	3
V$GKLF_Q4	GKLF	KLF4	0.0492646	3
V$PUR1_Q4	PUR1	PURA	0.0754386	3
V$HMGIY_01	HMGIY	HMGA1	0.21392	3
V$MAFB_01	MAFB	MAFB	0.31996	3
V$PARP_Q3	PARP	PARP1	0.222841	3
V$YY1_01	YY1	YY1	0.102029	3
V$AP4_Q6_02	AP-4	TFAP4	0.0055136	2
V$AHR_Q5	AhR	AHR	0.0348461	2
V$SREBP1_Q6	SREBP-1	SREBF1	0.0652646	2
V$GFI1_Q6_01	Gfi1	GFI1	0.292265	2
V$NFAT4_Q3	NF-AT4	NFATC3	0.123307	2
V$ZIC1_01	Zic1	ZIC1	0.555995	1
V$LEF1_Q5	LEF-1	LEF1	0.437343	1
V$RNF96_01	RNF96	TRIM28	0.0743387	1
V$TFIII_Q6	TFII-I	GTF2I	0.162326	1
V$SP2_01	SP2	SP2	0.158864	1
V$SOX10_Q6	SOX10	SOX10	0.324727	1
V$ERM_02	Erm	ETV5	0.123745	1

----

hsa-mir-181d

Matrix_id	transcription factor	Gene	PCC	Occurrence
V$PUR1_Q4	PUR1	PURA	0.167814	62
V$IK_Q5	Ikaros	IKZF1	0.0203872	60
V$PARP_Q4	PARP	PARP1	0.333632	58
V$MAFB_01	MAFB	MAFB	0.0994936	56
V$NFAT4_Q3	NF-AT4	NFATC3	0.0738611	52
V$YY1_01	YY1	YY1	0.0635341	50
V$AP4_Q6_02	AP-4	TFAP4	0.0547401	47
V$MEF2C_Q4	MEF-2C	MEF2C	0.603196	46
V$MAZ_Q6	MAZ	MAZ	0.040487	44
V$PARP_Q3	PARP	PARP1	0.333632	40
V$HMGIY_01	HMGIY	HMGA1	0.1661	35
V$CMYB_01	c-Myb	MYB	0.277851	33
V$TFIII_Q6	TFII-I	GTF2I	0.059118	32
V$SREBP1_Q6	SREBP-1	SREBF1	0.0261687	31
V$MAZ_Q6_01	MAZ	MAZ	0.040487	29
V$LEF1_Q5	LEF-1	LEF1	0.415919	29
V$GFI1_Q6_01	Gfi1	GFI1	0.257041	26
V$ERF_02	ERF	ERF	0.0149954	26
V$SOX10_Q6	SOX10	SOX10	0.469941	20

V$ZIC1_01	Zic1	ZIC1	0.733255	20
V$ERM_02	Erm	ETV5	0.0709157	13

hsa-mir-183

Matrix_id	transcription factor	Gene	PCC	Occurrence
V$MYB_Q6	c-Myb	MYB	0.772173	19
V$ELF1_Q6	Elf-1	ELF1	0.413991	19
V$IK_Q5	Ikaros	IKZF1	0.269933	19
V$CMYB_Q5	c-Myb	MYB	0.772173	19
V$GATA3_01	GATA-3	GATA3	0.142337	13
V$GATA3_02	GATA-3	GATA3	0.142337	13
V$GFI1_Q6_01	Gfi1	GFI1	0.630326	9
V$LEF1_Q5	LEF-1	LEF1	0.861342	9
V$ZABC1_01	ZABC1	ZNF217	0.0512304	8

hsa-mir-184

Matrix_id	transcription factor	Gene	PCC	Occurrence
V$PUR1_Q4	PUR1	PURA	0.236662	176
V$PEA3_Q6	PEA3	ETV4	0.0495682	173
V$PARP_Q4	PARP	PARP1	0.307426	171
V$ELF1_Q6	Elf-1	ELF1	0.0848758	167
V$GKLF_Q4	GKLF	KLF4	0.481682	166
V$MAFB_01	MAFB	MAFB	0.258459	164
V$ETS1_B	c-Ets-1	ETS1	0.118015	160
V$ZIC3_01	Zic3	ZIC3	0.195632	157
V$SMAD4_Q6_01	Smad4	SMAD4	0.259353	157
V$P300_01	p300	EP300	0.350625	152
V$CETS1_Q6	C-ets-1	ETS1	0.118015	151
V$GATA1_01	GATA-1	GATA1	0.0175807	149
V$SOX9_Q4	SOX9	SOX9	0.100345	149
V$GABPA_Q4	GABP-alpha	GABPA	0.412232	145
V$TBX5_02	TBX5	TBX5	0.0333064	140
V$GR_Q6	GR	NR3C1	0.116517	134
V$TBP_Q6	TBP	TBP	0.411472	134
V$AML1_Q6	AML1	RUNX1	0.00455339	131
V$AP4_Q6_02	AP-4	TFAP4	0.0603452	125
V$MEF2C_Q4	MEF-2C	MEF2C	0.182618	125
V$YY1_Q6_02	YY1	YY1	0.420658	124

V$YY1_Q6	YY1	YY1	0.420658	122
V$GATA1_02	GATA-1	GATA1	0.0175807	120
V$TBX5_01	TBX5	TBX5	0.0333064	115
V$GATA2_02	GATA-2	GATA2	0.572916	113
V$GATA1_05	GATA-1	GATA1	0.0175807	113
V$GATA1_06	GATA-1	GATA1	0.0175807	113
V$AHR_Q5	AhR	AHR	0.813665	112
V$PARP_Q3	PARP	PARP1	0.307426	110
V$ERBETA_Q5	ER-beta	ESR2	0.0870371	107
V$E2A_Q6	E2A	TCF3	0.0530606	106
V$E12_Q6	E12	TCF3	0.0530606	106
V$E47_02	E47	TCF3	0.0530606	104
V$MYOGENIN_Q6	myogenin	MYOG	0.00963463	100
V$GATA3_01	GATA-3	GATA3	0.797233	99
V$GATA1_04	GATA-1	GATA1	0.0175807	99
V$WT1_Q6	WT1	WT1	0.0130678	97
V$GATA3_02	GATA-3	GATA3	0.797233	97
V$E2A_Q2	E2A	TCF3	0.0530606	92
V$SREBP1_Q6	SREBP-1	SREBF1	0.180968	90
V$GATA2_01	GATA-2	GATA2	0.572916	89
V$AP4_Q5	AP-4	TFAP4	0.0603452	89
V$CMAF_01	c-Maf	MAF	0.122691	86
V$AML1_01	AML1a	RUNX1	0.00455339	84
V$GR_01	GR	NR3C1	0.116517	83
V$TCF4_01	TCF-4	TCF7L2	0.399469	83
V$CEBPA_Q6	C/EBPalpha	CEBPA	0.0113686	82
V$AP4_Q6	AP-4	TFAP4	0.0603452	80
V$TCF4_Q5	TCF-4	TCF7L2	0.399469	79
V$MYOGENIN_Q6_01	myogenin	MYOG	0.00963463	76
V$AML1_Q4	AML1	RUNX1	0.00455339	75
V$FOXM1_01	FOXM1	FOXM1	0.112631	75
V$NKX2B_Q3	NKX2B	NKX2-2	0.10446	71
V$NKX22_02	NKX2B	NKX2-2	0.10446	65
V$HBP1_Q2	hbp1	HBP1	0.499467	64
V$FAC1_01	FAC1	BPTF	0.0632506	61
V$CEBPB_02	C/EBPbeta	CEBPB	0.40595	58
V$E47_01	E47	TCF3	0.0530606	54
V$AP4_Q6_01	AP-4	TFAP4	0.0603452	50
V$GATA2_03	GATA-2	GATA2	0.572916	50
V$STAT3_03	STAT3	STAT3	0.0733801	49
V$MEF2A_Q6	mef2A	MEF2A	0.0784219	43
V$CIZ_01	CIZ	ZNF384	0.15858	40
V$RORBETA_Q2	RORBETA	RORB	0.0414371	33
V$POU6F1_03	POU6F1	POU6F1	0.0344948	31
V$E2F1_Q4	E2F-1	E2F1	0.0154376	28
V$FOXJ2_02	FOXJ2	FOXJ2	0.106655	28

V$CDP_04	CDP	CUX1	0.31857	25
V$MAFK_Q3	MafK	MAFK	0.163016	21
V$TCF4_Q5_01	TCF-4	TCF7L2	0.399469	12
V$STAT4_Q4	STAT4	STAT4	0.0124705	9
V$POU6F1_01	POU6F1	POU6F1	0.0344948	8
V$E4BP4_01	E4BP4	NFIL3	0.125057	7
V$CDP_02	CDP	CUX1	0.31857	3

----

hsa-mir-188

Matrix_id	transcription factor	Gene	PCC	Occurrence
V$IK_Q5	Ikaros	IKZF1	0.233897	15
V$YY1_01	YY1	YY1	0.308546	15
V$PUR1_Q4	PUR1	PURA	0.130449	15
V$AP2REP_01	AP-2rep	KLF12	0.273275	15
V$ZIC3_01	Zic3	ZIC3	0.523745	14
V$SMAD3_Q6_01	Smad3	SMAD3	0.271471	14
V$SMAD4_Q6_01	Smad4	SMAD4	0.433909	14
V$YY1_Q6	YY1	YY1	0.308546	14
V$AML1_Q6	AML1	RUNX1	0.287998	14
V$ELF1_Q6	Elf-1	ELF1	0.016575	14
V$GATA1_01	GATA-1	GATA1	0.0446157	14
V$P300_01	p300	EP300	0.484412	14
V$PARP_Q4	PARP	PARP1	0.242996	14
V$DLX5_01	dlx5	DLX5	0.564617	13
V$SOX5_01	SOX5	SOX5	0.44024	13
V$NR1B2_Q6	NR1B2	RARB	0.398326	13
V$IPF1_01	IPF1	PDX1	0.339611	13
V$GKLF_Q4	GKLF	KLF4	0.233783	13
V$TBX5_02	TBX5	TBX5	0.249654	12
V$TBP_Q6	TBP	TBP	0.394132	12
V$YY1_Q6_02	YY1	YY1	0.308546	12
V$CDX2_Q5_02	CDX-2	CDX2	0.307564	12
V$NFAT4_Q3	NF-AT4	NFATC3	0.195203	12
V$CRX_Q4	Crx	CRX	0.402791	12
V$IRF8_Q6	IRF-8	IRF8	0.0226705	11
V$HNF4A_Q6_01	HNF-4alpha	HNF4A	0.425346	11
V$YY1_Q6_03	YY1	YY1	0.308546	11
V$ZBP89_Q4	ZBP89	ZNF148	0.0739106	11
V$TBX5_01	TBX5	TBX5	0.249654	11
V$PITX3_Q2	PITX3	PITX3	0.201958	11
V$NANOG_02	Nanog	NANOG	0.295458	11
V$NFAT2_Q5	NF-AT2	NFATC1	0.250235	11

V$SRY_02	SRY	SRY	0.148689	11
V$GR_Q6	GR	NR3C1	0.325925	11
V$GATA1_02	GATA-1	GATA1	0.0446157	11
V$PITX2_Q2	Pitx2	PITX2	0.388841	11
V$GATA2_02	GATA-2	GATA2	0.464684	11
V$GATA1_05	GATA-1	GATA1	0.0446157	11
V$GATA1_06	GATA-1	GATA1	0.0446157	11
V$AP4_Q6_02	AP-4	TFAP4	0.21889	10
V$IPF1_Q4_01	IPF1	PDX1	0.339611	10
V$HNF3A_01	HNF3A	FOXA1	0.0653516	10
V$GATA1_04	GATA-1	GATA1	0.0446157	10
V$AHR_Q5	AhR	AHR	0.255312	10
V$CDX2_01	Cdx-2	CDX2	0.307564	10
V$BEN_01	BEN	GTF2IRD1	0.277114	10
V$GATA3_02	GATA-3	GATA3	0.477043	10
V$IPF1_Q6	IPF1	PDX1	0.339611	10
V$SP1_Q6	Sp1	SP1	0.366697	9
V$PBX1_04	Pbx1	PBX1	0.0885437	9
V$CDX2_Q5_01	Cdx-2	CDX2	0.307564	9
V$RFX1_02	RFX1	RFX1	0.305393	9
V$PITX2_01	PITX2	PITX2	0.388841	9
V$SP1_Q4_01	Sp1	SP1	0.366697	9
V$SP1_02	SP1	SP1	0.366697	9
V$WT1_Q6_01	WT1	WT1	0.164918	9
V$SP1_Q6_01	Sp1	SP1	0.366697	9
V$NKX32_01	Nkx3-2	NKX3-2	0.282579	9
V$SMAD3_Q6	SMAD3	SMAD3	0.271471	9
V$SREBP1_Q6	SREBP-1	SREBF1	0.178179	8
V$CRX_02	Crx	CRX	0.402791	8
V$CP2_01	CP2	TFCP2	0.474268	8
V$TEF1_Q6	TEF-1	TEAD1	0.342565	8
V$YY1_02	YY1	YY1	0.308546	8
V$PITX1_01	Pitx1	PITX1	0.23945	8
V$SP1_Q2_01	Sp1	SP1	0.366697	8
V$AP2ALPHA_01	AP-2alpha	TFAP2A	0.442639	8
V$PARP_Q3	PARP	PARP1	0.242996	8
V$IPF1_Q4	IPF1	PDX1	0.339611	7
V$FOXO4_02	FOXO4	FOXO4	0.440952	7
V$ZABC1_01	ZABC1	ZNF217	0.375635	7
V$HMGIY_01	HMGIY	HMGA1	0.224424	7
V$FOXJ2_01	FOXJ2	FOXJ2	0.420893	7
V$TCF4_01	TCF-4	TCF7L2	0.233212	7
V$GATA2_01	GATA-2	GATA2	0.464684	6
V$GATA3_01	GATA-3	GATA3	0.477043	6
V$CRX_Q4_01	CRX	CRX	0.402791	6
V$CEBPB_02	C/EBPbeta	CEBPB	0.105614	6

V$CDX1_01	Cdx-1	CDX1	0.130514	6
V$GATA3_03	GATA-3	GATA3	0.477043	6
V$GATA2_03	GATA-2	GATA2	0.464684	5
V$HIF1A_Q6	HIF-1alpha	HIF1A	0.217263	5
V$AP2GAMMA_01	AP-2gamma	TFAP2C	0.40565	5
V$HOXA9_01	hoxa9	HOXA9	0.549002	5
V$TEF1_Q6_03	TEF-1	TEAD1	0.342565	5
V$NKX32_02	Nkx3-2	NKX3-2	0.282579	5
V$IPF1_02	IPF1	PDX1	0.339611	5
V$ARNT_01	Arnt	ARNT	0.487393	5
V$TCF4_Q5	TCF-4	TCF7L2	0.233212	5
V$HOX13_02	HOXA5	HOXA5	0.206732	4
V$SP2_01	SP2	SP2	0.268538	4
V$HOXD9_Q2	Hoxd9	HOXD9	0.375816	4
V$NCX_02	Ncx	TLX2	0.0836869	4
V$E2F1_Q3_01	E2F-1	E2F1	0.360432	3
V$BACH2_01	Bach2	BACH2	0.18911	3
V$LHX3b_01	LHX3b	LHX3	0.318726	3
V$BCL6_Q3_01	Bcl-6	BCL6	0.176724	3
V$IPF1_03	IPF1	PDX1	0.339611	3
V$IPF1_06	ipf1	PDX1	0.339611	3
V$STAT4_Q5	STAT4	STAT4	0.327038	3
V$CART1_02	CART1	ALX1	0.416399	3
V$CEBPG_Q6_01	C/EBPgamma	CEBPG	0.06065	3
V$POU6F1_02	POU6F1	POU6F1	0.333475	2
V$POU6F1_03	POU6F1	POU6F1	0.333475	2
V$YY1_03	YY1	YY1	0.308546	2
V$NRF1_Q6	NRF-1	NRF1	0.27369	1
V$CDP_04	CDP	CUX1	0.253099	1

---

hsa-mir-18a

Matrix_id	transcription factor	Gene	PCC	Occurrence
V$ELK1_06	ELK-1	ELK1	0.026234	2
V$DLX5_01	dlx5	DLX5	0.32268	2
V$MAFB_01	MAFB	MAFB	0.0155749	2
V$PARP_Q3	PARP	PARP1	0.231703	2
V$IK_Q5	Ikaros	IKZF1	0.015615	2
V$SOX9_Q4	SOX9	SOX9	0.280814	2
V$TBP_Q6	TBP	TBP	0.478791	2
V$E2F1_Q6_01	E2F-1	E2F1	0.0578629	2
V$AHR_Q5	AhR	AHR	0.143207	2
V$AML1_Q4	AML1	RUNX1	0.035692	2

V$ING4_01	ING4	ING4	0.323642	2
V$PARP_Q4	PARP	PARP1	0.231703	2
V$HNF3B_Q6	HNF-3beta	FOXA2	0.0536362	2
V$GFI1_Q6_01	Gfi1	GFI1	0.105969	2
V$NFAT4_Q3	NF-AT4	NFATC3	0.218265	2
V$AP2ALPHA_Q6	AP-2alpha	TFAP2A	0.166616	2
V$GKLF_Q4	GKLF	KLF4	0.185677	2
V$CMYB_Q5	c-Myb	MYB	0.13213	2
V$SP2_01	SP2	SP2	0.0537527	2
V$AML1_Q6	AML1	RUNX1	0.035692	2
V$SREBP1_Q6	SREBP-1	SREBF1	0.170175	2
V$ELF1_Q6	Elf-1	ELF1	0.125885	2
V$E2F1_Q3	E2F-1	E2F1	0.0578629	2
V$ETS1_B	c-Ets-1	ETS1	0.0188762	2
V$AML1_01	AML1a	RUNX1	0.035692	2
V$MYB_Q6	c-Myb	MYB	0.13213	2
V$CP2_01	CP2	TFCP2	0.242186	2
V$YY1_01	YY1	YY1	0.0675607	2
V$ELK1_02	Elk-1	ELK1	0.026234	2
V$E2F1_Q6	E2F-1	E2F1	0.0578629	2
V$ZIC3_01	Zic3	ZIC3	0.101243	2
V$AP2ALPHA_01	AP-2alpha	TFAP2A	0.166616	2
V$AP2GAMMA_01	AP-2gamma	TFAP2C	0.0646002	2
V$MAZ_Q6	MAZ	MAZ	0.171785	2
V$CETS1_Q6	C-ets-1	ETS1	0.0188762	1
V$FOXO4_02	FOXO4	FOXO4	0.258419	1
V$STAT5A_Q6	STAT5A	STAT5A	0.0618496	1
V$SOX5_01	SOX5	SOX5	0.202431	1
V$E2F1_Q3_01	E2F-1	E2F1	0.0578629	1
V$ELK1_01	Elk-1	ELK1	0.026234	1
V$SPI1_Q5	SPI1	SPI1	0.0172005	1
V$ERM_02	Erm	ETV5	0.40874	1
V$FOXO4_01	FOXO4	FOXO4	0.258419	1
V$DEAF1_01	DEAF1	DEAF1	0.0849712	1
V$CMYB_01	c-Myb	MYB	0.13213	1
V$AP2ALPHA_02	AP-2alphaA	TFAP2A	0.166616	1
V$E2F1_Q4	E2F-1	E2F1	0.0578629	1
V$HNF1B_01	HNF-1beta	HNF1B	0.0114803	1
V$HMGIY_01	HMGIY	HMGA1	0.668701	1
V$FOXO3A_Q1	FOXO3A	FOXO3	0.0267939	1
V$SOX9_B1	SOX9	SOX9	0.280814	1
V$AML2_01	AML2	RUNX3	0.0495851	1
V$AP2ALPHA_03	AP-2alphaA	TFAP2A	0.166616	1
V$SPI1_03	SPI1	SPI1	0.0172005	1

----

hsa-mir-190a

Matrix_id	transcription factor	Gene	PCC	Occurrence
V$PUR1_Q4	PUR1	PURA	0.133412	108
V$PARP_Q4	PARP	PARP1	0.147112	106
V$IK_Q5	Ikaros	IKZF1	0.00502726	103
V$MAFB_01	MAFB	MAFB	0.0427256	101
V$SMAD4_Q6_01	Smad4	SMAD4	0.0187374	99
V$P300_01	p300	EP300	0.0592142	93
V$TBP_Q6	TBP	TBP	0.041402	93
V$CDX2_Q5_02	CDX-2	CDX2	0.069295	93
V$GR_Q6	GR	NR3C1	0.0744042	92
V$SOX9_Q4	SOX9	SOX9	0.0183352	91
V$ZIC3_01	Zic3	ZIC3	0.645027	89
V$SMAD3_Q6_01	Smad3	SMAD3	0.0855845	89
V$MEF2C_Q4	MEF-2C	MEF2C	0.146786	86
V$HNF4A_Q6_01	HNF-4alpha	HNF4A	0.642871	85
V$CDX2_Q5_01	Cdx-2	CDX2	0.069295	82
V$NANOG_02	Nanog	NANOG	0.00481978	81
V$DLX5_01	dlx5	DLX5	0.105009	80
V$IPF1_01	IPF1	PDX1	0.088095	78
V$IPF1_Q6	IPF1	PDX1	0.088095	75
V$Elf5_03	ELF5	ELF5	0.26605	74
V$IPF1_Q4_01	IPF1	PDX1	0.088095	74
V$GATA3_02	GATA-3	GATA3	0.0440501	72
V$CDX2_01	Cdx-2	CDX2	0.069295	70
V$IRF4_Q6	IRF-4	IRF4	0.174815	68
V$PBX1_04	Pbx1	PBX1	0.184061	67
V$PARP_Q3	PARP	PARP1	0.147112	66
V$IPF1_Q4	IPF1	PDX1	0.088095	66
V$ING4_01	ING4	ING4	0.255315	65
V$PBX1_Q3	Pbx1	PBX1	0.184061	64
V$E47_02	E47	TCF3	0.0276974	63
V$E2A_Q6	E2A	TCF3	0.0276974	63
V$E12_Q6	E12	TCF3	0.0276974	63
V$CMAF_01	c-Maf	MAF	0.650497	63
V$GFI1_Q6	Gfi1	GFI1	0.0674517	62
V$TEF1_Q6_03	TEF-1	TEAD1	0.0325027	61
V$CEBPE_Q6	CEBPE	CEBPE	0.233534	60
V$FOXJ2_01	FOXJ2	FOXJ2	0.00802484	59
V$TCF3_01	TCF-3	TCF7L1	0.295958	56
V$PITX3_Q2	PITX3	PITX3	0.0717642	55
V$GFI1_Q6_01	Gfi1	GFI1	0.0674517	55
V$SOX10_Q6	SOX10	SOX10	0.257553	54

V$ARNT_01	Arnt	ARNT	0.117427	53
V$CRX_Q4	Crx	CRX	0.273239	51
V$OC2_Q3	OC-2	ONECUT2	0.191773	50
V$IRF8_Q6	IRF-8	IRF8	0.029708	49
V$NKX2B_Q3	NKX2B	NKX2-2	0.1974	48
V$E2A_Q2	E2A	TCF3	0.0276974	47
V$HOXD9_Q2	Hoxd9	HOXD9	0.221267	46
V$NKX22_02	NKX2B	NKX2-2	0.1974	42
V$HOXA9_01	hoxa9	HOXA9	0.348983	40
V$TFIII_Q6	TFII-I	GTF2I	0.405843	40
V$PIT1_Q6	Pit-1	POU1F1	0.358872	39
V$ZIC1_01	Zic1	ZIC1	0.217433	38
V$CRX_02	Crx	CRX	0.273239	35
V$HNF6_Q6	HNF6	ONECUT1	0.0380773	34
V$VDR_Q3	VDR	VDR	0.227117	33
V$DEC2_Q2	2-Dec	BHLHE41	0.0108301	26
V$NMYC_01	N-Myc	MYCN	0.116638	23
V$MAX_01	Max	MAX	0.218547	23
V$ARNT_02	Arnt	ARNT	0.117427	23
V$USF2_Q6	USF2	USF2	0.0933076	23
V$POU6F1_03	POU6F1	POU6F1	0.591134	23
V$SOX2_Q6	SOX2	SOX2	0.354484	21
V$FOXJ2_02	FOXJ2	FOXJ2	0.00802484	18
V$POU6F1_02	POU6F1	POU6F1	0.591134	14
V$HOXA7_01	HOXA7	HOXA7	0.29698	8
V$MTF1_Q4	MTF-1	MTF1	0.0232495	7
V$RP58_01	RP58	ZNF238	0.202616	3

---

hsa-mir-191

Matrix_id	transcription factor	Gene	PCC	Occurrence
V$PUR1_Q4	PUR1	PURA	0.235691	133
V$PARP_Q4	PARP	PARP1	0.138169	131
V$ELF1_Q6	Elf-1	ELF1	0.170718	127
V$GKLF_Q4	GKLF	KLF4	0.486987	125
V$MAFB_01	MAFB	MAFB	0.28932	123
V$P300_01	p300	EP300	0.16206	118
V$SMAD4_Q6_01	Smad4	SMAD4	0.121392	117
V$SOX9_Q4	SOX9	SOX9	0.00311084	115
V$YY1_01	YY1	YY1	0.413492	107
V$GR_Q6	GR	NR3C1	0.0649368	104
V$MEF2C_Q4	MEF-2C	MEF2C	0.0862625	93
V$SOX9_B1	SOX9	SOX9	0.00311084	93

V$DLX5_01	dlx5	DLX5	0.598519	90
V$ING4_01	ING4	ING4	0.253979	84
V$GATA3_01	GATA-3	GATA3	0.792503	78
V$PBX1_Q3	Pbx1	PBX1	0.0895107	75
V$FKLF_Q5	FKLF	KLF11	0.109052	73
V$SREBP1_Q6	SREBP-1	SREBF1	0.404197	71
V$PBX1_04	Pbx1	PBX1	0.0895107	71
V$GATA2_01	GATA-2	GATA2	0.524715	70
V$GR_01	GR	NR3C1	0.0649368	66
V$TCF4_01	TCF-4	TCF7L2	0.39479	63
V$TCF4_Q5	TCF-4	TCF7L2	0.39479	59
V$CREM_Q6	CREM	CREM	0.271444	54
V$ZIC1_01	Zic1	ZIC1	0.149817	50
V$NKX2B_Q3	NKX2B	NKX2-2	0.0408919	50
V$NKX22_02	NKX2B	NKX2-2	0.0408919	45
V$GABPBETA_Q3	GABP-beta	GABPB1	0.165157	44
V$HBP1_Q2	hbp1	HBP1	0.485157	44
V$ESE1_Q3	ESE-1	ELF3	0.0199231	41
V$CEBPG_Q6_01	C/EBPgamma	CEBPG	0.522346	38
V$ATF3_Q6_01	ATF-3	ATF3	0.365668	37
V$ESE1_02	ESE-1	ELF3	0.0199231	31
V$HOX13_02	HOXA5	HOXA5	0.0606635	28
V$CEBPG_Q6	C/EBPgamma	CEBPG	0.522346	23
V$IRF1_Q6	IRF-1	IRF1	0.0552914	23
V$AMEF2_Q6	aMEF-2	MEF2A	0.104815	14
V$ERR3_Q2_01	ERR3	ESRRG	0.327532	12
V$ZID_01	ZID	ZBTB6	0.220683	11

hsa-mir-192

Matrix_id	transcription factor	Gene	PCC	Occurrence
V$NR1B2_Q6	NR1B2	RARB	0.174267	90
V$ELF1_Q6	Elf-1	ELF1	0.263702	89
V$ZIC3_01	Zic3	ZIC3	0.149787	87
V$MAFB_01	MAFB	MAFB	0.23255	87
V$TBX5_02	TBX5	TBX5	0.011504	82
V$CETS1_Q6	C-ets-1	ETS1	0.179198	77
V$SMAD3_Q6_01	Smad3	SMAD3	0.0877125	75
V$Elf5_03	ELF5	ELF5	0.0985981	74
V$GABPA_Q4	GABP-alpha	GABPA	0.422648	73
V$HNF4A_Q6_01	HNF-4alpha	HNF4A	0.538672	72
V$ETS1_B	c-Ets-1	ETS1	0.179198	72
V$TBX5_01	TBX5	TBX5	0.011504	71

V$SOX5_01	SOX5	SOX5	0.172314	70
V$MYOD_Q6_01	MyoD	MYOD1	0.245684	67
V$PITX3_Q2	PITX3	PITX3	0.142364	67
V$ERBETA_Q5	ER-beta	ESR2	0.298845	65
V$SPI1_Q5	SPI1	SPI1	0.0790735	63
V$SPI1_03	SPI1	SPI1	0.0790735	62
V$MYOGENIN_Q6	myogenin	MYOG	0.175591	60
V$ING4_01	ING4	ING4	0.0582535	59
V$CRX_Q4	Crx	CRX	0.305166	58
V$ERR1_Q3	ERR1	ESRRA	0.188443	55
V$HNF3B_Q6	HNF-3beta	FOXA2	0.288641	51
V$CEBPA_Q6	C/EBPalpha	CEBPA	0.735343	50
V$SREBP1_Q6	SREBP-1	SREBF1	0.0757703	48
V$CRX_02	Crx	CRX	0.305166	47
V$TCF3_01	TCF-3	TCF7L1	0.332695	46
V$MYOGENIN_Q6_01	myogenin	MYOG	0.175591	45
V$HNF1_02	HNF-1alpha	HNF1A	0.392393	44
V$CP2_01	CP2	TFCP2	0.00028534	44
V$CEBPE_Q6	CEBPE	CEBPE	0.113178	43
V$OC2_Q3	OC-2	ONECUT2	0.104081	43
V$FKLF_Q5	FKLF	KLF11	0.25873	42
V$ETV3_02	ETV3	ETV3	0.3477	41
V$MYOD_Q6	MyoD	MYOD1	0.245684	40
V$ER71_02	ER71	ETV2	0.260703	40
V$ETV7_01	ETV7	ETV7	0.0353326	39
V$CRX_Q4_01	CRX	CRX	0.305166	39
V$CEBPB_Q6	C/EBPbeta	CEBPB	0.0104741	38
V$CEBPB_02	C/EBPbeta	CEBPB	0.0104741	37
V$ESE1_Q3	ESE-1	ELF3	0.254816	36
V$MYOD_Q6_02	MyoD	MYOD1	0.245684	34
V$MATH1_Q2	MATH1	ATOH1	0.244011	33
V$ERF_02	ERF	ERF	0.316362	33
V$MYOD_01	MyoD	MYOD1	0.245684	30
V$ESE1_02	ESE-1	ELF3	0.254816	29
V$STAT3_03	STAT3	STAT3	0.233977	29
V$VDR_Q3	VDR	VDR	0.0688389	28
V$PDEF_02	PDEF	SPDEF	0.0137016	24
V$NCX_02	Ncx	TLX2	0.0481292	24
V$SOX2_Q6	SOX2	SOX2	0.514508	22
V$CART1_02	CART1	ALX1	0.0783869	19
V$ERG_03	ERG	ERG	0.023064	17
V$PET1_02	Pet-1	FEV	0.0306544	16
V$ERG_01	ERG	ERG	0.023064	15
V$PAX3_B	Pax-3	PAX3	0.140579	13
V$HOXA7_01	HOXA7	HOXA7	0.501182	6
V$HEN1_02	HEN1	NHLH1	0.164437	4

V$HEN1_01	HEN1	NHLH1	0.164437	3

---

hsa-mir-193b

Matrix_id	transcription factor	Gene	PCC	Occurrence
V$PUR1_Q4	PUR1	PURA	0.250979	215
V$PEA3_Q6	PEA3	ETV4	0.0872851	213
V$ELF1_Q6	Elf-1	ELF1	0.216438	208
V$GKLF_Q4	GKLF	KLF4	0.108803	205
V$SMAD4_Q6_01	Smad4	SMAD4	0.156396	186
V$SOX9_Q4	SOX9	SOX9	0.107994	176
V$ETS2_Q6	c-Ets-2	ETS2	0.0430831	176
V$Elf5_03	ELF5	ELF5	0.242552	173
V$ETS2_B	c-Ets-2	ETS2	0.0430831	169
V$SMAD3_Q6_01	Smad3	SMAD3	0.0159133	167
V$GR_Q6	GR	NR3C1	0.209921	162
V$NANOG_02	Nanog	NANOG	0.0902886	159
V$AP4_Q6_02	AP-4	TFAP4	0.077602	150
V$YY1_Q6	YY1	YY1	0.0482962	142
V$YY1_Q6_02	YY1	YY1	0.0482962	142
V$BEN_01	BEN	GTF2IRD1	0.0448514	138
V$AHR_Q5	AhR	AHR	0.0839122	133
V$IRF4_Q6	IRF-4	IRF4	0.0296239	131
V$AP2ALPHA_Q6	AP-2alpha	TFAP2A	0.0536582	130
V$MYOGENIN_Q6	myogenin	MYOG	0.0496787	125
V$AP2ALPHA_01	AP-2alpha	TFAP2A	0.0536582	121
V$WT1_Q6	WT1	WT1	0.121693	119
V$GATA3_01	GATA-3	GATA3	0.0570631	119
V$FKLF_Q5	FKLF	KLF11	0.00027765	119
V$ZBP89_Q4	ZBP89	ZNF148	0.133675	118
V$GATA2_01	GATA-2	GATA2	0.218081	107
V$TEF1_Q6_03	TEF-1	TEAD1	0.0556583	107
V$AP4_Q5	AP-4	TFAP4	0.077602	105
V$CMAF_01	c-Maf	MAF	0.151825	105
V$ER71_02	ER71	ETV2	0.119802	99
V$TEF1_Q6	TEF-1	TEAD1	0.0556583	98
V$WT1_Q6_01	WT1	WT1	0.121693	96
V$ELF5_01	ELF5	ELF5	0.242552	93
V$AP4_Q6	AP-4	TFAP4	0.077602	92
V$AP2BETA_Q3	AP-2beta	TFAP2B	0.00929266	90
V$EGR1_02	EGR-1	EGR1	0.306662	87
V$SMAD3_Q6	SMAD3	SMAD3	0.0159133	86
V$ESE1_Q3	ESE-1	ELF3	0.560503	86

V$FOXJ2_01	FOXJ2	FOXJ2	0.0204771	86
V$MYOGENIN_Q6_01	myogenin	MYOG	0.0496787	85
V$AP2GAMMA_01	AP-2gamma	TFAP2C	0.113782	84
V$GABPBETA_Q3	GABP-beta	GABPB1	0.120139	82
V$VDR_Q3	VDR	VDR	0.145623	82
V$EHF_03	EHF	EHF	0.258502	81
V$FAC1_01	FAC1	BPTF	0.117772	77
V$ESE1_02	ESE-1	ELF3	0.560503	67
V$PDEF_02	PDEF	SPDEF	0.479045	61
V$STAT3_03	STAT3	STAT3	0.167708	61
V$FOXO4_02	FOXO4	FOXO4	0.107459	59
V$ATF3_Q6_01	ATF-3	ATF3	0.331785	56
V$ATF1_Q6_01	ATF-1	ATF1	0.148103	56
V$CACCCBINDINGFACT	CACCC-binding factor	ZNF148	0.133675	55
V$PET1_02	Pet-1	FEV	0.318155	51
V$ERG_03	ERG	ERG	0.0554592	47
V$SP4_Q5	SP4	SP4	0.00014896	45
V$AP4_01	AP-4	TFAP4	0.077602	43
V$AR_03	AR	AR	0.0487227	39
V$EKLF_Q5	EKLF	KLF1	0.0402713	39
V$ERG_01	ERG	ERG	0.0554592	33
V$AP2ALPHA_02	AP-2alphaA	TFAP2A	0.0536582	32
V$ATF6_01	ATF6	ATF6	0.213098	23
V$RREB1_01	RREB-1	RREB1	0.0251675	22
V$STAT5A_Q6	STAT5A	STAT5A	0.0362782	18
V$MTF1_Q4	MTF-1	MTF1	0.0264957	13
V$AR_04	AR	AR	0.0487227	12
V$AR_01	AR	AR	0.0487227	9

----

hsa-mir-194-1

Matrix_id	transcription factor	Gene	PCC	Occurrence
V$MAFB_01	MAFB	MAFB	0.235128	109
V$ELF1_Q6	Elf-1	ELF1	0.268591	109
V$NR1B2_Q6	NR1B2	RARB	0.228664	107
V$ZIC3_01	Zic3	ZIC3	0.229358	106
V$TBX5_02	TBX5	TBX5	0.0245151	102
V$CDX2_Q5_02	CDX-2	CDX2	0.12792	98
V$CETS1_Q6	C-ets-1	ETS1	0.222682	91
V$SMAD3_Q6_01	Smad3	SMAD3	0.119538	90
V$GABPA_Q4	GABP-alpha	GABPA	0.38426	86
V$SOX5_01	SOX5	SOX5	0.220517	86
V$TBX5_01	TBX5	TBX5	0.0245151	85

V$HNF4A_Q6_01	HNF-4alpha	HNF4A	0.616141	83
V$DLX5_01	dlx5	DLX5	0.0106439	79
V$MYOD_Q6_01	MyoD	MYOD1	0.227871	79
V$PITX3_Q2	PITX3	PITX3	0.129216	78
V$SPI1_Q5	SPI1	SPI1	0.0707982	74
V$CDX2_Q5_01	Cdx-2	CDX2	0.12792	73
V$ING4_01	ING4	ING4	0.0931472	73
V$CDX2_01	Cdx-2	CDX2	0.12792	73
V$MYOGENIN_Q6	myogenin	MYOG	0.202626	71
V$LRF_Q2	LRF	ZBTB7A	0.147518	68
V$CRX_Q4	Crx	CRX	0.346269	68
V$TEF1_Q6_03	TEF-1	TEAD1	0.00561956	64
V$HNF3B_Q6	HNF-3beta	FOXA2	0.236636	63
V$TCF3_01	TCF-3	TCF7L1	0.397798	62
V$TEF1_Q6	TEF-1	TEAD1	0.00561956	58
V$ERR1_Q3	ERR1	ESRRA	0.211782	57
V$CMAF_01	c-Maf	MAF	0.490326	57
V$HNF3A_01	HNF3A	FOXA1	0.111148	54
V$MYOGENIN_Q6_01	myogenin	MYOG	0.202626	52
V$CRX_02	Crx	CRX	0.346269	52
V$MYOD_Q6	MyoD	MYOD1	0.227871	49
V$NKX2B_Q3	NKX2B	NKX2-2	0.0238043	48
V$SMAD3_Q6	SMAD3	SMAD3	0.119538	47
V$ELF5_01	ELF5	ELF5	0.155551	47
V$FOXP3_01	FOXP3	FOXP3	0.062356	46
V$NKX22_02	NKX2B	NKX2-2	0.0238043	45
V$MATH1_Q2	MATH1	ATOH1	0.294356	40
V$HOXA9_01	hoxa9	HOXA9	0.301727	40
V$PAX8_01	Pax-8	PAX8	0.128525	40
V$MYOD_Q6_02	MyoD	MYOD1	0.227871	38
V$MYOD_01	MyoD	MYOD1	0.227871	37
V$CDX1_01	Cdx-1	CDX1	0.374303	35
V$P53_02	p53	TP53	0.0126346	34
V$MITF_Q6	MITF	MITF	0.0671485	32
V$CDX2_Q5	Cdx-2	CDX2	0.12792	28
V$HOXD9_Q2	Hoxd9	HOXD9	0.0550375	28
V$ATF3_Q6_01	ATF-3	ATF3	0.0805224	28
V$EAR2_Q2	EAR2	NR2F6	0.514834	25
V$PAX3_B	Pax-3	PAX3	0.167833	15
V$RREB1_01	RREB-1	RREB1	0.0548464	14
V$ATF5_01	ATF5	ATF5	0.638786	14
V$STAT5A_Q6	STAT5A	STAT5A	0.00497811	9

----

hsa-mir-194-2

Matrix_id	transcription factor	Gene	PCC	Occurrence
V$MAFB_01	MAFB	MAFB	0.235128	109
V$ELF1_Q6	Elf-1	ELF1	0.268591	109
V$NR1B2_Q6	NR1B2	RARB	0.228664	107
V$ZIC3_01	Zic3	ZIC3	0.229358	106
V$TBX5_02	TBX5	TBX5	0.0245151	102
V$CETS1_Q6	C-ets-1	ETS1	0.222682	91
V$SMAD3_Q6_01	Smad3	SMAD3	0.119538	90
V$Elf5_03	ELF5	ELF5	0.155551	89
V$ETS1_B	c-Ets-1	ETS1	0.222682	87
V$GABPA_Q4	GABP-alpha	GABPA	0.38426	86
V$SOX5_01	SOX5	SOX5	0.220517	86
V$TBX5_01	TBX5	TBX5	0.0245151	85
V$HNF4A_Q6_01	HNF-4alpha	HNF4A	0.616141	83
V$ERBETA_Q5	ER-beta	ESR2	0.303082	81
V$DLX5_01	dlx5	DLX5	0.0106439	79
V$MYOD_Q6_01	MyoD	MYOD1	0.227871	79
V$PITX3_Q2	PITX3	PITX3	0.129216	78
V$SPI1_03	SPI1	SPI1	0.0707982	77
V$SPI1_Q5	SPI1	SPI1	0.0707982	74
V$ING4_01	ING4	ING4	0.0931472	73
V$MYOGENIN_Q6	myogenin	MYOG	0.202626	71
V$CRX_Q4	Crx	CRX	0.346269	68
V$TEF1_Q6_03	TEF-1	TEAD1	0.00561956	64
V$HNF3B_Q6	HNF-3beta	FOXA2	0.236636	63
V$TCF3_01	TCF-3	TCF7L1	0.397798	62
V$CEBPA_Q6	C/EBPalpha	CEBPA	0.668309	59
V$TEF1_Q6	TEF-1	TEAD1	0.00561956	58
V$ERR1_Q3	ERR1	ESRRA	0.211782	57
V$SREBP1_Q6	SREBP-1	SREBF1	0.0919328	55
V$HNF1_02	HNF-1alpha	HNF1A	0.401178	53
V$ETV3_02	ETV3	ETV3	0.318269	53
V$ETV7_01	ETV7	ETV7	0.0610358	52
V$OC2_Q3	OC-2	ONECUT2	0.146364	52
V$CRX_02	Crx	CRX	0.346269	52
V$MYOGENIN_Q6_01	myogenin	MYOG	0.202626	52
V$CEBPE_Q6	CEBPE	CEBPE	0.145397	52
V$ER71_02	ER71	ETV2	0.317594	52
V$MYOD_Q6	MyoD	MYOD1	0.227871	49
V$CP2_01	CP2	TFCP2	0.0443438	49
V$FKLF_Q5	FKLF	KLF11	0.24671	48
V$ESE1_Q3	ESE-1	ELF3	0.278426	47
V$EHF_03	EHF	EHF	0.00760882	46
V$CRX_Q4_01	CRX	CRX	0.346269	45

V$ERF_02	ERF	ERF	0.298374	41
V$MATH1_Q2	MATH1	ATOH1	0.294356	40
V$EGR1_02	EGR-1	EGR1	0.0176089	39
V$MYOD_Q6_02	MyoD	MYOD1	0.227871	38
V$STAT3_03	STAT3	STAT3	0.313471	38
V$MYOD_01	MyoD	MYOD1	0.227871	37
V$VDR_Q3	VDR	VDR	0.111962	36
V$ESE1_02	ESE-1	ELF3	0.278426	35
V$NCX_02	Ncx	TLX2	0.0447467	34
V$PDEF_02	PDEF	SPDEF	0.0290947	31
V$CART1_02	CART1	ALX1	0.122074	25
V$SOX2_Q6	SOX2	SOX2	0.519907	23
V$PET1_02	Pet-1	FEV	0.0516993	20
V$ERG_03	ERG	ERG	0.0692334	20
V$ERG_01	ERG	ERG	0.0692334	17
V$PAX3_B	Pax-3	PAX3	0.167833	15
V$HOXA7_01	HOXA7	HOXA7	0.525252	9
V$HEN1_02	HEN1	NHLH1	0.24943	6
V$HEN1_01	HEN1	NHLH1	0.24943	4

hsa-mir-196a-1

Matrix_id	transcription factor	Gene	PCC	Occurrence
V$PARP_Q4	PARP	PARP1	0.29521	18
V$AP2REP_01	AP-2rep	KLF12	0.370644	18
V$PEA3_Q6	PEA3	ETV4	0.33916	17
V$PUR1_Q4	PUR1	PURA	0.345507	17
V$GATA1_01	GATA-1	GATA1	0.0580835	17
V$CDX2_Q5_02	CDX-2	CDX2	0.475056	16
V$ETS1_B	c-Ets-1	ETS1	0.205573	16
V$DLX5_01	dlx5	DLX5	0.199298	16
V$TBP_Q6	TBP	TBP	0.216329	16
V$NFAT4_Q3	NF-AT4	NFATC3	0.261278	15
V$ETS2_Q6	c-Ets-2	ETS2	0.225616	15
V$MEF2C_Q4	MEF-2C	MEF2C	0.122383	15
V$CMYB_Q5	c-Myb	MYB	0.0527704	15
V$MYB_Q6	c-Myb	MYB	0.0527704	15
V$NR1B2_Q6	NR1B2	RARB	0.475451	15
V$P300_01	p300	EP300	0.424479	15
V$CETS1_Q6	C-ets-1	ETS1	0.205573	15
V$SMAD4_Q6_01	Smad4	SMAD4	0.500799	14
V$ZIC3_01	Zic3	ZIC3	0.562652	14
V$YY1_01	YY1	YY1	0.168675	14

V$IPF1_01	IPF1	PDX1	0.470566	14
V$GATA1_02	GATA-1	GATA1	0.0580835	14
V$PITX3_Q2	PITX3	PITX3	0.226267	14
V$Elf5_03	ELF5	ELF5	0.149021	14
V$CRX_Q4	Crx	CRX	0.473552	13
V$GFI1_Q6	Gfi1	GFI1	0.206615	13
V$SMAD3_Q6_01	Smad3	SMAD3	0.479328	13
V$HNF4A_Q6_01	HNF-4alpha	HNF4A	0.489039	13
V$SOX5_01	SOX5	SOX5	0.374336	13
V$GATA3_01	GATA-3	GATA3	0.0413238	13
V$GATA1_04	GATA-1	GATA1	0.0580835	13
V$SRY_02	SRY	SRY	0.399646	13
V$GABPA_Q4	GABP-alpha	GABPA	0.0585698	13
V$GR_Q6	GR	NR3C1	0.560868	13
V$PARP_Q3	PARP	PARP1	0.29521	13
V$YY1_Q6	YY1	YY1	0.168675	13
V$PBX1_04	Pbx1	PBX1	0.327322	12
V$GFI1_Q6_01	Gfi1	GFI1	0.206615	12
V$ETS2_B	c-Ets-2	ETS2	0.225616	12
V$TCF3_01	TCF-3	TCF7L1	0.499692	12
V$CEBPE_Q6	CEBPE	CEBPE	0.0736575	12
V$GATA1_06	GATA-1	GATA1	0.0580835	11
V$GATA1_05	GATA-1	GATA1	0.0580835	11
V$CIZ_01	CIZ	ZNF384	0.474889	11
V$CMYB_01	c-Myb	MYB	0.0527704	11
V$CDX2_Q5_01	Cdx-2	CDX2	0.475056	11
V$CDX2_01	Cdx-2	CDX2	0.475056	11
V$GATA2_02	GATA-2	GATA2	0.30476	11
V$GATA3_02	GATA-3	GATA3	0.0413238	11
V$CRX_Q4_01	CRX	CRX	0.473552	11
V$TBX5_02	TBX5	TBX5	0.27336	11
V$AP4_Q6_02	AP-4	TFAP4	0.358319	10
V$ERR1_Q3	ERR1	ESRRA	0.324647	10
V$TCF4_01	TCF-4	TCF7L2	0.045116	10
V$HNF1_02	HNF-1alpha	HNF1A	0.404818	10
V$YY1_Q6_03	YY1	YY1	0.168675	10
V$IRF4_Q6	IRF-4	IRF4	0.559725	10
V$NFAT2_Q5	NF-AT2	NFATC1	0.350808	10
V$IPF1_Q6	IPF1	PDX1	0.470566	9
V$PITX1_01	Pitx1	PITX1	0.428517	9
V$PBX1_Q3	Pbx1	PBX1	0.327322	9
V$IPF1_Q4_01	IPF1	PDX1	0.470566	9
V$YY1_Q6_02	YY1	YY1	0.168675	9
V$MYOGENIN_Q6_01	myogenin	MYOG	0.490776	8
V$BCL6_Q3_01	Bcl-6	BCL6	0.388452	8
V$PITX2_01	PITX2	PITX2	0.329188	8

V$IPF1_Q4	IPF1	PDX1	0.470566	8
V$SMAD3_Q6	SMAD3	SMAD3	0.479328	8
V$HNF3A_01	HNF3A	FOXA1	0.235549	8
V$GR_01	GR	NR3C1	0.560868	8
V$HOXA9_01	hoxa9	HOXA9	0.831662	8
V$RFX1_02	RFX1	RFX1	0.480903	8
V$CRX_02	Crx	CRX	0.473552	8
V$AP4_Q5	AP-4	TFAP4	0.358319	8
V$RFX1_01	RFX1	RFX1	0.480903	8
V$AP4_Q6	AP-4	TFAP4	0.358319	8
V$MEF2A_Q6	mef2A	MEF2A	0.108691	7
V$MSX1_01	Msx-1	MSX1	0.609175	7
V$TR4_Q2	TR4	NR2C2	0.453818	6
V$MATH1_Q2	MATH1	ATOH1	0.456671	6
V$NURR1_Q3	NURR1	NR4A2	0.0894904	6
V$FOXJ2_02	FOXJ2	FOXJ2	0.334262	6
V$HNF1B_01	HNF-1beta	HNF1B	0.7965	6
V$TEF1_Q6	TEF-1	TEAD1	0.476219	6
V$GATA3_03	GATA-3	GATA3	0.0413238	5
V$TEF1_Q6_03	TEF-1	TEAD1	0.476219	5
V$HNF6_Q6	HNF6	ONECUT1	0.133906	5
V$P53_02	p53	TP53	0.171675	5
V$STAT3_03	STAT3	STAT3	0.411935	5
V$EAR2_Q2	EAR2	NR2F6	0.105561	5
V$OC2_Q3	OC-2	ONECUT2	0.353174	4
V$PIT1_Q6	Pit-1	POU1F1	0.19872	4
V$NKX32_02	Nkx3-2	NKX3-2	0.433654	4
V$NKX32_01	Nkx3-2	NKX3-2	0.433654	4
V$STAT1_05	STAT1	STAT1	0.0832112	4
V$STAT4_Q5	STAT4	STAT4	0.434385	3
V$POU6F1_02	POU6F1	POU6F1	0.476432	3
V$IRF1_Q6_01	IRF-1	IRF1	0.138728	3
V$HBP1_Q2	hbp1	HBP1	0.328796	3
V$POU6F1_03	POU6F1	POU6F1	0.476432	3
V$RSRFC4_Q2	RSRFC4	MEF2A	0.108691	3
V$STAT1_Q6	STAT1	STAT1	0.0832112	3
V$FAC1_01	FAC1	BPTF	0.256198	3
V$YY1_03	YY1	YY1	0.168675	2
V$NANOG_01	Nanog	NANOG	0.482623	2
V$RSRFC4_01	RSRFC4	MEF2A	0.108691	2
V$POU3F2_02	POU3F2	POU3F2	0.323801	2
V$BACH2_01	Bach2	BACH2	0.479248	1

----

hsa-mir-196a-2

Matrix_id	transcription factor	Gene	PCC	Occurrence
V$PARP_Q4	PARP	PARP1	0.29521	18
V$IK_Q5	Ikaros	IKZF1	0.38432	18
V$PEA3_Q6	PEA3	ETV4	0.33916	17
V$PUR1_Q4	PUR1	PURA	0.345507	17
V$NANOG_02	Nanog	NANOG	0.482623	17
V$GATA1_01	GATA-1	GATA1	0.0580835	17
V$TBP_Q6	TBP	TBP	0.216329	16
V$DLX5_01	dlx5	DLX5	0.199298	16
V$CDX2_Q5_02	CDX-2	CDX2	0.475056	16
V$ETS1_B	c-Ets-1	ETS1	0.205573	16
V$CETS1_Q6	C-ets-1	ETS1	0.205573	15
V$ETS2_Q6	c-Ets-2	ETS2	0.225616	15
V$NFAT4_Q3	NF-AT4	NFATC3	0.261278	15
V$NR1B2_Q6	NR1B2	RARB	0.475451	15
V$P300_01	p300	EP300	0.424479	15
V$Elf5_03	ELF5	ELF5	0.149021	14
V$YY1_01	YY1	YY1	0.168675	14
V$ZIC3_01	Zic3	ZIC3	0.562652	14
V$SMAD4_Q6_01	Smad4	SMAD4	0.500799	14
V$IPF1_01	IPF1	PDX1	0.470566	14
V$YY1_Q6	YY1	YY1	0.168675	13
V$HNF4A_Q6_01	HNF-4alpha	HNF4A	0.489039	13
V$GR_Q6	GR	NR3C1	0.560868	13
V$SMAD3_Q6_01	Smad3	SMAD3	0.479328	13
V$GABPA_Q4	GABP-alpha	GABPA	0.0585698	13
V$PARP_Q3	PARP	PARP1	0.29521	13
V$ETS2_B	c-Ets-2	ETS2	0.225616	12
V$PBX1_04	Pbx1	PBX1	0.327322	12
V$SOX10_Q6	SOX10	SOX10	0.101599	12
V$ELK1_02	Elk-1	ELK1	0.34349	11
V$CP2_01	CP2	TFCP2	0.218464	11
V$IRF8_Q6	IRF-8	IRF8	0.139038	11
V$GATA1_05	GATA-1	GATA1	0.0580835	11
V$CDX2_Q5_01	Cdx-2	CDX2	0.475056	11
V$TEL1_02	TEL1	ETV6	0.347328	11
V$CMYB_01	c-Myb	MYB	0.0527704	11
V$HNF3B_Q6	HNF-3beta	FOXA2	0.080954	11
V$GATA2_02	GATA-2	GATA2	0.30476	11
V$GATA1_06	GATA-1	GATA1	0.0580835	11
V$HNF1_02	HNF-1alpha	HNF1A	0.404818	10
V$BEN_01	BEN	GTF2IRD1	0.409773	10
V$SP1_Q6	Sp1	SP1	0.256793	10
V$FOXM1_01	FOXM1	FOXM1	0.0327286	10

V$YY1_Q6_03	YY1	YY1	0.168675	10
V$FOXO3A_Q1	FOXO3A	FOXO3	0.350279	10
V$AP2ALPHA_01	AP-2alpha	TFAP2A	0.0481473	10
V$AML1_Q6	AML1	RUNX1	0.192676	10
V$SP1_Q6_01	Sp1	SP1	0.256793	10
V$ZBP89_Q4	ZBP89	ZNF148	0.421641	10
V$AP4_Q6_02	AP-4	TFAP4	0.358319	10
V$ERBETA_Q5	ER-beta	ESR2	0.380733	10
V$NFAT2_Q5	NF-AT2	NFATC1	0.350808	10
V$LRF_Q2	LRF	ZBTB7A	0.401618	10
V$LHX3b_01	LHX3b	LHX3	0.363009	9
V$FOXP3_01	FOXP3	FOXP3	0.527612	9
V$ETV3_02	ETV3	ETV3	0.0450707	9
V$AP2ALPHA_Q6	AP-2alpha	TFAP2A	0.0481473	9
V$ER71_02	ER71	ETV2	0.465185	9
V$DAX1_01	Dax1	NR0B1	0.191542	9
V$MYOGENIN_Q6	myogenin	MYOG	0.490776	9
V$SP1_Q4_01	Sp1	SP1	0.256793	9
V$FOXJ2_01	FOXJ2	FOXJ2	0.334262	9
V$YY1_Q6_02	YY1	YY1	0.168675	9
V$IPF1_06	ipf1	PDX1	0.470566	8
V$WT1_Q6_01	WT1	WT1	0.532888	8
V$RFX1_01	RFX1	RFX1	0.480903	8
V$SP1_Q2_01	Sp1	SP1	0.256793	8
V$AP2GAMMA_01	AP-2gamma	TFAP2C	0.0835625	8
V$CREM_Q6	CREM	CREM	0.122999	8
V$RFX1_02	RFX1	RFX1	0.480903	8
V$AP4_Q6	AP-4	TFAP4	0.358319	8
V$MYOGENIN_Q6_01	myogenin	MYOG	0.490776	8
V$CART1_02	CART1	ALX1	0.52064	8
V$IPF1_03	IPF1	PDX1	0.470566	8
V$ETV7_01	ETV7	ETV7	0.454921	8
V$HOX13_02	HOXA5	HOXA5	0.0385795	8
V$AP4_Q5	AP-4	TFAP4	0.358319	8
V$EGR1_02	EGR-1	EGR1	0.295031	7
V$SP1_02	SP1	SP1	0.256793	7
V$AP4_Q6_01	AP-4	TFAP4	0.358319	7
V$MAZ_Q6	MAZ	MAZ	0.0900641	7
V$MAZ_Q6_01	MAZ	MAZ	0.0900641	6
V$MITF_Q6	MITF	MITF	0.728963	6
V$EHF_03	EHF	EHF	0.0979391	6
V$NURR1_Q3	NURR1	NR4A2	0.0894904	6
V$MATH1_Q2	MATH1	ATOH1	0.456671	6
V$WT1_Q6	WT1	WT1	0.532888	6
V$ATF1_Q6_01	ATF-1	ATF1	0.264549	6
V$ESE1_Q3	ESE-1	ELF3	0.197042	6

V$CNOT3_01	CNOT3	CNOT3	0.413318	5
V$ZFX_01	Zfx	ZFX	0.441189	5
V$MYOD_Q6_02	MyoD	MYOD1	0.379909	5
V$KLF15_Q2	KLF15	KLF15	0.0538967	5
V$PDEF_02	PDEF	SPDEF	0.1032	5
V$GATA3_03	GATA-3	GATA3	0.0413238	5
V$TFIII_Q6	TFII-I	GTF2I	0.389862	5
V$AP2BETA_Q3	AP-2beta	TFAP2B	0.549085	5
V$ATF3_Q6_01	ATF-3	ATF3	0.20822	4
V$YY1_02	YY1	YY1	0.168675	4
V$ATF4_Q6	ATF-4	ATF4	0.29356	4
V$NKX32_01	Nkx3-2	NKX3-2	0.433654	4
V$BEN_02	BEN	GTF2IRD1	0.409773	4
V$RORBETA_Q2	RORBETA	RORB	0.270586	4
V$VDR_Q3	VDR	VDR	0.462669	4
V$E2F1_Q6	E2F-1	E2F1	0.378501	4
V$E2F1_Q3	E2F-1	E2F1	0.378501	4
V$E2F4_Q6	E2F-4	E2F4	0.278213	3
V$ATF2_Q5	ATF-2	ATF2	0.445493	3
V$SPIB_01	SPI-B	SPIB	0.146831	3
V$RREB1_01	RREB-1	RREB1	0.356807	3
V$SPIB_03	Spi-B	SPIB	0.146831	3
V$PAX3_B	Pax-3	PAX3	0.148743	3
V$E4F1_Q6_01	E4F1	E4F1	0.0145789	2
V$NANOG_01	Nanog	NANOG	0.482623	2
V$YY1_03	YY1	YY1	0.168675	2
V$ELK1_01	Elk-1	ELK1	0.34349	1

----

hsa-mir-196b

Matrix_id	transcription factor	Gene	PCC	Occurrence
V$GATA2_02	GATA-2	GATA2	0.531924	6
V$GATA1_05	GATA-1	GATA1	0.121931	6
V$GATA1_06	GATA-1	GATA1	0.121931	6
V$GKLF_Q4	GKLF	KLF4	0.0695809	6
V$CMYB_Q5	c-Myb	MYB	0.064782	6
V$PUR1_Q4	PUR1	PURA	0.552476	6
V$ZIC3_01	Zic3	ZIC3	0.152285	6
V$PBX1_04	Pbx1	PBX1	0.565614	6
V$PARP_Q3	PARP	PARP1	0.00011251	6
V$PEA3_Q6	PEA3	ETV4	0.0261107	6
V$IK_Q5	Ikaros	IKZF1	0.0298819	6
V$WT1_Q6	WT1	WT1	0.607949	6

V$ELF1_Q6	Elf-1	ELF1	0.23711	6
V$MYB_Q6	c-Myb	MYB	0.064782	6
V$SMAD3_Q6_01	Smad3	SMAD3	0.155295	6
V$PARP_Q4	PARP	PARP1	0.00011251	6
V$HNF4A_Q6_01	HNF-4alpha	HNF4A	0.041274	6
V$SMAD4_Q6_01	Smad4	SMAD4	0.478239	6
V$NR1B2_Q6	NR1B2	RARB	0.123795	6
V$NFAT4_Q3	NF-AT4	NFATC3	0.164295	5
V$SOX5_01	SOX5	SOX5	0.0410747	5
V$P300_01	p300	EP300	0.210786	5
V$NKX32_01	Nkx3-2	NKX3-2	0.141552	5
V$SP1_01	Sp1	SP1	0.0948142	5
V$TFIII_Q6	TFII-I	GTF2I	0.189732	5
V$CMYB_01	c-Myb	MYB	0.064782	5
V$CDX2_Q5_02	CDX-2	CDX2	0.0779083	5
V$TBP_Q6	TBP	TBP	0.0810398	5
V$AP2ALPHA_Q6	AP-2alpha	TFAP2A	0.182437	5
V$GATA1_02	GATA-1	GATA1	0.121931	5
V$FKLF_Q5	FKLF	KLF11	0.0407377	5
V$GATA1_01	GATA-1	GATA1	0.121931	5
V$CDX2_Q5_01	Cdx-2	CDX2	0.0779083	4
V$AP2BETA_Q3	AP-2beta	TFAP2B	0.155885	4
V$IRF4_Q6	IRF-4	IRF4	0.179097	4
V$LRF_Q2	LRF	ZBTB7A	0.11217	4
V$YY1_Q6_02	YY1	YY1	0.367346	4
V$NKX32_02	Nkx3-2	NKX3-2	0.141552	4
V$YY1_Q6	YY1	YY1	0.367346	4
V$GR_Q6	GR	NR3C1	0.585482	4
V$VDR_Q3	VDR	VDR	0.0510964	4
V$AP2ALPHA_01	AP-2alpha	TFAP2A	0.182437	4
V$PITX2_Q2	Pitx2	PITX2	0.21196	4
V$CRX_Q4	Crx	CRX	0.00887493	4
V$SRY_02	SRY	SRY	0.286663	4
V$GATA3_01	GATA-3	GATA3	0.27421	4
V$YY1_01	YY1	YY1	0.367346	4
V$E47_02	E47	TCF3	0.103691	3
V$E2A_Q6	E2A	TCF3	0.103691	3
V$GATA2_01	GATA-2	GATA2	0.531924	3
V$MYOD_Q6_01	MyoD	MYOD1	0.0114064	3
V$CRX_Q4_01	CRX	CRX	0.00887493	3
V$MYOD_01	MyoD	MYOD1	0.0114064	3
V$PITX1_01	Pitx1	PITX1	0.124336	3
V$E2A_Q2	E2A	TCF3	0.103691	3
V$PITX2_01	PITX2	PITX2	0.21196	3
V$E12_Q6	E12	TCF3	0.103691	3
V$YY1_02	YY1	YY1	0.367346	3

V$GATA1_04	GATA-1	GATA1	0.121931	3
V$MYOGENIN_Q6	myogenin	MYOG	0.0899556	3
V$PBX1_Q3	Pbx1	PBX1	0.565614	3
V$CRX_02	Crx	CRX	0.00887493	3
V$IRF7_Q3	IRF-7	IRF7	0.0635214	2
V$MYOD_Q6	MyoD	MYOD1	0.0114064	2
V$TCF3_01	TCF-3	TCF7L1	0.101706	2
V$HBP1_Q2	hbp1	HBP1	0.347048	2
V$IPF1_Q6	IPF1	PDX1	0.118723	2
V$TEF1_Q6_03	TEF-1	TEAD1	0.159093	2
V$TEF1_Q6	TEF-1	TEAD1	0.159093	2
V$CREM_Q6	CREM	CREM	0.0794977	2
V$E47_01	E47	TCF3	0.103691	2
V$IPF1_Q4_01	IPF1	PDX1	0.118723	2
V$ZABC1_01	ZABC1	ZNF217	0.217763	2
V$NANOG_01	Nanog	NANOG	0.154934	1
V$GR_01	GR	NR3C1	0.585482	1
V$HNF6_Q6	HNF6	ONECUT1	0.03058	1
V$E2F1_Q4	E2F-1	E2F1	0.0705747	1
V$ATF4_Q6	ATF-4	ATF4	0.0624708	1
V$ATF3_Q6_01	ATF-3	ATF3	0.16614	1
V$ATF2_Q5	ATF-2	ATF2	0.181426	1
V$ATF1_Q6_01	ATF-1	ATF1	0.291589	1
V$IPF1_Q4	IPF1	PDX1	0.118723	1
V$PITX1_Q6	PITX1	PITX1	0.124336	1
V$YY1_Q6_03	YY1	YY1	0.367346	1

----

hsa-mir-197

Matrix_id	transcription factor	Gene	PCC	Occurrence
V$PUR1_Q4	PUR1	PURA	0.297783	78
V$ZIC3_01	Zic3	ZIC3	0.109088	76
V$MAFB_01	MAFB	MAFB	0.129848	76
V$CETS1_Q6	C-ets-1	ETS1	0.0391643	72
V$PARP_Q4	PARP	PARP1	0.303089	70
V$AP4_Q6_02	AP-4	TFAP4	0.344389	60
V$TBP_Q6	TBP	TBP	0.0196647	60
V$SOX9_Q4	SOX9	SOX9	0.205447	56
V$YY1_Q6_02	YY1	YY1	0.0151553	53
V$YY1_01	YY1	YY1	0.0151553	52
V$SOX9_B1	SOX9	SOX9	0.205447	49
V$MEF2C_Q4	MEF-2C	MEF2C	0.583536	47
V$GATA1_02	GATA-1	GATA1	0.183713	46

V$AP4_Q5	AP-4	TFAP4	0.344389	44
V$SRY_02	SRY	SRY	0.119642	43
V$GATA6_01	GATA-6	GATA6	0.330473	41
V$GATA1_06	GATA-1	GATA1	0.183713	41
V$GATA1_05	GATA-1	GATA1	0.183713	41
V$PITX3_Q2	PITX3	PITX3	0.0936476	41
V$PARP_Q3	PARP	PARP1	0.303089	40
V$PBX1_Q3	Pbx1	PBX1	0.221783	38
V$AP4_Q6	AP-4	TFAP4	0.344389	37
V$GATA1_04	GATA-1	GATA1	0.183713	36
V$YY1_Q6_03	YY1	YY1	0.0151553	35
V$FOXJ2_01	FOXJ2	FOXJ2	0.0508024	34
V$IRF8_Q6	IRF-8	IRF8	0.00159862	34
V$PBX1_04	Pbx1	PBX1	0.221783	33
V$SOX10_Q6	SOX10	SOX10	0.621824	31
V$DAX1_01	Dax1	NR0B1	0.00164919	26
V$NKX2B_Q3	NKX2B	NKX2-2	0.645494	25
V$DBP_Q6_01	DBP	DBP	0.234283	22
V$PIT1_Q6	Pit-1	POU1F1	0.197699	22
V$LHX3b_01	LHX3b	LHX3	0.121585	22
V$OC2_Q3	OC-2	ONECUT2	0.0982846	21
V$AP4_Q6_01	AP-4	TFAP4	0.344389	20
V$HOX13_02	HOXA5	HOXA5	0.146798	19
V$MSX1_01	Msx-1	MSX1	0.0554121	18
V$CIZ_01	CIZ	ZNF384	0.0147559	15
V$NCX_02	Ncx	TLX2	0.0391919	13
V$LHX3_01	Lhx3	LHX3	0.121585	13
V$SOX2_Q6	SOX2	SOX2	0.223161	11
V$HSF1_01	HSF1	HSF1	0.257652	11
V$AMEF2_Q6	aMEF-2	MEF2A	0.493254	9
V$RSRFC4_Q2	RSRFC4	MEF2A	0.493254	4
V$RSRFC4_01	RSRFC4	MEF2A	0.493254	3

---

hsa-mir-198

Matrix_id	transcription factor	Gene	PCC	Occurrence
V$PUR1_Q4	PUR1	PURA	0.265489	238
V$PEA3_Q6	PEA3	ETV4	0.0643855	232
V$PARP_Q4	PARP	PARP1	0.0547425	229
V$ELF1_Q6	Elf-1	ELF1	0.229875	227
V$GKLF_Q4	GKLF	KLF4	0.56518	224
V$P300_01	p300	EP300	0.325138	212
V$ETS1_B	c-Ets-1	ETS1	0.100946	208

V$CETS1_Q6	C-ets-1	ETS1	0.100946	203
V$SMAD4_Q6_01	Smad4	SMAD4	0.321669	201
V$GABPA_Q4	GABP-alpha	GABPA	0.299109	193
V$GATA1_01	GATA-1	GATA1	0.0663318	191
V$TBP_Q6	TBP	TBP	0.123495	181
V$GR_Q6	GR	NR3C1	0.217691	180
V$YY1_01	YY1	YY1	0.419283	179
V$DLX5_01	dlx5	DLX5	0.616471	165
V$YY1_Q6	YY1	YY1	0.419283	162
V$YY1_Q6_02	YY1	YY1	0.419283	162
V$GATA1_02	GATA-1	GATA1	0.0663318	155
V$GATA2_02	GATA-2	GATA2	0.766715	152
V$GATA1_06	GATA-1	GATA1	0.0663318	152
V$GATA1_05	GATA-1	GATA1	0.0663318	152
V$ERBETA_Q5	ER-beta	ESR2	0.0165203	144
V$SP1_Q6	Sp1	SP1	0.318989	140
V$HMGIY_01	HMGIY	HMGA1	0.294196	132
V$GATA1_04	GATA-1	GATA1	0.0663318	130
V$FOXO3A_Q1	FOXO3A	FOXO3	0.00638242	125
V$GATA3_01	GATA-3	GATA3	0.923777	121
V$YY1_Q6_03	YY1	YY1	0.419283	120
V$GR_01	GR	NR3C1	0.217691	110
V$CEBPE_Q6	CEBPE	CEBPE	0.0279454	109
V$GATA2_01	GATA-2	GATA2	0.766715	108
V$PITX2_Q2	Pitx2	PITX2	0.424653	95
V$FOXJ2_01	FOXJ2	FOXJ2	0.0148145	94
V$PITX2_01	PITX2	PITX2	0.424653	92
V$GATA3_03	GATA-3	GATA3	0.923777	84
V$FAC1_01	FAC1	BPTF	0.0618355	82
V$HBP1_Q2	hbp1	HBP1	0.626253	82
V$CEBPG_Q6_01	C/EBPgamma	CEBPG	0.548857	76
V$HOXA9_01	hoxa9	HOXA9	0.022072	75
V$FOXO4_02	FOXO4	FOXO4	0.916441	65
V$CEBPB_Q6	C/EBPbeta	CEBPB	0.56084	65
V$ERR3_Q2	ERR3	ESRRG	0.456983	34
V$RORA1_01	RORalpha1	RORA	0.0240026	21
V$NKX3A_02	Nkx3A	NKX3-1	0.113294	17

---

hsa-mir-199a-1

Matrix_id	transcription factor	Gene	PCC	Occurrence
V$SP1_01	Sp1	SP1	0.0288378	2
V$TCF4_Q5	TCF-4	TCF7L2	0.337215	2

V$SMAD4_Q6_01	Smad4	SMAD4	0.0512127	2
V$ESE1_Q3	ESE-1	ELF3	0.10927	2
V$YY1_Q6	YY1	YY1	0.0233436	2
V$SP1_Q6_01	Sp1	SP1	0.0288378	2
V$SP1_Q4_01	Sp1	SP1	0.0288378	2
V$SP1_Q2_01	Sp1	SP1	0.0288378	2
V$YY1_Q6_02	YY1	YY1	0.0233436	2
V$DLX5_01	dlx5	DLX5	0.60436	2
V$GKLF_Q4	GKLF	KLF4	0.376316	2
V$SP1_02	SP1	SP1	0.0288378	2
V$YY1_01	YY1	YY1	0.0233436	2
V$ESE1_02	ESE-1	ELF3	0.10927	2
V$CEBPB_02	C/EBPbeta	CEBPB	0.223123	2
V$SP1_Q6	Sp1	SP1	0.0288378	2
V$WT1_Q6_01	WT1	WT1	0.154799	1
V$ZABC1_01	ZABC1	ZNF217	0.598131	1
V$ERM_02	Erm	ETV5	0.404524	1
V$STAT3_03	STAT3	STAT3	0.0148945	1
V$FKLF_Q5	FKLF	KLF11	0.171588	1
V$GABPA_Q4	GABP-alpha	GABPA	0.0112698	1
V$ERG_03	ERG	ERG	0.0493006	1
V$GABPBETA_Q3	GABP-beta	GABPB1	0.157046	1
V$ERG_01	ERG	ERG	0.0493006	1
V$HIF1A_Q6	HIF-1alpha	HIF1A	0.276095	1
V$AP2ALPHA_Q6	AP-2alpha	TFAP2A	0.601689	1
V$AHR_Q5	AhR	AHR	0.593301	1
V$YY1_02	YY1	YY1	0.0233436	1
V$CP2_01	CP2	TFCP2	0.263141	1
V$ARNT_01	Arnt	ARNT	0.057921	1
V$MEIS1_01	MEIS1	MEIS1	0.20358	1
V$AP2ALPHA_01	AP-2alpha	TFAP2A	0.601689	1
V$AP2GAMMA_01	AP-2gamma	TFAP2C	0.515526	1
V$FOXO4_02	FOXO4	FOXO4	0.628296	1
V$PITX2_Q2	Pitx2	PITX2	0.165026	1
V$PEA3_Q6	PEA3	ETV4	0.0972482	1
V$SMAD4_Q6	SMAD4	SMAD4	0.0512127	1
V$PR_01	PR	PGR	0.0443029	1
V$WT1_Q6	WT1	WT1	0.154799	1
V$CMYC_02	c-Myc	MYC	0.377521	1
V$RNF96_01	RNF96	TRIM28	0.109688	1
V$P300_01	p300	EP300	0.02017	1

----

hsa-mir-199a-2

Matrix_id	transcription factor	Gene	PCC	Occurrence
V$SP1_01	Sp1	SP1	0.0288378	2
V$YY1_Q6	YY1	YY1	0.0233436	2
V$TBP_Q6	TBP	TBP	0.115171	2
V$YY1_Q6_02	YY1	YY1	0.0233436	2
V$DLX5_01	dlx5	DLX5	0.60436	2
V$HOX13_02	HOXA5	HOXA5	0.182462	2
V$HBP1_Q2	hbp1	HBP1	0.470124	2
V$GKLF_Q4	GKLF	KLF4	0.376316	2
V$SMAD4_Q6_01	Smad4	SMAD4	0.0512127	2
V$HNF6_Q6	HNF6	ONECUT1	0.0228649	2
V$CEBPG_Q6	C/EBPgamma	CEBPG	0.233682	2
V$YY1_01	YY1	YY1	0.0233436	2
V$GATA3_01	GATA-3	GATA3	0.575135	2
V$GATA2_02	GATA-2	GATA2	0.35789	2
V$GATA3_02	GATA-3	GATA3	0.575135	2
V$GATA3_03	GATA-3	GATA3	0.575135	2
V$GATA6_01	GATA-6	GATA6	0.150895	2
V$NURR1_Q3	NURR1	NR4A2	0.0398462	1
V$E4BP4_01	E4BP4	NFIL3	0.190009	1
V$GABPBETA_Q3	GABP-beta	GABPB1	0.157046	1
V$CEBPG_Q6_01	C/EBPgamma	CEBPG	0.233682	1
V$PEA3_Q6	PEA3	ETV4	0.0972482	1
V$FOXO4_02	FOXO4	FOXO4	0.628296	1
V$GABPA_Q4	GABP-alpha	GABPA	0.0112698	1
V$PBX1_Q3	Pbx1	PBX1	0.105705	1

hsa-mir-199b

Matrix_id	transcription factor	Gene	PCC	Occurrence
V$SOX9_Q4	SOX9	SOX9	0.106406	19
V$PUR1_Q4	PUR1	PURA	0.155025	19
V$SMAD4_Q6_01	Smad4	SMAD4	0.248241	17
V$TBP_Q6	TBP	TBP	0.0170777	17
V$PEA3_Q6	PEA3	ETV4	0.145903	17
V$YY1_01	YY1	YY1	0.359415	16
V$ELF1_Q6	Elf-1	ELF1	0.331698	16
V$GR_Q6	GR	NR3C1	0.166492	15
V$DLX5_01	dlx5	DLX5	0.482213	15
V$NFAT4_Q3	NF-AT4	NFATC3	0.0446252	15
V$AML1_Q6	AML1	RUNX1	0.0478708	14
V$GATA2_02	GATA-2	GATA2	0.632944	14

V$GATA3_02	GATA-3	GATA3	0.746675	12
V$YY1_Q6	YY1	YY1	0.359415	12
V$YY1_Q6_03	YY1	YY1	0.359415	12
V$HMGIY_01	HMGIY	HMGA1	0.338787	11
V$HBP1_Q2	hbp1	HBP1	0.574933	11
V$ATF1_Q6_01	ATF-1	ATF1	0.108842	11
V$YY1_Q6_02	YY1	YY1	0.359415	11
V$SOX9_B1	SOX9	SOX9	0.106406	11
V$CREM_Q6	CREM	CREM	0.0812939	11
V$GR_01	GR	NR3C1	0.166492	10
V$FOXO4_02	FOXO4	FOXO4	0.73271	10
V$ATF3_Q6_01	ATF-3	ATF3	0.588162	9
V$PITX2_Q2	Pitx2	PITX2	0.329312	9
V$CEBPG_Q6_01	C/EBPgamma	CEBPG	0.461827	8
V$GATA3_01	GATA-3	GATA3	0.746675	8
V$TCF4_01	TCF-4	TCF7L2	0.566643	8
V$GATA2_01	GATA-2	GATA2	0.632944	8
V$TCF4_Q5	TCF-4	TCF7L2	0.566643	8
V$IRF7_Q3	IRF-7	IRF7	0.162963	7
V$PITX2_01	PITX2	PITX2	0.329312	7
V$AML1_01	AML1a	RUNX1	0.0478708	6
V$IRF1_Q6_01	IRF-1	IRF1	0.282213	4
V$CDP_04	CDP	CUX1	0.431369	3
V$ERR3_Q2	ERR3	ESRRG	0.196915	2

hsa-mir-200a

Matrix_id	transcription factor	Gene	PCC	Occurrence
V$ELF1_Q6	Elf-1	ELF1	0.323229	3
V$AP4_Q6_02	AP-4	TFAP4	0.095509	3
V$HNF4A_Q6_01	HNF-4alpha	HNF4A	0.0804495	3
V$SOX9_B1	SOX9	SOX9	0.268761	3
V$SOX9_Q4	SOX9	SOX9	0.268761	3
V$STAT3_03	STAT3	STAT3	0.205146	2
V$Elf5_03	ELF5	ELF5	0.360785	2
V$AP4_Q6_01	AP-4	TFAP4	0.095509	2
V$MYOGENIN_Q6_01	myogenin	MYOG	0.0447721	2
V$MYOGENIN_Q6	myogenin	MYOG	0.0447721	2
V$ZIC3_01	Zic3	ZIC3	0.122719	2
V$VDR_Q3	VDR	VDR	0.0421708	2
V$AP4_Q6	AP-4	TFAP4	0.095509	2
V$AP4_Q5	AP-4	TFAP4	0.095509	2
V$EGR1_02	EGR-1	EGR1	0.428292	1

V$ATF3_Q6_01	ATF-3	ATF3	0.256995	1
V$KLF15_Q2	KLF15	KLF15	0.172011	1
V$HOXA7_01	HOXA7	HOXA7	0.00626069	1
V$AP4_01	AP-4	TFAP4	0.095509	1

---

hsa-mir-200b

Matrix_id	transcription factor	Gene	PCC	Occurrence
V$TCF3_01	TCF-3	TCF7L1	0.050646	7
V$SOX9_Q4	SOX9	SOX9	0.47023	7
V$PUR1_Q4	PUR1	PURA	0.160423	7
V$GKLF_Q4	GKLF	KLF4	0.221354	7
V$AP4_Q6_02	AP-4	TFAP4	0.178565	7
V$AML1_Q6	AML1	RUNX1	0.00442887	7
V$ELF1_Q6	Elf-1	ELF1	0.613365	7
V$AP4_Q5	AP-4	TFAP4	0.178565	7
V$MYOGENIN_Q6	myogenin	MYOG	0.0201815	6
V$HNF4A_Q6_01	HNF-4alpha	HNF4A	0.00160818	6
V$SMAD3_Q6_01	Smad3	SMAD3	0.0790932	6
V$GR_Q6	GR	NR3C1	0.117797	6
V$AP4_Q6	AP-4	TFAP4	0.178565	6
V$Elf5_03	ELF5	ELF5	0.671401	6
V$MYOGENIN_Q6_01	myogenin	MYOG	0.0201815	5
V$YY1_Q6	YY1	YY1	0.0880359	5
V$HIF1A_Q6	HIF-1alpha	HIF1A	0.0695415	5
V$SOX9_B1	SOX9	SOX9	0.47023	5
V$TFIII_Q6	TFII-I	GTF2I	0.0250323	5
V$PBX1_Q3	Pbx1	PBX1	0.107605	4
V$ZABC1_01	ZABC1	ZNF217	0.0687805	4
V$AML2_Q3_01	AML2	RUNX3	0.0832866	4
V$AML2_Q3	AML2	RUNX3	0.0832866	4
V$AP4_Q6_01	AP-4	TFAP4	0.178565	3
V$HNF3A_01	HNF3A	FOXA1	0.318366	3
V$EGR1_02	EGR-1	EGR1	0.518484	3
V$STAT3_03	STAT3	STAT3	0.25257	3
V$ATF3_Q6_01	ATF-3	ATF3	0.332212	2
V$ATF1_Q6_01	ATF-1	ATF1	0.107667	2
V$KLF15_Q2	KLF15	KLF15	0.255104	2
V$AP4_01	AP-4	TFAP4	0.178565	2
V$E2F4_Q6	E2F-4	E2F4	0.0477645	1

---

hsa-mir-200c

Matrix_id	transcription factor	Gene	PCC	Occurrence
V$PUR1_Q4	PUR1	PURA	0.194482	32
V$SOX9_Q4	SOX9	SOX9	0.147116	31
V$GKLF_Q4	GKLF	KLF4	0.77484	31
V$PEA3_Q6	PEA3	ETV4	0.0686406	30
V$MAFB_01	MAFB	MAFB	0.346045	29
V$P300_01	p300	EP300	0.238027	29
V$GR_Q6	GR	NR3C1	0.160291	25
V$DLX5_01	dlx5	DLX5	0.476448	25
V$YY1_01	YY1	YY1	0.38971	25
V$SMAD4_Q6_01	Smad4	SMAD4	0.221659	25
V$TTF1_Q5	TTF-1	NKX2-1	0.0776565	24
V$SMAD3_Q6_01	Smad3	SMAD3	0.00368811	23
V$SOX9_B1	SOX9	SOX9	0.147116	23
V$AML1_Q6	AML1	RUNX1	0.0405414	21
V$AHR_Q5	AhR	AHR	0.838178	19
V$SP1_Q6	Sp1	SP1	0.118064	19
V$ARNT_01	Arnt	ARNT	0.125648	19
V$SP1_Q6_01	Sp1	SP1	0.118064	18
V$SP1_01	Sp1	SP1	0.118064	18
V$SP1_Q4_01	Sp1	SP1	0.118064	18
V$FOXJ2_01	FOXJ2	FOXJ2	0.0284769	18
V$SP1_Q2_01	Sp1	SP1	0.118064	17
V$TCF4_01	TCF-4	TCF7L2	0.642702	16
V$AP4_Q6_02	AP-4	TFAP4	0.00305426	16
V$AP2ALPHA_Q6	AP-2alpha	TFAP2A	0.727961	16
V$CEBPD_Q6	C/EBPdelta	CEBPD	0.486869	16
V$PITX2_Q2	Pitx2	PITX2	0.278717	15
V$AP2ALPHA_01	AP-2alpha	TFAP2A	0.727961	14
V$AML1_01	AML1a	RUNX1	0.0405414	14
V$GR_01	GR	NR3C1	0.160291	14
V$TCF4_Q5	TCF-4	TCF7L2	0.642702	14
V$FKLF_Q5	FKLF	KLF11	0.129642	13
V$CEBPB_Q6	C/EBPbeta	CEBPB	0.446757	13
V$PITX2_01	PITX2	PITX2	0.278717	13
V$SREBP1_Q6	SREBP-1	SREBF1	0.00619386	13
V$AP4_Q5	AP-4	TFAP4	0.00305426	12
V$SMAD3_Q6	SMAD3	SMAD3	0.00368811	12
V$AP2GAMMA_01	AP-2gamma	TFAP2C	0.733551	12
V$AML1_Q4	AML1	RUNX1	0.0405414	12
V$EGR1_02	EGR-1	EGR1	0.0590773	11
V$NURR1_Q3	NURR1	NR4A2	0.015698	11
V$ELF5_01	ELF5	ELF5	0.262205	9

V$RNF96_01	RNF96	TRIM28	0.189718	9
V$CNOT3_01	CNOT3	CNOT3	0.0279016	9
V$STAT1_05	STAT1	STAT1	0.243167	7
V$EGR2_01	Egr-2	EGR2	0.156615	6
V$GRE_C	GR	NR3C1	0.160291	5
V$SMAD4_Q6	SMAD4	SMAD4	0.221659	4
V$EGR1_01	Egr-1	EGR1	0.0590773	4
V$XBP1_01	XBP-1	XBP1	0.573719	3
V$BACH1_01	Bach1	BACH1	0.0419683	2

----

hsa-mir-202

Matrix_id	transcription factor	Gene	PCC	Occurrence
V$AP2REP_01	AP-2rep	KLF12	0.1073	298
V$GKLF_Q4	GKLF	KLF4	0.105036	295
V$PEA3_Q6	PEA3	ETV4	0.120687	291
V$PARP_Q4	PARP	PARP1	0.176953	291
V$ZIC3_01	Zic3	ZIC3	0.313953	264
V$TBP_Q6	TBP	TBP	0.784523	262
V$SOX9_Q4	SOX9	SOX9	0.606214	254
V$NFAT4_Q3	NF-AT4	NFATC3	0.0431241	251
V$GATA1_01	GATA-1	GATA1	0.080529	250
V$CETS1_Q6	C-ets-1	ETS1	0.0746848	247
V$ETS1_B	c-Ets-1	ETS1	0.0746848	243
V$GABPA_Q4	GABP-alpha	GABPA	0.00464486	239
V$DLX5_01	dlx5	DLX5	0.312612	233
V$HNF4A_Q6_01	HNF-4alpha	HNF4A	0.0407157	233
V$SOX5_01	SOX5	SOX5	0.582474	222
V$YY1_Q6	YY1	YY1	0.138611	221
V$AML1_Q6	AML1	RUNX1	0.143848	221
V$SOX9_B1	SOX9	SOX9	0.606214	221
V$YY1_Q6_02	YY1	YY1	0.138611	212
V$GATA6_01	GATA-6	GATA6	0.105333	205
V$GATA1_05	GATA-1	GATA1	0.080529	205
V$GATA1_06	GATA-1	GATA1	0.080529	205
V$PITX3_Q2	PITX3	PITX3	0.135303	196
V$SRY_02	SRY	SRY	0.0733843	194
V$ERBETA_Q5	ER-beta	ESR2	0.238874	191
V$E2A_Q6	E2A	TCF3	0.370986	191
V$E47_02	E47	TCF3	0.370986	191
V$E12_Q6	E12	TCF3	0.370986	191
V$ING4_01	ING4	ING4	0.0491314	181
V$HMGIY_01	HMGIY	HMGA1	0.569455	181

V$ELK1_02	Elk-1	ELK1	0.301088	177
V$HNF3B_Q6	HNF-3beta	FOXA2	0.00766143	174
V$ELK1_06	ELK-1	ELK1	0.301088	167
V$HIF1A_Q6	HIF-1alpha	HIF1A	0.205907	161
V$TEF1_Q6_03	TEF-1	TEAD1	0.0195563	138
V$FOXP3_01	FOXP3	FOXP3	0.0255528	134
V$OC2_Q3	OC-2	ONECUT2	0.0339969	131
V$TCF4_01	TCF-4	TCF7L2	0.0217157	128
V$TEF1_Q6	TEF-1	TEAD1	0.0195563	127
V$E47_01	E47	TCF3	0.370986	107
V$PDEF_02	PDEF	SPDEF	0.0132132	103
V$LHX3b_01	LHX3b	LHX3	0.148512	95
V$TR4_Q2	TR4	NR2C2	0.0943894	76
V$HOX13_02	HOXA5	HOXA5	0.0861647	72
V$YY1_03	YY1	YY1	0.138611	65
V$P53_02	p53	TP53	0.0902022	65
V$HOXB8_01	HOXB8	HOXB8	0.252883	60
V$LHX3_01	Lhx3	LHX3	0.148512	51
V$FOXJ2_02	FOXJ2	FOXJ2	0.302479	46

---

hsa-mir-203a

Matrix_id	transcription factor	Gene	PCC	Occurrence
V$PUR1_Q4	PUR1	PURA	0.0755609	17
V$MAFB_01	MAFB	MAFB	0.467928	16
V$GKLF_Q4	GKLF	KLF4	0.83526	16
V$ELF1_Q6	Elf-1	ELF1	0.480762	15
V$ZABC1_01	ZABC1	ZNF217	0.716748	15
V$GR_Q6	GR	NR3C1	0.0675384	15
V$SOX9_Q4	SOX9	SOX9	0.0004717	14
V$NFAT4_Q3	NF-AT4	NFATC3	0.021815	13
V$AML1_Q6	AML1	RUNX1	0.0678752	13
V$ETS2_Q6	c-Ets-2	ETS2	0.140909	13
V$SMAD4_Q6_01	Smad4	SMAD4	0.0976744	12
V$YY1_Q6	YY1	YY1	0.324002	12
V$Elf5_03	ELF5	ELF5	0.223713	12
V$YY1_Q6_02	YY1	YY1	0.324002	11
V$TCF4_01	TCF-4	TCF7L2	0.630403	11
V$SPI1_03	SPI1	SPI1	0.179009	11
V$AHR_Q5	AhR	AHR	0.771744	10
V$AP2GAMMA_01	AP-2gamma	TFAP2C	0.65752	10
V$LEF1_Q5	LEF-1	LEF1	0.0116866	10
V$AP2ALPHA_01	AP-2alpha	TFAP2A	0.640327	10

V$EHF_03	EHF	EHF	0.344943	9
V$ESE1_Q3	ESE-1	ELF3	0.50205	9
V$SP1_01	Sp1	SP1	0.0370407	9
V$TFIII_Q6	TFII-I	GTF2I	0.0415275	8
V$PDEF_02	PDEF	SPDEF	0.0520324	8
V$SP2_01	SP2	SP2	0.100923	8
V$ESE1_02	ESE-1	ELF3	0.50205	8
V$AP2ALPHA_Q6	AP-2alpha	TFAP2A	0.640327	8
V$SP1_Q6	Sp1	SP1	0.0370407	8
V$GR_01	GR	NR3C1	0.0675384	7
V$TCF4_Q5	TCF-4	TCF7L2	0.630403	7
V$FKLF_Q5	FKLF	KLF11	0.117459	7
V$SP1_Q6_01	Sp1	SP1	0.0370407	7
V$ERM_02	Erm	ETV5	0.370636	7
V$SP1_02	SP1	SP1	0.0370407	7
V$SP1_Q2_01	Sp1	SP1	0.0370407	7
V$SP1_Q4_01	Sp1	SP1	0.0370407	7
V$SREBP1_Q6	SREBP-1	SREBF1	0.0426744	7
V$KLF15_Q2	KLF15	KLF15	0.0168419	6
V$YY1_02	YY1	YY1	0.324002	6
V$ERG_03	ERG	ERG	0.0629793	6
V$EGR1_02	EGR-1	EGR1	0.0283619	6
V$RNF96_01	RNF96	TRIM28	0.177602	5
V$CP2_01	CP2	TFCP2	0.459927	4
V$EGR2_01	Egr-2	EGR2	0.254328	4
V$IRF7_Q3	IRF-7	IRF7	0.264988	4
V$AML2_Q3	AML2	RUNX3	0.0401921	4
V$STAT3_03	STAT3	STAT3	0.264897	4
V$YY1_03	YY1	YY1	0.324002	3
V$IRF1_Q6_01	IRF-1	IRF1	0.30332	3
V$IRF1_Q6	IRF-1	IRF1	0.30332	3
V$SP3_Q3	Sp3	SP3	0.325528	2
V$BLIMP1_Q6	Blimp-1	PRDM1	0.0493741	1

----

hsa-mir-204

Matrix_id	transcription factor	Gene	PCC	Occurrence
V$PUR1_Q4	PUR1	PURA	0.181171	84
V$PARP_Q4	PARP	PARP1	0.192357	83
V$ELF1_Q6	Elf-1	ELF1	0.0264517	77
V$SMAD4_Q6_01	Smad4	SMAD4	0.0303078	76
V$NR1B2_Q6	NR1B2	RARB	0.202756	72
V$CDX2_Q5_02	CDX-2	CDX2	0.0280741	72

V$TBP_Q6	TBP	TBP	0.213633	72
V$GR_Q6	GR	NR3C1	0.0818852	72
V$P300_01	p300	EP300	0.0699217	71
V$HNF4A_Q6_01	HNF-4alpha	HNF4A	0.546629	70
V$MEF2C_Q4	MEF-2C	MEF2C	0.186977	68
V$SMAD3_Q6_01	Smad3	SMAD3	0.0532211	68
V$ZIC3_01	Zic3	ZIC3	0.679025	66
V$YY1_01	YY1	YY1	0.0459148	65
V$CDX2_Q5_01	Cdx-2	CDX2	0.0280741	63
V$ETS1_B	c-Ets-1	ETS1	0.0940024	63
V$CETS1_Q6	C-ets-1	ETS1	0.0940024	61
V$IPF1_Q6	IPF1	PDX1	0.0338052	59
V$DLX5_01	dlx5	DLX5	0.203327	58
V$YY1_Q6_02	YY1	YY1	0.0459148	58
V$IPF1_Q4_01	IPF1	PDX1	0.0338052	58
V$Elf5_03	ELF5	ELF5	0.165555	58
V$GABPA_Q4	GABP-alpha	GABPA	0.0964245	58
V$AP4_Q6_02	AP-4	TFAP4	0.326813	58
V$YY1_Q6	YY1	YY1	0.0459148	57
V$ERBETA_Q5	ER-beta	ESR2	0.0998605	56
V$IPF1_01	IPF1	PDX1	0.0338052	56
V$NKX32_01	Nkx3-2	NKX3-2	0.0100384	53
V$ING4_01	ING4	ING4	0.262196	53
V$IRF4_Q6	IRF-4	IRF4	0.110294	52
V$PBX1_Q3	Pbx1	PBX1	0.276191	52
V$GATA3_01	GATA-3	GATA3	0.0268125	51
V$PARP_Q3	PARP	PARP1	0.192357	51
V$E47_02	E47	TCF3	0.0671175	48
V$E12_Q6	E12	TCF3	0.0671175	48
V$E2A_Q6	E2A	TCF3	0.0671175	48
V$FOXJ2_01	FOXJ2	FOXJ2	0.0444273	47
V$AP4_Q5	AP-4	TFAP4	0.326813	44
V$CEBPE_Q6	CEBPE	CEBPE	0.00392263	44
V$YY1_Q6_03	YY1	YY1	0.0459148	43
V$RFX1_02	RFX1	RFX1	0.0142555	40
V$PITX3_Q2	PITX3	PITX3	0.0487708	39
V$CRX_Q4	Crx	CRX	0.162233	39
V$GATA3_03	GATA-3	GATA3	0.0268125	38
V$HIF1A_Q6	HIF-1alpha	HIF1A	0.0914664	37
V$E2A_Q2	E2A	TCF3	0.0671175	37
V$ARNT_01	Arnt	ARNT	0.0198071	37
V$IRF8_Q6	IRF-8	IRF8	0.0398261	36
V$RFX1_01	RFX1	RFX1	0.0142555	35
V$SMAD3_Q6	SMAD3	SMAD3	0.0532211	35
V$PIT1_Q6	Pit-1	POU1F1	0.195302	35
V$NKX2B_Q3	NKX2B	NKX2-2	0.203899	34

V$ELF5_01	ELF5	ELF5	0.165555	33
V$SREBP1_Q6	SREBP-1	SREBF1	0.0446764	32
V$NURR1_Q3	NURR1	NR4A2	0.100714	30
V$EGR1_02	EGR-1	EGR1	0.274668	30
V$NKX22_02	NKX2B	NKX2-2	0.203899	29
V$ZIC1_01	Zic1	ZIC1	0.25411	28
V$CP2_01	CP2	TFCP2	0.30586	27
V$MEF2A_Q6	mef2A	MEF2A	0.0541596	27
V$PAX8_01	Pax-8	PAX8	0.213148	25
V$CRX_02	Crx	CRX	0.162233	22
V$SMAD4_Q6	SMAD4	SMAD4	0.0303078	17
V$GLI2_01	GLI2	GLI2	0.0301329	5

----

hsa-mir-205

Matrix_id	transcription factor	Gene	PCC	Occurrence
V$YY1_01	YY1	YY1	0.273947	5
V$IK_Q5	Ikaros	IKZF1	0.183815	5
V$GR_Q6	GR	NR3C1	0.18984	5
V$GKLF_Q4	GKLF	KLF4	0.1814	5
V$PUR1_Q4	PUR1	PURA	0.112523	5
V$NFAT4_Q3	NF-AT4	NFATC3	0.539784	4
V$SMAD4_Q6_01	Smad4	SMAD4	0.0261302	4
V$AHR_Q5	AhR	AHR	0.193886	4
V$AML1_Q6	AML1	RUNX1	0.0302832	4
V$ELF1_Q6	Elf-1	ELF1	0.735897	4
V$AML2_Q3	AML2	RUNX3	0.145346	4
V$SOX9_B1	SOX9	SOX9	0.147762	4
V$GATA2_02	GATA-2	GATA2	0.11398	4
V$CMYB_Q5	c-Myb	MYB	0.674471	3
V$Elf5_03	ELF5	ELF5	0.177529	3
V$AML1_Q4	AML1	RUNX1	0.0302832	3
V$HMGIY_01	HMGIY	HMGA1	0.428777	3
V$DLX5_01	dlx5	DLX5	0.0303426	3
V$SOX9_Q4	SOX9	SOX9	0.147762	3
V$AML2_Q3_01	AML2	RUNX3	0.145346	3
V$IRF7_Q3	IRF-7	IRF7	0.184351	3
V$MYB_Q6	c-Myb	MYB	0.674471	3
V$AML1_01	AML1a	RUNX1	0.0302832	3
V$GATA2_03	GATA-2	GATA2	0.11398	3
V$GATA3_02	GATA-3	GATA3	0.425813	3
V$YY1_Q6_03	YY1	YY1	0.273947	3
V$ESE1_02	ESE-1	ELF3	0.269092	2

V$EHF_03	EHF	EHF	0.310676	2
V$AML2_01	AML2	RUNX3	0.145346	2
V$GATA2_01	GATA-2	GATA2	0.11398	2
V$GATA3_01	GATA-3	GATA3	0.425813	2
V$FAC1_01	FAC1	BPTF	0.0889841	2
V$GR_01	GR	NR3C1	0.18984	2
V$ELF5_01	ELF5	ELF5	0.177529	2
V$SPIB_01	SPI-B	SPIB	0.0995714	2
V$ESE1_Q3	ESE-1	ELF3	0.269092	2
V$PET1_02	Pet-1	FEV	0.161418	2
V$IRF7_01	IRF-7	IRF7	0.184351	1
V$HOXB8_01	HOXB8	HOXB8	0.14782	1

hsa-mir-206

Matrix_id	transcription factor	Gene	PCC	Occurrence
V$PUR1_Q4	PUR1	PURA	0.427293	2324
V$IK_Q5	Ikaros	IKZF1	0.798112	2271
V$PARP_Q4	PARP	PARP1	0.655074	2245
V$PEA3_Q6	PEA3	ETV4	0.796234	2208
V$AP2REP_01	AP-2rep	KLF12	0.865701	2176
V$P300_01	p300	EP300	0.833364	2158
V$ZIC3_01	Zic3	ZIC3	0.363217	1992
V$MYB_Q6	c-Myb	MYB	0.288639	1979
V$CMYB_Q5	c-Myb	MYB	0.288639	1979
V$SMAD4_Q6_01	Smad4	SMAD4	0.73618	1979
V$TBP_Q6	TBP	TBP	0.252818	1945
V$CDX2_Q5_02	CDX-2	CDX2	0.888579	1945
V$NFAT4_Q3	NF-AT4	NFATC3	0.569094	1938
V$CETS1_Q6	C-ets-1	ETS1	0.399693	1901
V$ETS1_B	c-Ets-1	ETS1	0.399693	1885
V$GATA1_01	GATA-1	GATA1	0.441914	1868
V$NR1B2_Q6	NR1B2	RARB	0.710477	1866
V$TTF1_Q5	TTF-1	NKX2-1	0.342699	1864
V$ETS2_Q6	c-Ets-2	ETS2	0.696634	1829
V$GABPA_Q4	GABP-alpha	GABPA	0.475791	1805
V$MEF2C_Q4	MEF-2C	MEF2C	0.48074	1797
V$YY1_01	YY1	YY1	0.449055	1793
V$SMAD3_Q6_01	Smad3	SMAD3	0.884959	1766
V$DLX5_01	dlx5	DLX5	0.158001	1757
V$GR_Q6	GR	NR3C1	0.724236	1753
V$NANOG_02	Nanog	NANOG	0.940205	1745
V$Elf5_03	ELF5	ELF5	0.0498177	1743

V$IPF1_01	IPF1	PDX1	0.925961	1695
V$SOX5_01	SOX5	SOX5	0.506177	1680
V$SPI1_03	SPI1	SPI1	0.174527	1673
V$HNF4A_Q6_01	HNF-4alpha	HNF4A	0.188188	1656
V$AML1_Q6	AML1	RUNX1	0.6406	1652
V$ETS2_B	c-Ets-2	ETS2	0.696634	1625
V$YY1_Q6	YY1	YY1	0.449055	1618
V$IPF1_Q6	IPF1	PDX1	0.925961	1610
V$AP4_Q6_02	AP-4	TFAP4	0.653172	1599
V$GATA1_06	GATA-1	GATA1	0.441914	1591
V$GATA2_02	GATA-2	GATA2	0.403122	1591
V$GATA1_05	GATA-1	GATA1	0.441914	1591
V$SPI1_Q5	SPI1	SPI1	0.174527	1585
V$IPF1_Q4_01	IPF1	PDX1	0.925961	1552
V$YY1_Q6_02	YY1	YY1	0.449055	1535
V$SRY_02	SRY	SRY	0.701258	1532
V$NKX32_01	Nkx3-2	NKX3-2	0.847422	1468
V$CDX2_01	Cdx-2	CDX2	0.888579	1467
V$PITX3_Q2	PITX3	PITX3	0.653394	1447
V$PARP_Q3	PARP	PARP1	0.655074	1445
V$NFAT2_Q5	NF-AT2	NFATC1	0.882529	1419
V$IRF4_Q6	IRF-4	IRF4	0.87943	1407
V$HMGIY_01	HMGIY	HMGA1	0.0459198	1398
V$MAZ_Q6	MAZ	MAZ	0.185364	1398
V$ERBETA_Q5	ER-beta	ESR2	0.736373	1391
V$LRF_Q2	LRF	ZBTB7A	0.782433	1371
V$HNF3A_01	HNF3A	FOXA1	0.472129	1329
V$CRX_Q4	Crx	CRX	0.709578	1316
V$IPF1_Q4	IPF1	PDX1	0.925961	1299
V$MYOGENIN_Q6	myogenin	MYOG	0.794268	1219
V$LEF1_Q5	LEF-1	LEF1	0.0286833	1212
V$TCF3_01	TCF-3	TCF7L1	0.693715	1211
V$GFI1_Q6	Gfi1	GFI1	0.339111	1209
V$GFI1_Q6_01	Gfi1	GFI1	0.339111	1186
V$PITX2_Q2	Pitx2	PITX2	0.779874	1181
V$CEBPE_Q6	CEBPE	CEBPE	0.161939	1170
V$IRF8_Q6	IRF-8	IRF8	0.0278654	1130
V$SOX10_Q6	SOX10	SOX10	0.421037	1128
V$AP4_Q5	AP-4	TFAP4	0.653172	1126
V$FKLF_Q5	FKLF	KLF11	0.0978596	1121
V$TEF1_Q6_03	TEF-1	TEAD1	0.910778	1117
V$FOXM1_01	FOXM1	FOXM1	0.207297	1112
V$RFX1_02	RFX1	RFX1	0.942922	1061
V$CRX_Q4_01	CRX	CRX	0.709578	1003
V$TEF1_Q6	TEF-1	TEAD1	0.910778	984
V$RFX1_01	RFX1	RFX1	0.942922	979

V$AP4_Q6	AP-4	TFAP4	0.653172	971
V$IRF7_Q3	IRF-7	IRF7	0.030503	942
V$ELF5_01	ELF5	ELF5	0.0498177	937
V$MYOGENIN_Q6_01	myogenin	MYOG	0.794268	897
V$FAC1_01	FAC1	BPTF	0.375185	884
V$CEBPB_02	C/EBPbeta	CEBPB	0.231501	881
V$CEBPD_Q6	C/EBPdelta	CEBPD	0.198586	871
V$KLF15_Q2	KLF15	KLF15	0.0520265	795
V$HOXD9_Q2	Hoxd9	HOXD9	0.628729	777
V$DBP_Q6_01	DBP	DBP	0.860367	765
V$CEBPB_Q6	C/EBPbeta	CEBPB	0.231501	748
V$AP4_Q6_01	AP-4	TFAP4	0.653172	717
V$IPF1_02	IPF1	PDX1	0.925961	664
V$YY1_02	YY1	YY1	0.449055	661
V$HNF6_Q6	HNF6	ONECUT1	0.219596	650
V$HOXB8_01	HOXB8	HOXB8	0.522422	560
V$STAT4_Q5	STAT4	STAT4	0.799726	483
V$P53_02	p53	TP53	0.369416	479
V$EAR2_Q2	EAR2	NR2F6	0.301724	472
V$SOX2_Q6	SOX2	SOX2	0.252655	442
V$CREL_01	c-Rel	REL	0.636143	433
V$RORBETA_Q2	RORBETA	RORB	0.816269	417
V$POU6F1_03	POU6F1	POU6F1	0.517557	397
V$CEBPB_01	C/EBPbeta	CEBPB	0.231501	378
V$SPIB_01	SPI-B	SPIB	0.326225	322
V$POU6F1_02	POU6F1	POU6F1	0.517557	313
V$AMEF2_Q6	aMEF-2	MEF2A	0.173478	289

----

hsa-mir-20b

Matrix_id	transcription factor	Gene	PCC	Occurrence
V$CMYB_01	c-Myb	MYB	0.61151	3
V$IK_Q5	Ikaros	IKZF1	0.174961	3
V$YY1_Q6_02	YY1	YY1	0.149368	3
V$GR_01	GR	NR3C1	0.10348	3
V$YY1_Q6	YY1	YY1	0.149368	3
V$PUR1_Q4	PUR1	PURA	0.0599175	3
V$ELF1_Q6	Elf-1	ELF1	0.525298	3
V$CETS1_Q6	C-ets-1	ETS1	0.124359	3
V$GATA2_01	GATA-2	GATA2	0.0563743	3
V$ETS1_B	c-Ets-1	ETS1	0.124359	3
V$GR_Q6	GR	NR3C1	0.10348	3
V$NFAT4_Q3	NF-AT4	NFATC3	0.464244	3

V$GATA3_01	GATA-3	GATA3	0.420111	3
V$GFI1_Q6_01	Gfi1	GFI1	0.651682	2
V$IRF8_Q6	IRF-8	IRF8	0.213775	2
V$YY1_Q6_03	YY1	YY1	0.149368	2
V$IRF7_Q3	IRF-7	IRF7	0.291078	2
V$HMGIY_01	HMGIY	HMGA1	0.300849	2
V$STAT1_05	STAT1	STAT1	0.21721	2
V$GFI1_Q6	Gfi1	GFI1	0.651682	2
V$YY1_01	YY1	YY1	0.149368	2
V$GATA3_03	GATA-3	GATA3	0.420111	2
V$ZABC1_01	ZABC1	ZNF217	0.246383	1
V$CP2_01	CP2	TFCP2	0.0468669	1
V$AHR_01	AhR	AHR	0.0691786	1
V$AFP1_Q6	AFP1	ZFHX3	0.322208	1
V$STAT1_Q6	STAT1	STAT1	0.21721	1
V$AML2_Q3	AML2	RUNX3	0.0327857	1
V$ING4_01	ING4	ING4	0.218296	1
V$AHR_Q5	AhR	AHR	0.0691786	1
V$YY1_03	YY1	YY1	0.149368	1

hsa-mir-210

Matrix_id	transcription factor	Gene	PCC	Occurrence
V$PEA3_Q6	PEA3	ETV4	0.181904	31
V$PUR1_Q4	PUR1	PURA	0.22257	31
V$MAFB_01	MAFB	MAFB	0.325891	30
V$ELF1_Q6	Elf-1	ELF1	0.135001	29
V$PARP_Q4	PARP	PARP1	0.106653	28
V$P300_01	p300	EP300	0.328326	28
V$GATA1_01	GATA-1	GATA1	0.15547	28
V$TBP_Q6	TBP	TBP	0.263922	27
V$CETS1_Q6	C-ets-1	ETS1	0.0847119	27
V$ETS1_B	c-Ets-1	ETS1	0.0847119	26
V$SOX9_Q4	SOX9	SOX9	0.217622	26
V$SMAD4_Q6_01	Smad4	SMAD4	0.261466	26
V$ETS2_Q6	c-Ets-2	ETS2	0.191525	26
V$GABPA_Q4	GABP-alpha	GABPA	0.21627	26
V$YY1_Q6_02	YY1	YY1	0.323527	25
V$GR_Q6	GR	NR3C1	0.102412	24
V$YY1_Q6	YY1	YY1	0.323527	24
V$ETS2_B	c-Ets-2	ETS2	0.191525	24
V$YY1_01	YY1	YY1	0.323527	24
V$SPI1_Q5	SPI1	SPI1	0.138715	24

V$SPI1_03	SPI1	SPI1	0.138715	23
V$AML1_Q6	AML1	RUNX1	0.271904	21
V$SOX5_01	SOX5	SOX5	0.105141	21
V$GATA2_02	GATA-2	GATA2	0.656494	21
V$GATA1_06	GATA-1	GATA1	0.15547	21
V$GATA6_01	GATA-6	GATA6	0.0896153	21
V$DLX5_01	dlx5	DLX5	0.606654	21
V$AP4_Q6_02	AP-4	TFAP4	0.0973602	21
V$SOX9_B1	SOX9	SOX9	0.217622	21
V$GATA1_05	GATA-1	GATA1	0.15547	21
V$GATA3_02	GATA-3	GATA3	0.653216	19
V$FOXO3A_Q1	FOXO3A	FOXO3	0.168019	18
V$ZABC1_01	ZABC1	ZNF217	0.62802	17
V$HMGIY_01	HMGIY	HMGA1	0.253658	17
V$HNF3A_01	HNF3A	FOXA1	0.125	17
V$GATA3_01	GATA-3	GATA3	0.653216	17
V$GATA1_02	GATA-1	GATA1	0.15547	17
V$GATA2_01	GATA-2	GATA2	0.656494	16
V$NFAT2_Q5	NF-AT2	NFATC1	0.0251077	16
V$FOXJ2_01	FOXJ2	FOXJ2	0.260686	16
V$PITX2_01	PITX2	PITX2	0.382693	16
V$PARP_Q3	PARP	PARP1	0.106653	16
V$TCF4_Q5	TCF-4	TCF7L2	0.553351	16
V$TCF4_01	TCF-4	TCF7L2	0.553351	15
V$ELF5_01	ELF5	ELF5	0.263805	15
V$GATA1_04	GATA-1	GATA1	0.15547	15
V$SRY_02	SRY	SRY	0.0825501	15
V$OC2_Q3	OC-2	ONECUT2	0.0105236	15
V$LHX3b_01	LHX3b	LHX3	0.0998656	12
V$AML1_01	AML1a	RUNX1	0.271904	11
V$IRF7_Q3	IRF-7	IRF7	0.153576	11
V$GATA2_03	GATA-2	GATA2	0.656494	11
V$HOX13_02	HOXA5	HOXA5	0.0802415	10
V$CART1_02	CART1	ALX1	0.0311684	10
V$NURR1_Q3	NURR1	NR4A2	0.0111249	10
V$FOXO4_02	FOXO4	FOXO4	0.832802	9
V$LHX3_01	Lhx3	LHX3	0.0998656	7
V$P53_02	p53	TP53	0.379877	7
V$CIZ_01	CIZ	ZNF384	0.205713	7
V$IRF1_Q6_01	IRF-1	IRF1	0.464125	5
V$IRF1_Q6	IRF-1	IRF1	0.464125	5
V$HNF1B_01	HNF-1beta	HNF1B	0.0319033	4
V$IRF7_01	IRF-7	IRF7	0.153576	3
V$NKX3A_02	Nkx3A	NKX3-1	0.218517	3
V$LHX3A_01	Lhx3a	LHX3	0.0998656	2
V$BLIMP1_Q6	Blimp-1	PRDM1	0.214247	1

V$AFP1_Q6	AFP1	ZFHX3	0.229232	1

----

hsa-mir-211

Matrix_id	transcription factor	Gene	PCC	Occurrence
V$PUR1_Q4	PUR1	PURA	0.0403035	123
V$AP2REP_01	AP-2rep	KLF12	0.00540787	120
V$PARP_Q4	PARP	PARP1	0.0310238	119
V$IK_Q5	Ikaros	IKZF1	0.0572328	118
V$MAFB_01	MAFB	MAFB	0.00464994	115
V$ELF1_Q6	Elf-1	ELF1	0.124011	112
V$SMAD4_Q6_01	Smad4	SMAD4	0.0506633	112
V$NR1B2_Q6	NR1B2	RARB	0.264421	110
V$GR_Q6	GR	NR3C1	0.0918702	107
V$P300_01	p300	EP300	0.0959255	107
V$CDX2_Q5_02	CDX-2	CDX2	0.111862	103
V$TBP_Q6	TBP	TBP	0.0120179	103
V$SMAD3_Q6_01	Smad3	SMAD3	0.126372	101
V$ZIC3_01	Zic3	ZIC3	0.563813	100
V$NANOG_02	Nanog	NANOG	0.0533247	97
V$CETS1_Q6	C-ets-1	ETS1	0.116323	92
V$ETS1_B	c-Ets-1	ETS1	0.116323	90
V$DLX5_01	dlx5	DLX5	0.136087	89
V$CDX2_Q5_01	Cdx-2	CDX2	0.111862	89
V$IPF1_01	IPF1	PDX1	0.0935221	88
V$GABPA_Q4	GABP-alpha	GABPA	0.0779106	88
V$Elf5_03	ELF5	ELF5	0.269634	87
V$AP4_Q6_02	AP-4	TFAP4	0.203206	87
V$IPF1_Q6	IPF1	PDX1	0.0935221	87
V$SOX5_01	SOX5	SOX5	0.213081	87
V$ERBETA_Q5	ER-beta	ESR2	0.142962	86
V$IPF1_Q4_01	IPF1	PDX1	0.0935221	86
V$GATA2_02	GATA-2	GATA2	0.0036762	85
V$IRF4_Q6	IRF-4	IRF4	0.193121	82
V$GATA3_02	GATA-3	GATA3	0.104996	79
V$CDX2_01	Cdx-2	CDX2	0.111862	78
V$PBX1_04	Pbx1	PBX1	0.154783	77
V$IPF1_Q4	IPF1	PDX1	0.0935221	74
V$PBX1_Q3	Pbx1	PBX1	0.154783	74
V$CMAF_01	c-Maf	MAF	0.732006	74
V$LRF_Q2	LRF	ZBTB7A	0.119704	74
V$PARP_Q3	PARP	PARP1	0.0310238	72
V$CEBPE_Q6	CEBPE	CEBPE	0.143578	72

V$GATA3_01	GATA-3	GATA3	0.104996	71
V$HNF1_02	HNF-1alpha	HNF1A	0.22051	69
V$GFI1_Q6	Gfi1	GFI1	0.0794245	69
V$MYOGENIN_Q6	myogenin	MYOG	0.227856	67
V$AP4_Q5	AP-4	TFAP4	0.203206	66
V$ERR1_Q3	ERR1	ESRRA	0.160934	65
V$TEF1_Q6_03	TEF-1	TEAD1	0.0627031	65
V$AP4_Q6	AP-4	TFAP4	0.203206	64
V$SOX10_Q6	SOX10	SOX10	0.0342382	64
V$PITX3_Q2	PITX3	PITX3	0.062308	62
V$GFI1_Q6_01	Gfi1	GFI1	0.0794245	62
V$OC2_Q3	OC-2	ONECUT2	0.157844	58
V$GR_01	GR	NR3C1	0.0918702	55
V$IRF8_Q6	IRF-8	IRF8	0.0380851	55
V$MYOGENIN_Q6_01	myogenin	MYOG	0.227856	55
V$AP2ALPHA_Q6	AP-2alpha	TFAP2A	0.00711075	51
V$SMAD3_Q6	SMAD3	SMAD3	0.126372	51
V$FOXO4_02	FOXO4	FOXO4	0.0491282	50
V$HOXD9_Q2	Hoxd9	HOXD9	0.220421	49
V$PIT1_Q6	Pit-1	POU1F1	0.299175	48
V$AP4_Q6_01	AP-4	TFAP4	0.203206	45
V$HOXA9_01	hoxa9	HOXA9	0.444678	44
V$CRX_02	Crx	CRX	0.302851	42
V$CRX_Q4_01	CRX	CRX	0.302851	42
V$HNF6_Q6	HNF6	ONECUT1	0.104798	41
V$MATH1_Q2	MATH1	ATOH1	0.295429	38
V$MYOD_Q6_02	MyoD	MYOD1	0.0248383	35
V$STAT4_Q5	STAT4	STAT4	0.10052	34
V$IPF1_02	IPF1	PDX1	0.0935221	31
V$CART1_02	CART1	ALX1	0.195312	31
V$TR4_Q2	TR4	NR2C2	0.0619776	30
V$HOX13_02	HOXA5	HOXA5	0.0520911	30
V$IPF1_06	ipf1	PDX1	0.0935221	29
V$ELK1_01	Elk-1	ELK1	0.165919	28
V$MITF_Q6	MITF	MITF	0.270395	26
V$IPF1_03	IPF1	PDX1	0.0935221	26
V$HNF1B_01	HNF-1beta	HNF1B	0.802887	22
V$CDP_04	CDP	CUX1	0.049784	19
V$HOXA7_01	HOXA7	HOXA7	0.316996	8
V$RORA_Q4	RORalpha	RORA	0.305524	8

----

hsa-mir-212

Matrix_id	transcription factor	Gene	PCC	Occurrence

V$ZIC3_01	Zic3	ZIC3	0.143601	4
V$ING4_01	ING4	ING4	0.301261	4
V$PUR1_Q4	PUR1	PURA	0.0922767	4
V$SOX9_B1	SOX9	SOX9	0.163344	4
V$MAZ_Q6	MAZ	MAZ	0.214972	4
V$PARP_Q4	PARP	PARP1	0.136099	4
V$Elf5_03	ELF5	ELF5	0.0225727	4
V$AML1_Q6	AML1	RUNX1	0.024655	4
V$SOX9_Q4	SOX9	SOX9	0.163344	4
V$ELK1_02	Elk-1	ELK1	0.293691	3
V$AP4_Q6_02	AP-4	TFAP4	0.120424	3
V$HNF4A_Q6_01	HNF-4alpha	HNF4A	0.288847	3
V$ELK1_06	ELK-1	ELK1	0.293691	3
V$ER71_02	ER71	ETV2	0.112598	3
V$ELF5_01	ELF5	ELF5	0.0225727	3
V$NR1B2_Q6	NR1B2	RARB	0.0556042	3
V$TBP_Q6	TBP	TBP	0.0858653	3
V$VDR_Q3	VDR	VDR	0.0196072	2
V$AML1_01	AML1a	RUNX1	0.024655	2
V$MAZ_Q6_01	MAZ	MAZ	0.214972	2
V$CP2_01	CP2	TFCP2	0.134017	2
V$ERM_02	Erm	ETV5	0.275232	2
V$PET1_02	Pet-1	FEV	0.00412795	2
V$PDEF_02	PDEF	SPDEF	0.045207	2
V$CREM_Q6	CREM	CREM	0.245319	2
V$SP2_01	SP2	SP2	0.134856	2
V$GATA6_01	GATA-6	GATA6	0.617341	2
V$ELK1_03	Elk-1	ELK1	0.293691	1
V$ELK1_04	Elk-1	ELK1	0.293691	1
V$AP4_Q6_01	AP-4	TFAP4	0.120424	1
V$AML1_Q4	AML1	RUNX1	0.024655	1
V$HSF1_01	HSF1	HSF1	0.139059	1
V$AP4_Q5	AP-4	TFAP4	0.120424	1
V$AP4_Q6	AP-4	TFAP4	0.120424	1
V$MATH1_Q2	MATH1	ATOH1	0.0393266	1
V$ZIC1_01	Zic1	ZIC1	0.453233	1
V$GLI_Q2	GLI	GLI1	0.167099	1

---

hsa-mir-215

Matrix_id	transcription factor	Gene	PCC	Occurrence
V$PUR1_Q4	PUR1	PURA	0.43025	37

V$ELF1_Q6	Elf-1	ELF1	0.332399	33
V$SMAD4_Q6_01	Smad4	SMAD4	0.0271747	33
V$YY1_01	YY1	YY1	0.165673	31
V$CETS1_Q6	C-ets-1	ETS1	0.176191	31
V$ETS2_Q6	c-Ets-2	ETS2	0.0177435	29
V$MYB_Q6	c-Myb	MYB	0.0575176	28
V$SOX5_01	SOX5	SOX5	0.00241572	28
V$SOX9_Q4	SOX9	SOX9	0.234278	28
V$CMYB_Q5	c-Myb	MYB	0.0575176	28
V$SOX9_B1	SOX9	SOX9	0.234278	28
V$GATA1_02	GATA-1	GATA1	0.152478	26
V$SPI1_Q5	SPI1	SPI1	0.0653078	25
V$GATA2_02	GATA-2	GATA2	0.309857	25
V$GATA1_06	GATA-1	GATA1	0.152478	25
V$GATA1_05	GATA-1	GATA1	0.152478	25
V$GATA1_04	GATA-1	GATA1	0.152478	24
V$FOXO3A_Q1	FOXO3A	FOXO3	0.175063	22
V$CMYB_01	c-Myb	MYB	0.0575176	22
V$GATA3_02	GATA-3	GATA3	0.0856937	22
V$LRF_Q2	LRF	ZBTB7A	0.0275826	21
V$CMAF_01	c-Maf	MAF	0.233548	20
V$HNF3B_Q6	HNF-3beta	FOXA2	0.107165	20
V$GFI1_Q6_01	Gfi1	GFI1	0.106535	19
V$GFI1_Q6	Gfi1	GFI1	0.106535	18
V$TCF4_Q5	TCF-4	TCF7L2	0.0517736	17
V$HNF3A_01	HNF3A	FOXA1	0.735575	16
V$ELF5_01	ELF5	ELF5	0.0139213	16
V$GATA2_03	GATA-2	GATA2	0.309857	15
V$HOXA9_01	hoxa9	HOXA9	0.393327	15
V$TCF4_01	TCF-4	TCF7L2	0.0517736	13
V$HOXD9_Q2	Hoxd9	HOXD9	0.0607979	12
V$CDX1_01	Cdx-1	CDX1	0.0282404	12
V$EAR2_Q2	EAR2	NR2F6	0.181468	7
V$HTF4_Q2	HTF4	TCF12	0.05525	7
V$ATF5_01	ATF5	ATF5	0.0871825	2
V$E4F1_Q6_01	E4F1	E4F1	0.204939	2
V$E4F1_Q6	E4F1	E4F1	0.204939	1

---

hsa-mir-218-1

Matrix_id	transcription factor	Gene	PCC	Occurrence
V$MAFB_01	MAFB	MAFB	0.304714	17
V$PUR1_Q4	PUR1	PURA	0.393752	17

V$PARP_Q4	PARP	PARP1	0.228955	16
V$GKLF_Q4	GKLF	KLF4	0.212277	16
V$ZIC3_01	Zic3	ZIC3	0.0103631	16
V$AHR_Q5	AhR	AHR	0.208261	15
V$PBX1_04	Pbx1	PBX1	0.451471	14
V$AP4_Q6_02	AP-4	TFAP4	0.1857	14
V$ELK1_02	Elk-1	ELK1	0.108796	14
V$ELK1_06	ELK-1	ELK1	0.108796	13
V$FOXO3A_Q1	FOXO3A	FOXO3	0.0559445	13
V$SOX9_Q4	SOX9	SOX9	0.0759247	12
V$YY1_Q6_02	YY1	YY1	0.294638	12
V$HNF4A_Q6_01	HNF-4alpha	HNF4A	0.00970896	12
V$ETS2_Q6	c-Ets-2	ETS2	0.122033	12
V$YY1_Q6	YY1	YY1	0.294638	11
V$GATA3_02	GATA-3	GATA3	0.1817	11
V$GATA6_01	GATA-6	GATA6	0.318599	11
V$MAZ_Q6	MAZ	MAZ	0.0182289	11
V$GATA2_02	GATA-2	GATA2	0.108162	11
V$AP2ALPHA_Q6	AP-2alpha	TFAP2A	0.126621	10
V$AP2ALPHA_01	AP-2alpha	TFAP2A	0.126621	9
V$TCF4_01	TCF-4	TCF7L2	0.190838	9
V$AP4_Q5	AP-4	TFAP4	0.1857	9
V$GABPBETA_Q3	GABP-beta	GABPB1	0.0282979	8
V$GATA3_03	GATA-3	GATA3	0.1817	8
V$NURR1_Q3	NURR1	NR4A2	0.394577	8
V$CP2_01	CP2	TFCP2	0.396996	7
V$CEBPB_02	C/EBPbeta	CEBPB	0.163141	7
V$KLF15_Q2	KLF15	KLF15	0.0260843	7
V$ERM_02	Erm	ETV5	0.471077	6
V$AP4_Q6	AP-4	TFAP4	0.1857	6
V$FAC1_01	FAC1	BPTF	0.0478956	6
V$TCF4_Q5	TCF-4	TCF7L2	0.190838	6
V$E2F1_Q3_01	E2F-1	E2F1	0.0526963	5
V$YY1_02	YY1	YY1	0.294638	5
V$E2F1_Q6_01	E2F-1	E2F1	0.0526963	5
V$AP4_01	AP-4	TFAP4	0.1857	5
V$AP4_Q6_01	AP-4	TFAP4	0.1857	3
V$E2F1_Q4	E2F-1	E2F1	0.0526963	3
V$AP2GAMMA_01	AP-2gamma	TFAP2C	0.0930458	3
V$EAR2_Q2	EAR2	NR2F6	0.117923	2
V$ELK1_04	Elk-1	ELK1	0.108796	1

----

hsa-mir-218-2

Matrix_id	transcription factor	Gene	PCC	Occurrence
V$MAFB_01	MAFB	MAFB	0.304714	17
V$PUR1_Q4	PUR1	PURA	0.393752	17
V$PARP_Q4	PARP	PARP1	0.228955	16
V$PBX1_04	Pbx1	PBX1	0.451471	14
V$DLX5_01	dlx5	DLX5	0.123909	14
V$GATA3_01	GATA-3	GATA3	0.1817	13
V$FOXO3A_Q1	FOXO3A	FOXO3	0.0559445	13
V$SOX9_B1	SOX9	SOX9	0.0759247	13
V$SOX9_Q4	SOX9	SOX9	0.0759247	12
V$HNF4A_Q6_01	HNF-4alpha	HNF4A	0.00970896	12
V$ETS2_Q6	c-Ets-2	ETS2	0.122033	12
V$GR_Q6	GR	NR3C1	0.0615529	12
V$ETS2_B	c-Ets-2	ETS2	0.122033	12
V$GATA3_02	GATA-3	GATA3	0.1817	11
V$GATA6_01	GATA-6	GATA6	0.318599	11
V$GATA2_02	GATA-2	GATA2	0.108162	11
V$PITX3_Q2	PITX3	PITX3	0.0762143	10
V$GATA2_01	GATA-2	GATA2	0.108162	10
V$MEF2C_Q4	MEF-2C	MEF2C	0.43002	10
V$TCF4_01	TCF-4	TCF7L2	0.190838	9
V$MEF2A_Q6	mef2A	MEF2A	0.444574	7
V$HBP1_Q2	hbp1	HBP1	0.0495732	7
V$PARP_Q3	PARP	PARP1	0.228955	7
V$DBP_Q6_01	DBP	DBP	0.0257149	7
V$MSX1_01	Msx-1	MSX1	0.142232	7
V$TCF4_Q5	TCF-4	TCF7L2	0.190838	6
V$FAC1_01	FAC1	BPTF	0.0478956	6
V$SOX10_Q6	SOX10	SOX10	0.344962	6
V$IRF8_Q6	IRF-8	IRF8	0.129931	5
V$HOXB8_01	HOXB8	HOXB8	0.101634	5
V$GATA2_03	GATA-2	GATA2	0.108162	5
V$CEBPD_Q6	C/EBPdelta	CEBPD	0.179053	5
V$CEBPB_Q6	C/EBPbeta	CEBPB	0.163141	5
V$SOX2_Q6	SOX2	SOX2	0.164365	4
V$PIT1_Q6	Pit-1	POU1F1	0.0654956	4
V$CDP_04	CDP	CUX1	0.38867	4
V$CEBPB_01	C/EBPbeta	CEBPB	0.163141	3
V$RSRFC4_Q2	RSRFC4	MEF2A	0.444574	3
V$CIZ_01	CIZ	ZNF384	0.0728616	3
V$RSRFC4_01	RSRFC4	MEF2A	0.444574	2

hsa-mir-219-1

Matrix_id	transcription factor	Gene	PCC	Occurrence
V$PUR1_Q4	PUR1	PURA	0.419204	491
V$MAFB_01	MAFB	MAFB	0.17072	455
V$PARP_Q4	PARP	PARP1	0.42116	448
V$ZIC3_01	Zic3	ZIC3	0.198149	445
V$ETS2_Q6	c-Ets-2	ETS2	0.195107	409
V$SOX9_Q4	SOX9	SOX9	0.241243	395
V$GABPA_Q4	GABP-alpha	GABPA	0.140881	395
V$TBP_Q6	TBP	TBP	0.024102	386
V$NR1B2_Q6	NR1B2	RARB	0.00106793	385
V$MAZ_Q6	MAZ	MAZ	0.224981	361
V$GR_Q6	GR	NR3C1	0.0593375	354
V$YY1_Q6_02	YY1	YY1	0.0987488	332
V$ETS2_B	c-Ets-2	ETS2	0.195107	332
V$YY1_Q6	YY1	YY1	0.0987488	332
V$SOX9_B1	SOX9	SOX9	0.241243	322
V$ZBP89_Q4	ZBP89	ZNF148	0.11751	294
V$TFIII_Q6	TFII-I	GTF2I	0.268589	289
V$PARP_Q3	PARP	PARP1	0.42116	288
V$HNF3B_Q6	HNF-3beta	FOXA2	0.0642184	274
V$ELK1_02	Elk-1	ELK1	0.110979	273
V$FOXO3A_Q1	FOXO3A	FOXO3	0.0478665	264
V$PBX1_04	Pbx1	PBX1	0.230773	260
V$PITX3_Q2	PITX3	PITX3	0.0416587	260
V$MAZ_Q6_01	MAZ	MAZ	0.224981	256
V$PBX1_Q3	Pbx1	PBX1	0.230773	254
V$ELK1_06	ELK-1	ELK1	0.110979	238
V$KLF15_Q2	KLF15	KLF15	0.0413249	222
V$ERF_02	ERF	ERF	0.239771	213
V$SOX10_Q6	SOX10	SOX10	0.727578	209
V$GR_01	GR	NR3C1	0.0593375	205
V$SP2_01	SP2	SP2	0.0908908	198
V$E2F1_Q3	E2F-1	E2F1	0.138356	182
V$CP2_01	CP2	TFCP2	0.247501	174
V$ZIC1_01	Zic1	ZIC1	0.95447	172
V$SP4_Q5	SP4	SP4	0.0420767	164
V$FAC1_01	FAC1	BPTF	0.14205	152
V$E2F1_Q6	E2F-1	E2F1	0.138356	146
V$YY1_02	YY1	YY1	0.0987488	143
V$ELK1_01	Elk-1	ELK1	0.110979	127
V$ERM_02	Erm	ETV5	0.168486	121
V$CACCCBINDINGFACT	CACCC-binding factor	ZNF148	0.11751	119
V$YY1_03	YY1	YY1	0.0987488	86
V$E2F1_Q4	E2F-1	E2F1	0.138356	80

V$GRE_C	GR	NR3C1	0.0593375	30
V$ELK1_04	Elk-1	ELK1	0.110979	18

hsa-mir-219-2

Matrix_id	transcription factor	Gene	PCC	Occurrence
V$PUR1_Q4	PUR1	PURA	0.419204	491
V$MAFB_01	MAFB	MAFB	0.17072	455
V$PARP_Q4	PARP	PARP1	0.42116	448
V$ZIC3_01	Zic3	ZIC3	0.198149	445
V$TBP_Q6	TBP	TBP	0.024102	386
V$MAZ_Q6	MAZ	MAZ	0.224981	361
V$YY1_01	YY1	YY1	0.0987488	359
V$MEF2C_Q4	MEF-2C	MEF2C	0.812276	351
V$DLX5_01	dlx5	DLX5	0.00737451	327
V$SOX9_B1	SOX9	SOX9	0.241243	322
V$ZBP89_Q4	ZBP89	ZNF148	0.11751	294
V$SRY_02	SRY	SRY	0.180808	293
V$TFIII_Q6	TFII-I	GTF2I	0.268589	289
V$HNF3B_Q6	HNF-3beta	FOXA2	0.0642184	274
V$FOXO3A_Q1	FOXO3A	FOXO3	0.0478665	264
V$PITX3_Q2	PITX3	PITX3	0.0416587	260
V$PBX1_04	Pbx1	PBX1	0.230773	260
V$MAZ_Q6_01	MAZ	MAZ	0.224981	256
V$PBX1_Q3	Pbx1	PBX1	0.230773	254
V$KLF15_Q2	KLF15	KLF15	0.0413249	222
V$NKX22_02	NKX2B	NKX2-2	0.881091	151
V$MEF2A_Q6	mef2A	MEF2A	0.67377	149
V$DBP_Q6_01	DBP	DBP	0.27011	147
V$MSX1_01	Msx-1	MSX1	0.188441	118
V$USF2_Q6	USF2	USF2	0.336519	87
V$MAX_01	Max	MAX	0.546893	84
V$AMEF2_Q6	aMEF-2	MEF2A	0.67377	54
V$POU3F2_02	POU3F2	POU3F2	0.0699529	45
V$IRF2_01	IRF-2	IRF2	0.00408927	44
V$MAX_Q6	MAX	MAX	0.546893	38
V$CDP_02	CDP	CUX1	0.0613238	15

hsa-mir-22

Matrix_id	transcription factor	Gene	PCC	Occurrence

V$IK_Q5	Ikaros	IKZF1	0.510882	103
V$PUR1_Q4	PUR1	PURA	0.356422	102
V$GKLF_Q4	GKLF	KLF4	0.160058	101
V$PEA3_Q6	PEA3	ETV4	0.669774	100
V$AP2REP_01	AP-2rep	KLF12	0.725178	97
V$PARP_Q4	PARP	PARP1	0.537067	97
V$P300_01	p300	EP300	0.769728	95
V$MAFB_01	MAFB	MAFB	0.0292692	94
V$ZIC3_01	Zic3	ZIC3	0.337725	93
V$SMAD4_Q6_01	Smad4	SMAD4	0.692398	91
V$MYB_Q6	c-Myb	MYB	0.0563637	90
V$CMYB_Q5	c-Myb	MYB	0.0563637	90
V$ETS2_Q6	c-Ets-2	ETS2	0.429337	87
V$CETS1_Q6	C-ets-1	ETS1	0.539656	85
V$TBX5_02	TBX5	TBX5	0.778279	85
V$Elf5_03	ELF5	ELF5	0.0522657	83
V$GATA1_01	GATA-1	GATA1	0.585762	83
V$GABPA_Q4	GABP-alpha	GABPA	0.514659	83
V$ETS1_B	c-Ets-1	ETS1	0.539656	83
V$NR1B2_Q6	NR1B2	RARB	0.700365	82
V$TTF1_Q5	TTF-1	NKX2-1	0.151892	79
V$AML1_Q6	AML1	RUNX1	0.667671	78
V$NANOG_02	Nanog	NANOG	0.643979	77
V$DLX5_01	dlx5	DLX5	0.425646	77
V$GR_Q6	GR	NR3C1	0.547451	77
V$SPI1_Q5	SPI1	SPI1	0.160585	77
V$ETS2_B	c-Ets-2	ETS2	0.429337	76
V$YY1_Q6	YY1	YY1	0.483805	76
V$IPF1_01	IPF1	PDX1	0.722085	76
V$AP4_Q6_02	AP-4	TFAP4	0.417889	75
V$SMAD3_Q6_01	Smad3	SMAD3	0.550224	75
V$YY1_01	YY1	YY1	0.483805	74
V$MEF2C_Q4	MEF-2C	MEF2C	0.212044	73
V$TBX5_01	TBX5	TBX5	0.778279	73
V$YY1_Q6_02	YY1	YY1	0.483805	69
V$ZBP89_Q4	ZBP89	ZNF148	0.503578	69
V$BEN_01	BEN	GTF2IRD1	0.744349	69
V$SOX5_01	SOX5	SOX5	0.410951	66
V$SPI1_03	SPI1	SPI1	0.160585	66
V$E47_02	E47	TCF3	0.57359	65
V$E12_Q6	E12	TCF3	0.57359	65
V$E2A_Q6	E2A	TCF3	0.57359	65
V$MYOD_Q6_01	MyoD	MYOD1	0.580785	65
V$TEL1_02	TEL1	ETV6	0.685481	64
V$AHR_Q5	AhR	AHR	0.316666	64

V$GATA1_02	GATA-1	GATA1	0.585762	64
V$ING4_01	ING4	ING4	0.0651726	63
V$ELK1_02	Elk-1	ELK1	0.755558	63
V$ETV3_02	ETV3	ETV3	0.521562	62
V$ER71_02	ER71	ETV2	0.563336	60
V$SRY_02	SRY	SRY	0.368624	58
V$FOXO3A_Q1	FOXO3A	FOXO3	0.382459	56
V$ELK1_06	ELK-1	ELK1	0.755558	56
V$HNF3B_Q6	HNF-3beta	FOXA2	0.266096	55
V$ESE1_Q3	ESE-1	ELF3	0.042279	55
V$ETV7_01	ETV7	ETV7	0.725496	54
V$PARP_Q3	PARP	PARP1	0.537067	53
V$GATA1_04	GATA-1	GATA1	0.585762	53
V$ERF_02	ERF	ERF	0.353781	52
V$HNF1_02	HNF-1alpha	HNF1A	0.584638	51
V$EHF_03	EHF	EHF	0.07343	51
V$HIF1A_Q6	HIF-1alpha	HIF1A	0.132031	51
V$TFIII_Q6	TFII-I	GTF2I	0.56936	51
V$GATA3_01	GATA-3	GATA3	0.346647	51
V$ARNT_01	Arnt	ARNT	0.798673	51
V$ZFX_01	Zfx	ZFX	0.639109	50
V$CMAF_01	c-Maf	MAF	0.352015	49
V$AML2_Q3	AML2	RUNX3	0.296287	48
V$GFI1_Q6_01	Gfi1	GFI1	0.287909	47
V$GFI1_Q6	Gfi1	GFI1	0.287909	47
V$CP2_01	CP2	TFCP2	0.426252	46
V$FOXP3_01	FOXP3	FOXP3	0.700161	46
V$CREM_Q6	CREM	CREM	0.589525	45
V$FOXJ2_01	FOXJ2	FOXJ2	0.731814	45
V$ERR1_Q3	ERR1	ESRRA	0.762818	43
V$NEUROD_02	NeuroD	NEUROD1	0.620617	42
V$CEBPE_Q6	CEBPE	CEBPE	0.569255	42
V$ESE1_02	ESE-1	ELF3	0.042279	41
V$VDR_Q3	VDR	VDR	0.761277	41
V$RFX1_01	RFX1	RFX1	0.674361	40
V$NKX2B_Q3	NKX2B	NKX2-2	0.077318	39
V$AML1_01	AML1a	RUNX1	0.667671	39
V$SMAD3_Q6	SMAD3	SMAD3	0.550224	39
V$NKX22_02	NKX2B	NKX2-2	0.077318	37
V$IRF7_Q3	IRF-7	IRF7	0.151622	37
V$RFX1_02	RFX1	RFX1	0.674361	35
V$AML2_Q3_01	AML2	RUNX3	0.296287	35
V$ERG_03	ERG	ERG	0.762631	34
V$PAX8_01	Pax-8	PAX8	0.245238	34
V$AML1_Q4	AML1	RUNX1	0.667671	34
V$PET1_02	Pet-1	FEV	0.557489	33

V$CEBPB_02	C/EBPbeta	CEBPB	0.476175	33
V$CACCCBINDINGFACT	CACCC-binding factor	ZNF148	0.503578	32
V$CEBPD_Q6	C/EBPdelta	CEBPD	0.328161	32
V$NURR1_Q3	NURR1	NR4A2	0.138209	31
V$ATF3_Q6_01	ATF-3	ATF3	0.371438	30
V$AML2_01	AML2	RUNX3	0.296287	29
V$PDEF_02	PDEF	SPDEF	0.114186	29
V$CEBPB_Q6	C/EBPbeta	CEBPB	0.476175	27
V$MSX1_01	Msx-1	MSX1	0.363295	27
V$EKLF_Q5	EKLF	KLF1	0.708355	26
V$P53_02	p53	TP53	0.558624	26
V$LHX3b_01	LHX3b	LHX3	0.754471	23
V$ERG_01	ERG	ERG	0.762631	23
V$MITF_Q6	MITF	MITF	0.356943	20
V$ERM_02	Erm	ETV5	0.493488	20
V$HOXB8_01	HOXB8	HOXB8	0.352211	19
V$NCX_02	Ncx	TLX2	0.717563	19
V$USF2_Q6	USF2	USF2	0.626366	19
V$ARNT_02	Arnt	ARNT	0.798673	19
V$NMYC_01	N-Myc	MYCN	0.743842	19
V$CART1_02	CART1	ALX1	0.730223	18
V$CMYC_02	c-Myc	MYC	0.0730954	18
V$MAX_01	Max	MAX	0.106026	17
V$HOX13_02	HOXA5	HOXA5	0.333915	17
V$IPF1_03	IPF1	PDX1	0.722085	16
V$IPF1_06	ipf1	PDX1	0.722085	15
V$FLI1_02	Fli-1	FLI1	0.594115	13
V$SAP1A_01	SAP-1a	ELK4	0.755665	13
V$CEBPB_01	C/EBPbeta	CEBPB	0.476175	13
V$LHX3_01	Lhx3	LHX3	0.754471	11
V$ATF5_01	ATF5	ATF5	0.215914	10
V$LHX3A_01	Lhx3a	LHX3	0.754471	4

----

hsa-mir-221

Matrix_id	transcription factor	Gene	PCC	Occurrence
V$ARNT_01	Arnt	ARNT	0.268851	4
V$YY1_Q6	YY1	YY1	0.205565	4
V$TBP_Q6	TBP	TBP	0.185699	4
V$MEF2C_Q4	MEF-2C	MEF2C	0.0489535	4
V$PARP_Q4	PARP	PARP1	0.0915547	4
V$YY1_Q6_02	YY1	YY1	0.205565	4
V$ERR1_Q3	ERR1	ESRRA	0.161773	4

V$GKLF_Q4	GKLF	KLF4	0.404562	4
V$SMAD4_Q6_01	Smad4	SMAD4	0.233539	4
V$DLX5_01	dlx5	DLX5	0.358634	4
V$P300_01	p300	EP300	0.145849	4
V$NR1B2_Q6	NR1B2	RARB	0.0808548	4
V$YY1_02	YY1	YY1	0.205565	4
V$PUR1_Q4	PUR1	PURA	0.302581	4
V$GATA1_01	GATA-1	GATA1	0.451234	4
V$AP4_Q6_02	AP-4	TFAP4	0.00696687	3
V$YY1_03	YY1	YY1	0.205565	3
V$CMAF_01	c-Maf	MAF	0.0400562	3
V$E2A_Q6	E2A	TCF3	0.00426296	3
V$E2A_Q2	E2A	TCF3	0.00426296	3
V$MAFB_01	MAFB	MAFB	0.192797	3
V$ESE1_Q3	ESE-1	ELF3	0.0669412	3
V$YY1_Q6_03	YY1	YY1	0.205565	3
V$E12_Q6	E12	TCF3	0.00426296	3
V$ELK1_02	Elk-1	ELK1	0.16063	3
V$E47_02	E47	TCF3	0.00426296	3
V$GATA1_02	GATA-1	GATA1	0.451234	3
V$PARP_Q3	PARP	PARP1	0.0915547	3
V$ETV3_02	ETV3	ETV3	0.0570047	3
V$GATA1_04	GATA-1	GATA1	0.451234	3
V$ER71_02	ER71	ETV2	0.263589	3
V$ERF_02	ERF	ERF	0.0890964	3
V$ELK1_06	ELK-1	ELK1	0.16063	3
V$MAZ_Q6	MAZ	MAZ	0.190726	3
V$SOX9_B1	SOX9	SOX9	0.0166297	3
V$CEBPB_Q6	C/EBPbeta	CEBPB	0.311773	3
V$ING4_01	ING4	ING4	0.318031	2
V$IRF7_Q3	IRF-7	IRF7	0.298676	2
V$ERR3_Q2	ERR3	ESRRG	0.390229	2
V$AML2_Q3	AML2	RUNX3	0.0445551	2
V$OC2_Q3	OC-2	ONECUT2	0.027775	2
V$HBP1_Q2	hbp1	HBP1	0.222192	2
V$POU6F1_03	POU6F1	POU6F1	0.209975	2
V$GATA3_02	GATA-3	GATA3	0.657182	2
V$GATA6_01	GATA-6	GATA6	0.0962413	2
V$FOXO4_02	FOXO4	FOXO4	0.689583	2
V$GATA2_02	GATA-2	GATA2	0.547199	2
V$AML1_Q6	AML1	RUNX1	0.185218	2
V$GATA1_06	GATA-1	GATA1	0.451234	2
V$TBX5_01	TBX5	TBX5	0.161411	2
V$TBX5_02	TBX5	TBX5	0.161411	2
V$GATA1_05	GATA-1	GATA1	0.451234	2
V$GR_Q6	GR	NR3C1	0.124036	2

V$E47_01	E47	TCF3	0.00426296	2
V$HNF3A_01	HNF3A	FOXA1	0.0941203	2
V$SOX9_Q4	SOX9	SOX9	0.0166297	2
V$YY1_01	YY1	YY1	0.205565	2
V$AML1_01	AML1a	RUNX1	0.185218	1
V$ERR3_Q2_01	ERR3	ESRRG	0.390229	1
V$NKX2B_Q3	NKX2B	NKX2-2	0.0895642	1
V$AML1_Q4	AML1	RUNX1	0.185218	1
V$GATA3_03	GATA-3	GATA3	0.657182	1
V$POU6F1_02	POU6F1	POU6F1	0.209975	1
V$TCF4_01	TCF-4	TCF7L2	0.247904	1
V$AML2_01	AML2	RUNX3	0.0445551	1
V$SOX10_Q6	SOX10	SOX10	0.0328733	1
V$GFI1_Q6	Gfi1	GFI1	0.0591466	1
V$AML2_Q3_01	AML2	RUNX3	0.0445551	1
V$GR_01	GR	NR3C1	0.124036	1
V$IRF1_Q6	IRF-1	IRF1	0.2485	1
V$IRF1_Q6_01	IRF-1	IRF1	0.2485	1

---

hsa-mir-222

Matrix_id	transcription factor	Gene	PCC	Occurrence
V$PUR1_Q4	PUR1	PURA	0.463487	7
V$YY1_Q6_02	YY1	YY1	0.369631	6
V$TBP_Q6	TBP	TBP	0.0141806	6
V$ELF1_Q6	Elf-1	ELF1	0.22436	6
V$SOX9_B1	SOX9	SOX9	0.046787	6
V$MAFB_01	MAFB	MAFB	0.180042	6
V$ETS1_B	c-Ets-1	ETS1	0.0754019	6
V$ARNT_01	Arnt	ARNT	0.0834991	6
V$GKLF_Q4	GKLF	KLF4	0.5273	6
V$YY1_Q6	YY1	YY1	0.369631	6
V$SOX9_Q4	SOX9	SOX9	0.046787	5
V$HNF3A_01	HNF3A	FOXA1	0.26256	5
V$PBX1_04	Pbx1	PBX1	0.231469	5
V$P300_01	p300	EP300	0.0808588	5
V$CETS1_Q6	C-ets-1	ETS1	0.0754019	5
V$SMAD4_Q6_01	Smad4	SMAD4	0.245999	5
V$GATA1_02	GATA-1	GATA1	0.178448	5
V$GR_Q6	GR	NR3C1	0.259306	5
V$YY1_02	YY1	YY1	0.369631	5
V$ETS2_B	c-Ets-2	ETS2	0.0181786	5
V$AHR_Q5	AhR	AHR	0.749224	4

V$GATA1_01	GATA-1	GATA1	0.178448	4
V$GABPBETA_Q3	GABP-beta	GABPB1	0.254984	4
V$IRF1_Q6_01	IRF-1	IRF1	0.0131248	4
V$YY1_Q6_03	YY1	YY1	0.369631	4
V$TCF4_01	TCF-4	TCF7L2	0.528252	4
V$GATA1_04	GATA-1	GATA1	0.178448	4
V$TFIII_Q6	TFII-I	GTF2I	0.158473	4
V$ETS2_Q6	c-Ets-2	ETS2	0.0181786	4
V$GATA3_03	GATA-3	GATA3	0.695873	4
V$FKLF_Q5	FKLF	KLF11	0.00589394	3
V$GR_01	GR	NR3C1	0.259306	3
V$IRF1_Q6	IRF-1	IRF1	0.0131248	3
V$IRF7_Q3	IRF-7	IRF7	0.241972	3
V$WT1_Q6	WT1	WT1	0.0326525	3
V$CP2_01	CP2	TFCP2	0.270964	3
V$YY1_03	YY1	YY1	0.369631	3
V$CEBPB_Q6	C/EBPbeta	CEBPB	0.283131	2
V$HTF4_Q2	HTF4	TCF12	0.0696102	2

hsa-mir-223

Matrix_id	transcription factor	Gene	PCC	Occurrence
V$GKLF_Q4	GKLF	KLF4	0.745264	40
V$MAFB_01	MAFB	MAFB	0.530797	40
V$ELF1_Q6	Elf-1	ELF1	0.126578	35
V$Elf5_03	ELF5	ELF5	0.270236	34
V$AML1_Q6	AML1	RUNX1	0.154739	33
V$ETS2_Q6	c-Ets-2	ETS2	0.333527	32
V$SPI1_03	SPI1	SPI1	0.63079	32
V$TTF1_Q5	TTF-1	NKX2-1	0.372633	32
V$SPI1_Q5	SPI1	SPI1	0.63079	31
V$ETS2_B	c-Ets-2	ETS2	0.333527	31
V$YY1_01	YY1	YY1	0.0158048	30
V$FOXO3A_Q1	FOXO3A	FOXO3	0.24908	24
V$EHF_03	EHF	EHF	0.249606	22
V$IRF8_Q6	IRF-8	IRF8	0.244708	22
V$GATA3_01	GATA-3	GATA3	0.18934	22
V$AML1_01	AML1a	RUNX1	0.154739	22
V$ESE1_Q3	ESE-1	ELF3	0.371966	22
V$HNF3B_Q6	HNF-3beta	FOXA2	0.153113	21
V$CP2_01	CP2	TFCP2	0.129278	21
V$GATA2_02	GATA-2	GATA2	0.161776	21
V$GATA6_01	GATA-6	GATA6	0.222627	21

V$AML1_Q4	AML1	RUNX1	0.154739	20
V$STAT3_03	STAT3	STAT3	0.218242	19
V$GATA2_01	GATA-2	GATA2	0.161776	19
V$GATA3_02	GATA-3	GATA3	0.18934	16
V$YY1_Q6_03	YY1	YY1	0.0158048	15
V$AML2_01	AML2	RUNX3	0.315324	15
V$ESE1_02	ESE-1	ELF3	0.371966	15
V$ELF5_01	ELF5	ELF5	0.270236	15
V$CEBPG_Q6_01	C/EBPgamma	CEBPG	0.366348	13
V$CEBPD_Q6	C/EBPdelta	CEBPD	0.805267	13
V$CEBPB_Q6	C/EBPbeta	CEBPB	0.631874	12
V$FOXO4_02	FOXO4	FOXO4	0.247532	11
V$STAT1_05	STAT1	STAT1	0.146477	10
V$STAT1_Q6	STAT1	STAT1	0.146477	7
V$IRF7_01	IRF-7	IRF7	0.436966	7
V$GATA2_03	GATA-2	GATA2	0.161776	6
V$NUR77_Q5	NUR77	NR4A1	0.123743	5
V$IRF1_Q6_01	IRF-1	IRF1	0.227571	2

----

hsa-mir-224

Matrix_id	transcription factor	Gene	PCC	Occurrence
V$PEA3_Q6	PEA3	ETV4	0.0725604	248
V$PARP_Q4	PARP	PARP1	0.0571376	245
V$ELF1_Q6	Elf-1	ELF1	0.202112	241
V$P300_01	p300	EP300	0.319459	227
V$ETS1_B	c-Ets-1	ETS1	0.0636973	223
V$CETS1_Q6	C-ets-1	ETS1	0.0636973	218
V$SMAD4_Q6_01	Smad4	SMAD4	0.291295	215
V$GABPA_Q4	GABP-alpha	GABPA	0.335774	206
V$TBP_Q6	TBP	TBP	0.12449	195
V$TBX5_02	TBX5	TBX5	0.0523812	194
V$YY1_01	YY1	YY1	0.367911	187
V$DLX5_01	dlx5	DLX5	0.663186	173
V$YY1_Q6_02	YY1	YY1	0.367911	171
V$YY1_Q6	YY1	YY1	0.367911	171
V$GATA1_02	GATA-1	GATA1	0.0264886	168
V$GATA1_06	GATA-1	GATA1	0.0264886	162
V$GATA2_02	GATA-2	GATA2	0.697301	162
V$GATA1_05	GATA-1	GATA1	0.0264886	162
V$AHR_Q5	AhR	AHR	0.968232	160
V$ERBETA_Q5	ER-beta	ESR2	0.0107349	152
V$PARP_Q3	PARP	PARP1	0.0571376	151

V$GATA1_04	GATA-1	GATA1	0.0264886	142
V$HMGIY_01	HMGIY	HMGA1	0.335188	142
V$GATA3_02	GATA-3	GATA3	0.918317	139
V$GATA3_01	GATA-3	GATA3	0.918317	131
V$YY1_Q6_03	YY1	YY1	0.367911	127
V$CEBPE_Q6	CEBPE	CEBPE	0.0507636	118
V$GATA2_01	GATA-2	GATA2	0.697301	118
V$TCF4_01	TCF-4	TCF7L2	0.469584	116
V$CEBPA_Q6	C/EBPalpha	CEBPA	0.0611198	113
V$TCF4_Q5	TCF-4	TCF7L2	0.469584	106
V$PITX2_Q2	Pitx2	PITX2	0.41153	105
V$FOXJ2_01	FOXJ2	FOXJ2	0.0260835	103
V$CEBPD_Q6	C/EBPdelta	CEBPD	0.426639	93
V$HBP1_Q2	hbp1	HBP1	0.649891	88
V$CEBPB_02	C/EBPbeta	CEBPB	0.581277	86
V$CEBPG_Q6_01	C/EBPgamma	CEBPG	0.531679	80
V$GATA2_03	GATA-2	GATA2	0.697301	75
V$CEBPB_Q6	C/EBPbeta	CEBPB	0.581277	72
V$YY1_02	YY1	YY1	0.367911	71
V$CEBPA_01	C/EBPalpha	CEBPA	0.0611198	69
V$YY1_03	YY1	YY1	0.367911	56
V$IRF1_Q6_01	IRF-1	IRF1	0.330883	47
V$CEBPG_Q6	C/EBPgamma	CEBPG	0.531679	46
V$CEBPB_01	C/EBPbeta	CEBPB	0.581277	44
V$CDP_04	CDP	CUX1	0.379583	36
V$FOXO4_01	FOXO4	FOXO4	0.957626	35
V$FOXJ2_02	FOXJ2	FOXJ2	0.0260835	31
V$IRF7_01	IRF-7	IRF7	0.157129	29
V$IRF2_01	IRF-2	IRF2	0.087711	16

----

hsa-mir-23a

Matrix_id	transcription factor	Gene	PCC	Occurrence
V$PUR1_Q4	PUR1	PURA	0.201518	222
V$PEA3_Q6	PEA3	ETV4	0.139465	217
V$PARP_Q4	PARP	PARP1	0.0384419	214
V$GKLF_Q4	GKLF	KLF4	0.640138	211
V$ELF1_Q6	Elf-1	ELF1	0.210303	210
V$MAFB_01	MAFB	MAFB	0.211536	206
V$P300_01	p300	EP300	0.335915	204
V$ETS1_B	c-Ets-1	ETS1	0.153788	196
V$CETS1_Q6	C-ets-1	ETS1	0.153788	192
V$NFAT4_Q3	NF-AT4	NFATC3	0.0873675	185

V$GATA1_01	GATA-1	GATA1	0.184953	181
V$ETS2_Q6	c-Ets-2	ETS2	0.0517497	181
V$GABPA_Q4	GABP-alpha	GABPA	0.174132	180
V$TBX5_02	TBX5	TBX5	0.132633	178
V$TBP_Q6	TBP	TBP	0.134487	170
V$YY1_01	YY1	YY1	0.390864	167
V$ETS2_B	c-Ets-2	ETS2	0.0517497	166
V$AML1_Q6	AML1	RUNX1	0.167202	161
V$YY1_Q6	YY1	YY1	0.390864	150
V$YY1_Q6_02	YY1	YY1	0.390864	150
V$DLX5_01	dlx5	DLX5	0.579841	149
V$E12_Q6	E12	TCF3	0.0360801	141
V$E2A_Q6	E2A	TCF3	0.0360801	141
V$TBX5_01	TBX5	TBX5	0.132633	140
V$E47_02	E47	TCF3	0.0360801	139
V$AP2ALPHA_Q6	AP-2alpha	TFAP2A	0.907529	134
V$IRF4_Q6	IRF-4	IRF4	0.00040913	134
V$NFAT2_Q5	NF-AT2	NFATC1	0.00928479	125
V$FKLF_Q5	FKLF	KLF11	0.213048	124
V$HNF3A_01	HNF3A	FOXA1	0.0839298	124
V$GATA3_01	GATA-3	GATA3	0.907516	118
V$HMGIY_01	HMGIY	HMGA1	0.31888	118
V$E2A_Q2	E2A	TCF3	0.0360801	117
V$FOXO3A_Q1	FOXO3A	FOXO3	0.0544921	116
V$TFIII_Q6	TFII-I	GTF2I	0.151168	115
V$SREBP1_Q6	SREBP-1	SREBF1	0.157358	113
V$TEL1_02	TEL1	ETV6	0.0455432	108
V$ELK1_02	Elk-1	ELK1	0.072076	108
V$GATA2_01	GATA-2	GATA2	0.760531	107
V$YY1_Q6_03	YY1	YY1	0.390864	107
V$ER71_02	ER71	ETV2	0.0323767	99
V$ELK1_06	ELK-1	ELK1	0.072076	98
V$AML1_01	AML1a	RUNX1	0.167202	93
V$PITX2_Q2	Pitx2	PITX2	0.459572	92
V$ETV7_01	ETV7	ETV7	0.00486198	92
V$TCF4_Q5	TCF-4	TCF7L2	0.486527	90
V$ESE1_Q3	ESE-1	ELF3	0.204657	88
V$GABPBETA_Q3	GABP-beta	GABPB1	0.283782	87
V$PITX2_01	PITX2	PITX2	0.459572	87
V$CREM_Q6	CREM	CREM	0.138503	86
V$ZABC1_01	ZABC1	ZNF217	0.831181	83
V$E47_01	E47	TCF3	0.0360801	80
V$AML1_Q4	AML1	RUNX1	0.167202	79
V$GATA3_03	GATA-3	GATA3	0.907516	77
V$ESE1_02	ESE-1	ELF3	0.204657	66
V$FOXO4_02	FOXO4	FOXO4	0.898954	62

V$ATF3_Q6_01	ATF-3	ATF3	0.61725	62
V$ATF1_Q6_01	ATF-1	ATF1	0.0592608	62
V$SPIB_03	Spi-B	SPIB	0.0442871	59
V$P53_02	p53	TP53	0.617343	57
V$PET1_02	Pet-1	FEV	0.114676	56
V$ERG_03	ERG	ERG	0.352309	52
V$ERG_01	ERG	ERG	0.352309	33
V$AP2ALPHA_02	AP-2alphaA	TFAP2A	0.907529	30
V$NKX3A_02	Nkx3A	NKX3-1	0.133344	16
V$P53_04	p53	TP53	0.617343	7

----

hsa-mir-24-1

Matrix_id	transcription factor	Gene	PCC	Occurrence
V$PUR1_Q4	PUR1	PURA	0.24931	239
V$PEA3_Q6	PEA3	ETV4	0.0994133	234
V$PARP_Q4	PARP	PARP1	0.0530058	231
V$ELF1_Q6	Elf-1	ELF1	0.122152	227
V$GKLF_Q4	GKLF	KLF4	0.589086	226
V$MAFB_01	MAFB	MAFB	0.253642	220
V$P300_01	p300	EP300	0.344535	216
V$CETS1_Q6	C-ets-1	ETS1	0.207348	205
V$SMAD4_Q6_01	Smad4	SMAD4	0.380773	200
V$ETS2_Q6	c-Ets-2	ETS2	0.0294561	196
V$NR1B2_Q6	NR1B2	RARB	0.0381988	195
V$GABPA_Q4	GABP-alpha	GABPA	0.283468	194
V$GATA1_01	GATA-1	GATA1	0.246191	194
V$TBP_Q6	TBP	TBP	0.155753	183
V$GR_Q6	GR	NR3C1	0.218397	183
V$YY1_01	YY1	YY1	0.358358	178
V$AML1_Q6	AML1	RUNX1	0.127865	172
V$LRF_Q2	LRF	ZBTB7A	0.112859	169
V$DLX5_01	dlx5	DLX5	0.587562	162
V$YY1_Q6	YY1	YY1	0.358358	160
V$YY1_Q6_02	YY1	YY1	0.358358	160
V$AHR_Q5	AhR	AHR	0.93087	152
V$ERBETA_Q5	ER-beta	ESR2	0.0241903	149
V$PARP_Q3	PARP	PARP1	0.0530058	148
V$E2A_Q6	E2A	TCF3	0.00233027	146
V$E12_Q6	E12	TCF3	0.00233027	146
V$E47_02	E47	TCF3	0.00233027	144
V$ING4_01	ING4	ING4	0.0408535	144
V$AP2ALPHA_Q6	AP-2alpha	TFAP2A	0.932552	142

V$SP1_Q6	Sp1	SP1	0.429211	141
V$HNF3A_01	HNF3A	FOXA1	0.0379043	134
V$AP2ALPHA_01	AP-2alpha	TFAP2A	0.932552	133
V$SP1_Q6_01	Sp1	SP1	0.429211	129
V$SP1_Q4_01	Sp1	SP1	0.429211	124
V$SP1_01	Sp1	SP1	0.429211	121
V$SP1_Q2_01	Sp1	SP1	0.429211	121
V$ELK1_02	Elk-1	ELK1	0.0924981	121
V$TEL1_02	TEL1	ETV6	0.0306096	121
V$CMAF_01	c-Maf	MAF	0.113545	118
V$ARNT_01	Arnt	ARNT	0.394212	117
V$TEF1_Q6_03	TEF-1	TEAD1	0.0143519	115
V$SP1_02	SP1	SP1	0.429211	114
V$ER71_02	ER71	ETV2	0.0794144	113
V$ELK1_06	ELK-1	ELK1	0.0924981	111
V$CEBPE_Q6	CEBPE	CEBPE	0.263541	109
V$CEBPA_Q6	C/EBPalpha	CEBPA	0.0119739	109
V$ETV7_01	ETV7	ETV7	0.00707411	105
V$GFI1B_01	Gfi1b	GFI1B	0.045916	103
V$AML1_01	AML1a	RUNX1	0.127865	100
V$PITX2_Q2	Pitx2	PITX2	0.458122	99
V$ESE1_Q3	ESE-1	ELF3	0.151935	97
V$AP2GAMMA_01	AP-2gamma	TFAP2C	0.858973	96
V$PITX2_01	PITX2	PITX2	0.458122	96
V$FOXJ2_01	FOXJ2	FOXJ2	0.0878174	95
V$ZABC1_01	ZABC1	ZNF217	0.779205	89
V$GABPBETA_Q3	GABP-beta	GABPB1	0.283621	89
V$SP2_01	SP2	SP2	0.268898	88
V$CEBPD_Q6	C/EBPdelta	CEBPD	0.436878	86
V$AML1_Q4	AML1	RUNX1	0.127865	86
V$GATA3_03	GATA-3	GATA3	0.857401	83
V$CEBPB_02	C/EBPbeta	CEBPB	0.572352	82
V$HOXA9_01	hoxa9	HOXA9	0.0218861	76
V$ESE1_02	ESE-1	ELF3	0.151935	74
V$LHX3b_01	LHX3b	LHX3	0.021562	70
V$STAT3_03	STAT3	STAT3	0.157349	68
V$CEBPA_01	C/EBPalpha	CEBPA	0.0119739	67
V$FOXO4_02	FOXO4	FOXO4	0.924163	65
V$CEBPB_Q6	C/EBPbeta	CEBPB	0.572352	64
V$MSX1_01	Msx-1	MSX1	0.0407685	62
V$PET1_02	Pet-1	FEV	0.0870979	58
V$ERG_03	ERG	ERG	0.379452	54
V$CEBPB_01	C/EBPbeta	CEBPB	0.572352	40
V$PAX3_B	Pax-3	PAX3	0.116646	36
V$AFP1_Q6	AFP1	ZFHX3	0.274016	8

----

hsa-mir-24-2

Matrix_id	transcription factor	Gene	PCC	Occurrence
V$PUR1_Q4	PUR1	PURA	0.24931	239
V$PEA3_Q6	PEA3	ETV4	0.0994133	234
V$PARP_Q4	PARP	PARP1	0.0530058	231
V$ELF1_Q6	Elf-1	ELF1	0.122152	227
V$GKLF_Q4	GKLF	KLF4	0.589086	226
V$MAFB_01	MAFB	MAFB	0.253642	220
V$P300_01	p300	EP300	0.344535	216
V$ETS1_B	c-Ets-1	ETS1	0.207348	211
V$CETS1_Q6	C-ets-1	ETS1	0.207348	205
V$ETS2_Q6	c-Ets-2	ETS2	0.0294561	196
V$GABPA_Q4	GABP-alpha	GABPA	0.283468	194
V$GATA1_01	GATA-1	GATA1	0.246191	194
V$TBX5_02	TBX5	TBX5	0.171306	187
V$TBP_Q6	TBP	TBP	0.155753	183
V$ETS2_B	c-Ets-2	ETS2	0.0294561	180
V$YY1_01	YY1	YY1	0.358358	178
V$AML1_Q6	AML1	RUNX1	0.127865	172
V$DLX5_01	dlx5	DLX5	0.587562	162
V$YY1_Q6_02	YY1	YY1	0.358358	160
V$YY1_Q6	YY1	YY1	0.358358	160
V$TBX5_01	TBX5	TBX5	0.171306	150
V$E12_Q6	E12	TCF3	0.00233027	146
V$E2A_Q6	E2A	TCF3	0.00233027	146
V$ING4_01	ING4	ING4	0.0408535	144
V$E47_02	E47	TCF3	0.00233027	144
V$AP2ALPHA_Q6	AP-2alpha	TFAP2A	0.932552	142
V$HNF3A_01	HNF3A	FOXA1	0.0379043	134
V$FKLF_Q5	FKLF	KLF11	0.289596	131
V$HMGIY_01	HMGIY	HMGA1	0.249456	130
V$TFIII_Q6	TFII-I	GTF2I	0.178901	128
V$GATA3_01	GATA-3	GATA3	0.857401	124
V$E2A_Q2	E2A	TCF3	0.00233027	123
V$TEL1_02	TEL1	ETV6	0.0306096	121
V$SREBP1_Q6	SREBP-1	SREBF1	0.118821	121
V$ELK1_02	Elk-1	ELK1	0.0924981	121
V$YY1_Q6_03	YY1	YY1	0.358358	114
V$ER71_02	ER71	ETV2	0.0794144	113
V$ELK1_06	ELK-1	ELK1	0.0924981	111
V$GATA2_01	GATA-2	GATA2	0.753068	111
V$ETV7_01	ETV7	ETV7	0.00707411	105

V$AML1_01	AML1a	RUNX1	0.127865	100
V$PITX2_Q2	Pitx2	PITX2	0.458122	99
V$ESE1_Q3	ESE-1	ELF3	0.151935	97
V$PITX2_01	PITX2	PITX2	0.458122	96
V$TCF4_Q5	TCF-4	TCF7L2	0.481077	95
V$CREM_Q6	CREM	CREM	0.148719	90
V$GABPBETA_Q3	GABP-beta	GABPB1	0.283621	89
V$ZABC1_01	ZABC1	ZNF217	0.779205	89
V$AML1_Q4	AML1	RUNX1	0.127865	86
V$GATA3_03	GATA-3	GATA3	0.857401	83
V$E47_01	E47	TCF3	0.00233027	81
V$ESE1_02	ESE-1	ELF3	0.151935	74
V$FOXO4_02	FOXO4	FOXO4	0.924163	65
V$ATF1_Q6_01	ATF-1	ATF1	0.0803885	64
V$MSX1_01	Msx-1	MSX1	0.0407685	62
V$ATF3_Q6_01	ATF-3	ATF3	0.612585	61
V$SPIB_03	Spi-B	SPIB	0.0607785	61
V$P53_02	p53	TP53	0.546691	60
V$PET1_02	Pet-1	FEV	0.0870979	58
V$ERG_03	ERG	ERG	0.379452	54
V$ERG_01	ERG	ERG	0.379452	35
V$AP2ALPHA_02	AP-2alphaA	TFAP2A	0.932552	33
V$NKX3A_02	Nkx3A	NKX3-1	0.0742997	16
V$P53_04	p53	TP53	0.546691	7

----

hsa-mir-26a-1

Matrix_id	transcription factor	Gene	PCC	Occurrence
V$PUR1_Q4	PUR1	PURA	0.563907	16
V$P300_01	p300	EP300	0.11525	15
V$ETS2_Q6	c-Ets-2	ETS2	0.117818	15
V$SMAD4_Q6_01	Smad4	SMAD4	0.421393	15
V$GKLF_Q4	GKLF	KLF4	0.236903	15
V$SMAD3_Q6_01	Smad3	SMAD3	0.0341764	13
V$AP4_Q6_02	AP-4	TFAP4	0.197706	12
V$MEF2C_Q4	MEF-2C	MEF2C	0.0954616	12
V$GATA1_01	GATA-1	GATA1	0.0821563	11
V$BEN_01	BEN	GTF2IRD1	0.030314	10
V$TFIII_Q6	TFII-I	GTF2I	0.156115	9
V$ZBP89_Q4	ZBP89	ZNF148	0.357365	8
V$WT1_Q6	WT1	WT1	0.571403	8
V$EHF_03	EHF	EHF	0.0325475	7
V$SREBP1_Q6	SREBP-1	SREBF1	0.0254278	7

V$SP1_Q4_01	Sp1	SP1	0.031628	6
V$WT1_Q6_01	WT1	WT1	0.571403	6
V$SP1_Q6	Sp1	SP1	0.031628	6
V$HIF1A_Q6	HIF-1alpha	HIF1A	0.433227	6
V$ARNT_01	Arnt	ARNT	0.120178	6
V$FKLF_Q5	FKLF	KLF11	0.0902283	6
V$CREM_Q6	CREM	CREM	0.0974006	6
V$SP1_Q2_01	Sp1	SP1	0.031628	6
V$SP1_02	SP1	SP1	0.031628	6
V$SP1_Q6_01	Sp1	SP1	0.031628	6
V$ZFX_01	Zfx	ZFX	0.157093	5
V$GABPBETA_Q3	GABP-beta	GABPB1	0.535031	5
V$AP2ALPHA_Q6	AP-2alpha	TFAP2A	0.0853216	5
V$CACCCBINDINGFACT	CACCC-binding factor	ZNF148	0.357365	4
V$AP2GAMMA_01	AP-2gamma	TFAP2C	0.188662	4
V$AP2ALPHA_01	AP-2alpha	TFAP2A	0.0853216	3
V$EGR1_02	EGR-1	EGR1	0.0478022	3
V$SREBP1_02	SREBP-1	SREBF1	0.0254278	3
V$DEC2_Q2	2-Dec	BHLHE41	0.323923	3
V$ATF1_Q6_01	ATF-1	ATF1	0.540648	2
V$MTF1_Q4	MTF-1	MTF1	0.0140442	2
V$ATF6_01	ATF6	ATF6	0.0363864	2
V$PAX3_01	Pax-3	PAX3	0.0816502	1
V$CTCF_01	CTCF	CTCF	0.128407	1
V$CTCF_02	CTCF	CTCF	0.128407	1

---

hsa-mir-26a-2

Matrix_id	transcription factor	Gene	PCC	Occurrence
V$PUR1_Q4	PUR1	PURA	0.563907	16
V$P300_01	p300	EP300	0.11525	15
V$SMAD4_Q6_01	Smad4	SMAD4	0.421393	15
V$GATA1_05	GATA-1	GATA1	0.0821563	14
V$GATA2_02	GATA-2	GATA2	0.380356	14
V$NANOG_02	Nanog	NANOG	0.0279849	14
V$GATA1_06	GATA-1	GATA1	0.0821563	14
V$GATA6_01	GATA-6	GATA6	0.0993605	14
V$YY1_01	YY1	YY1	0.370567	13
V$SMAD3_Q6_01	Smad3	SMAD3	0.0341764	13
V$SRY_02	SRY	SRY	0.365856	13
V$GATA3_02	GATA-3	GATA3	0.0594854	12
V$ETS2_B	c-Ets-2	ETS2	0.117818	12
V$GR_Q6	GR	NR3C1	0.478103	12

V$AP4_Q6_02	AP-4	TFAP4	0.197706	12
V$TTF1_Q5	TTF-1	NKX2-1	0.102028	12
V$HNF3A_01	HNF3A	FOXA1	0.206352	11
V$GATA1_01	GATA-1	GATA1	0.0821563	11
V$FOXJ2_01	FOXJ2	FOXJ2	0.00835808	11
V$FOXO3A_Q1	FOXO3A	FOXO3	0.166433	11
V$YY1_Q6	YY1	YY1	0.370567	11
V$YY1_Q6_02	YY1	YY1	0.370567	10
V$PITX2_Q2	Pitx2	PITX2	0.0960251	9
V$PITX2_01	PITX2	PITX2	0.0960251	9
V$HOXD9_Q2	Hoxd9	HOXD9	0.180937	9
V$HOXA9_01	hoxa9	HOXA9	0.316058	9
V$TCF4_01	TCF-4	TCF7L2	0.611655	9
V$GATA3_01	GATA-3	GATA3	0.0594854	9
V$YY1_Q6_03	YY1	YY1	0.370567	9
V$GR_01	GR	NR3C1	0.478103	8
V$PBX1_Q3	Pbx1	PBX1	0.866374	8
V$TCF4_Q5	TCF-4	TCF7L2	0.611655	8
V$CMAF_01	c-Maf	MAF	0.237521	8
V$SP1_01	Sp1	SP1	0.031628	7
V$MITF_Q6	MITF	MITF	0.463916	7
V$FAC1_01	FAC1	BPTF	0.590496	7
V$BCL6_Q3_01	Bcl-6	BCL6	0.0362302	7
V$DBP_Q6_01	DBP	DBP	0.0787586	6
V$AP4_Q5	AP-4	TFAP4	0.197706	6
V$TEF1_Q6_03	TEF-1	TEAD1	0.00248875	6
V$GATA2_01	GATA-2	GATA2	0.380356	6
V$SP1_Q6	Sp1	SP1	0.031628	6
V$HOX13_02	HOXA5	HOXA5	0.119913	6
V$CEBPD_Q6	C/EBPdelta	CEBPD	0.11041	6
V$TEF1_Q6	TEF-1	TEAD1	0.00248875	6
V$SP1_Q6_01	Sp1	SP1	0.031628	6
V$SP1_Q4_01	Sp1	SP1	0.031628	6
V$SP1_Q2_01	Sp1	SP1	0.031628	6
V$GATA3_03	GATA-3	GATA3	0.0594854	6
V$SP1_02	SP1	SP1	0.031628	6
V$FOXO4_02	FOXO4	FOXO4	0.0164455	6
V$GATA2_03	GATA-2	GATA2	0.380356	5
V$AP4_Q6	AP-4	TFAP4	0.197706	5
V$FOXO4_01	FOXO4	FOXO4	0.0164455	3
V$AP4_Q6_01	AP-4	TFAP4	0.197706	3

----

hsa-mir-26b

Matrix_id	transcription factor	Gene	PCC	Occurrence
V$PUR1_Q4	PUR1	PURA	0.37632	113
V$ELF1_Q6	Elf-1	ELF1	0.261652	107
V$GKLF_Q4	GKLF	KLF4	0.58038	105
V$P300_01	p300	EP300	0.285296	104
V$MAFB_01	MAFB	MAFB	0.320268	101
V$ETS1_B	c-Ets-1	ETS1	0.0232732	99
V$SMAD4_Q6_01	Smad4	SMAD4	0.422451	96
V$CETS1_Q6	C-ets-1	ETS1	0.0232732	95
V$GABPA_Q4	GABP-alpha	GABPA	0.193552	90
V$GATA1_01	GATA-1	GATA1	0.121132	89
V$TTF1_Q5	TTF-1	NKX2-1	0.0454847	83
V$YY1_Q6_02	YY1	YY1	0.469256	80
V$YY1_Q6	YY1	YY1	0.469256	78
V$GATA1_02	GATA-1	GATA1	0.121132	76
V$WT1_Q6	WT1	WT1	0.156484	72
V$GATA2_02	GATA-2	GATA2	0.739507	72
V$GATA1_06	GATA-1	GATA1	0.121132	72
V$GATA1_05	GATA-1	GATA1	0.121132	72
V$SP1_Q6	Sp1	SP1	0.247414	69
V$AHR_Q5	AhR	AHR	0.887024	69
V$AP2ALPHA_Q6	AP-2alpha	TFAP2A	0.774806	66
V$TFIII_Q6	TFII-I	GTF2I	0.110465	65
V$FKLF_Q5	FKLF	KLF11	0.272347	64
V$SP1_Q6_01	Sp1	SP1	0.247414	63
V$CMAF_01	c-Maf	MAF	0.267866	63
V$GATA1_04	GATA-1	GATA1	0.121132	60
V$SP1_Q4_01	Sp1	SP1	0.247414	60
V$AP2ALPHA_01	AP-2alpha	TFAP2A	0.774806	60
V$SP1_Q2_01	Sp1	SP1	0.247414	59
V$HIF1A_Q6	HIF-1alpha	HIF1A	0.115015	58
V$ARNT_01	Arnt	ARNT	0.270966	58
V$YY1_Q6_03	YY1	YY1	0.469256	57
V$SP1_01	Sp1	SP1	0.247414	56
V$SP1_02	SP1	SP1	0.247414	56
V$WT1_Q6_01	WT1	WT1	0.156484	56
V$GATA3_02	GATA-3	GATA3	0.749806	56
V$SREBP1_Q6	SREBP-1	SREBF1	0.130423	55
V$CP2_01	CP2	TFCP2	0.341041	54
V$STAT3_03	STAT3	STAT3	0.0259077	40
V$CNOT3_01	CNOT3	CNOT3	0.0563983	38
V$ZABC1_01	ZABC1	ZNF217	0.722526	37
V$GATA2_03	GATA-2	GATA2	0.739507	34
V$YY1_02	YY1	YY1	0.469256	34
V$EAR2_Q2	EAR2	NR2F6	0.28209	34

V$YY1_03	YY1	YY1	0.469256	31
V$PDEF_02	PDEF	SPDEF	0.251218	28
V$CEBPG_Q6	C/EBPgamma	CEBPG	0.340389	19
V$SP3_Q3	Sp3	SP3	0.629746	17
V$ERM_02	Erm	ETV5	0.245617	15
V$AP2ALPHA_02	AP-2alphaA	TFAP2A	0.774806	8
V$AP2ALPHA_03	AP-2alphaA	TFAP2A	0.774806	3

---

hsa-mir-27a

Matrix_id	transcription factor	Gene	PCC	Occurrence
V$PUR1_Q4	PUR1	PURA	0.242899	287
V$PEA3_Q6	PEA3	ETV4	0.247266	280
V$PARP_Q4	PARP	PARP1	0.170886	275
V$ELF1_Q6	Elf-1	ELF1	0.117852	273
V$GKLF_Q4	GKLF	KLF4	0.619513	270
V$AP2REP_01	AP-2rep	KLF12	0.081282	269
V$MAFB_01	MAFB	MAFB	0.282625	266
V$P300_01	p300	EP300	0.48754	259
V$ETS1_B	c-Ets-1	ETS1	0.191012	255
V$CETS1_Q6	C-ets-1	ETS1	0.191012	245
V$NFAT4_Q3	NF-AT4	NFATC3	0.0538092	241
V$GABPA_Q4	GABP-alpha	GABPA	0.347079	233
V$ETS2_Q6	c-Ets-2	ETS2	0.125731	231
V$GATA1_01	GATA-1	GATA1	0.207548	229
V$TBX5_02	TBX5	TBX5	0.249495	225
V$TBP_Q6	TBP	TBP	0.19432	221
V$CDX2_Q5_02	CDX-2	CDX2	0.0485701	221
V$ETS2_B	c-Ets-2	ETS2	0.125731	218
V$YY1_01	YY1	YY1	0.454413	217
V$NANOG_02	Nanog	NANOG	0.0664269	209
V$AML1_Q6	AML1	RUNX1	0.205498	209
V$YY1_Q6	YY1	YY1	0.454413	196
V$YY1_Q6_02	YY1	YY1	0.454413	195
V$DLX5_01	dlx5	DLX5	0.644187	194
V$IPF1_01	IPF1	PDX1	0.0996363	184
V$TBX5_01	TBX5	TBX5	0.249495	182
V$E12_Q6	E12	TCF3	0.0988569	176
V$E2A_Q6	E2A	TCF3	0.0988569	176
V$ING4_01	ING4	ING4	0.0380184	174
V$E47_02	E47	TCF3	0.0988569	174
V$MYOD_Q6_01	MyoD	MYOD1	8.91E-05	174
V$IRF4_Q6	IRF-4	IRF4	0.120039	172

V$AP2ALPHA_Q6	AP-2alpha	TFAP2A	0.956865	168
V$CDX2_01	Cdx-2	CDX2	0.0485701	164
V$HNF3A_01	HNF3A	FOXA1	0.0591774	163
V$MYOGENIN_Q6	myogenin	MYOG	0.0578974	161
V$FKLF_Q5	FKLF	KLF11	0.293623	159
V$NFAT2_Q5	NF-AT2	NFATC1	0.103893	157
V$HMGIY_01	HMGIY	HMGA1	0.293527	155
V$FOXO3A_Q1	FOXO3A	FOXO3	0.0882284	155
V$GATA3_01	GATA-3	GATA3	0.880875	153
V$TFIII_Q6	TFII-I	GTF2I	0.226698	150
V$E2A_Q2	E2A	TCF3	0.0988569	148
V$TEL1_02	TEL1	ETV6	0.158641	147
V$ELK1_02	Elk-1	ELK1	0.184386	147
V$SREBP1_Q6	SREBP-1	SREBF1	0.173941	144
V$YY1_Q6_03	YY1	YY1	0.454413	141
V$ER71_02	ER71	ETV2	0.0488207	139
V$GATA2_01	GATA-2	GATA2	0.799861	139
V$ELK1_06	ELK-1	ELK1	0.184386	135
V$CRX_Q4	Crx	CRX	0.0384555	135
V$ETV7_01	ETV7	ETV7	0.136813	127
V$PITX2_Q2	Pitx2	PITX2	0.566223	123
V$ESE1_Q3	ESE-1	ELF3	0.162555	117
V$TCF4_Q5	TCF-4	TCF7L2	0.488612	116
V$AML1_01	AML1a	RUNX1	0.205498	116
V$RFX1_02	RFX1	RFX1	0.0865738	116
V$PITX2_01	PITX2	PITX2	0.566223	116
V$PITX1_01	Pitx1	PITX1	0.0675662	115
V$GABPBETA_Q3	GABP-beta	GABPB1	0.380156	110
V$ZABC1_01	ZABC1	ZNF217	0.830616	109
V$CREM_Q6	CREM	CREM	0.252268	108
V$MYOD_Q6	MyoD	MYOD1	8.91E-05	106
V$RFX1_01	RFX1	RFX1	0.0865738	105
V$CRX_02	Crx	CRX	0.0384555	102
V$AML1_Q4	AML1	RUNX1	0.205498	100
V$E47_01	E47	TCF3	0.0988569	99
V$GATA3_03	GATA-3	GATA3	0.880875	94
V$ESE1_02	ESE-1	ELF3	0.162555	88
V$ATF1_Q6_01	ATF-1	ATF1	0.17353	80
V$FOXO4_02	FOXO4	FOXO4	0.94623	78
V$P53_02	p53	TP53	0.62493	76
V$MSX1_01	Msx-1	MSX1	0.0701105	74
V$ATF3_Q6_01	ATF-3	ATF3	0.676997	72
V$SPIB_03	Spi-B	SPIB	0.0501615	70
V$PET1_02	Pet-1	FEV	0.103829	69
V$ERG_03	ERG	ERG	0.482871	65
V$AP2ALPHA_02	AP-2alphaA	TFAP2A	0.956865	43

V$ERG_01	ERG	ERG	0.482871	42
V$BACH2_01	Bach2	BACH2	0.0367081	23
V$NKX3A_02	Nkx3A	NKX3-1	0.0355355	17
V$P53_04	p53	TP53	0.62493	7

----

hsa-mir-27b

Matrix_id	transcription factor	Gene	PCC	Occurrence
V$AML1_01	AML1a	RUNX1	0.135358	2
V$SOX9_Q4	SOX9	SOX9	0.140409	2
V$HNF3A_01	HNF3A	FOXA1	0.393584	2
V$AML1_Q6	AML1	RUNX1	0.135358	2
V$MAFB_01	MAFB	MAFB	0.0204788	2
V$LHX3b_01	LHX3b	LHX3	0.124667	2
V$ETS2_Q6	c-Ets-2	ETS2	0.00948282	2
V$ELF1_Q6	Elf-1	ELF1	0.010526	2
V$LRF_Q2	LRF	ZBTB7A	0.204395	2
V$SMAD4_Q6_01	Smad4	SMAD4	0.252422	2
V$IRF4_Q6	IRF-4	IRF4	0.00665002	2
V$CMAF_01	c-Maf	MAF	0.117095	2
V$DBP_Q6_01	DBP	DBP	0.0172768	2
V$CETS1_Q6	C-ets-1	ETS1	0.376091	2
V$HOXD9_Q2	Hoxd9	HOXD9	0.246169	2
V$AP4_Q6_02	AP-4	TFAP4	0.119728	2
V$GKLF_Q4	GKLF	KLF4	0.183853	2
V$AML2_01	AML2	RUNX3	0.0202897	2
V$TBP_Q6	TBP	TBP	0.00279141	2
V$AML1_Q4	AML1	RUNX1	0.135358	2
V$P300_01	p300	EP300	0.00860273	2
V$PUR1_Q4	PUR1	PURA	0.468624	2
V$YY1_01	YY1	YY1	0.120744	2
V$GATA1_01	GATA-1	GATA1	0.536762	2
V$ZABC1_01	ZABC1	ZNF217	0.0450992	2
V$CEBPB_02	C/EBPbeta	CEBPB	0.0410464	2
V$SRY_02	SRY	SRY	0.0171504	2
V$GR_Q6	GR	NR3C1	0.212841	2
V$NR1B2_Q6	NR1B2	RARB	0.19589	2
V$NCX_02	Ncx	TLX2	0.0532897	2
V$DLX5_01	dlx5	DLX5	0.00596605	2
V$HNF1_02	HNF-1alpha	HNF1A	0.0988846	2
V$HOXA9_01	hoxa9	HOXA9	0.195387	2
V$MSX1_01	Msx-1	MSX1	0.128457	2
V$SOX9_B1	SOX9	SOX9	0.140409	2

V$SP2_01	SP2	SP2	0.227525	1
V$ETV3_02	ETV3	ETV3	0.208496	1
V$AP2ALPHA_Q6	AP-2alpha	TFAP2A	0.285761	1
V$PET1_02	Pet-1	FEV	0.506882	1
V$ER71_02	ER71	ETV2	0.48211	1
V$EHF_03	EHF	EHF	0.00902817	1
V$ERG_03	ERG	ERG	0.108289	1
V$ERF_02	ERF	ERF	0.184299	1
V$ELK1_06	ELK-1	ELK1	0.142603	1
V$Elf5_03	ELF5	ELF5	0.170086	1
V$ESE1_02	ESE-1	ELF3	0.178331	1
V$SP1_Q6	Sp1	SP1	0.170955	1
V$MEF2A_Q6	mef2A	MEF2A	0.174419	1
V$HNF4A_Q6_01	HNF-4alpha	HNF4A	0.0999134	1
V$CEBPB_Q6	C/EBPbeta	CEBPB	0.0410464	1
V$WT1_Q6_01	WT1	WT1	0.0208458	1
V$GABPBETA_Q3	GABP-beta	GABPB1	0.19321	1
V$CEBPE_Q6	CEBPE	CEBPE	0.544254	1
V$MITF_Q6	MITF	MITF	0.198124	1
V$HOXB8_01	HOXB8	HOXB8	0.272232	1
V$PITX2_Q2	Pitx2	PITX2	0.131238	1
V$FOXO4_02	FOXO4	FOXO4	0.269493	1
V$AP2GAMMA_01	AP-2gamma	TFAP2C	0.26768	1
V$AP2ALPHA_01	AP-2alpha	TFAP2A	0.285761	1
V$ZIC3_01	Zic3	ZIC3	0.140012	1
V$E2F1_Q6	E2F-1	E2F1	0.0428963	1
V$E2F1_Q3	E2F-1	E2F1	0.0428963	1
V$GATA3_03	GATA-3	GATA3	0.225892	1
V$PAX3_B	Pax-3	PAX3	0.334385	1
V$ARNT_01	Arnt	ARNT	0.153176	1
V$CEBPB_01	C/EBPbeta	CEBPB	0.0410464	1
V$CREL_01	c-Rel	REL	0.171588	1
V$ELK1_02	Elk-1	ELK1	0.142603	1
V$AFP1_Q6	AFP1	ZFHX3	0.518302	1
V$CEBPD_Q6	C/EBPdelta	CEBPD	0.0145005	1
V$PITX2_01	PITX2	PITX2	0.131238	1
V$SP1_02	SP1	SP1	0.170955	1
V$ESE1_Q3	ESE-1	ELF3	0.178331	1
V$GFI1B_01	Gfi1b	GFI1B	0.0349115	1
V$YY1_Q6_02	YY1	YY1	0.120744	1
V$E2F1_Q6_01	E2F-1	E2F1	0.0428963	1
V$E2F1_Q3_01	E2F-1	E2F1	0.0428963	1
V$SP1_Q2_01	Sp1	SP1	0.170955	1
V$SP1_Q4_01	Sp1	SP1	0.170955	1
V$SP1_Q6_01	Sp1	SP1	0.170955	1
V$PIT1_Q6	Pit-1	POU1F1	0.174473	1

V$YY1_Q6	YY1	YY1	0.120744	1
V$AHR_Q5	AhR	AHR	0.279422	1
V$SP1_01	Sp1	SP1	0.170955	1

----

hsa-mir-28

Matrix_id	transcription factor	Gene	PCC	Occurrence
V$PEA3_Q6	PEA3	ETV4	0.0750412	32
V$AP2REP_01	AP-2rep	KLF12	0.0210687	32
V$PUR1_Q4	PUR1	PURA	0.448339	32
V$P300_01	p300	EP300	0.142309	31
V$CDX2_Q5_02	CDX-2	CDX2	0.0612535	30
V$GKLF_Q4	GKLF	KLF4	0.113233	30
V$IK_Q5	Ikaros	IKZF1	0.0248169	30
V$ELF1_Q6	Elf-1	ELF1	0.255297	29
V$CMYB_Q5	c-Myb	MYB	0.0516481	28
V$SOX9_B1	SOX9	SOX9	0.141987	28
V$MYB_Q6	c-Myb	MYB	0.0516481	28
V$SMAD4_Q6_01	Smad4	SMAD4	0.321432	28
V$NFAT4_Q3	NF-AT4	NFATC3	0.129362	28
V$SOX9_Q4	SOX9	SOX9	0.141987	27
V$YY1_01	YY1	YY1	0.334341	27
V$Elf5_03	ELF5	ELF5	0.0236042	26
V$NANOG_02	Nanog	NANOG	0.138445	26
V$IPF1_01	IPF1	PDX1	0.113036	26
V$CETS1_Q6	C-ets-1	ETS1	0.255039	26
V$ETS2_Q6	c-Ets-2	ETS2	0.131472	26
V$SMAD3_Q6_01	Smad3	SMAD3	0.0826761	25
V$SRY_02	SRY	SRY	0.0966829	24
V$BEN_01	BEN	GTF2IRD1	0.134535	23
V$TBX5_02	TBX5	TBX5	0.113131	23
V$CDX2_Q5_01	Cdx-2	CDX2	0.0612535	23
V$SPI1_Q5	SPI1	SPI1	0.0640412	23
V$ETS1_B	c-Ets-1	ETS1	0.255039	23
V$SPI1_03	SPI1	SPI1	0.0640412	22
V$PITX1_01	Pitx1	PITX1	0.185173	22
V$SP1_Q6	Sp1	SP1	0.200981	21
V$CMYB_01	c-Myb	MYB	0.0516481	21
V$AHR_Q5	AhR	AHR	0.189366	21
V$ZBP89_Q4	ZBP89	ZNF148	0.205279	20
V$SP1_Q6_01	Sp1	SP1	0.200981	20
V$YY1_Q6_03	YY1	YY1	0.334341	20
V$CMAF_01	c-Maf	MAF	0.176484	20

V$NFAT2_Q5	NF-AT2	NFATC1	0.179036	19
V$SP1_Q2_01	Sp1	SP1	0.200981	19
V$YY1_Q6_02	YY1	YY1	0.334341	19
V$SP1_Q4_01	Sp1	SP1	0.200981	19
V$ETS2_B	c-Ets-2	ETS2	0.131472	19
V$TBX5_01	TBX5	TBX5	0.113131	19
V$FKLF_Q5	FKLF	KLF11	0.0255797	18
V$TEF1_Q6	TEF-1	TEAD1	0.15455	18
V$SP1_01	Sp1	SP1	0.200981	17
V$ELK1_02	Elk-1	ELK1	0.0324435	17
V$GFI1_Q6_01	Gfi1	GFI1	0.162873	17
V$PBX1_Q3	Pbx1	PBX1	0.134876	17
V$TEL1_02	TEL1	ETV6	0.187537	17
V$PBX1_04	Pbx1	PBX1	0.134876	17
V$ETV3_02	ETV3	ETV3	0.0862932	17
V$LRF_Q2	LRF	ZBTB7A	0.21155	17
V$ER71_02	ER71	ETV2	0.311685	17
V$HIF1A_Q6	HIF-1alpha	HIF1A	0.0111761	16
V$TEF1_Q6_03	TEF-1	TEAD1	0.15455	16
V$IRF4_Q6	IRF-4	IRF4	0.154592	16
V$ARNT_01	Arnt	ARNT	0.103846	16
V$ELK1_06	ELK-1	ELK1	0.0324435	16
V$GFI1_Q6	Gfi1	GFI1	0.162873	16
V$CREM_Q6	CREM	CREM	0.060236	15
V$TFIII_Q6	TFII-I	GTF2I	0.141662	15
V$RFX1_02	RFX1	RFX1	0.112624	14
V$AP2ALPHA_Q6	AP-2alpha	TFAP2A	0.140734	14
V$ETV7_01	ETV7	ETV7	0.0808462	14
V$SP1_02	SP1	SP1	0.200981	14
V$ESE1_Q3	ESE-1	ELF3	0.159757	14
V$ELF5_01	ELF5	ELF5	0.0236042	14
V$IRF7_Q3	IRF-7	IRF7	0.372284	14
V$STAT3_03	STAT3	STAT3	0.0365066	13
V$SP2_01	SP2	SP2	0.159219	13
V$RFX1_01	RFX1	RFX1	0.112624	13
V$CEBPD_Q6	C/EBPdelta	CEBPD	0.0313301	12
V$EHF_03	EHF	EHF	0.104317	12
V$GFI1B_01	Gfi1b	GFI1B	0.125365	12
V$ATF1_Q6_01	ATF-1	ATF1	0.246657	12
V$VDR_Q3	VDR	VDR	0.105582	12
V$NEUROD_02	NeuroD	NEUROD1	0.0595728	11
V$ZABC1_01	ZABC1	ZNF217	0.177879	11
V$SPIB_03	Spi-B	SPIB	0.334165	11
V$GABPBETA_Q3	GABP-beta	GABPB1	0.349585	10
V$ELK1_01	Elk-1	ELK1	0.0324435	10
V$ESE1_02	ESE-1	ELF3	0.159757	10

V$KAISO_01	KAISO	ZBTB33	0.20721	9
V$AP2BETA_Q3	AP-2beta	TFAP2B	0.0599479	8
V$CNOT3_01	CNOT3	CNOT3	0.0363908	8
V$MEIS1_01	MEIS1	MEIS1	0.1528	8
V$ATF3_Q6_01	ATF-3	ATF3	0.0814585	8
V$ATF4_Q6	ATF-4	ATF4	0.0803986	8
V$RNF96_01	RNF96	TRIM28	0.114558	6
V$ATF6_01	ATF6	ATF6	0.094943	5
V$IRF7_01	IRF-7	IRF7	0.372284	5
V$CREL_01	c-Rel	REL	0.118196	5
V$PAX3_01	Pax-3	PAX3	0.160383	4
V$MAFK_Q3	MafK	MAFK	0.150132	4
V$SREBP1_01	SREBP-1	SREBF1	0.129415	3
V$NMYC_01	N-Myc	MYCN	0.0836934	3
V$IRF2_01	IRF-2	IRF2	0.0859612	3
V$MAX_01	Max	MAX	0.17369	3
V$ARNT_02	Arnt	ARNT	0.103846	3
V$ATF2_Q5	ATF-2	ATF2	0.159399	3
V$GLI3_Q5_01	GLI3	GLI3	0.139925	3
V$GLI2_01	GLI2	GLI2	0.0678922	2
V$GLI_Q2	GLI	GLI1	0.222745	2
V$SRF_03	SRF	SRF	0.00643292	1
V$GLI3_01	GLI3	GLI3	0.139925	1
V$GLI3_02	GLI3	GLI3	0.139925	1

----

hsa-mir-299

Matrix_id	transcription factor	Gene	PCC	Occurrence
V$PUR1_Q4	PUR1	PURA	0.15479	12
V$PARP_Q4	PARP	PARP1	0.202273	11
V$PEA3_Q6	PEA3	ETV4	0.0830607	10
V$YY1_Q6_03	YY1	YY1	0.360428	9
V$GATA6_01	GATA-6	GATA6	0.350198	9
V$GATA2_02	GATA-2	GATA2	0.359908	9
V$TBP_Q6	TBP	TBP	0.192257	8
V$YY1_01	YY1	YY1	0.360428	8
V$MEF2C_Q4	MEF-2C	MEF2C	0.14782	8
V$GATA3_01	GATA-3	GATA3	0.551874	8
V$GATA3_02	GATA-3	GATA3	0.551874	8
V$DLX5_01	dlx5	DLX5	0.633356	7
V$GATA2_01	GATA-2	GATA2	0.359908	7
V$PITX3_Q2	PITX3	PITX3	0.223394	7
V$DAX1_01	Dax1	NR0B1	0.220852	7

V$PBX1_04	Pbx1	PBX1	0.197135	6
V$PARP_Q3	PARP	PARP1	0.202273	6
V$SREBP1_Q6	SREBP-1	SREBF1	0.662267	6
V$ING4_01	ING4	ING4	0.147261	6
V$PBX1_Q3	Pbx1	PBX1	0.197135	6
V$PITX2_01	PITX2	PITX2	0.239914	6
V$GR_Q6	GR	NR3C1	0.0314611	6
V$FOXJ2_01	FOXJ2	FOXJ2	0.190163	5
V$MEF2A_Q6	mef2A	MEF2A	0.0243615	5
V$PITX2_Q2	Pitx2	PITX2	0.239914	5
V$STAT3_03	STAT3	STAT3	0.0666073	5
V$PIT1_Q6	Pit-1	POU1F1	0.043835	5
V$NURR1_Q3	NURR1	NR4A2	0.255788	4
V$GATA3_03	GATA-3	GATA3	0.551874	4
V$CIZ_01	CIZ	ZNF384	0.250201	3
V$GR_01	GR	NR3C1	0.0314611	3
V$HNF6_Q6	HNF6	ONECUT1	0.0871508	3
V$CEBPD_Q6	C/EBPdelta	CEBPD	0.194752	3
V$RORA2_01	RORalpha2	RORA	0.343536	2
V$EAR2_Q2	EAR2	NR2F6	0.285887	2
V$RORBETA_Q2	RORBETA	RORB	0.00365565	2
V$CEBPB_Q6	C/EBPbeta	CEBPB	0.367332	2
V$TCF4_01	TCF-4	TCF7L2	0.252918	2
V$CEBPB_02	C/EBPbeta	CEBPB	0.367332	2
V$MSX1_01	Msx-1	MSX1	0.0170235	2
V$FOXO4_01	FOXO4	FOXO4	0.675947	2
V$SOX10_Q6	SOX10	SOX10	0.0437846	2
V$RORA_Q4	RORalpha	RORA	0.343536	2
V$CART1_02	CART1	ALX1	0.0128454	2
V$HOX13_02	HOXA5	HOXA5	0.473272	2
V$CEBPB_01	C/EBPbeta	CEBPB	0.367332	2
V$CDP_04	CDP	CUX1	0.153101	1

hsa-mir-29a

Matrix_id	transcription factor	Gene	PCC	Occurrence
V$TTF1_Q5	TTF-1	NKX2-1	0.155148	4
V$PARP_Q3	PARP	PARP1	0.358367	4
V$IPF1_01	IPF1	PDX1	0.499714	4
V$DLX5_01	dlx5	DLX5	0.10159	4
V$PARP_Q4	PARP	PARP1	0.358367	4
V$IRF8_Q6	IRF-8	IRF8	0.219905	4
V$YY1_Q6_02	YY1	YY1	0.261918	4

V$HNF3B_Q6	HNF-3beta	FOXA2	0.165239	4
V$NANOG_02	Nanog	NANOG	0.585116	4
V$AP2REP_01	AP-2rep	KLF12	0.44988	4
V$CDX2_Q5_01	Cdx-2	CDX2	0.520227	4
V$ETS2_B	c-Ets-2	ETS2	0.462824	4
V$PUR1_Q4	PUR1	PURA	0.358976	4
V$YY1_Q6	YY1	YY1	0.261918	4
V$CDX2_Q5_02	CDX-2	CDX2	0.520227	4
V$SRY_02	SRY	SRY	0.357033	4
V$PEA3_Q6	PEA3	ETV4	0.511042	4
V$SOX5_01	SOX5	SOX5	0.0186502	4
V$GATA3_01	GATA-3	GATA3	0.0148384	4
V$GATA2_01	GATA-2	GATA2	0.326998	4
V$YY1_01	YY1	YY1	0.261918	4
V$GFI1_Q6	Gfi1	GFI1	0.00372793	3
V$TEF1_Q6_03	TEF-1	TEAD1	0.556891	3
V$HBP1_Q2	hbp1	HBP1	0.561559	3
V$PBX1_04	Pbx1	PBX1	0.118677	3
V$GABPA_Q4	GABP-alpha	GABPA	0.204318	3
V$HNF1_02	HNF-1alpha	HNF1A	0.27284	3
V$CDX2_01	Cdx-2	CDX2	0.520227	3
V$PITX3_Q2	PITX3	PITX3	0.47697	3
V$HOXD9_Q2	Hoxd9	HOXD9	0.301871	3
V$MEF2C_Q4	MEF-2C	MEF2C	0.263921	3
V$FOXJ2_01	FOXJ2	FOXJ2	0.388292	3
V$PBX1_Q3	Pbx1	PBX1	0.118677	3
V$GATA3_03	GATA-3	GATA3	0.0148384	3
V$MSX1_01	Msx-1	MSX1	0.525522	3
V$ZABC1_01	ZABC1	ZNF217	0.114042	3
V$BCL6_Q3_01	Bcl-6	BCL6	0.565838	3
V$SMAD4_Q6_01	Smad4	SMAD4	0.421155	3
V$SMAD3_Q6_01	Smad3	SMAD3	0.489464	3
V$CEBPB_02	C/EBPbeta	CEBPB	0.0553315	3
V$NFAT4_Q3	NF-AT4	NFATC3	0.164029	3
V$MYOGENIN_Q6	myogenin	MYOG	0.401179	3
V$IRF4_Q6	IRF-4	IRF4	0.554067	3
V$P300_01	p300	EP300	0.540185	3
V$TCF3_01	TCF-3	TCF7L1	0.255292	2
V$AP4_Q6_02	AP-4	TFAP4	0.427192	2
V$MATH1_Q2	MATH1	ATOH1	0.2921	2
V$GFI1_Q6_01	Gfi1	GFI1	0.00372793	2
V$MYOGENIN_Q6_01	myogenin	MYOG	0.401179	2
V$IPF1_Q4	IPF1	PDX1	0.499714	2
V$AMEF2_Q6	aMEF-2	MEF2A	0.173239	2
V$IPF1_02	IPF1	PDX1	0.499714	2
V$CMAF_01	c-Maf	MAF	0.399208	2

V$FOXJ2_02	FOXJ2	FOXJ2	0.388292	2
V$IPF1_Q4_01	IPF1	PDX1	0.499714	2
V$PIT1_Q6	Pit-1	POU1F1	0.0812911	2
V$SMAD3_Q6	SMAD3	SMAD3	0.489464	2
V$CEBPD_Q6	C/EBPdelta	CEBPD	0.0171301	2
V$IPF1_Q6	IPF1	PDX1	0.499714	2
V$FAC1_01	FAC1	BPTF	0.344513	2
V$NKX3A_02	Nkx3A	NKX3-1	0.0805136	2
V$AP4_Q5	AP-4	TFAP4	0.427192	2
V$AP4_Q6	AP-4	TFAP4	0.427192	2
V$CDX1_01	Cdx-1	CDX1	0.150548	2
V$GATA2_02	GATA-2	GATA2	0.326998	2
V$GATA3_02	GATA-3	GATA3	0.0148384	2
V$HOXA9_01	hoxa9	HOXA9	0.542455	2
V$MEF2A_Q6	mef2A	MEF2A	0.173239	1
V$CDP_02	CDP	CUX1	0.0701507	1
V$IPF1_06	ipf1	PDX1	0.499714	1
V$HLF_01	HLF	HLF	0.0750673	1
V$BACH1_01	Bach1	BACH1	0.338456	1
V$HOX13_02	HOXA5	HOXA5	0.161241	1
V$POU6F1_02	POU6F1	POU6F1	0.37916	1
V$POU6F1_03	POU6F1	POU6F1	0.37916	1
V$OCT2_01	2-Oct	POU2F2	0.376439	1
V$CART1_02	CART1	ALX1	0.523123	1
V$IPF1_03	IPF1	PDX1	0.499714	1
V$GFI1B_01	Gfi1b	GFI1B	0.345115	1
V$IRF1_Q6_01	IRF-1	IRF1	0.324401	1
V$OC2_Q3	OC-2	ONECUT2	0.35004	1

----

hsa-mir-29b-1

Matrix_id	transcription factor	Gene	PCC	Occurrence
V$PUR1_Q4	PUR1	PURA	0.530335	172
V$PEA3_Q6	PEA3	ETV4	0.711858	170
V$PARP_Q4	PARP	PARP1	0.618848	168
V$AP2REP_01	AP-2rep	KLF12	0.619092	167
V$P300_01	p300	EP300	0.758039	164
V$NFAT4_Q3	NF-AT4	NFATC3	0.319925	151
V$CETS1_Q6	C-ets-1	ETS1	0.110153	149
V$ETS1_B	c-Ets-1	ETS1	0.110153	147
V$GATA1_01	GATA-1	GATA1	0.120139	145
V$GABPA_Q4	GABP-alpha	GABPA	0.331341	143
V$SMAD4_Q6_01	Smad4	SMAD4	0.615185	143

V$CDX2_Q5_02	CDX-2	CDX2	0.743958	141
V$TBP_Q6	TBP	TBP	0.268457	141
V$SOX9_Q4	SOX9	SOX9	0.0416165	138
V$TTF1_Q5	TTF-1	NKX2-1	0.0550804	135
V$SMAD3_Q6_01	Smad3	SMAD3	0.728804	134
V$MEF2C_Q4	MEF-2C	MEF2C	0.43422	127
V$SPI1_Q5	SPI1	SPI1	0.0875851	127
V$DLX5_01	dlx5	DLX5	0.435447	126
V$YY1_Q6	YY1	YY1	0.351718	126
V$AP4_Q6_02	AP-4	TFAP4	0.666064	125
V$YY1_Q6_02	YY1	YY1	0.351718	125
V$ETS2_B	c-Ets-2	ETS2	0.767429	124
V$YY1_01	YY1	YY1	0.351718	124
V$NANOG_02	Nanog	NANOG	0.787557	123
V$SOX9_B1	SOX9	SOX9	0.0416165	123
V$SOX5_01	SOX5	SOX5	0.41039	123
V$CDX2_Q5_01	Cdx-2	CDX2	0.743958	118
V$PARP_Q3	PARP	PARP1	0.618848	118
V$GATA2_02	GATA-2	GATA2	0.546534	116
V$GATA1_06	GATA-1	GATA1	0.120139	116
V$GATA1_05	GATA-1	GATA1	0.120139	116
V$GATA1_02	GATA-1	GATA1	0.120139	116
V$IPF1_01	IPF1	PDX1	0.723155	114
V$SRY_02	SRY	SRY	0.646544	112
V$IPF1_Q6	IPF1	PDX1	0.723155	111
V$HNF3B_Q6	HNF-3beta	FOXA2	0.450927	109
V$CDX2_01	Cdx-2	CDX2	0.743958	109
V$IPF1_Q4_01	IPF1	PDX1	0.723155	108
V$PITX3_Q2	PITX3	PITX3	0.607269	104
V$GATA3_02	GATA-3	GATA3	0.142481	103
V$GATA1_04	GATA-1	GATA1	0.120139	100
V$HNF1_02	HNF-1alpha	HNF1A	0.520495	96
V$IRF4_Q6	IRF-4	IRF4	0.759376	95
V$GATA3_01	GATA-3	GATA3	0.142481	94
V$PBX1_04	Pbx1	PBX1	0.0617585	92
V$IPF1_Q4	IPF1	PDX1	0.723155	91
V$MYOGENIN_Q6	myogenin	MYOG	0.730133	88
V$AP4_Q5	AP-4	TFAP4	0.666064	87
V$TEF1_Q6_03	TEF-1	TEAD1	0.760236	86
V$GFI1_Q6	Gfi1	GFI1	0.11204	85
V$CEBPE_Q6	CEBPE	CEBPE	0.0254242	84
V$FOXJ2_01	FOXJ2	FOXJ2	0.682982	84
V$PBX1_Q3	Pbx1	PBX1	0.0617585	82
V$CMAF_01	c-Maf	MAF	0.5767	82
V$GATA2_01	GATA-2	GATA2	0.546534	80
V$GFI1_Q6_01	Gfi1	GFI1	0.11204	79

V$FOXM1_01	FOXM1	FOXM1	0.0205374	79
V$TCF3_01	TCF-3	TCF7L1	0.555626	77
V$IRF8_Q6	IRF-8	IRF8	0.0741368	74
V$AP4_Q6	AP-4	TFAP4	0.666064	71
V$TCF4_Q5	TCF-4	TCF7L2	0.0901249	70
V$BCL6_Q3_01	Bcl-6	BCL6	0.750833	69
V$ELF5_01	ELF5	ELF5	0.173356	69
V$MYOGENIN_Q6_01	myogenin	MYOG	0.730133	67
V$FAC1_01	FAC1	BPTF	0.216539	67
V$CEBPD_Q6	C/EBPdelta	CEBPD	0.328507	67
V$GFI1B_01	Gfi1b	GFI1B	0.528938	66
V$PIT1_Q6	Pit-1	POU1F1	0.262906	65
V$OC2_Q3	OC-2	ONECUT2	0.510592	65
V$CEBPB_02	C/EBPbeta	CEBPB	0.322783	64
V$GATA3_03	GATA-3	GATA3	0.142481	62
V$ZABC1_01	ZABC1	ZNF217	0.122903	61
V$HOXA9_01	hoxa9	HOXA9	0.756695	60
V$SMAD3_Q6	SMAD3	SMAD3	0.728804	59
V$CDX1_01	Cdx-1	CDX1	0.431879	59
V$HBP1_Q2	hbp1	HBP1	0.592445	57
V$HOXD9_Q2	Hoxd9	HOXD9	0.618871	57
V$CEBPG_Q6_01	C/EBPgamma	CEBPG	0.17976	56
V$MSX1_01	Msx-1	MSX1	0.64799	51
V$MATH1_Q2	MATH1	ATOH1	0.576819	49
V$IPF1_02	IPF1	PDX1	0.723155	48
V$CART1_02	CART1	ALX1	0.754041	46
V$HOX13_02	HOXA5	HOXA5	0.195525	46
V$HNF6_Q6	HNF6	ONECUT1	0.0978169	45
V$MEF2A_Q6	mef2A	MEF2A	0.147381	45
V$IPF1_06	ipf1	PDX1	0.723155	42
V$IPF1_03	IPF1	PDX1	0.723155	41
V$IRF1_Q6_01	IRF-1	IRF1	0.530794	31
V$CEBPG_Q6	C/EBPgamma	CEBPG	0.17976	26
V$POU6F1_03	POU6F1	POU6F1	0.576554	26
V$POU6F1_02	POU6F1	POU6F1	0.576554	21
V$FOXJ2_02	FOXJ2	FOXJ2	0.682982	20
V$OCT2_01	2-Oct	POU2F2	0.682205	19
V$AMEF2_Q6	aMEF-2	MEF2A	0.147381	15
V$CDP_02	CDP	CUX1	0.337132	10
V$BACH1_01	Bach1	BACH1	0.564701	10
V$NKX3A_02	Nkx3A	NKX3-1	0.0829537	7
V$HLF_01	HLF	HLF	0.201804	3

----

hsa-mir-29b-2

Matrix_id	transcription factor	Gene	PCC	Occurrence
V$PUR1_Q4	PUR1	PURA	0.530335	172
V$PEA3_Q6	PEA3	ETV4	0.711858	170
V$PARP_Q4	PARP	PARP1	0.618848	168
V$AP2REP_01	AP-2rep	KLF12	0.619092	167
V$IK_Q5	Ikaros	IKZF1	0.560181	166
V$P300_01	p300	EP300	0.758039	164
V$GKLF_Q4	GKLF	KLF4	0.0950702	162
V$NFAT4_Q3	NF-AT4	NFATC3	0.319925	151
V$ZIC3_01	Zic3	ZIC3	0.459784	149
V$CETS1_Q6	C-ets-1	ETS1	0.110153	149
V$ETS1_B	c-Ets-1	ETS1	0.110153	147
V$GATA1_01	GATA-1	GATA1	0.120139	145
V$GABPA_Q4	GABP-alpha	GABPA	0.331341	143
V$SMAD4_Q6_01	Smad4	SMAD4	0.615185	143
V$Elf5_03	ELF5	ELF5	0.173356	141
V$ETS2_Q6	c-Ets-2	ETS2	0.767429	139
V$NR1B2_Q6	NR1B2	RARB	0.651012	139
V$SOX9_Q4	SOX9	SOX9	0.0416165	138
V$GR_Q6	GR	NR3C1	0.657552	137
V$TTF1_Q5	TTF-1	NKX2-1	0.0550804	135
V$SMAD3_Q6_01	Smad3	SMAD3	0.728804	134
V$SPI1_03	SPI1	SPI1	0.0875851	130
V$BEN_01	BEN	GTF2IRD1	0.764344	128
V$SPI1_Q5	SPI1	SPI1	0.0875851	127
V$YY1_Q6	YY1	YY1	0.351718	126
V$AML1_Q6	AML1	RUNX1	0.528544	125
V$YY1_Q6_02	YY1	YY1	0.351718	125
V$AP4_Q6_02	AP-4	TFAP4	0.666064	125
V$SOX9_B1	SOX9	SOX9	0.0416165	123
V$SOX5_01	SOX5	SOX5	0.41039	123
V$PARP_Q3	PARP	PARP1	0.618848	118
V$GATA1_02	GATA-1	GATA1	0.120139	116
V$GATA1_05	GATA-1	GATA1	0.120139	116
V$GATA2_02	GATA-2	GATA2	0.546534	116
V$GATA1_06	GATA-1	GATA1	0.120139	116
V$HNF4A_Q6_01	HNF-4alpha	HNF4A	0.343397	114
V$SRY_02	SRY	SRY	0.646544	112
V$LRF_Q2	LRF	ZBTB7A	0.602486	111
V$IPF1_Q6	IPF1	PDX1	0.723155	111
V$HNF3B_Q6	HNF-3beta	FOXA2	0.450927	109
V$IPF1_Q4_01	IPF1	PDX1	0.723155	108
V$MAZ_Q6	MAZ	MAZ	0.0613328	108
V$HNF3A_01	HNF3A	FOXA1	0.367146	105

V$ERBETA_Q5	ER-beta	ESR2	0.613175	103
V$GATA3_02	GATA-3	GATA3	0.142481	103
V$NFAT2_Q5	NF-AT2	NFATC1	0.642484	101
V$E2A_Q6	E2A	TCF3	0.581089	101
V$GATA1_04	GATA-1	GATA1	0.120139	100
V$E47_02	E47	TCF3	0.581089	100
V$E12_Q6	E12	TCF3	0.581089	100
V$YY1_Q6_03	YY1	YY1	0.351718	99
V$MYOD_Q6_01	MyoD	MYOD1	0.642041	98
V$FOXO3A_Q1	FOXO3A	FOXO3	0.788477	96
V$HNF1_02	HNF-1alpha	HNF1A	0.520495	96
V$IRF4_Q6	IRF-4	IRF4	0.759376	95
V$AP2ALPHA_Q6	AP-2alpha	TFAP2A	0.310934	95
V$GATA3_01	GATA-3	GATA3	0.142481	94
V$AP2ALPHA_01	AP-2alpha	TFAP2A	0.310934	94
V$ZBP89_Q4	ZBP89	ZNF148	0.649159	89
V$AML2_Q3	AML2	RUNX3	0.165156	89
V$AP4_Q5	AP-4	TFAP4	0.666064	87
V$TEF1_Q6_03	TEF-1	TEAD1	0.760236	86
V$GR_01	GR	NR3C1	0.657552	86
V$GFI1_Q6	Gfi1	GFI1	0.11204	85
V$CEBPE_Q6	CEBPE	CEBPE	0.0254242	84
V$TFIII_Q6	TFII-I	GTF2I	0.619884	83
V$FKLF_Q5	FKLF	KLF11	0.0891492	83
V$FOXP3_01	FOXP3	FOXP3	0.740134	82
V$CMAF_01	c-Maf	MAF	0.5767	82
V$TEF1_Q6	TEF-1	TEAD1	0.760236	81
V$GATA2_01	GATA-2	GATA2	0.546534	80
V$MAZ_Q6_01	MAZ	MAZ	0.0613328	79
V$GFI1_Q6_01	Gfi1	GFI1	0.11204	79
V$FOXM1_01	FOXM1	FOXM1	0.0205374	79
V$TCF3_01	TCF-3	TCF7L1	0.555626	77
V$ZFX_01	Zfx	ZFX	0.743259	76
V$RFX1_02	RFX1	RFX1	0.795275	75
V$IRF8_Q6	IRF-8	IRF8	0.0741368	74
V$KLF15_Q2	KLF15	KLF15	0.108618	74
V$AML1_01	AML1a	RUNX1	0.528544	73
V$NEUROD_02	NeuroD	NEUROD1	0.70818	73
V$AP4_Q6	AP-4	TFAP4	0.666064	71
V$TCF4_Q5	TCF-4	TCF7L2	0.0901249	70
V$EGR1_02	EGR-1	EGR1	0.286737	70
V$ELF5_01	ELF5	ELF5	0.173356	69
V$WT1_Q6	WT1	WT1	0.207727	67
V$MYOGENIN_Q6_01	myogenin	MYOG	0.730133	67
V$ERR1_Q3	ERR1	ESRRA	0.54526	66
V$GFI1B_01	Gfi1b	GFI1B	0.528938	66

V$AML1_Q4	AML1	RUNX1	0.528544	64
V$AP2BETA_Q3	AP-2beta	TFAP2B	0.802422	63
V$AP2GAMMA_01	AP-2gamma	TFAP2C	0.441301	62
V$ZABC1_01	ZABC1	ZNF217	0.122903	61
V$GATA2_03	GATA-2	GATA2	0.546534	59
V$YY1_02	YY1	YY1	0.351718	58
V$CEBPG_Q6_01	C/EBPgamma	CEBPG	0.17976	56
V$MATH1_Q2	MATH1	ATOH1	0.576819	49
V$ZIC1_01	Zic1	ZIC1	0.190298	48
V$NURR1_Q3	NURR1	NR4A2	0.222409	45
V$MEIS1_01	MEIS1	MEIS1	0.166181	42
V$AP4_01	AP-4	TFAP4	0.666064	35
V$RORBETA_Q2	RORBETA	RORB	0.669982	30
V$STAF_02	Staf	ZNF143	0.738901	29
V$CEBPG_Q6	C/EBPgamma	CEBPG	0.17976	26
V$GLI_Q2	GLI	GLI1	0.0455023	18
V$GLI2_01	GLI2	GLI2	0.770884	13
V$GLI3_01	GLI3	GLI3	0.765935	11
V$GLI3_02	GLI3	GLI3	0.765935	11
V$MEIS1_02	Meis1	MEIS1	0.166181	4

hsa-mir-29c

Matrix_id	transcription factor	Gene	PCC	Occurrence
V$SRY_02	SRY	SRY	0.215772	5
V$PUR1_Q4	PUR1	PURA	0.276416	5
V$SOX9_B1	SOX9	SOX9	0.270977	5
V$IRF4_Q6	IRF-4	IRF4	0.291293	5
V$ZIC3_01	Zic3	ZIC3	0.251676	5
V$GR_01	GR	NR3C1	0.321556	5
V$BEN_01	BEN	GTF2IRD1	0.299695	5
V$GKLF_Q4	GKLF	KLF4	0.0326416	5
V$YY1_Q6	YY1	YY1	0.134519	5
V$RFX1_02	RFX1	RFX1	0.279609	5
V$IK_Q5	Ikaros	IKZF1	0.142149	5
V$GR_Q6	GR	NR3C1	0.321556	5
V$P300_01	p300	EP300	0.273868	5
V$TTF1_Q5	TTF-1	NKX2-1	0.238232	5
V$SOX9_Q4	SOX9	SOX9	0.270977	5
V$PARP_Q4	PARP	PARP1	0.0647563	5
V$HNF4A_Q6_01	HNF-4alpha	HNF4A	0.0426996	5
V$FOXO3A_Q1	FOXO3A	FOXO3	0.202483	4
V$NR1B2_Q6	NR1B2	RARB	0.190087	4

V$YY1_Q6_02	YY1	YY1	0.134519	4
V$PARP_Q3	PARP	PARP1	0.0647563	4
V$ETS2_Q6	c-Ets-2	ETS2	0.29874	4
V$GFI1B_01	Gfi1b	GFI1B	0.0965739	4
V$AML1_Q6	AML1	RUNX1	0.1439	4
V$ELF1_Q6	Elf-1	ELF1	0.199867	4
V$GABPA_Q4	GABP-alpha	GABPA	0.126027	4
V$YY1_Q6_03	YY1	YY1	0.134519	4
V$GATA2_02	GATA-2	GATA2	0.0333829	4
V$AP2REP_01	AP-2rep	KLF12	0.169329	4
V$PEA3_Q6	PEA3	ETV4	0.284163	4
V$SMAD3_Q6_01	Smad3	SMAD3	0.375557	4
V$SMAD4_Q6_01	Smad4	SMAD4	0.236302	4
V$AML1_Q4	AML1	RUNX1	0.1439	3
V$FOXP3_01	FOXP3	FOXP3	0.190528	3
V$ZBP89_Q4	ZBP89	ZNF148	0.385165	3
V$TCF3_01	TCF-3	TCF7L1	0.232017	3
V$HNF3B_Q6	HNF-3beta	FOXA2	0.0618684	3
V$KLF15_Q2	KLF15	KLF15	0.13574	3
V$Elf5_03	ELF5	ELF5	0.456788	3
V$NFAT2_Q5	NF-AT2	NFATC1	0.205611	3
V$AML1_01	AML1a	RUNX1	0.1439	3
V$AP2BETA_Q3	AP-2beta	TFAP2B	0.241229	3
V$ERBETA_Q5	ER-beta	ESR2	0.0620713	3
V$AML2_Q3	AML2	RUNX3	0.115472	3
V$TEF1_Q6_03	TEF-1	TEAD1	0.238361	3
V$HNF1_02	HNF-1alpha	HNF1A	0.162336	3
V$LRF_Q2	LRF	ZBTB7A	0.132967	3
V$GATA2_03	GATA-2	GATA2	0.0333829	3
V$E12_Q6	E12	TCF3	0.151716	3
V$IPF1_Q4_01	IPF1	PDX1	0.209686	3
V$HNF3A_01	HNF3A	FOXA1	0.177868	3
V$E2A_Q6	E2A	TCF3	0.151716	3
V$E47_02	E47	TCF3	0.151716	3
V$IPF1_Q6	IPF1	PDX1	0.209686	3
V$MYOD_Q6_01	MyoD	MYOD1	0.205284	3
V$TCF4_Q5	TCF-4	TCF7L2	0.107403	3
V$NEUROD_02	NeuroD	NEUROD1	0.260504	2
V$ERR1_Q3	ERR1	ESRRA	0.138435	2
V$AP2GAMMA_01	AP-2gamma	TFAP2C	0.026114	2
V$EGR1_02	EGR-1	EGR1	0.594856	2
V$GATA2_01	GATA-2	GATA2	0.0333829	2
V$YY1_02	YY1	YY1	0.134519	2
V$TEF1_Q6	TEF-1	TEAD1	0.238361	2
V$TFIII_Q6	TFII-I	GTF2I	0.19271	2
V$ZFX_01	Zfx	ZFX	0.335417	2

V$NURR1_Q3	NURR1	NR4A2	0.359588	2
V$GLI2_01	GLI2	GLI2	0.242634	2
V$RORBETA_Q2	RORBETA	RORB	0.121526	2
V$GLI3_02	GLI3	GLI3	0.172244	2
V$ELF5_01	ELF5	ELF5	0.456788	2
V$CMAF_01	c-Maf	MAF	0.229772	2
V$MEIS1_01	MEIS1	MEIS1	0.14455	1
V$MYOGENIN_Q6_01	myogenin	MYOG	0.281392	1
V$MEIS1_02	Meis1	MEIS1	0.14455	1
V$GLI3_01	GLI3	GLI3	0.172244	1
V$MATH1_Q2	MATH1	ATOH1	0.144391	1
V$AP4_Q5	AP-4	TFAP4	0.511992	1
V$AP4_01	AP-4	TFAP4	0.511992	1
V$AP4_Q6_02	AP-4	TFAP4	0.511992	1
V$STAF_02	Staf	ZNF143	0.270257	1
V$AP4_Q6	AP-4	TFAP4	0.511992	1

---

hsa-mir-30a

Matrix_id	transcription factor	Gene	PCC	Occurrence
V$PUR1_Q4	PUR1	PURA	0.205624	59
V$GKLF_Q4	GKLF	KLF4	0.402135	59
V$PEA3_Q6	PEA3	ETV4	0.171281	59
V$PARP_Q4	PARP	PARP1	0.136019	59
V$ELF1_Q6	Elf-1	ELF1	0.0946024	58
V$ETS1_B	c-Ets-1	ETS1	0.245229	56
V$CETS1_Q6	C-ets-1	ETS1	0.245229	55
V$ZIC3_01	Zic3	ZIC3	0.296934	54
V$P300_01	p300	EP300	0.428844	54
V$SMAD4_Q6_01	Smad4	SMAD4	0.325055	53
V$GABPA_Q4	GABP-alpha	GABPA	0.401319	52
V$GATA1_01	GATA-1	GATA1	0.158639	51
V$ETS2_Q6	c-Ets-2	ETS2	0.145051	50
V$SPI1_Q5	SPI1	SPI1	0.0408765	49
V$Elf5_03	ELF5	ELF5	0.0327528	48
V$SPI1_03	SPI1	SPI1	0.0408765	47
V$IPF1_Q6	IPF1	PDX1	0.147068	46
V$GR_Q6	GR	NR3C1	0.204562	46
V$YY1_01	YY1	YY1	0.23408	46
V$NR1B2_Q6	NR1B2	RARB	0.23528	46
V$IPF1_Q4_01	IPF1	PDX1	0.147068	45
V$TBP_Q6	TBP	TBP	0.104342	45
V$CDX2_Q5_02	CDX-2	CDX2	0.114135	45

V$AML1_Q6	AML1	RUNX1	0.188501	44
V$HNF4A_Q6_01	HNF-4alpha	HNF4A	0.177764	44
V$GATA1_06	GATA-1	GATA1	0.158639	43
V$GATA2_02	GATA-2	GATA2	0.634761	43
V$NANOG_02	Nanog	NANOG	0.09471	43
V$GATA1_05	GATA-1	GATA1	0.158639	43
V$SMAD3_Q6_01	Smad3	SMAD3	0.125001	42
V$SOX5_01	SOX5	SOX5	0.059781	41
V$GATA1_02	GATA-1	GATA1	0.158639	40
V$LRF_Q2	LRF	ZBTB7A	0.244722	40
V$DLX5_01	dlx5	DLX5	0.550939	40
V$IRF4_Q6	IRF-4	IRF4	0.188197	39
V$AP4_Q6_02	AP-4	TFAP4	0.12997	39
V$GATA3_02	GATA-3	GATA3	0.743018	38
V$YY1_Q6_02	YY1	YY1	0.23408	38
V$YY1_Q6	YY1	YY1	0.23408	37
V$CDX2_Q5_01	Cdx-2	CDX2	0.114135	37
V$IPF1_Q4	IPF1	PDX1	0.147068	37
V$FOXO3A_Q1	FOXO3A	FOXO3	0.07902	36
V$MAZ_Q6_01	MAZ	MAZ	0.132808	36
V$PARP_Q3	PARP	PARP1	0.136019	35
V$NFAT2_Q5	NF-AT2	NFATC1	0.0541603	35
V$MAZ_Q6	MAZ	MAZ	0.132808	35
V$CDX2_01	Cdx-2	CDX2	0.114135	35
V$TFIII_Q6	TFII-I	GTF2I	0.373131	35
V$ING4_01	ING4	ING4	0.0387009	34
V$FKLF_Q5	FKLF	KLF11	0.260686	33
V$YY1_Q6_03	YY1	YY1	0.23408	33
V$HMGIY_01	HMGIY	HMGA1	0.175516	33
V$GATA1_04	GATA-1	GATA1	0.158639	33
V$GATA3_01	GATA-3	GATA3	0.743018	33
V$PITX3_Q2	PITX3	PITX3	0.0713503	32
V$GATA2_01	GATA-2	GATA2	0.634761	30
V$CEBPA_Q6	C/EBPalpha	CEBPA	0.00798439	30
V$ELK1_02	Elk-1	ELK1	0.222954	30
V$TEL1_02	TEL1	ETV6	0.129457	30
V$AML1_01	AML1a	RUNX1	0.188501	29
V$ETV3_02	ETV3	ETV3	0.00225999	28
V$PITX2_Q2	Pitx2	PITX2	0.48608	28
V$AML1_Q4	AML1	RUNX1	0.188501	28
V$BCL6_Q3_01	Bcl-6	BCL6	0.0350918	28
V$CEBPE_Q6	CEBPE	CEBPE	0.335988	28
V$CRX_Q4	Crx	CRX	0.190496	28
V$PITX1_01	Pitx1	PITX1	0.0610827	27
V$CRX_02	Crx	CRX	0.190496	27
V$PITX2_01	PITX2	PITX2	0.48608	26

V$SMAD3_Q6	SMAD3	SMAD3	0.125001	26
V$ELK1_06	ELK-1	ELK1	0.222954	26
V$ETV7_01	ETV7	ETV7	0.206445	26
V$ER71_02	ER71	ETV2	0.245723	26
V$CP2_01	CP2	TFCP2	0.596106	25
V$TEF1_Q6_03	TEF-1	TEAD1	0.16238	25
V$CRX_Q4_01	CRX	CRX	0.190496	25
V$HNF1_02	HNF-1alpha	HNF1A	0.194813	25
V$ESE1_Q3	ESE-1	ELF3	0.366871	24
V$TEF1_Q6	TEF-1	TEAD1	0.16238	23
V$ZABC1_01	ZABC1	ZNF217	0.555287	23
V$RFX1_02	RFX1	RFX1	0.134682	23
V$TCF3_01	TCF-3	TCF7L1	0.146726	23
V$CEBPB_02	C/EBPbeta	CEBPB	0.511608	23
V$ERF_02	ERF	ERF	0.0121565	21
V$YY1_02	YY1	YY1	0.23408	21
V$GATA3_03	GATA-3	GATA3	0.743018	21
V$PIT1_Q6	Pit-1	POU1F1	0.154687	20
V$OC2_Q3	OC-2	ONECUT2	0.155027	20
V$CEBPD_Q6	C/EBPdelta	CEBPD	0.408926	19
V$GATA2_03	GATA-2	GATA2	0.634761	19
V$RFX1_01	RFX1	RFX1	0.134682	19
V$HBP1_Q2	hbp1	HBP1	0.517935	18
V$HOXD9_Q2	Hoxd9	HOXD9	0.0786879	18
V$CEBPB_Q6	C/EBPbeta	CEBPB	0.511608	17
V$ESE1_02	ESE-1	ELF3	0.366871	17
V$AP4_01	AP-4	TFAP4	0.12997	16
V$IRF1_Q6_01	IRF-1	IRF1	0.460819	16
V$IPF1_02	IPF1	PDX1	0.147068	15
V$CDX1_01	Cdx-1	CDX1	0.0712971	15
V$CEBPA_01	C/EBPalpha	CEBPA	0.00798439	12
V$YY1_03	YY1	YY1	0.23408	12
V$CDP_04	CDP	CUX1	0.366261	11
V$FOXJ2_02	FOXJ2	FOXJ2	0.148898	10
V$CEBPB_01	C/EBPbeta	CEBPB	0.511608	9
V$OCT2_01	2-Oct	POU2F2	0.273116	6
V$HNF1B_01	HNF-1beta	HNF1B	0.369541	5
V$IRF1_01	IRF-1	IRF1	0.460819	2

----

hsa-mir-30b

Matrix_id	transcription factor	Gene	PCC	Occurrence
V$PUR1_Q4	PUR1	PURA	0.153821	146

V$PEA3_Q6	PEA3	ETV4	0.177397	144
V$GKLF_Q4	GKLF	KLF4	0.481599	142
V$PARP_Q4	PARP	PARP1	0.114808	142
V$ETS1_B	c-Ets-1	ETS1	0.350111	138
V$AP2REP_01	AP-2rep	KLF12	0.107431	136
V$P300_01	p300	EP300	0.407476	135
V$ZIC3_01	Zic3	ZIC3	0.252914	134
V$CETS1_Q6	C-ets-1	ETS1	0.350111	130
V$SMAD4_Q6_01	Smad4	SMAD4	0.38271	129
V$GATA1_01	GATA-1	GATA1	0.346727	126
V$Elf5_03	ELF5	ELF5	0.0203193	125
V$ETS2_Q6	c-Ets-2	ETS2	0.102137	124
V$GABPA_Q4	GABP-alpha	GABPA	0.314906	123
V$ETS2_B	c-Ets-2	ETS2	0.102137	118
V$NR1B2_Q6	NR1B2	RARB	0.253828	118
V$HNF4A_Q6_01	HNF-4alpha	HNF4A	0.139762	118
V$TBX5_02	TBX5	TBX5	0.345732	117
V$SPI1_Q5	SPI1	SPI1	0.0637004	115
V$SMAD3_Q6_01	Smad3	SMAD3	0.0559922	112
V$NANOG_02	Nanog	NANOG	0.0649465	111
V$AML1_Q6	AML1	RUNX1	0.293545	111
V$SPI1_03	SPI1	SPI1	0.0637004	109
V$YY1_01	YY1	YY1	0.20719	109
V$AP4_Q6_02	AP-4	TFAP4	0.0633867	104
V$MAZ_Q6	MAZ	MAZ	0.14265	103
V$LRF_Q2	LRF	ZBTB7A	0.294432	101
V$YY1_Q6_02	YY1	YY1	0.20719	100
V$NKX32_01	Nkx3-2	NKX3-2	0.0211098	97
V$YY1_Q6	YY1	YY1	0.20719	97
V$GATA1_02	GATA-1	GATA1	0.346727	97
V$ING4_01	ING4	ING4	0.0715095	94
V$ERBETA_Q5	ER-beta	ESR2	0.152843	93
V$E2A_Q6	E2A	TCF3	0.132085	92
V$E12_Q6	E12	TCF3	0.132085	92
V$MYOD_Q6_01	MyoD	MYOD1	0.0227158	91
V$MYOGENIN_Q6	myogenin	MYOG	0.122277	91
V$E47_02	E47	TCF3	0.132085	91
V$SP1_Q6	Sp1	SP1	0.530806	87
V$GATA2_02	GATA-2	GATA2	0.652842	85
V$AP2ALPHA_Q6	AP-2alpha	TFAP2A	0.853192	85
V$GATA1_05	GATA-1	GATA1	0.346727	85
V$GATA1_06	GATA-1	GATA1	0.346727	85
V$GATA6_01	GATA-6	GATA6	0.135435	85
V$GATA3_01	GATA-3	GATA3	0.738914	84
V$E2A_Q2	E2A	TCF3	0.132085	81
V$TFIII_Q6	TFII-I	GTF2I	0.396029	79

V$AP2ALPHA_01	AP-2alpha	TFAP2A	0.853192	79
V$SP1_Q6_01	Sp1	SP1	0.530806	79
V$GATA2_01	GATA-2	GATA2	0.652842	79
V$GATA1_04	GATA-1	GATA1	0.346727	78
V$NFAT2_Q5	NF-AT2	NFATC1	0.111695	77
V$SP1_Q4_01	Sp1	SP1	0.530806	75
V$CP2_01	CP2	TFCP2	0.535145	74
V$AP4_Q5	AP-4	TFAP4	0.0633867	74
V$FOXO3A_Q1	FOXO3A	FOXO3	0.00844199	74
V$SP1_Q2_01	Sp1	SP1	0.530806	74
V$PITX3_Q2	PITX3	PITX3	0.0219432	73
V$AML2_Q3	AML2	RUNX3	0.0481636	73
V$TCF3_01	TCF-3	TCF7L1	0.112805	71
V$GATA3_02	GATA-3	GATA3	0.738914	70
V$TEF1_Q6_03	TEF-1	TEAD1	0.162753	69
V$ARNT_01	Arnt	ARNT	0.549961	66
V$TEF1_Q6	TEF-1	TEAD1	0.162753	66
V$AP4_Q6	AP-4	TFAP4	0.0633867	66
V$MYOGENIN_Q6_01	myogenin	MYOG	0.122277	64
V$CRX_Q4	Crx	CRX	0.164671	62
V$ELF5_01	ELF5	ELF5	0.0203193	61
V$SMAD3_Q6	SMAD3	SMAD3	0.0559922	60
V$CEBPE_Q6	CEBPE	CEBPE	0.502775	59
V$DAX1_01	Dax1	NR0B1	0.0669372	58
V$AP2GAMMA_01	AP-2gamma	TFAP2C	0.736461	55
V$PITX1_01	Pitx1	PITX1	0.0443389	55
V$PITX2_01	PITX2	PITX2	0.488822	54
V$OC2_Q3	OC-2	ONECUT2	0.202946	54
V$MATH1_Q2	MATH1	ATOH1	0.0690456	54
V$CRX_Q4_01	CRX	CRX	0.164671	53
V$CRX_02	Crx	CRX	0.164671	50
V$SP2_01	SP2	SP2	0.423427	50
V$MYOD_Q6_02	MyoD	MYOD1	0.0227158	48
V$YY1_02	YY1	YY1	0.20719	46
V$SPIB_03	Spi-B	SPIB	0.131892	43
V$CEBPG_Q6_01	C/EBPgamma	CEBPG	0.322518	41
V$P53_02	p53	TP53	0.561939	41
V$KAISO_01	KAISO	ZBTB33	0.14712	37
V$AP4_01	AP-4	TFAP4	0.0633867	35
V$RORBETA_Q2	RORBETA	RORB	0.0798613	32
V$NCX_02	Ncx	TLX2	0.132521	28
V$NMYC_01	N-Myc	MYCN	0.568005	21
V$MAX_01	Max	MAX	0.114148	21
V$CMYC_02	c-Myc	MYC	0.17121	21
V$USF2_Q6	USF2	USF2	0.0922049	21
V$ARNT_02	Arnt	ARNT	0.549961	21

V$ZID_01	ZID	ZBTB6	0.133683	20
V$SPIB_01	SPI-B	SPIB	0.131892	17

----

hsa-mir-30c-1

Matrix_id	transcription factor	Gene	PCC	Occurrence
V$PARP_Q4	PARP	PARP1	0.117036	11
V$PEA3_Q6	PEA3	ETV4	0.0465617	10
V$ETS2_Q6	c-Ets-2	ETS2	0.00354579	10
V$GATA1_04	GATA-1	GATA1	0.443552	10
V$GABPA_Q4	GABP-alpha	GABPA	0.341935	10
V$SPI1_Q5	SPI1	SPI1	0.0398986	10
V$TBX5_02	TBX5	TBX5	0.365512	10
V$GATA1_01	GATA-1	GATA1	0.443552	10
V$ZIC3_01	Zic3	ZIC3	0.480506	10
V$ETS1_B	c-Ets-1	ETS1	0.524781	10
V$P300_01	p300	EP300	0.279335	10
V$PUR1_Q4	PUR1	PURA	0.151781	10
V$CETS1_Q6	C-ets-1	ETS1	0.524781	10
V$AP2REP_01	AP-2rep	KLF12	0.0998161	9
V$MAFB_01	MAFB	MAFB	0.214208	9
V$NR1B2_Q6	NR1B2	RARB	0.362231	9
V$GATA1_02	GATA-1	GATA1	0.443552	9
V$LRF_Q2	LRF	ZBTB7A	0.360859	9
V$SOX5_01	SOX5	SOX5	0.157286	9
V$SPI1_03	SPI1	SPI1	0.0398986	9
V$ESE1_Q3	ESE-1	ELF3	0.191542	8
V$TBX5_01	TBX5	TBX5	0.365512	8
V$GATA6_01	GATA-6	GATA6	0.145863	8
V$AML1_Q6	AML1	RUNX1	0.276538	8
V$NKX32_01	Nkx3-2	NKX3-2	0.0119755	8
V$GATA3_02	GATA-3	GATA3	0.511997	8
V$GATA2_02	GATA-2	GATA2	0.420271	8
V$ERF_02	ERF	ERF	0.263652	8
V$GATA3_01	GATA-3	GATA3	0.511997	8
V$ER71_02	ER71	ETV2	0.544703	8
V$GATA2_01	GATA-2	GATA2	0.420271	8
V$HNF4A_Q6_01	HNF-4alpha	HNF4A	0.405462	8
V$ELK1_06	ELK-1	ELK1	0.336555	8
V$ING4_01	ING4	ING4	0.231193	8
V$TEL1_02	TEL1	ETV6	0.0558909	8
V$ETS2_B	c-Ets-2	ETS2	0.00354579	8
V$ELK1_02	Elk-1	ELK1	0.336555	8

V$GATA1_05	GATA-1	GATA1	0.443552	8
V$GATA1_06	GATA-1	GATA1	0.443552	8
V$ETV3_02	ETV3	ETV3	0.247285	8
V$CDX2_Q5_02	CDX-2	CDX2	0.0773589	7
V$TBP_Q6	TBP	TBP	0.172819	7
V$AHR_Q5	AhR	AHR	0.458135	7
V$ETV7_01	ETV7	ETV7	0.188985	7
V$PARP_Q3	PARP	PARP1	0.117036	7
V$ERBETA_Q5	ER-beta	ESR2	0.175032	7
V$CDX2_01	Cdx-2	CDX2	0.0773589	7
V$GABPBETA_Q3	GABP-beta	GABPB1	0.113317	7
V$YY1_Q6_03	YY1	YY1	0.0533827	7
V$AP4_Q6_02	AP-4	TFAP4	0.163986	7
V$CEBPE_Q6	CEBPE	CEBPE	0.698043	7
V$SP1_Q2_01	Sp1	SP1	0.425149	7
V$SP1_Q4_01	Sp1	SP1	0.425149	7
V$SP1_Q6_01	Sp1	SP1	0.425149	7
V$SP1_Q6	Sp1	SP1	0.425149	7
V$CP2_01	CP2	TFCP2	0.455918	7
V$VDR_Q3	VDR	VDR	0.316197	7
V$AP4_Q6	AP-4	TFAP4	0.163986	7
V$GR_Q6	GR	NR3C1	0.0926383	7
V$IRF4_Q6	IRF-4	IRF4	0.200228	6
V$IPF1_Q6	IPF1	PDX1	0.173078	6
V$DLX5_01	dlx5	DLX5	0.362244	6
V$BEN_01	BEN	GTF2IRD1	0.117714	6
V$SP1_01	Sp1	SP1	0.425149	6
V$YY1_01	YY1	YY1	0.0533827	6
V$ERR1_Q3	ERR1	ESRRA	0.474459	5
V$NKX22_02	NKX2B	NKX2-2	0.0324537	5
V$SP1_02	SP1	SP1	0.425149	5
V$CDX2_Q5_01	Cdx-2	CDX2	0.0773589	5
V$DAX1_01	Dax1	NR0B1	0.0584798	5
V$ZIC1_01	Zic1	ZIC1	0.0313765	5
V$NANOG_02	Nanog	NANOG	0.0216817	5
V$TEF1_Q6_03	TEF-1	TEAD1	0.144685	5
V$IPF1_01	IPF1	PDX1	0.173078	5
V$GR_01	GR	NR3C1	0.0926383	5
V$OC2_Q3	OC-2	ONECUT2	0.324418	5
V$YY1_Q6_02	YY1	YY1	0.0533827	5
V$GFI1_Q6	Gfi1	GFI1	0.0385197	5
V$ESE1_02	ESE-1	ELF3	0.191542	5
V$RFX1_01	RFX1	RFX1	0.0790295	5
V$RFX1_02	RFX1	RFX1	0.0790295	5
V$GFI1_Q6_01	Gfi1	GFI1	0.0385197	5
V$ELF5_01	ELF5	ELF5	0.114596	5

V$TEF1_Q6	TEF-1	TEAD1	0.144685	5
V$HOXD9_Q2	Hoxd9	HOXD9	0.232327	4
V$CDX1_01	Cdx-1	CDX1	0.0919045	4
V$GATA2_03	GATA-2	GATA2	0.420271	4
V$MSX1_01	Msx-1	MSX1	0.0785742	4
V$STAT4_Q5	STAT4	STAT4	0.142192	4
V$P53_02	p53	TP53	0.387158	4
V$CEBPG_Q6_01	C/EBPgamma	CEBPG	0.109431	4
V$NFAT2_Q5	NF-AT2	NFATC1	0.0756145	4
V$TCF4_01	TCF-4	TCF7L2	0.0763432	4
V$FOXO4_02	FOXO4	FOXO4	0.647267	4
V$AP2ALPHA_02	AP-2alphaA	TFAP2A	0.612433	4
V$HMGIY_01	HMGIY	HMGA1	0.0915854	4
V$YY1_Q6	YY1	YY1	0.0533827	4
V$GFI1B_01	Gfi1b	GFI1B	0.186411	4
V$CMAF_01	c-Maf	MAF	0.364913	4
V$HBP1_Q2	hbp1	HBP1	0.194312	4
V$CDX2_Q5	Cdx-2	CDX2	0.0773589	3
V$HOXA9_01	hoxa9	HOXA9	0.14679	3
V$CIZ_01	CIZ	ZNF384	0.338769	3
V$PET1_02	Pet-1	FEV	0.0999727	3
V$CRX_Q4_01	CRX	CRX	0.254824	3
V$NKX32_02	Nkx3-2	NKX3-2	0.0119755	3
V$TCF3_01	TCF-3	TCF7L1	0.203975	3
V$PIT1_Q6	Pit-1	POU1F1	0.423663	3
V$AR_03	AR	AR	0.0113955	3
V$ELK1_01	Elk-1	ELK1	0.336555	2
V$AML1_01	AML1a	RUNX1	0.276538	2
V$YY1_03	YY1	YY1	0.0533827	2
V$AR_Q2	AR	AR	0.0113955	2
V$POU3F2_01	POU3F2	POU3F2	0.0961158	2
V$CRX_Q4	Crx	CRX	0.254824	2
V$CEBPG_Q6	C/EBPgamma	CEBPG	0.109431	2
V$AML2_01	AML2	RUNX3	0.0657713	2
V$AML1_Q4	AML1	RUNX1	0.276538	2
V$OCT2_01	2-Oct	POU2F2	0.358288	2
V$GATA3_03	GATA-3	GATA3	0.511997	2
V$CTCF_01	CTCF	CTCF	0.0430055	1
V$HLF_01	HLF	HLF	0.190744	1
V$PAX3_B	Pax-3	PAX3	0.391964	1
V$IRF1_Q6_01	IRF-1	IRF1	0.387561	1
V$CTCF_02	CTCF	CTCF	0.0430055	1
V$GRE_C	GR	NR3C1	0.0926383	1
V$AFP1_Q6	AFP1	ZFHX3	0.00659987	1
V$PAX8_01	Pax-8	PAX8	0.166413	1
V$LHX3b_01	LHX3b	LHX3	0.294303	1

----

hsa-mir-30c-2

Matrix_id	transcription factor	Gene	PCC	Occurrence
V$SMAD4_Q6_01	Smad4	SMAD4	0.263174	11
V$PARP_Q4	PARP	PARP1	0.117036	11
V$GKLF_Q4	GKLF	KLF4	0.191386	11
V$Elf5_03	ELF5	ELF5	0.114596	10
V$ETS2_Q6	c-Ets-2	ETS2	0.00354579	10
V$PEA3_Q6	PEA3	ETV4	0.0465617	10
V$ETS1_B	c-Ets-1	ETS1	0.524781	10
V$ZIC3_01	Zic3	ZIC3	0.480506	10
V$SPI1_Q5	SPI1	SPI1	0.0398986	10
V$GATA1_04	GATA-1	GATA1	0.443552	10
V$PUR1_Q4	PUR1	PURA	0.151781	10
V$SMAD3_Q6_01	Smad3	SMAD3	0.029369	10
V$GABPA_Q4	GABP-alpha	GABPA	0.341935	10
V$CETS1_Q6	C-ets-1	ETS1	0.524781	10
V$P300_01	p300	EP300	0.279335	10
V$GATA1_01	GATA-1	GATA1	0.443552	10
V$MAZ_Q6	MAZ	MAZ	0.420309	9
V$SMAD3_Q6	SMAD3	SMAD3	0.029369	9
V$SOX5_01	SOX5	SOX5	0.157286	9
V$LRF_Q2	LRF	ZBTB7A	0.360859	9
V$SPI1_03	SPI1	SPI1	0.0398986	9
V$NR1B2_Q6	NR1B2	RARB	0.362231	9
V$FKLF_Q5	FKLF	KLF11	0.277643	9
V$GATA1_02	GATA-1	GATA1	0.443552	9
V$TFIII_Q6	TFII-I	GTF2I	0.492362	9
V$ESE1_Q3	ESE-1	ELF3	0.191542	8
V$AML2_Q3	AML2	RUNX3	0.0657713	8
V$HNF4A_Q6_01	HNF-4alpha	HNF4A	0.405462	8
V$ING4_01	ING4	ING4	0.231193	8
V$AML1_Q6	AML1	RUNX1	0.276538	8
V$MAZ_Q6_01	MAZ	MAZ	0.420309	8
V$ELK1_06	ELK-1	ELK1	0.336555	8
V$GATA2_01	GATA-2	GATA2	0.420271	8
V$GATA3_01	GATA-3	GATA3	0.511997	8
V$GATA1_06	GATA-1	GATA1	0.443552	8
V$TEL1_02	TEL1	ETV6	0.0558909	8
V$GATA3_02	GATA-3	GATA3	0.511997	8
V$ETV3_02	ETV3	ETV3	0.247285	8
V$GATA1_05	GATA-1	GATA1	0.443552	8

V$ER71_02	ER71	ETV2	0.544703	8
V$ELK1_02	Elk-1	ELK1	0.336555	8
V$GATA6_01	GATA-6	GATA6	0.145863	8
V$ERF_02	ERF	ERF	0.263652	8
V$GATA2_02	GATA-2	GATA2	0.420271	8
V$ETV7_01	ETV7	ETV7	0.188985	7
V$GR_Q6	GR	NR3C1	0.0926383	7
V$CDX2_Q5_02	CDX-2	CDX2	0.0773589	7
V$CEBPE_Q6	CEBPE	CEBPE	0.698043	7
V$CDX2_01	Cdx-2	CDX2	0.0773589	7
V$CEBPA_Q6	C/EBPalpha	CEBPA	0.0526319	7
V$CP2_01	CP2	TFCP2	0.455918	7
V$AP4_Q6_02	AP-4	TFAP4	0.163986	7
V$TBP_Q6	TBP	TBP	0.172819	7
V$YY1_Q6_03	YY1	YY1	0.0533827	7
V$PARP_Q3	PARP	PARP1	0.117036	7
V$YY1_01	YY1	YY1	0.0533827	6
V$CEBPD_Q6	C/EBPdelta	CEBPD	0.178651	6
V$IPF1_Q4_01	IPF1	PDX1	0.173078	6
V$IRF4_Q6	IRF-4	IRF4	0.200228	6
V$DLX5_01	dlx5	DLX5	0.362244	6
V$IPF1_Q6	IPF1	PDX1	0.173078	6
V$SOX10_Q6	SOX10	SOX10	0.028698	6
V$GFI1_Q6	Gfi1	GFI1	0.0385197	5
V$DAX1_01	Dax1	NR0B1	0.0584798	5
V$IPF1_Q4	IPF1	PDX1	0.173078	5
V$TEF1_Q6_03	TEF-1	TEAD1	0.144685	5
V$RFX1_02	RFX1	RFX1	0.0790295	5
V$CDX2_Q5_01	Cdx-2	CDX2	0.0773589	5
V$OC2_Q3	OC-2	ONECUT2	0.324418	5
V$RFX1_01	RFX1	RFX1	0.0790295	5
V$CEBPB_02	C/EBPbeta	CEBPB	0.294498	5
V$ESE1_02	ESE-1	ELF3	0.191542	5
V$TEF1_Q6	TEF-1	TEAD1	0.144685	5
V$CEBPB_Q6	C/EBPbeta	CEBPB	0.294498	5
V$YY1_Q6_02	YY1	YY1	0.0533827	5
V$NANOG_02	Nanog	NANOG	0.0216817	5
V$HOXD9_Q2	Hoxd9	HOXD9	0.232327	4
V$ZABC1_01	ZABC1	ZNF217	0.248168	4
V$CDX1_01	Cdx-1	CDX1	0.0919045	4
V$HMGIY_01	HMGIY	HMGA1	0.0915854	4
V$IPF1_02	IPF1	PDX1	0.173078	4
V$YY1_Q6	YY1	YY1	0.0533827	4
V$GATA2_03	GATA-2	GATA2	0.420271	4
V$HBP1_Q2	hbp1	HBP1	0.194312	4
V$CEBPA_01	C/EBPalpha	CEBPA	0.0526319	4

V$NFAT2_Q5	NF-AT2	NFATC1	0.0756145	4
V$PIT1_Q6	Pit-1	POU1F1	0.423663	3
V$PITX3_Q2	PITX3	PITX3	0.0904877	3
V$YY1_02	YY1	YY1	0.0533827	3
V$HNF1_02	HNF-1alpha	HNF1A	0.347324	3
V$TCF3_01	TCF-3	TCF7L1	0.203975	3
V$CRX_Q4_01	CRX	CRX	0.254824	3
V$FOXJ2_02	FOXJ2	FOXJ2	0.106583	2
V$GATA3_03	GATA-3	GATA3	0.511997	2
V$CRX_Q4	Crx	CRX	0.254824	2
V$AML1_Q4	AML1	RUNX1	0.276538	2
V$YY1_03	YY1	YY1	0.0533827	2
V$AP4_01	AP-4	TFAP4	0.163986	2
V$AML1_01	AML1a	RUNX1	0.276538	2
V$CEBPB_01	C/EBPbeta	CEBPB	0.294498	2
V$CDP_04	CDP	CUX1	0.261056	2
V$OCT2_01	2-Oct	POU2F2	0.358288	2
V$AML2_01	AML2	RUNX3	0.0657713	2
V$HNF1B_01	HNF-1beta	HNF1B	0.351188	1
V$PITX2_Q2	Pitx2	PITX2	0.320224	1
V$ATF5_01	ATF5	ATF5	0.0260239	1
V$PITX1_01	Pitx1	PITX1	0.0064856	1
V$PITX2_01	PITX2	PITX2	0.320224	1
V$CRX_02	Crx	CRX	0.254824	1
V$IRF1_Q6_01	IRF-1	IRF1	0.387561	1
V$IRF1_01	IRF-1	IRF1	0.387561	1

---

hsa-mir-30d

Matrix_id	transcription factor	Gene	PCC	Occurrence
V$PUR1_Q4	PUR1	PURA	0.175534	269
V$PEA3_Q6	PEA3	ETV4	0.130508	263
V$PARP_Q4	PARP	PARP1	0.115786	261
V$ELF1_Q6	Elf-1	ELF1	0.120131	256
V$GKLF_Q4	GKLF	KLF4	0.559967	254
V$P300_01	p300	EP300	0.394081	242
V$ZIC3_01	Zic3	ZIC3	0.0117067	241
V$ETS1_B	c-Ets-1	ETS1	0.178271	238
V$CETS1_Q6	C-ets-1	ETS1	0.178271	232
V$SMAD4_Q6_01	Smad4	SMAD4	0.330104	226
V$NR1B2_Q6	NR1B2	RARB	0.0515325	223
V$ETS2_Q6	c-Ets-2	ETS2	0.0837523	221
V$GABPA_Q4	GABP-alpha	GABPA	0.380104	220

V$GATA1_01	GATA-1	GATA1	0.146934	218
V$TBX5_02	TBX5	TBX5	0.194463	212
V$YY1_01	YY1	YY1	0.329077	204
V$SPI1_Q5	SPI1	SPI1	0.0111963	203
V$ETS2_B	c-Ets-2	ETS2	0.0837523	203
V$SPI1_03	SPI1	SPI1	0.0111963	198
V$SMAD3_Q6_01	Smad3	SMAD3	0.0101463	198
V$AML1_Q6	AML1	RUNX1	0.121086	193
V$MAZ_Q6	MAZ	MAZ	0.00748807	185
V$YY1_Q6	YY1	YY1	0.329077	184
V$YY1_Q6_02	YY1	YY1	0.329077	184
V$LRF_Q2	LRF	ZBTB7A	0.117021	184
V$GATA1_02	GATA-1	GATA1	0.146934	180
V$GATA1_05	GATA-1	GATA1	0.146934	168
V$GATA1_06	GATA-1	GATA1	0.146934	168
V$GATA2_02	GATA-2	GATA2	0.721457	168
V$ERBETA_Q5	ER-beta	ESR2	0.101024	167
V$ING4_01	ING4	ING4	0.103379	166
V$E12_Q6	E12	TCF3	0.0187866	164
V$E2A_Q6	E2A	TCF3	0.0187866	164
V$E47_02	E47	TCF3	0.0187866	162
V$SP1_Q6	Sp1	SP1	0.436665	158
V$AP2ALPHA_Q6	AP-2alpha	TFAP2A	0.95564	155
V$GATA1_04	GATA-1	GATA1	0.146934	153
V$MYOGENIN_Q6	myogenin	MYOG	0.0113833	149
V$GATA3_01	GATA-3	GATA3	0.886369	147
V$AP2ALPHA_01	AP-2alpha	TFAP2A	0.95564	145
V$GATA3_02	GATA-3	GATA3	0.886369	145
V$SP1_Q6_01	Sp1	SP1	0.436665	144
V$FOXO3A_Q1	FOXO3A	FOXO3	0.023021	144
V$TFIII_Q6	TFII-I	GTF2I	0.193407	142
V$SREBP1_Q6	SREBP-1	SREBF1	0.0892681	139
V$E2A_Q2	E2A	TCF3	0.0187866	139
V$SP1_Q4_01	Sp1	SP1	0.436665	138
V$SP1_Q2_01	Sp1	SP1	0.436665	136
V$ARNT_01	Arnt	ARNT	0.4258	134
V$GATA2_01	GATA-2	GATA2	0.721457	134
V$TEF1_Q6_03	TEF-1	TEAD1	0.0473578	131
V$TEF1_Q6	TEF-1	TEAD1	0.0473578	125
V$CEBPE_Q6	CEBPE	CEBPE	0.202622	121
V$CEBPA_Q6	C/EBPalpha	CEBPA	0.0605995	117
V$OC2_Q3	OC-2	ONECUT2	0.00740365	112
V$MYOGENIN_Q6_01	myogenin	MYOG	0.0113833	110
V$PITX2_01	PITX2	PITX2	0.48746	109
V$CP2_01	CP2	TFCP2	0.575217	107
V$AP2GAMMA_01	AP-2gamma	TFAP2C	0.865652	106

V$SP2_01	SP2	SP2	0.281322	100
V$SMAD3_Q6	SMAD3	SMAD3	0.0101463	99
V$NEUROD_02	NeuroD	NEUROD1	0.00943963	96
V$CEBPG_Q6_01	C/EBPgamma	CEBPG	0.518326	82
V$YY1_02	YY1	YY1	0.329077	80
V$KAISO_01	KAISO	ZBTB33	0.00896647	72
V$P53_02	p53	TP53	0.620772	70
V$NCX_02	Ncx	TLX2	0.0145846	66
V$RORBETA_Q2	RORBETA	RORB	0.0184015	55
V$ARNT_02	Arnt	ARNT	0.4258	45
V$NMYC_01	N-Myc	MYCN	0.576199	45
V$MAX_01	Max	MAX	0.0880738	43
V$CMYC_02	c-Myc	MYC	0.357202	42
V$ZID_01	ZID	ZBTB6	0.195389	29
V$SREBP1_01	SREBP-1	SREBF1	0.0892681	21

----

hsa-mir-30e

Matrix_id	transcription factor	Gene	PCC	Occurrence
V$MAFB_01	MAFB	MAFB	0.11071	88
V$AP2REP_01	AP-2rep	KLF12	0.13813	87
V$GATA1_01	GATA-1	GATA1	0.60261	87
V$ZIC3_01	Zic3	ZIC3	0.0963733	86
V$TBP_Q6	TBP	TBP	0.0731733	80
V$CDX2_Q5_02	CDX-2	CDX2	0.0351697	80
V$TBX5_02	TBX5	TBX5	0.403674	80
V$AML1_Q6	AML1	RUNX1	0.27408	76
V$NR1B2_Q6	NR1B2	RARB	0.305332	76
V$CETS1_Q6	C-ets-1	ETS1	0.62958	75
V$ETS1_B	c-Ets-1	ETS1	0.62958	72
V$GABPA_Q4	GABP-alpha	GABPA	0.0814976	70
V$HNF4A_Q6_01	HNF-4alpha	HNF4A	0.440155	70
V$TBX5_01	TBX5	TBX5	0.403674	65
V$ING4_01	ING4	ING4	0.227516	62
V$SPI1_03	SPI1	SPI1	0.303594	61
V$IPF1_Q6	IPF1	PDX1	0.0870329	61
V$ERBETA_Q5	ER-beta	ESR2	0.120071	60
V$NKX32_01	Nkx3-2	NKX3-2	0.0238231	60
V$SPI1_Q5	SPI1	SPI1	0.303594	59
V$LRF_Q2	LRF	ZBTB7A	0.356303	59
V$GATA6_01	GATA-6	GATA6	0.435222	59
V$GATA1_06	GATA-1	GATA1	0.60261	59
V$GATA1_05	GATA-1	GATA1	0.60261	59

V$SOX5_01	SOX5	SOX5	0.175253	58
V$CDX2_01	Cdx-2	CDX2	0.0351697	58
V$CDX2_Q5_01	Cdx-2	CDX2	0.0351697	58
V$BEN_01	BEN	GTF2IRD1	0.00655114	57
V$SP1_Q6	Sp1	SP1	0.176856	57
V$SP1_01	Sp1	SP1	0.176856	56
V$NFAT2_Q5	NF-AT2	NFATC1	0.0986876	54
V$GATA1_02	GATA-1	GATA1	0.60261	53
V$TEL1_02	TEL1	ETV6	0.0037906	51
V$ELK1_02	Elk-1	ELK1	0.151045	51
V$CRX_Q4	Crx	CRX	0.259786	50
V$ETV3_02	ETV3	ETV3	0.686398	50
V$SP1_Q6_01	Sp1	SP1	0.176856	49
V$ER71_02	ER71	ETV2	0.65428	49
V$ELK1_06	ELK-1	ELK1	0.151045	49
V$ERF_02	ERF	ERF	0.546946	48
V$GATA1_04	GATA-1	GATA1	0.60261	47
V$ETV7_01	ETV7	ETV7	0.0664378	47
V$IRF4_Q6	IRF-4	IRF4	0.0635086	47
V$IPF1_01	IPF1	PDX1	0.0870329	46
V$GFI1_Q6	Gfi1	GFI1	0.0566	46
V$SP1_Q4_01	Sp1	SP1	0.176856	46
V$SP1_Q2_01	Sp1	SP1	0.176856	46
V$TCF3_01	TCF-3	TCF7L1	0.234369	45
V$SP1_02	SP1	SP1	0.176856	44
V$TEF1_Q6_03	TEF-1	TEAD1	0.038797	44
V$GFI1_Q6_01	Gfi1	GFI1	0.0566	44
V$ERR1_Q3	ERR1	ESRRA	0.494654	42
V$TEF1_Q6	TEF-1	TEAD1	0.038797	41
V$AML1_01	AML1a	RUNX1	0.27408	41
V$ELF5_01	ELF5	ELF5	0.196616	40
V$GFI1B_01	Gfi1b	GFI1B	0.295415	39
V$NKX22_02	NKX2B	NKX2-2	0.0169419	37
V$PET1_02	Pet-1	FEV	0.0903668	36
V$VDR_Q3	VDR	VDR	0.201648	36
V$AML1_Q4	AML1	RUNX1	0.27408	36
V$KLF15_Q2	KLF15	KLF15	0.0508774	35
V$OC2_Q3	OC-2	ONECUT2	0.355734	33
V$CRX_Q4_01	CRX	CRX	0.259786	29
V$NKX32_02	Nkx3-2	NKX3-2	0.0238231	28
V$DAX1_01	Dax1	NR0B1	0.0926887	28
V$ELK1_01	Elk-1	ELK1	0.151045	28
V$CEBPE_Q6	CEBPE	CEBPE	0.851436	27
V$DBP_Q6_01	DBP	DBP	0.0325909	26
V$CDX1_01	Cdx-1	CDX1	0.294083	26
V$HOXD9_Q2	Hoxd9	HOXD9	0.239078	26

V$LHX3b_01	LHX3b	LHX3	0.211574	26
V$P53_02	p53	TP53	0.0754324	25
V$AML2_01	AML2	RUNX3	0.423991	24
V$PIT1_Q6	Pit-1	POU1F1	0.588843	23
V$CDX2_Q5	Cdx-2	CDX2	0.0351697	22
V$STAT4_Q5	STAT4	STAT4	0.0982247	22
V$IRF1_Q6_01	IRF-1	IRF1	0.279224	20
V$PAX3_B	Pax-3	PAX3	0.528841	17
V$AP2ALPHA_02	AP-2alphaA	TFAP2A	0.0158611	16
V$CIZ_01	CIZ	ZNF384	0.127713	14
V$AR_03	AR	AR	0.399035	13
V$OCT2_01	2-Oct	POU2F2	0.227112	8
V$AR_Q2	AR	AR	0.399035	5
V$POU3F2_01	POU3F2	POU3F2	0.12024	3

----

hsa-mir-31

Matrix_id	transcription factor	Gene	PCC	Occurrence
V$P300_01	p300	EP300	0.181118	4
V$MAFB_01	MAFB	MAFB	0.164461	4
V$TBP_Q6	TBP	TBP	0.16974	4
V$ETS1_B	c-Ets-1	ETS1	0.0520794	4
V$ELF1_Q6	Elf-1	ELF1	0.452258	4
V$SMAD4_Q6_01	Smad4	SMAD4	0.195766	4
V$SRY_02	SRY	SRY	0.00045333	4
V$PUR1_Q4	PUR1	PURA	0.385098	4
V$SOX9_B1	SOX9	SOX9	0.528237	4
V$ZIC3_01	Zic3	ZIC3	0.172575	4
V$GR_Q6	GR	NR3C1	0.230328	4
V$PARP_Q4	PARP	PARP1	0.072931	4
V$GATA1_01	GATA-1	GATA1	0.003667	4
V$GR_01	GR	NR3C1	0.230328	4
V$SOX5_01	SOX5	SOX5	0.0687006	4
V$CMAF_01	c-Maf	MAF	0.256745	3
V$PARP_Q3	PARP	PARP1	0.072931	3
V$SOX9_Q4	SOX9	SOX9	0.528237	3
V$PBX1_04	Pbx1	PBX1	0.149024	3
V$DLX5_01	dlx5	DLX5	0.32871	3
V$YY1_Q6_03	YY1	YY1	0.514914	3
V$PBX1_Q3	Pbx1	PBX1	0.149024	3
V$SOX10_Q6	SOX10	SOX10	0.0389813	3
V$GATA1_05	GATA-1	GATA1	0.003667	3
V$GATA1_06	GATA-1	GATA1	0.003667	3

V$GATA2_02	GATA-2	GATA2	0.454981	3
V$GATA3_01	GATA-3	GATA3	0.524188	3
V$YY1_01	YY1	YY1	0.514914	3
V$FAC1_01	FAC1	BPTF	0.179193	3
V$GATA1_02	GATA-1	GATA1	0.003667	2
V$CEBPG_Q6_01	C/EBPgamma	CEBPG	0.459439	2
V$HOXA9_01	hoxa9	HOXA9	0.0876939	2
V$GATA1_04	GATA-1	GATA1	0.003667	2
V$SOX2_Q6	SOX2	SOX2	0.141134	2
V$STAT1_05	STAT1	STAT1	0.172558	2
V$MEF2A_Q6	mef2A	MEF2A	0.144564	2
V$GATA2_01	GATA-2	GATA2	0.454981	2
V$IRF1_Q6	IRF-1	IRF1	0.0145733	2
V$ZID_01	ZID	ZBTB6	0.154104	1
V$TCF4_Q5	TCF-4	TCF7L2	0.479506	1
V$TCF4_01	TCF-4	TCF7L2	0.479506	1
V$HMGIY_01	HMGIY	HMGA1	0.368383	1
V$P53_02	p53	TP53	0.0434883	1
V$AMEF2_Q6	aMEF-2	MEF2A	0.144564	1
V$GATA2_03	GATA-2	GATA2	0.454981	1
V$GATA3_02	GATA-3	GATA3	0.524188	1
V$TCF4_Q5_01	TCF-4	TCF7L2	0.479506	1

----

hsa-mir-320a

Matrix_id	transcription factor	Gene	PCC	Occurrence
V$PUR1_Q4	PUR1	PURA	0.221011	206
V$PEA3_Q6	PEA3	ETV4	0.0414501	199
V$ELF1_Q6	Elf-1	ELF1	0.243735	196
V$MAFB_01	MAFB	MAFB	0.260827	189
V$P300_01	p300	EP300	0.23833	185
V$SMAD4_Q6_01	Smad4	SMAD4	0.289483	173
V$GR_Q6	GR	NR3C1	0.16184	160
V$YY1_01	YY1	YY1	0.384438	156
V$TBP_Q6	TBP	TBP	0.0163815	155
V$YY1_Q6_02	YY1	YY1	0.384438	143
V$DLX5_01	dlx5	DLX5	0.617143	138
V$GATA2_02	GATA-2	GATA2	0.65942	128
V$HMGIY_01	HMGIY	HMGA1	0.230045	110
V$GATA3_01	GATA-3	GATA3	0.828443	109
V$SREBP1_Q6	SREBP-1	SREBF1	0.188508	108
V$HIF1A_Q6	HIF-1alpha	HIF1A	0.10311	108
V$GATA3_02	GATA-3	GATA3	0.828443	108

V$ARNT_01	Arnt	ARNT	0.206678	107
V$YY1_Q6_03	YY1	YY1	0.384438	104
V$TCF4_01	TCF-4	TCF7L2	0.632587	98
V$TCF4_Q5	TCF-4	TCF7L2	0.632587	88
V$FOXJ2_01	FOXJ2	FOXJ2	0.0110052	85
V$IRF7_Q3	IRF-7	IRF7	0.0368365	83
V$GATA3_03	GATA-3	GATA3	0.828443	77
V$CEBPD_Q6	C/EBPdelta	CEBPD	0.432222	72
V$CEBPB_02	C/EBPbeta	CEBPB	0.502583	68
V$CEBPG_Q6_01	C/EBPgamma	CEBPG	0.470147	66
V$GATA2_03	GATA-2	GATA2	0.65942	62
V$CEBPB_Q6	C/EBPbeta	CEBPB	0.502583	58
V$DEC2_Q2	2-Dec	BHLHE41	0.0553488	46
V$IRF1_Q6_01	IRF-1	IRF1	0.222741	40
V$CEBPB_01	C/EBPbeta	CEBPB	0.502583	38
V$CEBPG_Q6	C/EBPgamma	CEBPG	0.470147	38
V$IRF1_Q6	IRF-1	IRF1	0.222741	34
V$FOXO4_01	FOXO4	FOXO4	0.8894	29
V$CDP_04	CDP	CUX1	0.460219	27
V$GLI3_Q5_01	GLI3	GLI3	0.207304	27

----

hsa-mir-323a

Matrix_id	transcription factor	Gene	PCC	Occurrence
V$PUR1_Q4	PUR1	PURA	0.264644	30
V$PARP_Q4	PARP	PARP1	0.345641	29
V$ETS2_B	c-Ets-2	ETS2	0.0237793	27
V$MAFB_01	MAFB	MAFB	0.103148	26
V$GABPA_Q4	GABP-alpha	GABPA	0.165594	25
V$SOX9_Q4	SOX9	SOX9	0.0977431	24
V$YY1_01	YY1	YY1	0.187616	24
V$AP4_Q6_02	AP-4	TFAP4	0.209823	23
V$NR1B2_Q6	NR1B2	RARB	0.0138754	23
V$HNF4A_Q6_01	HNF-4alpha	HNF4A	0.1079	22
V$ETS2_Q6	c-Ets-2	ETS2	0.0237793	22
V$YY1_Q6_02	YY1	YY1	0.187616	22
V$YY1_Q6	YY1	YY1	0.187616	21
V$ING4_01	ING4	ING4	0.402796	20
V$AHR_Q5	AhR	AHR	0.0232897	18
V$PBX1_04	Pbx1	PBX1	0.326244	18
V$BEN_01	BEN	GTF2IRD1	0.00240317	18
V$AP2ALPHA_Q6	AP-2alpha	TFAP2A	0.0408258	16
V$GATA3_01	GATA-3	GATA3	0.0160458	15

V$CREM_Q6	CREM	CREM	0.42257	13
V$GABPBETA_Q3	GABP-beta	GABPB1	0.022182	13
V$SOX10_Q6	SOX10	SOX10	0.459172	13
V$PIT1_Q6	Pit-1	POU1F1	0.220041	12
V$CEBPB_02	C/EBPbeta	CEBPB	0.0347624	12
V$OC2_Q3	OC-2	ONECUT2	0.0735801	10
V$ELK1_01	Elk-1	ELK1	0.260017	9
V$ATF4_Q6	ATF-4	ATF4	0.0834233	9
V$ATF2_Q5	ATF-2	ATF2	0.00068644	8
V$AP4_01	AP-4	TFAP4	0.209823	7
V$YY1_02	YY1	YY1	0.187616	7
V$MAFK_Q3	MafK	MAFK	0.0743458	6
V$SOX2_Q6	SOX2	SOX2	0.210164	6
V$POU3F2_02	POU3F2	POU3F2	0.0426044	2

----

hsa-mir-324

Matrix_id	transcription factor	Gene	PCC	Occurrence
V$PUR1_Q4	PUR1	PURA	0.493825	111
V$ELF1_Q6	Elf-1	ELF1	0.0867232	103
V$MAFB_01	MAFB	MAFB	0.246264	103
V$GKLF_Q4	GKLF	KLF4	0.0953329	103
V$P300_01	p300	EP300	0.0366185	102
V$PARP_Q4	PARP	PARP1	0.369295	102
V$GABPA_Q4	GABP-alpha	GABPA	0.123118	91
V$TBP_Q6	TBP	TBP	0.022735	90
V$ETS2_Q6	c-Ets-2	ETS2	0.103604	87
V$YY1_01	YY1	YY1	0.370041	87
V$SOX9_Q4	SOX9	SOX9	0.280073	83
V$MEF2C_Q4	MEF-2C	MEF2C	0.573017	83
V$AHR_Q5	AhR	AHR	0.168963	83
V$ETS2_B	c-Ets-2	ETS2	0.103604	77
V$SOX9_B1	SOX9	SOX9	0.280073	76
V$HNF4A_Q6_01	HNF-4alpha	HNF4A	0.0192666	70
V$SRY_02	SRY	SRY	0.0278176	69
V$ING4_01	ING4	ING4	0.500808	67
V$ZBP89_Q4	ZBP89	ZNF148	0.0350651	65
V$PARP_Q3	PARP	PARP1	0.369295	64
V$FOXO3A_Q1	FOXO3A	FOXO3	0.0342312	63
V$PBX1_Q3	Pbx1	PBX1	0.352887	60
V$PBX1_04	Pbx1	PBX1	0.352887	58
V$IRF8_Q6	IRF-8	IRF8	0.0877557	54
V$FOXJ2_01	FOXJ2	FOXJ2	0.0539899	50

V$CREM_Q6	CREM	CREM	0.278181	46
V$HIF1A_Q6	HIF-1alpha	HIF1A	0.316272	46
V$GABPBETA_Q3	GABP-beta	GABPB1	0.0938843	44
V$TCF4_01	TCF-4	TCF7L2	0.089921	43
V$CP2_01	CP2	TFCP2	0.478285	43
V$EGR1_02	EGR-1	EGR1	0.19054	43
V$SP2_01	SP2	SP2	0.179139	41
V$SOX10_Q6	SOX10	SOX10	0.537846	39
V$FOXO4_02	FOXO4	FOXO4	0.226839	38
V$CEBPB_Q6	C/EBPbeta	CEBPB	0.0198975	37
V$HBP1_Q2	hbp1	HBP1	0.014323	35
V$ELK1_01	Elk-1	ELK1	0.1987	34
V$ATF1_Q6_01	ATF-1	ATF1	0.0260456	34
V$CACCCBINDINGFACT	CACCC-binding factor	ZNF148	0.0350651	30
V$NURR1_Q3	NURR1	NR4A2	0.417963	29
V$ZIC1_01	Zic1	ZIC1	0.72097	28
V$CIZ_01	CIZ	ZNF384	0.11844	20
V$EGR2_01	Egr-2	EGR2	0.124168	18
V$RORBETA_Q2	RORBETA	RORB	0.142094	18
V$PAX3_01	Pax-3	PAX3	0.00412714	3

----

hsa-mir-328

Matrix_id	transcription factor	Gene	PCC	Occurrence
V$PUR1_Q4	PUR1	PURA	0.35413	287
V$PARP_Q4	PARP	PARP1	0.376363	266
V$ZIC3_01	Zic3	ZIC3	0.0770942	262
V$MAFB_01	MAFB	MAFB	0.015989	261
V$SOX9_Q4	SOX9	SOX9	0.264574	230
V$GABPA_Q4	GABP-alpha	GABPA	0.0225432	230
V$TBP_Q6	TBP	TBP	0.0204302	230
V$Elf5_03	ELF5	ELF5	0.00770155	218
V$AP4_Q6_02	AP-4	TFAP4	0.389375	210
V$YY1_01	YY1	YY1	0.0958951	209
V$BEN_01	BEN	GTF2IRD1	0.0363937	206
V$MEF2C_Q4	MEF-2C	MEF2C	0.720637	202
V$ETS2_B	c-Ets-2	ETS2	0.149895	196
V$SOX9_B1	SOX9	SOX9	0.264574	194
V$YY1_Q6	YY1	YY1	0.0958951	194
V$DLX5_01	dlx5	DLX5	0.0353274	192
V$YY1_Q6_02	YY1	YY1	0.0958951	189
V$GATA6_01	GATA-6	GATA6	0.204651	178
V$PARP_Q3	PARP	PARP1	0.376363	174

V$ING4_01	ING4	ING4	0.471832	169
V$FOXO3A_Q1	FOXO3A	FOXO3	0.0410734	164
V$PITX3_Q2	PITX3	PITX3	0.22856	158
V$PBX1_04	Pbx1	PBX1	0.372444	158
V$PBX1_Q3	Pbx1	PBX1	0.372444	150
V$YY1_Q6_03	YY1	YY1	0.0958951	142
V$FOXJ2_01	FOXJ2	FOXJ2	0.0879686	133
V$EGR1_02	EGR-1	EGR1	0.211019	129
V$CREM_Q6	CREM	CREM	0.2478	126
V$CP2_01	CP2	TFCP2	0.257379	103
V$ZIC1_01	Zic1	ZIC1	0.829281	102
V$ELF5_01	ELF5	ELF5	0.00770155	102
V$OC2_Q3	OC-2	ONECUT2	0.0476887	99
V$ATF1_Q6_01	ATF-1	ATF1	0.00375509	97
V$FAC1_01	FAC1	BPTF	0.146555	94
V$MEF2A_Q6	mef2A	MEF2A	0.595996	93
V$PIT1_Q6	Pit-1	POU1F1	0.110538	91
V$NURR1_Q3	NURR1	NR4A2	0.48402	86
V$YY1_02	YY1	YY1	0.0958951	85
V$ATF4_Q6	ATF-4	ATF4	0.0787672	70
V$BEN_02	BEN	GTF2IRD1	0.0363937	67
V$CIZ_01	CIZ	ZNF384	0.0534678	65
V$ATF2_Q5	ATF-2	ATF2	0.060026	51
V$POU6F1_03	POU6F1	POU6F1	0.299797	33
V$SATB1_Q3	SATB1	SATB1	0.164416	30
V$POU6F1_02	POU6F1	POU6F1	0.299797	27

----

hsa-mir-330

Matrix_id	transcription factor	Gene	PCC	Occurrence
V$PUR1_Q4	PUR1	PURA	0.419204	491
V$MAFB_01	MAFB	MAFB	0.17072	455
V$ZIC3_01	Zic3	ZIC3	0.198149	445
V$ETS2_Q6	c-Ets-2	ETS2	0.195107	409
V$SOX9_Q4	SOX9	SOX9	0.241243	395
V$GABPA_Q4	GABP-alpha	GABPA	0.140881	395
V$TBP_Q6	TBP	TBP	0.024102	386
V$NR1B2_Q6	NR1B2	RARB	0.00106793	385
V$AP4_Q6_02	AP-4	TFAP4	0.416124	367
V$MAZ_Q6	MAZ	MAZ	0.224981	361
V$YY1_01	YY1	YY1	0.0987488	359
V$MEF2C_Q4	MEF-2C	MEF2C	0.812276	351
V$YY1_Q6_02	YY1	YY1	0.0987488	332

V$ETS2_B	c-Ets-2	ETS2	0.195107	332
V$YY1_Q6	YY1	YY1	0.0987488	332
V$DLX5_01	dlx5	DLX5	0.00737451	327
V$TFIII_Q6	TFII-I	GTF2I	0.268589	289
V$ING4_01	ING4	ING4	0.693231	285
V$ELK1_02	Elk-1	ELK1	0.110979	273
V$PITX3_Q2	PITX3	PITX3	0.0416587	260
V$AP4_Q5	AP-4	TFAP4	0.416124	254
V$PBX1_Q3	Pbx1	PBX1	0.230773	254
V$YY1_Q6_03	YY1	YY1	0.0987488	250
V$ELK1_06	ELK-1	ELK1	0.110979	238
V$AP4_Q6	AP-4	TFAP4	0.416124	232
V$KLF15_Q2	KLF15	KLF15	0.0413249	222
V$IRF8_Q6	IRF-8	IRF8	0.092706	216
V$ERF_02	ERF	ERF	0.239771	213
V$NEUROD_02	NeuroD	NEUROD1	0.100747	174
V$SP4_Q5	SP4	SP4	0.0420767	164
V$AP4_Q6_01	AP-4	TFAP4	0.416124	162
V$NKX2B_Q3	NKX2B	NKX2-2	0.881091	157
V$NKX22_02	NKX2B	NKX2-2	0.881091	151
V$MEF2A_Q6	mef2A	MEF2A	0.67377	149
V$CACCCBINDINGFACT	CACCC-binding factor	ZNF148	0.11751	119
V$DEC2_Q2	2-Dec	BHLHE41	0.672485	96
V$EGR2_01	Egr-2	EGR2	0.221246	81
V$E2F1_Q4	E2F-1	E2F1	0.138356	80
V$RORBETA_Q2	RORBETA	RORB	0.328927	77
V$CDP_02	CDP	CUX1	0.0613238	15

----

hsa-mir-331

Matrix_id	transcription factor	Gene	PCC	Occurrence
V$GKLF_Q4	GKLF	KLF4	0.111368	20
V$PARP_Q4	PARP	PARP1	0.628062	19
V$PUR1_Q4	PUR1	PURA	0.465841	19
V$ZIC3_01	Zic3	ZIC3	0.198548	18
V$SMAD4_Q6_01	Smad4	SMAD4	0.259763	18
V$AP2REP_01	AP-2rep	KLF12	0.250866	18
V$PEA3_Q6	PEA3	ETV4	0.239717	18
V$P300_01	p300	EP300	0.396224	18
V$MAFB_01	MAFB	MAFB	0.154707	17
V$GATA1_01	GATA-1	GATA1	0.135945	16
V$AHR_Q5	AhR	AHR	0.332835	16
V$AP4_Q6_02	AP-4	TFAP4	0.40995	16

V$YY1_Q6_02	YY1	YY1	0.393942	15
V$GABPA_Q4	GABP-alpha	GABPA	0.387096	15
V$CETS1_Q6	C-ets-1	ETS1	0.1203	15
V$NR1B2_Q6	NR1B2	RARB	0.312845	15
V$ETS1_B	c-Ets-1	ETS1	0.1203	15
V$YY1_Q6	YY1	YY1	0.393942	14
V$AML1_Q6	AML1	RUNX1	0.249442	14
V$TBP_Q6	TBP	TBP	0.242066	14
V$DLX5_01	dlx5	DLX5	0.471571	14
V$ETS2_B	c-Ets-2	ETS2	0.329858	14
V$CDX2_Q5_02	CDX-2	CDX2	0.19939	14
V$ETS2_Q6	c-Ets-2	ETS2	0.329858	13
V$TEL1_02	TEL1	ETV6	0.243051	13
V$IPF1_01	IPF1	PDX1	0.223324	13
V$ETV3_02	ETV3	ETV3	0.131544	13
V$CDX2_Q5_01	Cdx-2	CDX2	0.19939	12
V$ERBETA_Q5	ER-beta	ESR2	0.171018	12
V$HNF4A_Q6_01	HNF-4alpha	HNF4A	0.123791	12
V$MEF2C_Q4	MEF-2C	MEF2C	0.646327	12
V$ELK1_02	Elk-1	ELK1	0.532463	12
V$ER71_02	ER71	ETV2	0.0725933	12
V$ELK1_06	ELK-1	ELK1	0.532463	12
V$IPF1_Q6	IPF1	PDX1	0.223324	11
V$ERF_02	ERF	ERF	0.179298	11
V$CREM_Q6	CREM	CREM	0.482647	11
V$IPF1_Q4_01	IPF1	PDX1	0.223324	11
V$SOX9_B1	SOX9	SOX9	0.119318	11
V$CP2_01	CP2	TFCP2	0.654957	11
V$SOX5_01	SOX5	SOX5	0.0301567	11
V$PITX3_Q2	PITX3	PITX3	0.478068	11
V$HNF3A_01	HNF3A	FOXA1	0.032076	10
V$NFAT2_Q5	NF-AT2	NFATC1	0.193243	10
V$SREBP1_Q6	SREBP-1	SREBF1	0.445009	10
V$SRY_02	SRY	SRY	0.220141	10
V$RFX1_01	RFX1	RFX1	0.229449	10
V$GATA3_01	GATA-3	GATA3	0.331739	10
V$ATF1_Q6_01	ATF-1	ATF1	0.0751312	10
V$IRF4_Q6	IRF-4	IRF4	0.232655	10
V$YY1_Q6_03	YY1	YY1	0.393942	10
V$CDX2_01	Cdx-2	CDX2	0.19939	10
V$LHX3b_01	LHX3b	LHX3	0.373223	9
V$NKX32_01	Nkx3-2	NKX3-2	0.14343	9
V$HMGIY_01	HMGIY	HMGA1	0.102541	9
V$HBP1_Q2	hbp1	HBP1	0.331736	8
V$IPF1_02	IPF1	PDX1	0.223324	8
V$NANOG_02	Nanog	NANOG	0.157092	8

V$FOXO3A_Q1	FOXO3A	FOXO3	0.209402	8
V$PBX1_04	Pbx1	PBX1	0.0610794	8
V$IPF1_Q4	IPF1	PDX1	0.223324	8
V$FOXJ2_01	FOXJ2	FOXJ2	0.38801	8
V$CART1_02	CART1	ALX1	0.303745	7
V$HOX13_02	HOXA5	HOXA5	0.32138	7
V$OC2_Q3	OC-2	ONECUT2	0.34472	7
V$GATA6_01	GATA-6	GATA6	0.207091	7
V$GATA1_06	GATA-1	GATA1	0.135945	7
V$GATA3_02	GATA-3	GATA3	0.331739	7
V$GATA2_02	GATA-2	GATA2	0.349183	7
V$GATA1_05	GATA-1	GATA1	0.135945	7
V$FAC1_01	FAC1	BPTF	0.0649903	6
V$HNF3B_Q6	HNF-3beta	FOXA2	0.0555891	6
V$DBP_Q6_01	DBP	DBP	0.37896	6
V$STAT3_03	STAT3	STAT3	0.1754	6
V$FOXP3_01	FOXP3	FOXP3	0.246946	6
V$IPF1_06	ipf1	PDX1	0.223324	6
V$IPF1_03	IPF1	PDX1	0.223324	6
V$YY1_03	YY1	YY1	0.393942	5
V$HOXA9_01	hoxa9	HOXA9	0.03981	5
V$LHX3_01	Lhx3	LHX3	0.373223	5
V$CEBPE_Q6	CEBPE	CEBPE	0.152995	5
V$YY1_02	YY1	YY1	0.393942	5
V$CEBPB_02	C/EBPbeta	CEBPB	0.325866	4
V$MSX1_01	Msx-1	MSX1	0.223067	4
V$GATA2_03	GATA-2	GATA2	0.349183	4
V$HOXD9_Q2	Hoxd9	HOXD9	0.0679966	4
V$EAR2_Q2	EAR2	NR2F6	0.36846	4
V$PIT1_Q6	Pit-1	POU1F1	0.274542	4
V$STAT4_Q5	STAT4	STAT4	0.301812	3
V$POU6F1_02	POU6F1	POU6F1	0.441364	3
V$STAT1_Q6	STAT1	STAT1	0.0630329	3
V$CDX2_Q5	Cdx-2	CDX2	0.19939	3
V$STAT1_05	STAT1	STAT1	0.0630329	2
V$POU3F2_02	POU3F2	POU3F2	0.373993	2
V$POU6F1_03	POU6F1	POU6F1	0.441364	2
V$LHX3A_01	Lhx3a	LHX3	0.373223	1

---

hsa-mir-335

Matrix_id	transcription factor	Gene	PCC	Occurrence
V$PUR1_Q4	PUR1	PURA	0.150427	244

V$PEA3_Q6	PEA3	ETV4	0.0443719	237
V$PARP_Q4	PARP	PARP1	0.0760486	236
V$ELF1_Q6	Elf-1	ELF1	0.172088	233
V$GKLF_Q4	GKLF	KLF4	0.550471	229
V$MAFB_01	MAFB	MAFB	0.304288	222
V$SMAD4_Q6_01	Smad4	SMAD4	0.250583	208
V$GABPA_Q4	GABP-alpha	GABPA	0.353425	197
V$TBP_Q6	TBP	TBP	0.11378	187
V$GR_Q6	GR	NR3C1	0.11708	186
V$YY1_01	YY1	YY1	0.375681	182
V$YY1_Q6	YY1	YY1	0.375681	167
V$YY1_Q6_02	YY1	YY1	0.375681	166
V$DLX5_01	dlx5	DLX5	0.681671	166
V$GATA2_02	GATA-2	GATA2	0.640878	158
V$ING4_01	ING4	ING4	0.0994541	147
V$PARP_Q3	PARP	PARP1	0.0760486	147
V$FKLF_Q5	FKLF	KLF11	0.23254	137
V$HMGIY_01	HMGIY	HMGA1	0.319737	137
V$GATA3_01	GATA-3	GATA3	0.893577	131
V$SREBP1_Q6	SREBP-1	SREBF1	0.233227	129
V$YY1_Q6_03	YY1	YY1	0.375681	124
V$GATA2_01	GATA-2	GATA2	0.640878	119
V$CEBPA_Q6	C/EBPalpha	CEBPA	0.0393831	114
V$GR_01	GR	NR3C1	0.11708	113
V$TCF4_01	TCF-4	TCF7L2	0.463803	110
V$IRF7_Q3	IRF-7	IRF7	0.0608511	103
V$ESE1_Q3	ESE-1	ELF3	0.0781143	96
V$CREM_Q6	CREM	CREM	0.205777	91
V$GABPBETA_Q3	GABP-beta	GABPB1	0.196211	89
V$CEBPD_Q6	C/EBPdelta	CEBPD	0.414997	89
V$GATA3_03	GATA-3	GATA3	0.893577	88
V$CEBPB_02	C/EBPbeta	CEBPB	0.579904	86
V$CEBPG_Q6_01	C/EBPgamma	CEBPG	0.530121	79
V$CEBPB_Q6	C/EBPbeta	CEBPB	0.579904	71
V$YY1_02	YY1	YY1	0.375681	70
V$CEBPA_01	C/EBPalpha	CEBPA	0.0393831	67
V$ATF3_Q6_01	ATF-3	ATF3	0.59032	64
V$EAR2_Q2	EAR2	NR2F6	0.396424	60
V$CEBPG_Q6	C/EBPgamma	CEBPG	0.530121	45
V$CEBPB_01	C/EBPbeta	CEBPB	0.579904	44
V$IRF1_Q6	IRF-1	IRF1	0.253615	43
V$STAT1_Q6	STAT1	STAT1	0.0497363	37
V$E4BP4_01	E4BP4	NFIL3	0.166757	11

----

hsa-mir-338

Matrix_id	transcription factor	Gene	PCC	Occurrence
V$PUR1_Q4	PUR1	PURA	0.388227	425
V$MAFB_01	MAFB	MAFB	0.224377	392
V$PARP_Q4	PARP	PARP1	0.342928	387
V$ZIC3_01	Zic3	ZIC3	0.170904	385
V$ETS2_Q6	c-Ets-2	ETS2	0.245025	349
V$SOX9_Q4	SOX9	SOX9	0.244942	338
V$GABPA_Q4	GABP-alpha	GABPA	0.0724754	338
V$AP4_Q6_02	AP-4	TFAP4	0.402693	311
V$YY1_01	YY1	YY1	0.0511802	308
V$MEF2C_Q4	MEF-2C	MEF2C	0.745532	297
V$YY1_Q6	YY1	YY1	0.0511802	288
V$ETS2_B	c-Ets-2	ETS2	0.245025	286
V$YY1_Q6_02	YY1	YY1	0.0511802	284
V$SOX9_B1	SOX9	SOX9	0.244942	278
V$ZBP89_Q4	ZBP89	ZNF148	0.0634848	255
V$SRY_02	SRY	SRY	0.112372	253
V$PARP_Q3	PARP	PARP1	0.342928	251
V$PBX1_04	Pbx1	PBX1	0.212183	229
V$PBX1_Q3	Pbx1	PBX1	0.212183	221
V$YY1_Q6_03	YY1	YY1	0.0511802	220
V$AP4_Q5	AP-4	TFAP4	0.402693	216
V$AP4_Q6	AP-4	TFAP4	0.402693	197
V$SOX10_Q6	SOX10	SOX10	0.676265	175
V$CP2_01	CP2	TFCP2	0.193589	158
V$ZIC1_01	Zic1	ZIC1	0.915383	145
V$AP4_Q6_01	AP-4	TFAP4	0.402693	139
V$YY1_02	YY1	YY1	0.0511802	124
V$AP4_01	AP-4	TFAP4	0.402693	89
V$EAR2_Q2	EAR2	NR2F6	0.00168923	86
V$YY1_03	YY1	YY1	0.0511802	77

hsa-mir-340

Matrix_id	transcription factor	Gene	PCC	Occurrence
V$PUR1_Q4	PUR1	PURA	0.451883	544
V$MAFB_01	MAFB	MAFB	0.226668	498
V$ZIC3_01	Zic3	ZIC3	0.239744	494
V$PARP_Q4	PARP	PARP1	0.491561	493
V$P300_01	p300	EP300	0.047696	487

V$TBX5_02	TBX5	TBX5	0.00929668	452
V$CETS1_Q6	C-ets-1	ETS1	0.0932357	450
V$GATA1_01	GATA-1	GATA1	0.0720293	449
V$ETS2_Q6	c-Ets-2	ETS2	0.249327	446
V$ETS1_B	c-Ets-1	ETS1	0.0932357	442
V$NR1B2_Q6	NR1B2	RARB	0.144275	434
V$SOX9_Q4	SOX9	SOX9	0.260775	430
V$GABPA_Q4	GABP-alpha	GABPA	0.219896	429
V$SMAD3_Q6_01	Smad3	SMAD3	0.0235403	419
V$MAZ_Q6	MAZ	MAZ	0.367959	401
V$YY1_01	YY1	YY1	0.129669	399
V$BEN_01	BEN	GTF2IRD1	0.0651936	393
V$GR_Q6	GR	NR3C1	0.085777	390
V$LRF_Q2	LRF	ZBTB7A	0.0470768	383
V$HNF4A_Q6_01	HNF-4alpha	HNF4A	0.129145	376
V$AML1_Q6	AML1	RUNX1	0.00485028	376
V$YY1_Q6_02	YY1	YY1	0.129669	369
V$YY1_Q6	YY1	YY1	0.129669	368
V$ETS2_B	c-Ets-2	ETS2	0.249327	366
V$IPF1_Q6	IPF1	PDX1	0.00602966	357
V$TBX5_01	TBX5	TBX5	0.00929668	356
V$GATA1_06	GATA-1	GATA1	0.0720293	342
V$GATA1_05	GATA-1	GATA1	0.0720293	342
V$IPF1_Q4_01	IPF1	PDX1	0.00602966	341
V$ING4_01	ING4	ING4	0.745053	324
V$TEL1_02	TEL1	ETV6	0.00912305	306
V$ELK1_02	Elk-1	ELK1	0.252167	306
V$PBX1_04	Pbx1	PBX1	0.159625	291
V$PBX1_Q3	Pbx1	PBX1	0.159625	281
V$YY1_Q6_03	YY1	YY1	0.129669	272
V$ETV7_01	ETV7	ETV7	0.0313973	262
V$CNOT3_01	CNOT3	CNOT3	0.0348341	249
V$ERR1_Q3	ERR1	ESRRA	0.0638228	235
V$CREM_Q6	CREM	CREM	0.114089	231
V$GR_01	GR	NR3C1	0.085777	224
V$ZIC1_01	Zic1	ZIC1	0.938954	199
V$AML1_01	AML1a	RUNX1	0.00485028	197
V$OC2_Q3	OC-2	ONECUT2	0.0890272	184
V$AML1_Q4	AML1	RUNX1	0.00485028	174
V$FAC1_01	FAC1	BPTF	0.0645098	163
V$PIT1_Q6	Pit-1	POU1F1	0.119992	162
V$IPF1_02	IPF1	PDX1	0.00602966	162
V$DBP_Q6_01	DBP	DBP	0.325419	158
V$BEN_02	BEN	GTF2IRD1	0.0651936	130
V$GRE_C	GR	NR3C1	0.085777	32

----

hsa-mir-342

Matrix_id	transcription factor	Gene	PCC	Occurrence
V$IK_Q5	Ikaros	IKZF1	0.0278374	5
V$ELF1_Q6	Elf-1	ELF1	0.0285681	5
V$MYB_Q6	c-Myb	MYB	0.172515	4
V$GFI1_Q6_01	Gfi1	GFI1	0.191649	4
V$GFI1_Q6	Gfi1	GFI1	0.191649	4
V$SOX10_Q6	SOX10	SOX10	0.32917	4
V$CMYB_Q5	c-Myb	MYB	0.172515	4
V$MAFB_01	MAFB	MAFB	0.0534717	4
V$AML2_Q3	AML2	RUNX3	0.0658738	4
V$AML2_01	AML2	RUNX3	0.0658738	3
V$NFAT4_Q3	NF-AT4	NFATC3	0.0214746	3
V$ING4_01	ING4	ING4	0.561119	3
V$AML2_Q3_01	AML2	RUNX3	0.0658738	3
V$MAX_Q6	MAX	MAX	0.278294	2
V$MAX_01	Max	MAX	0.278294	2
V$USF2_Q6	USF2	USF2	0.0150535	2
V$LEF1_Q5	LEF-1	LEF1	0.379414	2

hsa-mir-345

Matrix_id	transcription factor	Gene	PCC	Occurrence
V$NFAT4_Q3	NF-AT4	NFATC3	0.116622	5
V$Elf5_03	ELF5	ELF5	0.223725	5
V$ZIC3_01	Zic3	ZIC3	0.378288	5
V$TBX5_02	TBX5	TBX5	0.3038	5
V$AP2REP_01	AP-2rep	KLF12	0.437791	5
V$PUR1_Q4	PUR1	PURA	0.442322	5
V$GABPA_Q4	GABP-alpha	GABPA	0.31736	5
V$PEA3_Q6	PEA3	ETV4	0.301631	5
V$CDX2_Q5_02	CDX-2	CDX2	0.4008	5
V$IK_Q5	Ikaros	IKZF1	0.309139	5
V$TBP_Q6	TBP	TBP	0.207674	5
V$CETS1_Q6	C-ets-1	ETS1	0.234633	5
V$P300_01	p300	EP300	0.383043	5
V$MAFB_01	MAFB	MAFB	0.0546553	5
V$SMAD4_Q6_01	Smad4	SMAD4	0.339399	5
V$GATA1_01	GATA-1	GATA1	0.29248	5

V$HIF1A_Q6	HIF-1alpha	HIF1A	0.466635	5
V$ARNT_01	Arnt	ARNT	0.171256	5
V$SOX10_Q6	SOX10	SOX10	0.602438	4
V$TBX5_01	TBX5	TBX5	0.3038	4
V$SOX9_Q4	SOX9	SOX9	0.446474	4
V$ETS2_Q6	c-Ets-2	ETS2	0.280203	4
V$HNF3A_01	HNF3A	FOXA1	0.369226	4
V$CDX2_01	Cdx-2	CDX2	0.4008	4
V$PARP_Q4	PARP	PARP1	0.488017	4
V$SPI1_Q5	SPI1	SPI1	0.0268809	4
V$PBX1_Q3	Pbx1	PBX1	0.268786	4
V$GR_01	GR	NR3C1	0.395673	4
V$HNF3B_Q6	HNF-3beta	FOXA2	0.26571	4
V$GATA1_05	GATA-1	GATA1	0.29248	4
V$GR_Q6	GR	NR3C1	0.395673	4
V$YY1_Q6_03	YY1	YY1	0.465057	4
V$ETS1_B	c-Ets-1	ETS1	0.234633	4
V$GATA1_06	GATA-1	GATA1	0.29248	4
V$NR1B2_Q6	NR1B2	RARB	0.472016	4
V$HNF4A_Q6_01	HNF-4alpha	HNF4A	0.284069	4
V$TEF1_Q6_03	TEF-1	TEAD1	0.415532	4
V$GATA6_01	GATA-6	GATA6	0.0634063	4
V$CDX2_Q5_01	Cdx-2	CDX2	0.4008	4
V$AML1_Q6	AML1	RUNX1	0.313813	4
V$FOXP3_01	FOXP3	FOXP3	0.39636	4
V$MEF2C_Q4	MEF-2C	MEF2C	0.536705	3
V$IPF1_Q6	IPF1	PDX1	0.450345	3
V$AML2_Q3	AML2	RUNX3	0.0108791	3
V$ERBETA_Q5	ER-beta	ESR2	0.336451	3
V$CDX1_01	Cdx-1	CDX1	0.362091	3
V$TTF1_Q5	TTF-1	NKX2-1	0.332642	3
V$NANOG_02	Nanog	NANOG	0.426255	3
V$CREL_01	c-Rel	REL	0.270234	3
V$GFI1_Q6	Gfi1	GFI1	0.0832091	3
V$CMAF_01	c-Maf	MAF	0.201735	3
V$MYB_Q6	c-Myb	MYB	0.100385	3
V$IPF1_Q4_01	IPF1	PDX1	0.450345	3
V$NKX32_01	Nkx3-2	NKX3-2	0.427066	3
V$MEIS1_01	MEIS1	MEIS1	0.0150208	3
V$CMYB_Q5	c-Myb	MYB	0.100385	3
V$TCF4_01	TCF-4	TCF7L2	0.00739518	2
V$CRX_Q4_01	CRX	CRX	0.367308	2
V$NFAT2_Q5	NF-AT2	NFATC1	0.422432	2
V$FOXM1_01	FOXM1	FOXM1	0.129656	2
V$RFX1_01	RFX1	RFX1	0.433161	2
V$HOXD9_Q2	Hoxd9	HOXD9	0.331713	2

V$IPF1_Q4	IPF1	PDX1	0.450345	2
V$RFX1_02	RFX1	RFX1	0.433161	2
V$TEF1_Q6	TEF-1	TEAD1	0.415532	2
V$NURR1_Q3	NURR1	NR4A2	0.577339	2
V$IPF1_01	IPF1	PDX1	0.450345	2
V$PARP_Q3	PARP	PARP1	0.488017	2
V$PBX1_04	Pbx1	PBX1	0.268786	2
V$FOXO3A_Q1	FOXO3A	FOXO3	0.27334	2
V$KAISO_01	KAISO	ZBTB33	0.379757	2
V$YY1_Q6_02	YY1	YY1	0.465057	2
V$YY1_Q6	YY1	YY1	0.465057	2
V$AML1_01	AML1a	RUNX1	0.313813	2
V$LHX3b_01	LHX3b	LHX3	0.478336	1
V$OC2_Q3	OC-2	ONECUT2	0.558597	1
V$MITF_Q6	MITF	MITF	0.212375	1
V$BCL6_Q3_01	Bcl-6	BCL6	0.404348	1
V$CMYB_01	c-Myb	MYB	0.100385	1
V$YY1_03	YY1	YY1	0.465057	1
V$STAT5A_Q6	STAT5A	STAT5A	0.0672547	1
V$TGIF_01	TGIF	TGIF1	0.0192112	1
V$HNF6_Q6	HNF6	ONECUT1	0.423477	1
V$IRF1_Q6	IRF-1	IRF1	0.0138021	1
V$PIT1_Q6	Pit-1	POU1F1	0.348329	1
V$IPF1_03	IPF1	PDX1	0.450345	1
V$PLZF_02	PLZF	ZBTB16	0.279435	1
V$CART1_02	CART1	ALX1	0.477732	1
V$HNF1_02	HNF-1alpha	HNF1A	0.287489	1
V$NCX_02	Ncx	TLX2	0.386285	1
V$HNF1B_01	HNF-1beta	HNF1B	0.21402	1
V$IPF1_06	ipf1	PDX1	0.450345	1
V$HOX13_02	HOXA5	HOXA5	0.276497	1
V$NKX32_02	Nkx3-2	NKX3-2	0.427066	1
V$IRF1_Q6_01	IRF-1	IRF1	0.0138021	1
V$YY1_02	YY1	YY1	0.465057	1

----

hsa-mir-346

Matrix_id	transcription factor	Gene	PCC	Occurrence
V$PUR1_Q4	PUR1	PURA	0.413645	468
V$MAFB_01	MAFB	MAFB	0.211012	431
V$PARP_Q4	PARP	PARP1	0.405642	430
V$YY1_01	YY1	YY1	0.163197	342
V$MEF2C_Q4	MEF-2C	MEF2C	0.79053	339

V$GR_Q6	GR	NR3C1	0.0673289	338
V$DLX5_01	dlx5	DLX5	0.00404639	320
V$YY1_Q6	YY1	YY1	0.163197	314
V$YY1_Q6_02	YY1	YY1	0.163197	308
V$SOX9_B1	SOX9	SOX9	0.297248	307
V$PARP_Q3	PARP	PARP1	0.405642	280
V$SRY_02	SRY	SRY	0.172584	280
V$ELK1_02	Elk-1	ELK1	0.105538	259
V$FOXO3A_Q1	FOXO3A	FOXO3	0.00097435	254
V$PITX3_Q2	PITX3	PITX3	0.0778311	253
V$PBX1_04	Pbx1	PBX1	0.305721	253
V$YY1_Q6_03	YY1	YY1	0.163197	241
V$ELK1_06	ELK-1	ELK1	0.105538	225
V$EHF_03	EHF	EHF	0.035893	205
V$ERF_02	ERF	ERF	0.177365	203
V$GR_01	GR	NR3C1	0.0673289	196
V$MEF2A_Q6	mef2A	MEF2A	0.677405	146
V$DBP_Q6_01	DBP	DBP	0.315027	145
V$CDP_04	CDP	CUX1	0.0481611	71
V$POU6F1_03	POU6F1	POU6F1	0.34266	65
V$AMEF2_Q6	aMEF-2	MEF2A	0.677405	52
V$RSRFC4_Q2	RSRFC4	MEF2A	0.677405	40
V$GRE_C	GR	NR3C1	0.0673289	29
V$MEF2A_05	MEF2A	MEF2A	0.677405	20

----

hsa-mir-34c

Matrix_id	transcription factor	Gene	PCC	Occurrence
V$AP2REP_01	AP-2rep	KLF12	0.0855397	244
V$GKLF_Q4	GKLF	KLF4	0.377182	240
V$PARP_Q4	PARP	PARP1	0.214071	238
V$PEA3_Q6	PEA3	ETV4	0.146561	237
V$P300_01	p300	EP300	0.0729686	236
V$TBP_Q6	TBP	TBP	0.725656	215
V$ZIC3_01	Zic3	ZIC3	0.230397	214
V$NFAT4_Q3	NF-AT4	NFATC3	0.0406524	205
V$GATA1_01	GATA-1	GATA1	0.108629	205
V$YY1_01	YY1	YY1	0.183	204
V$ETS1_B	c-Ets-1	ETS1	0.0547252	201
V$CETS1_Q6	C-ets-1	ETS1	0.0547252	195
V$DLX5_01	dlx5	DLX5	0.310251	189
V$Elf5_03	ELF5	ELF5	0.194918	184
V$SOX5_01	SOX5	SOX5	0.571417	181

V$SOX9_B1	SOX9	SOX9	0.758408	181
V$AML1_Q6	AML1	RUNX1	0.267972	180
V$SRY_02	SRY	SRY	0.21059	162
V$GATA1_02	GATA-1	GATA1	0.108629	162
V$AP4_Q6_02	AP-4	TFAP4	0.0875805	158
V$FOXO3A_Q1	FOXO3A	FOXO3	0.0565175	157
V$NKX32_01	Nkx3-2	NKX3-2	0.266625	154
V$MAZ_Q6	MAZ	MAZ	0.307648	152
V$E2A_Q6	E2A	TCF3	0.404414	152
V$E12_Q6	E12	TCF3	0.404414	152
V$ING4_01	ING4	ING4	0.0148851	152
V$E47_02	E47	TCF3	0.404414	152
V$ERBETA_Q5	ER-beta	ESR2	0.175775	151
V$HNF3B_Q6	HNF-3beta	FOXA2	0.0940334	146
V$HMGIY_01	HMGIY	HMGA1	0.636171	142
V$HIF1A_Q6	HIF-1alpha	HIF1A	0.077409	131
V$PITX1_01	Pitx1	PITX1	0.0198438	130
V$GATA1_04	GATA-1	GATA1	0.108629	128
V$FOXJ2_01	FOXJ2	FOXJ2	0.324129	126
V$NFAT2_Q5	NF-AT2	NFATC1	0.155941	125
V$FOXM1_01	FOXM1	FOXM1	0.721505	125
V$CREM_Q6	CREM	CREM	0.437026	123
V$MYOGENIN_Q6	myogenin	MYOG	0.160035	122
V$IRF8_Q6	IRF-8	IRF8	0.104782	114
V$AP4_Q5	AP-4	TFAP4	0.0875805	113
V$GABPBETA_Q3	GABP-beta	GABPB1	0.0873224	106
V$AML2_Q3	AML2	RUNX3	0.0941956	102
V$AML1_01	AML1a	RUNX1	0.267972	102
V$ELF5_01	ELF5	ELF5	0.194918	96
V$AML1_Q4	AML1	RUNX1	0.267972	92
V$FAC1_01	FAC1	BPTF	0.0279562	92
V$AP4_Q6	AP-4	TFAP4	0.0875805	90
V$E47_01	E47	TCF3	0.404414	88
V$FOXO4_02	FOXO4	FOXO4	0.0216649	81
V$HOXD9_Q2	Hoxd9	HOXD9	0.0458822	79
V$MYOGENIN_Q6_01	myogenin	MYOG	0.160035	78
V$LHX3b_01	LHX3b	LHX3	0.114691	74
V$TR4_Q2	TR4	NR2C2	0.0850578	65
V$AML2_01	AML2	RUNX3	0.0941956	60
V$P53_02	p53	TP53	0.0693211	58
V$MATH1_Q2	MATH1	ATOH1	0.133533	53
V$HOXB8_01	HOXB8	HOXB8	0.344733	48
V$ATF2_Q5	ATF-2	ATF2	0.0742922	42
V$ATF6_01	ATF6	ATF6	0.182334	39
V$FOXO4_01	FOXO4	FOXO4	0.0216649	36
V$GCNF_Q3	GCNF	NR6A1	0.5227	21

V$P53_04	p53	TP53	0.0693211	19
V$SRF_C	SRF	SRF	0.0148034	14
V$SRF_Q6	SRF	SRF	0.0148034	10
V$SRF_Q5_02	SRF	SRF	0.0148034	10
V$P53_05	p53	TP53	0.0693211	1

---

hsa-mir-361

Matrix_id	transcription factor	Gene	PCC	Occurrence
V$PARP_Q4	PARP	PARP1	0.242741	3
V$PUR1_Q4	PUR1	PURA	0.575586	3
V$DLX5_01	dlx5	DLX5	0.433865	3
V$HBP1_Q2	hbp1	HBP1	0.338652	3
V$YY1_Q6_03	YY1	YY1	0.319166	3
V$FOXO3A_Q1	FOXO3A	FOXO3	0.0996141	3
V$NURR1_Q3	NURR1	NR4A2	0.29215	2
V$PARP_Q3	PARP	PARP1	0.242741	2
V$HOXA9_01	hoxa9	HOXA9	0.190704	2
V$ETS2_Q6	c-Ets-2	ETS2	0.105998	2
V$PITX2_01	PITX2	PITX2	0.20834	2
V$GABPA_Q4	GABP-alpha	GABPA	0.153389	2
V$SOX9_Q4	SOX9	SOX9	0.0310897	2
V$TCF4_01	TCF-4	TCF7L2	0.251462	2
V$MEF2C_Q4	MEF-2C	MEF2C	0.424149	2
V$HNF4A_Q6_01	HNF-4alpha	HNF4A	0.0391071	2
V$LHX3b_01	LHX3b	LHX3	0.0656073	2
V$SMAD4_Q6_01	Smad4	SMAD4	0.239438	2
V$AP4_Q6_02	AP-4	TFAP4	0.264997	2
V$HOXD9_Q2	Hoxd9	HOXD9	0.0803202	2
V$OC2_Q3	OC-2	ONECUT2	0.00735986	2
V$PITX3_Q2	PITX3	PITX3	0.174056	2
V$SOX10_Q6	SOX10	SOX10	0.272868	2
V$TBP_Q6	TBP	TBP	0.116391	2
V$GATA3_03	GATA-3	GATA3	0.335796	2
V$GATA2_02	GATA-2	GATA2	0.394243	2
V$SOX9_B1	SOX9	SOX9	0.0310897	2
V$GATA6_01	GATA-6	GATA6	0.227973	2
V$PITX2_Q2	Pitx2	PITX2	0.20834	2
V$SRY_02	SRY	SRY	0.179844	2
V$TCF4_Q5	TCF-4	TCF7L2	0.251462	2
V$TEF1_Q6	TEF-1	TEAD1	0.0138214	2
V$YY1_01	YY1	YY1	0.319166	2
V$P300_01	p300	EP300	0.163468	2

V$YY1_Q6	YY1	YY1	0.319166	2
V$PBX1_04	Pbx1	PBX1	0.577604	2
V$CDP_02	CDP	CUX1	0.46026	1
V$YY1_03	YY1	YY1	0.319166	1
V$STAT5A_Q6	STAT5A	STAT5A	0.292727	1
V$CDP_01	CDP	CUX1	0.46026	1
V$GATA3_01	GATA-3	GATA3	0.335796	1
V$PBX1_Q3	Pbx1	PBX1	0.577604	1
V$GATA2_01	GATA-2	GATA2	0.394243	1
V$AP4_Q5	AP-4	TFAP4	0.264997	1
V$AP4_Q6	AP-4	TFAP4	0.264997	1
V$GR_01	GR	NR3C1	0.276212	1
V$PIT1_Q6	Pit-1	POU1F1	0.0624736	1
V$CIZ_01	CIZ	ZNF384	0.172328	1
V$SOX2_Q6	SOX2	SOX2	0.157375	1
V$AP4_Q6_01	AP-4	TFAP4	0.264997	1
V$HOX13_02	HOXA5	HOXA5	0.314794	1
V$FOXJ2_01	FOXJ2	FOXJ2	0.114989	1
V$YY1_Q6_02	YY1	YY1	0.319166	1
V$GR_Q6	GR	NR3C1	0.276212	1
V$TEF1_Q6_03	TEF-1	TEAD1	0.0138214	1

----

hsa-mir-362

Matrix_id	transcription factor	Gene	PCC	Occurrence
V$ZIC3_01	Zic3	ZIC3	0.190077	15
V$AP2REP_01	AP-2rep	KLF12	0.216454	15
V$IK_Q5	Ikaros	IKZF1	0.174526	15
V$NFAT4_Q3	NF-AT4	NFATC3	0.129991	15
V$PUR1_Q4	PUR1	PURA	0.098406	15
V$GKLF_Q4	GKLF	KLF4	0.228678	14
V$P300_01	p300	EP300	0.506506	14
V$PARP_Q4	PARP	PARP1	0.189432	14
V$TBP_Q6	TBP	TBP	0.295682	13
V$CDX2_Q5_02	CDX-2	CDX2	0.290073	13
V$IPF1_Q6	IPF1	PDX1	0.292057	13
V$SMAD4_Q6_01	Smad4	SMAD4	0.414288	13
V$NKX32_01	Nkx3-2	NKX3-2	0.232451	13
V$IPF1_Q4_01	IPF1	PDX1	0.292057	12
V$TBX5_02	TBX5	TBX5	0.289095	12
V$NFAT2_Q5	NF-AT2	NFATC1	0.201332	12
V$YY1_01	YY1	YY1	0.225514	12
V$SOX5_01	SOX5	SOX5	0.195692	12

V$SRY_02	SRY	SRY	0.124663	12
V$NANOG_02	Nanog	NANOG	0.26821	11
V$NR1B2_Q6	NR1B2	RARB	0.213143	11
V$CRX_Q4_01	CRX	CRX	0.284592	11
V$SMAD3_Q6_01	Smad3	SMAD3	0.183753	11
V$CDX2_Q5_01	Cdx-2	CDX2	0.290073	11
V$BEN_01	BEN	GTF2IRD1	0.241581	11
V$AML1_Q6	AML1	RUNX1	0.112051	10
V$GATA3_02	GATA-3	GATA3	0.698433	10
V$HNF3A_01	HNF3A	FOXA1	0.0273748	10
V$TBX5_01	TBX5	TBX5	0.289095	10
V$IPF1_Q4	IPF1	PDX1	0.292057	10
V$GR_Q6	GR	NR3C1	0.274419	10
V$AP2ALPHA_01	AP-2alpha	TFAP2A	0.762469	10
V$GATA2_02	GATA-2	GATA2	0.676145	10
V$YY1_Q6_02	YY1	YY1	0.225514	9
V$DLX5_01	dlx5	DLX5	0.666326	9
V$HNF4A_Q6_01	HNF-4alpha	HNF4A	0.0166495	9
V$HMGIY_01	HMGIY	HMGA1	0.170144	9
V$CRX_Q4	Crx	CRX	0.284592	9
V$PARP_Q3	PARP	PARP1	0.189432	9
V$YY1_Q6	YY1	YY1	0.225514	9
V$IPF1_01	IPF1	PDX1	0.292057	9
V$PITX3_Q2	PITX3	PITX3	0.154201	9
V$YY1_Q6_03	YY1	YY1	0.225514	9
V$CDX2_01	Cdx-2	CDX2	0.290073	8
V$ZBP89_Q4	ZBP89	ZNF148	0.0269183	8
V$NKX32_02	Nkx3-2	NKX3-2	0.232451	8
V$IRF8_Q6	IRF-8	IRF8	0.0550092	8
V$AHR_Q5	AhR	AHR	0.61451	8
V$SP1_Q6	Sp1	SP1	0.505314	8
V$TEF1_Q6_03	TEF-1	TEAD1	0.348281	8
V$FOXJ2_01	FOXJ2	FOXJ2	0.257759	8
V$LHX3b_01	LHX3b	LHX3	0.211605	7
V$HIF1A_Q6	HIF-1alpha	HIF1A	0.0177167	7
V$PITX2_01	PITX2	PITX2	0.610552	7
V$BCL6_Q3_01	Bcl-6	BCL6	0.240916	7
V$SP1_02	SP1	SP1	0.505314	7
V$SP1_Q2_01	Sp1	SP1	0.505314	7
V$SP1_Q4_01	Sp1	SP1	0.505314	7
V$SP1_Q6_01	Sp1	SP1	0.505314	7
V$ARNT_01	Arnt	ARNT	0.541924	7
V$CEBPB_02	C/EBPbeta	CEBPB	0.410043	7
V$RFX1_02	RFX1	RFX1	0.276688	7
V$GATA3_01	GATA-3	GATA3	0.698433	7
V$SREBP1_Q6	SREBP-1	SREBF1	0.0735677	7

V$GATA2_01	GATA-2	GATA2	0.676145	7
V$TCF4_Q5	TCF-4	TCF7L2	0.171632	7
V$PITX2_Q2	Pitx2	PITX2	0.610552	7
V$TEF1_Q6	TEF-1	TEAD1	0.348281	6
V$AP2GAMMA_01	AP-2gamma	TFAP2C	0.716064	6
V$TCF4_01	TCF-4	TCF7L2	0.171632	6
V$IRF7_Q3	IRF-7	IRF7	0.00306403	6
V$ZABC1_01	ZABC1	ZNF217	0.645854	6
V$PITX1_01	Pitx1	PITX1	0.226697	6
V$WT1_Q6_01	WT1	WT1	0.135075	6
V$POU6F1_03	POU6F1	POU6F1	0.153532	6
V$CRX_02	Crx	CRX	0.284592	6
V$CDP_04	CDP	CUX1	0.169744	5
V$FOXO4_02	FOXO4	FOXO4	0.720096	5
V$NCX_02	Ncx	TLX2	0.112141	5
V$SP2_01	SP2	SP2	0.145214	5
V$CEBPG_Q6_01	C/EBPgamma	CEBPG	0.286787	5
V$SMAD3_Q6	SMAD3	SMAD3	0.183753	5
V$POU6F1_02	POU6F1	POU6F1	0.153532	5
V$CP2_01	CP2	TFCP2	0.440291	4
V$STAT4_Q5	STAT4	STAT4	0.252212	4
V$YY1_02	YY1	YY1	0.225514	4
V$HOXA9_01	hoxa9	HOXA9	0.395195	4
V$CART1_02	CART1	ALX1	0.319631	4
V$CDX1_01	Cdx-1	CDX1	0.147711	4
V$GATA3_03	GATA-3	GATA3	0.698433	4
V$IPF1_02	IPF1	PDX1	0.292057	4
V$E2F1_Q3_01	E2F-1	E2F1	0.0255613	4
V$HOX13_02	HOXA5	HOXA5	0.0618025	4
V$IPF1_06	ipf1	PDX1	0.292057	4
V$STAT1_05	STAT1	STAT1	0.0398253	3
V$IPF1_03	IPF1	PDX1	0.292057	3
V$GATA2_03	GATA-2	GATA2	0.676145	3
V$HOXD9_Q2	Hoxd9	HOXD9	0.154698	3
V$NRF1_Q6	NRF-1	NRF1	0.323133	2
V$BACH2_01	Bach2	BACH2	0.210081	2
V$YY1_03	YY1	YY1	0.225514	1

----

hsa-mir-365a

Matrix_id	transcription factor	Gene	PCC	Occurrence
V$ETS1_B	c-Ets-1	ETS1	0.130054	4
V$NKX32_01	Nkx3-2	NKX3-2	0.432754	4

V$ETS2_B	c-Ets-2	ETS2	0.326752	4
V$SP2_01	SP2	SP2	0.267868	4
V$LRF_Q2	LRF	ZBTB7A	0.466183	4
V$ZIC3_01	Zic3	ZIC3	0.0559569	4
V$AP2REP_01	AP-2rep	KLF12	0.548359	4
V$AP2ALPHA_01	AP-2alpha	TFAP2A	0.461993	4
V$TBX5_02	TBX5	TBX5	0.479463	4
V$TBX5_01	TBX5	TBX5	0.479463	4
V$MAZ_Q6	MAZ	MAZ	0.0169194	4
V$PEA3_Q6	PEA3	ETV4	0.583692	4
V$SP1_Q2_01	Sp1	SP1	0.622868	4
V$SP1_Q4_01	Sp1	SP1	0.622868	4
V$SP1_Q6_01	Sp1	SP1	0.622868	4
V$CETS1_Q6	C-ets-1	ETS1	0.130054	4
V$AP2BETA_Q3	AP-2beta	TFAP2B	0.585111	4
V$ETS2_Q6	c-Ets-2	ETS2	0.326752	4
V$PUR1_Q4	PUR1	PURA	0.224183	4
V$SP1_01	Sp1	SP1	0.622868	4
V$GABPA_Q4	GABP-alpha	GABPA	0.410494	4
V$GATA1_01	GATA-1	GATA1	0.0617652	4
V$SP1_02	SP1	SP1	0.622868	4
V$AML1_Q6	AML1	RUNX1	0.264994	4
V$SP1_Q6	Sp1	SP1	0.622868	4
V$IK_Q5	Ikaros	IKZF1	0.506018	4
V$BEN_01	BEN	GTF2IRD1	0.565783	4
V$FOXP3_01	FOXP3	FOXP3	0.595191	3
V$AHR_Q5	AhR	AHR	0.254826	3
V$SOX10_Q6	SOX10	SOX10	0.0986482	3
V$YY1_Q6_02	YY1	YY1	0.225564	3
V$FKLF_Q5	FKLF	KLF11	0.0593576	3
V$ZBP89_Q4	ZBP89	ZNF148	0.369246	3
V$KLF15_Q2	KLF15	KLF15	0.0293971	3
V$E2F1_Q3_01	E2F-1	E2F1	0.270359	3
V$AP2ALPHA_02	AP-2alphaA	TFAP2A	0.461993	3
V$E2A_Q6	E2A	TCF3	0.323156	3
V$TBP_Q6	TBP	TBP	0.148473	3
V$NANOG_02	Nanog	NANOG	0.507456	3
V$AP2ALPHA_Q6	AP-2alpha	TFAP2A	0.461993	3
V$PDEF_02	PDEF	SPDEF	0.00844804	3
V$ELK1_06	ELK-1	ELK1	0.507991	3
V$GATA2_01	GATA-2	GATA2	0.466187	3
V$ER71_02	ER71	ETV2	0.294604	3
V$FOXJ2_01	FOXJ2	FOXJ2	0.481721	3
V$GATA3_01	GATA-3	GATA3	0.314489	3
V$CMYB_01	c-Myb	MYB	0.0386501	3
V$ETV3_02	ETV3	ETV3	0.338687	3

V$TEL1_02	TEL1	ETV6	0.562062	3
V$AP2GAMMA_01	AP-2gamma	TFAP2C	0.497461	3
V$PARP_Q4	PARP	PARP1	0.457459	3
V$CP2_01	CP2	TFCP2	0.425321	3
V$SMAD4_Q6_01	Smad4	SMAD4	0.391335	3
V$ELK1_02	Elk-1	ELK1	0.507991	3
V$E12_Q6	E12	TCF3	0.323156	3
V$HNF3B_Q6	HNF-3beta	FOXA2	0.19021	3
V$CDX2_Q5_02	CDX-2	CDX2	0.587654	3
V$P300_01	p300	EP300	0.535778	3
V$MAZ_Q6_01	MAZ	MAZ	0.0169194	3
V$SMAD3_Q6_01	Smad3	SMAD3	0.379041	3
V$SPIB_03	Spi-B	SPIB	0.192214	2
V$CREM_Q6	CREM	CREM	0.462824	2
V$TEF1_Q6_03	TEF-1	TEAD1	0.611416	2
V$EKLF_Q5	EKLF	KLF1	0.603123	2
V$ETV7_01	ETV7	ETV7	0.581179	2
V$GABPBETA_Q3	GABP-beta	GABPB1	0.370293	2
V$EGR1_02	EGR-1	EGR1	0.0610934	2
V$MYOGENIN_Q6_01	myogenin	MYOG	0.44021	2
V$MEF2C_Q4	MEF-2C	MEF2C	0.213937	2
V$ERF_02	ERF	ERF	0.112243	2
V$ATF3_Q6_01	ATF-3	ATF3	0.25106	2
V$ATF1_Q6_01	ATF-1	ATF1	0.128005	2
V$AP4_Q6_02	AP-4	TFAP4	0.126427	2
V$ERM_02	Erm	ETV5	0.473625	2
V$MYOD_Q6_02	MyoD	MYOD1	0.59367	2
V$SP4_Q5	SP4	SP4	0.434674	2
V$SREBP1_Q6	SREBP-1	SREBF1	0.335835	2
V$CACCCBINDINGFACT	CACCC-binding factor	ZNF148	0.369246	2
V$MYOGENIN_Q6	myogenin	MYOG	0.44021	2
V$TEF1_Q6	TEF-1	TEAD1	0.611416	2
V$AP4_Q6	AP-4	TFAP4	0.126427	2
V$MYOD_Q6	MyoD	MYOD1	0.59367	2
V$MTF1_Q4	MTF-1	MTF1	0.59351	2
V$ATF6_01	ATF6	ATF6	0.583517	2
V$AP4_Q5	AP-4	TFAP4	0.126427	2
V$E2F1_Q4	E2F-1	E2F1	0.270359	2
V$YY1_Q6	YY1	YY1	0.225564	2
V$VDR_Q3	VDR	VDR	0.567454	2
V$MATH1_Q2	MATH1	ATOH1	0.476702	2
V$STAT3_03	STAT3	STAT3	0.382854	2
V$CDX2_01	Cdx-2	CDX2	0.587654	2
V$SMAD3_Q6	SMAD3	SMAD3	0.379041	2
V$CNOT3_01	CNOT3	CNOT3	0.475232	2
V$GR_Q6	GR	NR3C1	0.377163	2

V$CEBPA_01	C/EBPalpha	CEBPA	0.0294349	2
V$AP4_01	AP-4	TFAP4	0.126427	1
V$PET1_02	Pet-1	FEV	0.389144	1
V$ELK1_01	Elk-1	ELK1	0.507991	1
V$CEBPA_Q6	C/EBPalpha	CEBPA	0.0294349	1
V$BCL6_Q3_01	Bcl-6	BCL6	0.543886	1
V$RREB1_01	RREB-1	RREB1	0.515728	1
V$FLI1_02	Fli-1	FLI1	0.455928	1
V$STAT1_01	STAT1	STAT1	0.271881	1
V$ERG_03	ERG	ERG	0.604714	1
V$FAC1_01	FAC1	BPTF	0.0638982	1
V$AR_04	AR	AR	0.111623	1
V$SAP1A_01	SAP-1a	ELK4	0.608952	1
V$ATF2_Q5	ATF-2	ATF2	0.503795	1
V$CMAF_01	c-Maf	MAF	0.19451	1
V$ATF4_Q6	ATF-4	ATF4	0.655392	1
V$AR_03	AR	AR	0.111623	1
V$IRF4_Q6	IRF-4	IRF4	0.529476	1
V$STAT5A_Q6	STAT5A	STAT5A	0.586201	1
V$ERG_01	ERG	ERG	0.604714	1
V$AR_01	AR	AR	0.111623	1
V$FOXO4_02	FOXO4	FOXO4	0.413191	1
V$STAT1_Q6	STAT1	STAT1	0.271881	1

----

hsa-mir-369

Matrix_id	transcription factor	Gene	PCC	Occurrence
V$MAFB_01	MAFB	MAFB	0.139994	21
V$GKLF_Q4	GKLF	KLF4	0.329988	21
V$PUR1_Q4	PUR1	PURA	0.0296301	21
V$P300_01	p300	EP300	0.187664	21
V$PARP_Q4	PARP	PARP1	0.0865865	20
V$PEA3_Q6	PEA3	ETV4	0.105346	20
V$ELF1_Q6	Elf-1	ELF1	0.0439128	19
V$SMAD4_Q6_01	Smad4	SMAD4	0.116426	17
V$GABPA_Q4	GABP-alpha	GABPA	0.243576	16
V$GR_Q6	GR	NR3C1	0.0219837	15
V$HNF4A_Q6_01	HNF-4alpha	HNF4A	0.0207914	14
V$AP2ALPHA_Q6	AP-2alpha	TFAP2A	0.601	14
V$YY1_Q6	YY1	YY1	0.29772	14
V$GATA3_01	GATA-3	GATA3	0.567555	14
V$AHR_Q5	AhR	AHR	0.568969	14
V$YY1_01	YY1	YY1	0.29772	13

V$YY1_Q6_02	YY1	YY1	0.29772	13
V$GATA2_01	GATA-2	GATA2	0.370963	13
V$PBX1_04	Pbx1	PBX1	0.133262	11
V$SMAD4_Q6	SMAD4	SMAD4	0.116426	10
V$FOXP3_01	FOXP3	FOXP3	0.0270266	10
V$ATF3_Q6_01	ATF-3	ATF3	0.285526	9
V$CREM_Q6	CREM	CREM	0.503532	9
V$GR_01	GR	NR3C1	0.0219837	9
V$PIT1_Q6	Pit-1	POU1F1	0.174978	8
V$YY1_02	YY1	YY1	0.29772	8
V$GABPBETA_Q3	GABP-beta	GABPB1	0.167411	7
V$MAFK_Q3	MafK	MAFK	0.35298	6
V$ATF4_Q6	ATF-4	ATF4	0.0582351	6
V$OC2_Q3	OC-2	ONECUT2	0.0804045	6
V$CEBPB_02	C/EBPbeta	CEBPB	0.453468	5
V$ELK1_01	Elk-1	ELK1	0.196143	5
V$TCF4_01	TCF-4	TCF7L2	0.231605	4

hsa-mir-370

Matrix_id	transcription factor	Gene	PCC	Occurrence
V$PUR1_Q4	PUR1	PURA	0.156473	34
V$MAFB_01	MAFB	MAFB	0.0712211	33
V$PARP_Q4	PARP	PARP1	0.295115	32
V$TBP_Q6	TBP	TBP	0.10359	30
V$ZIC3_01	Zic3	ZIC3	0.00991243	30
V$NR1B2_Q6	NR1B2	RARB	0.0183904	28
V$YY1_Q6	YY1	YY1	0.221147	25
V$MAZ_Q6	MAZ	MAZ	0.121773	25
V$SOX9_Q4	SOX9	SOX9	0.0549181	25
V$AP4_Q6_02	AP-4	TFAP4	0.101997	25
V$GABPA_Q4	GABP-alpha	GABPA	0.170194	24
V$YY1_Q6_02	YY1	YY1	0.221147	24
V$YY1_Q6_03	YY1	YY1	0.221147	23
V$HNF4A_Q6_01	HNF-4alpha	HNF4A	0.136147	22
V$FKLF_Q5	FKLF	KLF11	0.0719132	21
V$BEN_01	BEN	GTF2IRD1	0.0089106	20
V$ING4_01	ING4	ING4	0.267248	20
V$AP2ALPHA_Q6	AP-2alpha	TFAP2A	0.129315	18
V$AHR_Q5	AhR	AHR	0.0957825	17
V$AP2ALPHA_01	AP-2alpha	TFAP2A	0.129315	17
V$KLF15_Q2	KLF15	KLF15	0.12742	16
V$MAZ_Q6_01	MAZ	MAZ	0.121773	16

V$EGR1_02	EGR-1	EGR1	0.285039	14
V$SREBP1_Q6	SREBP-1	SREBF1	0.781713	14
V$HIF1A_Q6	HIF-1alpha	HIF1A	0.593717	13
V$FAC1_01	FAC1	BPTF	0.0047734	13
V$GABPBETA_Q3	GABP-beta	GABPB1	0.0692354	12
V$AP2GAMMA_01	AP-2gamma	TFAP2C	0.0730365	10
V$CP2_01	CP2	TFCP2	0.432997	10
V$HBP1_Q2	hbp1	HBP1	0.129361	10
V$YY1_02	YY1	YY1	0.221147	10
V$BEN_02	BEN	GTF2IRD1	0.0089106	9
V$EAR2_Q2	EAR2	NR2F6	0.165698	8
V$RNF96_01	RNF96	TRIM28	0.0163921	8
V$MAX_01	Max	MAX	0.320721	6
V$YY1_03	YY1	YY1	0.221147	6
V$NMYC_01	N-Myc	MYCN	0.0416571	6
V$OC2_Q3	OC-2	ONECUT2	0.115398	6
V$USF2_Q6	USF2	USF2	0.217458	6
V$CMYC_02	c-Myc	MYC	0.070049	6
V$CMYC_01	c-Myc	MYC	0.070049	5
V$MAX_Q6	MAX	MAX	0.320721	5
V$E2F1_Q4	E2F-1	E2F1	0.231783	4
V$ELK1_01	Elk-1	ELK1	0.298056	4
V$ZID_01	ZID	ZBTB6	0.182808	2

----

hsa-mir-371a

Matrix_id	transcription factor	Gene	PCC	Occurrence
V$PUR1_Q4	PUR1	PURA	0.16005	263
V$PEA3_Q6	PEA3	ETV4	0.0983111	257
V$PARP_Q4	PARP	PARP1	0.0995149	254
V$ELF1_Q6	Elf-1	ELF1	0.165773	249
V$GKLF_Q4	GKLF	KLF4	0.567492	246
V$MAFB_01	MAFB	MAFB	0.300398	241
V$P300_01	p300	EP300	0.349573	236
V$ETS1_B	c-Ets-1	ETS1	0.0679363	230
V$CETS1_Q6	C-ets-1	ETS1	0.0679363	224
V$SMAD4_Q6_01	Smad4	SMAD4	0.308843	223
V$GATA1_01	GATA-1	GATA1	0.0170848	213
V$GABPA_Q4	GABP-alpha	GABPA	0.364776	212
V$TBP_Q6	TBP	TBP	0.156746	202
V$YY1_01	YY1	YY1	0.367543	197
V$DLX5_01	dlx5	DLX5	0.691609	178
V$GATA1_02	GATA-1	GATA1	0.0170848	173

V$GATA1_06	GATA-1	GATA1	0.0170848	165
V$GATA2_02	GATA-2	GATA2	0.695429	165
V$GATA1_05	GATA-1	GATA1	0.0170848	165
V$ING4_01	ING4	ING4	0.116608	160
V$PARP_Q3	PARP	PARP1	0.0995149	159
V$AP2ALPHA_01	AP-2alpha	TFAP2A	0.959118	143
V$GATA3_02	GATA-3	GATA3	0.912183	141
V$GATA3_01	GATA-3	GATA3	0.912183	139
V$YY1_Q6_03	YY1	YY1	0.367543	132
V$GATA2_01	GATA-2	GATA2	0.695429	126
V$TCF4_01	TCF-4	TCF7L2	0.46753	118
V$CEBPA_Q6	C/EBPalpha	CEBPA	0.0713893	117
V$PITX2_Q2	Pitx2	PITX2	0.430072	111
V$FOXJ2_01	FOXJ2	FOXJ2	0.0461565	106
V$PITX2_01	PITX2	PITX2	0.430072	105
V$AP2GAMMA_01	AP-2gamma	TFAP2C	0.872738	104
V$ZABC1_01	ZABC1	ZNF217	0.865372	103
V$CEBPD_Q6	C/EBPdelta	CEBPD	0.438292	98
V$HBP1_Q2	hbp1	HBP1	0.668623	90
V$FAC1_01	FAC1	BPTF	0.0676199	90
V$CEBPB_02	C/EBPbeta	CEBPB	0.597165	88
V$GATA2_03	GATA-2	GATA2	0.695429	77
V$FOXO4_02	FOXO4	FOXO4	0.956047	75
V$E2F4_Q6	E2F-4	E2F4	0.0663263	65
V$CIZ_01	CIZ	ZNF384	0.0830076	56
V$STAT1_05	STAT1	STAT1	0.0593173	49
V$IRF2_01	IRF-2	IRF2	0.072088	17

----

hsa-mir-372

Matrix_id	transcription factor	Gene	PCC	Occurrence
V$PUR1_Q4	PUR1	PURA	0.151521	265
V$PEA3_Q6	PEA3	ETV4	0.102889	259
V$PARP_Q4	PARP	PARP1	0.1081	256
V$ELF1_Q6	Elf-1	ELF1	0.160558	251
V$GKLF_Q4	GKLF	KLF4	0.574165	247
V$MAFB_01	MAFB	MAFB	0.289796	242
V$P300_01	p300	EP300	0.352733	237
V$ETS1_B	c-Ets-1	ETS1	0.0751595	232
V$CETS1_Q6	C-ets-1	ETS1	0.0751595	226
V$SMAD4_Q6_01	Smad4	SMAD4	0.308667	225
V$GATA1_01	GATA-1	GATA1	0.0240919	214
V$GABPA_Q4	GABP-alpha	GABPA	0.366507	214

V$TBP_Q6	TBP	TBP	0.19111	205
V$YY1_01	YY1	YY1	0.37484	199
V$DLX5_01	dlx5	DLX5	0.705134	180
V$GATA1_02	GATA-1	GATA1	0.0240919	176
V$GATA1_06	GATA-1	GATA1	0.0240919	168
V$GATA2_02	GATA-2	GATA2	0.692862	168
V$GATA1_05	GATA-1	GATA1	0.0240919	168
V$ING4_01	ING4	ING4	0.121	160
V$PARP_Q3	PARP	PARP1	0.1081	159
V$AP2ALPHA_01	AP-2alpha	TFAP2A	0.958228	144
V$GATA3_02	GATA-3	GATA3	0.909859	144
V$GATA3_01	GATA-3	GATA3	0.909859	139
V$YY1_Q6_03	YY1	YY1	0.37484	133
V$GATA2_01	GATA-2	GATA2	0.692862	126
V$TCF4_01	TCF-4	TCF7L2	0.470797	120
V$CEBPA_Q6	C/EBPalpha	CEBPA	0.0679616	117
V$PITX2_Q2	Pitx2	PITX2	0.42604	111
V$FOXJ2_01	FOXJ2	FOXJ2	0.0577009	107
V$AP2GAMMA_01	AP-2gamma	TFAP2C	0.87051	105
V$PITX2_01	PITX2	PITX2	0.42604	104
V$ZABC1_01	ZABC1	ZNF217	0.872271	103
V$CEBPD_Q6	C/EBPdelta	CEBPD	0.428503	98
V$HBP1_Q2	hbp1	HBP1	0.662874	90
V$FAC1_01	FAC1	BPTF	0.066566	90
V$CEBPB_02	C/EBPbeta	CEBPB	0.589416	88
V$GATA2_03	GATA-2	GATA2	0.692862	78
V$FOXO4_02	FOXO4	FOXO4	0.957266	77
V$E2F4_Q6	E2F-4	E2F4	0.0760092	65
V$CIZ_01	CIZ	ZNF384	0.088232	56
V$STAT1_05	STAT1	STAT1	0.0495436	50
V$IRF2_01	IRF-2	IRF2	0.0618566	17

hsa-mir-373

Matrix_id	transcription factor	Gene	PCC	Occurrence
V$PUR1_Q4	PUR1	PURA	0.145456	266
V$PEA3_Q6	PEA3	ETV4	0.105956	260
V$PARP_Q4	PARP	PARP1	0.113937	257
V$ELF1_Q6	Elf-1	ELF1	0.156781	252
V$GKLF_Q4	GKLF	KLF4	0.5782	248
V$MAFB_01	MAFB	MAFB	0.282154	243
V$P300_01	p300	EP300	0.354561	238
V$ETS1_B	c-Ets-1	ETS1	0.0800871	233

V$CETS1_Q6	C-ets-1	ETS1	0.0800871	228
V$SMAD4_Q6_01	Smad4	SMAD4	0.308229	226
V$GABPA_Q4	GABP-alpha	GABPA	0.36733	216
V$GATA1_01	GATA-1	GATA1	0.0289222	216
V$TBP_Q6	TBP	TBP	0.214725	206
V$YY1_01	YY1	YY1	0.379512	199
V$DLX5_01	dlx5	DLX5	0.713783	182
V$GATA1_02	GATA-1	GATA1	0.0289222	177
V$GATA1_05	GATA-1	GATA1	0.0289222	168
V$GATA2_02	GATA-2	GATA2	0.690373	168
V$GATA1_06	GATA-1	GATA1	0.0289222	168
V$ING4_01	ING4	ING4	0.12392	160
V$PARP_Q3	PARP	PARP1	0.113937	159
V$GATA3_02	GATA-3	GATA3	0.907316	144
V$AP2ALPHA_01	AP-2alpha	TFAP2A	0.95663	143
V$GATA3_01	GATA-3	GATA3	0.907316	139
V$YY1_Q6_03	YY1	YY1	0.379512	134
V$GATA2_01	GATA-2	GATA2	0.690373	126
V$TCF4_01	TCF-4	TCF7L2	0.472578	121
V$CEBPA_Q6	C/EBPalpha	CEBPA	0.0655171	119
V$PITX2_Q2	Pitx2	PITX2	0.422809	112
V$FOXJ2_01	FOXJ2	FOXJ2	0.0656405	109
V$PITX2_01	PITX2	PITX2	0.422809	105
V$ZABC1_01	ZABC1	ZNF217	0.876158	104
V$AP2GAMMA_01	AP-2gamma	TFAP2C	0.868073	104
V$CEBPD_Q6	C/EBPdelta	CEBPD	0.421282	99
V$HBP1_Q2	hbp1	HBP1	0.658212	90
V$FAC1_01	FAC1	BPTF	0.0657675	90
V$CEBPB_02	C/EBPbeta	CEBPB	0.583443	88
V$GATA2_03	GATA-2	GATA2	0.690373	78
V$FOXO4_02	FOXO4	FOXO4	0.957129	77
V$E2F4_Q6	E2F-4	E2F4	0.0826402	65
V$CIZ_01	CIZ	ZNF384	0.0917614	57
V$STAT1_05	STAT1	STAT1	0.042721	50
V$HSF1_01	HSF1	HSF1	0.00571941	41
V$IRF2_01	IRF-2	IRF2	0.0547042	16

hsa-mir-374a

Matrix_id	transcription factor	Gene	PCC	Occurrence
V$P300_01	p300	EP300	0.191083	3
V$FKLF_Q5	FKLF	KLF11	0.266174	3
V$HOXD9_Q2	Hoxd9	HOXD9	0.0526632	3

V$PUR1_Q4	PUR1	PURA	0.209241	3
V$PEA3_Q6	PEA3	ETV4	0.014765	3
V$LRF_Q2	LRF	ZBTB7A	0.0340343	3
V$NR1B2_Q6	NR1B2	RARB	0.0350857	3
V$ELF1_Q6	Elf-1	ELF1	0.0172285	3
V$AML1_Q6	AML1	RUNX1	0.063576	3
V$YY1_Q6	YY1	YY1	0.268362	3
V$GR_01	GR	NR3C1	0.133719	3
V$TBP_Q6	TBP	TBP	0.124574	3
V$YY1_Q6_02	YY1	YY1	0.268362	3
V$DLX5_01	dlx5	DLX5	0.35641	3
V$FOXO3A_Q1	FOXO3A	FOXO3	0.235827	3
V$PBX1_04	Pbx1	PBX1	0.111876	3
V$HOXA9_01	hoxa9	HOXA9	0.213993	3
V$GATA6_01	GATA-6	GATA6	0.300894	3
V$CEBPE_Q6	CEBPE	CEBPE	0.113438	3
V$HNF3A_01	HNF3A	FOXA1	0.11592	3
V$GATA1_01	GATA-1	GATA1	0.00766157	3
V$GATA2_01	GATA-2	GATA2	0.635676	3
V$GATA3_01	GATA-3	GATA3	0.625838	3
V$PARP_Q4	PARP	PARP1	0.0122904	3
V$HNF4A_Q6_01	HNF-4alpha	HNF4A	0.0425484	3
V$GATA1_02	GATA-1	GATA1	0.00766157	3
V$GR_Q6	GR	NR3C1	0.133719	3
V$AML1_01	AML1a	RUNX1	0.063576	3
V$GATA1_05	GATA-1	GATA1	0.00766157	3
V$GATA1_06	GATA-1	GATA1	0.00766157	3
V$GATA2_02	GATA-2	GATA2	0.635676	3
V$GATA3_02	GATA-3	GATA3	0.625838	3
V$SMAD4_Q6_01	Smad4	SMAD4	0.143939	3
V$CEBPB_Q6	C/EBPbeta	CEBPB	0.583063	2
V$SPI1_03	SPI1	SPI1	0.224579	2
V$PBX1_Q3	Pbx1	PBX1	0.111876	2
V$AML1_Q4	AML1	RUNX1	0.063576	2
V$TCF4_01	TCF-4	TCF7L2	0.403973	2
V$CEBPG_Q6_01	C/EBPgamma	CEBPG	0.369886	2
V$FOXO4_02	FOXO4	FOXO4	0.620096	2
V$FOXJ2_01	FOXJ2	FOXJ2	0.105962	2
V$MSX1_01	Msx-1	MSX1	0.0188117	2
V$CEBPD_Q6	C/EBPdelta	CEBPD	0.605923	2
V$CEBPG_Q6	C/EBPgamma	CEBPG	0.369886	2
V$CIZ_01	CIZ	ZNF384	0.217114	2
V$TCF4_Q5	TCF-4	TCF7L2	0.403973	2
V$ETS2_B	c-Ets-2	ETS2	0.172148	2
V$CEBPB_02	C/EBPbeta	CEBPB	0.583063	2
V$CEBPB_01	C/EBPbeta	CEBPB	0.583063	2

V$YY1_02	YY1	YY1	0.268362	2
V$PARP_Q3	PARP	PARP1	0.0122904	2
V$YY1_01	YY1	YY1	0.268362	2
V$MAFB_01	MAFB	MAFB	0.274122	2
V$MEIS1_01	MEIS1	MEIS1	0.0908269	2
V$TCF4_Q5_01	TCF-4	TCF7L2	0.403973	1
V$YY1_03	YY1	YY1	0.268362	1
V$YY1_Q6_03	YY1	YY1	0.268362	1
V$FOXO4_01	FOXO4	FOXO4	0.620096	1
V$CDP_04	CDP	CUX1	0.549101	1
V$CTCF_01	CTCF	CTCF	0.270208	1
V$CART1_02	CART1	ALX1	0.0266726	1
V$HOXA7_01	HOXA7	HOXA7	0.127121	1
V$LHX3_01	Lhx3	LHX3	0.012182	1
V$STAT3_03	STAT3	STAT3	0.363652	1
V$GLI3_Q5_01	GLI3	GLI3	0.133621	1
V$CTCF_02	CTCF	CTCF	0.270208	1
V$HBP1_Q2	hbp1	HBP1	0.421938	1

hsa-mir-375

Matrix_id	transcription factor	Gene	PCC	Occurrence
V$SOX9_Q4	SOX9	SOX9	0.13851	39
V$PITX3_Q2	PITX3	PITX3	0.105937	31
V$SOX9_B1	SOX9	SOX9	0.13851	30
V$MEIS1_01	MEIS1	MEIS1	0.00421567	15
V$ESE1_Q3	ESE-1	ELF3	0.285638	9
V$EHF_03	EHF	EHF	0.0226993	6
V$CMYC_02	c-Myc	MYC	0.344965	5
V$ESE1_02	ESE-1	ELF3	0.285638	5
V$ATF6_01	ATF6	ATF6	0.0621595	4
V$ATF3_Q6_01	ATF-3	ATF3	0.0892773	4
V$ATF4_Q6	ATF-4	ATF4	0.0404105	4

hsa-mir-376a-1

Matrix_id	transcription factor	Gene	PCC	Occurrence
V$PUR1_Q4	PUR1	PURA	0.142977	193
V$PEA3_Q6	PEA3	ETV4	0.0926303	190
V$PARP_Q4	PARP	PARP1	0.113077	187

V$ELF1_Q6	Elf-1	ELF1	0.0878452	186
V$GKLF_Q4	GKLF	KLF4	0.481631	181
V$MAFB_01	MAFB	MAFB	0.211231	177
V$P300_01	p300	EP300	0.278437	172
V$SMAD4_Q6_01	Smad4	SMAD4	0.210455	167
V$GABPA_Q4	GABP-alpha	GABPA	0.337713	159
V$GR_Q6	GR	NR3C1	0.0794084	146
V$YY1_01	YY1	YY1	0.366792	143
V$YY1_Q6_02	YY1	YY1	0.366792	133
V$YY1_Q6	YY1	YY1	0.366792	133
V$AHR_Q5	AhR	AHR	0.853696	122
V$ERBETA_Q5	ER-beta	ESR2	0.0112268	118
V$AP2ALPHA_Q6	AP-2alpha	TFAP2A	0.863612	118
V$ING4_01	ING4	ING4	0.0746429	114
V$GATA3_01	GATA-3	GATA3	0.81847	107
V$PBX1_04	Pbx1	PBX1	0.0199438	103
V$GATA2_01	GATA-2	GATA2	0.590198	96
V$GR_01	GR	NR3C1	0.0794084	89
V$TCF4_01	TCF-4	TCF7L2	0.373772	81
V$CREM_Q6	CREM	CREM	0.374112	74
V$GABPBETA_Q3	GABP-beta	GABPB1	0.214315	71
V$CEBPB_02	C/EBPbeta	CEBPB	0.539998	61
V$YY1_02	YY1	YY1	0.366792	59
V$ATF3_Q6_01	ATF-3	ATF3	0.474903	54
V$ELK1_01	Elk-1	ELK1	0.11574	45
V$ATF4_Q6	ATF-4	ATF4	0.0610456	40
V$SMAD4_Q6	SMAD4	SMAD4	0.210455	34
V$MAFK_Q3	MafK	MAFK	0.314305	22

hsa-mir-376a-2

Matrix_id	transcription factor	Gene	PCC	Occurrence
V$PUR1_Q4	PUR1	PURA	0.142977	193
V$PEA3_Q6	PEA3	ETV4	0.0926303	190
V$PARP_Q4	PARP	PARP1	0.113077	187
V$ELF1_Q6	Elf-1	ELF1	0.0878452	186
V$GKLF_Q4	GKLF	KLF4	0.481631	181
V$MAFB_01	MAFB	MAFB	0.211231	177
V$P300_01	p300	EP300	0.278437	172
V$SMAD4_Q6_01	Smad4	SMAD4	0.210455	167
V$GABPA_Q4	GABP-alpha	GABPA	0.337713	159
V$GR_Q6	GR	NR3C1	0.0794084	146
V$YY1_01	YY1	YY1	0.366792	143

V$YY1_Q6_02	YY1	YY1	0.366792	133
V$YY1_Q6	YY1	YY1	0.366792	133
V$AHR_Q5	AhR	AHR	0.853696	122
V$ERBETA_Q5	ER-beta	ESR2	0.0112268	118
V$AP2ALPHA_Q6	AP-2alpha	TFAP2A	0.863612	118
V$ING4_01	ING4	ING4	0.0746429	114
V$GATA3_01	GATA-3	GATA3	0.81847	107
V$PBX1_04	Pbx1	PBX1	0.0199438	103
V$GATA2_01	GATA-2	GATA2	0.590198	96
V$GR_01	GR	NR3C1	0.0794084	89
V$TCF4_01	TCF-4	TCF7L2	0.373772	81
V$CREM_Q6	CREM	CREM	0.374112	74
V$GABPBETA_Q3	GABP-beta	GABPB1	0.214315	71
V$CEBPB_02	C/EBPbeta	CEBPB	0.539998	61
V$YY1_02	YY1	YY1	0.366792	59
V$ATF3_Q6_01	ATF-3	ATF3	0.474903	54
V$ELK1_01	Elk-1	ELK1	0.11574	45
V$ATF4_Q6	ATF-4	ATF4	0.0610456	40
V$SMAD4_Q6	SMAD4	SMAD4	0.210455	34
V$MAFK_Q3	MafK	MAFK	0.314305	22

---

hsa-mir-377

Matrix_id	transcription factor	Gene	PCC	Occurrence
V$PUR1_Q4	PUR1	PURA	0.16005	263
V$PEA3_Q6	PEA3	ETV4	0.0983111	257
V$PARP_Q4	PARP	PARP1	0.0995149	254
V$ELF1_Q6	Elf-1	ELF1	0.165773	249
V$GKLF_Q4	GKLF	KLF4	0.567492	246
V$MAFB_01	MAFB	MAFB	0.300398	241
V$P300_01	p300	EP300	0.349573	236
V$ETS1_B	c-Ets-1	ETS1	0.0679363	230
V$CETS1_Q6	C-ets-1	ETS1	0.0679363	224
V$SMAD4_Q6_01	Smad4	SMAD4	0.308843	223
V$GATA1_01	GATA-1	GATA1	0.0170848	213
V$GABPA_Q4	GABP-alpha	GABPA	0.364776	212
V$GR_Q6	GR	NR3C1	0.157584	201
V$TBX5_02	TBX5	TBX5	0.0788559	200
V$YY1_01	YY1	YY1	0.367543	197
V$YY1_Q6_02	YY1	YY1	0.367543	176
V$YY1_Q6	YY1	YY1	0.367543	176
V$GATA1_02	GATA-1	GATA1	0.0170848	173
V$AHR_Q5	AhR	AHR	0.965365	168

V$ERBETA_Q5	ER-beta	ESR2	0.0421464	160
V$ING4_01	ING4	ING4	0.116608	160
V$AP2ALPHA_Q6	AP-2alpha	TFAP2A	0.959118	153
V$GATA1_04	GATA-1	GATA1	0.0170848	148
V$GATA3_01	GATA-3	GATA3	0.912183	139
V$GATA2_01	GATA-2	GATA2	0.695429	126
V$CMAF_01	c-Maf	MAF	0.0948992	124
V$GR_01	GR	NR3C1	0.157584	119
V$TCF4_01	TCF-4	TCF7L2	0.46753	118
V$CREM_Q6	CREM	CREM	0.172813	98
V$GABPBETA_Q3	GABP-beta	GABPB1	0.228391	96
V$CEBPB_02	C/EBPbeta	CEBPB	0.597165	88
V$YY1_02	YY1	YY1	0.367543	74
V$ATF1_Q6_01	ATF-1	ATF1	0.0627481	70
V$ATF3_Q6_01	ATF-3	ATF3	0.62919	68
V$ELK1_01	Elk-1	ELK1	0.0202257	63
V$ATF4_Q6	ATF-4	ATF4	0.0658991	52
V$SMAD4_Q6	SMAD4	SMAD4	0.308843	46
V$MAFK_Q3	MafK	MAFK	0.278197	29

hsa-mir-378a

Matrix_id	transcription factor	Gene	PCC	Occurrence
V$PUR1_Q4	PUR1	PURA	0.308515	1920
V$IK_Q5	Ikaros	IKZF1	0.714059	1888
V$PARP_Q4	PARP	PARP1	0.553053	1841
V$PEA3_Q6	PEA3	ETV4	0.714305	1822
V$AP2REP_01	AP-2rep	KLF12	0.875255	1817
V$P300_01	p300	EP300	0.713635	1776
V$ZIC3_01	Zic3	ZIC3	0.427406	1664
V$SMAD4_Q6_01	Smad4	SMAD4	0.690403	1640
V$MYB_Q6	c-Myb	MYB	0.265206	1628
V$CMYB_Q5	c-Myb	MYB	0.265206	1628
V$TBP_Q6	TBP	TBP	0.280582	1588
V$CDX2_Q5_02	CDX-2	CDX2	0.823254	1588
V$CETS1_Q6	C-ets-1	ETS1	0.668178	1555
V$NR1B2_Q6	NR1B2	RARB	0.808739	1555
V$NFAT4_Q3	NF-AT4	NFATC3	0.493358	1549
V$GATA1_01	GATA-1	GATA1	0.728682	1546
V$TTF1_Q5	TTF-1	NKX2-1	0.266805	1542
V$ETS1_B	c-Ets-1	ETS1	0.668178	1538
V$ETS2_Q6	c-Ets-2	ETS2	0.590406	1516
V$GABPA_Q4	GABP-alpha	GABPA	0.378707	1478

V$YY1_01	YY1	YY1	0.250643	1471
V$SMAD3_Q6_01	Smad3	SMAD3	0.758721	1468
V$MEF2C_Q4	MEF-2C	MEF2C	0.350492	1457
V$Elf5_03	ELF5	ELF5	0.179456	1443
V$DLX5_01	dlx5	DLX5	0.070861	1424
V$NANOG_02	Nanog	NANOG	0.846331	1414
V$IPF1_01	IPF1	PDX1	0.916747	1377
V$SPI1_03	SPI1	SPI1	0.269746	1366
V$HNF4A_Q6_01	HNF-4alpha	HNF4A	0.360839	1359
V$YY1_Q6	YY1	YY1	0.250643	1357
V$AP4_Q6_02	AP-4	TFAP4	0.567156	1336
V$ETS2_B	c-Ets-2	ETS2	0.590406	1330
V$GATA1_02	GATA-1	GATA1	0.728682	1325
V$SPI1_Q5	SPI1	SPI1	0.269746	1298
V$YY1_Q6_02	YY1	YY1	0.250643	1289
V$GATA2_02	GATA-2	GATA2	0.350323	1285
V$GATA1_05	GATA-1	GATA1	0.728682	1285
V$GATA6_01	GATA-6	GATA6	0.086201	1285
V$GATA1_06	GATA-1	GATA1	0.728682	1285
V$BEN_01	BEN	GTF2IRD1	0.875679	1245
V$LRF_Q2	LRF	ZBTB7A	0.894405	1176
V$PARP_Q3	PARP	PARP1	0.553053	1168
V$E2A_Q6	E2A	TCF3	0.749352	1151
V$E12_Q6	E12	TCF3	0.749352	1150
V$PITX3_Q2	PITX3	PITX3	0.606073	1147
V$E47_02	E47	TCF3	0.749352	1144
V$MYOD_Q6_01	MyoD	MYOD1	0.782742	1142
V$ING4_01	ING4	ING4	0.0961257	1140
V$SP1_Q6	Sp1	SP1	0.870039	1122
V$GATA1_04	GATA-1	GATA1	0.728682	1111
V$IRF4_Q6	IRF-4	IRF4	0.873682	1110
V$CRX_Q4	Crx	CRX	0.756139	1055
V$MYOGENIN_Q6	myogenin	MYOG	0.745715	1044
V$SP1_Q6_01	Sp1	SP1	0.870039	1008
V$AP2ALPHA_Q6	AP-2alpha	TFAP2A	0.123668	1002
V$SP1_01	Sp1	SP1	0.870039	999
V$SP1_Q4_01	Sp1	SP1	0.870039	982
V$GFI1_Q6	Gfi1	GFI1	0.405214	970
V$TCF3_01	TCF-3	TCF7L1	0.720176	968
V$ARNT_01	Arnt	ARNT	0.823503	956
V$FOXJ2_01	FOXJ2	FOXJ2	0.689539	954
V$AP2ALPHA_01	AP-2alpha	TFAP2A	0.123668	953
V$SP1_Q2_01	Sp1	SP1	0.870039	951
V$GFI1_Q6_01	Gfi1	GFI1	0.405214	944
V$PITX2_Q2	Pitx2	PITX2	0.716547	942
V$AP4_Q5	AP-4	TFAP4	0.567156	930

V$WT1_Q6_01	WT1	WT1	0.0621345	923
V$E2A_Q2	E2A	TCF3	0.749352	915
V$SP1_02	SP1	SP1	0.870039	883
V$CMAF_01	c-Maf	MAF	0.378985	883
V$RFX1_02	RFX1	RFX1	0.865122	881
V$PITX2_01	PITX2	PITX2	0.716547	881
V$PITX1_01	Pitx1	PITX1	0.844111	876
V$TFIII_Q6	TFII-I	GTF2I	0.704533	864
V$ZFX_01	Zfx	ZFX	0.796023	855
V$WT1_Q6	WT1	WT1	0.0621345	850
V$AP4_Q6	AP-4	TFAP4	0.567156	822
V$CRX_02	Crx	CRX	0.756139	814
V$GFI1B_01	Gfi1b	GFI1B	0.92407	792
V$ERR1_Q3	ERR1	ESRRA	0.888858	792
V$ELF5_01	ELF5	ELF5	0.179456	765
V$MYOGENIN_Q6_01	myogenin	MYOG	0.745715	750
V$SMAD3_Q6	SMAD3	SMAD3	0.758721	727
V$NEUROD_02	NeuroD	NEUROD1	0.791881	708
V$CP2_01	CP2	TFCP2	0.111587	694
V$MYOD_Q6	MyoD	MYOD1	0.782742	690
V$AP2GAMMA_01	AP-2gamma	TFAP2C	0.241683	688
V$GABPBETA_Q3	GABP-beta	GABPB1	0.579386	673
V$AP2BETA_Q3	AP-2beta	TFAP2B	0.802051	668
V$SP2_01	SP2	SP2	0.734295	662
V$CNOT3_01	CNOT3	CNOT3	0.777575	642
V$PAX8_01	Pax-8	PAX8	0.33615	634
V$E2F1_Q3	E2F-1	E2F1	0.5605	622
V$E2F1_Q3_01	E2F-1	E2F1	0.5605	602
V$GATA2_03	GATA-2	GATA2	0.350323	601
V$AP4_Q6_01	AP-4	TFAP4	0.567156	600
V$MATH1_Q2	MATH1	ATOH1	0.677245	542
V$NURR1_Q3	NURR1	NR4A2	0.00494998	531
V$KAISO_01	KAISO	ZBTB33	0.928694	520
V$RNF96_01	RNF96	TRIM28	0.0646496	514
V$MYOD_01	MyoD	MYOD1	0.782742	511
V$TR4_Q2	TR4	NR2C2	0.875259	422
V$SP4_Q5	SP4	SP4	0.767834	418
V$SMAD4_Q6	SMAD4	SMAD4	0.690403	310
V$ZID_01	ZID	ZBTB6	0.369345	244
V$AMEF2_Q6	aMEF-2	MEF2A	0.0422741	214

----

hsa-mir-379

Matrix_id	transcription factor	Gene	PCC	Occurrence

V$MAFB_01	MAFB	MAFB	0.0728568	15
V$PARP_Q4	PARP	PARP1	0.23061	14
V$P300_01	p300	EP300	0.0975698	13
V$PEA3_Q6	PEA3	ETV4	0.03589	13
V$GATA2_02	GATA-2	GATA2	0.208269	12
V$YY1_Q6	YY1	YY1	0.297849	12
V$GATA6_01	GATA-6	GATA6	0.409645	12
V$SMAD4_Q6_01	Smad4	SMAD4	0.0176811	12
V$YY1_Q6_02	YY1	YY1	0.297849	12
V$TBP_Q6	TBP	TBP	0.17341	11
V$ZIC1_01	Zic1	ZIC1	0.278131	11
V$GATA3_02	GATA-3	GATA3	0.368536	11
V$GABPA_Q4	GABP-alpha	GABPA	0.198908	10
V$PITX3_Q2	PITX3	PITX3	0.2852	10
V$MEF2C_Q4	MEF-2C	MEF2C	0.230243	10
V$DLX5_01	dlx5	DLX5	0.524752	10
V$SOX9_Q4	SOX9	SOX9	0.069732	9
V$PBX1_Q3	Pbx1	PBX1	0.239657	9
V$YY1_01	YY1	YY1	0.297849	9
V$PBX1_04	Pbx1	PBX1	0.239657	9
V$CREM_Q6	CREM	CREM	0.58617	9
V$ATF3_Q6_01	ATF-3	ATF3	0.0370153	9
V$SREBP1_Q6	SREBP-1	SREBF1	0.752608	8
V$SOX9_B1	SOX9	SOX9	0.069732	8
V$ATF4_Q6	ATF-4	ATF4	0.0334967	7
V$HIF1A_Q6	HIF-1alpha	HIF1A	0.540741	7
V$PITX2_01	PITX2	PITX2	0.115951	7
V$PIT1_Q6	Pit-1	POU1F1	0.105052	7
V$FOXJ2_01	FOXJ2	FOXJ2	0.187789	6
V$PITX2_Q2	Pitx2	PITX2	0.115951	6
V$GATA2_03	GATA-2	GATA2	0.208269	6
V$ING4_01	ING4	ING4	0.193797	6
V$HBP1_Q2	hbp1	HBP1	0.309685	5
V$HNF6_Q6	HNF6	ONECUT1	0.197123	5
V$GLI3_Q5_01	GLI3	GLI3	0.00788451	5
V$HMGIY_01	HMGIY	HMGA1	0.0831277	5
V$OC2_Q3	OC-2	ONECUT2	0.0207437	5
V$TCF4_Q5	TCF-4	TCF7L2	0.138151	4
V$FOXO4_02	FOXO4	FOXO4	0.487934	4
V$SREBP1_02	SREBP-1	SREBF1	0.752608	3
V$FOXJ2_02	FOXJ2	FOXJ2	0.187789	3
V$CART1_02	CART1	ALX1	0.0142576	3
V$HOX13_02	HOXA5	HOXA5	0.579097	3
V$FOXO4_01	FOXO4	FOXO4	0.487934	2
V$CDP_02	CDP	CUX1	0.0749813	2

V$CDP_04	CDP	CUX1	0.0749813	2
V$DMRT1_01	DMRT1	DMRT1	0.0101864	1
V$ZABC1_01	ZABC1	ZNF217	0.282236	1

hsa-mir-381

Matrix_id	transcription factor	Gene	PCC	Occurrence
V$MAFB_01	MAFB	MAFB	0.193434	38
V$PUR1_Q4	PUR1	PURA	0.0668109	38
V$ELF1_Q6	Elf-1	ELF1	0.0798617	37
V$GKLF_Q4	GKLF	KLF4	0.418251	36
V$PARP_Q4	PARP	PARP1	0.0965008	36
V$PEA3_Q6	PEA3	ETV4	0.111304	35
V$P300_01	p300	EP300	0.245057	33
V$SMAD4_Q6_01	Smad4	SMAD4	0.176696	33
V$GABPA_Q4	GABP-alpha	GABPA	0.294215	30
V$YY1_01	YY1	YY1	0.338613	29
V$GATA3_01	GATA-3	GATA3	0.702235	28
V$GR_Q6	GR	NR3C1	0.0599845	27
V$YY1_Q6_02	YY1	YY1	0.338613	25
V$ERBETA_Q5	ER-beta	ESR2	0.00724766	24
V$YY1_Q6	YY1	YY1	0.338613	24
V$AHR_Q5	AhR	AHR	0.717636	24
V$GATA2_01	GATA-2	GATA2	0.485597	24
V$AP2ALPHA_Q6	AP-2alpha	TFAP2A	0.741788	22
V$CREM_Q6	CREM	CREM	0.452336	20
V$CMAF_01	c-Maf	MAF	0.00640241	20
V$FOXP3_01	FOXP3	FOXP3	0.0107221	19
V$PBX1_04	Pbx1	PBX1	0.0851922	19
V$GABPBETA_Q3	GABP-beta	GABPB1	0.19623	19
V$ATF3_Q6_01	ATF-3	ATF3	0.398947	16
V$YY1_02	YY1	YY1	0.338613	15
V$GR_01	GR	NR3C1	0.0599845	15
V$CEBPB_02	C/EBPbeta	CEBPB	0.525769	14
V$PIT1_Q6	Pit-1	POU1F1	0.0836381	14
V$ATF4_Q6	ATF-4	ATF4	0.0646266	13
V$TCF4_01	TCF-4	TCF7L2	0.312118	13
V$OC2_Q3	OC-2	ONECUT2	0.0369472	13
V$SMAD4_Q6	SMAD4	SMAD4	0.176696	12
V$ELK1_01	Elk-1	ELK1	0.163572	9
V$MAFK_Q3	MafK	MAFK	0.359209	6

hsa-mir-382

Matrix_id	transcription factor	Gene	PCC	Occurrence
V$PUR1_Q4	PUR1	PURA	0.0666371	44
V$GKLF_Q4	GKLF	KLF4	0.129527	42
V$MAFB_01	MAFB	MAFB	0.0469591	42
V$PARP_Q4	PARP	PARP1	0.164563	41
V$P300_01	p300	EP300	0.0725838	40
V$PEA3_Q6	PEA3	ETV4	0.0482744	39
V$GABPA_Q4	GABP-alpha	GABPA	0.174264	34
V$NR1B2_Q6	NR1B2	RARB	0.00810715	32
V$YY1_Q6_02	YY1	YY1	0.243341	31
V$YY1_01	YY1	YY1	0.243341	31
V$YY1_Q6	YY1	YY1	0.243341	31
V$HNF4A_Q6_01	HNF-4alpha	HNF4A	0.0965072	31
V$GATA3_01	GATA-3	GATA3	0.278887	30
V$BEN_01	BEN	GTF2IRD1	0.00184701	29
V$GATA2_01	GATA-2	GATA2	0.123279	27
V$AP2ALPHA_Q6	AP-2alpha	TFAP2A	0.304833	26
V$PBX1_04	Pbx1	PBX1	0.240835	24
V$AHR_Q5	AhR	AHR	0.26536	23
V$ING4_01	ING4	ING4	0.0522931	22
V$SOX10_Q6	SOX10	SOX10	0.0553344	21
V$CREM_Q6	CREM	CREM	0.582704	19
V$TCF4_01	TCF-4	TCF7L2	0.0594113	17
V$PIT1_Q6	Pit-1	POU1F1	0.261331	17
V$ATF3_Q6_01	ATF-3	ATF3	0.00942728	17
V$FOXP3_01	FOXP3	FOXP3	0.0113991	16
V$GABPBETA_Q3	GABP-beta	GABPB1	0.119655	15
V$ATF4_Q6	ATF-4	ATF4	0.0463478	13
V$YY1_02	YY1	YY1	0.243341	12
V$MAFK_Q3	MafK	MAFK	0.261456	12
V$CEBPB_02	C/EBPbeta	CEBPB	0.231086	11
V$ELK1_01	Elk-1	ELK1	0.276799	9
V$OC2_Q3	OC-2	ONECUT2	0.114576	8

hsa-mir-383

Matrix_id	transcription factor	Gene	PCC	Occurrence
V$PARP_Q4	PARP	PARP1	0.324129	250
V$ZIC3_01	Zic3	ZIC3	0.126025	248

V$ETS2_Q6	c-Ets-2	ETS2	0.0363169	224
V$SOX9_Q4	SOX9	SOX9	0.312324	215
V$TBP_Q6	TBP	TBP	0.103656	210
V$YY1_01	YY1	YY1	0.0590585	199
V$MAZ_Q6	MAZ	MAZ	0.161474	199
V$MEF2C_Q4	MEF-2C	MEF2C	0.694704	188
V$ETS2_B	c-Ets-2	ETS2	0.0363169	186
V$SOX9_B1	SOX9	SOX9	0.312324	181
V$SRY_02	SRY	SRY	0.124904	169
V$PARP_Q3	PARP	PARP1	0.324129	164
V$PBX1_04	Pbx1	PBX1	0.140828	151
V$PBX1_Q3	Pbx1	PBX1	0.140828	145
V$HMGIY_01	HMGIY	HMGA1	0.0889473	144
V$ELK1_02	Elk-1	ELK1	0.0719113	139
V$YY1_Q6_03	YY1	YY1	0.0590585	132
V$LEF1_Q5	LEF-1	LEF1	0.132255	130
V$GFI1_Q6	Gfi1	GFI1	0.0228158	129
V$ELK1_06	ELK-1	ELK1	0.0719113	123
V$GFI1_Q6_01	Gfi1	GFI1	0.0228158	123
V$FOXM1_01	FOXM1	FOXM1	0.137856	117
V$ERF_02	ERF	ERF	0.0923585	115
V$SOX10_Q6	SOX10	SOX10	0.591931	112
V$CEBPG_Q6_01	C/EBPgamma	CEBPG	0.138027	94
V$NKX2B_Q3	NKX2B	NKX2-2	0.765517	89
V$NKX22_02	NKX2B	NKX2-2	0.765517	88
V$MEF2A_Q6	mef2A	MEF2A	0.553299	86
V$CACCCBINDINGFACT	CACCC-binding factor	ZNF148	0.00312383	70
V$SATB1_Q3	SATB1	SATB1	0.37927	25
V$POU6F1_01	POU6F1	POU6F1	0.267865	18

----

hsa-mir-409

Matrix_id	transcription factor	Gene	PCC	Occurrence
V$GKLF_Q4	GKLF	KLF4	0.0651953	49
V$PARP_Q4	PARP	PARP1	0.0482167	48
V$P300_01	p300	EP300	0.0201707	48
V$AP2REP_01	AP-2rep	KLF12	0.0580681	46
V$PEA3_Q6	PEA3	ETV4	0.0730017	45
V$NR1B2_Q6	NR1B2	RARB	0.0344599	41
V$YY1_01	YY1	YY1	0.14981	39
V$GABPA_Q4	GABP-alpha	GABPA	0.0822054	37
V$YY1_Q6	YY1	YY1	0.14981	37
V$YY1_Q6_02	YY1	YY1	0.14981	37

V$HNF4A_Q6_01	HNF-4alpha	HNF4A	0.174762	35
V$BEN_01	BEN	GTF2IRD1	0.0296612	35
V$AP2ALPHA_Q6	AP-2alpha	TFAP2A	0.165628	31
V$PBX1_04	Pbx1	PBX1	0.222953	29
V$CREM_Q6	CREM	CREM	0.536064	27
V$GATA3_01	GATA-3	GATA3	0.152355	27
V$AHR_Q5	AhR	AHR	0.120668	27
V$ZFX_01	Zfx	ZFX	0.0174768	25
V$GATA2_01	GATA-2	GATA2	0.0370907	24
V$TCF4_01	TCF-4	TCF7L2	0.00215121	21
V$FOXP3_01	FOXP3	FOXP3	0.0606143	20
V$PIT1_Q6	Pit-1	POU1F1	0.359035	18
V$CEBPB_02	C/EBPbeta	CEBPB	0.204655	17
V$GABPBETA_Q3	GABP-beta	GABPB1	0.0705598	16
V$ATF4_Q6	ATF-4	ATF4	0.0330784	15
V$YY1_02	YY1	YY1	0.14981	15
V$OC2_Q3	OC-2	ONECUT2	0.16847	14
V$MAFK_Q3	MafK	MAFK	0.276862	13
V$ELK1_01	Elk-1	ELK1	0.238477	8

hsa-mir-422a

Matrix_id	transcription factor	Gene	PCC	Occurrence
V$PUR1_Q4	PUR1	PURA	0.236882	722
V$IK_Q5	Ikaros	IKZF1	0.576902	708
V$AP2REP_01	AP-2rep	KLF12	0.775231	682
V$PEA3_Q6	PEA3	ETV4	0.596389	678
V$PARP_Q4	PARP	PARP1	0.434934	676
V$P300_01	p300	EP300	0.540305	654
V$ZIC3_01	Zic3	ZIC3	0.444452	630
V$SMAD4_Q6_01	Smad4	SMAD4	0.576519	625
V$NR1B2_Q6	NR1B2	RARB	0.811307	606
V$CMYB_Q5	c-Myb	MYB	0.194824	595
V$MYB_Q6	c-Myb	MYB	0.194824	595
V$TBX5_02	TBX5	TBX5	0.880519	586
V$CDX2_Q5_02	CDX-2	CDX2	0.69663	579
V$TBP_Q6	TBP	TBP	0.264176	579
V$TTF1_Q5	TTF-1	NKX2-1	0.117163	578
V$CETS1_Q6	C-ets-1	ETS1	0.743875	567
V$NFAT4_Q3	NF-AT4	NFATC3	0.364751	561
V$SMAD3_Q6_01	Smad3	SMAD3	0.59523	559
V$ETS2_Q6	c-Ets-2	ETS2	0.468685	557
V$ETS1_B	c-Ets-1	ETS1	0.743875	551

V$Elf5_03	ELF5	ELF5	0.306361	545
V$GABPA_Q4	GABP-alpha	GABPA	0.225917	540
V$MEF2C_Q4	MEF-2C	MEF2C	0.243984	536
V$AML1_Q6	AML1	RUNX1	0.802759	528
V$GR_Q6	GR	NR3C1	0.45721	525
V$YY1_Q6	YY1	YY1	0.0705024	517
V$NANOG_02	Nanog	NANOG	0.698039	507
V$HNF4A_Q6_01	HNF-4alpha	HNF4A	0.479983	504
V$DLX5_01	dlx5	DLX5	0.0149381	503
V$IPF1_01	IPF1	PDX1	0.807003	496
V$YY1_Q6_02	YY1	YY1	0.0705024	496
V$SOX5_01	SOX5	SOX5	0.503409	490
V$TBX5_01	TBX5	TBX5	0.880519	487
V$ETS2_B	c-Ets-2	ETS2	0.468685	482
V$ERBETA_Q5	ER-beta	ESR2	0.577366	481
V$GATA1_02	GATA-1	GATA1	0.801471	475
V$SPI1_Q5	SPI1	SPI1	0.263501	465
V$GATA6_01	GATA-6	GATA6	0.246824	457
V$GATA2_02	GATA-2	GATA2	0.272401	457
V$GATA1_06	GATA-1	GATA1	0.801471	457
V$GATA1_05	GATA-1	GATA1	0.801471	457
V$CDX2_Q5_01	Cdx-2	CDX2	0.69663	449
V$IPF1_Q6	IPF1	PDX1	0.807003	448
V$IPF1_Q4_01	IPF1	PDX1	0.807003	436
V$CDX2_01	Cdx-2	CDX2	0.69663	436
V$SRY_02	SRY	SRY	0.449059	430
V$PITX3_Q2	PITX3	PITX3	0.530369	426
V$FOXO3A_Q1	FOXO3A	FOXO3	0.352378	417
V$PARP_Q3	PARP	PARP1	0.434934	416
V$IRF4_Q6	IRF-4	IRF4	0.790363	399
V$HNF3B_Q6	HNF-3beta	FOXA2	0.370497	397
V$CRX_Q4	Crx	CRX	0.720034	386
V$HNF3A_01	HNF3A	FOXA1	0.41439	367
V$GATA2_01	GATA-2	GATA2	0.272401	359
V$IPF1_Q4	IPF1	PDX1	0.807003	353
V$PITX2_Q2	Pitx2	PITX2	0.590155	349
V$CMAF_01	c-Maf	MAF	0.333872	348
V$SOX10_Q6	SOX10	SOX10	0.325047	340
V$GFI1_Q6	Gfi1	GFI1	0.396568	340
V$RFX1_02	RFX1	RFX1	0.720734	327
V$TEF1_Q6_03	TEF-1	TEAD1	0.752515	320
V$CEBPE_Q6	CEBPE	CEBPE	0.804192	318
V$ERR1_Q3	ERR1	ESRRA	0.905361	315
V$AML1_01	AML1a	RUNX1	0.802759	303
V$FOXM1_01	FOXM1	FOXM1	0.0982682	302
V$GR_01	GR	NR3C1	0.45721	284

V$TEF1_Q6	TEF-1	TEAD1	0.752515	283
V$OC2_Q3	OC-2	ONECUT2	0.835899	280
V$AML1_Q4	AML1	RUNX1	0.802759	274
V$SMAD3_Q6	SMAD3	SMAD3	0.59523	273
V$NEUROD_02	NeuroD	NEUROD1	0.643652	266
V$HOXA9_01	hoxa9	HOXA9	0.453302	259
V$IRF7_Q3	IRF-7	IRF7	0.305314	259
V$HBP1_Q2	hbp1	HBP1	0.149123	256
V$CEBPB_02	C/EBPbeta	CEBPB	0.136818	250
V$CEBPD_Q6	C/EBPdelta	CEBPD	0.119341	248
V$NKX22_02	NKX2B	NKX2-2	0.175308	245
V$NKX2B_Q3	NKX2B	NKX2-2	0.175308	241
V$BCL6_Q3_01	Bcl-6	BCL6	0.654333	237
V$CDX1_01	Cdx-1	CDX1	0.53273	233
V$CEBPB_Q6	C/EBPbeta	CEBPB	0.136818	217
V$GATA2_03	GATA-2	GATA2	0.272401	217
V$AML2_01	AML2	RUNX3	0.627736	212
V$CDX2_Q5	Cdx-2	CDX2	0.69663	191
V$IPF1_02	IPF1	PDX1	0.807003	181
V$HNF6_Q6	HNF6	ONECUT1	0.348004	179
V$MEIS1_01	MEIS1	MEIS1	0.00174989	174
V$RORBETA_Q2	RORBETA	RORB	0.662983	154
V$CIZ_01	CIZ	ZNF384	0.528773	148
V$STAT4_Q5	STAT4	STAT4	0.714796	143
V$POU6F1_03	POU6F1	POU6F1	0.692995	113
V$SOX2_Q6	SOX2	SOX2	0.0940149	107
V$HSF1_01	HSF1	HSF1	0.811883	93
V$POU6F1_02	POU6F1	POU6F1	0.692995	88
V$SPIB_01	SPI-B	SPIB	0.731066	83
V$MAFK_Q3	MafK	MAFK	0.779143	77
V$HOXA7_01	HOXA7	HOXA7	0.376998	70
V$RORA1_01	RORalpha1	RORA	0.1754	59
V$POU3F2_02	POU3F2	POU3F2	0.741385	59
V$RORA_Q4	RORalpha	RORA	0.1754	54
V$BACH2_01	Bach2	BACH2	0.660162	47
V$RORA2_01	RORalpha2	RORA	0.1754	28
V$HSF2_01	HSF2	HSF2	0.396999	25

---

hsa-mir-423

Matrix_id	transcription factor	Gene	PCC	Occurrence
V$PARP_Q4	PARP	PARP1	0.399176	24
V$PUR1_Q4	PUR1	PURA	0.411233	24

V$MAFB_01	MAFB	MAFB	0.0802959	23
V$P300_01	p300	EP300	0.04919	22
V$ZIC3_01	Zic3	ZIC3	0.0577577	22
V$YY1_Q6_02	YY1	YY1	0.302139	21
V$DLX5_01	dlx5	DLX5	0.198325	21
V$GKLF_Q4	GKLF	KLF4	0.116376	21
V$YY1_Q6	YY1	YY1	0.302139	20
V$TBP_Q6	TBP	TBP	0.0874085	20
V$MEF2C_Q4	MEF-2C	MEF2C	0.56015	19
V$AML1_Q6	AML1	RUNX1	0.0729633	18
V$NR1B2_Q6	NR1B2	RARB	0.113636	18
V$BEN_01	BEN	GTF2IRD1	0.0544974	17
V$GATA6_01	GATA-6	GATA6	0.313382	17
V$GATA3_02	GATA-3	GATA3	0.0611715	17
V$GATA2_02	GATA-2	GATA2	0.0373258	17
V$PITX3_Q2	PITX3	PITX3	0.211306	17
V$AHR_Q5	AhR	AHR	0.00804603	16
V$SOX9_Q4	SOX9	SOX9	0.196371	16
V$ING4_01	ING4	ING4	0.40576	16
V$HIF1A_Q6	HIF-1alpha	HIF1A	0.471096	16
V$YY1_01	YY1	YY1	0.302139	16
V$GR_Q6	GR	NR3C1	0.181128	16
V$HNF4A_Q6_01	HNF-4alpha	HNF4A	0.11483	14
V$GATA3_01	GATA-3	GATA3	0.0611715	13
V$LHX3b_01	LHX3b	LHX3	0.0838779	12
V$PDEF_02	PDEF	SPDEF	0.0999079	10
V$AML1_01	AML1a	RUNX1	0.0729633	10
V$CREM_Q6	CREM	CREM	0.355357	10
V$STAT3_03	STAT3	STAT3	0.0663789	9
V$MEF2A_Q6	mef2A	MEF2A	0.444348	9
V$SREBP1_Q6	SREBP-1	SREBF1	0.517251	9
V$HOX13_02	HOXA5	HOXA5	0.482217	8
V$AML1_Q4	AML1	RUNX1	0.0729633	8
V$E2F1_Q3	E2F-1	E2F1	0.273847	8
V$ZIC1_01	Zic1	ZIC1	0.624681	8
V$ATF1_Q6_01	ATF-1	ATF1	0.0214282	8
V$E2F1_Q3_01	E2F-1	E2F1	0.273847	7
V$E2F1_Q6_01	E2F-1	E2F1	0.273847	7
V$FOXJ2_02	FOXJ2	FOXJ2	0.209407	7
V$CART1_02	CART1	ALX1	0.0770741	7
V$PAX3_B	Pax-3	PAX3	0.0031746	7
V$IRF8_Q6	IRF-8	IRF8	0.140856	7
V$E2F1_Q6	E2F-1	E2F1	0.273847	7
V$DEC2_Q2	2-Dec	BHLHE41	0.451867	6
V$ATF4_Q6	ATF-4	ATF4	0.0573288	5
V$CDP_02	CDP	CUX1	0.276147	4

V$IRF2_01	IRF-2	IRF2	0.544343	3
V$BEN_02	BEN	GTF2IRD1	0.0544974	3
V$PAX3_01	Pax-3	PAX3	0.0031746	3

----

hsa-mir-424

Matrix_id	transcription factor	Gene	PCC	Occurrence
V$PUR1_Q4	PUR1	PURA	0.133935	254
V$PEA3_Q6	PEA3	ETV4	0.149245	247
V$PARP_Q4	PARP	PARP1	0.0977696	246
V$ELF1_Q6	Elf-1	ELF1	0.0903022	244
V$GKLF_Q4	GKLF	KLF4	0.556734	236
V$P300_01	p300	EP300	0.363605	225
V$ETS1_B	c-Ets-1	ETS1	0.0662677	225
V$CETS1_Q6	C-ets-1	ETS1	0.0662677	219
V$SMAD4_Q6_01	Smad4	SMAD4	0.324426	216
V$GABPA_Q4	GABP-alpha	GABPA	0.362676	208
V$TBX5_02	TBX5	TBX5	0.0999859	197
V$TBP_Q6	TBP	TBP	0.228594	194
V$GR_Q6	GR	NR3C1	0.141711	194
V$YY1_01	YY1	YY1	0.357751	190
V$DLX5_01	dlx5	DLX5	0.732503	174
V$YY1_Q6_02	YY1	YY1	0.357751	173
V$YY1_Q6	YY1	YY1	0.357751	173
V$GATA1_02	GATA-1	GATA1	0.0146568	170
V$GATA1_06	GATA-1	GATA1	0.0146568	163
V$GATA2_02	GATA-2	GATA2	0.693203	163
V$GATA1_05	GATA-1	GATA1	0.0146568	163
V$TBX5_01	TBX5	TBX5	0.0999859	155
V$PARP_Q3	PARP	PARP1	0.0977696	153
V$ERBETA_Q5	ER-beta	ESR2	0.0915042	152
V$GATA1_04	GATA-1	GATA1	0.0146568	144
V$HMGIY_01	HMGIY	HMGA1	0.359626	141
V$GATA3_02	GATA-3	GATA3	0.890682	141
V$YY1_Q6_03	YY1	YY1	0.357751	129
V$CEBPE_Q6	CEBPE	CEBPE	0.00415779	116
V$CEBPA_Q6	C/EBPalpha	CEBPA	0.0512646	114
V$TCF4_01	TCF-4	TCF7L2	0.466281	114
V$TCF4_Q5	TCF-4	TCF7L2	0.466281	106
V$IRF7_Q3	IRF-7	IRF7	0.0609313	103
V$FOXJ2_01	FOXJ2	FOXJ2	0.0817866	100
V$GABPBETA_Q3	GABP-beta	GABPB1	0.279504	96
V$CREM_Q6	CREM	CREM	0.246988	93

V$HBP1_Q2	hbp1	HBP1	0.670397	88
V$CEBPG_Q6_01	C/EBPgamma	CEBPG	0.559674	79
V$GATA2_03	GATA-2	GATA2	0.693203	78
V$FOXO4_02	FOXO4	FOXO4	0.941503	69
V$CEBPA_01	C/EBPalpha	CEBPA	0.0512646	68
V$P53_02	p53	TP53	0.619805	64
V$CIZ_01	CIZ	ZNF384	0.101214	54
V$IRF1_Q6_01	IRF-1	IRF1	0.318356	47
V$CEBPG_Q6	C/EBPgamma	CEBPG	0.559674	45
V$IRF1_Q6	IRF-1	IRF1	0.318356	43
V$CDP_04	CDP	CUX1	0.330086	37
V$FOXO4_01	FOXO4	FOXO4	0.941503	32
V$FOXJ2_02	FOXJ2	FOXJ2	0.0817866	31
V$TCF4_Q5_01	TCF-4	TCF7L2	0.466281	17

----

hsa-mir-425

Matrix_id	transcription factor	Gene	PCC	Occurrence
V$ING4_01	ING4	ING4	0.51036	7
V$MAFB_01	MAFB	MAFB	0.25457	7
V$GKLF_Q4	GKLF	KLF4	0.199429	7
V$PUR1_Q4	PUR1	PURA	0.360231	7
V$PARP_Q4	PARP	PARP1	0.186204	7
V$GABPBETA_Q3	GABP-beta	GABPB1	0.075266	6
V$SOX9_B1	SOX9	SOX9	0.0913982	6
V$GR_Q6	GR	NR3C1	0.0240025	6
V$PBX1_Q3	Pbx1	PBX1	0.175736	6
V$GATA3_01	GATA-3	GATA3	0.46837	6
V$DLX5_01	dlx5	DLX5	0.239588	6
V$MEF2C_Q4	MEF-2C	MEF2C	0.375138	5
V$TFIII_Q6	TFII-I	GTF2I	0.0296081	5
V$GATA2_01	GATA-2	GATA2	0.309342	5
V$CREM_Q6	CREM	CREM	0.0958731	4
V$HOXB8_01	HOXB8	HOXB8	0.0922613	4
V$ATF3_Q6_01	ATF-3	ATF3	0.00619989	4
V$PBX1_04	Pbx1	PBX1	0.175736	4
V$SOX9_Q4	SOX9	SOX9	0.0913982	4
V$GR_01	GR	NR3C1	0.0240025	4
V$SREBP1_Q6	SREBP-1	SREBF1	0.274121	4
V$YY1_01	YY1	YY1	0.296622	4
V$CEBPG_Q6_01	C/EBPgamma	CEBPG	0.463941	3
V$TCF4_01	TCF-4	TCF7L2	0.165885	3
V$ZIC1_01	Zic1	ZIC1	0.520813	3

V$CEBPG_Q6	C/EBPgamma	CEBPG	0.463941	3
V$TCF4_Q5	TCF-4	TCF7L2	0.165885	3
V$NKX22_02	NKX2B	NKX2-2	0.407206	3
V$HBP1_Q2	hbp1	HBP1	0.136215	3
V$NKX2B_Q3	NKX2B	NKX2-2	0.407206	3
V$AMEF2_Q6	aMEF-2	MEF2A	0.423249	2
V$ZID_01	ZID	ZBTB6	0.140433	1
V$ERR3_Q2_01	ERR3	ESRRG	0.149949	1

---

hsa-mir-429

Matrix_id	transcription factor	Gene	PCC	Occurrence
V$SOX9_B1	SOX9	SOX9	0.215688	2
V$Elf5_03	ELF5	ELF5	0.119329	2
V$HNF4A_Q6_01	HNF-4alpha	HNF4A	0.0611599	2
V$STAT3_03	STAT3	STAT3	0.197422	2
V$SOX9_Q4	SOX9	SOX9	0.215688	2
V$MYOGENIN_Q6_01	myogenin	MYOG	0.0230405	2
V$VDR_Q3	VDR	VDR	0.0094247	2
V$ELF1_Q6	Elf-1	ELF1	0.290406	2
V$MYOGENIN_Q6	myogenin	MYOG	0.0230405	2
V$ZIC3_01	Zic3	ZIC3	0.169117	2
V$EGR1_02	EGR-1	EGR1	0.235455	1
V$ATF3_Q6_01	ATF-3	ATF3	0.0933666	1
V$KLF15_Q2	KLF15	KLF15	0.0930646	1
V$HNF3A_01	HNF3A	FOXA1	0.0802477	1
V$HOXA7_01	HOXA7	HOXA7	0.119591	1

---

hsa-mir-432

Matrix_id	transcription factor	Gene	PCC	Occurrence
V$PUR1_Q4	PUR1	PURA	0.203029	26
V$MAFB_01	MAFB	MAFB	0.218449	26
V$GKLF_Q4	GKLF	KLF4	0.352655	25
V$ELF1_Q6	Elf-1	ELF1	0.0246084	25
V$PEA3_Q6	PEA3	ETV4	0.0356127	24
V$PARP_Q4	PARP	PARP1	0.213237	24
V$GATA6_01	GATA-6	GATA6	0.237396	20
V$GATA2_02	GATA-2	GATA2	0.439235	20
V$GABPA_Q4	GABP-alpha	GABPA	0.344267	20

V$TBP_Q6	TBP	TBP	0.13134	20
V$P300_01	p300	EP300	0.2232	19
V$GATA3_02	GATA-3	GATA3	0.661694	18
V$YY1_01	YY1	YY1	0.363741	18
V$GATA3_01	GATA-3	GATA3	0.661694	17
V$DLX5_01	dlx5	DLX5	0.647185	17
V$GATA2_01	GATA-2	GATA2	0.439235	16
V$AHR_Q5	AhR	AHR	0.698504	16
V$MEF2C_Q4	MEF-2C	MEF2C	0.160399	15
V$PARP_Q3	PARP	PARP1	0.213237	14
V$ING4_01	ING4	ING4	0.198323	13
V$PBX1_Q3	Pbx1	PBX1	0.131841	13
V$HMGIY_01	HMGIY	HMGA1	0.179264	12
V$PBX1_04	Pbx1	PBX1	0.131841	11
V$GATA2_03	GATA-2	GATA2	0.439235	11
V$GABPBETA_Q3	GABP-beta	GABPB1	0.175006	10
V$GATA3_03	GATA-3	GATA3	0.661694	10
V$TCF4_01	TCF-4	TCF7L2	0.265251	9
V$YY1_02	YY1	YY1	0.363741	9
V$MEF2A_Q6	mef2A	MEF2A	0.0373571	8
V$FOXJ2_01	FOXJ2	FOXJ2	0.123366	8
V$IRF1_Q6_01	IRF-1	IRF1	0.0778297	7
V$HBP1_Q2	hbp1	HBP1	0.517055	7
V$MEIS1_01	MEIS1	MEIS1	0.0609311	7
V$HOX13_02	HOXA5	HOXA5	0.359085	7
V$PIT1_Q6	Pit-1	POU1F1	0.0102932	7
V$IRF1_Q6	IRF-1	IRF1	0.0778297	7
V$ELK1_01	Elk-1	ELK1	0.17984	6
V$CIZ_01	CIZ	ZNF384	0.213989	6
V$IRF2_01	IRF-2	IRF2	0.346608	2
V$RORA1_01	RORalpha1	RORA	0.262882	1
V$BLIMP1_Q6	Blimp-1	PRDM1	0.0976295	1

hsa-mir-433

Matrix_id	transcription factor	Gene	PCC	Occurrence
V$PUR1_Q4	PUR1	PURA	0.395881	63
V$GKLF_Q4	GKLF	KLF4	0.112909	61
V$MAFB_01	MAFB	MAFB	0.251445	61
V$P300_01	p300	EP300	0.124034	59
V$PARP_Q4	PARP	PARP1	0.40133	58
V$AHR_Q5	AhR	AHR	0.398285	54
V$ZIC3_01	Zic3	ZIC3	0.0698666	52

V$TBP_Q6	TBP	TBP	0.0976548	50
V$YY1_01	YY1	YY1	0.29662	49
V$DLX5_01	dlx5	DLX5	0.425357	48
V$GABPA_Q4	GABP-alpha	GABPA	0.303479	47
V$ING4_01	ING4	ING4	0.566442	46
V$ETS2_Q6	c-Ets-2	ETS2	0.0878396	45
V$MEF2C_Q4	MEF-2C	MEF2C	0.610186	44
V$MAZ_Q6	MAZ	MAZ	0.134893	44
V$SOX9_B1	SOX9	SOX9	0.0799589	43
V$GATA2_02	GATA-2	GATA2	0.202364	41
V$GATA6_01	GATA-6	GATA6	0.0460925	41
V$ETS2_B	c-Ets-2	ETS2	0.0878396	41
V$PBX1_Q3	Pbx1	PBX1	0.233389	38
V$GATA3_02	GATA-3	GATA3	0.352864	38
V$SRY_02	SRY	SRY	0.0540553	36
V$HMGIY_01	HMGIY	HMGA1	0.0547366	35
V$PARP_Q3	PARP	PARP1	0.40133	33
V$MAZ_Q6_01	MAZ	MAZ	0.134893	33
V$GATA3_01	GATA-3	GATA3	0.352864	32
V$GATA2_01	GATA-2	GATA2	0.202364	28
V$PBX1_04	Pbx1	PBX1	0.233389	27
V$FOXJ2_01	FOXJ2	FOXJ2	0.0322552	27
V$GATA3_03	GATA-3	GATA3	0.352864	26
V$GABPBETA_Q3	GABP-beta	GABPB1	0.0947236	25
V$YY1_02	YY1	YY1	0.29662	22
V$HBP1_Q2	hbp1	HBP1	0.214151	21
V$GATA2_03	GATA-2	GATA2	0.202364	20
V$MEF2A_Q6	mef2A	MEF2A	0.442267	20
V$TCF4_01	TCF-4	TCF7L2	0.0887237	18
V$ELK1_01	Elk-1	ELK1	0.188351	15
V$HOXB8_01	HOXB8	HOXB8	0.108716	14
V$HOX13_02	HOXA5	HOXA5	0.152961	13
V$POU6F1_03	POU6F1	POU6F1	0.217573	11
V$CIZ_01	CIZ	ZNF384	0.0983586	9
V$POU6F1_02	POU6F1	POU6F1	0.217573	7
V$IRF2_01	IRF-2	IRF2	0.238237	6
V$RORA1_01	RORalpha1	RORA	0.0873993	6
V$SATB1_Q3	SATB1	SATB1	0.0501951	4
V$POU3F2_01	POU3F2	POU3F2	0.0275594	3

----

hsa-mir-449a

Matrix_id	transcription factor	Gene	PCC	Occurrence

V$AP2REP_01	AP-2rep	KLF12	0.0926368	318
V$GKLF_Q4	GKLF	KLF4	0.216446	318
V$PEA3_Q6	PEA3	ETV4	0.110895	314
V$PARP_Q4	PARP	PARP1	0.183884	313
V$P300_01	p300	EP300	0.0521469	309
V$TBP_Q6	TBP	TBP	0.789443	282
V$SOX9_Q4	SOX9	SOX9	0.681885	276
V$GATA1_01	GATA-1	GATA1	0.145615	270
V$CETS1_Q6	C-ets-1	ETS1	0.126744	269
V$YY1_01	YY1	YY1	0.155546	267
V$NFAT4_Q3	NF-AT4	NFATC3	0.0634676	266
V$ETS1_B	c-Ets-1	ETS1	0.126744	263
V$DLX5_01	dlx5	DLX5	0.28082	251
V$SOX5_01	SOX5	SOX5	0.618166	243
V$SOX9_B1	SOX9	SOX9	0.681885	242
V$SRY_02	SRY	SRY	0.134494	216
V$GATA1_06	GATA-1	GATA1	0.145615	215
V$GATA1_05	GATA-1	GATA1	0.145615	215
V$GATA1_02	GATA-1	GATA1	0.145615	214
V$NKX32_01	Nkx3-2	NKX3-2	0.301695	211
V$PITX3_Q2	PITX3	PITX3	0.00976624	210
V$MAZ_Q6	MAZ	MAZ	0.344514	205
V$ERBETA_Q5	ER-beta	ESR2	0.225526	205
V$ING4_01	ING4	ING4	0.0549097	197
V$HNF3B_Q6	HNF-3beta	FOXA2	0.0854144	186
V$PARP_Q3	PARP	PARP1	0.183884	183
V$NFAT2_Q5	NF-AT2	NFATC1	0.151835	174
V$GATA1_04	GATA-1	GATA1	0.145615	172
V$YY1_Q6_03	YY1	YY1	0.155546	166
V$FOXJ2_01	FOXJ2	FOXJ2	0.280397	165
V$IRF8_Q6	IRF-8	IRF8	0.084734	151
V$TCF4_Q5	TCF-4	TCF7L2	0.109658	148
V$TCF4_01	TCF-4	TCF7L2	0.109658	135
V$WT1_Q6	WT1	WT1	0.185784	122
V$CDX1_01	Cdx-1	CDX1	0.0828025	106
V$LHX3b_01	LHX3b	LHX3	0.133695	98
V$CIZ_01	CIZ	ZNF384	0.0954951	84
V$IRF1_Q6	IRF-1	IRF1	0.0101855	62
V$IRF1_Q6_01	IRF-1	IRF1	0.0101855	58
V$LHX3_01	Lhx3	LHX3	0.133695	55
V$HSF1_01	HSF1	HSF1	0.198501	33
V$AR_Q2	AR	AR	0.0398411	19

---

hsa-mir-450a-1

Matrix_id	transcription factor	Gene	PCC	Occurrence
V$PUR1_Q4	PUR1	PURA	0.16005	263
V$PEA3_Q6	PEA3	ETV4	0.0983111	257
V$PARP_Q4	PARP	PARP1	0.0995149	254
V$ELF1_Q6	Elf-1	ELF1	0.165773	249
V$GKLF_Q4	GKLF	KLF4	0.567492	246
V$MAFB_01	MAFB	MAFB	0.300398	241
V$ETS1_B	c-Ets-1	ETS1	0.0679363	230
V$CETS1_Q6	C-ets-1	ETS1	0.0679363	224
V$SMAD4_Q6_01	Smad4	SMAD4	0.308843	223
V$GATA1_01	GATA-1	GATA1	0.0170848	213
V$GABPA_Q4	GABP-alpha	GABPA	0.364776	212
V$TBP_Q6	TBP	TBP	0.156746	202
V$GR_Q6	GR	NR3C1	0.157584	201
V$YY1_01	YY1	YY1	0.367543	197
V$AML1_Q6	AML1	RUNX1	0.00174447	188
V$DLX5_01	dlx5	DLX5	0.691609	178
V$YY1_Q6_02	YY1	YY1	0.367543	176
V$YY1_Q6	YY1	YY1	0.367543	176
V$GATA1_02	GATA-1	GATA1	0.0170848	173
V$GATA1_05	GATA-1	GATA1	0.0170848	165
V$GATA2_02	GATA-2	GATA2	0.695429	165
V$GATA1_06	GATA-1	GATA1	0.0170848	165
V$ERBETA_Q5	ER-beta	ESR2	0.0421464	160
V$PARP_Q3	PARP	PARP1	0.0995149	159
V$FKLF_Q5	FKLF	KLF11	0.23915	148
V$HMGIY_01	HMGIY	HMGA1	0.341965	145
V$GATA3_02	GATA-3	GATA3	0.912183	141
V$SREBP1_Q6	SREBP-1	SREBF1	0.138094	137
V$YY1_Q6_03	YY1	YY1	0.367543	132
V$CEBPE_Q6	CEBPE	CEBPE	0.0285527	121
V$GR_01	GR	NR3C1	0.157584	119
V$TCF4_01	TCF-4	TCF7L2	0.46753	118
V$CEBPA_Q6	C/EBPalpha	CEBPA	0.0713893	117
V$TCF4_Q5	TCF-4	TCF7L2	0.46753	111
V$IRF7_Q3	IRF-7	IRF7	0.130981	107
V$FOXJ2_01	FOXJ2	FOXJ2	0.0461565	106
V$CREM_Q6	CREM	CREM	0.172813	98
V$GABPBETA_Q3	GABP-beta	GABPB1	0.228391	96
V$HBP1_Q2	hbp1	HBP1	0.668623	90
V$CEBPG_Q6_01	C/EBPgamma	CEBPG	0.547814	82
V$GATA2_03	GATA-2	GATA2	0.695429	77
V$STAT3_03	STAT3	STAT3	0.146628	76
V$FOXO4_02	FOXO4	FOXO4	0.956047	75

V$YY1_02	YY1	YY1	0.367543	74
V$CEBPA_01	C/EBPalpha	CEBPA	0.0713893	72
V$P53_02	p53	TP53	0.605859	66
V$E2F4_Q6	E2F-4	E2F4	0.0663263	65
V$ELK1_01	Elk-1	ELK1	0.0202257	63
V$CIZ_01	CIZ	ZNF384	0.0830076	56
V$IRF1_Q6_01	IRF-1	IRF1	0.338402	49
V$CEBPG_Q6	C/EBPgamma	CEBPG	0.547814	46
V$IRF1_Q6	IRF-1	IRF1	0.338402	44
V$CDP_04	CDP	CUX1	0.363955	38
V$FOXO4_01	FOXO4	FOXO4	0.956047	35
V$FOXJ2_02	FOXJ2	FOXJ2	0.0461565	30
V$TCF4_Q5_01	TCF-4	TCF7L2	0.46753	18

---

hsa-mir-451a

Matrix_id	transcription factor	Gene	PCC	Occurrence
V$PEA3_Q6	PEA3	ETV4	0.028152	123
V$PARP_Q4	PARP	PARP1	0.00849742	120
V$ELF1_Q6	Elf-1	ELF1	0.163205	119
V$GKLF_Q4	GKLF	KLF4	0.34008	118
V$P300_01	p300	EP300	0.211471	110
V$GABPA_Q4	GABP-alpha	GABPA	0.366636	104
V$TBX5_02	TBX5	TBX5	0.00641066	101
V$YY1_01	YY1	YY1	0.187438	100
V$GR_Q6	GR	NR3C1	0.0228463	92
V$TBX5_01	TBX5	TBX5	0.00641066	87
V$YY1_Q6_02	YY1	YY1	0.187438	87
V$YY1_Q6	YY1	YY1	0.187438	86
V$DLX5_01	dlx5	DLX5	0.554156	80
V$PARP_Q3	PARP	PARP1	0.00849742	80
V$ING4_01	ING4	ING4	0.018751	78
V$GATA2_02	GATA-2	GATA2	0.52507	75
V$GATA3_01	GATA-3	GATA3	0.785111	73
V$AHR_Q5	AhR	AHR	0.832834	71
V$SREBP1_Q6	SREBP-1	SREBF1	0.0269049	67
V$HMGIY_01	HMGIY	HMGA1	0.294189	66
V$GATA2_01	GATA-2	GATA2	0.52507	65
V$GR_01	GR	NR3C1	0.0228463	64
V$YY1_Q6_03	YY1	YY1	0.187438	63
V$GATA3_02	GATA-3	GATA3	0.785111	61
V$ARNT_01	Arnt	ARNT	0.196696	61
V$TCF4_01	TCF-4	TCF7L2	0.222608	57

V$CEBPA_Q6	C/EBPalpha	CEBPA	0.10742	54
V$IRF7_Q3	IRF-7	IRF7	0.140305	46
V$GATA3_03	GATA-3	GATA3	0.785111	45
V$ZABC1_01	ZABC1	ZNF217	0.711344	45
V$PITX2_Q2	Pitx2	PITX2	0.37737	44
V$PITX2_01	PITX2	PITX2	0.37737	40
V$CEBPB_02	C/EBPbeta	CEBPB	0.520402	39
V$CEBPG_Q6_01	C/EBPgamma	CEBPG	0.508837	34
V$IRF1_Q6_01	IRF-1	IRF1	0.28624	22
V$CDP_04	CDP	CUX1	0.193439	16
V$CDP_01	CDP	CUX1	0.193439	3

hsa-mir-452

Matrix_id	transcription factor	Gene	PCC	Occurrence
V$PEA3_Q6	PEA3	ETV4	0.0983111	257
V$PARP_Q4	PARP	PARP1	0.0995149	254
V$ELF1_Q6	Elf-1	ELF1	0.165773	249
V$P300_01	p300	EP300	0.349573	236
V$ETS1_B	c-Ets-1	ETS1	0.0679363	230
V$CETS1_Q6	C-ets-1	ETS1	0.0679363	224
V$SMAD4_Q6_01	Smad4	SMAD4	0.308843	223
V$GABPA_Q4	GABP-alpha	GABPA	0.364776	212
V$TBP_Q6	TBP	TBP	0.156746	202
V$TBX5_02	TBX5	TBX5	0.0788559	200
V$YY1_01	YY1	YY1	0.367543	197
V$AML1_Q6	AML1	RUNX1	0.00174447	188
V$DLX5_01	dlx5	DLX5	0.691609	178
V$YY1_Q6_02	YY1	YY1	0.367543	176
V$YY1_Q6	YY1	YY1	0.367543	176
V$GATA1_02	GATA-1	GATA1	0.0170848	173
V$AHR_Q5	AhR	AHR	0.965365	168
V$GATA1_06	GATA-1	GATA1	0.0170848	165
V$GATA2_02	GATA-2	GATA2	0.695429	165
V$GATA1_05	GATA-1	GATA1	0.0170848	165
V$ERBETA_Q5	ER-beta	ESR2	0.0421464	160
V$PARP_Q3	PARP	PARP1	0.0995149	159
V$GATA1_04	GATA-1	GATA1	0.0170848	148
V$HMGIY_01	HMGIY	HMGA1	0.341965	145
V$GATA3_02	GATA-3	GATA3	0.912183	141
V$GATA3_01	GATA-3	GATA3	0.912183	139
V$YY1_Q6_03	YY1	YY1	0.367543	132
V$GATA2_01	GATA-2	GATA2	0.695429	126

V$CEBPE_Q6	CEBPE	CEBPE	0.0285527	121
V$TCF4_01	TCF-4	TCF7L2	0.46753	118
V$CEBPA_Q6	C/EBPalpha	CEBPA	0.0713893	117
V$TCF4_Q5	TCF-4	TCF7L2	0.46753	111
V$PITX2_Q2	Pitx2	PITX2	0.430072	111
V$FOXJ2_01	FOXJ2	FOXJ2	0.0461565	106
V$AML1_01	AML1a	RUNX1	0.00174447	105
V$CEBPD_Q6	C/EBPdelta	CEBPD	0.438292	98
V$AML1_Q4	AML1	RUNX1	0.00174447	90
V$HBP1_Q2	hbp1	HBP1	0.668623	90
V$CEBPB_02	C/EBPbeta	CEBPB	0.597165	88
V$CEBPG_Q6_01	C/EBPgamma	CEBPG	0.547814	82
V$GATA2_03	GATA-2	GATA2	0.695429	77
V$CEBPB_Q6	C/EBPbeta	CEBPB	0.597165	76
V$YY1_02	YY1	YY1	0.367543	74
V$CEBPA_01	C/EBPalpha	CEBPA	0.0713893	72
V$YY1_03	YY1	YY1	0.367543	58
V$IRF1_Q6_01	IRF-1	IRF1	0.338402	49
V$CEBPG_Q6	C/EBPgamma	CEBPG	0.547814	46
V$CEBPB_01	C/EBPbeta	CEBPB	0.597165	46
V$CDP_04	CDP	CUX1	0.363955	38
V$FOXO4_01	FOXO4	FOXO4	0.956047	35
V$IRF7_01	IRF-7	IRF7	0.130981	31
V$FOXJ2_02	FOXJ2	FOXJ2	0.0461565	30
V$IRF2_01	IRF-2	IRF2	0.072088	17

hsa-mir-485

Matrix_id	transcription factor	Gene	PCC	Occurrence
V$PUR1_Q4	PUR1	PURA	0.183734	21
V$MAFB_01	MAFB	MAFB	0.0816709	21
V$P300_01	p300	EP300	0.0146241	20
V$PARP_Q4	PARP	PARP1	0.285893	20
V$GABPA_Q4	GABP-alpha	GABPA	0.18758	17
V$AP4_Q6_02	AP-4	TFAP4	0.114852	16
V$NR1B2_Q6	NR1B2	RARB	0.00132639	16
V$SOX9_Q4	SOX9	SOX9	0.0353639	15
V$YY1_Q6	YY1	YY1	0.201268	15
V$YY1_Q6_02	YY1	YY1	0.201268	14
V$HNF4A_Q6_01	HNF-4alpha	HNF4A	0.106423	14
V$YY1_01	YY1	YY1	0.201268	14
V$GATA3_01	GATA-3	GATA3	0.11806	14
V$AP2ALPHA_Q6	AP-2alpha	TFAP2A	0.151577	13

V$AHR_Q5	AhR	AHR	0.123682	13
V$ING4_01	ING4	ING4	0.267673	12
V$CREM_Q6	CREM	CREM	0.4933	12
V$PBX1_04	Pbx1	PBX1	0.293788	12
V$PIT1_Q6	Pit-1	POU1F1	0.247375	11
V$SOX10_Q6	SOX10	SOX10	0.312386	10
V$GABPBETA_Q3	GABP-beta	GABPB1	0.0377907	9
V$ATF4_Q6	ATF-4	ATF4	0.0826135	8
V$MAFK_Q3	MafK	MAFK	0.160717	6
V$OC2_Q3	OC-2	ONECUT2	0.0852206	5
V$CEBPB_02	C/EBPbeta	CEBPB	0.120719	5
V$SOX2_Q6	SOX2	SOX2	0.0667337	4
V$YY1_02	YY1	YY1	0.201268	4
V$AP4_01	AP-4	TFAP4	0.114852	4
V$ELK1_01	Elk-1	ELK1	0.267713	3
V$POU3F2_02	POU3F2	POU3F2	0.0243298	1

hsa-mir-489

Matrix_id	transcription factor	Gene	PCC	Occurrence
V$PUR1_Q4	PUR1	PURA	0.301679	186
V$PEA3_Q6	PEA3	ETV4	0.361981	185
V$IK_Q5	Ikaros	IKZF1	0.18795	183
V$GKLF_Q4	GKLF	KLF4	0.359224	180
V$ELF1_Q6	Elf-1	ELF1	0.141051	178
V$AP2REP_01	AP-2rep	KLF12	0.219215	176
V$ZIC3_01	Zic3	ZIC3	0.324106	170
V$ETS1_B	c-Ets-1	ETS1	0.249833	170
V$P300_01	p300	EP300	0.615159	168
V$MAFB_01	MAFB	MAFB	0.180416	168
V$CETS1_Q6	C-ets-1	ETS1	0.249833	164
V$GATA1_01	GATA-1	GATA1	0.0604924	160
V$SMAD4_Q6_01	Smad4	SMAD4	0.527986	160
V$NR1B2_Q6	NR1B2	RARB	0.339113	158
V$GABPA_Q4	GABP-alpha	GABPA	0.482463	156
V$NFAT4_Q3	NF-AT4	NFATC3	0.176606	153
V$ETS2_Q6	c-Ets-2	ETS2	0.173266	151
V$Elf5_03	ELF5	ELF5	0.00485435	151
V$ETS2_B	c-Ets-2	ETS2	0.173266	147
V$AML1_Q6	AML1	RUNX1	0.213501	145
V$TBX5_02	TBX5	TBX5	0.325125	144
V$SMAD3_Q6_01	Smad3	SMAD3	0.324383	144
V$GR_Q6	GR	NR3C1	0.421081	144

V$YY1_01	YY1	YY1	0.400262	142
V$AP4_Q6_02	AP-4	TFAP4	0.203886	130
V$YY1_Q6	YY1	YY1	0.400262	127
V$YY1_Q6_02	YY1	YY1	0.400262	127
V$SOX5_01	SOX5	SOX5	0.202029	126
V$ING4_01	ING4	ING4	0.125499	123
V$NKX32_01	Nkx3-2	NKX3-2	0.184541	122
V$AHR_Q5	AhR	AHR	0.722359	120
V$LRF_Q2	LRF	ZBTB7A	0.327999	119
V$PARP_Q3	PARP	PARP1	0.335005	119
V$MAZ_Q6	MAZ	MAZ	0.130453	117
V$TBX5_01	TBX5	TBX5	0.325125	117
V$ERBETA_Q5	ER-beta	ESR2	0.373296	116
V$BEN_01	BEN	GTF2IRD1	0.272587	113
V$SP1_Q6	Sp1	SP1	0.51601	113
V$AP2ALPHA_Q6	AP-2alpha	TFAP2A	0.870528	105
V$HNF3A_01	HNF3A	FOXA1	0.0335661	105
V$E2A_Q6	E2A	TCF3	0.243207	105
V$MYOGENIN_Q6	myogenin	MYOG	0.346926	105
V$E12_Q6	E12	TCF3	0.243207	105
V$ZBP89_Q4	ZBP89	ZNF148	0.108967	104
V$MYOD_Q6_01	MyoD	MYOD1	0.211189	103
V$E47_02	E47	TCF3	0.243207	103
V$FKLF_Q5	FKLF	KLF11	0.20559	102
V$NFAT2_Q5	NF-AT2	NFATC1	0.17979	100
V$AP2ALPHA_01	AP-2alpha	TFAP2A	0.870528	98
V$SREBP1_Q6	SREBP-1	SREBF1	0.0538091	96
V$TFIII_Q6	TFII-I	GTF2I	0.379764	96
V$SP1_Q6_01	Sp1	SP1	0.51601	95
V$TEL1_02	TEL1	ETV6	0.267939	94
V$ELK1_02	Elk-1	ELK1	0.336991	94
V$AP4_Q5	AP-4	TFAP4	0.203886	92
V$SP1_Q4_01	Sp1	SP1	0.51601	92
V$SP1_02	SP1	SP1	0.51601	90
V$CMAF_01	c-Maf	MAF	0.603482	89
V$TEF1_Q6_03	TEF-1	TEAD1	0.314448	89
V$E2A_Q2	E2A	TCF3	0.243207	88
V$ETV3_02	ETV3	ETV3	0.0134195	88
V$GR_01	GR	NR3C1	0.421081	86
V$WT1_Q6_01	WT1	WT1	0.07186	86
V$TEF1_Q6	TEF-1	TEAD1	0.314448	86
V$ER71_02	ER71	ETV2	0.198968	86
V$ELF5_01	ELF5	ELF5	0.00485435	86
V$ELK1_06	ELK-1	ELK1	0.336991	86
V$AP4_Q6	AP-4	TFAP4	0.203886	83
V$ETV7_01	ETV7	ETV7	0.349655	82

V$KLF15_Q2	KLF15	KLF15	0.0133101	76
V$DAX1_01	Dax1	NR0B1	0.13156	76
V$ESE1_Q3	ESE-1	ELF3	0.300406	75
V$MYOGENIN_Q6_01	myogenin	MYOG	0.346926	74
V$EHF_03	EHF	EHF	0.0567966	71
V$ERF_02	ERF	ERF	0.011515	70
V$SP2_01	SP2	SP2	0.312155	70
V$CEBPD_Q6	C/EBPdelta	CEBPD	0.37357	68
V$AP2BETA_Q3	AP-2beta	TFAP2B	0.358305	68
V$MYOD_Q6	MyoD	MYOD1	0.211189	67
V$VDR_Q3	VDR	VDR	0.325361	65
V$E47_01	E47	TCF3	0.243207	64
V$MATH1_Q2	MATH1	ATOH1	0.241646	60
V$PAX8_01	Pax-8	PAX8	0.118261	60
V$MYOD_Q6_02	MyoD	MYOD1	0.211189	56
V$ESE1_02	ESE-1	ELF3	0.300406	55
V$STAT3_03	STAT3	STAT3	0.548029	53
V$CACCCBINDINGFACT	CACCC-binding factor	ZNF148	0.108967	53
V$YY1_02	YY1	YY1	0.400262	51
V$E2F4_Q6	E2F-4	E2F4	0.363932	48
V$MYOD_01	MyoD	MYOD1	0.211189	48
V$PET1_02	Pet-1	FEV	0.0953682	46
V$ERG_03	ERG	ERG	0.670624	43
V$AP4_01	AP-4	TFAP4	0.203886	40
V$EGR2_01	Egr-2	EGR2	0.030369	34
V$HTF4_Q2	HTF4	TCF12	0.151298	34
V$ERG_01	ERG	ERG	0.670624	33
V$EKLF_Q5	EKLF	KLF1	0.281594	33
V$BEN_02	BEN	GTF2IRD1	0.272587	23
V$SREBP1_02	SREBP-1	SREBF1	0.0538091	23
V$MAFK_Q3	MafK	MAFK	0.392057	22
V$NANOG_01	Nanog	NANOG	0.257987	22
V$CTCF_02	CTCF	CTCF	0.209937	18
V$CTCF_01	CTCF	CTCF	0.209937	12
V$GRE_C	GR	NR3C1	0.421081	11

----

hsa-mir-490

Matrix_id	transcription factor	Gene	PCC	Occurrence
V$AP2REP_01	AP-2rep	KLF12	0.15918	104
V$ZIC3_01	Zic3	ZIC3	0.214602	99
V$GATA1_01	GATA-1	GATA1	0.657181	91
V$TBP_Q6	TBP	TBP	0.128643	86

V$NR1B2_Q6	NR1B2	RARB	0.26815	85
V$HNF4A_Q6_01	HNF-4alpha	HNF4A	0.310489	77
V$SOX5_01	SOX5	SOX5	0.111082	73
V$GATA1_02	GATA-1	GATA1	0.657181	71
V$E2A_Q6	E2A	TCF3	0.152616	67
V$E47_02	E47	TCF3	0.152616	67
V$E12_Q6	E12	TCF3	0.152616	67
V$NFAT2_Q5	NF-AT2	NFATC1	0.176785	64
V$IPF1_Q6	IPF1	PDX1	0.120426	64
V$GATA1_04	GATA-1	GATA1	0.657181	61
V$IPF1_Q4_01	IPF1	PDX1	0.120426	61
V$IPF1_01	IPF1	PDX1	0.120426	59
V$CRX_Q4	Crx	CRX	0.149663	53
V$IRF4_Q6	IRF-4	IRF4	0.106713	52
V$CRX_02	Crx	CRX	0.149663	49
V$IRF7_Q3	IRF-7	IRF7	0.391912	45
V$GFI1_Q6_01	Gfi1	GFI1	0.143232	44
V$GFI1_Q6	Gfi1	GFI1	0.143232	43
V$FOXJ2_01	FOXJ2	FOXJ2	0.0108751	41
V$FOXP3_01	FOXP3	FOXP3	0.0244998	39
V$CRX_Q4_01	CRX	CRX	0.149663	38
V$CEBPE_Q6	CEBPE	CEBPE	0.883683	36
V$GFI1B_01	Gfi1b	GFI1B	0.227485	36
V$STAT3_03	STAT3	STAT3	0.004889	34
V$P53_02	p53	TP53	0.05944	33
V$FOXO4_02	FOXO4	FOXO4	0.00643233	32
V$IPF1_02	IPF1	PDX1	0.120426	25
V$E2F1_Q4	E2F-1	E2F1	0.107374	19
V$FOXO4_01	FOXO4	FOXO4	0.00643233	13
V$IRF3_Q3	IRF-3	IRF3	0.131933	5

----

hsa-mir-491

Matrix_id	transcription factor	Gene	PCC	Occurrence
V$PUR1_Q4	PUR1	PURA	0.372872	75
V$IK_Q5	Ikaros	IKZF1	0.192787	73
V$AP2REP_01	AP-2rep	KLF12	0.33799	70
V$PEA3_Q6	PEA3	ETV4	0.208569	69
V$ZIC3_01	Zic3	ZIC3	0.336089	67
V$NR1B2_Q6	NR1B2	RARB	0.385186	66
V$PARP_Q4	PARP	PARP1	0.596644	66
V$ETS2_Q6	c-Ets-2	ETS2	0.335067	64
V$SMAD4_Q6_01	Smad4	SMAD4	0.121462	63

V$ETS1_B	c-Ets-1	ETS1	0.166814	62
V$CETS1_Q6	C-ets-1	ETS1	0.166814	61
V$TBX5_02	TBX5	TBX5	0.295808	60
V$SOX9_Q4	SOX9	SOX9	0.151088	59
V$LRF_Q2	LRF	ZBTB7A	0.316067	59
V$GATA1_01	GATA-1	GATA1	0.232038	57
V$TBP_Q6	TBP	TBP	0.212651	56
V$GR_Q6	GR	NR3C1	0.173022	56
V$CDX2_Q5_02	CDX-2	CDX2	0.275298	56
V$SMAD3_Q6_01	Smad3	SMAD3	0.135857	56
V$ETS2_B	c-Ets-2	ETS2	0.335067	55
V$AML1_Q6	AML1	RUNX1	0.281284	55
V$AP4_Q6_02	AP-4	TFAP4	0.471519	54
V$YY1_01	YY1	YY1	0.0762335	52
V$YY1_Q6	YY1	YY1	0.0762335	51
V$SP1_Q6_01	Sp1	SP1	0.328837	50
V$HNF4A_Q6_01	HNF-4alpha	HNF4A	0.250728	49
V$ERBETA_Q5	ER-beta	ESR2	0.163482	48
V$SP1_Q2_01	Sp1	SP1	0.328837	48
V$NANOG_02	Nanog	NANOG	0.230193	47
V$TBX5_01	TBX5	TBX5	0.295808	47
V$YY1_Q6_02	YY1	YY1	0.0762335	47
V$MAZ_Q6_01	MAZ	MAZ	0.332684	46
V$ING4_01	ING4	ING4	0.484665	46
V$DLX5_01	dlx5	DLX5	0.136829	46
V$GATA6_01	GATA-6	GATA6	0.290387	45
V$IPF1_01	IPF1	PDX1	0.317745	45
V$GATA1_05	GATA-1	GATA1	0.232038	45
V$GATA1_06	GATA-1	GATA1	0.232038	45
V$ELK1_02	Elk-1	ELK1	0.568764	44
V$TEL1_02	TEL1	ETV6	0.272595	44
V$AML2_Q3	AML2	RUNX3	0.0817732	44
V$ETV3_02	ETV3	ETV3	0.273746	43
V$PARP_Q3	PARP	PARP1	0.596644	41
V$NKX32_01	Nkx3-2	NKX3-2	0.250202	41
V$GATA1_02	GATA-1	GATA1	0.232038	40
V$ER71_02	ER71	ETV2	0.258937	40
V$IRF8_Q6	IRF-8	IRF8	0.0960786	39
V$HIF1A_Q6	HIF-1alpha	HIF1A	0.135983	37
V$ELK1_06	ELK-1	ELK1	0.568764	37
V$ARNT_01	Arnt	ARNT	0.230308	37
V$FOXO3A_Q1	FOXO3A	FOXO3	0.183346	36
V$SMAD3_Q6	SMAD3	SMAD3	0.135857	36
V$AML1_01	AML1a	RUNX1	0.281284	35
V$SOX10_Q6	SOX10	SOX10	0.687805	35
V$HNF3B_Q6	HNF-3beta	FOXA2	0.15145	34

V$PBX1_Q3	Pbx1	PBX1	0.0704719	34
V$HNF3A_01	HNF3A	FOXA1	0.0185626	34
V$NEUROD_02	NeuroD	NEUROD1	0.389107	33
V$AML2_Q3_01	AML2	RUNX3	0.0817732	33
V$AP4_Q5	AP-4	TFAP4	0.471519	33
V$FOXJ2_01	FOXJ2	FOXJ2	0.335192	32
V$NFAT2_Q5	NF-AT2	NFATC1	0.285079	32
V$TCF3_01	TCF-3	TCF7L1	0.0464049	32
V$AML1_Q4	AML1	RUNX1	0.281284	31
V$GATA1_04	GATA-1	GATA1	0.232038	31
V$GR_01	GR	NR3C1	0.173022	30
V$CP2_01	CP2	TFCP2	0.350683	29
V$PBX1_04	Pbx1	PBX1	0.0704719	27
V$BCL6_Q3_01	Bcl-6	BCL6	0.29389	27
V$FOXP3_01	FOXP3	FOXP3	0.292759	26
V$NKX32_02	Nkx3-2	NKX3-2	0.250202	25
V$AP4_Q6	AP-4	TFAP4	0.471519	25
V$YY1_02	YY1	YY1	0.0762335	24
V$AML2_01	AML2	RUNX3	0.0817732	23
V$MYOGENIN_Q6_01	myogenin	MYOG	0.234835	23
V$SPIB_03	Spi-B	SPIB	0.217552	23
V$MATH1_Q2	MATH1	ATOH1	0.242766	21
V$NKX2B_Q3	NKX2B	NKX2-2	0.765307	19
V$DEC2_Q2	2-Dec	BHLHE41	0.322688	18
V$NKX22_02	NKX2B	NKX2-2	0.765307	18
V$AP4_01	AP-4	TFAP4	0.471519	15
V$STAF_02	Staf	ZNF143	0.211401	15
V$CIZ_01	CIZ	ZNF384	0.315416	14
V$AP4_Q6_01	AP-4	TFAP4	0.471519	14
V$MITF_Q6	MITF	MITF	0.105988	14
V$HOXA7_01	HOXA7	HOXA7	0.149293	5

----

hsa-mir-493

Matrix_id	transcription factor	Gene	PCC	Occurrence
V$PUR1_Q4	PUR1	PURA	0.0893183	180
V$PEA3_Q6	PEA3	ETV4	0.104051	177
V$PARP_Q4	PARP	PARP1	0.0672683	174
V$ELF1_Q6	Elf-1	ELF1	0.109873	173
V$GKLF_Q4	GKLF	KLF4	0.461413	169
V$MAFB_01	MAFB	MAFB	0.206165	167
V$P300_01	p300	EP300	0.270143	162
V$SMAD4_Q6_01	Smad4	SMAD4	0.196196	154

V$GABPA_Q4	GABP-alpha	GABPA	0.340139	149
V$TBP_Q6	TBP	TBP	0.0875786	137
V$GR_Q6	GR	NR3C1	0.0652598	136
V$YY1_01	YY1	YY1	0.322781	135
V$YY1_Q6	YY1	YY1	0.322781	126
V$YY1_Q6_02	YY1	YY1	0.322781	125
V$PARP_Q3	PARP	PARP1	0.0672683	116
V$ERBETA_Q5	ER-beta	ESR2	0.0103181	112
V$AHR_Q5	AhR	AHR	0.842406	110
V$AP2ALPHA_Q6	AP-2alpha	TFAP2A	0.855177	107
V$FKLF_Q5	FKLF	KLF11	0.259522	105
V$SP1_Q6	Sp1	SP1	0.288308	105
V$PITX3_Q2	PITX3	PITX3	0.076534	97
V$AP2ALPHA_01	AP-2alpha	TFAP2A	0.855177	96
V$PBX1_Q3	Pbx1	PBX1	0.00229058	96
V$SREBP1_Q6	SREBP-1	SREBF1	0.434436	94
V$ELK1_02	Elk-1	ELK1	0.0879281	85
V$CEBPE_Q6	CEBPE	CEBPE	0.00664291	80
V$ELK1_06	ELK-1	ELK1	0.0879281	77
V$CEBPA_Q6	C/EBPalpha	CEBPA	0.0187781	77
V$TCF4_01	TCF-4	TCF7L2	0.347641	76
V$TCF4_Q5	TCF-4	TCF7L2	0.347641	74
V$CP2_01	CP2	TFCP2	0.59812	73
V$CREM_Q6	CREM	CREM	0.331846	72
V$ESE1_Q3	ESE-1	ELF3	0.0967427	71
V$PITX2_Q2	Pitx2	PITX2	0.37631	69
V$GABPBETA_Q3	GABP-beta	GABPB1	0.169096	68
V$ZABC1_01	ZABC1	ZNF217	0.724792	65
V$PITX2_01	PITX2	PITX2	0.37631	64
V$CEBPD_Q6	C/EBPdelta	CEBPD	0.356791	59
V$CNOT3_01	CNOT3	CNOT3	0.119487	58
V$YY1_02	YY1	YY1	0.322781	56
V$CEBPB_02	C/EBPbeta	CEBPB	0.559343	55
V$STAT3_03	STAT3	STAT3	0.188556	55
V$ATF3_Q6_01	ATF-3	ATF3	0.520233	53
V$P53_02	p53	TP53	0.500253	53
V$ESE1_02	ESE-1	ELF3	0.0967427	53
V$EAR2_Q2	EAR2	NR2F6	0.402434	50
V$CIZ_01	CIZ	ZNF384	0.184718	46
V$CEBPB_Q6	C/EBPbeta	CEBPB	0.559343	44
V$CEBPA_01	C/EBPalpha	CEBPA	0.0187781	43
V$ERG_03	ERG	ERG	0.264218	41
V$ATF4_Q6	ATF-4	ATF4	0.0758398	40
V$SMAD4_Q6	SMAD4	SMAD4	0.196196	32
V$AP2ALPHA_02	AP-2alphaA	TFAP2A	0.855177	27
V$ERM_02	Erm	ETV5	0.665003	27

V$SREBP1_02	SREBP-1	SREBF1	0.434436	20

hsa-mir-494

Matrix_id	transcription factor	Gene	PCC	Occurrence
V$GKLF_Q4	GKLF	KLF4	0.0650543	31
V$P300_01	p300	EP300	0.00936063	30
V$PEA3_Q6	PEA3	ETV4	0.0662351	30
V$GABPA_Q4	GABP-alpha	GABPA	0.134904	27
V$YY1_Q6_02	YY1	YY1	0.100715	25
V$HNF4A_Q6_01	HNF-4alpha	HNF4A	0.075898	25
V$YY1_Q6	YY1	YY1	0.100715	25
V$YY1_01	YY1	YY1	0.100715	25
V$GATA3_01	GATA-3	GATA3	0.233176	22
V$AP2ALPHA_Q6	AP-2alpha	TFAP2A	0.269761	19
V$PBX1_04	Pbx1	PBX1	0.154179	19
V$GATA2_01	GATA-2	GATA2	0.0700902	19
V$AHR_Q5	AhR	AHR	0.229793	18
V$CREM_Q6	CREM	CREM	0.458207	14
V$FOXP3_01	FOXP3	FOXP3	0.00558172	12
V$ATF3_Q6_01	ATF-3	ATF3	0.0810135	12
V$YY1_02	YY1	YY1	0.100715	12
V$ATF4_Q6	ATF-4	ATF4	0.0555603	11
V$CEBPB_02	C/EBPbeta	CEBPB	0.23209	11
V$PIT1_Q6	Pit-1	POU1F1	0.276984	11
V$MAFK_Q3	MafK	MAFK	0.311624	9
V$ELK1_01	Elk-1	ELK1	0.169856	6
V$OC2_Q3	OC-2	ONECUT2	0.0761184	6

hsa-mir-495

Matrix_id	transcription factor	Gene	PCC	Occurrence
V$PUR1_Q4	PUR1	PURA	0.123894	114
V$PARP_Q4	PARP	PARP1	0.120403	111
V$ELF1_Q6	Elf-1	ELF1	0.0711661	111
V$PEA3_Q6	PEA3	ETV4	0.0773785	111
V$GKLF_Q4	GKLF	KLF4	0.397486	108
V$MAFB_01	MAFB	MAFB	0.202865	106
V$SMAD4_Q6_01	Smad4	SMAD4	0.152645	102
V$P300_01	p300	EP300	0.239312	100

V$GABPA_Q4	GABP-alpha	GABPA	0.339167	92
V$YY1_01	YY1	YY1	0.323929	90
V$GR_Q6	GR	NR3C1	0.051421	89
V$YY1_Q6_02	YY1	YY1	0.323929	82
V$YY1_Q6	YY1	YY1	0.323929	80
V$ING4_01	ING4	ING4	0.0441198	69
V$AHR_Q5	AhR	AHR	0.76656	68
V$AP2ALPHA_Q6	AP-2alpha	TFAP2A	0.782516	67
V$GATA3_01	GATA-3	GATA3	0.729031	67
V$GR_01	GR	NR3C1	0.051421	62
V$GATA2_01	GATA-2	GATA2	0.491094	62
V$PBX1_04	Pbx1	PBX1	0.0664494	58
V$TCF4_01	TCF-4	TCF7L2	0.291219	52
V$CREM_Q6	CREM	CREM	0.385604	49
V$PIT1_Q6	Pit-1	POU1F1	0.0117583	41
V$YY1_02	YY1	YY1	0.323929	41
V$GABPBETA_Q3	GABP-beta	GABPB1	0.153601	40
V$CEBPB_02	C/EBPbeta	CEBPB	0.516703	34
V$ATF3_Q6_01	ATF-3	ATF3	0.437817	34
V$ELK1_01	Elk-1	ELK1	0.129753	29
V$ATF4_Q6	ATF-4	ATF4	0.0853371	28
V$SMAD4_Q6	SMAD4	SMAD4	0.152645	20
V$MAFK_Q3	MafK	MAFK	0.340169	16

----

hsa-mir-500a

Matrix_id	transcription factor	Gene	PCC	Occurrence
V$PUR1_Q4	PUR1	PURA	0.132303	32
V$GKLF_Q4	GKLF	KLF4	0.232327	31
V$PARP_Q4	PARP	PARP1	0.0871839	31
V$P300_01	p300	EP300	0.295486	30
V$MAFB_01	MAFB	MAFB	0.189739	30
V$SMAD4_Q6_01	Smad4	SMAD4	0.232933	29
V$ELF1_Q6	Elf-1	ELF1	0.204915	29
V$YY1_01	YY1	YY1	0.191736	28
V$ZIC3_01	Zic3	ZIC3	0.40935	27
V$TBP_Q6	TBP	TBP	0.106919	27
V$CDX2_Q5_02	CDX-2	CDX2	0.0315046	27
V$SMAD3_Q6_01	Smad3	SMAD3	0.0776807	26
V$NR1B2_Q6	NR1B2	RARB	0.178504	26
V$GR_Q6	GR	NR3C1	0.173327	25
V$TBX5_02	TBX5	TBX5	0.0233848	24
V$AP4_Q6_02	AP-4	TFAP4	0.0908161	24

V$YY1_Q6	YY1	YY1	0.191736	24
V$YY1_Q6_02	YY1	YY1	0.191736	24
V$HNF4A_Q6_01	HNF-4alpha	HNF4A	0.359395	24
V$CRX_Q4	Crx	CRX	0.184091	23
V$IPF1_Q4_01	IPF1	PDX1	0.025169	23
V$CDX2_Q5_01	Cdx-2	CDX2	0.0315046	23
V$IPF1_Q6	IPF1	PDX1	0.025169	23
V$SOX5_01	SOX5	SOX5	0.111776	22
V$SP1_Q6	Sp1	SP1	0.140344	22
V$PBX1_04	Pbx1	PBX1	0.0763577	22
V$DLX5_01	dlx5	DLX5	0.540294	22
V$GATA2_02	GATA-2	GATA2	0.433401	22
V$PITX2_01	PITX2	PITX2	0.229599	21
V$AHR_Q5	AhR	AHR	0.48884	21
V$IPF1_01	IPF1	PDX1	0.025169	21
V$GATA3_02	GATA-3	GATA3	0.651143	20
V$PITX2_Q2	Pitx2	PITX2	0.229599	20
V$SP1_Q4_01	Sp1	SP1	0.140344	19
V$SP1_Q6_01	Sp1	SP1	0.140344	19
V$WT1_Q6_01	WT1	WT1	0.0330903	19
V$YY1_Q6_03	YY1	YY1	0.191736	19
V$CRX_02	Crx	CRX	0.184091	19
V$HMGIY_01	HMGIY	HMGA1	0.145345	18
V$IPF1_Q4	IPF1	PDX1	0.025169	18
V$SP1_Q2_01	Sp1	SP1	0.140344	18
V$CEBPB_02	C/EBPbeta	CEBPB	0.188735	18
V$TBX5_01	TBX5	TBX5	0.0233848	18
V$CDX2_01	Cdx-2	CDX2	0.0315046	17
V$SP1_02	SP1	SP1	0.140344	17
V$PARP_Q3	PARP	PARP1	0.0871839	17
V$TCF4_Q5	TCF-4	TCF7L2	0.250641	16
V$TCF4_01	TCF-4	TCF7L2	0.250641	15
V$CRX_Q4_01	CRX	CRX	0.184091	15
V$ARNT_01	Arnt	ARNT	0.311153	15
V$RFX1_02	RFX1	RFX1	0.0131056	15
V$SMAD3_Q6	SMAD3	SMAD3	0.0776807	15
V$GATA3_01	GATA-3	GATA3	0.651143	15
V$AP2ALPHA_01	AP-2alpha	TFAP2A	0.59942	14
V$SREBP1_Q6	SREBP-1	SREBF1	0.0818041	14
V$HOXD9_Q2	Hoxd9	HOXD9	0.0378089	13
V$GATA2_03	GATA-2	GATA2	0.433401	13
V$GATA3_03	GATA-3	GATA3	0.651143	12
V$AP2GAMMA_01	AP-2gamma	TFAP2C	0.464678	12
V$ZABC1_01	ZABC1	ZNF217	0.448698	12
V$LHX3b_01	LHX3b	LHX3	0.0129278	12
V$YY1_02	YY1	YY1	0.191736	11

V$SP2_01	SP2	SP2	0.0845856	11
V$HOXA9_01	hoxa9	HOXA9	0.33191	11
V$FOXJ2_01	FOXJ2	FOXJ2	0.0548937	11
V$CP2_01	CP2	TFCP2	0.590002	11
V$GATA2_01	GATA-2	GATA2	0.433401	11
V$TEF1_Q6	TEF-1	TEAD1	0.0272431	11
V$TEF1_Q6_03	TEF-1	TEAD1	0.0272431	11
V$IPF1_02	IPF1	PDX1	0.025169	10
V$E2F1_Q3_01	E2F-1	E2F1	0.042001	10
V$FOXO4_02	FOXO4	FOXO4	0.63219	9
V$CEBPG_Q6_01	C/EBPgamma	CEBPG	0.132434	9
V$POU6F1_03	POU6F1	POU6F1	0.257002	8
V$SOX2_Q6	SOX2	SOX2	0.0673171	8
V$CART1_02	CART1	ALX1	0.122673	7
V$IPF1_06	ipf1	PDX1	0.025169	6
V$YY1_03	YY1	YY1	0.191736	6
V$STAT4_Q5	STAT4	STAT4	0.0214643	6
V$POU6F1_02	POU6F1	POU6F1	0.257002	5
V$IPF1_03	IPF1	PDX1	0.025169	5
V$HLF_01	HLF	HLF	0.142828	4
V$CDP_04	CDP	CUX1	0.266313	3
V$NRF1_Q6	NRF-1	NRF1	0.0662365	3

hsa-mir-501

Matrix_id	transcription factor	Gene	PCC	Occurrence
V$ZIC3_01	Zic3	ZIC3	0.31427	4
V$SOX9_Q4	SOX9	SOX9	0.0893275	4
V$GKLF_Q4	GKLF	KLF4	0.0926548	4
V$HIF1A_Q6	HIF-1alpha	HIF1A	0.431904	4
V$ARNT_01	Arnt	ARNT	0.00314431	4
V$NR1B2_Q6	NR1B2	RARB	0.140588	4
V$PARP_Q4	PARP	PARP1	0.0655986	4
V$GATA3_01	GATA-3	GATA3	0.124143	4
V$TBP_Q6	TBP	TBP	0.113966	3
V$PARP_Q3	PARP	PARP1	0.0655986	3
V$CRX_Q4_01	CRX	CRX	0.180587	3
V$P300_01	p300	EP300	0.016275	3
V$TCF4_01	TCF-4	TCF7L2	0.0549985	3
V$IRF8_Q6	IRF-8	IRF8	0.067542	3
V$HOX13_02	HOXA5	HOXA5	0.445259	3
V$E2F1_Q3_01	E2F-1	E2F1	0.233106	3
V$SREBP1_Q6	SREBP-1	SREBF1	0.390022	3

V$TCF4_Q5	TCF-4	TCF7L2	0.0549985	3
V$AP2ALPHA_01	AP-2alpha	TFAP2A	0.0817444	3
V$GATA6_01	GATA-6	GATA6	0.195242	3
V$AP4_Q6_02	AP-4	TFAP4	0.153962	3
V$GATA3_02	GATA-3	GATA3	0.124143	3
V$DLX5_01	dlx5	DLX5	0.381536	3
V$PITX3_Q2	PITX3	PITX3	0.159132	2
V$HNF4A_Q6_01	HNF-4alpha	HNF4A	0.454403	2
V$MEF2C_Q4	MEF-2C	MEF2C	0.0227768	2
V$FOXJ2_01	FOXJ2	FOXJ2	0.136857	2
V$SOX9_B1	SOX9	SOX9	0.0893275	2
V$CRX_Q4	Crx	CRX	0.180587	2
V$CRX_02	Crx	CRX	0.180587	2
V$PBX1_04	Pbx1	PBX1	0.243288	2
V$CP2_01	CP2	TFCP2	0.428566	2
V$CART1_02	CART1	ALX1	0.113906	2
V$SOX5_01	SOX5	SOX5	0.109682	2
V$NKX2B_Q3	NKX2B	NKX2-2	0.0237923	1
V$GATA3_03	GATA-3	GATA3	0.124143	1
V$NKX22_02	NKX2B	NKX2-2	0.0237923	1
V$HLF_01	HLF	HLF	0.244499	1
V$FOXO4_02	FOXO4	FOXO4	0.183671	1
V$HOXD9_Q2	Hoxd9	HOXD9	0.0178621	1
V$SOX2_Q6	SOX2	SOX2	0.0901642	1
V$POU6F1_03	POU6F1	POU6F1	0.222141	1
V$POU6F1_02	POU6F1	POU6F1	0.222141	1
V$HOXA9_01	hoxa9	HOXA9	0.149993	1
V$STAT4_Q5	STAT4	STAT4	0.0156793	1

---

hsa-mir-502

Matrix_id	transcription factor	Gene	PCC	Occurrence
V$GATA1_01	GATA-1	GATA1	0.0780276	6
V$ZIC3_01	Zic3	ZIC3	0.190632	6
V$AP2REP_01	AP-2rep	KLF12	0.0772799	6
V$HNF3A_01	HNF3A	FOXA1	0.122196	6
V$PUR1_Q4	PUR1	PURA	0.313241	6
V$GKLF_Q4	GKLF	KLF4	0.35021	6
V$AML1_Q6	AML1	RUNX1	0.107093	6
V$IK_Q5	Ikaros	IKZF1	0.0159994	6
V$PBX1_04	Pbx1	PBX1	0.16298	6
V$PARP_Q4	PARP	PARP1	0.124271	6
V$AHR_Q5	AhR	AHR	0.62425	5

V$SP1_02	SP1	SP1	0.412709	5
V$TBX5_02	TBX5	TBX5	0.228665	5
V$E2F1_Q3_01	E2F-1	E2F1	0.0581372	5
V$SP1_Q2_01	Sp1	SP1	0.412709	5
V$TCF4_01	TCF-4	TCF7L2	0.450467	5
V$NANOG_02	Nanog	NANOG	0.213604	5
V$BEN_01	BEN	GTF2IRD1	0.173788	5
V$IPF1_01	IPF1	PDX1	0.225111	5
V$SP1_Q6_01	Sp1	SP1	0.412709	5
V$TEF1_Q6	TEF-1	TEAD1	0.264361	5
V$SP1_Q4_01	Sp1	SP1	0.412709	5
V$P300_01	p300	EP300	0.465047	5
V$GATA1_04	GATA-1	GATA1	0.0780276	5
V$GR_Q6	GR	NR3C1	0.383087	5
V$GATA1_02	GATA-1	GATA1	0.0780276	5
V$SP1_Q6	Sp1	SP1	0.412709	5
V$NFAT4_Q3	NF-AT4	NFATC3	0.0766737	5
V$ARNT_01	Arnt	ARNT	0.501711	5
V$SMAD4_Q6_01	Smad4	SMAD4	0.543116	5
V$DLX5_01	dlx5	DLX5	0.690418	5
V$NKX32_01	Nkx3-2	NKX3-2	0.24046	4
V$SMAD3_Q6_01	Smad3	SMAD3	0.183914	4
V$WT1_Q6_01	WT1	WT1	0.445965	4
V$CDX2_Q5_02	CDX-2	CDX2	0.175389	4
V$NR1B2_Q6	NR1B2	RARB	0.181561	4
V$CART1_02	CART1	ALX1	0.213308	4
V$PARP_Q3	PARP	PARP1	0.124271	4
V$IPF1_02	IPF1	PDX1	0.225111	4
V$IPF1_03	IPF1	PDX1	0.225111	4
V$TEF1_Q6_03	TEF-1	TEAD1	0.264361	4
V$SP2_01	SP2	SP2	0.0983049	4
V$IPF1_Q6	IPF1	PDX1	0.225111	4
V$CDX2_Q5_01	Cdx-2	CDX2	0.175389	4
V$IPF1_06	ipf1	PDX1	0.225111	4
V$TBX5_01	TBX5	TBX5	0.228665	4
V$IPF1_Q4_01	IPF1	PDX1	0.225111	4
V$GATA2_01	GATA-2	GATA2	0.734581	4
V$IPF1_Q4	IPF1	PDX1	0.225111	4
V$SRY_02	SRY	SRY	0.33211	4
V$GATA1_05	GATA-1	GATA1	0.0780276	4
V$GATA3_02	GATA-3	GATA3	0.655805	4
V$GATA2_02	GATA-2	GATA2	0.734581	4
V$CRX_Q4	Crx	CRX	0.125065	4
V$AP2ALPHA_01	AP-2alpha	TFAP2A	0.719629	4
V$TCF4_Q5	TCF-4	TCF7L2	0.450467	4
V$GATA1_06	GATA-1	GATA1	0.0780276	4

V$YY1_01	YY1	YY1	0.325653	4
V$SOX5_01	SOX5	SOX5	0.264399	4
V$TBP_Q6	TBP	TBP	0.377506	4
V$GATA3_01	GATA-3	GATA3	0.655805	4
V$GATA2_03	GATA-2	GATA2	0.734581	3
V$CDX2_01	Cdx-2	CDX2	0.175389	3
V$ZBP89_Q4	ZBP89	ZNF148	0.131298	3
V$RFX1_02	RFX1	RFX1	0.176992	3
V$NKX32_02	Nkx3-2	NKX3-2	0.24046	3
V$HMGIY_01	HMGIY	HMGA1	0.18199	3
V$AP4_Q6_02	AP-4	TFAP4	0.0731815	3
V$CRX_Q4_01	CRX	CRX	0.125065	3
V$SMAD3_Q6	SMAD3	SMAD3	0.183914	3
V$FOXO4_02	FOXO4	FOXO4	0.628571	3
V$AP2GAMMA_01	AP-2gamma	TFAP2C	0.71921	3
V$YY1_Q6_03	YY1	YY1	0.325653	3
V$FOXJ2_01	FOXJ2	FOXJ2	0.22841	3
V$CEBPB_02	C/EBPbeta	CEBPB	0.302265	3
V$CP2_01	CP2	TFCP2	0.351751	3
V$NFAT2_Q5	NF-AT2	NFATC1	0.143232	3
V$LHX3b_01	LHX3b	LHX3	0.145688	2
V$STAT4_Q5	STAT4	STAT4	0.164764	2
V$BCL6_Q3_01	Bcl-6	BCL6	0.186492	2
V$NCX_02	Ncx	TLX2	0.00159238	2
V$YY1_Q6_02	YY1	YY1	0.325653	2
V$YY1_Q6	YY1	YY1	0.325653	2
V$PITX2_Q2	Pitx2	PITX2	0.544399	2
V$HOXA9_01	hoxa9	HOXA9	0.415816	2
V$GATA3_03	GATA-3	GATA3	0.655805	2
V$CDX1_01	Cdx-1	CDX1	0.0722411	2
V$POU6F1_02	POU6F1	POU6F1	0.0621987	2
V$PITX2_01	PITX2	PITX2	0.544399	2
V$POU6F1_03	POU6F1	POU6F1	0.0621987	2
V$PITX1_01	Pitx1	PITX1	0.168749	1
V$BACH2_01	Bach2	BACH2	0.143896	1
V$YY1_03	YY1	YY1	0.325653	1
V$YY1_02	YY1	YY1	0.325653	1
V$CRX_02	Crx	CRX	0.125065	1
V$NRF1_Q6	NRF-1	NRF1	0.247564	1
V$ZABC1_01	ZABC1	ZNF217	0.649136	1
V$CDP_04	CDP	CUX1	0.43184	1
V$HLF_01	HLF	HLF	0.032312	1
V$HOXD9_Q2	Hoxd9	HOXD9	0.216455	1
V$CEBPG_Q6_01	C/EBPgamma	CEBPG	0.398984	1

----

hsa-mir-503

Matrix_id	transcription factor	Gene	PCC	Occurrence
V$PUR1_Q4	PUR1	PURA	0.16005	263
V$PEA3_Q6	PEA3	ETV4	0.0983111	257
V$PARP_Q4	PARP	PARP1	0.0995149	254
V$ELF1_Q6	Elf-1	ELF1	0.165773	249
V$GKLF_Q4	GKLF	KLF4	0.567492	246
V$MAFB_01	MAFB	MAFB	0.300398	241
V$ETS1_B	c-Ets-1	ETS1	0.0679363	230
V$CETS1_Q6	C-ets-1	ETS1	0.0679363	224
V$SMAD4_Q6_01	Smad4	SMAD4	0.308843	223
V$GATA1_01	GATA-1	GATA1	0.0170848	213
V$GABPA_Q4	GABP-alpha	GABPA	0.364776	212
V$TBP_Q6	TBP	TBP	0.156746	202
V$GR_Q6	GR	NR3C1	0.157584	201
V$YY1_01	YY1	YY1	0.367543	197
V$AML1_Q6	AML1	RUNX1	0.00174447	188
V$DLX5_01	dlx5	DLX5	0.691609	178
V$YY1_Q6_02	YY1	YY1	0.367543	176
V$YY1_Q6	YY1	YY1	0.367543	176
V$GATA1_02	GATA-1	GATA1	0.0170848	173
V$GATA1_05	GATA-1	GATA1	0.0170848	165
V$GATA1_06	GATA-1	GATA1	0.0170848	165
V$GATA2_02	GATA-2	GATA2	0.695429	165
V$ERBETA_Q5	ER-beta	ESR2	0.0421464	160
V$PARP_Q3	PARP	PARP1	0.0995149	159
V$HMGIY_01	HMGIY	HMGA1	0.341965	145
V$GATA3_02	GATA-3	GATA3	0.912183	141
V$YY1_Q6_03	YY1	YY1	0.367543	132
V$CEBPE_Q6	CEBPE	CEBPE	0.0285527	121
V$GR_01	GR	NR3C1	0.157584	119
V$TCF4_01	TCF-4	TCF7L2	0.46753	118
V$CEBPA_Q6	C/EBPalpha	CEBPA	0.0713893	117
V$TCF4_Q5	TCF-4	TCF7L2	0.46753	111
V$IRF7_Q3	IRF-7	IRF7	0.130981	107
V$FOXJ2_01	FOXJ2	FOXJ2	0.0461565	106
V$CREM_Q6	CREM	CREM	0.172813	98
V$GABPBETA_Q3	GABP-beta	GABPB1	0.228391	96
V$HBP1_Q2	hbp1	HBP1	0.668623	90
V$CEBPG_Q6_01	C/EBPgamma	CEBPG	0.547814	82
V$GATA2_03	GATA-2	GATA2	0.695429	77
V$STAT3_03	STAT3	STAT3	0.146628	76
V$FOXO4_02	FOXO4	FOXO4	0.956047	75

V$YY1_02	YY1	YY1	0.367543	74
V$CEBPA_01	C/EBPalpha	CEBPA	0.0713893	72
V$P53_02	p53	TP53	0.605859	66
V$E2F4_Q6	E2F-4	E2F4	0.0663263	65
V$ELK1_01	Elk-1	ELK1	0.0202257	63
V$CIZ_01	CIZ	ZNF384	0.0830076	56
V$IRF1_Q6_01	IRF-1	IRF1	0.338402	49
V$CEBPG_Q6	C/EBPgamma	CEBPG	0.547814	46
V$IRF1_Q6	IRF-1	IRF1	0.338402	44
V$CDP_04	CDP	CUX1	0.363955	38
V$FOXO4_01	FOXO4	FOXO4	0.956047	35
V$FOXJ2_02	FOXJ2	FOXJ2	0.0461565	30
V$TCF4_Q5_01	TCF-4	TCF7L2	0.46753	18

----

hsa-mir-505

Matrix_id	transcription factor	Gene	PCC	Occurrence
V$SREBP1_Q6	SREBP-1	SREBF1	0.368536	2
V$MATH1_Q2	MATH1	ATOH1	0.180232	2
V$MAFB_01	MAFB	MAFB	0.152178	2
V$HNF3A_01	HNF3A	FOXA1	0.279508	2
V$HNF3B_Q6	HNF-3beta	FOXA2	0.024055	2
V$HOXA9_01	hoxa9	HOXA9	0.0271782	2
V$SMAD3_Q6_01	Smad3	SMAD3	0.0594151	2
V$ERBETA_Q5	ER-beta	ESR2	0.0475718	2
V$DBP_Q6_01	DBP	DBP	0.00534351	2
V$CDX2_01	Cdx-2	CDX2	0.00641135	2
V$FKLF_Q5	FKLF	KLF11	0.169834	2
V$CDX2_Q5_01	Cdx-2	CDX2	0.00641135	2
V$PUR1_Q4	PUR1	PURA	0.0287421	2
V$HOXA7_01	HOXA7	HOXA7	0.272034	2
V$MYOD_Q6_01	MyoD	MYOD1	0.117618	2
V$HNF6_Q6	HNF6	ONECUT1	0.580756	2
V$PBX1_Q3	Pbx1	PBX1	0.0769166	2
V$SOX9_B1	SOX9	SOX9	0.0697598	2
V$NR1B2_Q6	NR1B2	RARB	0.0845622	2
V$MYOD_Q6	MyoD	MYOD1	0.117618	2
V$SMAD3_Q6	SMAD3	SMAD3	0.0594151	2
V$MYOD_Q6_02	MyoD	MYOD1	0.117618	2
V$ELF1_Q6	Elf-1	ELF1	0.358206	2
V$CDX2_Q5_02	CDX-2	CDX2	0.00641135	2
V$OCAB_Q6	OCA-B	POU2AF1	0.157388	2
V$GATA6_01	GATA-6	GATA6	0.126434	2

V$EAR2_Q2	EAR2	NR2F6	0.340353	1
V$PITX3_Q2	PITX3	PITX3	0.146355	1
V$OC2_Q3	OC-2	ONECUT2	0.0772972	1
V$Elf5_03	ELF5	ELF5	0.389197	1
V$CEBPB_Q6	C/EBPbeta	CEBPB	0.00754331	1
V$HIF1A_Q6	HIF-1alpha	HIF1A	0.237978	1
V$HNF4A_Q6_01	HNF-4alpha	HNF4A	0.257157	1
V$CRX_Q4	Crx	CRX	0.15804	1
V$MAZ_Q6_01	MAZ	MAZ	0.0974725	1
V$CRX_Q4_01	CRX	CRX	0.15804	1
V$NURR1_Q3	NURR1	NR4A2	0.415395	1
V$ELF5_01	ELF5	ELF5	0.389197	1
V$RORA_Q4	RORalpha	RORA	0.371331	1
V$PIT1_Q6	Pit-1	POU1F1	0.203055	1
V$CEBPB_01	C/EBPbeta	CEBPB	0.00754331	1
V$CDX2_Q5	Cdx-2	CDX2	0.00641135	1
V$TCF4_Q5	TCF-4	TCF7L2	0.0111451	1
V$MEIS1_01	MEIS1	MEIS1	0.249875	1
V$SOX2_Q6	SOX2	SOX2	0.332306	1
V$SOX9_Q4	SOX9	SOX9	0.0697598	1
V$TCF4_01	TCF-4	TCF7L2	0.0111451	1
V$IRF8_Q6	IRF-8	IRF8	0.0197326	1
V$GABPA_Q4	GABP-alpha	GABPA	0.152094	1
V$TCF3_01	TCF-3	TCF7L1	0.217092	1
V$CRX_02	Crx	CRX	0.15804	1
V$MEIS1_02	Meis1	MEIS1	0.249875	1
V$CDX1_01	Cdx-1	CDX1	0.366999	1
V$NEUROD_02	NeuroD	NEUROD1	0.00387019	1
V$CEBPA_01	C/EBPalpha	CEBPA	0.617238	1

----

hsa-mir-506

Matrix_id	transcription factor	Gene	PCC	Occurrence
V$GKLF_Q4	GKLF	KLF4	0.12645	337
V$AP2REP_01	AP-2rep	KLF12	0.106276	337
V$PEA3_Q6	PEA3	ETV4	0.1091	333
V$P300_01	p300	EP300	0.0552819	324
V$ZIC3_01	Zic3	ZIC3	0.3663	299
V$SOX9_Q4	SOX9	SOX9	0.643662	294
V$GATA1_01	GATA-1	GATA1	0.144191	288
V$CETS1_Q6	C-ets-1	ETS1	0.150928	287
V$NFAT4_Q3	NF-AT4	NFATC3	0.0723661	283
V$ETS1_B	c-Ets-1	ETS1	0.150928	282

V$YY1_01	YY1	YY1	0.154561	281
V$GABPA_Q4	GABP-alpha	GABPA	0.0230381	276
V$TBX5_02	TBX5	TBX5	0.020796	274
V$HNF4A_Q6_01	HNF-4alpha	HNF4A	0.0276837	269
V$YY1_Q6	YY1	YY1	0.154561	254
V$AML1_Q6	AML1	RUNX1	0.170594	253
V$YY1_Q6_02	YY1	YY1	0.154561	243
V$SP1_Q6	Sp1	SP1	0.0227717	236
V$NKX32_01	Nkx3-2	NKX3-2	0.316326	225
V$TBX5_01	TBX5	TBX5	0.020796	224
V$PITX3_Q2	PITX3	PITX3	0.0656226	220
V$MAZ_Q6	MAZ	MAZ	0.379129	219
V$ERBETA_Q5	ER-beta	ESR2	0.257076	217
V$E12_Q6	E12	TCF3	0.422671	216
V$E2A_Q6	E2A	TCF3	0.422671	216
V$E47_02	E47	TCF3	0.422671	215
V$SP1_01	Sp1	SP1	0.0227717	208
V$ING4_01	ING4	ING4	0.0891331	207
V$ELK1_02	Elk-1	ELK1	0.274016	206
V$SP1_Q6_01	Sp1	SP1	0.0227717	204
V$SP1_Q4_01	Sp1	SP1	0.0227717	198
V$HNF3B_Q6	HNF-3beta	FOXA2	0.0605376	196
V$ELK1_06	ELK-1	ELK1	0.274016	193
V$SP1_Q2_01	Sp1	SP1	0.0227717	190
V$SP1_02	SP1	SP1	0.0227717	184
V$ERF_02	ERF	ERF	0.00326475	179
V$CREM_Q6	CREM	CREM	0.553724	173
V$MYOGENIN_Q6	myogenin	MYOG	0.181789	172
V$E2A_Q2	E2A	TCF3	0.422671	162
V$SREBP1_Q6	SREBP-1	SREBF1	0.190199	154
V$FKLF_Q5	FKLF	KLF11	0.31863	153
V$FOXP3_01	FOXP3	FOXP3	0.0202327	150
V$GABPBETA_Q3	GABP-beta	GABPB1	0.157454	147
V$AML1_01	AML1a	RUNX1	0.170594	145
V$AML1_Q4	AML1	RUNX1	0.170594	132
V$WT1_Q6	WT1	WT1	0.206392	131
V$MAZ_Q6_01	MAZ	MAZ	0.379129	126
V$YY1_02	YY1	YY1	0.154561	122
V$E47_01	E47	TCF3	0.422671	121
V$DAX1_01	Dax1	NR0B1	0.584553	118
V$MYOGENIN_Q6_01	myogenin	MYOG	0.181789	113
V$ERM_02	Erm	ETV5	0.373026	95
V$ELK1_01	Elk-1	ELK1	0.274016	93
V$SPIB_03	Spi-B	SPIB	0.0291967	85
V$MATH1_Q2	MATH1	ATOH1	0.187522	78
V$EGR2_01	Egr-2	EGR2	0.267075	51

V$SREBP1_02	SREBP-1	SREBF1	0.190199	29
V$GLI3_Q5_01	GLI3	GLI3	0.00061332	29

----

hsa-mir-507

Matrix_id	transcription factor	Gene	PCC	Occurrence
V$AP2REP_01	AP-2rep	KLF12	0.104783	339
V$GKLF_Q4	GKLF	KLF4	0.135731	338
V$PEA3_Q6	PEA3	ETV4	0.105244	335
V$P300_01	p300	EP300	0.06091	326
V$ZIC3_01	Zic3	ZIC3	0.364024	300
V$SOX9_Q4	SOX9	SOX9	0.650877	295
V$GATA1_01	GATA-1	GATA1	0.166628	290
V$CETS1_Q6	C-ets-1	ETS1	0.169672	289
V$NFAT4_Q3	NF-AT4	NFATC3	0.0812033	283
V$ETS1_B	c-Ets-1	ETS1	0.169672	282
V$YY1_01	YY1	YY1	0.158969	282
V$TBX5_02	TBX5	TBX5	0.0305597	277
V$GABPA_Q4	GABP-alpha	GABPA	0.0261348	276
V$HNF4A_Q6_01	HNF-4alpha	HNF4A	0.00676163	270
V$YY1_Q6	YY1	YY1	0.158969	257
V$AML1_Q6	AML1	RUNX1	0.17817	252
V$YY1_Q6_02	YY1	YY1	0.158969	245
V$SP1_Q6	Sp1	SP1	0.0292724	240
V$NKX32_01	Nkx3-2	NKX3-2	0.32374	227
V$TBX5_01	TBX5	TBX5	0.0305597	225
V$PITX3_Q2	PITX3	PITX3	0.0419136	221
V$MAZ_Q6	MAZ	MAZ	0.380747	221
V$E12_Q6	E12	TCF3	0.433743	218
V$ERBETA_Q5	ER-beta	ESR2	0.256708	218
V$E2A_Q6	E2A	TCF3	0.433743	218
V$E47_02	E47	TCF3	0.433743	217
V$SP1_01	Sp1	SP1	0.0292724	212
V$ING4_01	ING4	ING4	0.100046	208
V$SP1_Q6_01	Sp1	SP1	0.0292724	208
V$ELK1_02	Elk-1	ELK1	0.259013	207
V$SP1_Q4_01	Sp1	SP1	0.0292724	202
V$HNF3B_Q6	HNF-3beta	FOXA2	0.0794719	197
V$ELK1_06	ELK-1	ELK1	0.259013	194
V$SP1_Q2_01	Sp1	SP1	0.0292724	194
V$SP1_02	SP1	SP1	0.0292724	189
V$ERF_02	ERF	ERF	0.0218276	180
V$MYOGENIN_Q6	myogenin	MYOG	0.182505	174

V$CREM_Q6	CREM	CREM	0.528217	174
V$E2A_Q2	E2A	TCF3	0.433743	164
V$FKLF_Q5	FKLF	KLF11	0.314366	155
V$SREBP1_Q6	SREBP-1	SREBF1	0.149918	154
V$FOXP3_01	FOXP3	FOXP3	0.0130914	152
V$GABPBETA_Q3	GABP-beta	GABPB1	0.156045	147
V$AML1_01	AML1a	RUNX1	0.17817	144
V$WT1_Q6	WT1	WT1	0.209998	132
V$AML1_Q4	AML1	RUNX1	0.17817	131
V$MAZ_Q6_01	MAZ	MAZ	0.380747	127
V$E47_01	E47	TCF3	0.433743	123
V$YY1_02	YY1	YY1	0.158969	123
V$DAX1_01	Dax1	NR0B1	0.569677	118
V$MYOGENIN_Q6_01	myogenin	MYOG	0.182505	112
V$ERM_02	Erm	ETV5	0.353231	97
V$ELK1_01	Elk-1	ELK1	0.259013	94
V$SPIB_03	Spi-B	SPIB	0.0443451	85
V$MATH1_Q2	MATH1	ATOH1	0.169532	78
V$EGR2_01	Egr-2	EGR2	0.284316	53
V$GLI3_Q5_01	GLI3	GLI3	0.00951285	31
V$SREBP1_02	SREBP-1	SREBF1	0.149918	28

hsa-mir-508

Matrix_id	transcription factor	Gene	PCC	Occurrence
V$GKLF_Q4	GKLF	KLF4	0.11594	327
V$AP2REP_01	AP-2rep	KLF12	0.0991236	327
V$PEA3_Q6	PEA3	ETV4	0.114528	322
V$PARP_Q4	PARP	PARP1	0.180163	322
V$P300_01	p300	EP300	0.0407174	315
V$ZIC3_01	Zic3	ZIC3	0.350747	290
V$TBP_Q6	TBP	TBP	0.803342	288
V$SOX9_Q4	SOX9	SOX9	0.63011	283
V$CETS1_Q6	C-ets-1	ETS1	0.126874	276
V$NFAT4_Q3	NF-AT4	NFATC3	0.0574944	273
V$YY1_01	YY1	YY1	0.135432	273
V$ETS1_B	c-Ets-1	ETS1	0.126874	271
V$GABPA_Q4	GABP-alpha	GABPA	0.0102325	267
V$TBX5_02	TBX5	TBX5	0.010328	267
V$HNF4A_Q6_01	HNF-4alpha	HNF4A	0.0239438	261
V$DLX5_01	dlx5	DLX5	0.305241	258
V$SOX5_01	SOX5	SOX5	0.621603	251
V$SOX9_B1	SOX9	SOX9	0.63011	250

V$YY1_Q6	YY1	YY1	0.135432	246
V$AML1_Q6	AML1	RUNX1	0.156987	244
V$YY1_Q6_02	YY1	YY1	0.135432	235
V$GATA6_01	GATA-6	GATA6	0.00267406	223
V$GATA1_06	GATA-1	GATA1	0.120545	223
V$GATA1_05	GATA-1	GATA1	0.120545	223
V$SRY_02	SRY	SRY	0.099541	221
V$GATA1_02	GATA-1	GATA1	0.120545	220
V$TBX5_01	TBX5	TBX5	0.010328	218
V$PITX3_Q2	PITX3	PITX3	0.0720997	214
V$E47_02	E47	TCF3	0.406131	212
V$E12_Q6	E12	TCF3	0.406131	212
V$E2A_Q6	E2A	TCF3	0.406131	212
V$HMGIY_01	HMGIY	HMGA1	0.604314	203
V$ING4_01	ING4	ING4	0.075464	199
V$HNF3B_Q6	HNF-3beta	FOXA2	0.0369565	192
V$PARP_Q3	PARP	PARP1	0.180163	188
V$GATA1_04	GATA-1	GATA1	0.120545	178
V$FOXJ2_01	FOXJ2	FOXJ2	0.278795	167
V$MYOGENIN_Q6	myogenin	MYOG	0.1752	167
V$CREM_Q6	CREM	CREM	0.56577	165
V$FOXM1_01	FOXM1	FOXM1	0.799265	162
V$E2A_Q2	E2A	TCF3	0.406131	159
V$TEF1_Q6_03	TEF-1	TEAD1	0.018304	157
V$TCF4_Q5	TCF-4	TCF7L2	0.0387432	153
V$IRF8_Q6	IRF-8	IRF8	0.0584665	150
V$OC2_Q3	OC-2	ONECUT2	0.0128522	149
V$FOXP3_01	FOXP3	FOXP3	0.0206458	146
V$TEF1_Q6	TEF-1	TEAD1	0.018304	145
V$TCF4_01	TCF-4	TCF7L2	0.0387432	142
V$AML1_01	AML1a	RUNX1	0.156987	141
V$YY1_02	YY1	YY1	0.135432	119
V$E47_01	E47	TCF3	0.406131	118
V$CDX1_01	Cdx-1	CDX1	0.100143	110
V$MYOGENIN_Q6_01	myogenin	MYOG	0.1752	109
V$CEBPG_Q6_01	C/EBPgamma	CEBPG	0.460806	97
V$CIZ_01	CIZ	ZNF384	0.142596	86
V$HNF6_Q6	HNF6	ONECUT1	0.0505377	84
V$SPIB_01	SPI-B	SPIB	0.0120223	43
V$HSF1_01	HSF1	HSF1	0.190525	33
V$ZID_01	ZID	ZBTB6	0.556456	31
V$HSF2_01	HSF2	HSF2	0.506197	8

----

hsa-mir-509-1

Matrix_id	transcription factor	Gene	PCC	Occurrence
V$AP2REP_01	AP-2rep	KLF12	0.0883173	299
V$GKLF_Q4	GKLF	KLF4	0.100851	297
V$PEA3_Q6	PEA3	ETV4	0.122104	294
V$PARP_Q4	PARP	PARP1	0.156797	294
V$P300_01	p300	EP300	0.0249685	289
V$TBP_Q6	TBP	TBP	0.788573	266
V$SOX9_Q4	SOX9	SOX9	0.60576	256
V$NFAT4_Q3	NF-AT4	NFATC3	0.0352869	252
V$YY1_01	YY1	YY1	0.109669	251
V$ETS1_B	c-Ets-1	ETS1	0.0951685	244
V$HNF4A_Q6_01	HNF-4alpha	HNF4A	0.033607	238
V$DLX5_01	dlx5	DLX5	0.315905	237
V$SOX5_01	SOX5	SOX5	0.609371	227
V$SOX9_B1	SOX9	SOX9	0.60576	226
V$YY1_Q6	YY1	YY1	0.109669	223
V$YY1_Q6_02	YY1	YY1	0.109669	212
V$GATA6_01	GATA-6	GATA6	0.03567	205
V$GATA1_06	GATA-1	GATA1	0.0818286	205
V$GATA1_05	GATA-1	GATA1	0.0818286	205
V$GATA1_02	GATA-1	GATA1	0.0818286	204
V$SRY_02	SRY	SRY	0.0843625	200
V$ERBETA_Q5	ER-beta	ESR2	0.248456	193
V$E2A_Q6	E2A	TCF3	0.381515	191
V$E47_02	E47	TCF3	0.381515	191
V$E12_Q6	E12	TCF3	0.381515	191
V$HMGIY_01	HMGIY	HMGA1	0.574802	187
V$ING4_01	ING4	ING4	0.0567677	185
V$PARP_Q3	PARP	PARP1	0.156797	169
V$GATA1_04	GATA-1	GATA1	0.0818286	165
V$NFAT2_Q5	NF-AT2	NFATC1	0.120848	160
V$YY1_Q6_03	YY1	YY1	0.109669	159
V$MYOGENIN_Q6	myogenin	MYOG	0.170496	153
V$FOXM1_01	FOXM1	FOXM1	0.777291	149
V$FOXJ2_01	FOXJ2	FOXJ2	0.273451	149
V$E2A_Q2	E2A	TCF3	0.381515	144
V$TEF1_Q6_03	TEF-1	TEAD1	0.0151646	144
V$IRF8_Q6	IRF-8	IRF8	0.0456705	140
V$TCF4_01	TCF-4	TCF7L2	0.0255368	133
V$FOXP3_01	FOXP3	FOXP3	0.0262323	132
V$TEF1_Q6	TEF-1	TEAD1	0.0151646	131
V$WT1_Q6	WT1	WT1	0.203131	111
V$MAZ_Q6_01	MAZ	MAZ	0.333863	108
V$DAX1_01	Dax1	NR0B1	0.592752	107

V$ZABC1_01	ZABC1	ZNF217	0.0911128	101
V$CDX1_01	Cdx-1	CDX1	0.0912888	100
V$MYOGENIN_Q6_01	myogenin	MYOG	0.170496	99
V$CEBPG_Q6_01	C/EBPgamma	CEBPG	0.4263	84
V$HNF6_Q6	HNF6	ONECUT1	0.0872916	79
V$CIZ_01	CIZ	ZNF384	0.151928	77
V$TR4_Q2	TR4	NR2C2	0.0944493	76
V$HOX13_02	HOXA5	HOXA5	0.00250656	73
V$YY1_03	YY1	YY1	0.109669	64
V$STAT4_Q5	STAT4	STAT4	0.215562	63
V$HOXB8_01	HOXB8	HOXB8	0.262687	60
V$CEBPG_Q6	C/EBPgamma	CEBPG	0.4263	51
V$HNF1B_01	HNF-1beta	HNF1B	0.0211581	42
V$OCT2_01	2-Oct	POU2F2	0.049126	34
V$RREB1_01	RREB-1	RREB1	0.125111	28
V$RORA1_01	RORalpha1	RORA	0.376472	28
V$NKX3A_02	Nkx3A	NKX3-1	0.0765738	28
V$DMRT1_01	DMRT1	DMRT1	0.951294	9

----

hsa-mir-509-2

Matrix_id	transcription factor	Gene	PCC	Occurrence
V$AP2REP_01	AP-2rep	KLF12	0.0883173	299
V$GKLF_Q4	GKLF	KLF4	0.100851	297
V$PEA3_Q6	PEA3	ETV4	0.122104	294
V$PARP_Q4	PARP	PARP1	0.156797	294
V$P300_01	p300	EP300	0.0249685	289
V$TBP_Q6	TBP	TBP	0.788573	266
V$SOX9_Q4	SOX9	SOX9	0.60576	256
V$NFAT4_Q3	NF-AT4	NFATC3	0.0352869	252
V$YY1_01	YY1	YY1	0.109669	251
V$ETS1_B	c-Ets-1	ETS1	0.0951685	244
V$HNF4A_Q6_01	HNF-4alpha	HNF4A	0.033607	238
V$DLX5_01	dlx5	DLX5	0.315905	237
V$SOX5_01	SOX5	SOX5	0.609371	227
V$SOX9_B1	SOX9	SOX9	0.60576	226
V$YY1_Q6	YY1	YY1	0.109669	223
V$YY1_Q6_02	YY1	YY1	0.109669	212
V$GATA6_01	GATA-6	GATA6	0.03567	205
V$GATA1_06	GATA-1	GATA1	0.0818286	205
V$GATA1_05	GATA-1	GATA1	0.0818286	205
V$GATA1_02	GATA-1	GATA1	0.0818286	204
V$SRY_02	SRY	SRY	0.0843625	200

V$ERBETA_Q5	ER-beta	ESR2	0.248456	193
V$E2A_Q6	E2A	TCF3	0.381515	191
V$E47_02	E47	TCF3	0.381515	191
V$E12_Q6	E12	TCF3	0.381515	191
V$HMGIY_01	HMGIY	HMGA1	0.574802	187
V$ING4_01	ING4	ING4	0.0567677	185
V$PARP_Q3	PARP	PARP1	0.156797	169
V$GATA1_04	GATA-1	GATA1	0.0818286	165
V$NFAT2_Q5	NF-AT2	NFATC1	0.120848	160
V$YY1_Q6_03	YY1	YY1	0.109669	159
V$MYOGENIN_Q6	myogenin	MYOG	0.170496	153
V$FOXM1_01	FOXM1	FOXM1	0.777291	149
V$FOXJ2_01	FOXJ2	FOXJ2	0.273451	149
V$E2A_Q2	E2A	TCF3	0.381515	144
V$TEF1_Q6_03	TEF-1	TEAD1	0.0151646	144
V$IRF8_Q6	IRF-8	IRF8	0.0456705	140
V$TCF4_01	TCF-4	TCF7L2	0.0255368	133
V$FOXP3_01	FOXP3	FOXP3	0.0262323	132
V$TEF1_Q6	TEF-1	TEAD1	0.0151646	131
V$WT1_Q6	WT1	WT1	0.203131	111
V$MAZ_Q6_01	MAZ	MAZ	0.333863	108
V$DAX1_01	Dax1	NR0B1	0.592752	107
V$ZABC1_01	ZABC1	ZNF217	0.0911128	101
V$CDX1_01	Cdx-1	CDX1	0.0912888	100
V$MYOGENIN_Q6_01	myogenin	MYOG	0.170496	99
V$CEBPG_Q6_01	C/EBPgamma	CEBPG	0.4263	84
V$HNF6_Q6	HNF6	ONECUT1	0.0872916	79
V$CIZ_01	CIZ	ZNF384	0.151928	77
V$TR4_Q2	TR4	NR2C2	0.0944493	76
V$HOX13_02	HOXA5	HOXA5	0.00250656	73
V$YY1_03	YY1	YY1	0.109669	64
V$STAT4_Q5	STAT4	STAT4	0.215562	63
V$HOXB8_01	HOXB8	HOXB8	0.262687	60
V$CEBPG_Q6	C/EBPgamma	CEBPG	0.4263	51
V$HNF1B_01	HNF-1beta	HNF1B	0.0211581	42
V$OCT2_01	2-Oct	POU2F2	0.049126	34
V$RREB1_01	RREB-1	RREB1	0.125111	28
V$RORA1_01	RORalpha1	RORA	0.376472	28
V$NKX3A_02	Nkx3A	NKX3-1	0.0765738	28
V$DMRT1_01	DMRT1	DMRT1	0.951294	9

----

hsa-mir-509-3

Matrix_id	transcription factor	Gene	PCC	Occurrence

V$AP2REP_01	AP-2rep	KLF12	0.0883173	299
V$GKLF_Q4	GKLF	KLF4	0.100851	297
V$PEA3_Q6	PEA3	ETV4	0.122104	294
V$PARP_Q4	PARP	PARP1	0.156797	294
V$P300_01	p300	EP300	0.0249685	289
V$TBP_Q6	TBP	TBP	0.788573	266
V$SOX9_Q4	SOX9	SOX9	0.60576	256
V$NFAT4_Q3	NF-AT4	NFATC3	0.0352869	252
V$YY1_01	YY1	YY1	0.109669	251
V$ETS1_B	c-Ets-1	ETS1	0.0951685	244
V$HNF4A_Q6_01	HNF-4alpha	HNF4A	0.033607	238
V$DLX5_01	dlx5	DLX5	0.315905	237
V$SOX5_01	SOX5	SOX5	0.609371	227
V$SOX9_B1	SOX9	SOX9	0.60576	226
V$YY1_Q6	YY1	YY1	0.109669	223
V$YY1_Q6_02	YY1	YY1	0.109669	212
V$GATA6_01	GATA-6	GATA6	0.03567	205
V$GATA1_06	GATA-1	GATA1	0.0818286	205
V$GATA1_05	GATA-1	GATA1	0.0818286	205
V$GATA1_02	GATA-1	GATA1	0.0818286	204
V$SRY_02	SRY	SRY	0.0843625	200
V$ERBETA_Q5	ER-beta	ESR2	0.248456	193
V$E2A_Q6	E2A	TCF3	0.381515	191
V$E47_02	E47	TCF3	0.381515	191
V$E12_Q6	E12	TCF3	0.381515	191
V$HMGIY_01	HMGIY	HMGA1	0.574802	187
V$ING4_01	ING4	ING4	0.0567677	185
V$PARP_Q3	PARP	PARP1	0.156797	169
V$GATA1_04	GATA-1	GATA1	0.0818286	165
V$NFAT2_Q5	NF-AT2	NFATC1	0.120848	160
V$YY1_Q6_03	YY1	YY1	0.109669	159
V$MYOGENIN_Q6	myogenin	MYOG	0.170496	153
V$FOXM1_01	FOXM1	FOXM1	0.777291	149
V$FOXJ2_01	FOXJ2	FOXJ2	0.273451	149
V$E2A_Q2	E2A	TCF3	0.381515	144
V$TEF1_Q6_03	TEF-1	TEAD1	0.0151646	144
V$IRF8_Q6	IRF-8	IRF8	0.0456705	140
V$TCF4_01	TCF-4	TCF7L2	0.0255368	133
V$FOXP3_01	FOXP3	FOXP3	0.0262323	132
V$TEF1_Q6	TEF-1	TEAD1	0.0151646	131
V$WT1_Q6	WT1	WT1	0.203131	111
V$MAZ_Q6_01	MAZ	MAZ	0.333863	108
V$DAX1_01	Dax1	NR0B1	0.592752	107
V$ZABC1_01	ZABC1	ZNF217	0.0911128	101
V$CDX1_01	Cdx-1	CDX1	0.0912888	100

V$MYOGENIN_Q6_01	myogenin	MYOG	0.170496	99
V$CEBPG_Q6_01	C/EBPgamma	CEBPG	0.4263	84
V$HNF6_Q6	HNF6	ONECUT1	0.0872916	79
V$CIZ_01	CIZ	ZNF384	0.151928	77
V$TR4_Q2	TR4	NR2C2	0.0944493	76
V$HOX13_02	HOXA5	HOXA5	0.00250656	73
V$YY1_03	YY1	YY1	0.109669	64
V$STAT4_Q5	STAT4	STAT4	0.215562	63
V$HOXB8_01	HOXB8	HOXB8	0.262687	60
V$CEBPG_Q6	C/EBPgamma	CEBPG	0.4263	51
V$HNF1B_01	HNF-1beta	HNF1B	0.0211581	42
V$OCT2_01	2-Oct	POU2F2	0.049126	34
V$RREB1_01	RREB-1	RREB1	0.125111	28
V$RORA1_01	RORalpha1	RORA	0.376472	28
V$NKX3A_02	Nkx3A	NKX3-1	0.0765738	28
V$DMRT1_01	DMRT1	DMRT1	0.951294	9

----

hsa-mir-510

Matrix_id	transcription factor	Gene	PCC	Occurrence
V$AP2REP_01	AP-2rep	KLF12	0.100422	332
V$GKLF_Q4	GKLF	KLF4	0.116949	332
V$PEA3_Q6	PEA3	ETV4	0.112262	327
V$PARP_Q4	PARP	PARP1	0.185538	327
V$P300_01	p300	EP300	0.0475435	319
V$TBP_Q6	TBP	TBP	0.807544	292
V$SOX9_Q4	SOX9	SOX9	0.63498	288
V$NFAT4_Q3	NF-AT4	NFATC3	0.0621294	277
V$YY1_01	YY1	YY1	0.139398	277
V$ETS1_B	c-Ets-1	ETS1	0.139731	276
V$HNF4A_Q6_01	HNF-4alpha	HNF4A	0.0348707	265
V$DLX5_01	dlx5	DLX5	0.306603	262
V$SOX5_01	SOX5	SOX5	0.631459	256
V$SOX9_B1	SOX9	SOX9	0.63498	255
V$YY1_Q6	YY1	YY1	0.139398	249
V$YY1_Q6_02	YY1	YY1	0.139398	238
V$GATA1_06	GATA-1	GATA1	0.127105	228
V$GATA1_05	GATA-1	GATA1	0.127105	228
V$SRY_02	SRY	SRY	0.101361	226
V$GATA1_02	GATA-1	GATA1	0.127105	225
V$ERBETA_Q5	ER-beta	ESR2	0.257316	213
V$E2A_Q6	E2A	TCF3	0.414266	213
V$E47_02	E47	TCF3	0.414266	213

V$E12_Q6	E12	TCF3	0.414266	213
V$HMGIY_01	HMGIY	HMGA1	0.610782	206
V$ING4_01	ING4	ING4	0.0815857	202
V$HNF3B_Q6	HNF-3beta	FOXA2	0.0431588	195
V$PARP_Q3	PARP	PARP1	0.185538	190
V$GATA1_04	GATA-1	GATA1	0.127105	183
V$NFAT2_Q5	NF-AT2	NFATC1	0.14101	181
V$YY1_Q6_03	YY1	YY1	0.139398	173
V$FOXJ2_01	FOXJ2	FOXJ2	0.278522	170
V$MYOGENIN_Q6	myogenin	MYOG	0.182893	169
V$FOXM1_01	FOXM1	FOXM1	0.802362	163
V$TEF1_Q6_03	TEF-1	TEAD1	0.0202019	161
V$E2A_Q2	E2A	TCF3	0.414266	160
V$IRF8_Q6	IRF-8	IRF8	0.0603646	152
V$FOXP3_01	FOXP3	FOXP3	0.023785	148
V$TEF1_Q6	TEF-1	TEAD1	0.0202019	148
V$TCF4_01	TCF-4	TCF7L2	0.042094	147
V$WT1_Q6	WT1	WT1	0.208067	129
V$MAZ_Q6_01	MAZ	MAZ	0.369919	124
V$DAX1_01	Dax1	NR0B1	0.584086	117
V$ZABC1_01	ZABC1	ZNF217	0.107751	112
V$CDX1_01	Cdx-1	CDX1	0.100049	112
V$MYOGENIN_Q6_01	myogenin	MYOG	0.182893	111
V$CEBPG_Q6_01	C/EBPgamma	CEBPG	0.465169	99
V$CIZ_01	CIZ	ZNF384	0.147374	87
V$HNF6_Q6	HNF6	ONECUT1	0.03957	86
V$TR4_Q2	TR4	NR2C2	0.111017	86
V$YY1_03	YY1	YY1	0.139398	75
V$HOXB8_01	HOXB8	HOXB8	0.298687	68
V$STAT4_Q5	STAT4	STAT4	0.230881	65
V$CEBPG_Q6	C/EBPgamma	CEBPG	0.465169	56
V$HNF1B_01	HNF-1beta	HNF1B	0.0297195	47
V$OCT2_01	2-Oct	POU2F2	0.045411	37
V$RREB1_01	RREB-1	RREB1	0.135226	34
V$NKX3A_02	Nkx3A	NKX3-1	0.0961027	30
V$RORA1_01	RORalpha1	RORA	0.353568	28
V$DMRT1_01	DMRT1	DMRT1	0.964003	9

----

hsa-mir-511-1

Matrix_id	transcription factor	Gene	PCC	Occurrence
V$SPI1_Q5	SPI1	SPI1	0.465709	4
V$PEA3_Q6	PEA3	ETV4	0.0170145	4

V$YY1_Q6	YY1	YY1	0.177383	4
V$YY1_Q6_02	YY1	YY1	0.177383	4
V$MAFB_01	MAFB	MAFB	0.481216	4
V$ETS2_B	c-Ets-2	ETS2	0.219412	4
V$ELF1_Q6	Elf-1	ELF1	0.117506	4
V$GABPA_Q4	GABP-alpha	GABPA	0.0448007	4
V$IRF8_Q6	IRF-8	IRF8	0.280656	4
V$GKLF_Q4	GKLF	KLF4	0.541425	4
V$CEBPA_Q6	C/EBPalpha	CEBPA	0.0843088	4
V$SPI1_03	SPI1	SPI1	0.465709	4
V$P300_01	p300	EP300	0.0883388	4
V$TCF4_01	TCF-4	TCF7L2	0.294731	3
V$SREBP1_Q6	SREBP-1	SREBF1	0.525024	3
V$IRF7_Q3	IRF-7	IRF7	0.274642	3
V$CIZ_01	CIZ	ZNF384	0.0772149	3
V$CP2_01	CP2	TFCP2	0.211354	3
V$GATA3_03	GATA-3	GATA3	0.327223	3
V$YY1_02	YY1	YY1	0.177383	3
V$IRF7_01	IRF-7	IRF7	0.274642	3
V$FKLF_Q5	FKLF	KLF11	0.314316	2
V$EKLF_Q5	EKLF	KLF1	0.0212612	2
V$EHF_03	EHF	EHF	0.0045321	2
V$IRF1_Q6_01	IRF-1	IRF1	0.210435	2
V$EGR1_02	EGR-1	EGR1	0.142433	2
V$TCF4_Q5	TCF-4	TCF7L2	0.294731	2
V$STAT5A_Q6	STAT5A	STAT5A	0.249788	2
V$IRF1_Q6	IRF-1	IRF1	0.210435	2
V$ESE1_Q3	ESE-1	ELF3	0.214527	2
V$ETS2_Q6	c-Ets-2	ETS2	0.219412	2
V$AHR_Q5	AhR	AHR	0.454223	2
V$MEIS1_01	MEIS1	MEIS1	0.0237784	2
V$SP1_01	Sp1	SP1	0.0457024	2
V$FLI1_02	Fli-1	FLI1	0.103015	1
V$HIF1A_Q6	HIF-1alpha	HIF1A	0.214565	1
V$MAFK_Q3	MafK	MAFK	0.221987	1
V$STAT1_05	STAT1	STAT1	0.218824	1
V$ERM_02	Erm	ETV5	0.515329	1
V$ESE1_02	ESE-1	ELF3	0.214527	1
V$ERG_03	ERG	ERG	0.0423963	1
V$NMYC_01	N-Myc	MYCN	0.116347	1
V$ARNT_02	Arnt	ARNT	0.0009537	1
V$ARNT_01	Arnt	ARNT	0.0009537	1
V$ZABC1_01	ZABC1	ZNF217	0.300837	1
V$MAX_01	Max	MAX	0.213001	1
V$KLF15_Q2	KLF15	KLF15	0.216376	1
V$ERG_01	ERG	ERG	0.0423963	1

V$STAT1_Q6	STAT1	STAT1	0.218824	1
V$IRF2_01	IRF-2	IRF2	0.545082	1
V$IRF1_01	IRF-1	IRF1	0.210435	1
V$YY1_03	YY1	YY1	0.177383	1

----

hsa-mir-511-2

Matrix_id	transcription factor	Gene	PCC	Occurrence
V$GABPA_Q4	GABP-alpha	GABPA	0.0448007	4
V$MAFB_01	MAFB	MAFB	0.481216	4
V$IRF8_Q6	IRF-8	IRF8	0.280656	4
V$YY1_Q6_02	YY1	YY1	0.177383	4
V$YY1_Q6	YY1	YY1	0.177383	4
V$GKLF_Q4	GKLF	KLF4	0.541425	4
V$ELF1_Q6	Elf-1	ELF1	0.117506	4
V$CEBPA_Q6	C/EBPalpha	CEBPA	0.0843088	4
V$PEA3_Q6	PEA3	ETV4	0.0170145	4
V$ETS2_B	c-Ets-2	ETS2	0.219412	4
V$P300_01	p300	EP300	0.0883388	4
V$SPI1_03	SPI1	SPI1	0.465709	4
V$SPI1_Q5	SPI1	SPI1	0.465709	4
V$CP2_01	CP2	TFCP2	0.211354	3
V$TCF4_01	TCF-4	TCF7L2	0.294731	3
V$YY1_02	YY1	YY1	0.177383	3
V$SREBP1_Q6	SREBP-1	SREBF1	0.525024	3
V$IRF7_Q3	IRF-7	IRF7	0.274642	3
V$CIZ_01	CIZ	ZNF384	0.0772149	3
V$IRF7_01	IRF-7	IRF7	0.274642	3
V$FKLF_Q5	FKLF	KLF11	0.314316	2
V$EGR1_02	EGR-1	EGR1	0.142433	2
V$STAT5A_Q6	STAT5A	STAT5A	0.249788	2
V$EKLF_Q5	EKLF	KLF1	0.0212612	2
V$EHF_03	EHF	EHF	0.0045321	2
V$IRF1_Q6_01	IRF-1	IRF1	0.210435	2
V$IRF1_Q6	IRF-1	IRF1	0.210435	2
V$MEIS1_01	MEIS1	MEIS1	0.0237784	2
V$AHR_Q5	AhR	AHR	0.454223	2
V$TCF4_Q5	TCF-4	TCF7L2	0.294731	2
V$ETS2_Q6	c-Ets-2	ETS2	0.219412	2
V$ESE1_Q3	ESE-1	ELF3	0.214527	2
V$SP1_01	Sp1	SP1	0.0457024	2
V$IRF1_01	IRF-1	IRF1	0.210435	1
V$NMYC_01	N-Myc	MYCN	0.116347	1

V$STAT1_05	STAT1	STAT1	0.218824	1
V$ESE1_02	ESE-1	ELF3	0.214527	1
V$ZABC1_01	ZABC1	ZNF217	0.300837	1
V$ERG_03	ERG	ERG	0.0423963	1
V$ERM_02	Erm	ETV5	0.515329	1
V$FLI1_02	Fli-1	FLI1	0.103015	1
V$MAFK_Q3	MafK	MAFK	0.221987	1
V$HIF1A_Q6	HIF-1alpha	HIF1A	0.214565	1
V$IRF2_01	IRF-2	IRF2	0.545082	1
V$MAX_01	Max	MAX	0.213001	1
V$ARNT_01	Arnt	ARNT	0.0009537	1
V$ARNT_02	Arnt	ARNT	0.0009537	1
V$STAT1_Q6	STAT1	STAT1	0.218824	1
V$ERG_01	ERG	ERG	0.0423963	1
V$KLF15_Q2	KLF15	KLF15	0.216376	1
V$YY1_03	YY1	YY1	0.177383	1

---

hsa-mir-512-1

Matrix_id	transcription factor	Gene	PCC	Occurrence
V$PUR1_Q4	PUR1	PURA	0.158745	261
V$PEA3_Q6	PEA3	ETV4	0.0981089	255
V$PARP_Q4	PARP	PARP1	0.0979871	252
V$GKLF_Q4	GKLF	KLF4	0.565909	243
V$MAFB_01	MAFB	MAFB	0.297844	240
V$P300_01	p300	EP300	0.348238	234
V$ETS1_B	c-Ets-1	ETS1	0.0654302	229
V$CETS1_Q6	C-ets-1	ETS1	0.0654302	223
V$SMAD4_Q6_01	Smad4	SMAD4	0.306641	222
V$GABPA_Q4	GABP-alpha	GABPA	0.365113	211
V$GATA1_01	GATA-1	GATA1	0.0152112	210
V$TBP_Q6	TBP	TBP	0.156064	201
V$GR_Q6	GR	NR3C1	0.155247	199
V$TBX5_02	TBX5	TBX5	0.0774492	199
V$YY1_01	YY1	YY1	0.365515	195
V$AML1_Q6	AML1	RUNX1	0.00034091	186
V$DLX5_01	dlx5	DLX5	0.691916	178
V$YY1_Q6_02	YY1	YY1	0.365515	176
V$YY1_Q6	YY1	YY1	0.365515	175
V$AHR_Q5	AhR	AHR	0.964949	168
V$TBX5_01	TBX5	TBX5	0.0774492	158
V$ERBETA_Q5	ER-beta	ESR2	0.0420443	158
V$ING4_01	ING4	ING4	0.113542	157

V$PARP_Q3	PARP	PARP1	0.0979871	157
V$SP1_Q6	Sp1	SP1	0.347444	155
V$HMGIY_01	HMGIY	HMGA1	0.342856	145
V$FKLF_Q5	FKLF	KLF11	0.238573	145
V$SREBP1_Q6	SREBP-1	SREBF1	0.13659	134
V$SP1_01	Sp1	SP1	0.347444	133
V$ELK1_02	Elk-1	ELK1	0.0191925	133
V$CMAF_01	c-Maf	MAF	0.0922767	124
V$ELK1_06	ELK-1	ELK1	0.0191925	122
V$CEBPE_Q6	CEBPE	CEBPE	0.0271494	120
V$GR_01	GR	NR3C1	0.155247	117
V$PITX2_Q2	Pitx2	PITX2	0.429884	109
V$ESE1_Q3	ESE-1	ELF3	0.103228	108
V$AML1_01	AML1a	RUNX1	0.00034091	104
V$PITX2_01	PITX2	PITX2	0.429884	103
V$CP2_01	CP2	TFCP2	0.575131	103
V$ZABC1_01	ZABC1	ZNF217	0.864941	102
V$CEBPD_Q6	C/EBPdelta	CEBPD	0.435924	97
V$SP2_01	SP2	SP2	0.144037	97
V$AML1_Q4	AML1	RUNX1	0.00034091	90
V$HBP1_Q2	hbp1	HBP1	0.667881	89
V$CEBPB_02	C/EBPbeta	CEBPB	0.59578	88
V$ESE1_02	ESE-1	ELF3	0.103228	81
V$CEBPB_Q6	C/EBPbeta	CEBPB	0.59578	76
V$YY1_02	YY1	YY1	0.365515	73
V$CEBPA_01	C/EBPalpha	CEBPA	0.07053	71
V$E2F4_Q6	E2F-4	E2F4	0.0653664	64
V$ERG_03	ERG	ERG	0.33733	58
V$YY1_03	YY1	YY1	0.365515	57
V$CIZ_01	CIZ	ZNF384	0.0810194	55
V$CEBPB_01	C/EBPbeta	CEBPB	0.59578	45
V$ERM_02	Erm	ETV5	0.535537	40
V$ERR3_Q2	ERR3	ESRRG	0.469832	38
V$ERG_01	ERG	ERG	0.33733	38
V$P53_04	p53	TP53	0.605023	7

---

hsa-mir-512-2

Matrix_id	transcription factor	Gene	PCC	Occurrence
V$PUR1_Q4	PUR1	PURA	0.158745	261
V$PARP_Q4	PARP	PARP1	0.0979871	252
V$GKLF_Q4	GKLF	KLF4	0.565909	243
V$MAFB_01	MAFB	MAFB	0.297844	240

V$P300_01	p300	EP300	0.348238	234
V$SMAD4_Q6_01	Smad4	SMAD4	0.306641	222
V$TBX5_02	TBX5	TBX5	0.0774492	199
V$GR_Q6	GR	NR3C1	0.155247	199
V$YY1_01	YY1	YY1	0.365515	195
V$AML1_Q6	AML1	RUNX1	0.00034091	186
V$DLX5_01	dlx5	DLX5	0.691916	178
V$YY1_Q6_02	YY1	YY1	0.365515	176
V$YY1_Q6	YY1	YY1	0.365515	175
V$GATA1_02	GATA-1	GATA1	0.0152112	172
V$GATA1_05	GATA-1	GATA1	0.0152112	165
V$GATA2_02	GATA-2	GATA2	0.693821	165
V$GATA1_06	GATA-1	GATA1	0.0152112	165
V$TBX5_01	TBX5	TBX5	0.0774492	158
V$PARP_Q3	PARP	PARP1	0.0979871	157
V$ING4_01	ING4	ING4	0.113542	157
V$SP1_Q6	Sp1	SP1	0.347444	155
V$GATA1_04	GATA-1	GATA1	0.0152112	147
V$HMGIY_01	HMGIY	HMGA1	0.342856	145
V$GATA3_02	GATA-3	GATA3	0.911284	141
V$ARNT_01	Arnt	ARNT	0.331932	132
V$CEBPE_Q6	CEBPE	CEBPE	0.0271494	120
V$GR_01	GR	NR3C1	0.155247	117
V$TCF4_01	TCF-4	TCF7L2	0.465518	116
V$PITX2_Q2	Pitx2	PITX2	0.429884	109
V$TCF4_Q5	TCF-4	TCF7L2	0.465518	108
V$IRF7_Q3	IRF-7	IRF7	0.129484	106
V$FOXJ2_01	FOXJ2	FOXJ2	0.0449965	104
V$AML1_01	AML1a	RUNX1	0.00034091	104
V$CP2_01	CP2	TFCP2	0.575131	103
V$PITX2_01	PITX2	PITX2	0.429884	103
V$CEBPD_Q6	C/EBPdelta	CEBPD	0.435924	97
V$GATA3_03	GATA-3	GATA3	0.911284	91
V$AML1_Q4	AML1	RUNX1	0.00034091	90
V$STAT3_03	STAT3	STAT3	0.14519	77
V$CEBPB_Q6	C/EBPbeta	CEBPB	0.59578	76
V$FOXO4_02	FOXO4	FOXO4	0.95676	74
V$CEBPA_01	C/EBPalpha	CEBPA	0.07053	71
V$P53_02	p53	TP53	0.605023	66
V$IRF1_Q6_01	IRF-1	IRF1	0.337987	48
V$CDP_04	CDP	CUX1	0.361793	38
V$GRE_C	GR	NR3C1	0.155247	16

----

hsa-mir-513a-1

Matrix_id	transcription factor	Gene	PCC	Occurrence
V$AP2REP_01	AP-2rep	KLF12	0.104783	339
V$GKLF_Q4	GKLF	KLF4	0.135731	338
V$PEA3_Q6	PEA3	ETV4	0.105244	335
V$P300_01	p300	EP300	0.06091	326
V$ZIC3_01	Zic3	ZIC3	0.364024	300
V$SOX9_Q4	SOX9	SOX9	0.650877	295
V$GATA1_01	GATA-1	GATA1	0.166628	290
V$CETS1_Q6	C-ets-1	ETS1	0.169672	289
V$NFAT4_Q3	NF-AT4	NFATC3	0.0812033	283
V$ETS1_B	c-Ets-1	ETS1	0.169672	282
V$YY1_01	YY1	YY1	0.158969	282
V$TBX5_02	TBX5	TBX5	0.0305597	277
V$GABPA_Q4	GABP-alpha	GABPA	0.0261348	276
V$HNF4A_Q6_01	HNF-4alpha	HNF4A	0.00676163	270
V$YY1_Q6	YY1	YY1	0.158969	257
V$AML1_Q6	AML1	RUNX1	0.17817	252
V$YY1_Q6_02	YY1	YY1	0.158969	245
V$SP1_Q6	Sp1	SP1	0.0292724	240
V$NKX32_01	Nkx3-2	NKX3-2	0.32374	227
V$TBX5_01	TBX5	TBX5	0.0305597	225
V$PITX3_Q2	PITX3	PITX3	0.0419136	221
V$MAZ_Q6	MAZ	MAZ	0.380747	221
V$E12_Q6	E12	TCF3	0.433743	218
V$ERBETA_Q5	ER-beta	ESR2	0.256708	218
V$E2A_Q6	E2A	TCF3	0.433743	218
V$E47_02	E47	TCF3	0.433743	217
V$SP1_01	Sp1	SP1	0.0292724	212
V$ING4_01	ING4	ING4	0.100046	208
V$SP1_Q6_01	Sp1	SP1	0.0292724	208
V$ELK1_02	Elk-1	ELK1	0.259013	207
V$SP1_Q4_01	Sp1	SP1	0.0292724	202
V$HNF3B_Q6	HNF-3beta	FOXA2	0.0794719	197
V$ELK1_06	ELK-1	ELK1	0.259013	194
V$SP1_Q2_01	Sp1	SP1	0.0292724	194
V$SP1_02	SP1	SP1	0.0292724	189
V$ERF_02	ERF	ERF	0.0218276	180
V$MYOGENIN_Q6	myogenin	MYOG	0.182505	174
V$CREM_Q6	CREM	CREM	0.528217	174
V$E2A_Q2	E2A	TCF3	0.433743	164
V$FKLF_Q5	FKLF	KLF11	0.314366	155
V$SREBP1_Q6	SREBP-1	SREBF1	0.149918	154
V$FOXP3_01	FOXP3	FOXP3	0.0130914	152
V$GABPBETA_Q3	GABP-beta	GABPB1	0.156045	147

V$AML1_01	AML1a	RUNX1	0.17817	144
V$WT1_Q6	WT1	WT1	0.209998	132
V$AML1_Q4	AML1	RUNX1	0.17817	131
V$MAZ_Q6_01	MAZ	MAZ	0.380747	127
V$E47_01	E47	TCF3	0.433743	123
V$YY1_02	YY1	YY1	0.158969	123
V$DAX1_01	Dax1	NR0B1	0.569677	118
V$MYOGENIN_Q6_01	myogenin	MYOG	0.182505	112
V$ERM_02	Erm	ETV5	0.353231	97
V$ELK1_01	Elk-1	ELK1	0.259013	94
V$SPIB_03	Spi-B	SPIB	0.0443451	85
V$MATH1_Q2	MATH1	ATOH1	0.169532	78
V$EGR2_01	Egr-2	EGR2	0.284316	53
V$GLI3_Q5_01	GLI3	GLI3	0.00951285	31
V$SREBP1_02	SREBP-1	SREBF1	0.149918	28

----

hsa-mir-513a-2

Matrix_id	transcription factor	Gene	PCC	Occurrence
V$AP2REP_01	AP-2rep	KLF12	0.104783	339
V$GKLF_Q4	GKLF	KLF4	0.135731	338
V$PEA3_Q6	PEA3	ETV4	0.105244	335
V$P300_01	p300	EP300	0.06091	326
V$ZIC3_01	Zic3	ZIC3	0.364024	300
V$SOX9_Q4	SOX9	SOX9	0.650877	295
V$GATA1_01	GATA-1	GATA1	0.166628	290
V$CETS1_Q6	C-ets-1	ETS1	0.169672	289
V$NFAT4_Q3	NF-AT4	NFATC3	0.0812033	283
V$ETS1_B	c-Ets-1	ETS1	0.169672	282
V$YY1_01	YY1	YY1	0.158969	282
V$TBX5_02	TBX5	TBX5	0.0305597	277
V$GABPA_Q4	GABP-alpha	GABPA	0.0261348	276
V$HNF4A_Q6_01	HNF-4alpha	HNF4A	0.00676163	270
V$YY1_Q6	YY1	YY1	0.158969	257
V$AML1_Q6	AML1	RUNX1	0.17817	252
V$YY1_Q6_02	YY1	YY1	0.158969	245
V$SP1_Q6	Sp1	SP1	0.0292724	240
V$NKX32_01	Nkx3-2	NKX3-2	0.32374	227
V$TBX5_01	TBX5	TBX5	0.0305597	225
V$PITX3_Q2	PITX3	PITX3	0.0419136	221
V$MAZ_Q6	MAZ	MAZ	0.380747	221
V$E12_Q6	E12	TCF3	0.433743	218
V$ERBETA_Q5	ER-beta	ESR2	0.256708	218

V$E2A_Q6	E2A	TCF3	0.433743	218
V$E47_02	E47	TCF3	0.433743	217
V$SP1_01	Sp1	SP1	0.0292724	212
V$ING4_01	ING4	ING4	0.100046	208
V$SP1_Q6_01	Sp1	SP1	0.0292724	208
V$ELK1_02	Elk-1	ELK1	0.259013	207
V$SP1_Q4_01	Sp1	SP1	0.0292724	202
V$HNF3B_Q6	HNF-3beta	FOXA2	0.0794719	197
V$ELK1_06	ELK-1	ELK1	0.259013	194
V$SP1_Q2_01	Sp1	SP1	0.0292724	194
V$SP1_02	SP1	SP1	0.0292724	189
V$ERF_02	ERF	ERF	0.0218276	180
V$MYOGENIN_Q6	myogenin	MYOG	0.182505	174
V$CREM_Q6	CREM	CREM	0.528217	174
V$E2A_Q2	E2A	TCF3	0.433743	164
V$FKLF_Q5	FKLF	KLF11	0.314366	155
V$SREBP1_Q6	SREBP-1	SREBF1	0.149918	154
V$FOXP3_01	FOXP3	FOXP3	0.0130914	152
V$GABPBETA_Q3	GABP-beta	GABPB1	0.156045	147
V$AML1_01	AML1a	RUNX1	0.17817	144
V$WT1_Q6	WT1	WT1	0.209998	132
V$AML1_Q4	AML1	RUNX1	0.17817	131
V$MAZ_Q6_01	MAZ	MAZ	0.380747	127
V$E47_01	E47	TCF3	0.433743	123
V$YY1_02	YY1	YY1	0.158969	123
V$DAX1_01	Dax1	NR0B1	0.569677	118
V$MYOGENIN_Q6_01	myogenin	MYOG	0.182505	112
V$ERM_02	Erm	ETV5	0.353231	97
V$ELK1_01	Elk-1	ELK1	0.259013	94
V$SPIB_03	Spi-B	SPIB	0.0443451	85
V$MATH1_Q2	MATH1	ATOH1	0.169532	78
V$EGR2_01	Egr-2	EGR2	0.284316	53
V$GLI3_Q5_01	GLI3	GLI3	0.00951285	31
V$SREBP1_02	SREBP-1	SREBF1	0.149918	28

---

hsa-mir-514a-1

Matrix_id	transcription factor	Gene	PCC	Occurrence
V$AP2REP_01	AP-2rep	KLF12	0.10671	338
V$GKLF_Q4	GKLF	KLF4	0.126129	338
V$PARP_Q4	PARP	PARP1	0.198157	334
V$PEA3_Q6	PEA3	ETV4	0.110615	334
V$P300_01	p300	EP300	0.0582464	325

V$TBP_Q6	TBP	TBP	0.813787	298
V$SOX9_Q4	SOX9	SOX9	0.643841	296
V$ETS1_B	c-Ets-1	ETS1	0.155006	283
V$NFAT4_Q3	NF-AT4	NFATC3	0.0735074	283
V$YY1_01	YY1	YY1	0.154308	281
V$HNF4A_Q6_01	HNF-4alpha	HNF4A	0.0307412	271
V$DLX5_01	dlx5	DLX5	0.305071	264
V$SOX5_01	SOX5	SOX5	0.635963	260
V$SOX9_B1	SOX9	SOX9	0.643841	259
V$YY1_Q6	YY1	YY1	0.154308	255
V$YY1_Q6_02	YY1	YY1	0.154308	243
V$GATA1_02	GATA-1	GATA1	0.145362	232
V$GATA1_05	GATA-1	GATA1	0.145362	232
V$GATA1_06	GATA-1	GATA1	0.145362	232
V$SRY_02	SRY	SRY	0.10894	230
V$ERBETA_Q5	ER-beta	ESR2	0.260269	217
V$E2A_Q6	E2A	TCF3	0.425768	216
V$E12_Q6	E12	TCF3	0.425768	216
V$E47_02	E47	TCF3	0.425768	215
V$HMGIY_01	HMGIY	HMGA1	0.62437	208
V$ING4_01	ING4	ING4	0.0904487	207
V$HNF3B_Q6	HNF-3beta	FOXA2	0.0610178	196
V$PARP_Q3	PARP	PARP1	0.198157	194
V$GATA1_04	GATA-1	GATA1	0.145362	188
V$NFAT2_Q5	NF-AT2	NFATC1	0.150335	184
V$YY1_Q6_03	YY1	YY1	0.154308	177
V$FOXJ2_01	FOXJ2	FOXJ2	0.282763	171
V$MYOGENIN_Q6	myogenin	MYOG	0.186003	171
V$FOXM1_01	FOXM1	FOXM1	0.810248	165
V$TEF1_Q6_03	TEF-1	TEAD1	0.0234842	163
V$E2A_Q2	E2A	TCF3	0.425768	162
V$GFI1B_01	Gfi1b	GFI1B	0.00827918	162
V$IRF8_Q6	IRF-8	IRF8	0.0664966	154
V$TCF4_01	TCF-4	TCF7L2	0.0501635	151
V$FOXP3_01	FOXP3	FOXP3	0.0230266	150
V$TEF1_Q6	TEF-1	TEAD1	0.0234842	149
V$WT1_Q6	WT1	WT1	0.20827	132
V$MAZ_Q6_01	MAZ	MAZ	0.380903	127
V$DAX1_01	Dax1	NR0B1	0.582789	118
V$CDX1_01	Cdx-1	CDX1	0.105528	117
V$ZABC1_01	ZABC1	ZNF217	0.117898	114
V$MYOGENIN_Q6_01	myogenin	MYOG	0.186003	112
V$CEBPG_Q6_01	C/EBPgamma	CEBPG	0.482196	99
V$CIZ_01	CIZ	ZNF384	0.145557	89
V$HNF6_Q6	HNF6	ONECUT1	0.0228486	87
V$TR4_Q2	TR4	NR2C2	0.117873	86

V$YY1_03	YY1	YY1	0.154308	77
V$HOXB8_01	HOXB8	HOXB8	0.31066	68
V$STAT4_Q5	STAT4	STAT4	0.236328	67
V$CEBPG_Q6	C/EBPgamma	CEBPG	0.482196	57
V$HNF1B_01	HNF-1beta	HNF1B	0.0258563	48
V$OCT2_01	2-Oct	POU2F2	0.0423763	36
V$RREB1_01	RREB-1	RREB1	0.141112	35
V$NKX3A_02	Nkx3A	NKX3-1	0.10426	30
V$RORA1_01	RORalpha1	RORA	0.345375	27
V$DMRT1_01	DMRT1	DMRT1	0.965998	9

----

hsa-mir-514a-2

Matrix_id	transcription factor	Gene	PCC	Occurrence
V$AP2REP_01	AP-2rep	KLF12	0.10671	338
V$GKLF_Q4	GKLF	KLF4	0.126129	338
V$PARP_Q4	PARP	PARP1	0.198157	334
V$PEA3_Q6	PEA3	ETV4	0.110615	334
V$P300_01	p300	EP300	0.0582464	325
V$TBP_Q6	TBP	TBP	0.813787	298
V$SOX9_Q4	SOX9	SOX9	0.643841	296
V$ETS1_B	c-Ets-1	ETS1	0.155006	283
V$NFAT4_Q3	NF-AT4	NFATC3	0.0735074	283
V$YY1_01	YY1	YY1	0.154308	281
V$HNF4A_Q6_01	HNF-4alpha	HNF4A	0.0307412	271
V$DLX5_01	dlx5	DLX5	0.305071	264
V$SOX5_01	SOX5	SOX5	0.635963	260
V$SOX9_B1	SOX9	SOX9	0.643841	259
V$YY1_Q6	YY1	YY1	0.154308	255
V$YY1_Q6_02	YY1	YY1	0.154308	243
V$GATA1_02	GATA-1	GATA1	0.145362	232
V$GATA1_05	GATA-1	GATA1	0.145362	232
V$GATA1_06	GATA-1	GATA1	0.145362	232
V$SRY_02	SRY	SRY	0.10894	230
V$ERBETA_Q5	ER-beta	ESR2	0.260269	217
V$E2A_Q6	E2A	TCF3	0.425768	216
V$E12_Q6	E12	TCF3	0.425768	216
V$E47_02	E47	TCF3	0.425768	215
V$HMGIY_01	HMGIY	HMGA1	0.62437	208
V$ING4_01	ING4	ING4	0.0904487	207
V$HNF3B_Q6	HNF-3beta	FOXA2	0.0610178	196
V$PARP_Q3	PARP	PARP1	0.198157	194
V$GATA1_04	GATA-1	GATA1	0.145362	188

V$NFAT2_Q5	NF-AT2	NFATC1	0.150335	184
V$YY1_Q6_03	YY1	YY1	0.154308	177
V$FOXJ2_01	FOXJ2	FOXJ2	0.282763	171
V$MYOGENIN_Q6	myogenin	MYOG	0.186003	171
V$FOXM1_01	FOXM1	FOXM1	0.810248	165
V$TEF1_Q6_03	TEF-1	TEAD1	0.0234842	163
V$E2A_Q2	E2A	TCF3	0.425768	162
V$GFI1B_01	Gfi1b	GFI1B	0.00827918	162
V$IRF8_Q6	IRF-8	IRF8	0.0664966	154
V$TCF4_01	TCF-4	TCF7L2	0.0501635	151
V$FOXP3_01	FOXP3	FOXP3	0.0230266	150
V$TEF1_Q6	TEF-1	TEAD1	0.0234842	149
V$WT1_Q6	WT1	WT1	0.20827	132
V$MAZ_Q6_01	MAZ	MAZ	0.380903	127
V$DAX1_01	Dax1	NR0B1	0.582789	118
V$CDX1_01	Cdx-1	CDX1	0.105528	117
V$ZABC1_01	ZABC1	ZNF217	0.117898	114
V$MYOGENIN_Q6_01	myogenin	MYOG	0.186003	112
V$CEBPG_Q6_01	C/EBPgamma	CEBPG	0.482196	99
V$CIZ_01	CIZ	ZNF384	0.145557	89
V$HNF6_Q6	HNF6	ONECUT1	0.0228486	87
V$TR4_Q2	TR4	NR2C2	0.117873	86
V$YY1_03	YY1	YY1	0.154308	77
V$HOXB8_01	HOXB8	HOXB8	0.31066	68
V$STAT4_Q5	STAT4	STAT4	0.236328	67
V$CEBPG_Q6	C/EBPgamma	CEBPG	0.482196	57
V$HNF1B_01	HNF-1beta	HNF1B	0.0258563	48
V$OCT2_01	2-Oct	POU2F2	0.0423763	36
V$RREB1_01	RREB-1	RREB1	0.141112	35
V$NKX3A_02	Nkx3A	NKX3-1	0.10426	30
V$RORA1_01	RORalpha1	RORA	0.345375	27
V$DMRT1_01	DMRT1	DMRT1	0.965998	9

----

hsa-mir-514a-3

Matrix_id	transcription factor	Gene	PCC	Occurrence
V$AP2REP_01	AP-2rep	KLF12	0.10671	338
V$GKLF_Q4	GKLF	KLF4	0.126129	338
V$PARP_Q4	PARP	PARP1	0.198157	334
V$PEA3_Q6	PEA3	ETV4	0.110615	334
V$P300_01	p300	EP300	0.0582464	325
V$TBP_Q6	TBP	TBP	0.813787	298
V$SOX9_Q4	SOX9	SOX9	0.643841	296

V$ETS1_B	c-Ets-1	ETS1	0.155006	283
V$NFAT4_Q3	NF-AT4	NFATC3	0.0735074	283
V$YY1_01	YY1	YY1	0.154308	281
V$HNF4A_Q6_01	HNF-4alpha	HNF4A	0.0307412	271
V$DLX5_01	dlx5	DLX5	0.305071	264
V$SOX5_01	SOX5	SOX5	0.635963	260
V$SOX9_B1	SOX9	SOX9	0.643841	259
V$YY1_Q6	YY1	YY1	0.154308	255
V$YY1_Q6_02	YY1	YY1	0.154308	243
V$GATA1_02	GATA-1	GATA1	0.145362	232
V$GATA1_05	GATA-1	GATA1	0.145362	232
V$GATA1_06	GATA-1	GATA1	0.145362	232
V$SRY_02	SRY	SRY	0.10894	230
V$ERBETA_Q5	ER-beta	ESR2	0.260269	217
V$E2A_Q6	E2A	TCF3	0.425768	216
V$E12_Q6	E12	TCF3	0.425768	216
V$E47_02	E47	TCF3	0.425768	215
V$HMGIY_01	HMGIY	HMGA1	0.62437	208
V$ING4_01	ING4	ING4	0.0904487	207
V$HNF3B_Q6	HNF-3beta	FOXA2	0.0610178	196
V$PARP_Q3	PARP	PARP1	0.198157	194
V$GATA1_04	GATA-1	GATA1	0.145362	188
V$NFAT2_Q5	NF-AT2	NFATC1	0.150335	184
V$YY1_Q6_03	YY1	YY1	0.154308	177
V$FOXJ2_01	FOXJ2	FOXJ2	0.282763	171
V$MYOGENIN_Q6	myogenin	MYOG	0.186003	171
V$FOXM1_01	FOXM1	FOXM1	0.810248	165
V$TEF1_Q6_03	TEF-1	TEAD1	0.0234842	163
V$E2A_Q2	E2A	TCF3	0.425768	162
V$GFI1B_01	Gfi1b	GFI1B	0.00827918	162
V$IRF8_Q6	IRF-8	IRF8	0.0664966	154
V$TCF4_01	TCF-4	TCF7L2	0.0501635	151
V$FOXP3_01	FOXP3	FOXP3	0.0230266	150
V$TEF1_Q6	TEF-1	TEAD1	0.0234842	149
V$WT1_Q6	WT1	WT1	0.20827	132
V$MAZ_Q6_01	MAZ	MAZ	0.380903	127
V$DAX1_01	Dax1	NR0B1	0.582789	118
V$CDX1_01	Cdx-1	CDX1	0.105528	117
V$ZABC1_01	ZABC1	ZNF217	0.117898	114
V$MYOGENIN_Q6_01	myogenin	MYOG	0.186003	112
V$CEBPG_Q6_01	C/EBPgamma	CEBPG	0.482196	99
V$CIZ_01	CIZ	ZNF384	0.145557	89
V$HNF6_Q6	HNF6	ONECUT1	0.0228486	87
V$TR4_Q2	TR4	NR2C2	0.117873	86
V$YY1_03	YY1	YY1	0.154308	77
V$HOXB8_01	HOXB8	HOXB8	0.31066	68

V$STAT4_Q5	STAT4	STAT4	0.236328	67
V$CEBPG_Q6	C/EBPgamma	CEBPG	0.482196	57
V$HNF1B_01	HNF-1beta	HNF1B	0.0258563	48
V$OCT2_01	2-Oct	POU2F2	0.0423763	36
V$RREB1_01	RREB-1	RREB1	0.141112	35
V$NKX3A_02	Nkx3A	NKX3-1	0.10426	30
V$RORA1_01	RORalpha1	RORA	0.345375	27
V$DMRT1_01	DMRT1	DMRT1	0.965998	9

----

hsa-mir-515-1

Matrix_id	transcription factor	Gene	PCC	Occurrence
V$PUR1_Q4	PUR1	PURA	0.158324	261
V$PARP_Q4	PARP	PARP1	0.0983878	252
V$GKLF_Q4	GKLF	KLF4	0.566234	243
V$MAFB_01	MAFB	MAFB	0.297314	240
V$P300_01	p300	EP300	0.348388	234
V$SMAD4_Q6_01	Smad4	SMAD4	0.306618	222
V$TBX5_02	TBX5	TBX5	0.0775044	199
V$GR_Q6	GR	NR3C1	0.154802	199
V$YY1_01	YY1	YY1	0.365858	195
V$AML1_Q6	AML1	RUNX1	0.00068527	186
V$DLX5_01	dlx5	DLX5	0.692598	178
V$YY1_Q6_02	YY1	YY1	0.365858	176
V$YY1_Q6	YY1	YY1	0.365858	175
V$GATA1_02	GATA-1	GATA1	0.0155287	172
V$GATA1_05	GATA-1	GATA1	0.0155287	165
V$GATA2_02	GATA-2	GATA2	0.693704	165
V$GATA1_06	GATA-1	GATA1	0.0155287	165
V$TBX5_01	TBX5	TBX5	0.0775044	158
V$PARP_Q3	PARP	PARP1	0.0983878	157
V$ING4_01	ING4	ING4	0.113723	157
V$SP1_Q6	Sp1	SP1	0.347534	155
V$GATA1_04	GATA-1	GATA1	0.0155287	147
V$HMGIY_01	HMGIY	HMGA1	0.344195	145
V$GATA3_02	GATA-3	GATA3	0.911195	141
V$ARNT_01	Arnt	ARNT	0.331656	132
V$CEBPE_Q6	CEBPE	CEBPE	0.0269462	120
V$GR_01	GR	NR3C1	0.154802	117
V$TCF4_01	TCF-4	TCF7L2	0.46567	116
V$PITX2_Q2	Pitx2	PITX2	0.429703	109
V$TCF4_Q5	TCF-4	TCF7L2	0.46567	108
V$IRF7_Q3	IRF-7	IRF7	0.128957	106

V$FOXJ2_01	FOXJ2	FOXJ2	0.0455424	104
V$AML1_01	AML1a	RUNX1	0.00068527	104
V$CP2_01	CP2	TFCP2	0.574999	103
V$PITX2_01	PITX2	PITX2	0.429703	103
V$CEBPD_Q6	C/EBPdelta	CEBPD	0.43544	97
V$GATA3_03	GATA-3	GATA3	0.911195	91
V$AML1_Q4	AML1	RUNX1	0.00068527	90
V$STAT3_03	STAT3	STAT3	0.144926	77
V$CEBPB_Q6	C/EBPbeta	CEBPB	0.595412	76
V$FOXO4_02	FOXO4	FOXO4	0.956862	74
V$CEBPA_01	C/EBPalpha	CEBPA	0.0703573	71
V$P53_02	p53	TP53	0.605339	66
V$IRF1_Q6_01	IRF-1	IRF1	0.337992	48
V$CDP_04	CDP	CUX1	0.361474	38
V$GRE_C	GR	NR3C1	0.154802	16

---

hsa-mir-515-2

Matrix_id	transcription factor	Gene	PCC	Occurrence
V$PUR1_Q4	PUR1	PURA	0.158324	261
V$PARP_Q4	PARP	PARP1	0.0983878	252
V$GKLF_Q4	GKLF	KLF4	0.566234	243
V$MAFB_01	MAFB	MAFB	0.297314	240
V$P300_01	p300	EP300	0.348388	234
V$SMAD4_Q6_01	Smad4	SMAD4	0.306618	222
V$TBX5_02	TBX5	TBX5	0.0775044	199
V$GR_Q6	GR	NR3C1	0.154802	199
V$YY1_01	YY1	YY1	0.365858	195
V$AML1_Q6	AML1	RUNX1	0.00068527	186
V$DLX5_01	dlx5	DLX5	0.692598	178
V$YY1_Q6_02	YY1	YY1	0.365858	176
V$YY1_Q6	YY1	YY1	0.365858	175
V$GATA1_02	GATA-1	GATA1	0.0155287	172
V$GATA1_05	GATA-1	GATA1	0.0155287	165
V$GATA2_02	GATA-2	GATA2	0.693704	165
V$GATA1_06	GATA-1	GATA1	0.0155287	165
V$TBX5_01	TBX5	TBX5	0.0775044	158
V$PARP_Q3	PARP	PARP1	0.0983878	157
V$ING4_01	ING4	ING4	0.113723	157
V$SP1_Q6	Sp1	SP1	0.347534	155
V$GATA1_04	GATA-1	GATA1	0.0155287	147
V$HMGIY_01	HMGIY	HMGA1	0.344195	145
V$GATA3_02	GATA-3	GATA3	0.911195	141

V$ARNT_01	Arnt	ARNT	0.331656	132
V$CEBPE_Q6	CEBPE	CEBPE	0.0269462	120
V$GR_01	GR	NR3C1	0.154802	117
V$TCF4_01	TCF-4	TCF7L2	0.46567	116
V$PITX2_Q2	Pitx2	PITX2	0.429703	109
V$TCF4_Q5	TCF-4	TCF7L2	0.46567	108
V$IRF7_Q3	IRF-7	IRF7	0.128957	106
V$FOXJ2_01	FOXJ2	FOXJ2	0.0455424	104
V$AML1_01	AML1a	RUNX1	0.00068527	104
V$CP2_01	CP2	TFCP2	0.574999	103
V$PITX2_01	PITX2	PITX2	0.429703	103
V$CEBPD_Q6	C/EBPdelta	CEBPD	0.43544	97
V$GATA3_03	GATA-3	GATA3	0.911195	91
V$AML1_Q4	AML1	RUNX1	0.00068527	90
V$STAT3_03	STAT3	STAT3	0.144926	77
V$CEBPB_Q6	C/EBPbeta	CEBPB	0.595412	76
V$FOXO4_02	FOXO4	FOXO4	0.956862	74
V$CEBPA_01	C/EBPalpha	CEBPA	0.0703573	71
V$P53_02	p53	TP53	0.605339	66
V$IRF1_Q6_01	IRF-1	IRF1	0.337992	48
V$CDP_04	CDP	CUX1	0.361474	38
V$GRE_C	GR	NR3C1	0.154802	16

---

hsa-mir-517a

Matrix_id	transcription factor	Gene	PCC	Occurrence
V$PUR1_Q4	PUR1	PURA	0.159597	262
V$PARP_Q4	PARP	PARP1	0.0985748	253
V$GKLF_Q4	GKLF	KLF4	0.566562	244
V$MAFB_01	MAFB	MAFB	0.299378	241
V$P300_01	p300	EP300	0.348845	235
V$SMAD4_Q6_01	Smad4	SMAD4	0.307764	223
V$TBX5_02	TBX5	TBX5	0.0781343	200
V$GR_Q6	GR	NR3C1	0.156632	200
V$YY1_01	YY1	YY1	0.366383	196
V$AML1_Q6	AML1	RUNX1	0.00089125	187
V$DLX5_01	dlx5	DLX5	0.691452	178
V$YY1_Q6_02	YY1	YY1	0.366383	177
V$YY1_Q6	YY1	YY1	0.366383	176
V$GATA1_02	GATA-1	GATA1	0.0160112	172
V$GATA1_05	GATA-1	GATA1	0.0160112	165
V$GATA2_02	GATA-2	GATA2	0.69469	165
V$GATA1_06	GATA-1	GATA1	0.0160112	165

V$TBX5_01	TBX5	TBX5	0.0781343	159
V$PARP_Q3	PARP	PARP1	0.0985748	158
V$ING4_01	ING4	ING4	0.115007	158
V$SP1_Q6	Sp1	SP1	0.347907	156
V$GATA1_04	GATA-1	GATA1	0.0160112	147
V$HMGIY_01	HMGIY	HMGA1	0.341793	145
V$GATA3_02	GATA-3	GATA3	0.911784	141
V$ARNT_01	Arnt	ARNT	0.332974	132
V$CEBPE_Q6	CEBPE	CEBPE	0.027951	121
V$GR_01	GR	NR3C1	0.156632	117
V$TCF4_01	TCF-4	TCF7L2	0.466466	116
V$PITX2_Q2	Pitx2	PITX2	0.430065	110
V$TCF4_Q5	TCF-4	TCF7L2	0.466466	109
V$IRF7_Q3	IRF-7	IRF7	0.130482	106
V$AML1_01	AML1a	RUNX1	0.00089125	105
V$FOXJ2_01	FOXJ2	FOXJ2	0.0453317	104
V$PITX2_01	PITX2	PITX2	0.430065	104
V$CP2_01	CP2	TFCP2	0.575514	103
V$CEBPD_Q6	C/EBPdelta	CEBPD	0.437343	98
V$GATA3_03	GATA-3	GATA3	0.911784	91
V$AML1_Q4	AML1	RUNX1	0.00089125	91
V$STAT3_03	STAT3	STAT3	0.146038	77
V$CEBPB_Q6	C/EBPbeta	CEBPB	0.596652	77
V$FOXO4_02	FOXO4	FOXO4	0.956358	74
V$CEBPA_01	C/EBPalpha	CEBPA	0.0710433	72
V$P53_02	p53	TP53	0.605304	66
V$IRF1_Q6_01	IRF-1	IRF1	0.338196	48
V$CDP_04	CDP	CUX1	0.363033	38
V$GRE_C	GR	NR3C1	0.156632	16

----

hsa-mir-518b

Matrix_id	transcription factor	Gene	PCC	Occurrence
V$PUR1_Q4	PUR1	PURA	0.158994	261
V$PARP_Q4	PARP	PARP1	0.0960098	252
V$GKLF_Q4	GKLF	KLF4	0.567167	244
V$MAFB_01	MAFB	MAFB	0.299154	240
V$P300_01	p300	EP300	0.348224	234
V$SMAD4_Q6_01	Smad4	SMAD4	0.306605	222
V$GR_Q6	GR	NR3C1	0.15543	199
V$TBX5_02	TBX5	TBX5	0.0759386	199
V$YY1_01	YY1	YY1	0.366001	195
V$DLX5_01	dlx5	DLX5	0.690771	177

V$YY1_Q6_02	YY1	YY1	0.366001	176
V$YY1_Q6	YY1	YY1	0.366001	176
V$GATA1_02	GATA-1	GATA1	0.0137705	171
V$GATA1_06	GATA-1	GATA1	0.0137705	164
V$GATA1_05	GATA-1	GATA1	0.0137705	164
V$GATA2_02	GATA-2	GATA2	0.692805	164
V$ING4_01	ING4	ING4	0.109653	158
V$TBX5_01	TBX5	TBX5	0.0759386	158
V$PARP_Q3	PARP	PARP1	0.0960098	157
V$SP1_Q6	Sp1	SP1	0.346647	155
V$GATA1_04	GATA-1	GATA1	0.0137705	146
V$HMGIY_01	HMGIY	HMGA1	0.342415	144
V$GATA3_02	GATA-3	GATA3	0.910615	140
V$ARNT_01	Arnt	ARNT	0.33102	132
V$CEBPE_Q6	CEBPE	CEBPE	0.0261909	120
V$GR_01	GR	NR3C1	0.15543	117
V$TCF4_01	TCF-4	TCF7L2	0.467338	116
V$PITX2_Q2	Pitx2	PITX2	0.429709	110
V$TCF4_Q5	TCF-4	TCF7L2	0.467338	109
V$IRF7_Q3	IRF-7	IRF7	0.128621	106
V$PITX2_01	PITX2	PITX2	0.429709	104
V$FOXJ2_01	FOXJ2	FOXJ2	0.0442497	104
V$CP2_01	CP2	TFCP2	0.575438	102
V$CEBPD_Q6	C/EBPdelta	CEBPD	0.435813	98
V$GATA3_03	GATA-3	GATA3	0.910615	90
V$STAT3_03	STAT3	STAT3	0.144555	76
V$CEBPB_Q6	C/EBPbeta	CEBPB	0.595079	76
V$FOXO4_02	FOXO4	FOXO4	0.957536	73
V$CEBPA_01	C/EBPalpha	CEBPA	0.0697542	72
V$P53_02	p53	TP53	0.602834	66
V$IRF1_Q6_01	IRF-1	IRF1	0.338483	48
V$CDP_04	CDP	CUX1	0.361576	38
V$GRE_C	GR	NR3C1	0.15543	16

----

hsa-mir-518c

Matrix_id	transcription factor	Gene	PCC	Occurrence
V$PUR1_Q4	PUR1	PURA	0.158856	260
V$PARP_Q4	PARP	PARP1	0.0970415	251
V$GKLF_Q4	GKLF	KLF4	0.565031	243
V$MAFB_01	MAFB	MAFB	0.297709	239
V$P300_01	p300	EP300	0.347649	233
V$SMAD4_Q6_01	Smad4	SMAD4	0.305999	221

V$GR_Q6	GR	NR3C1	0.155078	198
V$TBX5_02	TBX5	TBX5	0.0769572	198
V$YY1_01	YY1	YY1	0.364485	194
V$DLX5_01	dlx5	DLX5	0.691174	177
V$YY1_Q6_02	YY1	YY1	0.364485	175
V$YY1_Q6	YY1	YY1	0.364485	175
V$GATA1_02	GATA-1	GATA1	0.0142632	171
V$GATA1_06	GATA-1	GATA1	0.0142632	164
V$GATA1_05	GATA-1	GATA1	0.0142632	164
V$GATA2_02	GATA-2	GATA2	0.693465	164
V$ING4_01	ING4	ING4	0.112397	157
V$TBX5_01	TBX5	TBX5	0.0769572	157
V$PARP_Q3	PARP	PARP1	0.0970415	156
V$SP1_Q6	Sp1	SP1	0.347029	154
V$GATA1_04	GATA-1	GATA1	0.0142632	146
V$HMGIY_01	HMGIY	HMGA1	0.341503	144
V$GATA3_02	GATA-3	GATA3	0.911105	140
V$ARNT_01	Arnt	ARNT	0.331714	132
V$CEBPE_Q6	CEBPE	CEBPE	0.0269708	119
V$GR_01	GR	NR3C1	0.155078	117
V$TCF4_01	TCF-4	TCF7L2	0.46472	116
V$PITX2_Q2	Pitx2	PITX2	0.430039	109
V$TCF4_Q5	TCF-4	TCF7L2	0.46472	108
V$IRF7_Q3	IRF-7	IRF7	0.129665	106
V$FOXJ2_01	FOXJ2	FOXJ2	0.0439877	104
V$PITX2_01	PITX2	PITX2	0.430039	103
V$CP2_01	CP2	TFCP2	0.575089	102
V$CEBPD_Q6	C/EBPdelta	CEBPD	0.435786	97
V$GATA3_03	GATA-3	GATA3	0.911105	90
V$STAT3_03	STAT3	STAT3	0.145073	76
V$CEBPB_Q6	C/EBPbeta	CEBPB	0.595797	75
V$FOXO4_02	FOXO4	FOXO4	0.956836	73
V$CEBPA_01	C/EBPalpha	CEBPA	0.0704781	71
V$P53_02	p53	TP53	0.604381	66
V$IRF1_Q6_01	IRF-1	IRF1	0.337851	48
V$CDP_04	CDP	CUX1	0.36152	38
V$GRE_C	GR	NR3C1	0.155078	16

----

hsa-mir-518f

Matrix_id	transcription factor	Gene	PCC	Occurrence
V$PUR1_Q4	PUR1	PURA	0.159235	261
V$PARP_Q4	PARP	PARP1	0.0978238	252

V$GKLF_Q4	GKLF	KLF4	0.565814	244
V$MAFB_01	MAFB	MAFB	0.298562	240
V$P300_01	p300	EP300	0.34826	234
V$SMAD4_Q6_01	Smad4	SMAD4	0.3069	222
V$TBX5_02	TBX5	TBX5	0.0775578	199
V$GR_Q6	GR	NR3C1	0.155871	199
V$YY1_01	YY1	YY1	0.365454	195
V$AML1_Q6	AML1	RUNX1	0.00021059	186
V$DLX5_01	dlx5	DLX5	0.691319	177
V$YY1_Q6	YY1	YY1	0.365454	176
V$YY1_Q6_02	YY1	YY1	0.365454	176
V$GATA1_02	GATA-1	GATA1	0.0151546	171
V$GATA1_06	GATA-1	GATA1	0.0151546	164
V$GATA2_02	GATA-2	GATA2	0.694093	164
V$GATA1_05	GATA-1	GATA1	0.0151546	164
V$TBX5_01	TBX5	TBX5	0.0775578	158
V$ING4_01	ING4	ING4	0.113729	158
V$PARP_Q3	PARP	PARP1	0.0978238	157
V$SP1_Q6	Sp1	SP1	0.347479	155
V$GATA1_04	GATA-1	GATA1	0.0151546	146
V$HMGIY_01	HMGIY	HMGA1	0.341653	144
V$GATA3_02	GATA-3	GATA3	0.911455	140
V$ARNT_01	Arnt	ARNT	0.332358	132
V$CEBPE_Q6	CEBPE	CEBPE	0.0274708	120
V$GR_01	GR	NR3C1	0.155871	117
V$TCF4_01	TCF-4	TCF7L2	0.465613	116
V$PITX2_Q2	Pitx2	PITX2	0.430054	110
V$TCF4_Q5	TCF-4	TCF7L2	0.465613	109
V$IRF7_Q3	IRF-7	IRF7	0.130082	106
V$FOXJ2_01	FOXJ2	FOXJ2	0.0446732	104
V$PITX2_01	PITX2	PITX2	0.430054	104
V$AML1_01	AML1a	RUNX1	0.00021059	104
V$CP2_01	CP2	TFCP2	0.575309	102
V$CEBPD_Q6	C/EBPdelta	CEBPD	0.436582	98
V$GATA3_03	GATA-3	GATA3	0.911455	90
V$AML1_Q4	AML1	RUNX1	0.00021059	90
V$STAT3_03	STAT3	STAT3	0.145566	76
V$CEBPB_Q6	C/EBPbeta	CEBPB	0.596236	76
V$FOXO4_02	FOXO4	FOXO4	0.956597	73
V$CEBPA_01	C/EBPalpha	CEBPA	0.0707667	72
V$P53_02	p53	TP53	0.604854	66
V$IRF1_Q6_01	IRF-1	IRF1	0.338028	48
V$CDP_04	CDP	CUX1	0.362293	38
V$GRE_C	GR	NR3C1	0.155871	16

----

hsa-mir-519b

Matrix_id	transcription factor	Gene	PCC	Occurrence
V$PUR1_Q4	PUR1	PURA	0.16005	263
V$PARP_Q4	PARP	PARP1	0.0995149	254
V$GKLF_Q4	GKLF	KLF4	0.567492	246
V$MAFB_01	MAFB	MAFB	0.300398	241
V$P300_01	p300	EP300	0.349573	236
V$SMAD4_Q6_01	Smad4	SMAD4	0.308843	223
V$GR_Q6	GR	NR3C1	0.157584	201
V$TBX5_02	TBX5	TBX5	0.0788559	200
V$YY1_01	YY1	YY1	0.367543	197
V$AML1_Q6	AML1	RUNX1	0.00174447	188
V$DLX5_01	dlx5	DLX5	0.691609	178
V$YY1_Q6	YY1	YY1	0.367543	176
V$YY1_Q6_02	YY1	YY1	0.367543	176
V$GATA1_02	GATA-1	GATA1	0.0170848	173
V$GATA1_06	GATA-1	GATA1	0.0170848	165
V$GATA2_02	GATA-2	GATA2	0.695429	165
V$GATA1_05	GATA-1	GATA1	0.0170848	165
V$ING4_01	ING4	ING4	0.116608	160
V$PARP_Q3	PARP	PARP1	0.0995149	159
V$TBX5_01	TBX5	TBX5	0.0788559	159
V$SP1_Q6	Sp1	SP1	0.34844	156
V$GATA1_04	GATA-1	GATA1	0.0170848	148
V$HMGIY_01	HMGIY	HMGA1	0.341965	145
V$GATA3_02	GATA-3	GATA3	0.912183	141
V$ARNT_01	Arnt	ARNT	0.333742	134
V$CEBPE_Q6	CEBPE	CEBPE	0.0285527	121
V$GR_01	GR	NR3C1	0.157584	119
V$TCF4_01	TCF-4	TCF7L2	0.46753	118
V$TCF4_Q5	TCF-4	TCF7L2	0.46753	111
V$PITX2_Q2	Pitx2	PITX2	0.430072	111
V$IRF7_Q3	IRF-7	IRF7	0.130981	107
V$FOXJ2_01	FOXJ2	FOXJ2	0.0461565	106
V$PITX2_01	PITX2	PITX2	0.430072	105
V$AML1_01	AML1a	RUNX1	0.00174447	105
V$CP2_01	CP2	TFCP2	0.575764	103
V$CEBPD_Q6	C/EBPdelta	CEBPD	0.438292	98
V$GATA3_03	GATA-3	GATA3	0.912183	92
V$AML1_Q4	AML1	RUNX1	0.00174447	90
V$STAT3_03	STAT3	STAT3	0.146628	76
V$CEBPB_Q6	C/EBPbeta	CEBPB	0.597165	76
V$FOXO4_02	FOXO4	FOXO4	0.956047	75

V$CEBPA_01	C/EBPalpha	CEBPA	0.0713893	72
V$P53_02	p53	TP53	0.605859	66
V$IRF1_Q6_01	IRF-1	IRF1	0.338402	49
V$CDP_04	CDP	CUX1	0.363955	38
V$GRE_C	GR	NR3C1	0.157584	16

----

hsa-mir-519c

Matrix_id	transcription factor	Gene	PCC	Occurrence
V$PUR1_Q4	PUR1	PURA	0.157957	261
V$PARP_Q4	PARP	PARP1	0.0982677	252
V$GKLF_Q4	GKLF	KLF4	0.566075	243
V$MAFB_01	MAFB	MAFB	0.296697	240
V$P300_01	p300	EP300	0.348212	234
V$SMAD4_Q6_01	Smad4	SMAD4	0.306236	222
V$TBX5_02	TBX5	TBX5	0.0772853	199
V$GR_Q6	GR	NR3C1	0.154252	199
V$YY1_01	YY1	YY1	0.365631	195
V$AML1_Q6	AML1	RUNX1	0.00056746	186
V$DLX5_01	dlx5	DLX5	0.692881	178
V$YY1_Q6_02	YY1	YY1	0.365631	176
V$YY1_Q6	YY1	YY1	0.365631	175
V$GATA1_02	GATA-1	GATA1	0.0153221	172
V$GATA1_05	GATA-1	GATA1	0.0153221	165
V$GATA2_02	GATA-2	GATA2	0.693386	165
V$GATA1_06	GATA-1	GATA1	0.0153221	165
V$TBX5_01	TBX5	TBX5	0.0772853	158
V$PARP_Q3	PARP	PARP1	0.0982677	157
V$ING4_01	ING4	ING4	0.113268	157
V$SP1_Q6	Sp1	SP1	0.347394	155
V$GATA1_04	GATA-1	GATA1	0.0153221	147
V$HMGIY_01	HMGIY	HMGA1	0.344807	145
V$GATA3_02	GATA-3	GATA3	0.911005	141
V$ARNT_01	Arnt	ARNT	0.331252	132
V$CEBPE_Q6	CEBPE	CEBPE	0.0266386	120
V$GR_01	GR	NR3C1	0.154252	117
V$TCF4_01	TCF-4	TCF7L2	0.465379	116
V$PITX2_Q2	Pitx2	PITX2	0.429606	109
V$TCF4_Q5	TCF-4	TCF7L2	0.465379	108
V$IRF7_Q3	IRF-7	IRF7	0.128521	106
V$FOXJ2_01	FOXJ2	FOXJ2	0.0455351	104
V$AML1_01	AML1a	RUNX1	0.00056746	104
V$CP2_01	CP2	TFCP2	0.574842	103

V$PITX2_01	PITX2	PITX2	0.429606	103
V$CEBPD_Q6	C/EBPdelta	CEBPD	0.434869	97
V$GATA3_03	GATA-3	GATA3	0.911005	91
V$AML1_Q4	AML1	RUNX1	0.00056746	90
V$STAT3_03	STAT3	STAT3	0.144591	77
V$CEBPB_Q6	C/EBPbeta	CEBPB	0.595046	76
V$FOXO4_02	FOXO4	FOXO4	0.957011	74
V$CEBPA_01	C/EBPalpha	CEBPA	0.0701517	71
V$P53_02	p53	TP53	0.605303	66
V$IRF1_Q6_01	IRF-1	IRF1	0.337922	48
V$CDP_04	CDP	CUX1	0.360996	38
V$GRE_C	GR	NR3C1	0.154252	16

hsa-mir-519d

Matrix_id	transcription factor	Gene	PCC	Occurrence
V$PUR1_Q4	PUR1	PURA	0.158599	261
V$PARP_Q4	PARP	PARP1	0.0980386	252
V$GKLF_Q4	GKLF	KLF4	0.56594	243
V$MAFB_01	MAFB	MAFB	0.297633	240
V$P300_01	p300	EP300	0.348233	234
V$SMAD4_Q6_01	Smad4	SMAD4	0.306566	222
V$TBX5_02	TBX5	TBX5	0.0774188	199
V$GR_Q6	GR	NR3C1	0.155064	199
V$YY1_01	YY1	YY1	0.365537	195
V$AML1_Q6	AML1	RUNX1	0.00038244	186
V$DLX5_01	dlx5	DLX5	0.692094	178
V$YY1_Q6_02	YY1	YY1	0.365537	176
V$YY1_Q6	YY1	YY1	0.365537	175
V$GATA1_02	GATA-1	GATA1	0.0152314	172
V$GATA1_05	GATA-1	GATA1	0.0152314	165
V$GATA2_02	GATA-2	GATA2	0.693741	165
V$GATA1_06	GATA-1	GATA1	0.0152314	165
V$TBX5_01	TBX5	TBX5	0.0774188	158
V$PARP_Q3	PARP	PARP1	0.0980386	157
V$ING4_01	ING4	ING4	0.113491	157
V$SP1_Q6	Sp1	SP1	0.347435	155
V$GATA1_04	GATA-1	GATA1	0.0152314	147
V$HMGIY_01	HMGIY	HMGA1	0.343216	145
V$GATA3_02	GATA-3	GATA3	0.911233	141
V$ARNT_01	Arnt	ARNT	0.331807	132
V$CEBPE_Q6	CEBPE	CEBPE	0.027055	120
V$GR_01	GR	NR3C1	0.155064	117

V$TCF4_01	TCF-4	TCF7L2	0.465492	116
V$PITX2_Q2	Pitx2	PITX2	0.429833	109
V$TCF4_Q5	TCF-4	TCF7L2	0.465492	108
V$IRF7_Q3	IRF-7	IRF7	0.129306	106
V$FOXJ2_01	FOXJ2	FOXJ2	0.0450956	104
V$AML1_01	AML1a	RUNX1	0.00038244	104
V$CP2_01	CP2	TFCP2	0.575078	103
V$PITX2_01	PITX2	PITX2	0.429833	103
V$CEBPD_Q6	C/EBPdelta	CEBPD	0.435729	97
V$GATA3_03	GATA-3	GATA3	0.911233	91
V$AML1_Q4	AML1	RUNX1	0.00038244	90
V$STAT3_03	STAT3	STAT3	0.14508	77
V$CEBPB_Q6	C/EBPbeta	CEBPB	0.595645	76
V$FOXO4_02	FOXO4	FOXO4	0.956807	74
V$CEBPA_01	C/EBPalpha	CEBPA	0.0704602	71
V$P53_02	p53	TP53	0.605075	66
V$IRF1_Q6_01	IRF-1	IRF1	0.337975	48
V$CDP_04	CDP	CUX1	0.361646	38
V$GRE_C	GR	NR3C1	0.155064	16

hsa-mir-519e

Matrix_id	transcription factor	Gene	PCC	Occurrence
V$PUR1_Q4	PUR1	PURA	0.16005	263
V$PARP_Q4	PARP	PARP1	0.0995149	254
V$GKLF_Q4	GKLF	KLF4	0.567492	246
V$MAFB_01	MAFB	MAFB	0.300398	241
V$P300_01	p300	EP300	0.349573	236
V$SMAD4_Q6_01	Smad4	SMAD4	0.308843	223
V$GR_Q6	GR	NR3C1	0.157584	201
V$TBX5_02	TBX5	TBX5	0.0788559	200
V$YY1_01	YY1	YY1	0.367543	197
V$AML1_Q6	AML1	RUNX1	0.00174447	188
V$DLX5_01	dlx5	DLX5	0.691609	178
V$YY1_Q6	YY1	YY1	0.367543	176
V$YY1_Q6_02	YY1	YY1	0.367543	176
V$GATA1_02	GATA-1	GATA1	0.0170848	173
V$GATA1_06	GATA-1	GATA1	0.0170848	165
V$GATA2_02	GATA-2	GATA2	0.695429	165
V$GATA1_05	GATA-1	GATA1	0.0170848	165
V$ING4_01	ING4	ING4	0.116608	160
V$PARP_Q3	PARP	PARP1	0.0995149	159
V$TBX5_01	TBX5	TBX5	0.0788559	159

V$SP1_Q6	Sp1	SP1	0.34844	156
V$GATA1_04	GATA-1	GATA1	0.0170848	148
V$HMGIY_01	HMGIY	HMGA1	0.341965	145
V$GATA3_02	GATA-3	GATA3	0.912183	141
V$ARNT_01	Arnt	ARNT	0.333742	134
V$CEBPE_Q6	CEBPE	CEBPE	0.0285527	121
V$GR_01	GR	NR3C1	0.157584	119
V$TCF4_01	TCF-4	TCF7L2	0.46753	118
V$TCF4_Q5	TCF-4	TCF7L2	0.46753	111
V$PITX2_Q2	Pitx2	PITX2	0.430072	111
V$IRF7_Q3	IRF-7	IRF7	0.130981	107
V$FOXJ2_01	FOXJ2	FOXJ2	0.0461565	106
V$PITX2_01	PITX2	PITX2	0.430072	105
V$AML1_01	AML1a	RUNX1	0.00174447	105
V$CP2_01	CP2	TFCP2	0.575764	103
V$CEBPD_Q6	C/EBPdelta	CEBPD	0.438292	98
V$GATA3_03	GATA-3	GATA3	0.912183	92
V$AML1_Q4	AML1	RUNX1	0.00174447	90
V$STAT3_03	STAT3	STAT3	0.146628	76
V$CEBPB_Q6	C/EBPbeta	CEBPB	0.597165	76
V$FOXO4_02	FOXO4	FOXO4	0.956047	75
V$CEBPA_01	C/EBPalpha	CEBPA	0.0713893	72
V$P53_02	p53	TP53	0.605859	66
V$IRF1_Q6_01	IRF-1	IRF1	0.338402	49
V$CDP_04	CDP	CUX1	0.363955	38
V$GRE_C	GR	NR3C1	0.157584	16

----

hsa-mir-520a

Matrix_id	transcription factor	Gene	PCC	Occurrence
V$PUR1_Q4	PUR1	PURA	0.16005	263
V$PARP_Q4	PARP	PARP1	0.0995149	254
V$GKLF_Q4	GKLF	KLF4	0.567492	246
V$MAFB_01	MAFB	MAFB	0.300398	241
V$P300_01	p300	EP300	0.349573	236
V$SMAD4_Q6_01	Smad4	SMAD4	0.308843	223
V$GR_Q6	GR	NR3C1	0.157584	201
V$TBX5_02	TBX5	TBX5	0.0788559	200
V$YY1_01	YY1	YY1	0.367543	197
V$AML1_Q6	AML1	RUNX1	0.00174447	188
V$DLX5_01	dlx5	DLX5	0.691609	178
V$YY1_Q6	YY1	YY1	0.367543	176
V$YY1_Q6_02	YY1	YY1	0.367543	176

V$GATA1_02	GATA-1	GATA1	0.0170848	173
V$GATA1_06	GATA-1	GATA1	0.0170848	165
V$GATA2_02	GATA-2	GATA2	0.695429	165
V$GATA1_05	GATA-1	GATA1	0.0170848	165
V$ING4_01	ING4	ING4	0.116608	160
V$PARP_Q3	PARP	PARP1	0.0995149	159
V$TBX5_01	TBX5	TBX5	0.0788559	159
V$SP1_Q6	Sp1	SP1	0.34844	156
V$GATA1_04	GATA-1	GATA1	0.0170848	148
V$HMGIY_01	HMGIY	HMGA1	0.341965	145
V$GATA3_02	GATA-3	GATA3	0.912183	141
V$ARNT_01	Arnt	ARNT	0.333742	134
V$CEBPE_Q6	CEBPE	CEBPE	0.0285527	121
V$GR_01	GR	NR3C1	0.157584	119
V$TCF4_01	TCF-4	TCF7L2	0.46753	118
V$TCF4_Q5	TCF-4	TCF7L2	0.46753	111
V$PITX2_Q2	Pitx2	PITX2	0.430072	111
V$IRF7_Q3	IRF-7	IRF7	0.130981	107
V$FOXJ2_01	FOXJ2	FOXJ2	0.0461565	106
V$PITX2_01	PITX2	PITX2	0.430072	105
V$AML1_01	AML1a	RUNX1	0.00174447	105
V$CP2_01	CP2	TFCP2	0.575764	103
V$CEBPD_Q6	C/EBPdelta	CEBPD	0.438292	98
V$GATA3_03	GATA-3	GATA3	0.912183	92
V$AML1_Q4	AML1	RUNX1	0.00174447	90
V$STAT3_03	STAT3	STAT3	0.146628	76
V$CEBPB_Q6	C/EBPbeta	CEBPB	0.597165	76
V$FOXO4_02	FOXO4	FOXO4	0.956047	75
V$CEBPA_01	C/EBPalpha	CEBPA	0.0713893	72
V$P53_02	p53	TP53	0.605859	66
V$IRF1_Q6_01	IRF-1	IRF1	0.338402	49
V$CDP_04	CDP	CUX1	0.363955	38
V$GRE_C	GR	NR3C1	0.157584	16

hsa-mir-520b

Matrix_id	transcription factor	Gene	PCC	Occurrence
V$PUR1_Q4	PUR1	PURA	0.129834	256
V$PARP_Q4	PARP	PARP1	0.128049	248
V$GKLF_Q4	GKLF	KLF4	0.586135	240
V$MAFB_01	MAFB	MAFB	0.262109	235
V$P300_01	p300	EP300	0.357818	228
V$SMAD4_Q6_01	Smad4	SMAD4	0.306016	218

V$TBX5_02	TBX5	TBX5	0.0831157	199
V$GR_Q6	GR	NR3C1	0.126488	196
V$YY1_01	YY1	YY1	0.389756	190
V$AML1_Q6	AML1	RUNX1	0.0271627	182
V$DLX5_01	dlx5	DLX5	0.732704	176
V$YY1_Q6	YY1	YY1	0.389756	172
V$YY1_Q6_02	YY1	YY1	0.389756	171
V$GATA1_02	GATA-1	GATA1	0.0408367	171
V$GATA1_05	GATA-1	GATA1	0.0408367	164
V$GATA1_06	GATA-1	GATA1	0.0408367	164
V$GATA2_02	GATA-2	GATA2	0.681665	164
V$TBX5_01	TBX5	TBX5	0.0831157	158
V$E12_Q6	E12	TCF3	0.0161893	153
V$E2A_Q6	E2A	TCF3	0.0161893	153
V$ING4_01	ING4	ING4	0.130733	152
V$SP1_Q6	Sp1	SP1	0.352173	152
V$PARP_Q3	PARP	PARP1	0.128049	152
V$E47_02	E47	TCF3	0.0161893	151
V$GATA1_04	GATA-1	GATA1	0.0408367	145
V$HMGIY_01	HMGIY	HMGA1	0.432412	143
V$GATA3_02	GATA-3	GATA3	0.897683	143
V$ARNT_01	Arnt	ARNT	0.312448	131
V$E2A_Q2	E2A	TCF3	0.0161893	131
V$TCF4_01	TCF-4	TCF7L2	0.47529	117
V$CEBPE_Q6	CEBPE	CEBPE	0.0150726	117
V$GR_01	GR	NR3C1	0.126488	113
V$PITX2_Q2	Pitx2	PITX2	0.413229	108
V$TCF4_Q5	TCF-4	TCF7L2	0.47529	107
V$IRF7_Q3	IRF-7	IRF7	0.0937712	104
V$AML1_01	AML1a	RUNX1	0.0271627	104
V$FOXJ2_01	FOXJ2	FOXJ2	0.0851587	103
V$PITX2_01	PITX2	PITX2	0.413229	102
V$CP2_01	CP2	TFCP2	0.561315	100
V$CEBPD_Q6	C/EBPdelta	CEBPD	0.401777	94
V$AML1_Q4	AML1	RUNX1	0.0271627	91
V$GATA3_03	GATA-3	GATA3	0.897683	88
V$STAT3_03	STAT3	STAT3	0.127716	75
V$CEBPB_Q6	C/EBPbeta	CEBPB	0.566453	74
V$FOXO4_02	FOXO4	FOXO4	0.953303	71
V$CEBPA_01	C/EBPalpha	CEBPA	0.0591953	67
V$P53_02	p53	TP53	0.622868	63
V$IRF1_Q6_01	IRF-1	IRF1	0.335772	46
V$CDP_04	CDP	CUX1	0.339614	37
V$GRE_C	GR	NR3C1	0.126488	14

----

hsa-mir-520c

Matrix_id	transcription factor	Gene	PCC	Occurrence
V$PUR1_Q4	PUR1	PURA	0.121906	255
V$PARP_Q4	PARP	PARP1	0.134743	247
V$GKLF_Q4	GKLF	KLF4	0.588919	239
V$MAFB_01	MAFB	MAFB	0.251754	234
V$P300_01	p300	EP300	0.358738	227
V$SMAD4_Q6_01	Smad4	SMAD4	0.304344	217
V$TBX5_02	TBX5	TBX5	0.0839126	199
V$GR_Q6	GR	NR3C1	0.118351	196
V$YY1_01	YY1	YY1	0.39405	189
V$AML1_Q6	AML1	RUNX1	0.0334012	182
V$DLX5_01	dlx5	DLX5	0.740611	176
V$GATA1_02	GATA-1	GATA1	0.0466174	171
V$YY1_Q6	YY1	YY1	0.39405	171
V$YY1_Q6_02	YY1	YY1	0.39405	170
V$GATA1_06	GATA-1	GATA1	0.0466174	164
V$GATA2_02	GATA-2	GATA2	0.676084	164
V$GATA1_05	GATA-1	GATA1	0.0466174	164
V$TBX5_01	TBX5	TBX5	0.0839126	158
V$PARP_Q3	PARP	PARP1	0.134743	153
V$E2A_Q6	E2A	TCF3	0.0315502	153
V$E12_Q6	E12	TCF3	0.0315502	153
V$SP1_Q6	Sp1	SP1	0.351988	151
V$E47_02	E47	TCF3	0.0315502	151
V$ING4_01	ING4	ING4	0.133834	150
V$GATA1_04	GATA-1	GATA1	0.0466174	145
V$GATA3_02	GATA-3	GATA3	0.891235	144
V$HMGIY_01	HMGIY	HMGA1	0.453548	142
V$E2A_Q2	E2A	TCF3	0.0315502	131
V$ARNT_01	Arnt	ARNT	0.306161	130
V$TCF4_01	TCF-4	TCF7L2	0.475717	116
V$CEBPE_Q6	CEBPE	CEBPE	0.0116709	116
V$GR_01	GR	NR3C1	0.118351	113
V$PITX2_Q2	Pitx2	PITX2	0.407731	108
V$TCF4_Q5	TCF-4	TCF7L2	0.475717	107
V$AML1_01	AML1a	RUNX1	0.0334012	104
V$FOXJ2_01	FOXJ2	FOXJ2	0.0945935	103
V$IRF7_Q3	IRF-7	IRF7	0.0842163	102
V$PITX2_01	PITX2	PITX2	0.407731	102
V$CP2_01	CP2	TFCP2	0.555944	100
V$CEBPD_Q6	C/EBPdelta	CEBPD	0.391421	92
V$AML1_Q4	AML1	RUNX1	0.0334012	91

V$GATA3_03	GATA-3	GATA3	0.891235	88
V$STAT3_03	STAT3	STAT3	0.122606	76
V$CEBPB_Q6	C/EBPbeta	CEBPB	0.557019	72
V$FOXO4_02	FOXO4	FOXO4	0.949605	70
V$CEBPA_01	C/EBPalpha	CEBPA	0.055974	65
V$P53_02	p53	TP53	0.625129	63
V$IRF1_Q6_01	IRF-1	IRF1	0.334055	45
V$CDP_04	CDP	CUX1	0.332484	36
V$GRE_C	GR	NR3C1	0.118351	14

hsa-mir-520e

Matrix_id	transcription factor	Gene	PCC	Occurrence
V$PUR1_Q4	PUR1	PURA	0.141146	262
V$PARP_Q4	PARP	PARP1	0.11796	254
V$GKLF_Q4	GKLF	KLF4	0.580731	244
V$MAFB_01	MAFB	MAFB	0.276674	240
V$P300_01	p300	EP300	0.355662	234
V$SMAD4_Q6_01	Smad4	SMAD4	0.307769	223
V$TBX5_02	TBX5	TBX5	0.0817609	201
V$GR_Q6	GR	NR3C1	0.138112	201
V$YY1_01	YY1	YY1	0.382588	195
V$AML1_Q6	AML1	RUNX1	0.0179696	187
V$DLX5_01	dlx5	DLX5	0.719471	180
V$YY1_Q6	YY1	YY1	0.382588	177
V$YY1_Q6_02	YY1	YY1	0.382588	176
V$GATA1_02	GATA-1	GATA1	0.0322829	173
V$GATA1_05	GATA-1	GATA1	0.0322829	167
V$GATA1_06	GATA-1	GATA1	0.0322829	167
V$GATA2_02	GATA-2	GATA2	0.68829	167
V$TBX5_01	TBX5	TBX5	0.0817609	159
V$ING4_01	ING4	ING4	0.125898	156
V$PARP_Q3	PARP	PARP1	0.11796	156
V$SP1_Q6	Sp1	SP1	0.351646	155
V$GATA1_04	GATA-1	GATA1	0.0322829	147
V$HMGIY_01	HMGIY	HMGA1	0.400494	147
V$GATA3_02	GATA-3	GATA3	0.905088	144
V$ARNT_01	Arnt	ARNT	0.320942	134
V$CEBPE_Q6	CEBPE	CEBPE	0.0200177	121
V$TCF4_01	TCF-4	TCF7L2	0.473588	120
V$GR_01	GR	NR3C1	0.138112	117
V$TCF4_Q5	TCF-4	TCF7L2	0.473588	111
V$PITX2_Q2	Pitx2	PITX2	0.420341	111

V$IRF7_Q3	IRF-7	IRF7	0.107545	107
V$FOXJ2_01	FOXJ2	FOXJ2	0.0711555	107
V$AML1_01	AML1a	RUNX1	0.0179696	106
V$CP2_01	CP2	TFCP2	0.567905	103
V$PITX2_01	PITX2	PITX2	0.420341	103
V$CEBPD_Q6	C/EBPdelta	CEBPD	0.416026	98
V$AML1_Q4	AML1	RUNX1	0.0179696	92
V$GATA3_03	GATA-3	GATA3	0.905088	91
V$STAT3_03	STAT3	STAT3	0.134906	77
V$CEBPB_Q6	C/EBPbeta	CEBPB	0.578978	77
V$FOXO4_02	FOXO4	FOXO4	0.956558	75
V$CEBPA_01	C/EBPalpha	CEBPA	0.0637764	71
V$P53_02	p53	TP53	0.618147	66
V$IRF1_Q6_01	IRF-1	IRF1	0.337524	47
V$CDP_04	CDP	CUX1	0.349283	38
V$GRE_C	GR	NR3C1	0.138112	14

----

hsa-mir-520f

Matrix_id	transcription factor	Gene	PCC	Occurrence
V$PUR1_Q4	PUR1	PURA	0.111474	249
V$PARP_Q4	PARP	PARP1	0.143112	241
V$GKLF_Q4	GKLF	KLF4	0.591415	233
V$MAFB_01	MAFB	MAFB	0.237958	228
V$P300_01	p300	EP300	0.359259	221
V$SMAD4_Q6_01	Smad4	SMAD4	0.30163	212
V$NFAT4_Q3	NF-AT4	NFATC3	0.00337426	207
V$TBX5_02	TBX5	TBX5	0.0847834	194
V$GR_Q6	GR	NR3C1	0.107657	190
V$YY1_01	YY1	YY1	0.398849	187
V$AML1_Q6	AML1	RUNX1	0.0413699	179
V$DLX5_01	dlx5	DLX5	0.749421	170
V$YY1_Q6	YY1	YY1	0.398849	166
V$YY1_Q6_02	YY1	YY1	0.398849	165
V$GATA1_02	GATA-1	GATA1	0.0539723	165
V$GATA2_02	GATA-2	GATA2	0.66765	158
V$GATA1_06	GATA-1	GATA1	0.0539723	158
V$GATA1_05	GATA-1	GATA1	0.0539723	158
V$TBX5_01	TBX5	TBX5	0.0847834	155
V$E12_Q6	E12	TCF3	0.0512653	149
V$E2A_Q6	E2A	TCF3	0.0512653	149
V$PARP_Q3	PARP	PARP1	0.143112	148
V$SP1_Q6	Sp1	SP1	0.351098	148

V$E47_02	E47	TCF3	0.0512653	147
V$ING4_01	ING4	ING4	0.137579	146
V$HMGIY_01	HMGIY	HMGA1	0.479917	139
V$GATA1_04	GATA-1	GATA1	0.0539723	139
V$GATA3_02	GATA-3	GATA3	0.881288	138
V$ARNT_01	Arnt	ARNT	0.297503	129
V$E2A_Q2	E2A	TCF3	0.0512653	127
V$TCF4_01	TCF-4	TCF7L2	0.475388	114
V$CEBPE_Q6	CEBPE	CEBPE	0.00727047	113
V$GR_01	GR	NR3C1	0.107657	111
V$TCF4_Q5	TCF-4	TCF7L2	0.475388	106
V$PITX2_Q2	Pitx2	PITX2	0.399899	104
V$AML1_01	AML1a	RUNX1	0.0413699	102
V$FOXJ2_01	FOXJ2	FOXJ2	0.106564	101
V$PITX2_01	PITX2	PITX2	0.399899	99
V$CP2_01	CP2	TFCP2	0.548003	98
V$IRF7_Q3	IRF-7	IRF7	0.0717609	98
V$CEBPD_Q6	C/EBPdelta	CEBPD	0.377366	91
V$AML1_Q4	AML1	RUNX1	0.0413699	89
V$GATA3_03	GATA-3	GATA3	0.881288	86
V$STAT3_03	STAT3	STAT3	0.115802	73
V$CEBPB_Q6	C/EBPbeta	CEBPB	0.543846	71
V$FOXO4_02	FOXO4	FOXO4	0.943089	69
V$CEBPA_01	C/EBPalpha	CEBPA	0.0517239	64
V$P53_02	p53	TP53	0.626885	60
V$IRF1_Q6_01	IRF-1	IRF1	0.331228	43
V$CDP_04	CDP	CUX1	0.32269	36
V$GRE_C	GR	NR3C1	0.107657	14

----

hsa-mir-521-2

Matrix_id	transcription factor	Gene	PCC	Occurrence
V$PUR1_Q4	PUR1	PURA	0.16005	263
V$PARP_Q4	PARP	PARP1	0.0995149	254
V$GKLF_Q4	GKLF	KLF4	0.567492	246
V$MAFB_01	MAFB	MAFB	0.300398	241
V$P300_01	p300	EP300	0.349573	236
V$SMAD4_Q6_01	Smad4	SMAD4	0.308843	223
V$GR_Q6	GR	NR3C1	0.157584	201
V$TBX5_02	TBX5	TBX5	0.0788559	200
V$YY1_01	YY1	YY1	0.367543	197
V$AML1_Q6	AML1	RUNX1	0.00174447	188
V$DLX5_01	dlx5	DLX5	0.691609	178

V$YY1_Q6	YY1	YY1	0.367543	176
V$YY1_Q6_02	YY1	YY1	0.367543	176
V$GATA1_02	GATA-1	GATA1	0.0170848	173
V$GATA1_06	GATA-1	GATA1	0.0170848	165
V$GATA2_02	GATA-2	GATA2	0.695429	165
V$GATA1_05	GATA-1	GATA1	0.0170848	165
V$ING4_01	ING4	ING4	0.116608	160
V$PARP_Q3	PARP	PARP1	0.0995149	159
V$TBX5_01	TBX5	TBX5	0.0788559	159
V$SP1_Q6	Sp1	SP1	0.34844	156
V$GATA1_04	GATA-1	GATA1	0.0170848	148
V$HMGIY_01	HMGIY	HMGA1	0.341965	145
V$GATA3_02	GATA-3	GATA3	0.912183	141
V$ARNT_01	Arnt	ARNT	0.333742	134
V$CEBPE_Q6	CEBPE	CEBPE	0.0285527	121
V$GR_01	GR	NR3C1	0.157584	119
V$TCF4_01	TCF-4	TCF7L2	0.46753	118
V$TCF4_Q5	TCF-4	TCF7L2	0.46753	111
V$PITX2_Q2	Pitx2	PITX2	0.430072	111
V$IRF7_Q3	IRF-7	IRF7	0.130981	107
V$FOXJ2_01	FOXJ2	FOXJ2	0.0461565	106
V$PITX2_01	PITX2	PITX2	0.430072	105
V$AML1_01	AML1a	RUNX1	0.00174447	105
V$CP2_01	CP2	TFCP2	0.575764	103
V$CEBPD_Q6	C/EBPdelta	CEBPD	0.438292	98
V$GATA3_03	GATA-3	GATA3	0.912183	92
V$AML1_Q4	AML1	RUNX1	0.00174447	90
V$STAT3_03	STAT3	STAT3	0.146628	76
V$CEBPB_Q6	C/EBPbeta	CEBPB	0.597165	76
V$FOXO4_02	FOXO4	FOXO4	0.956047	75
V$CEBPA_01	C/EBPalpha	CEBPA	0.0713893	72
V$P53_02	p53	TP53	0.605859	66
V$IRF1_Q6_01	IRF-1	IRF1	0.338402	49
V$CDP_04	CDP	CUX1	0.363955	38
V$GRE_C	GR	NR3C1	0.157584	16

hsa-mir-523

Matrix_id	transcription factor	Gene	PCC	Occurrence
V$PUR1_Q4	PUR1	PURA	0.16005	263
V$PARP_Q4	PARP	PARP1	0.0995149	254
V$GKLF_Q4	GKLF	KLF4	0.567492	246
V$MAFB_01	MAFB	MAFB	0.300398	241

V$P300_01	p300	EP300	0.349573	236
V$SMAD4_Q6_01	Smad4	SMAD4	0.308843	223
V$GR_Q6	GR	NR3C1	0.157584	201
V$TBX5_02	TBX5	TBX5	0.0788559	200
V$YY1_01	YY1	YY1	0.367543	197
V$AML1_Q6	AML1	RUNX1	0.00174447	188
V$DLX5_01	dlx5	DLX5	0.691609	178
V$YY1_Q6	YY1	YY1	0.367543	176
V$YY1_Q6_02	YY1	YY1	0.367543	176
V$GATA1_02	GATA-1	GATA1	0.0170848	173
V$GATA1_06	GATA-1	GATA1	0.0170848	165
V$GATA2_02	GATA-2	GATA2	0.695429	165
V$GATA1_05	GATA-1	GATA1	0.0170848	165
V$ING4_01	ING4	ING4	0.116608	160
V$PARP_Q3	PARP	PARP1	0.0995149	159
V$TBX5_01	TBX5	TBX5	0.0788559	159
V$SP1_Q6	Sp1	SP1	0.34844	156
V$GATA1_04	GATA-1	GATA1	0.0170848	148
V$HMGIY_01	HMGIY	HMGA1	0.341965	145
V$GATA3_02	GATA-3	GATA3	0.912183	141
V$ARNT_01	Arnt	ARNT	0.333742	134
V$CEBPE_Q6	CEBPE	CEBPE	0.0285527	121
V$GR_01	GR	NR3C1	0.157584	119
V$TCF4_01	TCF-4	TCF7L2	0.46753	118
V$TCF4_Q5	TCF-4	TCF7L2	0.46753	111
V$PITX2_Q2	Pitx2	PITX2	0.430072	111
V$IRF7_Q3	IRF-7	IRF7	0.130981	107
V$FOXJ2_01	FOXJ2	FOXJ2	0.0461565	106
V$PITX2_01	PITX2	PITX2	0.430072	105
V$AML1_01	AML1a	RUNX1	0.00174447	105
V$CP2_01	CP2	TFCP2	0.575764	103
V$CEBPD_Q6	C/EBPdelta	CEBPD	0.438292	98
V$GATA3_03	GATA-3	GATA3	0.912183	92
V$AML1_Q4	AML1	RUNX1	0.00174447	90
V$STAT3_03	STAT3	STAT3	0.146628	76
V$CEBPB_Q6	C/EBPbeta	CEBPB	0.597165	76
V$FOXO4_02	FOXO4	FOXO4	0.956047	75
V$CEBPA_01	C/EBPalpha	CEBPA	0.0713893	72
V$P53_02	p53	TP53	0.605859	66
V$IRF1_Q6_01	IRF-1	IRF1	0.338402	49
V$CDP_04	CDP	CUX1	0.363955	38
V$GRE_C	GR	NR3C1	0.157584	16

----

hsa-mir-524

Matrix_id	transcription factor	Gene	PCC	Occurrence
V$PUR1_Q4	PUR1	PURA	0.16005	263
V$PARP_Q4	PARP	PARP1	0.0995149	254
V$GKLF_Q4	GKLF	KLF4	0.567492	246
V$MAFB_01	MAFB	MAFB	0.300398	241
V$P300_01	p300	EP300	0.349573	236
V$SMAD4_Q6_01	Smad4	SMAD4	0.308843	223
V$GR_Q6	GR	NR3C1	0.157584	201
V$TBX5_02	TBX5	TBX5	0.0788559	200
V$YY1_01	YY1	YY1	0.367543	197
V$AML1_Q6	AML1	RUNX1	0.00174447	188
V$DLX5_01	dlx5	DLX5	0.691609	178
V$YY1_Q6	YY1	YY1	0.367543	176
V$YY1_Q6_02	YY1	YY1	0.367543	176
V$GATA1_02	GATA-1	GATA1	0.0170848	173
V$GATA1_06	GATA-1	GATA1	0.0170848	165
V$GATA2_02	GATA-2	GATA2	0.695429	165
V$GATA1_05	GATA-1	GATA1	0.0170848	165
V$ING4_01	ING4	ING4	0.116608	160
V$PARP_Q3	PARP	PARP1	0.0995149	159
V$TBX5_01	TBX5	TBX5	0.0788559	159
V$SP1_Q6	Sp1	SP1	0.34844	156
V$GATA1_04	GATA-1	GATA1	0.0170848	148
V$HMGIY_01	HMGIY	HMGA1	0.341965	145
V$GATA3_02	GATA-3	GATA3	0.912183	141
V$ARNT_01	Arnt	ARNT	0.333742	134
V$CEBPE_Q6	CEBPE	CEBPE	0.0285527	121
V$GR_01	GR	NR3C1	0.157584	119
V$TCF4_01	TCF-4	TCF7L2	0.46753	118
V$TCF4_Q5	TCF-4	TCF7L2	0.46753	111
V$PITX2_Q2	Pitx2	PITX2	0.430072	111
V$IRF7_Q3	IRF-7	IRF7	0.130981	107
V$FOXJ2_01	FOXJ2	FOXJ2	0.0461565	106
V$PITX2_01	PITX2	PITX2	0.430072	105
V$AML1_01	AML1a	RUNX1	0.00174447	105
V$CP2_01	CP2	TFCP2	0.575764	103
V$CEBPD_Q6	C/EBPdelta	CEBPD	0.438292	98
V$GATA3_03	GATA-3	GATA3	0.912183	92
V$AML1_Q4	AML1	RUNX1	0.00174447	90
V$STAT3_03	STAT3	STAT3	0.146628	76
V$CEBPB_Q6	C/EBPbeta	CEBPB	0.597165	76
V$FOXO4_02	FOXO4	FOXO4	0.956047	75
V$CEBPA_01	C/EBPalpha	CEBPA	0.0713893	72
V$P53_02	p53	TP53	0.605859	66

V$IRF1_Q6_01	IRF-1	IRF1	0.338402	49
V$CDP_04	CDP	CUX1	0.363955	38
V$GRE_C	GR	NR3C1	0.157584	16

----

hsa-mir-525

Matrix_id	transcription factor	Gene	PCC	Occurrence
V$PUR1_Q4	PUR1	PURA	0.16005	263
V$PARP_Q4	PARP	PARP1	0.0995149	254
V$GKLF_Q4	GKLF	KLF4	0.567492	246
V$MAFB_01	MAFB	MAFB	0.300398	241
V$P300_01	p300	EP300	0.349573	236
V$SMAD4_Q6_01	Smad4	SMAD4	0.308843	223
V$GR_Q6	GR	NR3C1	0.157584	201
V$TBX5_02	TBX5	TBX5	0.0788559	200
V$YY1_01	YY1	YY1	0.367543	197
V$AML1_Q6	AML1	RUNX1	0.00174447	188
V$DLX5_01	dlx5	DLX5	0.691609	178
V$YY1_Q6	YY1	YY1	0.367543	176
V$YY1_Q6_02	YY1	YY1	0.367543	176
V$GATA1_02	GATA-1	GATA1	0.0170848	173
V$GATA1_06	GATA-1	GATA1	0.0170848	165
V$GATA2_02	GATA-2	GATA2	0.695429	165
V$GATA1_05	GATA-1	GATA1	0.0170848	165
V$ING4_01	ING4	ING4	0.116608	160
V$PARP_Q3	PARP	PARP1	0.0995149	159
V$TBX5_01	TBX5	TBX5	0.0788559	159
V$SP1_Q6	Sp1	SP1	0.34844	156
V$GATA1_04	GATA-1	GATA1	0.0170848	148
V$HMGIY_01	HMGIY	HMGA1	0.341965	145
V$GATA3_02	GATA-3	GATA3	0.912183	141
V$ARNT_01	Arnt	ARNT	0.333742	134
V$CEBPE_Q6	CEBPE	CEBPE	0.0285527	121
V$GR_01	GR	NR3C1	0.157584	119
V$TCF4_01	TCF-4	TCF7L2	0.46753	118
V$TCF4_Q5	TCF-4	TCF7L2	0.46753	111
V$PITX2_Q2	Pitx2	PITX2	0.430072	111
V$IRF7_Q3	IRF-7	IRF7	0.130981	107
V$FOXJ2_01	FOXJ2	FOXJ2	0.0461565	106
V$PITX2_01	PITX2	PITX2	0.430072	105
V$AML1_01	AML1a	RUNX1	0.00174447	105
V$CP2_01	CP2	TFCP2	0.575764	103
V$CEBPD_Q6	C/EBPdelta	CEBPD	0.438292	98

V$GATA3_03	GATA-3	GATA3	0.912183	92
V$AML1_Q4	AML1	RUNX1	0.00174447	90
V$STAT3_03	STAT3	STAT3	0.146628	76
V$CEBPB_Q6	C/EBPbeta	CEBPB	0.597165	76
V$FOXO4_02	FOXO4	FOXO4	0.956047	75
V$CEBPA_01	C/EBPalpha	CEBPA	0.0713893	72
V$P53_02	p53	TP53	0.605859	66
V$IRF1_Q6_01	IRF-1	IRF1	0.338402	49
V$CDP_04	CDP	CUX1	0.363955	38
V$GRE_C	GR	NR3C1	0.157584	16

----

hsa-mir-526a-1

Matrix_id	transcription factor	Gene	PCC	Occurrence
V$PUR1_Q4	PUR1	PURA	0.16005	263
V$PARP_Q4	PARP	PARP1	0.0995149	254
V$GKLF_Q4	GKLF	KLF4	0.567492	246
V$MAFB_01	MAFB	MAFB	0.300398	241
V$P300_01	p300	EP300	0.349573	236
V$SMAD4_Q6_01	Smad4	SMAD4	0.308843	223
V$GR_Q6	GR	NR3C1	0.157584	201
V$TBX5_02	TBX5	TBX5	0.0788559	200
V$YY1_01	YY1	YY1	0.367543	197
V$AML1_Q6	AML1	RUNX1	0.00174447	188
V$DLX5_01	dlx5	DLX5	0.691609	178
V$YY1_Q6	YY1	YY1	0.367543	176
V$YY1_Q6_02	YY1	YY1	0.367543	176
V$GATA1_02	GATA-1	GATA1	0.0170848	173
V$GATA1_06	GATA-1	GATA1	0.0170848	165
V$GATA2_02	GATA-2	GATA2	0.695429	165
V$GATA1_05	GATA-1	GATA1	0.0170848	165
V$ING4_01	ING4	ING4	0.116608	160
V$PARP_Q3	PARP	PARP1	0.0995149	159
V$TBX5_01	TBX5	TBX5	0.0788559	159
V$SP1_Q6	Sp1	SP1	0.34844	156
V$GATA1_04	GATA-1	GATA1	0.0170848	148
V$HMGIY_01	HMGIY	HMGA1	0.341965	145
V$GATA3_02	GATA-3	GATA3	0.912183	141
V$ARNT_01	Arnt	ARNT	0.333742	134
V$CEBPE_Q6	CEBPE	CEBPE	0.0285527	121
V$GR_01	GR	NR3C1	0.157584	119
V$TCF4_01	TCF-4	TCF7L2	0.46753	118
V$TCF4_Q5	TCF-4	TCF7L2	0.46753	111

V$PITX2_Q2	Pitx2	PITX2	0.430072	111
V$IRF7_Q3	IRF-7	IRF7	0.130981	107
V$FOXJ2_01	FOXJ2	FOXJ2	0.0461565	106
V$PITX2_01	PITX2	PITX2	0.430072	105
V$AML1_01	AML1a	RUNX1	0.00174447	105
V$CP2_01	CP2	TFCP2	0.575764	103
V$CEBPD_Q6	C/EBPdelta	CEBPD	0.438292	98
V$GATA3_03	GATA-3	GATA3	0.912183	92
V$AML1_Q4	AML1	RUNX1	0.00174447	90
V$STAT3_03	STAT3	STAT3	0.146628	76
V$CEBPB_Q6	C/EBPbeta	CEBPB	0.597165	76
V$FOXO4_02	FOXO4	FOXO4	0.956047	75
V$CEBPA_01	C/EBPalpha	CEBPA	0.0713893	72
V$P53_02	p53	TP53	0.605859	66
V$IRF1_Q6_01	IRF-1	IRF1	0.338402	49
V$CDP_04	CDP	CUX1	0.363955	38
V$GRE_C	GR	NR3C1	0.157584	16

----

hsa-mir-526b

Matrix_id	transcription factor	Gene	PCC	Occurrence
V$PUR1_Q4	PUR1	PURA	0.16005	263
V$PARP_Q4	PARP	PARP1	0.0995149	254
V$GKLF_Q4	GKLF	KLF4	0.567492	246
V$MAFB_01	MAFB	MAFB	0.300398	241
V$P300_01	p300	EP300	0.349573	236
V$SMAD4_Q6_01	Smad4	SMAD4	0.308843	223
V$GR_Q6	GR	NR3C1	0.157584	201
V$TBX5_02	TBX5	TBX5	0.0788559	200
V$YY1_01	YY1	YY1	0.367543	197
V$AML1_Q6	AML1	RUNX1	0.00174447	188
V$DLX5_01	dlx5	DLX5	0.691609	178
V$YY1_Q6	YY1	YY1	0.367543	176
V$YY1_Q6_02	YY1	YY1	0.367543	176
V$GATA1_02	GATA-1	GATA1	0.0170848	173
V$GATA1_06	GATA-1	GATA1	0.0170848	165
V$GATA2_02	GATA-2	GATA2	0.695429	165
V$GATA1_05	GATA-1	GATA1	0.0170848	165
V$ING4_01	ING4	ING4	0.116608	160
V$PARP_Q3	PARP	PARP1	0.0995149	159
V$TBX5_01	TBX5	TBX5	0.0788559	159
V$SP1_Q6	Sp1	SP1	0.34844	156
V$GATA1_04	GATA-1	GATA1	0.0170848	148

V$HMGIY_01	HMGIY	HMGA1	0.341965	145
V$GATA3_02	GATA-3	GATA3	0.912183	141
V$ARNT_01	Arnt	ARNT	0.333742	134
V$CEBPE_Q6	CEBPE	CEBPE	0.0285527	121
V$GR_01	GR	NR3C1	0.157584	119
V$TCF4_01	TCF-4	TCF7L2	0.46753	118
V$TCF4_Q5	TCF-4	TCF7L2	0.46753	111
V$PITX2_Q2	Pitx2	PITX2	0.430072	111
V$IRF7_Q3	IRF-7	IRF7	0.130981	107
V$FOXJ2_01	FOXJ2	FOXJ2	0.0461565	106
V$PITX2_01	PITX2	PITX2	0.430072	105
V$AML1_01	AML1a	RUNX1	0.00174447	105
V$CP2_01	CP2	TFCP2	0.575764	103
V$CEBPD_Q6	C/EBPdelta	CEBPD	0.438292	98
V$GATA3_03	GATA-3	GATA3	0.912183	92
V$AML1_Q4	AML1	RUNX1	0.00174447	90
V$STAT3_03	STAT3	STAT3	0.146628	76
V$CEBPB_Q6	C/EBPbeta	CEBPB	0.597165	76
V$FOXO4_02	FOXO4	FOXO4	0.956047	75
V$CEBPA_01	C/EBPalpha	CEBPA	0.0713893	72
V$P53_02	p53	TP53	0.605859	66
V$IRF1_Q6_01	IRF-1	IRF1	0.338402	49
V$CDP_04	CDP	CUX1	0.363955	38
V$GRE_C	GR	NR3C1	0.157584	16

---

hsa-mir-7-3

Matrix_id	transcription factor	Gene	PCC	Occurrence
V$PARP_Q4	PARP	PARP1	0.0747165	43
V$GABPA_Q4	GABP-alpha	GABPA	0.0231452	34
V$YY1_01	YY1	YY1	0.0404993	34
V$MEF2C_Q4	MEF-2C	MEF2C	0.0811236	34
V$GATA6_01	GATA-6	GATA6	0.627543	32
V$HNF4A_Q6_01	HNF-4alpha	HNF4A	0.169696	32
V$YY1_Q6_02	YY1	YY1	0.0404993	31
V$YY1_Q6	YY1	YY1	0.0404993	31
V$PITX3_Q2	PITX3	PITX3	0.498045	28
V$YY1_Q6_03	YY1	YY1	0.0404993	27
V$PBX1_04	Pbx1	PBX1	0.24639	26
V$CRX_Q4	Crx	CRX	0.0679382	25
V$PARP_Q3	PARP	PARP1	0.0747165	25
V$ELK1_02	Elk-1	ELK1	0.216887	23
V$SREBP1_Q6	SREBP-1	SREBF1	0.786694	21

V$CREM_Q6	CREM	CREM	0.464418	21
V$NKX22_02	NKX2B	NKX2-2	0.116087	20
V$NKX2B_Q3	NKX2B	NKX2-2	0.116087	18
V$STAT3_03	STAT3	STAT3	0.0791977	18
V$IRF8_Q6	IRF-8	IRF8	0.117009	17
V$FOXJ2_01	FOXJ2	FOXJ2	0.155797	16
V$CP2_01	CP2	TFCP2	0.218646	14
V$HNF6_Q6	HNF6	ONECUT1	0.432245	14
V$ATF4_Q6	ATF-4	ATF4	0.0365872	13
V$OC2_Q3	OC-2	ONECUT2	0.122353	12
V$STAT1_05	STAT1	STAT1	0.0326217	10
V$CIZ_01	CIZ	ZNF384	0.276404	10
V$STAT1_Q6	STAT1	STAT1	0.0326217	7
V$FOXJ2_02	FOXJ2	FOXJ2	0.155797	6

---

hsa-mir-9-1

Matrix_id	transcription factor	Gene	PCC	Occurrence
V$PUR1_Q4	PUR1	PURA	0.418516	494
V$PARP_Q4	PARP	PARP1	0.420723	450
V$ZIC3_01	Zic3	ZIC3	0.195803	447
V$SOX9_Q4	SOX9	SOX9	0.239554	395
V$TBP_Q6	TBP	TBP	0.0239134	387
V$YY1_01	YY1	YY1	0.101384	361
V$GR_Q6	GR	NR3C1	0.0589527	355
V$MEF2C_Q4	MEF-2C	MEF2C	0.810655	352
V$YY1_Q6	YY1	YY1	0.101384	333
V$YY1_Q6_02	YY1	YY1	0.101384	333
V$ZBP89_Q4	ZBP89	ZNF148	0.115985	296
V$PARP_Q3	PARP	PARP1	0.420723	289
V$HNF3B_Q6	HNF-3beta	FOXA2	0.0570425	275
V$FOXO3A_Q1	FOXO3A	FOXO3	0.0449524	265
V$PBX1_04	Pbx1	PBX1	0.236112	262
V$PITX3_Q2	PITX3	PITX3	0.0440164	261
V$SREBP1_Q6	SREBP-1	SREBF1	0.0269379	218
V$IRF8_Q6	IRF-8	IRF8	0.0927724	216
V$CEBPG_Q6_01	C/EBPgamma	CEBPG	0.127674	157
V$FAC1_01	FAC1	BPTF	0.142423	152
V$LHX3b_01	LHX3b	LHX3	0.0166661	150
V$NURR1_Q3	NURR1	NR4A2	0.135327	150
V$YY1_02	YY1	YY1	0.101384	143
V$HOXB8_01	HOXB8	HOXB8	0.317406	106
V$YY1_03	YY1	YY1	0.101384	86

V$ERR3_Q2	ERR3	ESRRG	0.102072	77
V$ERR3_Q2_01	ERR3	ESRRG	0.102072	60

----

hsa-mir-9-2

Matrix_id	transcription factor	Gene	PCC	Occurrence
V$PUR1_Q4	PUR1	PURA	0.418516	494
V$MAFB_01	MAFB	MAFB	0.166082	456
V$PARP_Q4	PARP	PARP1	0.420723	450
V$ZIC3_01	Zic3	ZIC3	0.195803	447
V$ETS2_Q6	c-Ets-2	ETS2	0.189664	410
V$GABPA_Q4	GABP-alpha	GABPA	0.13762	397
V$SOX9_Q4	SOX9	SOX9	0.239554	395
V$TBP_Q6	TBP	TBP	0.0239134	387
V$AP4_Q6_02	AP-4	TFAP4	0.411949	369
V$MAZ_Q6	MAZ	MAZ	0.224203	363
V$YY1_01	YY1	YY1	0.101384	361
V$MEF2C_Q4	MEF-2C	MEF2C	0.810655	352
V$YY1_Q6	YY1	YY1	0.101384	333
V$YY1_Q6_02	YY1	YY1	0.101384	333
V$DLX5_01	dlx5	DLX5	0.009445	328
V$SOX9_B1	SOX9	SOX9	0.239554	324
V$SRY_02	SRY	SRY	0.176672	295
V$PARP_Q3	PARP	PARP1	0.420723	289
V$ING4_01	ING4	ING4	0.692803	286
V$HNF3B_Q6	HNF-3beta	FOXA2	0.0570425	275
V$FOXO3A_Q1	FOXO3A	FOXO3	0.0449524	265
V$PBX1_04	Pbx1	PBX1	0.236112	262
V$HIF1A_Q6	HIF-1alpha	HIF1A	0.00335261	259
V$AP4_Q5	AP-4	TFAP4	0.411949	255
V$YY1_Q6_03	YY1	YY1	0.101384	251
V$AP4_Q6	AP-4	TFAP4	0.411949	233
V$IRF8_Q6	IRF-8	IRF8	0.0927724	216
V$CREM_Q6	CREM	CREM	0.0272754	214
V$SOX10_Q6	SOX10	SOX10	0.724748	209
V$AP4_Q6_01	AP-4	TFAP4	0.411949	162
V$CEBPG_Q6_01	C/EBPgamma	CEBPG	0.127674	157
V$NURR1_Q3	NURR1	NR4A2	0.135327	150
V$DBP_Q6_01	DBP	DBP	0.267264	148
V$MSX1_01	Msx-1	MSX1	0.188893	119
V$AP4_01	AP-4	TFAP4	0.411949	107
V$HOXB8_01	HOXB8	HOXB8	0.317406	106
V$DEC2_Q2	2-Dec	BHLHE41	0.67018	97

V$USF2_Q6	USF2	USF2	0.33501	87
V$POU6F1_03	POU6F1	POU6F1	0.399284	66
V$POU6F1_02	POU6F1	POU6F1	0.399284	52
V$ZID_01	ZID	ZBTB6	0.00176523	46
V$POU6F1_01	POU6F1	POU6F1	0.399284	34
V$CDP_02	CDP	CUX1	0.0621111	15

hsa-mir-9-3

Matrix_id	transcription factor	Gene	PCC	Occurrence
V$PUR1_Q4	PUR1	PURA	0.418516	494
V$MAFB_01	MAFB	MAFB	0.166082	456
V$PARP_Q4	PARP	PARP1	0.420723	450
V$ETS2_Q6	c-Ets-2	ETS2	0.189664	410
V$GABPA_Q4	GABP-alpha	GABPA	0.13762	397
V$TBP_Q6	TBP	TBP	0.0239134	387
V$AP4_Q6_02	AP-4	TFAP4	0.411949	369
V$YY1_01	YY1	YY1	0.101384	361
V$GR_Q6	GR	NR3C1	0.0589527	355
V$ETS2_B	c-Ets-2	ETS2	0.189664	334
V$YY1_Q6	YY1	YY1	0.101384	333
V$YY1_Q6_02	YY1	YY1	0.101384	333
V$ZBP89_Q4	ZBP89	ZNF148	0.115985	296
V$ING4_01	ING4	ING4	0.692803	286
V$ELK1_02	Elk-1	ELK1	0.112507	275
V$PBX1_04	Pbx1	PBX1	0.236112	262
V$HIF1A_Q6	HIF-1alpha	HIF1A	0.00335261	259
V$MAZ_Q6_01	MAZ	MAZ	0.224203	257
V$AP4_Q5	AP-4	TFAP4	0.411949	255
V$KLF15_Q2	KLF15	KLF15	0.0387339	223
V$IRF8_Q6	IRF-8	IRF8	0.0927724	216
V$CREM_Q6	CREM	CREM	0.0272754	214
V$CP2_01	CP2	TFCP2	0.250059	175
V$ATF1_Q6_01	ATF-1	ATF1	0.00366055	169
V$SP4_Q5	SP4	SP4	0.039477	165
V$FAC1_01	FAC1	BPTF	0.142423	152
V$NURR1_Q3	NURR1	NR4A2	0.135327	150
V$ATF4_Q6	ATF-4	ATF4	0.0789839	116
V$ATF2_Q5	ATF-2	ATF2	0.0167795	81
V$PAX5_01	Pax-5	PAX5	0.0167537	18

hsa-mir-95

Matrix_id	transcription factor	Gene	PCC	Occurrence
V$PUR1_Q4	PUR1	PURA	0.440369	2045
V$IK_Q5	Ikaros	IKZF1	0.766584	2000
V$PARP_Q4	PARP	PARP1	0.65494	1983
V$PEA3_Q6	PEA3	ETV4	0.752847	1948
V$P300_01	p300	EP300	0.820295	1905
V$MYB_Q6	c-Myb	MYB	0.273058	1749
V$CMYB_Q5	c-Myb	MYB	0.273058	1749
V$SMAD4_Q6_01	Smad4	SMAD4	0.714806	1734
V$TBP_Q6	TBP	TBP	0.219236	1730
V$CDX2_Q5_02	CDX-2	CDX2	0.857089	1730
V$NFAT4_Q3	NF-AT4	NFATC3	0.531771	1713
V$TBX5_02	TBX5	TBX5	0.769689	1680
V$CETS1_Q6	C-ets-1	ETS1	0.381904	1672
V$ETS1_B	c-Ets-1	ETS1	0.381904	1658
V$NR1B2_Q6	NR1B2	RARB	0.688062	1658
V$GATA1_01	GATA-1	GATA1	0.433141	1646
V$ETS2_Q6	c-Ets-2	ETS2	0.680785	1603
V$YY1_01	YY1	YY1	0.466767	1588
V$GABPA_Q4	GABP-alpha	GABPA	0.498469	1587
V$MEF2C_Q4	MEF-2C	MEF2C	0.514804	1583
V$GR_Q6	GR	NR3C1	0.713436	1559
V$DLX5_01	dlx5	DLX5	0.124737	1552
V$NANOG_02	Nanog	NANOG	0.911473	1546
V$Elf5_03	ELF5	ELF5	0.0321759	1529
V$SOX5_01	SOX5	SOX5	0.468268	1498
V$IPF1_01	IPF1	PDX1	0.902338	1497
V$SPI1_03	SPI1	SPI1	0.166441	1483
V$ETS2_B	c-Ets-2	ETS2	0.680785	1433
V$IPF1_Q6	IPF1	PDX1	0.902338	1425
V$YY1_Q6	YY1	YY1	0.466767	1422
V$GATA1_05	GATA-1	GATA1	0.433141	1408
V$GATA1_06	GATA-1	GATA1	0.433141	1408
V$GATA2_02	GATA-2	GATA2	0.369201	1408
V$SPI1_Q5	SPI1	SPI1	0.166441	1386
V$IPF1_Q4_01	IPF1	PDX1	0.902338	1374
V$BEN_01	BEN	GTF2IRD1	0.915067	1369
V$CDX2_Q5_01	Cdx-2	CDX2	0.857089	1366
V$SRY_02	SRY	SRY	0.675973	1361
V$YY1_Q6_02	YY1	YY1	0.466767	1352
V$NKX32_01	Nkx3-2	NKX3-2	0.809063	1308
V$CDX2_01	Cdx-2	CDX2	0.857089	1308
V$PARP_Q3	PARP	PARP1	0.65494	1288

V$FOXO3A_Q1	FOXO3A	FOXO3	0.631679	1283
V$NFAT2_Q5	NF-AT2	NFATC1	0.848469	1262
V$IRF4_Q6	IRF-4	IRF4	0.85156	1252
V$ERBETA_Q5	ER-beta	ESR2	0.697212	1231
V$MAZ_Q6	MAZ	MAZ	0.196312	1228
V$TEL1_02	TEL1	ETV6	0.913405	1184
V$E2A_Q6	E2A	TCF3	0.701162	1179
V$E12_Q6	E12	TCF3	0.701162	1178
V$ELK1_02	Elk-1	ELK1	0.626416	1178
V$E47_02	E47	TCF3	0.701162	1172
V$MYOD_Q6_01	MyoD	MYOD1	0.80511	1170
V$CRX_Q4	Crx	CRX	0.681139	1155
V$ETV3_02	ETV3	ETV3	0.435371	1146
V$IPF1_Q4	IPF1	PDX1	0.902338	1143
V$ER71_02	ER71	ETV2	0.338935	1126
V$YY1_Q6_03	YY1	YY1	0.466767	1119
V$GFI1_Q6	Gfi1	GFI1	0.315518	1074
V$ELK1_06	ELK-1	ELK1	0.626416	1070
V$MYOGENIN_Q6	myogenin	MYOG	0.761651	1068
V$LEF1_Q5	LEF-1	LEF1	0.016484	1068
V$TCF3_01	TCF-3	TCF7L1	0.675858	1067
V$HNF1_02	HNF-1alpha	HNF1A	0.66645	1062
V$GFI1_Q6_01	Gfi1	GFI1	0.315518	1049
V$HIF1A_Q6	HIF-1alpha	HIF1A	0.0885175	1049
V$CEBPE_Q6	CEBPE	CEBPE	0.153452	1042
V$ARNT_01	Arnt	ARNT	0.739741	1038
V$AP2ALPHA_Q6	AP-2alpha	TFAP2A	0.0850891	1024
V$IRF8_Q6	IRF-8	IRF8	0.0216152	998
V$FOXP3_01	FOXP3	FOXP3	0.826706	993
V$FOXM1_01	FOXM1	FOXM1	0.17449	991
V$AP2ALPHA_01	AP-2alpha	TFAP2A	0.0850891	978
V$CMYB_01	c-Myb	MYB	0.273058	964
V$ERF_02	ERF	ERF	0.453723	963
V$PITX2_01	PITX2	PITX2	0.745591	958
V$EHF_03	EHF	EHF	0.224708	944
V$OC2_Q3	OC-2	ONECUT2	0.73376	940
V$CMAF_01	c-Maf	MAF	0.42115	920
V$E2A_Q2	E2A	TCF3	0.701162	919
V$GFI1B_01	Gfi1b	GFI1B	0.857762	910
V$CRX_Q4_01	CRX	CRX	0.681139	883
V$ERR1_Q3	ERR1	ESRRA	0.687804	841
V$ELF5_01	ELF5	ELF5	0.0321759	825
V$BCL6_Q3_01	Bcl-6	BCL6	0.918328	797
V$CEBPD_Q6	C/EBPdelta	CEBPD	0.175743	762
V$NEUROD_02	NeuroD	NEUROD1	0.871325	732
V$E47_01	E47	TCF3	0.701162	722

V$ZIC1_01	Zic1	ZIC1	0.266484	715
V$HOXD9_Q2	Hoxd9	HOXD9	0.592089	694
V$LHX3b_01	LHX3b	LHX3	0.778417	690
V$PIT1_Q6	Pit-1	POU1F1	0.311721	690
V$MYOD_Q6	MyoD	MYOD1	0.80511	690
V$DBP_Q6_01	DBP	DBP	0.880217	679
V$DAX1_01	Dax1	NR0B1	0.550275	659
V$CEBPB_Q6	C/EBPbeta	CEBPB	0.208978	653
V$ERG_03	ERG	ERG	0.72351	641
V$PET1_02	Pet-1	FEV	0.501743	640
V$AML2_01	AML2	RUNX3	0.313156	613
V$NCX_02	Ncx	TLX2	0.763032	591
V$IPF1_02	IPF1	PDX1	0.902338	584
V$HNF6_Q6	HNF6	ONECUT1	0.191297	578
V$CART1_02	CART1	ALX1	0.814604	559
V$HOX13_02	HOXA5	HOXA5	0.0466749	523
V$HOXB8_01	HOXB8	HOXB8	0.521114	510
V$IPF1_06	ipf1	PDX1	0.902338	503
V$MYOD_01	MyoD	MYOD1	0.80511	492
V$IPF1_03	IPF1	PDX1	0.902338	474
V$HTF4_Q2	HTF4	TCF12	0.596694	450
V$SOX2_Q6	SOX2	SOX2	0.274732	391
V$LHX3_01	Lhx3	LHX3	0.778417	367
V$BEN_02	BEN	GTF2IRD1	0.915067	361
V$CEBPB_01	C/EBPbeta	CEBPB	0.208978	330
V$HNF1B_01	HNF-1beta	HNF1B	0.434836	324
V$NUR77_Q5	NUR77	NR4A1	0.515472	269
V$RREB1_01	RREB-1	RREB1	0.731149	234
V$GLI_Q2	GLI	GLI1	0.368034	223
V$GLI2_01	GLI2	GLI2	0.869929	152
V$GLI3_02	GLI3	GLI3	0.774868	148

----

hsa-mir-96

Matrix_id	transcription factor	Gene	PCC	Occurrence
V$ELF1_Q6	Elf-1	ELF1	0.402265	19
V$MYB_Q6	c-Myb	MYB	0.750565	18
V$IK_Q5	Ikaros	IKZF1	0.257737	18
V$CMYB_Q5	c-Myb	MYB	0.750565	18
V$GATA3_01	GATA-3	GATA3	0.13135	12
V$GATA3_02	GATA-3	GATA3	0.13135	12
V$GFI1_Q6_01	Gfi1	GFI1	0.6067	9
V$LEF1_Q5	LEF-1	LEF1	0.839296	9

V$ZABC1_01	ZABC1	ZNF217	0.0426443	7

---

hsa-mir-98

Matrix_id	transcription factor	Gene	PCC	Occurrence
V$PUR1_Q4	PUR1	PURA	0.530866	150
V$AP2REP_01	AP-2rep	KLF12	0.036882	141
V$ELF1_Q6	Elf-1	ELF1	0.00846873	138
V$MAFB_01	MAFB	MAFB	0.104388	137
V$ZIC3_01	Zic3	ZIC3	0.476204	136
V$P300_01	p300	EP300	0.100704	135
V$PARP_Q4	PARP	PARP1	0.449885	135
V$NR1B2_Q6	NR1B2	RARB	0.21982	124
V$SOX9_Q4	SOX9	SOX9	0.300195	123
V$CDX2_Q5_02	CDX-2	CDX2	0.0604623	115
V$TBP_Q6	TBP	TBP	0.0559374	115
V$YY1_01	YY1	YY1	0.313048	110
V$HNF4A_Q6_01	HNF-4alpha	HNF4A	0.256104	107
V$AP4_Q6_02	AP-4	TFAP4	0.550676	106
V$GR_Q6	GR	NR3C1	0.260696	104
V$AML1_Q6	AML1	RUNX1	0.0381445	103
V$SOX5_01	SOX5	SOX5	0.0736912	100
V$MEF2C_Q4	MEF-2C	MEF2C	0.668841	99
V$SOX9_B1	SOX9	SOX9	0.300195	98
V$YY1_Q6	YY1	YY1	0.313048	98
V$IPF1_Q6	IPF1	PDX1	0.147463	94
V$YY1_Q6_02	YY1	YY1	0.313048	94
V$NANOG_02	Nanog	NANOG	0.0691387	94
V$GATA1_05	GATA-1	GATA1	0.116816	92
V$GATA1_06	GATA-1	GATA1	0.116816	92
V$IPF1_Q4_01	IPF1	PDX1	0.147463	91
V$PARP_Q3	PARP	PARP1	0.449885	90
V$GATA1_02	GATA-1	GATA1	0.116816	90
V$HIF1A_Q6	HIF-1alpha	HIF1A	0.154434	90
V$TFIII_Q6	TFII-I	GTF2I	0.484154	90
V$CDX2_01	Cdx-2	CDX2	0.0604623	88
V$CDX2_Q5_01	Cdx-2	CDX2	0.0604623	88
V$SRY_02	SRY	SRY	0.129506	87
V$IPF1_01	IPF1	PDX1	0.147463	86
V$ELK1_02	Elk-1	ELK1	0.353587	85
V$TEL1_02	TEL1	ETV6	0.0655315	85
V$NKX32_01	Nkx3-2	NKX3-2	0.0646674	82
V$HNF1_02	HNF-1alpha	HNF1A	0.045051	80

V$PBX1_Q3	Pbx1	PBX1	0.328616	80
V$NFAT2_Q5	NF-AT2	NFATC1	0.0286644	80
V$YY1_Q6_03	YY1	YY1	0.313048	79
V$TCF3_01	TCF-3	TCF7L1	0.088662	77
V$GATA1_04	GATA-1	GATA1	0.116816	76
V$FOXO3A_Q1	FOXO3A	FOXO3	0.175065	76
V$PBX1_04	Pbx1	PBX1	0.328616	75
V$CREM_Q6	CREM	CREM	0.197366	74
V$CMYB_01	c-Myb	MYB	0.129934	67
V$FOXJ2_01	FOXJ2	FOXJ2	0.119345	66
V$GFI1_Q6	Gfi1	GFI1	0.171663	66
V$GFI1_Q6_01	Gfi1	GFI1	0.171663	64
V$LEF1_Q5	LEF-1	LEF1	0.045179	63
V$SOX10_Q6	SOX10	SOX10	0.626052	59
V$OC2_Q3	OC-2	ONECUT2	0.224795	57
V$SREBP1_Q6	SREBP-1	SREBF1	0.234367	57
V$BCL6_Q3_01	Bcl-6	BCL6	0.0800593	56
V$ATF1_Q6_01	ATF-1	ATF1	0.114852	56
V$AML1_01	AML1a	RUNX1	0.0381445	55
V$CMAF_01	c-Maf	MAF	0.240619	53
V$GR_01	GR	NR3C1	0.260696	53
V$NURR1_Q3	NURR1	NR4A2	0.314579	51
V$GFI1B_01	Gfi1b	GFI1B	0.023968	51
V$NKX32_02	Nkx3-2	NKX3-2	0.0646674	50
V$PAX8_01	Pax-8	PAX8	0.23018	49
V$FAC1_01	FAC1	BPTF	0.0432653	46
V$DAX1_01	Dax1	NR0B1	0.0763903	46
V$LHX3b_01	LHX3b	LHX3	0.252668	45
V$HOXA9_01	hoxa9	HOXA9	0.190252	45
V$NKX2B_Q3	NKX2B	NKX2-2	0.725863	43
V$DEC2_Q2	2-Dec	BHLHE41	0.582114	41
V$ATF4_Q6	ATF-4	ATF4	0.135251	41
V$NKX22_02	NKX2B	NKX2-2	0.725863	40
V$HOXD9_Q2	Hoxd9	HOXD9	0.153101	39
V$CART1_02	CART1	ALX1	0.151738	38
V$HNF6_Q6	HNF6	ONECUT1	0.0205047	37
V$HOX13_02	HOXA5	HOXA5	0.116842	36
V$HOXB8_01	HOXB8	HOXB8	0.565587	34
V$IPF1_06	ipf1	PDX1	0.147463	34
V$NCX_02	Ncx	TLX2	0.0515589	33
V$IPF1_03	IPF1	PDX1	0.147463	33
V$ATF2_Q5	ATF-2	ATF2	0.204797	27
V$LHX3_01	Lhx3	LHX3	0.252668	27
V$USF2_Q6	USF2	USF2	0.471535	25
V$NMYC_01	N-Myc	MYCN	0.0147076	25
V$SOX2_Q6	SOX2	SOX2	0.544709	25

V$MAX_01	Max	MAX	0.664848	25
V$STAT4_Q5	STAT4	STAT4	0.157358	24
V$CDP_04	CDP	CUX1	0.201871	22
V$HNF1B_01	HNF-1beta	HNF1B	0.235226	20
V$HSF1_01	HSF1	HSF1	0.0820563	16
V$SREBP1_01	SREBP-1	SREBF1	0.234367	12
V$POU6F1_01	POU6F1	POU6F1	0.593997	11
V$EVI1_06	Evi-1	MECOM	0.0641628	6

---

hsa-mir-99b

Matrix_id	transcription factor	Gene	PCC	Occurrence
V$PUR1_Q4	PUR1	PURA	0.242859	174
V$PARP_Q4	PARP	PARP1	0.140581	170
V$ELF1_Q6	Elf-1	ELF1	0.0332038	167
V$GKLF_Q4	GKLF	KLF4	0.513914	165
V$ETS1_B	c-Ets-1	ETS1	0.00359139	157
V$ZIC3_01	Zic3	ZIC3	0.00854804	156
V$SMAD4_Q6_01	Smad4	SMAD4	0.207056	154
V$CETS1_Q6	C-ets-1	ETS1	0.00359139	151
V$GATA1_01	GATA-1	GATA1	0.0176781	146
V$GABPA_Q4	GABP-alpha	GABPA	0.26928	144
V$GR_Q6	GR	NR3C1	0.0845845	131
V$YY1_01	YY1	YY1	0.366003	129
V$TBP_Q6	TBP	TBP	0.217618	129
V$MAZ_Q6	MAZ	MAZ	0.0145113	121
V$GATA1_02	GATA-1	GATA1	0.0176781	114
V$GATA6_01	GATA-6	GATA6	0.120907	112
V$GATA2_02	GATA-2	GATA2	0.585385	112
V$GATA1_06	GATA-1	GATA1	0.0176781	112
V$GATA1_05	GATA-1	GATA1	0.0176781	112
V$PARP_Q3	PARP	PARP1	0.140581	110
V$AHR_Q5	AhR	AHR	0.850182	109
V$AP2ALPHA_Q6	AP-2alpha	TFAP2A	0.837976	105
V$SP1_Q6	Sp1	SP1	0.250004	103
V$ING4_01	ING4	ING4	0.223996	103
V$FKLF_Q5	FKLF	KLF11	0.226701	99
V$GATA3_01	GATA-3	GATA3	0.793724	97
V$MAZ_Q6_01	MAZ	MAZ	0.0145113	95
V$TFIII_Q6	TFII-I	GTF2I	0.0742778	95
V$GATA1_04	GATA-1	GATA1	0.0176781	94
V$SP1_Q6_01	Sp1	SP1	0.250004	93
V$AP2ALPHA_01	AP-2alpha	TFAP2A	0.837976	92

V$GATA3_02	GATA-3	GATA3	0.793724	92
V$YY1_Q6_03	YY1	YY1	0.366003	89
V$SP1_Q4_01	Sp1	SP1	0.250004	89
V$SP1_Q2_01	Sp1	SP1	0.250004	88
V$GATA2_01	GATA-2	GATA2	0.585385	87
V$SP1_01	Sp1	SP1	0.250004	85
V$SP1_02	SP1	SP1	0.250004	82
V$GR_01	GR	NR3C1	0.0845845	82
V$ELK1_02	Elk-1	ELK1	0.118397	77
V$CP2_01	CP2	TFCP2	0.595874	74
V$TCF4_Q5	TCF-4	TCF7L2	0.414844	72
V$FOXJ2_01	FOXJ2	FOXJ2	0.0448589	72
V$ELK1_06	ELK-1	ELK1	0.118397	69
V$ZABC1_01	ZABC1	ZNF217	0.688671	66
V$CREM_Q6	CREM	CREM	0.300086	66
V$GABPBETA_Q3	GABP-beta	GABPB1	0.220132	62
V$SP2_01	SP2	SP2	0.173129	61
V$ESE1_Q3	ESE-1	ELF3	0.0323613	61
V$P53_02	p53	TP53	0.39835	48
V$ATF3_Q6_01	ATF-3	ATF3	0.423389	47
V$E2F4_Q6	E2F-4	E2F4	0.0132877	47
V$RNF96_01	RNF96	TRIM28	0.366368	46
V$PDEF_02	PDEF	SPDEF	0.00071877	45
V$CIZ_01	CIZ	ZNF384	0.103151	40
V$ELK1_01	Elk-1	ELK1	0.118397	36
V$EGR2_01	Egr-2	EGR2	0.00873037	34
V$SMAD4_Q6	SMAD4	SMAD4	0.207056	27
V$GLI3_Q5_01	GLI3	GLI3	0.173122	24
